# Supplementary material for: Photo-induced trifunctionalization of bromostyrenes via remote radical migration reactions of tetracoordinate boron species
Source: Nat Commun. 2022 Apr 4;13:1784. doi: 10.1038/s41467-022-29466-3 (PMC8980057; doi:10.1038/s41467-022-29466-3)
Supplement: Supplementary file 1 — Supplementary information [file 41467_2022_29466_MOESM1_ESM.pdf]

Supporting Information

Photo-induced Trifunctionalization of Bromostyrenes via Remote Radical Migration Reactions of Tetracoordinate Boron Species

Chaokun Li,[1] Shangteng liao,[1] Shanglin Chen,[1] Nan Chen,[1] Feng Zhang,[1] Kai Yang[1] and Qiuling Song[1,2,3]*

[1] *Key Laboratory of Molecule Synthesis and Function Discovery, Fujian Province University, College of Chemistry and College of Materials Science at Fuzhou University Fuzhou, Fujian, 350108 (China)*

[2] *Institute of Next Generation Matter Transformation, College of Materials Science Engineering at Huaqiao University 668 Jimei Boulevard, Xiamen, Fujian, 361021 (China)*

[3] *School of Chemistry and Chemical Engineering, Henan Normal University, Xinxiang, Henan, 453007 (China)*

E-mail: qsong@hqu.edu.cn

Contents

1. Supplementary Methods 3
1.1 General information 3
1.2 Blue LED reactors 3
2. Supplementary Discussion 4
2.1 Optimization of Experimental Conditions 4
2.2. General Procedures 5
2.2.1 General procedure A for preparation of 3° alkyl-Bpin 5
2.2.2 General procedure B for preparation of 1,2-diborons 6
2.2.3 General procedure C for preparation of 1,1-diborons 7
2.2.4 General procedure D_1 for remote radical migration reactions 8
2.2.5 General procedure D_2 for remote radical migration reactions 8
2.3. Characterization of Starting Materials 9
2.4. Characterization of Products 15
2.5. Scale-up Reactions and Synthetic Applications 53
2.5.1 10 times scale-up reactions. 53
2.5.2 Synthetic applications.. 54
2.6. Control Experiments 61
2.6.1 Radical capture experiment 61
2.6.2 Deuteration experiments. 62
2.6.3 Radical-clock experiments. 63
2.6.4 Validation experiment. 64
2.6.5 Cross-over experiment. 65
2.7. Cyclic voltammetry measurement 66
2.8. Luminescence quenching experiment 66
2.9. NMR spectroscopic data 68
2.10. Supplementary references 191

1. Supplementary Methods

1.1 General information

All experiments were conducted with a Schlenk tube under an argon atmosphere. Flash column chromatography was performed over silica gel (200-300 mesh). Analytical thin-layer chromatography (TLC) was carried out on Merck 60 F_{254} pre-coated silica gel plate (0.2 mm thickness). Visualization was accomplished by UV light (254 nm), phosphomolybdic acid or $KMnO_4$ staining solutions followed by heating, also by Gas Chromatograph-Mass spectrometer analysis (GC-MS). Unless otherwise noted, materials obtained from commercial suppliers were used without further purification.
^{1}H NMR, ^{11}B NMR, ^{13}C NMR and ^{19}F NMR spectra were recorded at ambient temperature using Bruker Ascend^{TM} 400 (400 MHz) spectrometer, Bruker AVANCE III 500M spectrometers or JNM-ECZ500R/S1 (500 MHz) spectrometer. ^{1}H NMR chemical shifts (in ppm) were referenced to $CDCl_3$ (δ = 7.26 ppm) as internal standards. ^{13}C NMR spectra were obtained by using the same NMR spectrometers and were calibrated with $CDCl_3$ (δ = 77.0 ppm). The following abbreviations are used: s = singlet, d = doublet, t = triplet, q = quartet, dd, = double doublet, dt = double triplet, td = triple doublet, m = multiplet. HRMS data were obtained on Thermo Scientific Orbitrap Elite Mass Spectrometer with an ESI source (Ion Trap) or Agilent 7820A GC-MS with EI mode.

1.2 Blue LED reactors

Visible light irradiation was performed with a 20 W LED lamp (at λ_{ir} = 450 ± 10 nm) for photocatalytic reactions. All photoredox reactions were carried out at room temperature. Fan assisted cooling was used to maintain this temperature.

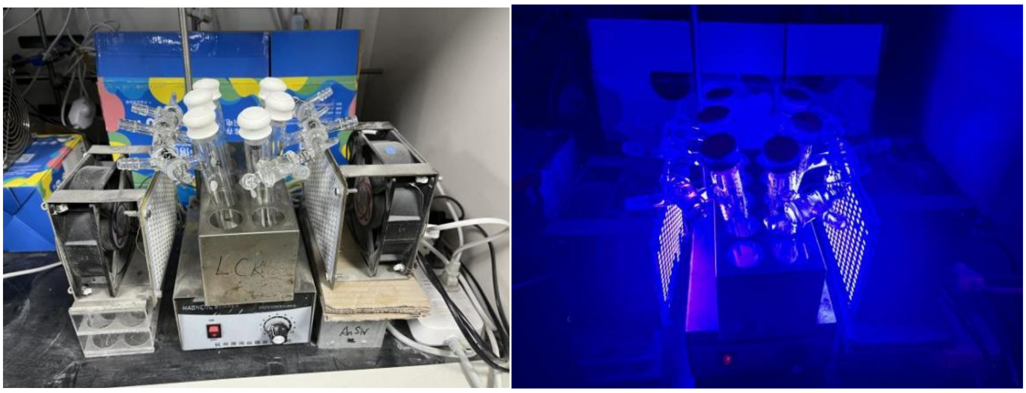

Supplementary Figure 1. Blue LED reactors

2. Supplementary Discussion

2.1 Optimization of Experimental Conditions

Supplementary Table 1. The effects of proton source

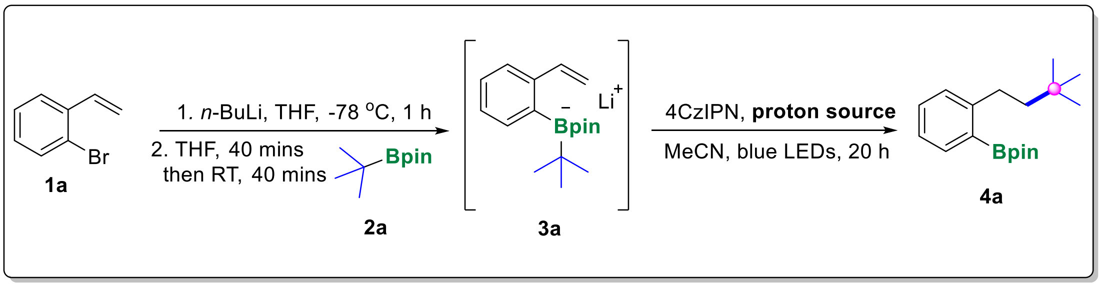

Entry	**proton source (3.0 equiv)**	Yield of **4a** (%)[b]
1	*t*-BuOH	28
2	MeOH	26
3	*i*-PrOH	5
4	$HSCH_2CO_2Me$	14
5	CH_3CH_2OH	33
6	PhCOOH	15
7	H_2O	37
8	TFE	55
9	HFIP	66

Reaction conditions: To a solution of *o*-bromostyrene **1a** (0.22 mmol, 1.1 equiv) in THF (0.6 mL) was added *n*-BuLi (0.22 mmol, 1.1 equiv) at -78 °C under Ar, the resulting mixture was stirred at -78 °C for 1 h, then **2a** (0.2 mmol, 1.0 equiv) in THF (0.2 ml) was added and stirred at -78 °C for 40 mins and warm to room temperature for 40 mins, followed by **proton source (0.6mmol, 3.0 equiv)** and 4CzIPN (2 mol%) in MeCN (2 mL, 0.1 M). The resulting mixture was irradiated by blue LEDs for 20 hours.

Supplementary Table 2. The effects of amount of HFIP

Entry	**HFIP (X equiv)**	Yield of **4a** (%)[b]
1	4.0	50
2	5.0	80(74)
3	6.0	72

Reaction conditions: To a solution of *o*-bromostyrene **1a** (0.22 mmol, 1.1 equiv) in THF (0.6 mL) was added *n*-BuLi (0.22 mmol, 1.1 equiv) at -78 °C under Ar, the resulting mixture was stirred at -78 °C for 1 h, then **2a** (0.2 mmol, 1.0 equiv) in THF (0.2 ml) was added and stirred at -78 °C for 40 mins and warm to room temperature for 40 mins, followed by **HFIP (X equiv)** and 4CzIPN (2 mol%) in MeCN (2 mL, 0.1 M). The resulting mixture was irradiated by blue LEDs for 20 hours.

Supplementary Table 3. The effects of solvent

Entry	**Solvent**	Yield of **4a** (%)[b]
1	DMF	8
2	DMSO	37

Reaction conditions: To a solution of *o*-bromostyrene **1a** (0.22 mmol, 1.1 equiv) in THF (0.6 mL) was added *n*-BuLi (0.22 mmol, 1.1 equiv) at -78 °C under Ar, the resulting mixture was stirred at -78 °C for 1 h, then **2a** (0.2 mmol, 1.0 equiv) in THF (0.2 ml) was added and stirred at -78 °C for 40 mins and warm to room temperature for 40 mins, followed by HFIP (1.0 mmol, 5.0 equiv) and 4CzIPN (2 mol%) in **solvent (2 mL, 0.1 M)**. The resulting mixture was irradiated by blue LEDs for 20 hours.

Supplementary Table 4. The effects of photocatalyst

Entry	Photocatalyst (2 mol%)	Yield of **4a** (%)[b]
1	Ir[dF(CF_3)ppy)]$_2$(bpy)PF_6	26
2	Ru(bpy)$_3$(PF_6)$_2$	3
3	Ir[dF(CF_3)ppy)]$_2$(dtbpy)PF_6	33

Reaction conditions: To a solution of *o*-bromostyrene **1a** (0.22 mmol, 1.1 equiv) in THF (0.6 mL) was added *n*-BuLi (0.22 mmol, 1.1 equiv) at -78 ºC under Ar, the resulting mixture was stirred at -78 ºC for 1 h, then **2a** (0.2 mmol, 1.0 equiv) in THF (0.2 ml) was added and stirred at -78 ºC for 40 mins and warm to room temperature for 40 mins, followed by HFIP (1.0 mmol, 5.0 equiv) and **photocatalyst (2 mol%)** in MeCN (2 mL, 0.1 M). The resulting mixture was irradiated by blue LEDs for 20 hours.

Supplementary Table 5 . The effects of 1a and *n*-BuLi

Entry	1a/*n*-BuLi (X equiv)	Yield of **4a** (%)[b]
1	1.2	76
2	1.3	86 (81[c])
3	1.4	65
4	1.5	57
5	1.3 **1a/*n*-BuLi** + 5 mol% 4CzIPN	90

Reaction conditions: Reaction conditions: To a solution of *o*-bromostyrene **1a (X equiv)** in THF (0.6 mL) was added *n*-BuLi **(X equiv)** at -78 ºC under Ar, the resulting mixture was stirred at -78 ºC for 1 h, then **2a** (0.2 mmol, 1.0 equiv) in THF (0.2 ml) was added and stirred at -78 ºC for 40 mins and warm to room temperature for 40 mins, followed by HFIP (1.0 mmol, 5.0 equiv) and 4CzIPN (2 mol%) in MeCN (2 mL, 0.1 M). The resulting mixture was irradiated by blue LEDs for 20 hours.

2.2 General procedures

The synthesis of tertiary alkyl boronic esters **2b**[4], **2c**[1], **2d**[4], **2e**[2], **2f**[4], **2h-2n**[4], **2ah**[4], **2ai**[2], **2aj**[4], **2ak**[4] used in this work were prepared according to the methods reported in literature. Secondary alkyl boronic esters **2ag**[3] used in this work were prepared according to the methods reported in literature. 1,2-bis-boronic esters compounds **5a**[5], **5c-5g**[5], **5b**[6] were prepared according to the reference. *Gem*-bis-boronic esters compounds **5'a-5'i**[7] were prepared according to the reference. Unless otherwise noted, materials obtained from commercial suppliers were used without further purification.

2.2.1 General procedure A for preparation of 3º alkyl-Bpin

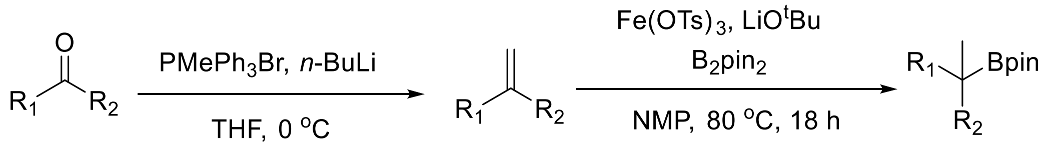

Supplementary Figure 2. Synthetic methods for 3º alkyl-Bpin

Tertiary alkyl boronic esters compounds **2b**[4], **2d**[4], **2f**[4], **2h-2n**[4], **2ah**[4], **2aj**[4], **2ak**[4] were

prepared according to the reference.

Step 1: To an oven-dried 100 mL Schlenk flask equipped with a magnetic stir bar, $PMePh_3Br$ (1.8 equiv, 12.8 mmol) was added and the flask was evacuated and refilled with argon (three times). Dry THF (40 mL) was added and the reaction was placed in the ice bath. Then, *n*-BuLi (1.6 M in hexane, 12.5mmol) was dropped over 10 min into the flask. After 0.5 h, a solution of ketone substrates (8.5 mmol) in 10 ml of THF was added and the reaction mixture was allowed to warm up to RT. After overnight resting, the mixture was quenched with an aqueous saturated solution of NH_4Cl, extracted with EtOAc (three times), dried over Na_2SO_4 and evaporated to dryness in vacuo. The crude product was purified by flash chromatography to give corresponding product.

Step 2: To a 50 mL Schlenk flask equipped with a magnetic stir bar, $Fe(OTs)_3$ (142.4 mg, 0.125mmol, 2.5 mol%), LiO^tBu (1.2 g, 15mmol) and B_2pin_2 (3.18g, 12.5mmol) was added and the flask was evacuated and refilled with argon (three times). The tube was evacuated and backfilled with argon for three times, and then NMP (25mL) and alkene (5 mmol) was added by syringe under argon flow. The reaction mixture was stirred and heated at 80 °C for 18 h. Then reaction was cooled to room temperature. Et_2O (50 mL) and water were added and the layers were separated. The aqueous phase was extracted with Et_2O (50 mL x 2) and the combined organic layers were dried over Na_2SO_4 and concentrated. The residue was purified by flash column chromatography on silica gel to give corresponding product.

2.2.2 General procedure B for preparation of 1,2-diborons

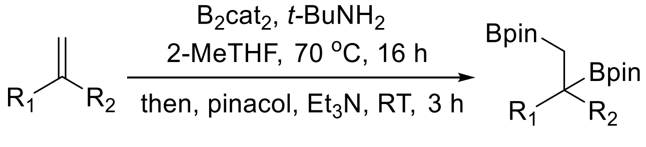

Supplementary Figure 3. Synthetic methods for 1,2-diborons

1,2-bis-boronic esters compounds **5a**[5], **5c-5g**[5] were prepared according to the reference.

1,2-bis-boronic esters compounds **5b**[6] were prepared according to the reference.

To a 50 mL Schlenk flask equipped with a magnetic stir bar, B_2cat_2 was added and the flask was evacuated and refilled with argon (three times). *t*-$BuNH_2$ and 2-MeTHF (4

mL) were added and the reaction stirred at 70 °C for 16 h. Then reaction was cooled to room temperature. Pinacol and Et_3N were added and the reaction stirred at room temperature for 3 h. The aqueous phase was extracted with EtOAc (three times) and the combined organic layers were dried over Na_2SO_4 and concentrated.The residue was purified by flash column chromatography on silica gel to give corresponding product.

2.2.3 General procedure C for preparation of 1,1-diborons

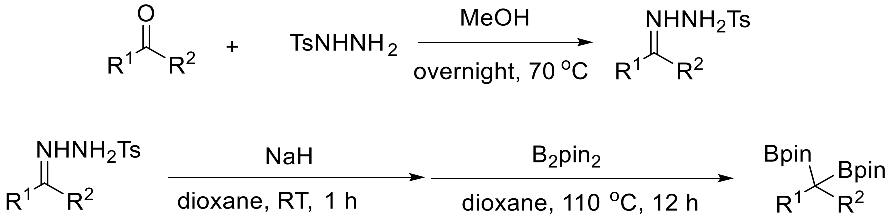

Supplementary Figure 4. Synthetic methods for 1,1-diborons

Gem-bis-boronic esters compounds **5'a-5'i**[7] were prepared according to the reference. To a 50 mL Schlenk flask equipped with a magnetic stir bar, carbonyl compound (5 mmol) and tosylhydrazine (5 mmol) was added and the flask was evacuated and refilled with argon (three times). Methanol (5 mL) were added and the reaction stirred at 70 °C overninght. Then the solution was cooled down, and N-tosylhydrazone precipitated. The precipitate was collected, washed with petroleum ether (5 mL×3), and dried in vacuum. Then, a modified Schlenk tube was charged with N-tosylhydrazone (5 mmol), 60% NaH (6 mmol), and BTMAC (0.5 mmol). After degassed and filled with argon, the tube was charged with dioxane (35 mL). The mixture was stirred at room temperature for 1 h. Then a solution of B_2pin_2 (3 mmol) dioxane (5 mL) was added via syringe. Then the tube was sealed and heated at 110 °C for 12 h. After cooled to room temperature, Et_2O and H_2O were added. The mixture was stirred vigorously for 10 minutes. After separation of organic layer, the aqueous layer was extracted with Et_2O. The combined organic solution was washed with saturated brine and dried over anhydrous Na_2SO_4. After the solvent was evaporated, the crude product was purified by silica gel chromatography.

2.2.4 General procedure D$_1$ for remote radical migration reactions

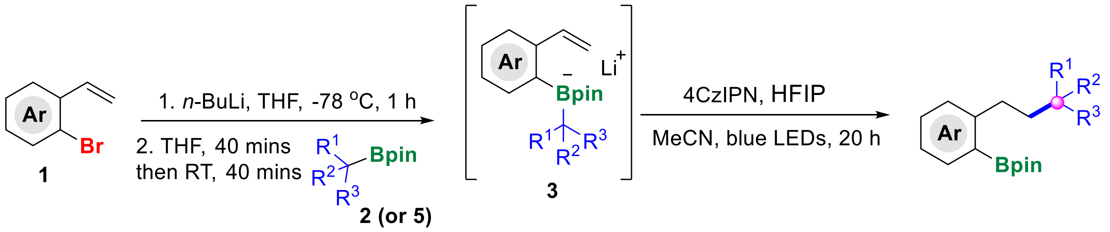

Supplementary Figure 5. Synthetic methods for aryl boronic acid pinocol esters

To a flame-dried Schlenk tube were added bromostyrene (**1**, 0.26 mmol, 1.3 equiv) and THF (0.6 mL) and the resulting solution was cooled to -78 °C. Subsequently, *n*-BuLi (0.16 mL, 1.6 M in hexane, 0.26 mmol, 1.3 equiv) was added dropwise and the resulting mixture was stirred at -78 °C for 1 h, before dropwise addition of **2 (or 5 or 5`)** (0.2 mmol, 1.0 equiv, in 0.2 mL THF) to the solution of aryllithium reagent. And the resulting mixture was stirred at -78 °C for 40 mins, then allowed it to ambient temperature and stirred for another 40 mins. Next, without removing the THF (with removing the THF when *gem*-diborons as substrate), the 4CzIPN (3.2 mg, 0.004 mmol, 2 mol%), HFIP (105 μL, 1.0 mmol, 5.0 equiv), MeCN (2 mL, 0.1 M) was added to Schlenk tube under Ar, after which the Schlenk tube was sealed with parafilm and the mixture was stirred vigorously under blue LED irradiation for 20 h. The reaction mixture was diluted with EtOAC and the solution washed with saturated aqueous NH_4Cl, water and brine. The combined organic layers were dried over Na_2SO_4 and concentrated under reduced pressure. The crude product was then purified by flash column chromatography.

2.2.5 General procedure D$_2$ for remote radical migration reactions

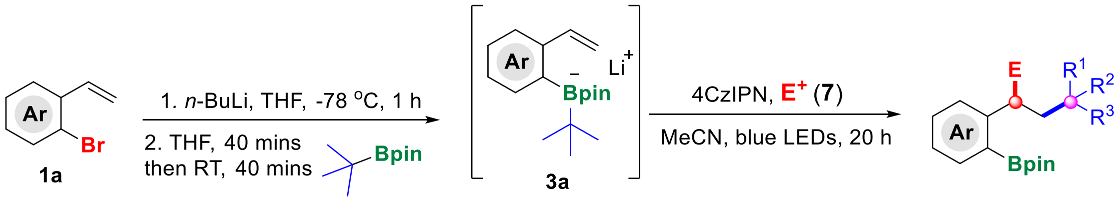

Supplementary Figure 6. Synthetic methods for aryl boronic acid pinocol esters

To a flame-dried Schlenk tube were added *o*-bromostyrene (**1a**, 0.26 mmol, 1.3 equiv) and THF (0.6 mL) and the resulting solution was cooled to -78 °C. Subsequently, *n*-

BuLi (0.16 mL, 1.6 M in hexane, 0.26 mmol, 1.3 equiv) was added dropwise and the resulting mixture was stirred at -78 ^{o}C for 1 h, before dropwise addition of *t*-BuBPin (**2a**, 0.2 mmol, 1.0 equiv, in 0.2 mL THF) to the solution of aryllithium reagent. And the resulting mixture was stirred at -78 ^{o}C for 40 mins, then allowed it to ambient temperature and stirred for another 40 mins. Next, without removing the THF (with removing the THF when *gem*-diborons as substrate), the 4CzIPN (3.2 mg, 0.004 mmol, 2 mol%), electrophile (**7**, 1.0 mmol, 5.0 equiv), MeCN (2 mL, 0.1 M) was added to Schlenk tube under Ar, after which the Schlenk tube was sealed with parafilm and the mixture was stirred vigorously under blue LED irradiation for 20 h. The reaction mixture was diluted with EtOAC and the solution washed with saturated aqueous $NaHCO_3$, water and brine upon completion. The combined organic layers were dried over Na_2SO_4 and concentrated under reduced pressure. The crude product was then purified by flash column chromatography.

2.3 Characterization of Starting Materials

4,4,5,5-tetramethyl-2-(2-methyl-4-phenylbutan-2-yl)-1,3,2-dioxaborolane (2i)

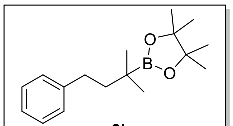

2i

Following the **procedure A** on 5 mmol scale, colorless oil liquid, yield: 58% (794.6 mg), R_f = 0.45 (silica gel, PE: EtOAC = 20:1, v/v), column chromatography (silica gel, PE: EtOAC = 50:1, v/v).

^{1}H NMR (500 MHz, $CDCl_3$) δ 7.28 (t, *J* = 5.1 Hz, 2H), 7.22 – 7.15 (m, 3H), 2.63 – 2.54 (m, 2H), 1.62 – 1.57 (m, 2H), 1.27 (s, 12H), 1.01 (s, 6H).

^{13}C NMR (126 MHz, $CDCl_3$) δ 143.64 (s), 128.31 (s), 128.20 (s), 125.43 (s), 82.98 (s), 43.49 (s), 33.05 (s), 24.75 (s), 24.73 (s).

^{11}B NMR (128 MHz, $CDCl_3$) δ 35.15 (s).

HRMS (EI-QTOF) *m/z*: $[M]^+$ Calcd. for $C_{17}H_{27}BO_2$ 274.2104; Found 274.2110.

4,4,5,5-tetramethyl-2-((2R)-2,6,6-trimethylbicyclo[3.1.1]heptan-2-yl)-1,3,2-dioxaborolane (2ah)

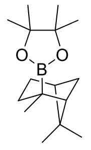

2ah

Following the **procedure A** on 5 mmol scale, colorless oil liquid, yield: 46% (607.2 mg), R_f = 0.5 (silica gel, PE: EtOAC = 20:1, v/v), column chromatography (silica gel, PE: EtOAC = 50:1, v/v).

^{1}H NMR (500 MHz, CDCl$_3$) δ 2.31 (dtd, *J* = 8.0, 6.5, 1.9 Hz, 1H), 2.22 – 2.13 (m, 1H), 1.99 – 1.91 (m, 1H), 1.88 (dd, *J* = 10.7, 4.4 Hz, 1H), 1.81 (tdd, *J* = 13.3, 6.6, 2.6 Hz, 2H), 1.48 (ddd, *J* = 15.1, 11.1, 6.2 Hz, 1H), 1.25 (s, 1H), 1.21 (s, 6H), 1.20 (s, 6H), 1.17 (s, 3H), 1.06 (s, 3H), 1.05 (s, 3H).

^{13}C NMR (101 MHz, CDCl$_3$) δ 82.89 (s), 48.66 (s), 40.78 (s), 33.57 (s), 28.43 (s), 26.85 (s), 25.01 (s), 24.63 (s), 24.56 (s), 23.67 (s).

^{11}B NMR (128 MHz, CDCl$_3$) δ 35.00 (s).

HRMS (EI-QTOF) *m/z*: $[M]^+$ Calcd. for $C_{16}H_{29}BO_2$ 264.2261; Found 264.2258.

2-(1-(benzo[d][1,3]dioxol-5-yl)-2-methylpropan-2-yl)-4,4,5,5-tetramethyl-1,3,2-dioxaborolane (2aj)

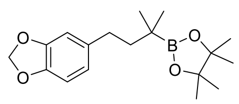

2aj

Following the **procedure A** on 5 mmol scale, white solid (m. p. = 67.1-69.0 ºC), yield: 68% (1.08 g), R_f = 0.45 (silica gel, PE: EtOAC = 20:1, v/v), column chromatography (silica gel, PE: EtOAC = 50:1,v/v).

^{1}H NMR (500 MHz, CDCl$_3$) δ 6.71 (d, *J* = 7.9 Hz, 1H), 6.69 (d, *J* = 1.6 Hz, 1H), 6.62 (dd, *J* = 7.8, 1.7 Hz, 1H), 5.90 (s, 2H), 2.50 – 2.43 (m, 2H), 1.55 – 1.50 (m, 2H), 1.25 (s, 12H), 0.98 (s, 6H).

^{13}C NMR (126 MHz, CDCl$_3$) δ 147.37 (s), 145.26 (s), 137.54 (s), 120.83 (s), 108.86 (s), 107.99 (s), 100.60 (s), 82.96 (s), 43.78 (s), 32.82 (s), 24.71 (s). *overlapped*

^{11}B NMR (128 MHz, CDCl$_3$) δ 35.25 (s).

HRMS (EI-QTOF) *m/z*: $[M]^+$ Calcd. for $C_{18}H_{27}BO_4$ 318.2002; Found 318.2008.

2-(3-(4-isobutylphenyl)-2-methylbutan-2-yl)-4,4,5,5-tetramethyl-1,3,2-dioxaborolane (2ak)

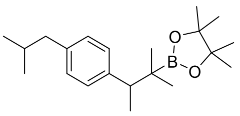

2ak

Following the **procedure A** on 5 mmol scale, colorless oil liquid, yield: 51% (841.5 mg), R_f = 0.4 (silica gel, PE: EtOAC = 20:1, v/v), column chromatography (silica gel, PE: EtOAC = 60:1, v/v).

^{1}H NMR (500 MHz, CDCl$_3$) δ 7.10 (d, *J* = 8.0 Hz, 2H), 7.02 (d, *J* = 8.0 Hz, 2H), 2.82 (q, *J* = 7.2 Hz, 1H), 2.43 (d, *J* = 7.1 Hz, 2H), 1.27 (d, *J* = 7.3 Hz, 3H), 1.24 (s, 6H), 1.21 (s, 6H), 0.90 (d, *J* = 1.1 Hz, 3H), 0.89 (d, *J* = 1.4 Hz, 3H), 0.87 (s, 3H), 0.84 (s, 3H).

^{13}C NMR (126 MHz, CDCl$_3$) δ 141.72 (s), 138.92 (s), 128.75 (s), 128.10 (s), 82.89 (s), 45.65 (s), 45.04 (s), 30.21 (s), 24.81 (s), 22.42 (s), 20.50 (s), 17.23 (s).

^{11}B NMR (128 MHz, CDCl$_3$) δ 34.46 (s).

HRMS (EI-QTOF) *m/z*: $[M]^+$ Calcd. for $C_{21}H_{35}BO_2$ 330.2730; Found 330.2733.

2,2'-(2-ethylbutane-1,2-diyl)bis(4,4,5,5-tetramethyl-1,3,2-dioxaborolane) (5a)

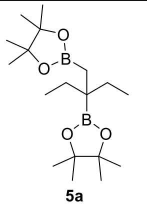

5a

Following the **procedure B** on 4 mmol scale, colorless oil liquid, yield: 62% (838.2 mg), R_f = 0.4 (silica gel, PE: EtOAC = 20:1, v/v), column chromatography (silica gel, PE: EtOAC = 60:1, v/v).

^{1}H NMR (500 MHz, CDCl$_3$) δ 1.43 (qd, *J* = 7.4, 1.3 Hz, 4H), 1.24 (s, 12H), 1.21 (s, 12H), 0.83 (s, 2H), 0.80 (t, *J* = 7.5 Hz, 6H).

^{13}C NMR (126 MHz, CDCl$_3$) δ 82.77 (s), 82.59 (s), 29.10 (s), 24.88 (s), 24.83 (s), 9.33 (s).

^{11}B NMR (128 MHz, CDCl$_3$) δ 34.71 (s).

HRMS (EI-QTOF) *m/z*: [M]$^+$ Calcd. for $C_{18}H_{36}B_2O_4$ 338.2800; Found 338.2804.

2,2'-(2-methylpentane-1,2-diyl)bis(4,4,5,5-tetramethyl-1,3,2-dioxaborolane) (5c)

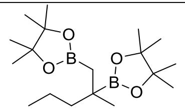

5c

Following the **procedure B** on 4 mmol scale, colorless oil liquid, yield: 56% (757.1 mg), R_f = 0.45 (silica gel, PE: EtOAC = 20:1, v/v), column chromatography (silica gel, PE: EtOAC = 60:1, v/v).

^{1}H NMR (400 MHz, CDCl$_3$) δ 1.32 – 1.24 (m, 2H), 1.19 (s, 12H), 1.17 (s, 12H), 0.93 (s, 0.5H), 0.91 (ss, 0.5H), 0.90 (s, 3H), 0.83 – 0.80 (m, 3H), 0.64 (s, 0.5H), 0.61 (s, 0.5H).

^{13}C NMR (126 MHz, CDCl$_3$) δ 82.75 (s), 82.64 (s), 44.51 (s), 24.95 (s), 24.69 (s), 23.86 (s), 18.88 (s), 15.00 (s).

^{11}B NMR (160 MHz, CDCl$_3$) δ 34.19 (s).

HRMS (EI-QTOF) *m/z*: [M]$^+$ Calcd. for $C_{18}H_{36}B_2O_4$ 338.2800; Found 338.2806.

2,2'-(2-methylhexane-1,2-diyl)bis(4,4,5,5-tetramethyl-1,3,2-dioxaborolane) (5d)

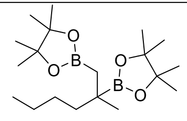

5d

Following the **procedure B** on 4 mmol scale, colorless oil liquid, yield: 63% (887.1 mg), R_f = 0.5 (silica gel, PE: EtOAC = 20:1, v/v), column chromatography (silica gel, PE: EtOAC = 60:1, v/v).

^{1}H NMR (500 MHz, CDCl$_3$) δ 1.36 – 1.28 (m, 6H), 1.21 (s, 12H), 1.19 (s, 12H), 1.16 (s, 2H), 0.93 (s, 3H), 0.84 (t, *J* = 6.9 Hz, 3H).

^{13}C NMR (126 MHz, CDCl$_3$) δ 82.76 (s), 82.65 (s), 41.69 (s), 27.98 (s), 24.96 (s), 24.69 (s), 23.87 (s), 23.54 (s), 14.11 (s).

^{11}B NMR (128 MHz, CDCl$_3$) δ 33.75 (s).

HRMS (EI-QTOF) *m/z*: $[M]^+$ Calcd. for $C_{19}H_{38}B_2O_4$ 352.2956; Found 352.2954.

2,2'-(2-cyclohexylpropane-1,2-diyl)bis(4,4,5,5-tetramethyl-1,3,2-dioxaborolane) (5e)

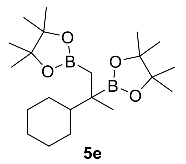

5e

Following the **procedure B** on 4 mmol scale, colorless oil liquid, yield: 68% (1.03 g), R_f = 0.45 (silica gel, PE: EtOAC = 20:1, v/v), column chromatography (silica gel, PE: EtOAC = 60:1, v/v).

^{1}H NMR (500 MHz, CDCl$_3$) δ 1.76 – 1.54 (m, 6H), 1.23 (s, 12H), 1.20 (s, 6H), 1.19 (s, 6H), 1.12 – 0.99 (m, 4H), 0.95 (d, *J* = 15.5 Hz, 1H), 0.91 (s, 3H), 0.67 (d, *J* = 15.3 Hz, 1H).

^{13}C NMR (126 MHz, CDCl$_3$) δ 82.77 (s), 82.62 (s), 47.05 (s), 29.06 (s), 28.24 (s), 26.91 (s), 25.11 (s), 24.80 (s), 20.79 (s).

^{11}B NMR (160 MHz, CDCl$_3$) δ 34.81 (s), 22.59 (s).

HRMS (EI-QTOF) *m/z*: $[M]^+$ Calcd. for $C_{21}H_{40}B_2O_4$ 378.3113; Found 378.3118.

2,2'-(2-methyl-4-phenylbutane-1,2-diyl)bis(4,4,5,5-tetramethyl-1,3,2-dioxaborolane) (5f)

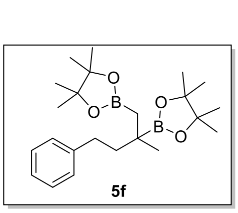

5f

Following the **procedure B** on 4 mmol scale, colorless oil liquid, yield: 70% (1.12 g), R_f = 0.35 (silica gel, PE: EtOAC = 20:1, v/v), column chromatography (silica gel, PE: EtOAC = 60:1, v/v).

^{1}H NMR (500 MHz, CDCl$_3$) δ 7.28 (t, *J* = 5.5 Hz, 2H), 7.19 (dd, *J* = 15.2, 7.1 Hz, 3H), 2.63 – 2.57 (m, 2H), 1.74 – 1.60 (m, 2H), 1.30 (s, 6H), 1.29 (s, 6H), 1.26 (s, 12H), 1.09 (s, 3H).

^{13}C NMR (126 MHz, CDCl$_3$) δ 143.75 (s), 128.31 (s), 128.11 (s), 125.29 (s), 82.91 (s), 82.73 (s), 44.17 (s), 32.32 (s), 24.96 (s), 24.68 (s), 23.91 (s).

^{11}B NMR (160 MHz, CDCl$_3$) δ 34.95 (s).

HRMS (EI-QTOF) *m/z*: [M]$^+$ Calcd. for $C_{23}H_{38}B_2O_4$ 400.2956; Found 400.2950.

2,2'-(2,3,3-trimethylbutane-1,2-diyl)bis(4,4,5,5-tetramethyl-1,3,2-dioxaborolane) (5g)

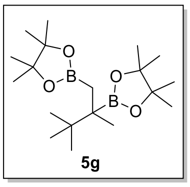

Following the **procedure B** on 4 mmol scale, white solid (m. p. = 33.8-35.0 ºC), yield: 52% (732.2 mg), R_f = 0.35 (silica gel, PE: EtOAC = 20:1, v/v), column chromatography (silica gel, PE: EtOAC = 60:1, v/v).

^{1}H NMR (500 MHz, CDCl$_3$) δ 1.59 (s, 2H), 1.27 (s, 12H), 1.22 (s, 6H), 1.21 (s, 6H), 0.93 (s, 3H), 0.88 (s, 9H).

^{13}C NMR (126 MHz, CDCl$_3$) δ 82.89 (s), 82.72 (s), 34.88 (s), 26.56 (s), 25.46 (s), 25.19 (s), 24.49 (s).

^{11}B NMR (128 MHz, CDCl$_3$) δ 34.77 (s).

HRMS (EI-QTOF) *m/z*: [M]$^+$ Calcd. for $C_{19}H_{38}B_2O_4$ 352.2956; Found 352.2958.

2,2'-(4-phenylbutane-2,2-diyl)bis(4,4,5,5-tetramethyl-1,3,2-dioxaborolane) (5'f)

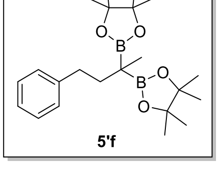

Following the **procedure C** on 5 mmol scale, white solid (m. p. = 103.2-105.0 ºC), yield: 32% (617.6 mg), R_f = 0.35 (silica gel, PE: EtOAC = 20:1, v/v), column chromatography

(silica gel, PE: EtOAC = 60:1, v/v).

^{1}H NMR (400 MHz, CDCl$_3$) δ 7.32 – 7.27 (m, 1H), 7.21 (d, *J* = 6.8 Hz, 1H), 2.68 – 2.61 (m, 1H), 1.93 – 1.85 (m, 1H), 1.29 (s, 7H), 1.23 (s, 1H).

^{13}C NMR (126 MHz, CDCl$_3$) δ 143.70 (s), 128.50 (s), 128.08 (s), 125.34 (s), 82.96 (s), 36.69 (s), 34.15 (s), 24.72 (s), 15.95 (s).

^{11}B NMR (128 MHz, CDCl$_3$) δ 34.22 (s).

HRMS (EI-QTOF) *m/z*: $[M]^+$ Calcd. for $C_{22}H_{36}B_2O_4$ 386.2800; Found 386.2805.

2,2'-(4-(4-methoxyphenyl)butane-2,2-diyl)bis(4,4,5,5-tetramethyl-1,3,2-dioxaborolane) (5'g)

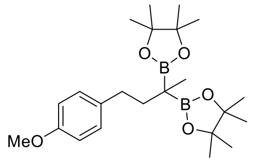

5'g

Following the **procedure C** on 5 mmol scale, white solid (m. p. = 104.9-106.1 ºC), yield: 36% (748.8 mg), R_f = 0.35 (silica gel, PE: EtOAC = 20:1, v/v), column chromatography (silica gel, PE: EtOAC = 60:1, v/v).

^{1}H NMR (400 MHz, CDCl$_3$) δ 7.12 (d, *J* = 7.6 Hz, 2H), 6.80 (d, *J* = 7.5 Hz, 2H), 3.77 (s, 3H), 2.57 – 2.46 (m, 2H), 1.85 – 1.73 (m, 2H), 1.22 (s, 24H), 1.15 (s, 3H).

^{13}C NMR (126 MHz, CDCl$_3$) δ 157.45 (s), 135.87 (s), 129.34 (s), 113.54 (s), 82.95 (s), 55.23 (s), 36.91 (s), 33.20 (s), 24.72 (s), 24.60 (s), 15.94 (s).

^{11}B NMR (128 MHz, CDCl$_3$) δ 33.95 (s).

HRMS (EI-QTOF) *m/z*: $[M]^+$ Calcd. for $C_{23}H_{38}B_2O_5$ 416.2905; Found 416.2909.

2.4 Characterization of Products

2-(2-(3,3-dimethylbutyl)phenyl)-4,4,5,5-tetramethyl-1,3,2-dioxaborolane (4a)

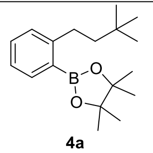

4a

Following the general **procedure D** on 0.2 mmol scale, colorless liquid, yield: 81%

(46.6 mg), R_f = 0.5 (silica gel, PE: Et_2O = 40:1, v/v), column chromatography (silica gel, PE: Et_2O = 100:1, v/v).

^{1}H NMR (400 MHz, $CDCl_3$) δ 7.79 (dd, *J* = 8.0, 1.3 Hz, 1H), 7.39 – 7.31 (m, 1H), 7.19 (s, 1H), 7.17 (s, 1H), 2.90 – 2.84 (m, 2H), 1.48 – 1.42 (m, 2H), 1.36 (s, 12H), 0.99 (s, 9H).

^{13}C NMR (101 MHz, $CDCl_3$) δ 150.82 (s), 136.10 (s), 130.91 (s), 129.24 (s), 124.72 (s), 83.34 (s), 48.08 (s), 31.28 (s), 30.81 (s), 29.45 (s), 24.89 (s).

^{11}B NMR (128 MHz, $CDCl_3$) δ 31.42 (s).

HRMS (EI-QTOF) *m/z*: $[M]^+$ Calcd. for $C_{18}H_{29}BO_2$ 288.2261; Found 288.2264.

2-(2-(3-ethyl-3-methylpentyl)phenyl)-4,4,5,5-tetramethyl-1,3,2-dioxaborolane (4b)

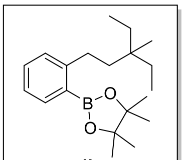

4b

Following the **procedure D** on 0.2 mmol scale, colorless liquid, yield: 84% (53.1 mg), R_f = 0.5 (silica gel, PE: Et_2O = 40:1, v/v), column chromatography (silica gel, PE: Et_2O = 100:1, v/v).

^{1}H NMR (500 MHz, $CDCl_3$) δ 7.80 (d, *J* = 7.3 Hz, 1H), 7.36 (td, *J* = 7.6, 1.3 Hz, 1H), 7.18 (t, *J* = 7.8 Hz, 2H), 2.83 – 2.78 (m, 2H), 1.46 – 1.41 (m, 2H), 1.36 (s, 12H), 1.34 – 1.30 (m, 4H), 0.90 (s, 3H), 0.85 (t, *J* = 7.5 Hz, 6H).

^{13}C NMR (126 MHz, $CDCl_3$) δ 151.02 (s), 136.10 (s), 130.94 (s), 129.28 (s), 124.70 (s), 42.48 (s), 35.43 (s), 30.96 (s), 30.10 (s), 24.85 (s), 23.93 (s), 8.03 (s).

^{11}B NMR (128 MHz, $CDCl_3$) δ 31.27 (s).

HRMS (EI-QTOF) *m/z*: $[M]^+$ Calcd. for $C_{20}H_{33}BO_2$ 316.2574; Found 316.2575.

2-(2-(2-((1r,3s)-adamantan-1-yl)ethyl)phenyl)-4,4,5,5-tetramethyl-1,3,2-dioxaborolan-e (4c)

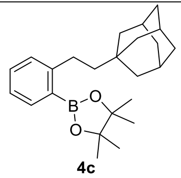

4c

Following the **procedure D** on 0.2 mmol scale, colorless liquid, yield: 78% (57.1 mg), $R_f = 0.5$ (silica gel, PE: Et_2O = 40:1, v/v), column chromatography (silica gel, PE: Et_2O = 100:1, v/v).

^{1}H NMR (400 MHz, CDCl$_3$) δ 7.77 (dd, *J* = 7.6, 1.3 Hz, 1H), 7.34 (td, *J* = 7.6, 1.5 Hz, 1H), 7.19 – 7.13 (m, 2H), 2.88 – 2.79 (m, 2H), 1.98 (s, 3H), 1.71 (dd, *J* = 27.1, 11.9 Hz, 7H), 1.59 (d, *J* = 2.2 Hz, 7H), 1.35 (s, 12H).

^{13}C NMR (126 MHz, CDCl$_3$) δ 151.09 (s), 136.03 (s), 130.87 (s), 129.26 (s), 124.66 (s), 83.34 (s), 48.65 (s), 42.56 (s), 37.36 (s), 32.80 (s), 29.20 (s), 28.86 (s), 24.92 (s).

^{11}B NMR (128 MHz, CDCl$_3$) δ 31.41 (s).

HRMS (EI-QTOF) *m/z*: $[M]^+$ Calcd. for $C_{24}H_{35}BO_2$ 366.2730; Found 366.2733.

4,4,5,5-tetramethyl-2-(2-(2-(3,3,9-trimethyl-1,5-dioxaspiro[5.5]undecan-9-yl)ethyl)phenyl)-1,3,2-dioxaborolane (4d)

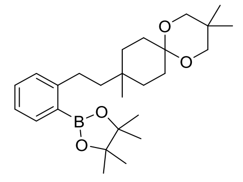

4d

Following the **procedure D** on 0.2 mmol scale, colorless liquid, yield: 77% (65.9 mg), $R_f = 0.5$ (silica gel, PE: Et_2O = 40:1, v/v), column chromatography (silica gel, PE: Et_2O = 100:1, v/v).

^{1}H NMR (500 MHz, CDCl$_3$) δ 7.78 (d, *J* = 7.3 Hz, 1H), 7.34 (td, *J* = 7.5, 1.5 Hz, 1H), 7.16 (t, *J* = 7.4 Hz, 2H), 3.52 (s, 2H), 3.49 (s, 2H), 2.86 – 2.78 (m, 2H), 1.91 – 1.84 (m, 2H), 1.80 – 1.72 (m, 2H), 1.54 – 1.44 (m, 4H), 1.40 – 1.35 (m, 2H), 1.34 (s, 12H), 1.03 (s, 3H), 0.97 (s, 6H).

^{13}C NMR (126 MHz, CDCl$_3$) δ 150.66 (s), 136.17 (s), 130.99 (s), 129.33 (s), 124.78 (s), 97.99 (s), 83.37 (s), 69.91 (s), 69.81 (s), 33.57 (s), 32.74 (s), 30.35 (s), 30.18 (s), 28.24 (s), 24.86 (s), 22.76 (s).

^{11}B NMR (128 MHz, CDCl$_3$) δ 31.99 (s).

HRMS (EI-QTOF) *m/z*: $[M]^+$ Calcd. for $C_{26}H_{41}BO_4$ 428.3098; Found 428.3101.

Ethyl3,3-dimethyl-5-(2-(4,4,5,5-tetramethyl-1,3,2-dioxaborolan-2-yl)phenyl)pentanoate (4e)

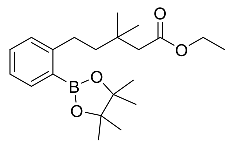

4e

Following the **procedure D** on 0.2 mmol scale, colorless liquid, yield: 80% (57.6 mg), R_f = 0.5 (silica gel, PE: Et_2O = 40:1, v/v), column chromatography (silica gel, PE: Et_2O = 100:1, v/v).

^{1}H NMR (500 MHz, CDCl$_3$) δ 7.80 – 7.78 (m, 1H), 7.37 – 7.33 (m, 1H), 7.17 (dd, J = 11.8, 4.3 Hz, 2H), 4.14 (q, J = 7.0 Hz, 2H), 2.91 – 2.85 (m, 2H), 2.31 (s, 2H), 1.58 – 1.52 (m, 2H), 1.35 (s, 12H), 1.27 (t, J = 7.1 Hz, 3H), 1.11 (s, 6H).

^{13}C NMR (126 MHz, CDCl$_3$) δ 172.48 (s), 150.13 (s), 136.20 (s), 131.00 (s), 129.33 (s), 124.87 (s), 83.37 (s), 59.84 (s), 46.49 (s), 46.21 (s), 33.68 (s), 30.64 (s), 27.05 (s), 24.88 (s), 14.33 (s).

^{11}B NMR (128 MHz, CDCl$_3$) δ 31.25 (s).

HRMS (EI-QTOF) *m/z*: $[M]^+$ Calcd. for $C_{21}H_{33}BO_4$ 360.2472; Found 360.2477.

Benzyl4-methyl-4-(2-(4,4,5,5-tetramethyl-1,3,2-dioxaborolan-2-yl)phenethyl)piperidine-1-carboxylate (4f)

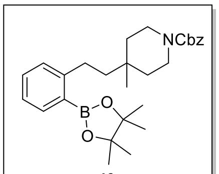

4f

Following the **procedure D** on 0.2 mmol scale, colorless liquid, yield: 70% (64.8 mg),

R_f = 0.45 (silica gel, PE: Et_2O = 20:1, v/v), column chromatography (silica gel, PE: Et_2O = 80:1, v/v).

^{1}H NMR (500 MHz, $CDCl_3$) δ 7.80 (dd, *J* = 7.4, 1.4 Hz, 1H), 7.39 – 7.31 (m, 6H), 7.20 – 7.14 (m, 2H), 5.14 (s, 2H), 3.63 (s, 2H), 3.44 – 3.35 (m, 2H), 2.84 (dd, *J* = 11.5, 5.2 Hz, 2H), 1.57 – 1.36 (m, 6H), 1.33 (s, 12H), 1.07 (s, 3H).

^{13}C NMR (126 MHz, $CDCl_3$) δ 155.42 (s), 150.15 (s), 137.02 (s), 136.33 (s), 131.07 (s), 129.29 (s), 128.41 (s), 127.84 (s), 127.75 (s), 124.95 (s), 83.41 (s), 66.84 (s), 44.90 (s), 40.19 (s), 31.71 (s), 29.83 (s), 24.88 (s), 23.91 (s).

^{11}B NMR (128 MHz, $CDCl_3$) δ 30.97 (s).

HRMS (ESI) m/z: $[M+H]^+$ Calcd. for $C_{28}H_{39}BNO_4^+$ 464.2967; Found:464.2972.

Tert-butyl((3,3-dimethyl-5-(2-(4,4,5,5-tetramethyl-1,3,2-dioxaborolan-2-yl)phenyl)pentyl)oxy)diphenylsilane (4g)

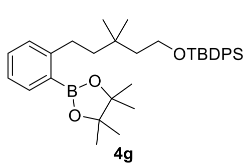

4g

Following the **procedure D** on 0.2 mmol scale, colorless liquid, yield: 78% (86.7 mg), R_f= 0.5 (silica gel, PE: Et_2O = 40:1, v/v), column chromatography (silica gel, PE: Et_2O = 100:1, v/v).

^{1}H NMR (500 MHz, $CDCl_3$) δ 7.81 (dd, *J* = 7.4, 1.3 Hz, 1H), 7.75 – 7.71 (m, 4H), 7.47 – 7.38 (m, 6H), 7.35 (td, *J* = 7.5, 1.5 Hz, 1H), 7.19 (td, *J* = 7.4, 1.1 Hz, 1H), 7.07 (d, *J* = 7.6 Hz, 1H), 3.83 – 3.79 (m, 2H), 2.85 – 2.79 (m, 2H), 1.71 – 1.65 (m, 2H), 1.43 – 1.38 (m, 2H), 1.35 (s, 12H), 1.09 (s, 9H), 0.95 (s, 6H).

^{13}C NMR (126 MHz, $CDCl_3$) δ 150.58 (s), 136.10 (s), 135.56 (s), 134.08 (s), 130.91 (s), 129.48 (s), 129.29 (s), 127.57 (s), 124.74 (s), 83.33 (s), 61.06 (s), 46.53 (s), 44.23 (s), 32.52 (s), 30.64 (s), 27.27 (s), 26.85 (s), 24.87 (s), 19.10 (s).

^{11}B NMR (128 MHz, $CDCl_3$) δ 30.84 (s).

HRMS (ESI) m/z: $[M+H]^+$ Calcd. for $C_{35}H_{50}BO_3Si^+$ 557.3617; Found:557.3624.

4,4,5,5-tetramethyl-2-(2-(2-(1-methyl-4-phenylcyclohexyl)ethyl)phenyl)-1,3,2-dioxaborolane (4h)

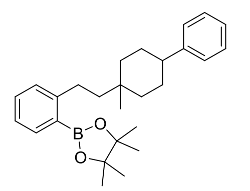

4h

Following the **procedure D** on 0.2 mmol scale, colorless liquid, yield: 82% (66.2 mg), R_f = 0.5 (silica gel, PE: Et_2O = 40:1, v/v), column chromatography (silica gel, PE: Et_2O = 100:1, v/v).

^{1}H NMR (500 MHz, CDCl$_3$) δ 7.80 (dd, *J* = 7.4, 1.4 Hz, 1H), 7.38 (td, *J* = 7.5, 1.6 Hz, 1H), 7.30 – 7.27 (m, 2H), 7.23 – 7.16 (m, 5H), 2.86 – 2.81 (m, 2H), 1.75 – 1.62 (m, 8H), 1.56 (s, 1H), 1.36 (t, *J* = 1.3 Hz, 2H), 1.32 (s, 12H), 1.06 (s).

^{13}C NMR (126 MHz, CDCl$_3$) δ 150.76 (s), 147.74 (s), 136.11 (s), 130.99 (s), 129.38 (s), 128.19 (s), 126.89 (s), 125.74 (s), 124.81 (s), 44.37 (s), 39.75 (s), 38.11 (s), 32.50 (s), 30.09 (s), 29.63 (s), 29.49 (s), 24.86 (s).

^{11}B NMR (128 MHz, CDCl$_3$) δ 31.78 (s).

HRMS (EI-QTOF) *m/z*: $[M]^+$ Calcd. for $C_{27}H_{37}BO_2$ 404.2887; Found 404.2890.

2-(2-(3,3-dimethyl-5-phenylpentyl)phenyl)-4,4,5,5-tetramethyl-1,3,2-dioxaborolane (4i)

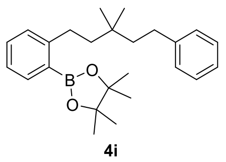

4i

Following the **procedure D** on 0.2 mmol scale, colorless liquid, yield: 76% (57.4 mg), R_f = 0.5 (silica gel, PE: Et2O = 40:1, v/v), column chromatography (silica gel, PE: Et2O = 100:1, v/v).

^{1}H NMR (500 MHz, CDCl$_3$) δ 7.80 – 7.78 (m, 1H), 7.38 – 7.33 (m, 1H), 7.30 – 7.26 (m, 2H), 7.22 – 7.16 (m, 5H), 2.91 – 2.85 (m, 2H), 2.65 – 2.59 (m, 2H), 1.61 – 1.56 (m, 2H), 1.55 – 1.50 (m, 2H), 1.31 (s, 12H), 1.04 (s, 6H).

^{13}C NMR (126 MHz, $CDCl_3$) δ 150.70 (s), 143.70 (s), 136.21 (s), 131.01 (s), 129.29 (s), 128.36 (s), 128.28 (s), 125.46 (s), 124.82 (s), 83.38 (s), 45.85 (s), 44.74 (s), 33.41 (s), 30.80 (s), 30.78 (s), 27.06 (s), 24.85 (s).

^{11}B NMR (128 MHz, $CDCl_3$) δ 31.97 (s).

HRMS (EI-QTOF) *m/z*: $[M]^+$ Calcd. for $C_{25}H_{35}BO_2$ 378.2730; Found 378.2734.

2-(2-(5-(4-methoxyphenyl)-3,3-dimethylpentyl)phenyl)-4,4,5,5-tetramethyl-1,3,2-dioxaborolane (4j)

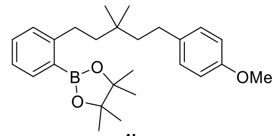

4j

Following the **procedure D** on 0.2 mmol scale, colorless liquid, yield: 72% (58.7 mg), R_f = 0.4 (silica gel, PE: Et_2O = 40:1, v/v), column chromatography (silica gel, PE: Et_2O = 80:1, v/v).

^{1}H NMR (500 MHz, $CDCl_3$) δ 7.80 (dd, *J* = 7.6, 1.4 Hz, 1H), 7.36 (td, *J* = 7.6, 1.4 Hz, 1H), 7.19 (d, *J* = 6.7 Hz, 2H), 7.13 (d, *J* = 8.5 Hz, 2H), 6.85 – 6.83 (m, 2H), 3.80 (s, 3H), 2.92 – 2.85 (m, 2H), 2.60 – 2.53 (m, 2H), 1.60 – 1.50 (m, 4H), 1.32 (s, 12H), 1.04 (s, 6H).

^{13}C NMR (126 MHz, $CDCl_3$) δ 157.52 (s), 150.72 (s), 136.20 (s), 135.75 (s), 131.00 (s), 129.29 (s), 129.18 (s), 124.81 (s), 113.71 (s), 83.37 (s), 55.26 (s), 45.83 (s), 44.96 (s), 33.37 (s), 30.78 (s), 29.81 (s), 27.08 (s), 24.85 (s).

^{11}B NMR (128 MHz, $CDCl_3$) δ 31.69 (s).

HRMS (EI-QTOF) *m/z*: $[M]^+$ Calcd. for $C_{26}H_{37}BO_3$ 408.2836; Found 408.2842.

2-(2-(5-(4-methoxyphenoxy)-3,3-dimethylpentyl)phenyl)-4,4,5,5-tetramethyl-1,3,2-dioxaborolane (4k)

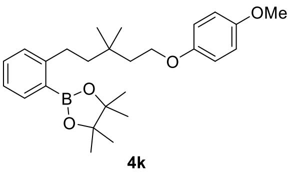

4k

Following the **procedure D** on 0.2 mmol scale, colorless liquid, yield: 72% (61.0 mg), R_f = 0.4 (silica gel, PE: Et_2O = 40:1, v/v), column chromatography (silica gel, PE: Et_2O = 80:1, v/v).

^{1}H NMR (500 MHz, $CDCl_3$) δ 7.82 – 7.78 (m, 1H), 7.36 (td, *J* = 7.6, 1.5 Hz, 1H), 7.21 – 7.16 (m, 2H), 6.84 (d, *J* = 1.1 Hz, 4H), 4.06 – 3.99 (m, 2H), 3.78 (s, 3H), 2.93 – 2.86 (m, 2H), 1.86 – 1.78 (m, 2H), 1.56 – 1.49 (m, 2H), 1.34 (s, 12H), 1.07 (s, 6H).

^{13}C NMR (126 MHz, $CDCl_3$) δ 153.59 (s), 153.21 (s), 150.40 (s), 136.21 (s), 131.00 (s), 129.27 (s), 124.86 (s), 115.35 (s), 114.58 (s), 83.40 (s), 65.61 (s), 55.71 (s), 46.42 (s), 40.69 (s), 32.65 (s), 30.66 (s), 27.31 (s), 24.86 (s).

^{11}B NMR (128 MHz, $CDCl_3$) δ 31.62 (s).

HRMS (EI-QTOF) *m/z*: $[M]^+$ Calcd. for $C_{26}H_{37}BO_4$ 424.2785; Found 424.2793.

2-(2-(5-(4-fluorophenoxy)-3,3-dimethylpentyl)phenyl)-4,4,5,5-tetramethyl-1,3,2-dioxaborolane (4l)

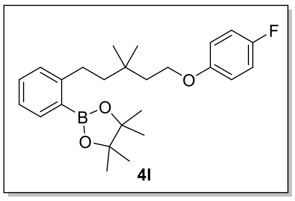

Following the **procedure D** on 0.2 mmol scale, colorless liquid, yield: 78% (64.2 mg), R_f = 0.5 (silica gel, PE: Et_2O = 40:1, v/v), column chromatography (silica gel, PE: Et_2O = 100:1, v/v).

^{1}H NMR (500 MHz, $CDCl_3$) δ 7.83 – 7.78 (m, 1H), 7.36 (td, *J* = 7.5, 1.5 Hz, 1H), 7.19 (dd, *J* = 11.2, 4.2 Hz, 2H), 7.02 – 6.92 (m, 2H), 6.90 – 6.79 (m, 2H), 4.03 (t, *J* = 7.5 Hz, 2H), 2.93 – 2.86 (m, 2H), 1.83 (t, *J* = 7.5 Hz, 2H), 1.57 – 1.49 (m, 2H), 1.34 (s, 12H), 1.07 (s, 6H).

^{13}C NMR (126 MHz, $CDCl_3$) δ 157.04 (d, *J* = 237.6 Hz), 155.14 (d, *J* = 2.2 Hz), 150.32 (s), 136.24 (s), 131.02 (s), 129.25 (s), 124.90 (s), 115.68 (d, *J* = 23.0 Hz), 115.33 (d, *J* = 7.9 Hz), 83.40 (s), 65.63 (s), 46.40 (s), 40.59 (s), 32.65 (s), 30.64 (s), 27.29 (s), 24.86 (s).

^{11}B NMR (128 MHz, $CDCl_3$) δ 31.12 (s).

^{19}F NMR (471 MHz, CDCl$_3$) δ -124.39 (s).

HRMS (EI-QTOF) *m/z*: $[M]^+$ Calcd. for $C_{25}H_{34}BFO_3$ 412.2585; Found 412.2584.

.

2-(2-(3,3-dimethyl-5-(4-(trifluoromethyl)phenoxy)pentyl)phenyl)-4,4,5,5-tetramethyl-1,3,2-dioxaborolane (4m)

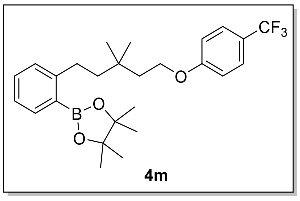

Following the **procedure D** on 0.2 mmol scale, colorless liquid, yield: 73% (67.6 mg), R_f= 0.5 (silica gel, PE: Et_2O = 40:1, v/v), column chromatography (silica gel, PE: Et_2O = 100:1, v/v).

^{1}H NMR (500 MHz, CDCl$_3$) δ 7.80 (dd, *J* = 7.3, 0.9 Hz, 1H), 7.54 (d, *J* = 8.5 Hz, 2H), 7.36 (td, *J* = 7.5, 1.5 Hz, 1H), 7.19 (dd, *J* = 11.8, 4.3 Hz, 2H), 6.95 (d, *J* = 8.5 Hz, 2H), 4.11 (t, *J* = 7.4 Hz, 2H), 2.94 – 2.83 (m, 2H), 1.86 (t, *J* = 7.4 Hz, 2H), 1.56 – 1.51 (m, 2H), 1.33 (s, 12H), 1.08 (s, 6H).

^{13}C NMR (126 MHz, CDCl$_3$) δ 161.48 (s), 150.24 (s), 136.28 (s), 131.05 (s), 129.24 (s), 126.81 (q, *J* = 3.9 Hz), 124.95 (s), 122.66 (s), 122.40 (s), 114.42 (s), 83.42 (s), 65.36 (s), 46.37 (s), 40.39 (s), 32.68 (s), 30.64 (s), 27.27 (s), 24.86 (s).

^{11}B NMR (128 MHz, CDCl$_3$) δ 31.55 (s).

^{19}F NMR (471 MHz, CDCl$_3$) δ -61.29 (s).

HRMS (EI-QTOF) *m/z*: $[M]^+$ Calcd. for $C_{26}H_{34}BF_3O_3$ 462.2553; Found 462.2556.

2-(2-(3,3-dimethyl-5-(4-(trifluoromethoxy)phenoxy)pentyl)phenyl)-4,4,5,5-tetramethyl-1,3,2-dioxaborolane (4n)

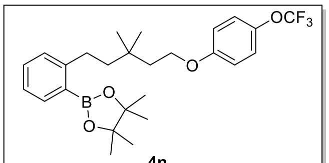

Following the **procedure D** on 0.2 mmol scale, colorless liquid, yield: 83% (79.3 mg), R_f = 0.5 (silica gel, PE: Et_2O = 40:1, v/v), column chromatography (silica gel, PE: Et_2O

= 100:1, v/v).

^{1}H NMR (500 MHz, CDCl$_3$) δ 7.82 – 7.78 (m, 1H), 7.36 (td, *J* = 7.5, 1.5 Hz, 1H), 7.19 (dd, *J* = 11.5, 4.3 Hz, 2H), 7.15 – 7.12 (m, 2H), 6.88 (d, *J* = 2.3 Hz, 1H), 6.87 (d, *J* = 2.3 Hz, 1H), 4.05 (t, *J* = 7.4 Hz, 2H), 2.93 – 2.86 (m, 2H), 1.84 (t, *J* = 7.4 Hz, 2H), 1.56 – 1.50 (m, 2H), 1.33 (s, 12H), 1.07 (s, 6H).

^{13}C NMR (126 MHz, CDCl$_3$) δ 157.68 (s), 150.39 (s), 142.58 (q, *J* = 3.8 Hz), 136.37 (s), 131.14 (s), 129.36 (s), 125.03 (s), 122.48 (s), 119.66 (s), 115.25 (s), 83.52 (s), 65.63 (s), 46.50 (s), 40.60 (s), 32.78 (s), 30.75 (s), 27.39 (s), 24.96 (s).

^{11}B NMR (128 MHz, CDCl$_3$) δ 31.60 (s).

^{19}F NMR (471 MHz, CDCl$_3$) δ -58.29 (s).

HRMS (EI-QTOF) *m/z*: [M]$^+$ Calcd. for $C_{26}H_{34}BF_3O_4$ 478.2502; Found 478.2507.

2-(2-(3,3-dimethylbutyl)-5-methylphenyl)-4,4,5,5-tetramethyl-1,3,2-dioxaborolane (4o)

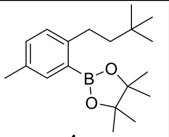

4o

Following the **procedure D** on 0.2 mmol scale, colorless liquid, yield: 72% (43.5 mg), R_f = 0.5 (silica gel, PE: Et_2O = 40:1, v/v), column chromatography (silica gel, PE: Et_2O = 100:1, v/v).

^{1}H NMR (400 MHz, CDCl$_3$) δ 7.59 (s, 1H), 7.16 (dd, *J* = 7.7, 1.6 Hz, 1H), 7.07 (d, *J* = 7.8 Hz, 1H), 2.85 – 2.78 (m, 2H), 2.31 (s, 3H), 1.45 – 1.39 (m, 2H), 1.34 (s, 12H), 0.97 (s, 9H).

^{13}C NMR (126 MHz, CDCl$_3$) δ 147.82 (s), 136.56 (s), 133.94 (s), 131.74 (s), 129.29 (s), 83.30 (s), 48.25 (s), 30.78 (s), 30.21 (s), 29.45 (s), 24.88 (s), 20.81 (s).

^{11}B NMR (128 MHz, CDCl$_3$) δ 30.98 (s).

HRMS (EI-QTOF) *m/z*: [M]$^+$ Calcd. for $C_{19}H_{31}BO_2$ 302.2417; Found 302.2420.

2-(2-(3,3-dimethylbutyl)-4-methoxyphenyl)-4,4,5,5-tetramethyl-1,3,2-

dioxaborolane (4p)

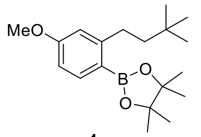

4p

Following the **procedure D** on 0.2 mmol scale, colorless liquid, yield: 78% (49.6 mg), R_f = 0.45 (silica gel, PE: Et_2O = 40:1, v/v), column chromatography (silica gel, PE: Et_2O = 90:1, v/v).

^{1}H NMR (500 MHz, CDCl$_3$) δ 7.76 – 7.73 (m, 1H), 6.71 (dt, *J* = 6.1, 3.0 Hz, 2H), 3.82 (s, 3H), 2.86 – 2.82 (m, 2H), 1.47 – 1.42 (m, 2H), 1.33 (s, 12H), 0.98 (s, 9H).

^{13}C NMR (126 MHz, CDCl$_3$) δ 161.78 (s), 153.27 (s), 138.09 (s), 114.90 (s), 110.11 (s), 83.06 (s), 54.99 (s), 47.89 (s), 31.43 (s), 30.80 (s), 29.43 (s), 24.86 (s).

^{11}B NMR (160 MHz, CDCl$_3$) δ 31.49 (s).

HRMS (EI-QTOF) *m/z*: $[M]^+$ Calcd. for $C_{19}H_{31}BO_3$ 318.2366; Found 318.2370.

2-(2-(3,3-dimethylbutyl)-4,5-dimethoxyphenyl)-4,4,5,5-tetramethyl-1,3,2-dioxaborolane (4q)

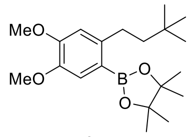

4q

Following the **procedure D** on 0.2 mmol scale, colorless liquid, yield: 69% (48.0 mg), R_f = 0.3 (silica gel, PE: Et_2O = 40:1, v/v), column chromatography (silica gel, PE: Et_2O = 60:1, v/v).

^{1}H NMR (400 MHz, CDCl$_3$) δ 7.29 (s, 1H), 6.70 (s, 1H), 3.92 (s, 6H), 2.88 – 2.79 (m, 2H), 1.48 – 1.42 (m, 2H), 1.36 (s, 12H), 1.00 (s, 9H).

^{13}C NMR (101 MHz, CDCl$_3$) δ 151.11 (s), 146.12 (s), 145.18 (s), 118.27 (s), 112.46 (s), 83.18 (s), 55.95 (s), 55.67 (s), 48.19 (s), 30.90 (s), 30.79 (s), 29.46 (s), 24.88 (s).

^{11}B NMR (128 MHz, CDCl$_3$) δ 31.05 (s).

HRMS (EI-QTOF) *m/z*: $[M]^+$ Calcd. for $C_{20}H_{33}BO_4$ 348.2472; Found 348.2477.

2-(6-(3,3-dimethylbutyl)benzo[d][1,3]dioxol-5-yl)-4,4,5,5-tetramethyl-1,3,2-dioxaborolane (4r)

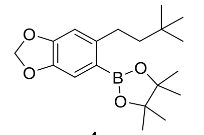

4r

Following the **procedure D** on 0.2 mmol scale, colorless liquid, yield: 76% (50.4 mg), R_f = 0.5 (silica gel, PE: Et_2O = 40:1, v/v), column chromatography (silica gel, PE: Et_2O = 100:1, v/v).

^{1}H NMR (500 MHz, $CDCl_3$) δ 7.22 (s, 1H), 6.66 (s, 1H), 5.90 (s, 2H), 2.84 – 2.75 (m, 2H), 1.42 – 1.36 (m, 2H), 1.32 (s, 12H), 0.96 (s, 9H).

^{13}C NMR (126 MHz, $CDCl_3$) δ 149.83 (s), 146.67 (s), 144.95 (s), 114.85 (s), 109.87 (s), 100.68 (s), 83.25 (s), 48.20 (s), 30.99 (s), 30.76 (s), 29.43 (s), 24.86 (s).

^{11}B NMR (128 MHz, $CDCl_3$) δ 31.02 (s).

HRMS (EI-QTOF) *m/z*: $[M]^+$ Calcd. for $C_{19}H_{29}BO_4$ 332.2159; Found 332.2162.

2-(2-(3,3-dimethylbutyl)-3-fluorophenyl)-4,4,5,5-tetramethyl-1,3,2-dioxaborolane (4s)

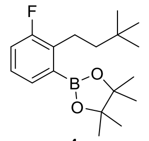

4s

Following the **procedure D** on 0.2 mmol scale, colorless liquid, yield: 65% (39.8 mg), R_f = 0.5 (silica gel, PE: Et_2O = 40:1, v/v), column chromatography (silica gel, PE: Et_2O = 100:1, v/v).

^{1}H NMR (500 MHz, $CDCl_3$) δ 7.54 (dd, *J* = 7.4, 1.1 Hz, 1H), 7.13 (td, *J* = 7.7, 5.2 Hz, 1H), 7.09 – 7.04 (m, 1H), 2.93 – 2.88 (m, 2H), 1.44 – 1.39 (m, 2H), 1.35 (s, 12H), 0.99 (s, 9H).

^{13}C NMR (126 MHz, $CDCl_3$) δ 161.12 (d, *J* = 244.3 Hz), 136.92 (d, *J* = 14.3 Hz), 131.53 (d, *J* = 3.3 Hz), 126.32 (d, *J* = 8.0 Hz), 117.65 (d, *J* = 23.2 Hz), 83.62 (s), 45.79 (s), 30.86 (s), 29.30 (s), 24.87 (s), 23.27 (s).

^{11}B NMR (128 MHz, $CDCl_3$) δ 31.05 (s).

^{19}F NMR (471 MHz, CDCl$_3$) δ -119.96 (s).

HRMS (EI-QTOF) *m/z*: $[M]^+$ Calcd. for $C_{18}H_{28}BFO_2$ 306.2166; Found 306.2168.

2-(2-(3,3-dimethylbutyl)-4-fluorophenyl)-4,4,5,5-tetramethyl-1,3,2-dioxaborolane(4t)

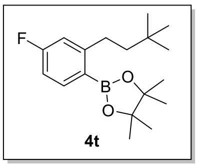

Following the **procedure D** on 0.2 mmol scale, colorless liquid, yield: 71% (43.5 mg), R_f = 0.5 (silica gel, PE: Et_2O = 40:1, v/v), column chromatography (silica gel, PE: Et_2O = 100:1, v/v).

^{1}H NMR (500 MHz, CDCl$_3$) δ 7.78 – 7.73 (m, 1H), 6.88 – 6.84 (m, 2H), 2.87 – 2.82 (m, 2H), 1.44 – 1.40 (m, 2H), 1.33 (s, 12H), 0.97 (s, 9H).

^{13}C NMR (126 MHz, CDCl$_3$) δ 164.77 (d, J = 249.7 Hz), 154.14 (d, J = 7.3 Hz), 138.34 (d, J = 8.4 Hz), 115.90 (d, J = 19.3 Hz), 111.78 (d, J = 19.8 Hz), 83.43 (s), 47.63 (s), 31.21 (s), 30.80 (s), 29.40 (s), 24.87 (s).

^{11}B NMR (128 MHz, CDCl$_3$) δ 30.99 (s).

^{19}F NMR (471 MHz, CDCl$_3$) δ -109.97 (s).

HRMS (EI-QTOF) *m/z*: $[M]^+$ Calcd. for $C_{18}H_{28}BFO_2$ 306.2166; Found 306.2168.

2-(2-(3,3-dimethylbutyl)-5-fluorophenyl)-4,4,5,5-tetramethyl-1,3,2-dioxaborolan (4u)

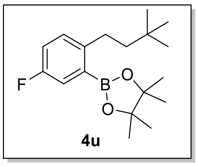

Following the **procedure D** on 0.2 mmol scale, colorless liquid, yield: 69% (42.2 mg), R_f = 0.5 (silica gel, PE: Et_2O = 40:1, v/v), column chromatography (silica gel, PE: Et_2O = 100:1, v/v).

^{1}H NMR (500 MHz, $CDCl_3$) δ 7.44 (dd, *J* = 9.4, 2.9 Hz, 1H), 7.12 (dd, *J* = 8.5, 5.3 Hz, 1H), 7.02 (td, *J* = 8.5, 3.0 Hz, 1H), 2.85 – 2.79 (m, 2H), 1.43 – 1.38 (m, 2H), 1.35 (s, 12H), 0.97 (s, 9H).

^{13}C NMR (126 MHz, $CDCl_3$) δ 160.62 (d, *J* = 243.4 Hz), 146.43 (d, *J* = 2.7 Hz), 130.91 (d, *J* = 6.5 Hz), 121.96 (d, *J* = 19.2 Hz), 117.74 (d, *J* = 20.7 Hz), 83.79 (s), 48.26 (s), 30.88 (s), 30.53 (s), 29.52 (s), 24.97 (s).

^{11}B NMR (128 MHz, $CDCl_3$) δ 30.70 (s).

^{19}F NMR (471 MHz, $CDCl_3$) δ -119.47 (s).

HRMS (EI-QTOF) *m/z*: $[M]^+$ Calcd. for $C_{18}H_{28}BFO_2$ 306.2166; Found 306.2165.

2-(2-(3,3-dimethylbutyl)naphthalen-1-yl)-4,4,5,5-tetramethyl-1,3,2-dioxaborolane(4v)

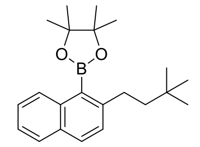

4v

Following the **procedure D** on 0.2 mmol scale, colorless liquid, yield: 80% (54.1 mg), R_f = 0.5 (silica gel, PE: Et_2O = 40:1, v/v), column chromatography (silica gel, PE: Et_2O = 100:1, v/v).

^{1}H NMR (500 MHz, $CDCl_3$) δ 8.12 (d, *J* = 8.3 Hz, 1H), 7.77 (t, *J* = 7.6 Hz, 2H), 7.44 (ddd, *J* = 8.4, 6.9, 1.3 Hz, 1H), 7.40 – 7.36 (m, 1H), 7.30 (d, *J* = 8.4 Hz, 1H), 2.89 – 2.82 (m, 2H), 1.60 – 1.53 (m, 2H), 1.49 (s, 12H), 1.01 (s, 9H).

^{13}C NMR (126 MHz, $CDCl_3$) δ 146.96 (s), 136.59 (s), 131.44 (s), 129.72 (s), 128.09 (s), 127.65 (s), 127.54 (s), 125.91 (s), 124.56 (s), 83.97 (s), 47.40 (s), 32.22 (s), 30.83 (s), 29.47 (s), 25.21 (s).

^{11}B NMR (128 MHz, $CDCl_3$) δ 32.78 (s).

HRMS (EI-QTOF) *m/z*: $[M]^+$ Calcd. for $C_{22}H_{31}BO_2$ 338.2417; Found 338.2422.

2-(3-(3,3-dimethylbutyl)phenyl)-4,4,5,5-tetramethyl-1,3,2-dioxaborolane (4w)

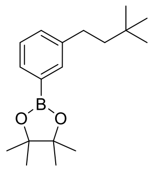

4w

Following the **procedure D** on 0.2 mmol scale, colorless liquid, yield: 55% (31.6 mg), R_f = 0.5 (silica gel, PE: Et_2O = 40:1, v/v), column chromatography (silica gel, PE: Et_2O = 100:1, v/v).

^{1}H NMR (500 MHz, CDCl$_3$) δ 7.65 – 7.61 (m, 2H), 7.29 (dd, *J* = 4.0, 1.6 Hz, 2H), 2.63 – 2.52 (m, 2H), 1.53 – 1.47 (m, 2H), 1.35 (s, 12H), 0.96 (s, 9H).

^{13}C NMR (126 MHz, CDCl$_3$) δ 142.83 (s), 134.57 (s), 132.03 (s), 131.37 (s), 127.74 (s), 83.69 (s), 46.54 (s), 31.17 (s), 30.55 (s), 29.33 (s), 24.85 (s).

^{11}B NMR (128 MHz, CDCl$_3$) δ 31.07 (s).

HRMS (EI-QTOF) *m/z*: $[M]^+$ Calcd. for $C_{18}H_{29}BO_2$ 288.2261; Found 288.2264.

2-(4-(3,3-dimethylbutyl)phenyl)-4,4,5,5-tetramethyl-1,3,2-dioxaborolane (4x)

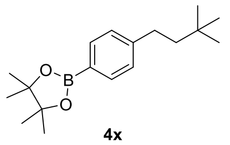

4x

Following the **procedure D** on 0.2 mmol scale, colorless liquid, yield: 80% (46.1 mg), R_f = 0.5 (silica gel, PE: Et_2O = 40:1, v/v), column chromatography (silica gel, PE: Et_2O = 100:1, v/v).

^{1}H NMR (500 MHz, CDCl$_3$) δ 7.73 (d, *J* = 7.9 Hz, 2H), 7.20 (d, *J* = 7.8 Hz, 2H), 2.61 – 2.51 (m, 2H), 1.52 – 1.46 (m, 2H), 1.34 (s, 12H), 0.96 (s, 9H).

^{13}C NMR (126 MHz, CDCl$_3$) δ 147.12 (s), 134.87 (s), 127.80 (s), 83.59 (s), 46.25 (s), 31.52 (s), 30.57 (s), 29.32 (s), 24.84 (s).

^{11}B NMR (128 MHz, CDCl$_3$) δ 31.42 (s).

HRMS (EI-QTOF) *m/z*: $[M]^+$ Calcd. for $C_{18}H_{29}BO_2$ 288.2261; Found 288.2259.

2-(4-(4,4-dimethylpentan-2-yl)phenyl)-4,4,5,5-tetramethyl-1,3,2-dioxaborolane (4y)

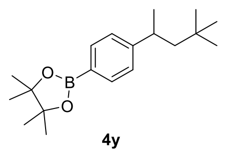

4y

Following the **procedure D** on 0.2 mmol scale, white solid (m. p. = 80.0-81.6), yield: 74% (44.7 mg), R_f = 0.5 (silica gel, PE: Et_2O = 40:1, v/v), column chromatography (silica gel, PE: Et_2O = 100:1, v/v).

^{1}H NMR (500 MHz, $CDCl_3$) δ 7.73 (d, J = 8.0 Hz, 2H), 7.23 (d, J = 8.0 Hz, 2H), 2.86 – 2.81 (m, 1H), 1.74 (dd, J = 14.0, 8.0 Hz, 1H), 1.49 (dd, J = 14.0, 4.4 Hz, 1H), 1.34 (s, 12H), 1.22 (d, J = 7.0 Hz, 2H), 0.80 (s, 9H).

^{13}C NMR (126 MHz, $CDCl_3$) δ 153.14 (s), 134.89 (s), 126.59 (s), 83.56 (s), 51.81 (s), 37.09 (s), 31.32 (s), 30.13 (s), 25.80 (s), 24.84 (s).

^{11}B NMR (128 MHz, $CDCl_3$) δ 31.05 (s).

HRMS (EI-QTOF) *m/z*: $[M]^+$ Calcd. for $C_{19}H_{31}BO_2$ 302.2417; Found 304.2421.

2-(4-(5,5-dimethylhexan-3-yl)phenyl)-4,4,5,5-tetramethyl-1,3,2-dioxaborolane (4z)

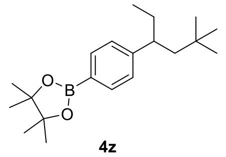

4z

Following the **procedure D** on 0.2 mmol scale, colorless liquid, yield: 73% (46.1 mg), R_f= 0.5 (silica gel, PE: Et_2O = 40:1, v/v), column chromatography (silica gel, PE: Et_2O = 100:1, v/v).

^{1}H NMR (500 MHz, $CDCl_3$) δ 7.70 (d, J = 8.0 Hz, 2H), 7.16 (d, J = 8.0 Hz, 2H), 2.50 (ddd, J = 9.0, 5.5, 2.0 Hz, 1H), 1.66-1.74 (m, 1H), 1.61 – 1.57 (m, 1H), 1.54 – 1.46 (m, 2H), 1.33 (s, 12H), 0.75 (s, 9H), 0.69 (t, J = 7.4 Hz, 3H).

^{13}C NMR (126 MHz, $CDCl_3$) δ 151.34 (s), 134.67 (s), 127.47 (s), 83.56 (s), 50.23 (s), 44.68 (s), 32.54 (s), 31.28 (s), 30.13 (s), 24.89 (s), 24.86 (s), 12.23 (s).

^{11}B NMR (128 MHz, $CDCl_3$) δ 31.19 (s).

HRMS (EI-QTOF) *m/z*: $[M]^+$ Calcd. for $C_{20}H_{33}BO_2$ 316.2574; Found 316.2579.

2-(4-(2,2-dimethyloctan-4-yl)phenyl)-4,4,5,5-tetramethyl-1,3,2-dioxaborolane (4aa)

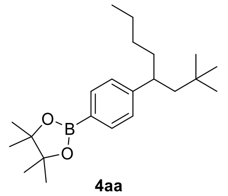

4aa

Following the **procedure D** on 0.2 mmol scale, colorless liquid, yield: 74% (50.9 mg), R_f = 0.5 (silica gel, PE: Et_2O = 40:1, v/v), column chromatography (silica gel, PE: Et_2O = 100:1, v/v).

^{1}H NMR (500 MHz, $CDCl_3$) δ 7.71 (d, *J* = 8.0 Hz, 2H), 7.18 (d, *J* = 8.0 Hz, 2H), 2.61 (tdd, *J* = 8.9, 5.5, 3.3 Hz, 1H), 1.71 (dd, *J* = 14.0, 8.8 Hz, 1H), 1.55 (ddd, *J* = 10.0, 7.2, 4.7 Hz, 1H), 1.49 (ddd, *J* = 13.4, 8.2, 4.1 Hz, 2H), 1.34 (s, 12H), 1.28 – 1.20 (m, 2H), 1.15 – 1.05 (m, 1H), 1.03 – 0.93 (m, 1H), 0.80 (t, *J* = 7.3 Hz, 3H), 0.76 (s, 9H).

^{13}C NMR (126 MHz, $CDCl_3$) δ 151.68 (s), 134.68 (s), 127.37 (s), 83.55 (s), 79.67 – 71.46 (m), 50.59 (s), 42.80 (s), 39.57 (s), 31.32 (s), 30.14 (s), 29.82 (s), 24.87 (s), 22.68 (s), 13.98 (s).

^{11}B NMR (128 MHz, $CDCl_3$) δ 30.81 (s).

HRMS (EI-QTOF) *m/z*: $[M]^+$ Calcd. for $C_{22}H_{37}BO_2$ 344.2887; Found 344.2885.

2-(4-(4,4-dimethyl-1-phenylpentan-2-yl)phenyl)-4,4,5,5-tetramethyl-1,3,2-dioxaborolane (4ab)

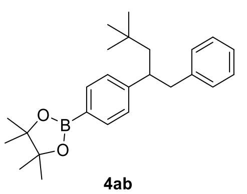

4ab

Following the **procedure D** on 0.2 mmol scale, white solid (m. p. = 83.1-83.4), yield: 67% (54.1 mg), R_f= 0.5 (silica gel, PE: Et_2O = 40:1, v/v), column chromatography (silica gel, PE: Et_2O = 100:1, v/v).

^{1}H NMR (500 MHz, CDCl$_3$) δ 7.67 (d, *J* = 7.9 Hz, 2H), 7.20 – 7.11 (m, 5H), 6.98 (d, *J* = 7.1 Hz, 2H), 2.97 – 2.91 (m, 1H), 2.80 (d, *J* = 7.5 Hz, 2H), 1.61 (dd, *J* = 13.8, 2.8 Hz, 2H), 1.34 (s, 12H), 0.72 (s, 9H).

^{13}C NMR (126 MHz, CDCl$_3$) δ 150.54 (s), 140.61 (s), 134.67 (s), 129.14 (s), 127.95 (s), 127.47 (s), 125.69 (s), 83.59 (s), 48.82 (s), 46.23 (s), 45.03 (s), 31.25 (s), 30.07 (s), 24.89 (s).

^{11}B NMR (128 MHz, CDCl$_3$) δ 31.61 (s).

HRMS (EI-QTOF) *m/z*: $[M]^+$ Calcd. for $C_{25}H_{35}BO_2$ 378.2730; Found 378.2734.

4,4,5,5-tetramethyl-2-(1-neopentyl-2,3-dihydro-1H-inden-5-yl)-1,3,2-dioxaborolane (4ac)

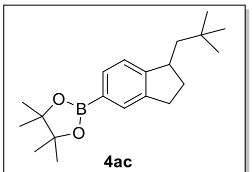

4ac

Following the **procedure D** on 0.2 mmol scale, colorless liquid, yield: 67% (42.1 mg), R_f = 0.5 (silica gel, PE: Et_2O = 40:1, v/v), column chromatography (silica gel, PE: Et_2O = 100:1, v/v).

^{1}H NMR (500 MHz, CDCl$_3$) δ 7.67 (s, 1H), 7.63 (d, *J* = 7.5 Hz, 1H), 7.19 (d, *J* = 7.5 Hz, 1H), 3.12 (q, *J* = 8.2 Hz, 1H), 2.92 – 2.88 (m, 1H), 2.87 – 2.76 (m, 1H), 2.45 – 2.34 (m, 1H), 1.87 (dd, *J* = 14.0, 2.1 Hz, 1H), 1.72 – 1.60 (m, 2H), 1.34 (s, 12H), 1.01 (s, 9H).

^{13}C NMR (126 MHz, CDCl$_3$) δ 152.57 (s), 143.17 (s), 132.85 (s), 130.54 (s), 122.92 (s), 83.56 (s), 49.92 (s), 41.89 (s), 35.35 (s), 31.61 (s), 31.06 (s), 30.16 (s), 24.82 (s).

^{11}B NMR (128 MHz, CDCl$_3$) δ 31.20 (s).

HRMS (EI-QTOF) *m/z*: $[M]^+$ Calcd. for $C_{20}H_{31}BO_2$ 314.2417; Found 314.2419.

2-(2-(2-cyclobutylethyl)-4-methoxyphenyl)-4,4,5,5-tetramethyl-1,3,2-dioxaborolane (4ad)

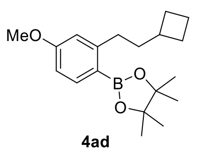

4ad

Following the **procedure D** on 0.2 mmol scale, colorless liquid, yield: 60% (37.9 mg), R_f = 0.45 (silica gel, PE: Et_2O = 40:1, v/v), column chromatography (silica gel, PE: Et_2O = 90:1, v/v).

^{1}H NMR (500 MHz, CDCl$_3$) δ 7.73 (d, *J* = 8.1 Hz, 1H), 6.73 – 6.68 (m, 2H), 3.81 (s, 3H), 2.79 – 2.73 (m, 2H), 2.31 (dq, *J* = 15.4, 7.7 Hz, 1H), 2.05 (tdd, *J* = 7.8, 6.2, 3.2 Hz, 2H), 1.88 – 1.78 (m, 2H), 1.71 – 1.61 (m, 4H), 1.33 (s, 12H).

^{13}C NMR (126 MHz, CDCl$_3$) δ 161.77 (s), 152.60 (s), 138.14 (s), 115.05 (s), 110.21 (s), 83.17 (s), 55.08 (s), 40.60 (s), 36.32 (s), 33.77 (s), 28.19 (s), 24.95 (s), 18.57 (s).

^{11}B NMR (128 MHz, CDCl$_3$) δ 31.47 (s).

HRMS (EI-QTOF) *m/z*: $[M]^+$ Calcd. for $C_{19}H_{29}BO_3$ 316.2210; Found 316.2212.

2-(2-(2-cyclopentylethyl)-4-methoxyphenyl)-4,4,5,5-tetramethyl-1,3,2-dioxaborolane (4ae)

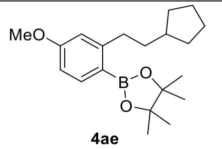

4ae

Following the **procedure D** on 0.2 mmol scale, colorless liquid, yield: 64% (42.4 mg), R_f= 0.45 (silica gel, PE: Et_2O = 40:1, v/v), column chromatography (silica gel, PE: Et_2O = 90:1, v/v).

^{1}H NMR (500 MHz, CDCl$_3$) δ 7.73 (d, *J* = 8.0 Hz, 1H), 6.76 – 6.68 (m, 2H), 3.81 (s, 3H), 2.91 – 2.83 (m, 2H), 1.90 – 1.82 (m, 1H), 1.78 (dd, *J* = 11.0, 5.3 Hz, 2H), 1.62 – 1.49 (m, 8H), 1.33 (s, 12H).

^{13}C NMR (126 MHz, CDCl$_3$) δ 161.69 (s), 152.79 (s), 138.02 (s), 114.87 (s), 110.10 (s), 83.05 (s), 54.98 (s), 40.32 (s), 40.01 (s), 35.36 (s), 32.51 (s), 25.30 (s), 24.84 (s).

^{11}B NMR (160 MHz, CDCl$_3$) δ 31.39 (s).

HRMS (EI-QTOF) *m/z*: $[M]^+$ Calcd. for $C_{20}H_{31}BO_3$ 330.2366; Found 330.2368.

2-(2-(2-cyclohexylethyl)-4-methoxyphenyl)-4,4,5,5-tetramethyl-1,3,2-

dioxaborolane (4af)

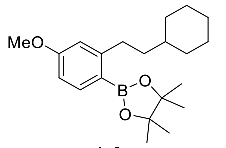
4af

Following the **procedure D** on 0.2 mmol scale, colorless liquid, yield: 68% (46.8 mg), R_f = 0.45 (silica gel, PE: Et_2O = 40:1, v/v), column chromatography (silica gel, PE: Et_2O = 90:1, v/v).

^{1}H NMR (500 MHz, $CDCl_3$) δ 7.75 – 7.70 (m, 1H), 6.70 (dd, *J* = 6.9, 2.2 Hz, 2H), 3.81 (s, 3H), 2.89 – 2.83 (m, 2H), 1.79 (d, *J* = 13.1 Hz, 2H), 1.74 – 1.68 (m, 2H), 1.66 (dd, *J* = 8.2, 4.8 Hz, 1H), 1.47 – 1.40 (m, 2H), 1.32 (s, 12H), 1.29 – 1.13 (m, 4H), 0.95 (qd, *J* = 12.5, 3.0 Hz, 2H).

^{13}C NMR (126 MHz, $CDCl_3$) δ 161.71 (s), 153.04 (s), 138.02 (s), 114.86 (s), 110.09 (s), 83.06 (s), 54.99 (s), 41.32 (s), 38.18 (s), 33.63 (s), 33.38 (s), 26.78 (s), 26.44 (s), 24.86 (s).

^{11}B NMR (128 MHz, $CDCl_3$) δ 31.49 (s).

HRMS (EI-QTOF) *m/z*: $[M]^+$ Calcd. for $C_{21}H_{33}BO_3$ 344.2523 Found 344.2529.

2-(2-(2-cycloheptylethyl)-4-methoxyphenyl)-4,4,5,5-tetramethyl-1,3,2-dioxaborolane (4ag)

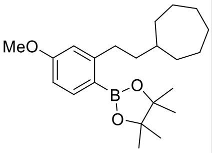
4ag

Following the **procedure D** on 0.2 mmol scale, yellow oil liquid, yield: 62% (53.1 mg), R_f = 0.45 (silica gel, PE: Et_2O = 40:1, v/v), column chromatography (silica gel, PE: Et_2O = 90:1, v/v).

^{1}H NMR (400 MHz, $CDCl_3$) δ 7.76 – 7.70 (m, 1H), 6.70 (dd, *J* = 6.4, 2.5 Hz, 2H), 3.81 (s, 3H), 2.91 – 2.81 (m, 2H), 1.78 (ddd, *J* = 13.5, 7.0, 3.3 Hz, 2H), 1.70 – 1.37 (m, 13H), 1.33 (s, 12H).

^{13}C NMR (126 MHz, CDCl$_3$) δ 161.71 (s), 152.98 (s), 138.03 (s), 114.89 (s), 110.11 (s), 83.05 (s), 54.97 (s), 41.99 (s), 39.70 (s), 34.47 (s), 34.18 (s), 28.68 (s), 26.60 (s), 24.85 (s).

^{11}B NMR (160 MHz, CDCl$_3$) δ 31.44 (s).

HRMS (EI-QTOF) *m/z*: [M]$^+$ Calcd. for $C_{22}H_{35}BO_3$ 358.2679; Found 358.2674.

4,4,5,5-tetramethyl-2-(2-(2-((2S,4R)-1,2,3,3,4-pentamethylcyclohexyl)ethyl)phenyl)-1,3,2-dioxaborolane (4ah)

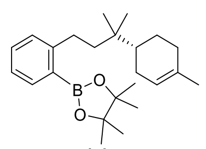

4ah

Following the **procedure D** on 0.2 mmol scale, colorless liquid, yield: 65% (47.8 mg), R_f = 0.45 (silica gel, PE: Et_2O = 40:1, v/v), column chromatography (silica gel, PE: Et_2O = 90:1, v/v).

^{1}H NMR (400 MHz, CDCl$_3$) δ 7.78 (d, *J* = 6.5 Hz, 1H), 7.35 (td, *J* = 7.5, 1.4 Hz, 1H), 7.20 – 7.13 (m, 2H), 5.41 (s, 1H), 2.90 – 2.75 (m, 2H), 2.09 – 1.76 (m, 6H), 1.65 (s, 3H), 1.54 – 1.42 (m, 3H), 1.34 (s, 12H), 0.95 (s, 3H), 0.93 (s, 3H).

^{13}C NMR (101 MHz, CDCl$_3$) δ 150.98 (s), 136.15 (s), 133.86 (s), 130.96 (s), 129.30 (s), 124.74 (s), 121.47 (s), 83.37 (s), 44.19 (s), 42.27 (s), 35.08 (s), 31.64 (s), 30.46 (s), 26.43 (s), 24.89 (s), 24.31 (s), 23.89 (s), 23.36 (s).

^{11}B NMR (128 MHz, CDCl$_3$) δ 31.66 (s).

HRMS (EI-QTOF) *m/z*: [M]$^+$ Calcd. for $C_{24}H_{37}BO_2$ 368.2887; Found 368.2889.

2-(2-(6-(2,5-dimethylphenoxy)-3,3-dimethylhexyl)phenyl)-4,4,5,5-tetramethyl-1,3,2-dioxaborolane (4ai)

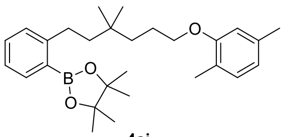

4ai

Following the **procedure D** on 0.2 mmol scale, colorless liquid, yield: 79% (63.6 mg), R_f = 0.5 (silica gel, PE: Et_2O = 40:1, v/v), column chromatography (silica gel, PE: Et_2O

= 100:1, v/v).

^{1}H NMR (400 MHz, CDCl$_3$) δ 7.83 – 7.78 (m, 1H), 7.39 – 7.33 (m, 1H), 7.18 (t, *J* = 7.1 Hz, 2H), 7.02 (d, *J* = 7.4 Hz, 1H), 6.70 – 6.63 (m, 2H), 3.96 (t, *J* = 6.5 Hz, 2H), 2.93 – 2.83 (m, 2H), 2.33 (s, 3H), 2.20 (s, 3H), 1.88 – 1.78 (m, 2H), 1.54 – 1.43 (m, 4H), 1.35 (s, 13H), 1.02 (s, 6H).

^{13}C NMR (126 MHz, CDCl$_3$) δ 157.10 (s), 150.73 (s), 136.39 (s), 136.16 (s), 130.98 (s), 130.23 (s), 129.30 (s), 124.78 (s), 123.57 (s), 120.50 (s), 111.92 (s), 83.37 (s), 68.69 (s), 45.78 (s), 38.32 (s), 33.02 (s), 30.76 (s), 27.13 (s), 24.88 (s), 24.30 (s), 21.41 (s), 15.80 (s).

^{11}B NMR (128 MHz, CDCl$_3$) δ 31.61 (s).

HRMS (EI-QTOF) *m/z*: $[M]^+$ Calcd. for $C_{28}H_{41}BO_3$ 436.3149; Found 436.3158.

2-(2-(5-(benzo[d][1,3]dioxol-5-yl)-3,3-dimethylpentyl)phenyl)-4,4,5,5-tetramethyl-1,3,2-dioxaborolane (4aj)

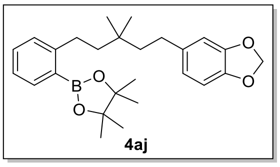

Following the **procedure D** on 0.2 mmol scale, colorless liquid, yield: 81% (68.3 mg), R_f = 0.5 (silica gel, PE: Et_2O = 40:1, v/v), column chromatography (silica gel, PE: Et_2O = 100:1, v/v).

^{1}H NMR (500 MHz, CDCl$_3$) δ 7.82 (dd, *J* = 7.7, 1.5 Hz, 1H), 7.38 (td, *J* = 7.6, 1.6 Hz, 1H), 7.21 – 7.18 (m, 2H), 6.73 (dd, *J* = 12.8, 4.7 Hz, 2H), 6.67 (dd, *J* = 7.9, 1.7 Hz, 1H), 5.92 (s, 2H), 2.92 – 2.85 (m, 2H), 2.58 – 2.52 (m, 2H), 1.59 – 1.50 (m, 4H), 1.34 (s, 12H), 1.04 (s, 6H).

^{13}C NMR (126 MHz, CDCl$_3$) δ 150.79 (s), 147.59 (s), 145.44 (s), 137.69 (s), 136.35 (s), 131.14 (s), 129.41 (s), 124.95 (s), 121.01 (s), 109.01 (s), 108.21 (s), 100.78 (s), 83.50 (s), 45.94 (s), 45.11 (s), 33.49 (s), 30.89 (s), 30.66 (s), 27.19 (s), 24.98 (s).

^{11}B NMR (128 MHz, CDCl$_3$) δ 31.51 (s).

HRMS (EI-QTOF) *m/z*: $[M]^+$ Calcd. for $C_{26}H_{35}BO_4$ 422.2628; Found 422.2634.

2-(4-(4-(4-isobutylphenyl)-3,3-dimethylpentyl)phenyl)-4,4,5,5-tetramethyl-1,3,2-dioxaborolane (4ak)

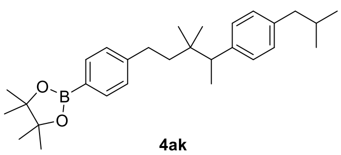

4ak

Following the **procedure D** on 0.2 mmol scale, colorless liquid, yield: 68% (59.0 mg), R_f = 0.5 (silica gel, PE: Et_2O = 40:1, v/v), column chromatography (silica gel, PE: Et_2O = 100:1, v/v).

^{1}H NMR (500 MHz, $CDCl_3$) δ 7.72 (d, *J* = 7.8 Hz, 2H), 7.17 (d, *J* = 7.9 Hz, 2H), 7.07 (d, *J* = 8.1 Hz, 2H), 7.02 (d, *J* = 8.1 Hz, 2H), 2.72 – 2.66 (m, 1H), 2.64 – 2.52 (m, 2H), 2.44 (d, *J* = 7.2 Hz, 2H), 1.89 – 1.78 (m, 1H), 1.59 – 1.52 (m, 1H), 1.50 – 1.42 (m, 1H), 1.34 (s, 12H), 1.26 (d, *J* = 7.2 Hz, 3H), 0.92 (s, 3H), 0.91 (s, 3H), 0.89 (s, 3H), 0.88 (s, 3H).

^{13}C NMR (126 MHz, $CDCl_3$) δ 147.06 (s), 141.86 (s), 139.10 (s), 134.87 (s), 128.88 (s), 128.19 (s), 127.84 (s), 83.60 (s), 47.59 (s), 45.01 (s), 42.75 (s), 36.25 (s), 30.88 (s), 30.19 (s), 24.83 (s), 24.40 (s), 22.44 (s), 15.62 (s).

^{11}B NMR (128 MHz, $CDCl_3$) δ 32.78 (s).

HRMS (EI-QTOF) *m/z*: $[M]^+$ Calcd. for $C_{29}H_{43}BO_2$ 434.3356; Found 434.3354.

2-(2,2-diethyl-4-(2-(4,4,5,5-tetramethyl-1,3,2-dioxaborolan-2-yl)phenyl)butyl)-4,4,5,5-tetramethyl-1,3,2-dioxaborolane (6a)

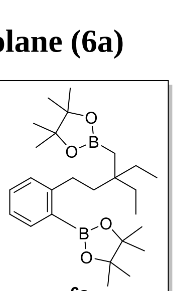

6a

Following the **procedure D** on 0.2 mmol scale, colorless liquid, yield: 72% (63.6 mg), R_f= 0.5 (silica gel, PE: Et_2O = 40:1, v/v), column chromatography (silica gel, PE: Et_2O = 100:1, v/v).

^{1}H NMR (500 MHz, $CDCl_3$) δ 7.77 (d, *J* = 7.4 Hz, 1H), 7.34 (dd, *J* = 10.7, 4.3 Hz, 1H), 7.27 (s, 1H), 7.15 (t, *J* = 7.3 Hz, 1H), 2.84 – 2.78 (m, 2H), 1.55 – 1.49 (m, 2H), 1.41 (q, *J* = 7.4 Hz, 4H), 1.34 (s, 12H), 1.24 (s, 12H), 0.85 (dd, *J* = 8.3, 6.5 Hz, 8H).

^{13}C NMR (126 MHz, $CDCl_3$) δ 151.35 (s), 136.05 (s), 130.90 (s), 129.60 (s), 124.68 (s), 83.45 (s), 82.65 (s), 42.23 (s), 37.97 (s), 30.89 (s), 30.03 (s), 24.97 (s), 24.95 (s), 8.31 (s).

^{11}B NMR (160 MHz, $CDCl_3$) δ 33.81 (s), 31.71 (s).

HRMS (EI-QTOF) *m/z*: $[M]^+$ Calcd. for $C_{26}H_{44}B_2O_4$ 442.3426; Found 442.3428.

2-(2-(3,4-dimethyl-3-((4,4,5,5-tetramethyl-1,3,2-dioxaborolan-2-yl)methyl)pentyl)phenyl)-4,4,5,5-tetramethyl-1,3,2-dioxaborolane (6b)

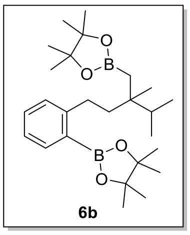

Following the **procedure D** on 0.2 mmol scale, colorless liquid, yield: 66% (63.6 mg), R_f = 0.5 (silica gel, PE: Et_2O = 40:1, v/v), column chromatography (silica gel, PE: Et_2O = 100:1, v/v).

^{1}H NMR (500 MHz, $CDCl_3$) δ 7.76 (dd, *J* = 7.4, 1.2 Hz, 1H), 7.34 (td, *J* = 7.5, 1.5 Hz, 1H), 7.25 (s, 1H), 7.17 – 7.13 (m, 1H), 2.88 – 2.81 (m, 2H), 1.69 (dt, *J* = 13.6, 6.8 Hz, 1H), 1.55 (dd, *J* = 11.5, 5.9 Hz, 2H), 1.34 (s, 12H), 1.24 (s, 12H), 1.01 (s, 3H), 0.90 – 0.86 (m, 8H).

^{13}C NMR (126 MHz, $CDCl_3$) δ 151.27 (s), 135.94 (s), 130.78 (s), 129.53 (s), 124.57 (s), 83.33 (s), 82.59 (s), 43.23 (s), 37.82 (s), 35.97 (s), 30.32 (s), 24.89 (s), 24.84 (s), 23.81 (s), 17.59 (s).

^{11}B NMR (128 MHz, $CDCl_3$) δ 34.17 (s), 31.63 (s).

HRMS (EI-QTOF) *m/z*: $[M]^+$ Calcd. for $C_{26}H_{44}B_2O_4$ 442.3426; Found 442.3422.

4,4,5,5-tetramethyl-2-(2-methyl-2-(2-(4,4,5,5-tetramethyl-1,3,2-dioxaborolan-2-yl)phenethyl)pentyl)-1,3,2-dioxaborolane (6c)

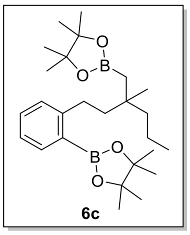

Following the **procedure D** on 0.2 mmol scale, colorless liquid, yield: 73% (64.5 mg), R_f = 0.5 (silica gel, PE: Et_2O = 40:1, v/v), column chromatography (silica gel, PE: Et_2O = 100:1, v/v).

^{1}H NMR (500 MHz, $CDCl_3$) δ 7.76 (dd, *J* = 7.4, 1.1 Hz, 1H), 7.33 (td, *J* = 7.5, 1.5 Hz, 1H), 7.22 (d, *J* = 7.6 Hz, 1H), 7.15 (dd, *J* = 11.0, 4.1 Hz, 1H), 2.88 – 2.77 (m, 2H), 1.54 – 1.48 (m, 2H), 1.34 (s, 12H), 1.30 (d, *J* = 9.1 Hz, 2H), 1.25 (s, 12H), 1.04 (s, 3H), 0.93 – 0.83 (m, 6H).

^{13}C NMR (126 MHz, $CDCl_3$) δ 151.09 (s), 135.96 (s), 130.81 (s), 129.42 (s), 124.59 (s), 83.32 (s), 82.62 (s), 46.14 (s), 44.49 (s), 35.33 (s), 31.42 (s), 30.54 (s), 27.16 (s), 24.87 (s), 17.15 (s), 15.10 (s).

^{11}B NMR (160 MHz, $CDCl_3$) δ 34.18 (s), 31.83 (s).

HRMS (EI-QTOF) *m/z*: $[M]^+$ Calcd. for $C_{26}H_{44}B_2O_4$ 442.3426; Found 442.3430.

4,4,5,5-tetramethyl-2-(2-methyl-2-(2-(4,4,5,5-tetramethyl-1,3,2-dioxaborolan-2-yl)phenethyl)hexyl)-1,3,2-dioxaborolane (6d)

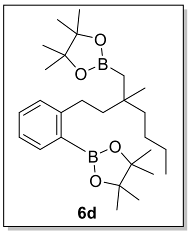

Following the **procedure D** on 0.2 mmol scale, colorless liquid, yield: 70% (63.8 mg), R_f = 0.5 (silica gel, PE: Et_2O = 40:1, v/v), column chromatography (silica gel, PE: Et_2O = 100:1, v/v).

^{1}H NMR (500 MHz, $CDCl_3$) δ 7.76 (dd, *J* = 7.3, 1.0 Hz, 1H), 7.34 (td, *J* = 7.6, 1.4 Hz, 1H), 7.22 (d, *J* = 7.6 Hz, 1H), 7.17 – 7.13 (m, 1H), 2.87 – 2.80 (m, 2H), 1.54 – 1.48 (m, 2H), 1.34 (s, 12H), 1.31 – 1.27 (m, 4H), 1.25 (s, 12H), 1.04 (s, 3H), 0.93 – 0.87 (m, 6H).

^{13}C NMR (126 MHz, CDCl$_3$) δ 151.09 (s), 135.95 (s), 130.81 (s), 129.38 (s), 124.59 (s), 83.31 (s), 82.61 (s), 46.17 (s), 41.68 (s), 35.21 (s), 30.56 (s), 27.09 (s), 26.21 (s), 24.87 (s), 23.65 (s), 14.18 (s).

^{11}B NMR (160 MHz, CDCl$_3$) δ 33.47 (s), 31.61 (s).

HRMS (EI-QTOF) *m/z*: $[M]^+$ Calcd. for $C_{27}H_{46}B_2O_4$ 456.3582; Found 456.3584.

2-(2-cyclohexyl-2-methyl-4-(2-(4,4,5,5-tetramethyl-1,3,2-dioxaborolan-2-yl)phenyl)butyl)-4,4,5,5-tetramethyl-1,3,2-dioxaborolane (6e)

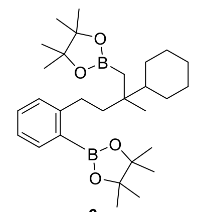

6e

Following the **procedure D** on 0.2 mmol scale, colorless liquid, yield: 71% (68.4 mg), R_f = 0.5 (silica gel, PE: Et_2O = 40:1, v/v), column chromatography (silica gel, PE: Et_2O = 100:1, v/v).

^{1}H NMR (500 MHz, CDCl$_3$) δ 7.79 (d, *J* = 7.2 Hz, 1H), 7.35 (dd, *J* = 7.4, 1.0 Hz, 1H), 7.28 (d, *J* = 4.2 Hz, 1H), 7.17 (t, *J* = 7.4 Hz, 1H), 2.84 (dt, *J* = 19.6, 9.7 Hz, 2H), 1.88 – 1.74 (m, 5H), 1.66 (d, *J* = 13.3 Hz, 2H), 1.57 (dd, *J* = 10.9, 6.4 Hz,), 1.36 (s, 12H), 1.31 – 1.27 (m, 2H), 1.26 (s, 12H), 1.05 (s, 3H), 0.99 – 0.88 (m, 4H).

^{13}C NMR (126 MHz, CDCl$_3$) δ 151.30 (s), 135.95 (s), 130.79 (s), 129.52 (s), 124.55 (s), 83.32 (s), 82.58 (s), 47.13 (s), 43.53 (s), 37.89 (s), 30.38 (s), 27.44 (s), 27.27 (s), 26.87 (s), 24.88 (s), 24.86 (s), 24.53 (s).

^{11}B NMR (160 MHz, CDCl$_3$) δ 33.82 (s), 31.82 (s).

HRMS (EI-QTOF) *m/z*: $[M]^+$ Calcd. for $C_{29}H_{48}B_2O_4$ 482.3739; Found 482.3744.

4,4,5,5-tetramethyl-2-(2-methyl-2-phenethyl-4-(2-(4,4,5,5-tetramethyl-1,3,2-dioxaborolan-2-yl)phenyl)butyl)-1,3,2-dioxaborolane (6f)

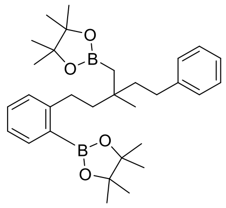

6f

Following the **procedure D** on 0.2 mmol scale, colorless liquid, yield: 67% (63.6 mg), R_f = 0.5 (silica gel, PE: Et_2O = 40:1, v/v), column chromatography (silica gel, PE: Et_2O = 100:1, v/v).

^{1}H NMR (500 MHz, $CDCl_3$) δ 7.78 (d, *J* = 7.4 Hz, 1H), 7.34 (t, *J* = 7.4 Hz, 1H), 7.23 (t, *J* = 7.5 Hz, 5H), 7.18 – 7.12 (m, 2H), 2.89 (dd, *J* = 15.8, 6.1 Hz, 2H), 2.66 – 2.59 (m, 2H), 1.63 (ddd, *J* = 24.8, 13.9, 7.2 Hz, 6H), 1.28 (s, 12H), 1.25 (s, 12H), 1.12 (s, 3H).

^{13}C NMR (126 MHz, $CDCl_3$) δ 150.85 (s), 143.96 (s), 136.10 (s), 130.91 (s), 129.41 (s), 128.46 (s), 128.17 (s), 125.32 (s), 124.70 (s), 83.33 (s), 82.75 (s), 46.23 (s), 44.52 (s), 35.48 (s), 30.81 (s), 30.64 (s), 27.18 (s), 24.91 (s), 24.81 (s).

^{11}B NMR (160 MHz, $CDCl_3$) δ 33.47 (s), 31.87 (s).

HRMS (EI-QTOF) *m/z*: $[M]^+$ Calcd. for $C_{31}H_{46}B_2O_4$ 504.3582; Found 504.3586.

4,4,5,5-tetramethyl-2-(2,3,3-trimethyl-2-(2-(4,4,5,5-tetramethyl-1,3,2-dioxaborolan-2-yl)phenethyl)butyl)-1,3,2-dioxaborolane (6g)

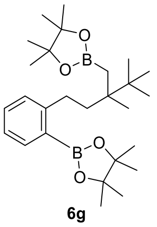

6g

Following the **procedure D** on 0.2 mmol scale, colorless liquid, yield: 44% (40.1 mg), R_f= 0.5 (silica gel, PE: Et_2O = 40:1, v/v), column chromatography (silica gel, PE: Et_2O = 100:1, v/v).

^{1}H NMR (500 MHz, $CDCl_3$) δ 7.77 (dd, *J* = 7.4, 1.3 Hz, 1H), 7.36 – 7.29 (m, 2H), 7.16 (td, *J* = 7.3, 1.5 Hz, 2H), 2.98 (td, *J* = 12.1, 5.0 Hz, 1H), 2.77 (td, *J* = 12.1, 4.8 Hz, 1H), 1.59 (ddd, *J* = 17.6, 12.6, 4.7 Hz, 4H), 1.33 (s, 12H), 1.25 (s, 6H), 1.25 (s, 6H), 1.05 (s, 3H), 0.87 (s, 9H).

^{13}C NMR (126 MHz, CDCl$_3$) δ 151.59 (s), 135.94 (s), 130.79 (s), 129.72 (s), 124.60 (s), 83.33 (s), 82.52 (s), 41.56 (s), 40.73 (s), 37.16 (s), 29.69 (s), 26.22 (s), 21.94 (s).

^{11}B NMR (160 MHz, CDCl$_3$) δ 34.03 (s), 31.33 (s).

HRMS (EI-QTOF) *m/z*: $[M]^+$ Calcd. for $C_{27}H_{46}B_2O_4$ 456.3582; Found 456.3588.

4,4,5,5-tetramethyl-2-(2-(5-phenyl-3-(4,4,5,5-tetramethyl-1,3,2-dioxaborolan-2-yl)pentyl)phenyl)-1,3,2-dioxaborolane (6'a)

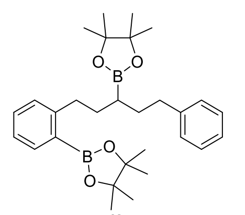

6'a

Following the **procedure D** on 0.2 mmol scale, colorless liquid, yield: 73% (69.5 mg), R_f = 0.5 (silica gel, PE: Et_2O = 40:1, v/v), column chromatography (silica gel, PE: Et_2O = 100:1, v/v).

^{1}H NMR (500 MHz, CDCl$_3$) δ 7.37 – 7.31 (m, 1H), 7.29 – 7.27 (m, 1H), 7.23 – 7.15 (m, 2H), 7.23 – 7.14 (m, 5H),2.99 (m, 1H), 2.88 – 2.80 (m, 1H), 2.72 – 2.58 (m, 2H), 1.84 – 1.73 (m, 4H), 1.71 – 1.65 (m, 1H), 1.31 (s, 12H), 1.30 (s, 12H).

^{13}C NMR (126 MHz, CDCl$_3$) δ 150.11 (s), 143.15 (s), 136.01 (s), 130.79 (s), 129.23 (s), 128.38 (s), 128.16 (s), 125.46 (s), 124.83 (s), 83.29 (s), 82.90 (s), 35.70 (s), 35.58 (s), 34.95 (s), 33.33 (s), 24.83 (s), 24.77 (s).

^{11}B NMR (128 MHz, CDCl$_3$) δ 34.71 (s), 31.56 (s).

HRMS (EI-QTOF) *m/z*: $[M]^+$ Calcd. for $C_{29}H_{42}B_2O_4$ 476.3269; Found 476.3276.

4,4,5,5-tetramethyl-2-(2-(3-(4,4,5,5-tetramethyl-1,3,2-dioxaborolan-2-yl)-4-(m-tolyl)butyl)phenyl)-1,3,2-dioxaborolane (6'b)

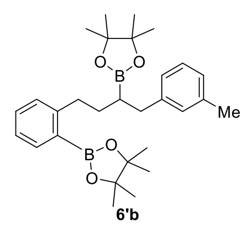

6'b

Following the **procedure D** on 0.2 mmol scale, colorless liquid, yield: 69% (65.7 mg), R_f = 0.5 (silica gel, PE: Et_2O = 40:1, v/v), column chromatography (silica gel, PE: Et_2O = 100:1, v/v).

^{1}H NMR (500 MHz, CDCl$_3$) δ 7.76 (dd, *J* = 7.4, 0.8 Hz, 1H), 7.34 (td, *J* = 7.6, 1.3 Hz, 1H), 7.20 – 7.11 (m, 3H), 7.05 – 6.99 (m, 2H), 6.94 (d, *J* = 7.5 Hz, 1H), 3.08 – 3.01 (m, 1H), 2.86 – 2.78 (m, 1H), 2.76 – 2.69 (m, 2H), 2.30 (s, 3H), 1.73 – 1.63 (m, 2H), 1.49 – 1.43 (m, 1H), 1.31 (s, 6H), 1.30 (s, 6H), 1.19 (s, 6H), 1.16 (s, 6H).

^{13}C NMR (126 MHz, CDCl$_3$) δ 149.95 (s), 142.39 (s), 137.32 (s), 135.99 (s), 130.80 (s), 129.67 (s), 129.24 (s), 127.90 (s), 126.19 (s), 125.91 (s), 124.87 (s), 83.31 (s), 82.91 (s), 37.22 (s), 35.60 (s), 35.08 (s), 24.82 (s), 24.72 (s), 21.36 (s).

^{11}B NMR (128 MHz, CDCl$_3$) δ 34.50 (s), 32.16 (s).

HRMS (EI-QTOF) *m/z*: $[M]^+$ Calcd. for $C_{29}H_{42}B_2O_4$ 476.3269; Found 476.3273.

2-(2-(4-(3-chlorophenyl)-3-(4,4,5,5-tetramethyl-1,3,2-dioxaborolan-2-yl)butyl)phenyl)-4,4,5,5-tetramethyl-1,3,2-dioxaborolane (6'c)

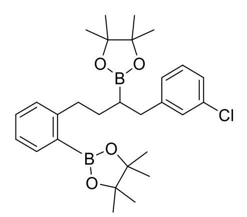

6'c

Following the **procedure D** on 0.2 mmol scale, colorless liquid, yield: 70% (69.4 mg), R_f = 0.5 (silica gel, PE: Et_2O = 40:1, v/v), column chromatography (silica gel, PE: Et_2O = 100:1, v/v).

^{1}H NMR (400 MHz, CDCl$_3$) δ 7.77 (d, *J* = 7.4 Hz, 1H), 7.34 (t, *J* = 7.5 Hz, 1H), 7.23 (s, 1H), 7.19 – 7.07 (m, 5H), 3.07 – 2.99 (m, 1H), 2.88 – 2.79 (m, 1H), 2.72 (dd, *J* =

15.8, 8.4 Hz, 2H), 1.73 – 1.58 (m, 2H), 1.49 – 1.39 (m, 1H), 1.31 (s, 12H), 1.20 (s, 6H), 1.17 (s, 6H).

^{13}C NMR (126 MHz, CDCl$_3$) δ 149.73 (s), 144.21 (d, *J* = 110.6 Hz), 136.07 (s), 133.71 (s), 130.85 (s), 129.25 (s), 129.23 (s), 129.03 (s), 127.09 (s), 125.65 (s), 124.95 (s), 83.34 (s), 83.08 (s), 36.93 (s), 35.49 (s), 34.93 (s), 24.79 (s), 24.75 (s).

^{11}B NMR (128 MHz, CDCl$_3$) δ 34.26 (s), 31.84 (s).

HRMS (EI-QTOF) *m/z*: $[M]^+$ Calcd. for $C_{28}H_{39}B_2ClO_4$ 496.2723; Found 496.2724.

2-(2-(4-(3-fluorophenyl)-3-(4,4,5,5-tetramethyl-1,3,2-dioxaborolan-2-yl)butyl)phenyl)-4,4,5,5-tetramethyl-1,3,2-dioxaborolane (6'd)

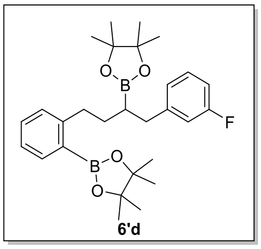

Following the **procedure D** on 0.2 mmol scale, colorless liquid, yield: 67% (64.3 mg), R_f = 0.5 (silica gel, PE: Et_2O = 40:1, v/v), column chromatography (silica gel, PE: Et_2O = 100:1, v/v).

^{1}H NMR (500 MHz, CDCl$_3$) δ 7.77 (d, *J* = 7.0 Hz, 1H), 7.34 (t, *J* = 7.4 Hz, 1H), 7.21 – 7.14 (m, 3H), 6.95 (dd, *J* = 20.6, 8.8 Hz, 2H), 6.83 (t, *J* = 8.4 Hz, 1H), 3.04 (td, *J* = 11.8, 5.4 Hz, 1H), 2.83 (td, *J* = 11.9, 5.4 Hz, 1H), 2.77 – 2.71 (m, 2H), 1.76 – 1.62 (m, 2H), 1.45 (dt, *J* = 15.0, 7.7 Hz, 1H), 1.31 (s, 6H), 1.31 (s, 6H), 1.19 (s, 6H), 1.17 (s, 6H).

^{13}C NMR (126 MHz, CDCl$_3$) δ 162.72 (d, *J* = 244.7 Hz), 149.76 (s), 145.18 (d, *J* = 7.4 Hz), 136.07 (s), 130.85 (s), 129.34 (d, *J* = 8.4 Hz), 129.23 (s), 124.95 (s), 124.53 (d, *J* = 2.9 Hz), 115.66 (d, *J* = 20.7 Hz), 112.31 (d, *J* = 20.9 Hz), 83.33 (s), 83.06 (s), 36.99 (s), 35.51 (s), 34.92 (s), 24.83 (s), 24.79 (s).

^{11}B NMR (128 MHz, CDCl$_3$) δ 34.28 (s), 32.31 (s).

^{19}F NMR (471 MHz, CDCl$_3$) δ -114.36 (s).

HRMS (EI-QTOF) *m/z*: $[M]^+$ Calcd. for $C_{28}H_{39}B_2FO_4$ 480.3018; Found 480.3020.

2-(2-(4-(4-(tert-butyl)phenyl)-3-(4,4,5,5-tetramethyl-1,3,2-dioxaborolan-2-yl)butyl)phenyl)-4,4,5,5-tetramethyl-1,3,2-dioxaborolane (6'e)

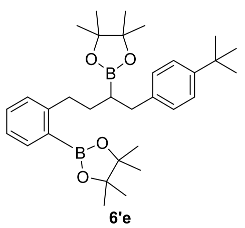

6'e

Following the **procedure D** on 0.2 mmol scale, colorless liquid, yield: 77% (79.8 mg), R_f = 0.5 (silica gel, PE: Et_2O = 40:1, v/v), column chromatography (silica gel, PE: Et_2O = 100:1, v/v).

^{1}H NMR (400 MHz, CDCl$_3$) δ 7.78 (d, J = 7.3 Hz, 1H), 7.35 (t, J = 7.4 Hz, 1H), 7.28 (s, 1H), 7.18 (t, J = 10.4 Hz, 4H), 3.06 (m, 1H), 2.88 – 2.78 (m, 1H), 2.79 – 2.68 (m, 2H), 1.70 (m, 2H), 1.50 (dt, J = 16.6, 8.3 Hz, 1H), 1.33 (s, 9H), 1.31 (s, 12H), 1.20 (s, 6H), 1.16 (s, 6H).

^{13}C NMR (126 MHz, CDCl$_3$) δ 149.98 (s), 148.18 (s), 139.30 (s), 135.97 (s), 130.79 (s), 129.22 (s), 128.51 (s), 124.84 (s), 83.29 (s), 82.88 (s), 36.76 (s), 35.65 (s), 35.08 (s), 34.24 (s), 31.39 (s), 24.81 (s), 24.63 (s).

^{11}B NMR (128 MHz, CDCl$_3$) δ 34.25 (s), 31.24 (s).

HRMS (EI-QTOF) *m/z*: [M]$^+$ Calcd. For $C_{32}H_{48}B_2O_4$ 518.3739; Found 518.3734.

4,4,5,5-tetramethyl-2-(3-methyl-1-phenyl-5-(2-(4,4,5,5-tetramethyl-1,3,2-

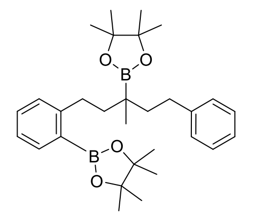

6'f

dioxaborolan-2-yl)phenyl)pentan-3-yl)-1,3,2-dioxaborolane (6'f)

Following the **procedure D** on 0.2 mmol scale, colorless liquid, yield: 68% (64.7 mg), R_f = 0.5 (silica gel, PE: Et_2O = 40:1, v/v), column chromatography (silica gel, PE: Et_2O = 100:1, v/v).

^{1}H NMR (500 MHz, CDCl$_3$) δ 7.83 (d, *J* = 6.3 Hz, 1H), 7.41 (td, *J* = 7.5, 1.3 Hz, 1H), 7.33 (t, *J* = 7.5 Hz, 3H), 7.29 – 7.25 (m, 2H), 7.24 – 7.19 (m, 2H), 3.07 (td, *J* = 12.6, 4.6 Hz, 1H), 2.83 (td, *J* = 12.6, 4.4 Hz, 1H), 2.75 – 2.59 (m, 2H), 1.85 (td, *J* = 12.8, 4.9 Hz, 1H), 1.77 (td, *J* = 12.9, 4.5 Hz, 1H), 1.69 – 1.62 (m, 2H), 1.37 (s, 12H), 1.36 (s, 12H), 1.19 (s, 3H).

^{13}C NMR (126 MHz, CDCl$_3$) δ 150.71 (s), 143.79 (s), 135.99 (s), 130.94 (s), 129.36 (s), 128.36 (s), 128.19 (s), 125.40 (s), 124.74 (s), 83.35 (s), 83.04 (s), 42.51 (s), 41.69 (s), 32.67 (s), 31.91 (s), 24.94 (s), 24.83 (s), 21.13 (s).

^{11}B NMR (128 MHz, CDCl$_3$) δ 35.23 (s), 31.71 (s).

HRMS (EI-QTOF) *m/z*: $[M]^+$ Calcd. for $C_{29}H_{42}B_2O_4$ 476.3269; Found 476.3266.

2-(2-(5-(4-methoxyphenyl)-3-methyl-3-(4,4,5,5-tetramethyl-1,3,2-dioxaborolan-2-yl)pentyl)phenyl)-4,4,5,5-tetramethyl-1,3,2-dioxaborolane (6'g)

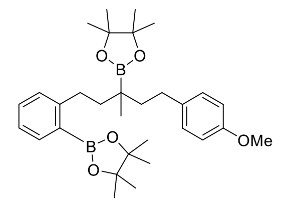

6'g

Following the **procedure D** on 0.2 mmol scale, colorless liquid, yield: 72% (72.9 mg), R_f = 0.45 (silica gel, PE: Et_2O = 40:1, v/v), column chromatography (silica gel, PE: Et_2O = 90:1, v/v).

^{1}H NMR (500 MHz, CDCl$_3$) δ 7.83 (d, *J* = 6.3 Hz, 1H), 7.41 (td, *J* = 7.5, 1.3 Hz, 1H), 7.33 (t, *J* = 7.5 Hz, 3H), 7.27 (d, *J* = 1.8 Hz, 2H), 7.24 – 7.20 (m, 2H), 3.07 (td, *J* = 12.6, 4.6 Hz, 1H), 2.83 (td, *J* = 12.6, 4.4 Hz, 1H), 2.74 – 2.61 (m, 2H), 1.85 (td, *J* = 12.8, 4.9 Hz, 1H), 1.77 (td, *J* = 12.9, 4.5 Hz, 1H), 1.66 (dd, *J* = 8.6, 3.7 Hz, 2H), 1.37 (s, 12H), 1.36 (s, 12H), 1.19 (s, 3H).

^{13}C NMR (126 MHz, CDCl$_3$) δ 150.83 (s), 143.91 (s), 136.12 (s), 131.07 (s), 129.49 (s), 128.49 (s), 128.32 (s), 125.53 (s), 124.87 (s), 83.47 (s), 83.16 (s), 42.64 (s), 41.82 (s), 32.80 (s), 32.03 (s), 25.06 (s), 24.95 (s), 21.26 (s).

^{11}B NMR (128 MHz, CDCl$_3$) δ 35.61 (s), 32.08 (s).

HRMS (EI-QTOF) *m/z*: $[M]^+$ Calcd. for $C_{30}H_{44}B_2O_5$ 506.3375; Found 506.3380.

4,4,5,5-tetramethyl-2-(2-(2-(1-(4,4,5,5-tetramethyl-1,3,2-dioxaborolan-2-yl)cyclopentyl)ethyl)phenyl)-1,3,2-dioxaborolane (6'h)

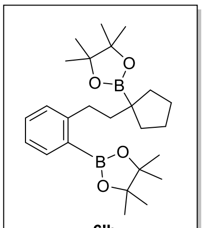

6'h

Following the **procedure D** on 0.2 mmol scale, whtie solid (m. p. = 63.1-64.8 ºC), yield: 65% (55.4 mg), R_f = 0.5 (silica gel, PE: Et_2O = 40:1, v/v), column chromatography (silica gel, PE: Et_2O = 100:1, v/v).

^{1}H NMR (500 MHz, $CDCl_3$) δ 7.76 (dd, *J* = 7.4, 0.8 Hz, 1H), 7.34 (td, *J* = 7.6, 1.3 Hz, 1H), 7.20 (d, *J* = 7.6 Hz, 1H), 7.15 (t, *J* = 7.4 Hz, 1H), 2.87 – 2.82 (m, 2H), 1.87 – 1.81 (m, 2H), 1.67 – 1.60 (m, 4H), 1.59 – 1.52 (m, 2H), 1.50 – 1.43 (m, 2H), 1.34 (s, 12H), 1.28 (s, 12H).

^{13}C NMR (126 MHz, $CDCl_3$) δ 150.62 (s), 135.89 (s), 130.88 (s), 129.34 (s), 124.70 (s), 83.33 (s), 82.86 (s), 42.32 (s), 34.83 (s), 34.25 (s), 25.52 (s), 24.86 (s), 24.76 (s).

^{11}B NMR (128 MHz, $CDCl_3$) δ 34.97 (s), 31.08 (s).

HRMS (EI-QTOF) *m/z*: $[M]^+$ Calcd. for $C_{25}H_{40}B_2O_4$ 426.3113; Found 426.3112.

4,4,5,5-tetramethyl-2-(2-(2-(1-(4,4,5,5-tetramethyl-1,3,2-dioxaborolan-2-yl)cyclohexyl)ethyl)phenyl)-1,3,2-dioxaborolane (6'i)

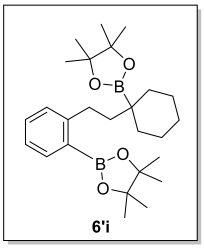

6'i

Following the **procedure D** on 0.2 mmol scale, white solid (m. p. = 75.0-76.9 ºC), yield: 74% (65.1 mg), R_f = 0.5 (silica gel, PE: Et_2O = 40:1, v/v), column chromatography (silica gel, PE: Et_2O = 100:1, v/v).

^{1}H NMR (500 MHz, $CDCl_3$) δ 7.75 (d, *J* = 7.6 Hz, 1H), 7.33 (t, *J* = 7.4 Hz, 1H), 7.18 – 7.13 (m, 2H), 2.91 – 2.81 (m, 2H), 1.97 (d, *J* = 12.8 Hz, 2H), 1.74 – 1.59 (m, 4H),

1.57 – 1.48 (m, 2H), 1.35 (s, 12H), 1.31 (s, 12H), 1.23 – 1.13 (m, 2H), 1.07 (td, *J* = 12.6, 2.9 Hz, 2H).

^{13}C NMR (126 MHz, CDCl$_3$) δ 150.81 (s), 135.91 (s), 130.87 (s), 129.29 (s), 124.70 (s), 83.36 (s), 82.89 (s), 44.40 (s), 35.07 (s), 32.00 (s), 26.82 (s), 25.33 (s), 24.95 (s), 24.88 (s).

^{11}B NMR (128 MHz, CDCl$_3$) δ 35.04 (s), 30.98 (s).

HRMS (EI-QTOF) *m/z*: [M]$^+$ Calcd. for $C_{26}H_{42}B_2O_4$ 440.3269; Found 440.3271.

2-(2-(4,4-dimethyl-1-phenylpentan-2-yl)phenyl)-4,4,5,5-tetramethyl-1,3,2-dioxaborolane (8a)

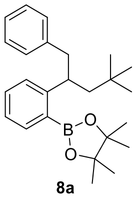

8a

Following the **procedure D** on 0.2 mmol scale, colorless liquid, yield: 70% (52.9 mg), R_f = 0.5 (silica gel, PE: Et_2O = 40:1, v/v), column chromatography (silica gel, PE: Et_2O = 100:1, v/v).

^{1}H NMR (500 MHz, CDCl$_3$) δ 7.73 (d, *J* = 7.4 Hz, 1H), 7.40 (d, *J* = 5.4 Hz, 2H), 7.22 – 7.17 (m, 2H), 7.14 (dd, *J* = 10.4, 4.3 Hz, 4H), 3.90-4.05 (m, 1H), 2.84 (dd, *J* = 13.2, 6.1 Hz, 1H), 2.65 (dd, *J* = 13.2, 8.8 Hz, 1H), 1.79 (dd, *J* = 14.0, 9.4 Hz, 1H), 1.58 – 1.52 (m, 2H), 1.35 (s, 6H), 1.33 (s, 6H), 0.67 (s, 9H).

^{13}C NMR (126 MHz, CDCl$_3$) δ 154.83 (s), 141.39 (s), 135.97 (s), 130.79 (s), 129.51 (s), 127.69 (s), 126.60 (s), 125.45 (s), 124.66 (s), 83.35 (s), 48.24 (s), 47.06 (s), 31.41 (s), 30.06 (s), 25.11 (s), 24.78 (s).

^{11}B NMR (128 MHz, CDCl$_3$) δ 31.41 (s).

HRMS (EI-QTOF) *m/z*: [M]$^+$ Calcd. for $C_{25}H_{35}BO_2$ 378.2730; Found 378.2733.

2-(2-(1-(4-fluorophenyl)-4,4-dimethylpentan-2-yl)phenyl)-4,4,5,5-tetramethyl-1,3,2-dioxaborolane (8b)

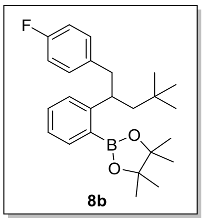

8b

Following the **procedure D** on 0.2 mmol scale, colorless liquid, yield: 74% (58.6 mg), R_f = 0.5 (silica gel, PE: Et_2O = 40:1, v/v), column chromatography (silica gel, PE: Et_2O = 100:1, v/v).

^{1}H NMR (500 MHz, CDCl$_3$) δ 7.73 (d, *J* = 7.4 Hz, 1H), 7.39 (dt, *J* = 13.9, 7.2 Hz, 2H), 7.14 (td, *J* = 7.4, 1.0 Hz, 1H), 7.06 (dd, *J* = 8.3, 5.7 Hz, 2H), 6.86 (t, *J* = 8.7 Hz, 2H), 3.90-4.03 (m, 1H), 2.79 (dd, *J* = 13.3, 6.5 Hz, 1H), 2.65 (dd, *J* = 13.3, 8.4 Hz, 1H), 1.79 (dd, *J* = 14.0, 9.3 Hz, 1H), 1.53 (dd, *J* = 14.0, 2.4 Hz, 1H), 1.34 (s, 6H), 1.32 (s, 6H), 0.68 (s, 9H).

^{13}C NMR (126 MHz, CDCl$_3$) δ 161.13 (d, *J* = 242.7 Hz), 154.49 (s), 136.98 (d, *J* = 2.9 Hz), 136.03 (s), 130.85 (s), 130.75 (d, *J* = 7.6 Hz), 126.55 (s), 124.75 (s), 114.34 (d, *J* = 20.8 Hz), 83.36 (s), 48.41 (s), 46.20 (s), 31.42 (s), 30.05 (s), 25.09 (s), 24.75 (s).

^{19}F NMR (471 MHz, CDCl$_3$) δ -118.25 (s).

^{11}B NMR (160 MHz, CDCl$_3$) δ 31.42 (s).

HRMS (EI-QTOF) *m/z*: $[M]^+$ Calcd. for $C_{25}H_{34}BFO_2$ 396.2636; Found 396.2639.

2-(2-(4,4-dimethyl-1-(p-tolyl)pentan-2-yl)phenyl)-4,4,5,5-tetramethyl-1,3,2-dioxaborolane (8c)

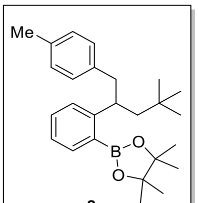

8c

Following the **procedure D** on 0.2 mmol scale, colorless liquid, yield: 64% (50.2 mg), R_f = 0.5 (silica gel, PE: Et_2O = 40:1, v/v), column chromatography (silica gel, PE: Et_2O = 100:1, v/v).

^{1}H NMR (500 MHz, CDCl$_3$) δ 7.72 (d, *J* = 7.8 Hz, 1H), 7.43 – 7.35 (m, 2H), 7.16 – 7.11 (m, 1H), 7.02 (dd, *J* = 15.9, 7.7 Hz, 4H), 4.03 – 3.83 (m, 1H), 2.80 (dd, *J* = 13.3,

6.0 Hz, 1H), 2.61 (dd, *J* = 13.2, 8.9 Hz, 1H), 2.29 (s, 3H), 1.77 (dd, *J* = 14.0, 9.6 Hz, 1H), 1.54 (dd, *J* = 13.9, 2.3 Hz, 1H), 1.35 (s, 6H), 1.32 (s, 6H), 0.67 (s, 9H).

^{13}C NMR (126 MHz, $CDCl_3$) δ 154.96 (s), 138.25 (s), 135.91 (s), 134.73 (s), 130.75 (s), 129.34 (s), 128.39 (s), 126.60 (s), 124.61 (s), 83.33 (s), 48.17 (s), 46.64 (s), 31.40 (s), 30.08 (s), 25.08 (s), 24.75 (s), 20.99 (s).

^{11}B NMR (160 MHz, $CDCl_3$) δ 31.27 (s).

HRMS (EI-QTOF) *m/z*: $[M]^+$ Calcd. for $C_{26}H_{37}BO_2$ 392.2887; Found 392.2890.

2-(2-(1-(3-bromophenyl)-4,4-dimethylpentan-2-yl)phenyl)-4,4,5,5-tetramethyl-1,3,2-dioxaborolane (8d)

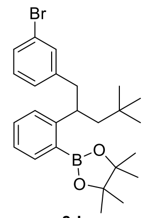

8d

Following the **procedure D** on 0.2 mmol scale, colorless liquid, yield: 66% (60.3 mg), R_f = 0.5 (silica gel, PE: Et_2O = 40:1, v/v), column chromatography (silica gel, PE: Et_2O = 100:1, v/v).

^{1}H NMR (400 MHz, $CDCl_3$) δ 7.75 (d, *J* = 7.4 Hz, 1H), 7.40 (dd, *J* = 22.4, 10.0 Hz, 3H), 7.28 (s, 1H), 7.16 (t, *J* = 7.0 Hz, 1H), 7.05 (t, *J* = 7.6 Hz, 1H), 6.97 (d, *J* = 7.4 Hz, 1H), 4.00 (d, *J* = 5.6 Hz, 1H), 2.76 – 2.76 (m, 1H), 2.67 – 2.48 (m, 1H), 1.84 – 1.73 (m, 1H), 1.53 – 1.42 (m, 1H), 1.38 (s, 6H), 1.35 (s, 6H), 0.67 (s, 9H).

^{13}C NMR (126 MHz, $CDCl_3$) δ 154.29 (s), 143.86 (s), 136.15 (s), 132.34 (s), 130.91 (s), 129.20 (s), 128.61 (s), 128.20 (s), 126.42 (s), 124.87 (s), 122.00 (s), 83.50 (s), 48.06 (s), 46.57 (s), 31.38 (s), 30.04 (s), 25.16 (s), 24.76 (s).

^{11}B NMR (160 MHz, $CDCl_3$) δ 31.33 (s).

HRMS (EI-QTOF) *m/z*: $[M]^+$ Calcd. for $C_{25}H_{34}BBrO_2$ 456.1835; Found 456.1832.

2-(2-(1-(3-methoxyphenyl)-4,4-dimethylpentan-2-yl)phenyl)-4,4,5,5-tetramethyl-1,3,2-dioxaborolane (8e)

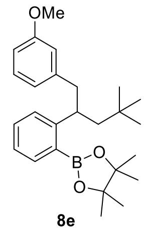

8e

Following the **procedure D** on 0.2 mmol scale, colorless liquid, yield: 67% (54.6 mg), R_f = 0.45 (silica gel, PE: Et_2O = 40:1, v/v), column chromatography (silica gel, PE: Et_2O = 90:1, v/v).

^{1}H NMR (400 MHz, $CDCl_3$) δ 7.73 (d, *J* = 7.3 Hz, 1H), 7.39 (s, 2H), 7.17 – 7.03 (m, 2H), 6.75 (d, *J* = 7.3 Hz, 1H), 6.67 (d, *J* = 13.4 Hz, 2H), 4.01 (d, *J* = 5.9 Hz, 1H), 3.72 (s, 3H), 2.84 – 2.78 (m, 1H), 2.68 – 2.59 (m, 1H), 1.84 – 1.73 (m, 1H), 1.57 (s, 1H), 1.34 (s, 6H), 1.32 (s, 6H), 0.69 (s, 9H).

^{13}C NMR (126 MHz, $CDCl_3$) δ 159.15 (s), 154.74 (s), 142.94 (s), 135.97 (s), 130.77 (s), 128.57 (s), 124.67 (s), 122.04 (s), 115.32 (s), 110.79 (s), 83.35 (s), 55.05 (s), 48.32 (s), 47.09 (s), 31.43 (s), 30.09 (s), 25.07 (s), 24.74 (s).

^{11}B NMR (128 MHz, $CDCl_3$) δ 31.69 (s).

HRMS (EI-QTOF) *m/z*: $[M]^+$ Calcd. for $C_{26}H_{37}BO_3$ 408.2836; Found 408.2844.

***2*-(2-(6,6-dimethylhept-1-en-4-yl)phenyl)-4,4,5,5-tetramethyl-1,3,2-dioxaborolane (8f)**

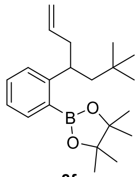

8f

Following the **procedure D** on 0.2 mmol scale, colorless liquid, yield: 75% (49.2 mg), R_f = 0.5 (silica gel, PE: Et_2O = 40:1, v/v), column chromatography (silica gel, PE: Et_2O = 100:1, v/v).

^{1}H NMR (500 MHz, $CDCl_3$) δ 7.72 (d, *J* = 7.4 Hz, 1H), 7.38 – 7.34 (m, 1H), 7.28 (d, *J* = 7.8 Hz, 1H), 7.13 (t, *J* = 7.2 Hz, 1H), 5.66 (ddt, *J* = 17.2, 10.2, 7.0 Hz, 1H), 4.93 –

4.84 (m, 2H), 3.77 – 3.69 (m, 1H), 2.22 (t, *J* = 7.1 Hz, 2H), 1.76 (dd, *J* = 14.0, 9.1 Hz, 1H), 1.58 – 1.55 (m, 1H), 1.35 (s, 12H), 0.77 (s, 9H).

^{13}C NMR (126 MHz, $CDCl_3$) δ 154.40 (s), 137.78 (s), 135.55 (s), 130.63 (s), 126.41 (s), 124.60 (s), 115.33 (s), 83.36 (s), 48.90 (s), 45.08 (s), 38.72 (s), 31.53 (s), 30.16 (s), 24.88 (s).

^{11}B NMR (128 MHz, $CDCl_3$) δ 31.65 (s).

HRMS (EI-QTOF) *m/z*: $[M]^+$ Calcd. for $C_{21}H_{33}BO_2$ 328.2574; Found 328.2572.

4,4,5,5-tetramethyl-2-(2-(2,6,6-trimethylhept-1-en-4-yl)phenyl)-1,3,2-dioxaborolane (8g)

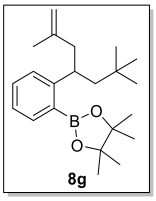

Following the **procedure D** on 0.2 mmol scale, colorless liquid, yield: 71% (52.9 mg), R_f = 0.5 (silica gel, PE: Et_2O = 40:1, v/v), column chromatography (silica gel, PE: Et_2O = 100:1, v/v).

^{1}H NMR (500 MHz, $CDCl_3$) δ 7.76 (d, *J* = 7.2 Hz, 1H), 7.37 (t, *J* = 7.3 Hz, 1H), 7.31 (d, *J* = 7.8 Hz, 1H), 7.14 (t, *J* = 7.2 Hz, 1H), 4.70 (s, 1H), 4.62 (s, 1H), 3.76-3.93 (m, 1H), 2.23 – 2.10 (m, 2H), 1.75 (s, 3H), 1.61 – 1.55 (m, 2H), 1.35 (s, 12H), 0.75 (s, 9H).

^{13}C NMR (126 MHz, $CDCl_3$) δ 155.35 (s), 144.88 (s), 135.98 (s), 130.73 (s), 126.47 (s), 124.55 (s), 112.04 (s), 83.36 (s), 49.27 (s), 48.02 (s), 31.28 (s), 30.25 (s), 25.04 (s), 24.79 (s), 22.17 (s).

^{11}B NMR (128 MHz, $CDCl_3$) δ 31.51 (s).

HRMS (EI-QTOF) *m/z*: $[M]^+$ Calcd. for $C_{22}H_{35}BO_2$ 342.2730; Found 342.2734.

2-(2-(1-(cyclohex-2-en-1-yl)-3,3-dimethylbutyl)phenyl)-4,4,5,5-tetramethyl-1,3,2-dioxaborolane (8h)

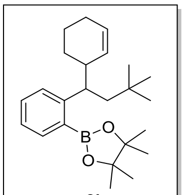

8h

Following the **procedure D** on 0.2 mmol scale, colorless liquid, yield: 56% (44.2 mg), R_f = 0.5 (silica gel, PE: Et_2O = 40:1, v/v), column chromatography (silica gel, PE: Et_2O = 100:1, v/v).

^{1}H NMR (500 MHz, $CDCl_3$) δ 7.75 (d, J = 7.1 Hz, 1H), 7.38 – 7.31 (m, 2H), 7.14 (dt, J = 8.4, 2.4 Hz, 1H), 5.83 (d, J = 10.2 Hz, 0.5H), 5.72 – 5.67 (m, 0.5H), 5.67 – 5.60 (m, 0.5H), 5.43 – 5.35 (m, 0.5H), 3.77 – 3.50 (m, 1H), 1.94 – 1.88 (m, 2H), 1.80 (td, J = 14.5, 10.1 Hz, 1H), 1.67 – 1.54 (m, 2H), 1.42 – 1.37 (m, 2H), 1.35 (s, 12H), 1.30 – 1.26 (m, 2H), 0.76 (s, 9H).

^{13}C NMR (126 MHz, $CDCl_3$) δ 153.15 (s), 135.84 (s), 132.07 (s), 130.08 (s), 127.61 (s), 127.27 (s), 124.60 (s), 83.45 (s), 44.65 (s), 43.50 (s), 42.78 (s), 31.63 (s), 30.23 (s), 28.00 (s), 25.59 (s), 24.97 (s), 21.79 (s).

^{11}B NMR (160 MHz, $CDCl_3$) δ 32.01 (s).

HRMS (EI-QTOF) *m/z*: $[M]^+$ Calcd. for $C_{24}H_{37}BO_2$ 368.2887; Found 368.2884.

2.5 Scale-up reactions and synthetic applications.

2.5.1 10 times scale-up reactions.

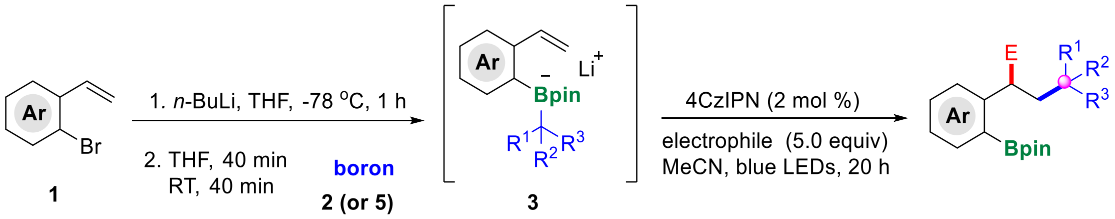

Supplementary Figure 7. Scale-up reactions for aryl boronic acid pinocol esters

To a flame-dried Schlenk tube were added **1p (or 1a)** (2.6 mmol, 1.3 equiv) and THF (5 mL) and the resulting solution was cooled to -78 °C. Subsequently, *n*-BuLi (1.6 mL, 1.6 M in hexane, 2.6 mmol, 1.3 equiv) was added dropwise and the resulting mixture was stirred at -78 °C for 1 h, before dropwise addition of **2a (or 5a)** (in 1 mL THF) to the solution of aryllithium reagent. And the resulting mixture was stirred at -78 °C for

40 mins, then allowed it to warm to ambient temperature and stirred for another 40 mins. Next, the THF was carefully removed in vacuo and the 4CzIPN (32 mg, 0.04 mmol, 2 mol%), electrophile (10 mmol, 5.0 equiv), MeCN (20 mL) were added to Schlenk tube under Ar, after which the Schlenk tube was sealed with parafilm and the mixture was stirred vigorously under blue LED irradiation for 20 h. The reaction mixture was diluted with EtOAC and the solution washed with saturated aqueous NH_4Cl, water and brine. The combined organic layers were dried over Na_2SO_4 and concentrated under reduced pressure. The crude product was then purified by flash column chromatography.

2.5.2 Synthetic applications.

a.

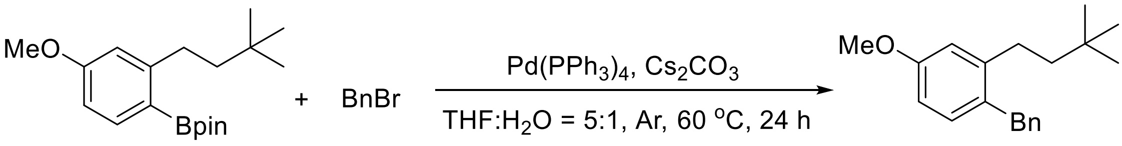

4p **9**

Supplementary Figure 8. Deboronation and bromination reaction

To a flame-dried Schlenk tube were added **4p** (0.20 mmol), $Pd(PPh_3)_4$ (0.005 equiv), and Cs_2CO_3 (3 equiv) in 2.0 mL of THF/H_2O (v/v = 20:1) and added BnBr (1.2 equiv) under argon atmosphere. After stirring at 60 °C for 24 h, the reaction mixture was quenched with water, extracted with EtOAc, washed with brine, dried over anhydrous Na_2SO_4, and concentrated. The crude product was purified by silica gel chromatography (silica gel, PE: EA = 100:1, v/v) to afford the product **9** as a colorless oil liquid with the yield of 84%.

1-benzyl-2-(3,3-dimethylbutyl)-4-methoxybenzene (9)[8]

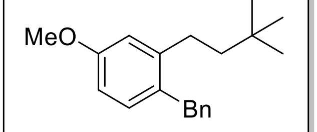

9

^{1}H NMR (500 MHz, CDCl$_3$) δ 7.25 – 7.22 (m, 1H), 7.16 (t, *J* = 7.3 Hz, 1H), 7.12 – 7.07 (m, 2H), 7.00 (d, *J* = 8.2 Hz, 1H), 6.72 (t, *J* = 3.0 Hz, 1H), 6.70 – 6.65 (m, 1H), 3.93 (s, 2H), 3.78 (s, 3H), 2.49 – 2.42 (m, 2H), 1.37 – 1.30 (m, 2H), 0.88 (s, 9H).

^{13}C NMR (126 MHz, CDCl$_3$) δ 158.24 (s), 143.18 (s), 141.42 (s), 131.39 (s), 130.48 (s), 128.61 (s), 128.31 (s), 125.81 (s), 115.09 (s), 110.73 (s), 55.16 (s), 45.58 (s), 38.05 (s), 30.50 (s), 29.19 (s), 28.44 (s).

HRMS (EI-QTOF) *m/z*: $[M]^+$ Calcd. for $C_{20}H_{26}O$ 282.1984; Found 282.1992.

b.

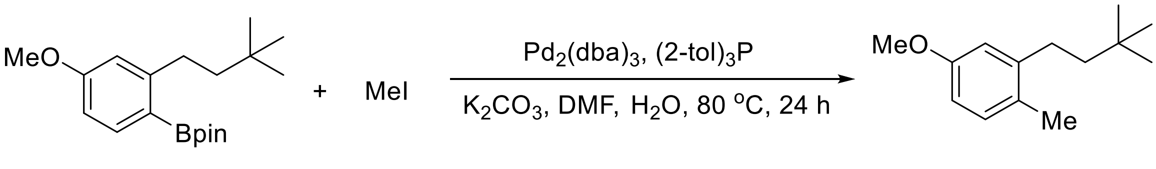

Supplementary Figure 9. Deboronmethylation reaction

To a flame-dried Schlenk tube were added **4p** (0.20 mmol), $Pd_2(dba)_3$ (0.05 equiv), (2-tol)$_3$P (0.1 equiv) and K_2CO_3 (3 equiv) in 2.0 mL of DMF/H_2O (v/v = 20:1) and added MeI (1.2 equiv) under argon atmosphere. After stirring at 80 °C for 24 h, the reaction mixture was quenched with water, extracted with EtOAc, washed with brine, dried over anhydrous Na_2SO_4, and concentrated. The crude product was purified by silica gel chromatography (silica gel, PE: EA = 100:1, v/v) to afford the product **10** as a colorless oil liquid with the yield of 78%.

2-(3,3-dimethylbutyl)-4-methoxy-1-methylbenzene (10)[8]

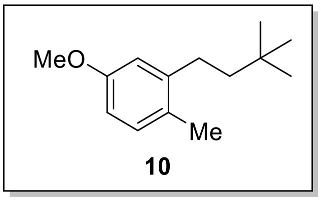

^{1}H NMR (500 MHz, CDCl$_3$) δ 7.05 (d, *J* = 8.3 Hz, 1H), 6.72 (d, *J* = 2.4 Hz, 1H), 6.66 (dd, *J* = 8.3, 2.6 Hz, 1H), 3.80 (s, 3H), 2.57 – 2.47 (m, 2H), 2.25 (s, 3H), 1.48 – 1.39 (m, 2H), 1.00 (s, 9H).

^{13}C NMR (126 MHz, CDCl$_3$) δ 157.87 (s), 142.86 (s), 130.82 (s), 127.73 (s), 114.61 (s), 110.58 (s), 55.20 (s), 44.95 (s), 30.58 (s), 29.24 (s), 28.86 (s), 18.19 (s).

HRMS (EI-QTOF) *m/z*: $[M]^+$ Calcd. for $C_{14}H_{22}O$ 206.1671; Found 206.1673.

c.

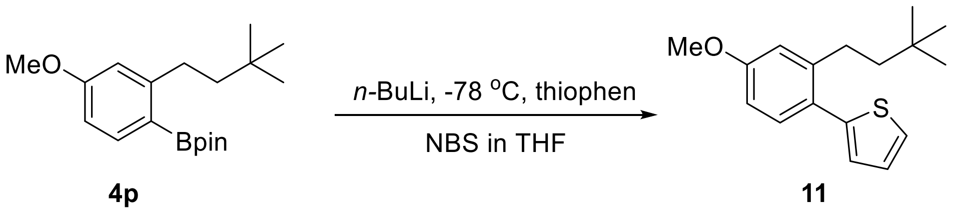

Supplementary Figure 10. Deboronation and thiophenation reaction

To a flame-dried Schlenk tube were added thiophen (0.5 mmol, 2.5 equiv) in THF (2.0 mL) was cooled to -78 °C and treated with *n*-BuLi (1.6 M in hexane, 0.6 mmol, 3 equiv). The cooling bath was removed and the mixture was stirred at room temperature for 1 h. The mixture was cooled to -78 °C and a solution of **4p** (0.2 mmol, 1.0 equiv) in THF (1 mL) was added. The mixture was stirred at -78 °C for 1 h, and then a solution of NBS (0.5 mmol, 2.5 equiv) in THF (2.0 mL) was added. After 10 h at room temperature, saturated $Na_2S_2O_3$ (aq.) (4 mL) was added. The reaction mixture was diluted with water and extracted with ethyl acetate. The combined organic layers were dried over Na_2SO_4 and concentrated in vacuo. The crude product was purified by silica gel chromatography (silica gel, PE: EA = 100:1, v/v) to afford the product **11** as a colorless oil liquid in 70%.

2-(2-(3,3-dimethylbutyl)-4-methoxyphenyl)furan (11)[9]

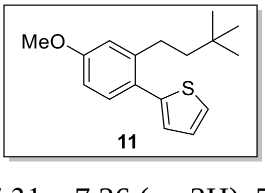

^{1}H NMR (500 MHz, CDCl$_3$) δ 7.31 – 7.26 (m, 2H), 7.06 (dd, *J* = 5.1, 3.5 Hz, 1H), 6.97 (dd, *J* = 3.4, 1.1 Hz, 1H), 6.81 (d, *J* = 2.6 Hz, 1H), 6.76 (dd, *J* = 8.4, 2.7 Hz, 1H), 3.84 (s, 3H), 2.67 – 2.59 (m, 2H), 1.44 – 1.38 (m, 2H), 0.85 (s, 9H).

^{13}C NMR (126 MHz, CDCl$_3$) δ 159.42 (s), 143.72 (s), 142.63 (s), 132.24 (s), 126.77 (s), 126.34 (s), 126.17 (s), 124.71 (s), 115.10 (s), 110.82 (s), 55.26 (s), 46.22 (s), 30.59 (s), 29.22 (s), 29.16 (s).

HRMS (EI-QTOF) ***m/z*****:** $[M]^+$ Calcd. for Chemical Formula: $C_{17}H_{22}OS$ 274.1391; Found 274.1394.

d.

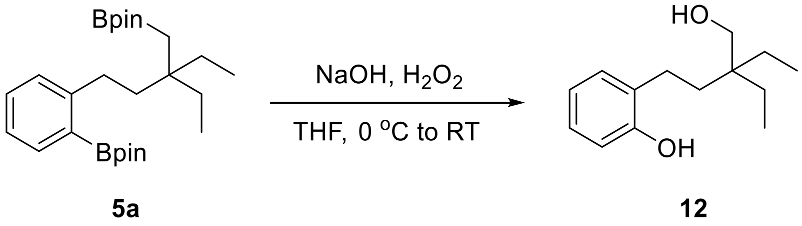

Supplementary Figure 11. Oxidation of borons to hydroxyl

To a flame-dried Schlenk tube equipped with a magnetic stirring bar was charged with **5a** (0.2 mmol) and THF (5 mL). The solution was cooled to 0 °C, then 3M NaOH (4 mL) was added, followed by 30% H_2O_2 (2 mL) dropwise and stirring for 4 h at room temperature. The crude product was purified by silica gel chromatography (silica gel, PE: EA = 2:1, v/v) to afford the product **12** as a colorless liquid with the yield of 88%.

3-(3-ethyl-3-(hydroxymethyl)pentyl)phenol (12)[10]

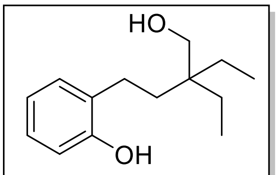

12

^{1}H NMR (400 MHz, CDCl$_3$) δ 7.09 (t, *J* = 8.2 Hz, 2H), 6.93 (s, 1H), 6.83 (t, *J* = 7.4 Hz, 2H), 3.59 (s, 2H), 2.59 – 2.45 (m, 2H), 2.36 (s, 1H), 1.56 – 1.43 (m, 2H), 1.37 – 1.19 (m, 4H), 0.83 (t, *J* = 7.4 Hz, 6H).

^{13}C NMR (126 MHz, CDCl$_3$) δ 153.97 (s), 129.89 (s), 128.79 (s), 127.33 (s), 120.27 (s), 115.74 (s), 66.81 (s), 39.68 (s), 34.05 (s), 25.33 (s), 24.65 (s), 7.34 (s).

HRMS (EI-QTOF) ***m/z*****:** $[M]^+$ Calcd. for $C_{14}H_{22}O_2$ 222.1620; Found 222.1619.

e.

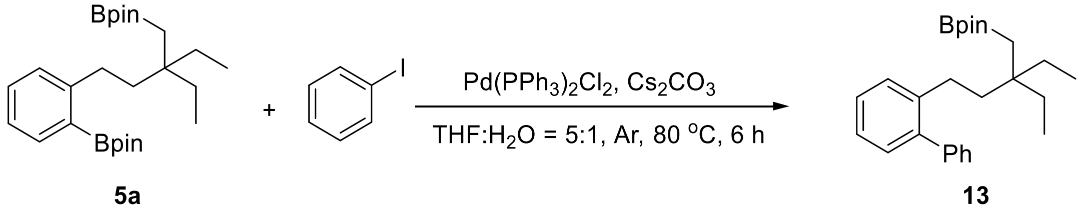

Supplementary Figure 12. Pd-catalyzed coupling reaction

To a flame-dried Schlenk tube were added **5a** (0.20 mmol), $Pd(PPh_3)_2Cl_2$ (0.5 mol%), and Cs_2CO_3 (0.60 mmol, 3 equiv) in 2.0 mL of THF/H_2O (v/v = 5:1) and added iodobenzene (0.24 mmol, 1.2 equiv) under argon atmosphere. After stirring at 80 °C for 20 h, the reaction mixture was quenched with water, extracted with EtOAc, washed with brine, dried over anhydrous Na_2SO_4, and concentrated. The crude product was purified by silica gel chromatography (silica gel, PE: EA = 100:1, v/v) to afford the product **13** as a colorless oil liquid with the yield of 92%.

2-(4-([1,1'-biphenyl]-2-yl)-2,2-diethylbutyl)-4,4,5,5-tetramethyl-1,3,2-dioxaborolane (13) [8]

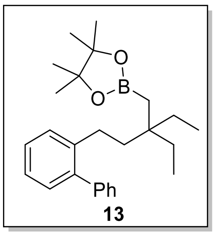

^{1}H NMR (500 MHz, CDCl$_3$) δ 7.39 (dd, *J* = 7.7, 5.3 Hz, 2H), 7.32 (ddd, *J* = 6.9, 4.3, 3.1 Hz, 4H), 7.28 (dd, *J* = 7.6, 2.0 Hz, 1H), 7.21 (dd, *J* = 8.4, 1.6 Hz, 2H), 2.50 – 2.46 (m, 2H), 1.42 – 1.38 (m, 2H), 1.23 (s, 12H), 1.20 – 1.15 (m, 6H), 0.68 (s, 2H), 0.60 (t, *J* = 7.5 Hz, 6H).

^{13}C NMR (126 MHz, CDCl$_3$) δ 141.92 (s), 141.78 (s), 141.45 (s), 129.90 (s), 129.61 (s), 129.29 (s), 127.93 (s), 127.28 (s), 126.67 (s), 125.28 (s), 82.56 (s), 40.47 (s), 37.50 (s), 30.48 (s), 27.34 (s), 24.86 (s), 7.74 (s).

^{11}B NMR (160 MHz, CDCl$_3$) δ 34.24 (s).

HRMS (EI-QTOF) *m/z*: $[M]^+$ Calcd. for $C_{26}H_{37}BO_2$ 392.2887; Found 382.2886.

f.

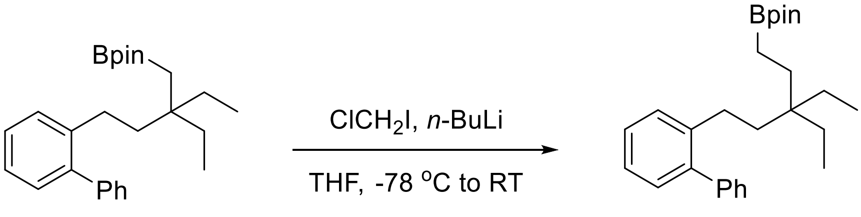

13 **14**

Supplementary Figure 13. Carbon-adding reaction

To a 25 mL Schlenk tube containing a magnetic stirbar, **13** (0.20 mmol, 1.0 equiv) and chloroiodomethane (0.6 mmol, 3.0 equiv) and THF (2.0 mL) were added. The reaction mixture was cooled to –78 °C and a solution of *n*-BuLi (0.6 mmol, 1.6 M in hexane, 3.0 equiv) was slowly added at -78 °C. After stirring for 1 h, the reaction mixture was warmed to room temperature and additionally stirred for 24 h. The reaction was quenched with a saturated aqueous NH_4Cl solution and extracted with EtOAc. The combined organic layers were dried over Na_2SO_4, filtered and concentrated under reduced pressure. The crude mixture was purified by column chromatography (silica gel, PE: EA = 100:1, v/v) to give the corresponding product **14** as a light yellow oil with the yield of 82%.

2-(5-([1,1'-biphenyl]-2-yl)-3,3-diethylpentyl)-4,4,5,5-tetramethyl-1,3,2-dioxaborolane (14)[11]

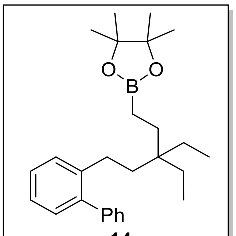

14

^{1}H NMR (500 MHz, $CDCl_3$) δ 7.39 – 7.35 (m, 3H), 7.31 (t, *J* = 6.2 Hz, 4H), 7.22 – 7.17 (m, 2H), 2.44 – 2.40 (m, 2H), 1.23 (s, 12H), 1.18 – 1.13 (m, 4H), 1.06 (dd, *J* = 15.0, 7.4 Hz, 4H), 0.56 (t, *J* = 7.5 Hz, 6H), 0.48 – 0.42 (m, 2H)

^{13}C NMR (126 MHz, CDCl$_3$) δ 141.85 (s), 141.74 (s), 141.26 (s), 129.91 (s), 129.47 (s), 129.24 (s), 127.96 (s), 127.38 (s), 126.71 (s), 125.34 (s), 82.75 (s), 37.76 (s), 37.48 (s), 28.55 (s), 27.13 (s), 26.98 (s), 24.78 (s), 7.25 (s).

^{11}B NMR (128 MHz, CDCl$_3$) δ 35.36 (s).

HRMS (EI-QTOF) *m/z*: $[M]^+$ Calcd. for $C_{27}H_{39}BO_2$ 406.3043; Found 406.3052.

g.

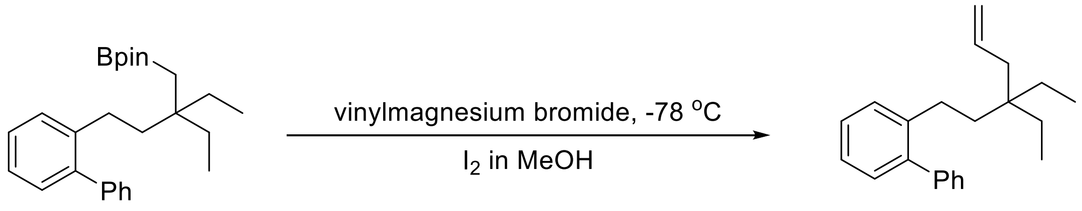

13 **15**

Supplementary Figure 14. Deboronation and vinylation reaction

To a solution of substrate **13** (0.2 mmol in 2 mL THF) was added vinylmagnesium bromide solution (1.0 M in THF, 0.8 mmol) and the reaction mixture was stirred for 1 h at –78 °C. A solution of iodine (0.8 mmol, in 1 mL MeOH) was then added dropwise to the reaction mixture, 0.5 h later followed by a solution of MeONa (1.6 mmol) in MeOH (3.5 mL). The reaction mixture was then allowed to warm to room temperature and stirred for an additional one hour, and then quenched with sat. $Na_2S_2O_3$ (aq.) and extracted with ethyl acetate. The combined organic layers were dried over sodium sulfate and concentrated in vacuo. The crude product was purified by silica gel chromatography (silica gel, PE: EA = 100:1, v/v) to afford the product **15** as a colorless oil in 62%.

2-(3,3-diethylhex-5-en-1-yl)-1,1'-biphenyl (15)[11]

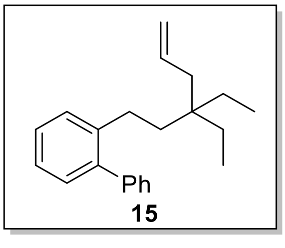

^{1}H NMR (500 MHz, CDCl$_3$) δ 7.40 (t, *J* = 7.3 Hz, 2H), 7.35 – 7.26 (m, 5H), 7.20 (ddd, *J* = 12.4, 7.1, 1.3 Hz, 2H), 5.59 – 5.50 (m, 1H), 4.96 – 4.88 (m, 2H), 2.50 – 2.45

(m, 2H), 1.83 (d, *J* = 7.4 Hz, 2H), 1.27 – 1.25 (m, 2H), 1.10 (q, *J* = 7.5 Hz, 4H), 0.60 (t, *J* = 7.5 Hz, 6H).

^{13}C NMR (126 MHz, CDCl$_3$) δ 141.85 (s), 141.79 (s), 141.06 (s), 135.11 (s), 129.99 (s), 129.45 (s), 129.28 (s), 128.01 (s), 127.44 (s), 126.76 (s), 125.46 (s), 116.43 (s), 39.77 (s), 37.98 (s), 37.92 (s), 27.71 (s), 26.90 (s), 7.16 (s).

HRMS (EI-QTOF) *m/z*: [M]$^+$ Calcd. for $C_{22}H_{28}$ 292.2191; Found 292.2196.

2.6 Control experiments.

2.6.1 Radical capture experiment.

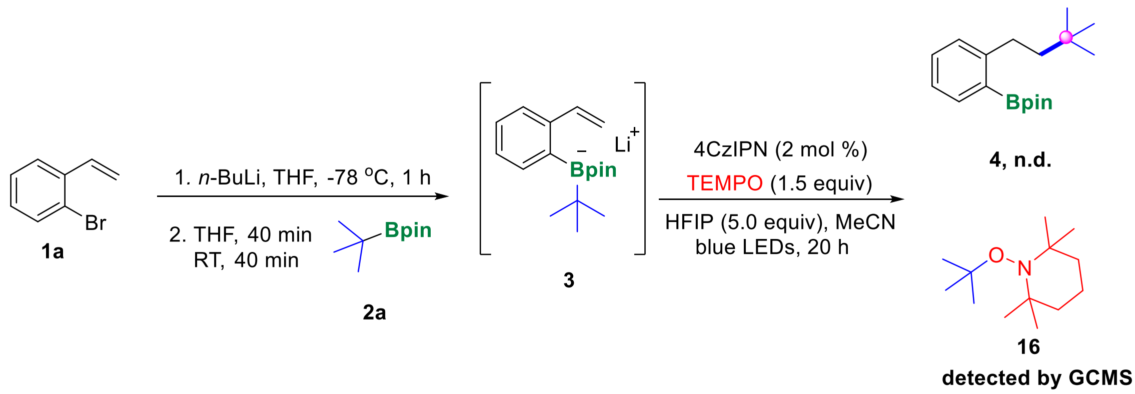

Supplementary Figure 15. Radical capture experiment

According to **general procedure D1**, to a flame-dried Schlenk tube were added *o*-bromostyrene (**1a**, 32.5 μL, 0.26 mmol, 1.3 equiv) and THF (0.6 mL) and the resulting solution was cooled to -78 °C. Subsequently, *n*-BuLi (0.16 mL, 1.6 M in hexane, 0.26 mmol, 1.3 equiv) was added dropwise and the resulting mixture was stirred at -78 °C for 1 h, before dropwise addition of **2a** (in 0.2 mL THF) to the solution of aryllithium reagent. And the resulting mixture was stirred at -78 °C for 40 mins, then allowed it to warm to ambient temperature and stirred for another 40 mins. Next, without removed the THF, the 4CzIPN (3.2 mg, 0.004 mmol, 2 mol%), 2,2-6,6-tetramethyl piperidine-*N*-oxyl (TEMPO, 0.3 mmol, 1.5 equiv), 1,1,1,3,3,3-hexafluoropropan-2-ol (HFIP, 105 μL, 1.0 mmol, 5.0 equiv), MeCN (2 mL, 0.1M) was added to Schlenk tube under Ar, after which the Schlenk tube was sealed with parafilm and the mixture was stirred vigorously under blue LED irradiation for 20 h. Upon completion, no desired product was detected by GC-MS.

1-(tert-butoxy)-2,2,6,6-tetramethylpiperidine

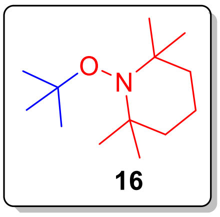

Chemical Formula: $C_{13}H_{27}NO$ Exact Mass: 213; Found 213. Detected by GC-MS.

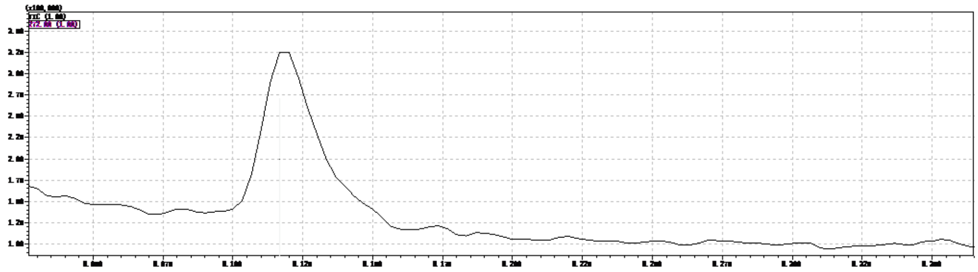

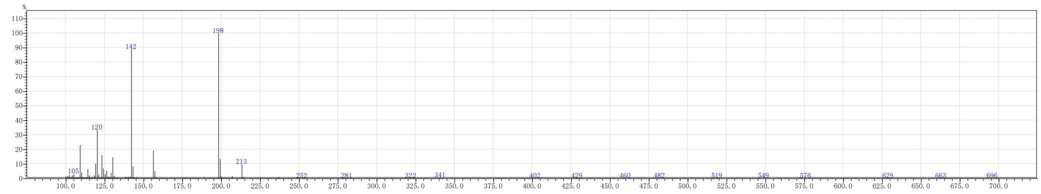

Supplementary Figure 16. The GC-MS spectrum of TEMPO-adduct 16

2.6.2 Supplementary Table 6. Deuteration experiments.

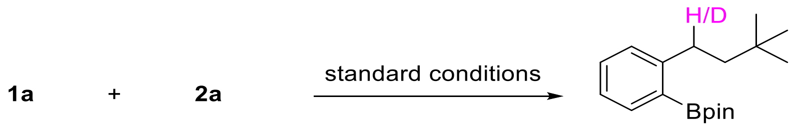

4a-D

Deuterium source	Deuteration rate
d_8-THF	0
d_3-MeCN	0
d_2-HFIP	72%

According to **general procedure D$_1$**, to a flame-dried Schlenk tube were added *o*-bromostyrene (**1a**, 32.5 μL, 0.26 mmol, 1.3 equiv) and THF (0.6 mL) and the resulting solution was cooled to -78 °C. Subsequently, *n*-BuLi (0.16 mL, 1.6 M in hexane, 0.26 mmol, 1.3 equiv) was added dropwise and the resulting mixture was stirred at -78 °C for 1 h, before dropwise addition of **2a** (in 0.2 mL THF) to the solution of aryllithium

reagent. And the resulting mixture was stirred at -78 °C for 40 mins, then allowed it to warm to ambient temperature and stirred for another 40 mins. Next, without removed the THF, the 4CzIPN (3.2 mg, 0.004 mmol, 2 mol%), 1,1,1,3,3,3-hexafluoropropan-2-ol-D2 (d_2-HFIP, 105 µL, 1.0 mmol, 5.0 equiv), MeCN (2 mL, 0.1M) was added to Schlenk tube under Ar, after which the Schlenk tube was sealed with parafilm. The reaction mixture was diluted with EtOAC and the solution washed with saturated aqueous NH_4Cl, water and brine. The combined organic layers were dried over Na_2SO_4 and concentrated under reduced pressure. The crude product was then purified by flash column chromatography. This result indirectly confirmed the existence of benzylic carbanion.

2-(2-(3,3-dimethylbutyl-1-d)phenyl)-4,4,5,5-tetramethyl-1,3,2-dioxaborolane (4a-D)

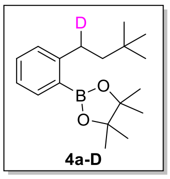

^{1}H NMR (500 MHz, $CDCl_3$) δ 7.79 (d, J = 7.1 Hz, 1H), 7.37 – 7.32 (m, 1H), 7.17 (t, J = 7.3 Hz, 2H), 2.89 – 2.81 (m, 1.18H), 1.44 (d, J = 9.2 Hz, 2H), 1.35 (s, 12H), 0.99 (s, 9H).

^{13}C NMR (126 MHz, $CDCl_3$) δ 150.81 (s), 136.08 (s), 130.91 (s), 129.25 (s), 124.72 (s), 83.35 (s), 48.08 (s), 31.28 (s), 30.81 (s), 29.44 (s), 24.88 (s).

^{11}B NMR (160 MHz, $CDCl_3$) δ 31.41 (s).

HRMS (EI-QTOF) *m/z*: $[M]^+$ Calcd for. $C_{18}H_{28}DBO_2$ 289.2323; Found 289.2330.

2.6.3 Radical-clock experiments[6].

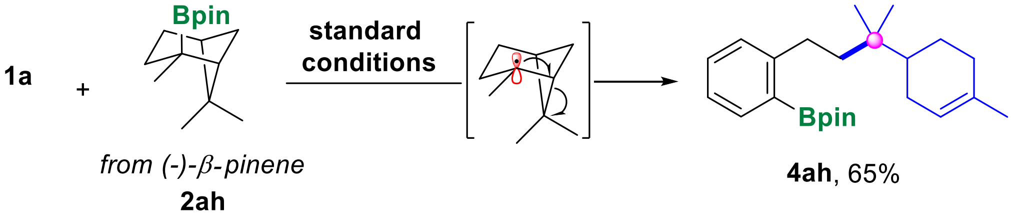

Supplementary Figure 17. Radical-clock experiments

According to **general procedure D1**, to a flame-dried Schlenk tube were added *o*-bromostyrene (**1a**, 0.26 mmol, 1.3 equiv) and THF (0.6 mL) and the resulting solution was cooled to -78 °C. Subsequently, *n*-BuLi (0.16 mL, 1.6 M in hexane, 0.26 mmol, 1.3 equiv) was added dropwise and the resulting mixture was stirred at -78 °C for 1 h, then **2ah** (0.2mmol, 1.0 equiv, in 0.2 mL THF) was added dropwise to the solution of aryllithium reagent. And the resulting mixture was stirred at -78 °C for 40 mins, then allowed it to warm to ambient temperature and stirred for another 40 mins. Next, without removed the THF, the 4CzIPN (3.2 mg, 0.004 mmol, 2 mol%), HFIP (105 μL, 1.0 mmol, 5.0 equiv), MeCN (2 mL, 0.1M) were added to Schlenk tube under Ar, after which the Schlenk tube was sealed with parafilm. The reaction mixture was diluted with Et_2O and the solution washed with saturated aqueous NH_4Cl, water and brine. The combined organic layers were dried over Na_2SO_4 and concentrated under reduced pressure.

2.6.4 Validation experiment.

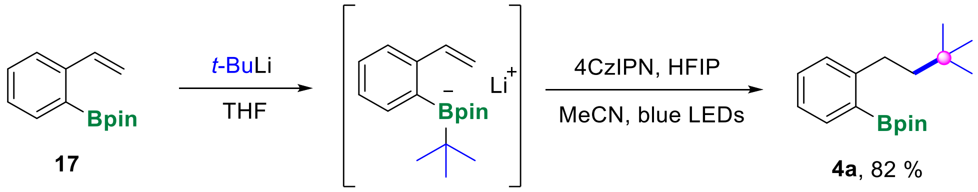

Supplementary Figure 18. Validation experiment

To a flame-dried Schlenk tube were added **17** (0.2 mmol, 1.0 equiv) and THF (1 mL) and the resulting solution was cooled to -78 °C. Subsequently, *t*-BuLi (0.26 mL, 1.0 M, 0.26 mmol, 1.3 equiv) was added dropwise and the resulting mixture was stirred at -78 °C for 1 h, then allowed it to warm to ambient temperature and stirred for another 40 mins. Next, without removing the THF, the 4CzIPN (3.2 mg, 0.004 mmol, 2 mol%), 1,1,1,3,3,3-hexafluoropropan-2-ol (HFIP, 105 μL, 1.0 mmol, 5.0 equiv), MeCN (2 mL, 0.1M) was added to Schlenk tube under Ar, after which the Schlenk tube was sealed with parafilm. The reaction mixture was diluted with EtOAC and the solution washed with saturated aqueous NH_4Cl, water and brine. The combined organic layers were dried over Na_2SO_4 and concentrated under reduced pressure. The crude product was then purified by flash column chromatography.

2-(2-(3,3-dimethylbutyl)phenyl)-4,4,5,5-tetramethyl-1,3,2-dioxaborolane (4a)

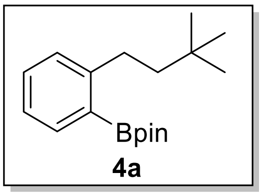

^{1}H NMR (500 MHz, $CDCl_3$) δ 7.78 (d, *J* = 7.6 Hz, 1H), 7.37 – 7.32 (m, 1H), 7.17 (t, *J* = 7.5 Hz, 2H), 2.89 – 2.80 (m, 2H), 1.47 – 1.41 (m, 2H), 1.35 (s, 12H), 0.98 (s, 9H).

^{13}C NMR (126 MHz, $CDCl_3$) δ 150.81 (s), 136.08 (s), 130.91 (s), 129.25 (s), 124.72 (s), 83.35 (s), 48.08 (s), 31.28 (s), 30.81 (s), 29.44 (s), 24.88 (s).

^{11}B NMR (128 MHz, $CDCl_3$) δ 31.42 (s).

HRMS (EI-QTOF) *m/z*: $[M]^+$ Calcd. for $C_{18}H_{29}BO_2$ 288.2261; Found 288.2266.

2.6.5 Cross-over experiment.

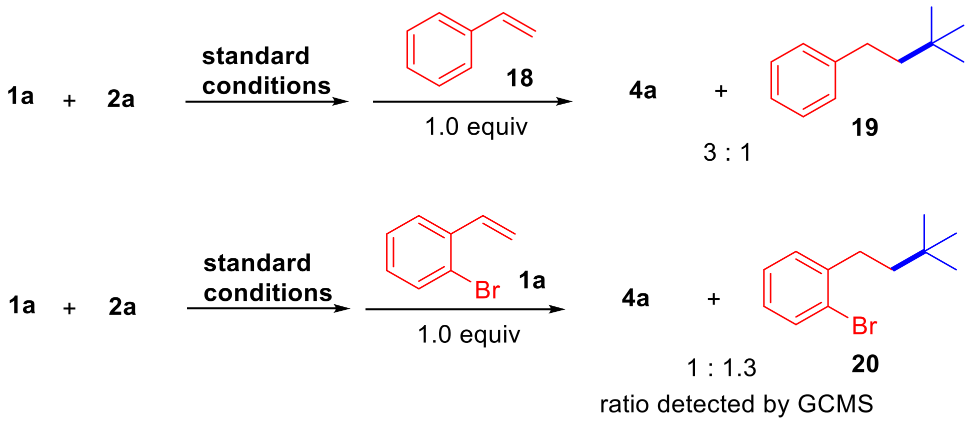

Supplementary Figure 19. Cross-over experiment

According to **general procedure D_1**, to a flame-dried Schlenk tube were added *o*-bromostyrene (**1a**, 32.5 μL, 0.26 mmol, 1.3 equiv) and THF (0.6 mL) and the resulting solution was cooled to -78 °C. Subsequently, *n*-BuLi (0.16 mL, 1.6 M in hexane, 0.26 mmol, 1.3 equiv) was added dropwise and the resulting mixture was stirred at -78 °C for 1 h, before **2a** (0.2mmol, 1.0 equiv, in 0.2 mL THF) was added dropwise to the solution of aryllithium reagent. And the resulting mixture was stirred at -78 °C for 40 mins, then allowed it to warm to ambient temperature and stirred for another 40 mins. Next, without removed the THF, the 4CzIPN (3.2 mg, 0.004 mmol, 2 mol%), HFIP (105 μL, 1.0 mmol, 5.0 equiv), **1a** or **18** (0.2 mmol, 1.0 equiv), MeCN (2 mL, 0.1M) was

added to Schlenk tube under Ar, after which the Schlenk tube was sealed with parafilm. The resulting ration was detected by GC-MS.

2.7 Cyclic voltammetry measurement

Cyclic voltammetry (CV) experiments were conducted in a 10 mL glass vial fitted with a glassy carbon working electrode (3 mm in diameter), a platinum wire auxiliary electrode and submerged in saturated aqueous KCl solution Ag/AgCl reference electrode.

MeCN (5 mL) containing 0.02 mmol $^{n}Bu_4NBF_4$ and 0.05 mmol complex **3**, which was pre-prepared, were poured into the electrochemical cell. The oxidation potential of complex **3** was 1.09 V versus Ag/AgCl in MeCN.

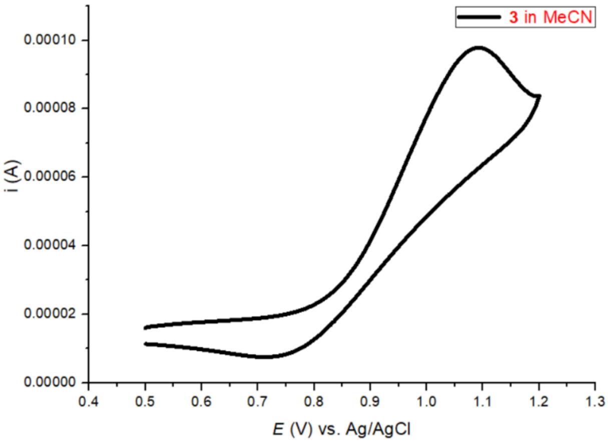

Supplementary Figure 20. Cyclic voltammetry measurement

2.8 Luminescence quenching experiment

Steady-state emission spectra were acquired using an Edinburgh Instruments, FLS920 spectrometer. In a typical experiment, the emission spectrum of a 1×10^{-4} M solution of in THF was collected. Photocatalyst 4CzIPN (0.01 mmol, 7.9 mg) was dissolved in THF (100 mL) to set the concentration is $1*10^{-4}$ M. *t*-BuBpin (0.1 mmol) and (2-vinylphenyl)lithium (0.13 mmol), which was pre-prepared, were dissolved in 2 mL THF and stirred for 1 h at -78 °C, warmed to room temperature for 40 mins and diluted to 25 mL to set the concentration is $4*10^{-3}$ M. The Schlenk tube was charged with 2 mL 4CzIPN ($1*10^{-4}$ M). 1000 µL, 700 µL, 500 µL, 300 µL, 200 µL, 100 µL, 50 µL, 0 µL **3**

Complex were added and the fluorescence spectra were collected, respectively.

The relative intensity I_0/I was calculated as a function of quencher concentration, where I_0 is the luminescence intensity in the absence of quencher, while I is the intensity in the presence of the quencher.

*4CzIPN quenched by Complex **3*** *Stern−Volmer plot*

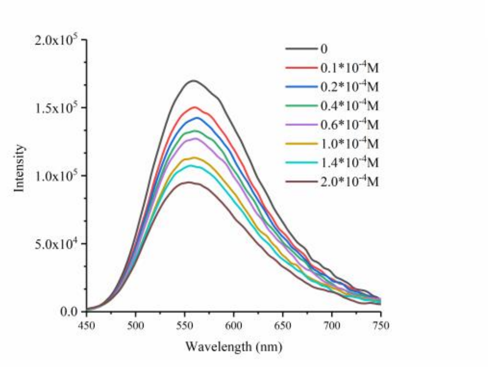

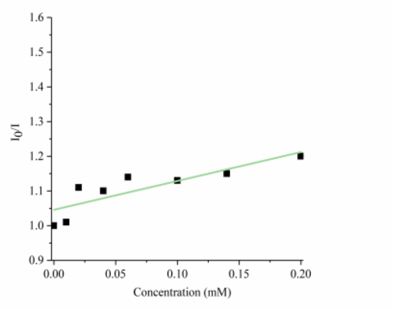

Supplementary Figure 21. Luminescence quenching experiment

2.9 NMR Spectroscopic Data

4,4,5,5-tetramethyl-2-(2-methyl-4-phenylbutan-2-yl)-1,3,2-dioxaborolane (2i)

^{1}H spectrum (500 MHz, room temperature, $CDCl_3$) of (**2i**)

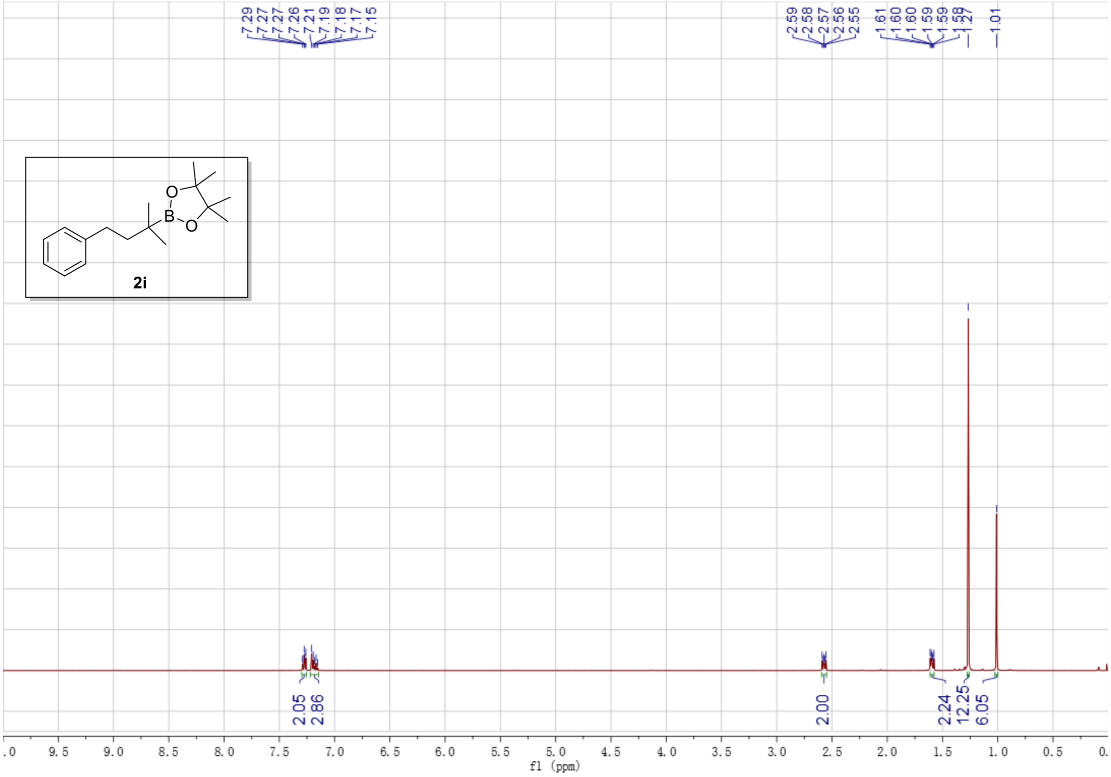

Supplementary Figure 22. ^{1}H NMR spectrum of **2i.**

^{13}C spectrum (126 MHz, room temperature, $CDCl_3$) of (**2i**)

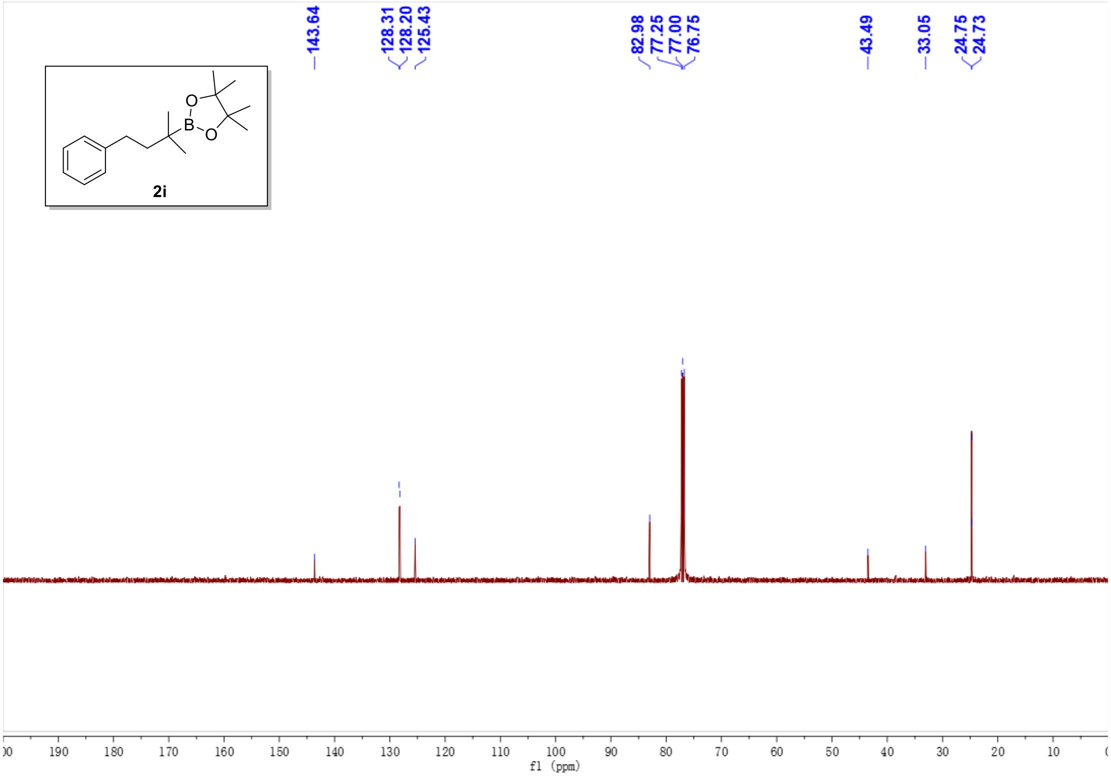

Supplementary Figure 23. ^{13}C NMR spectrum of **2i**.

^{11}B spectrum (128 MHz, room temperature, $CDCl_3$) of (**2i**)

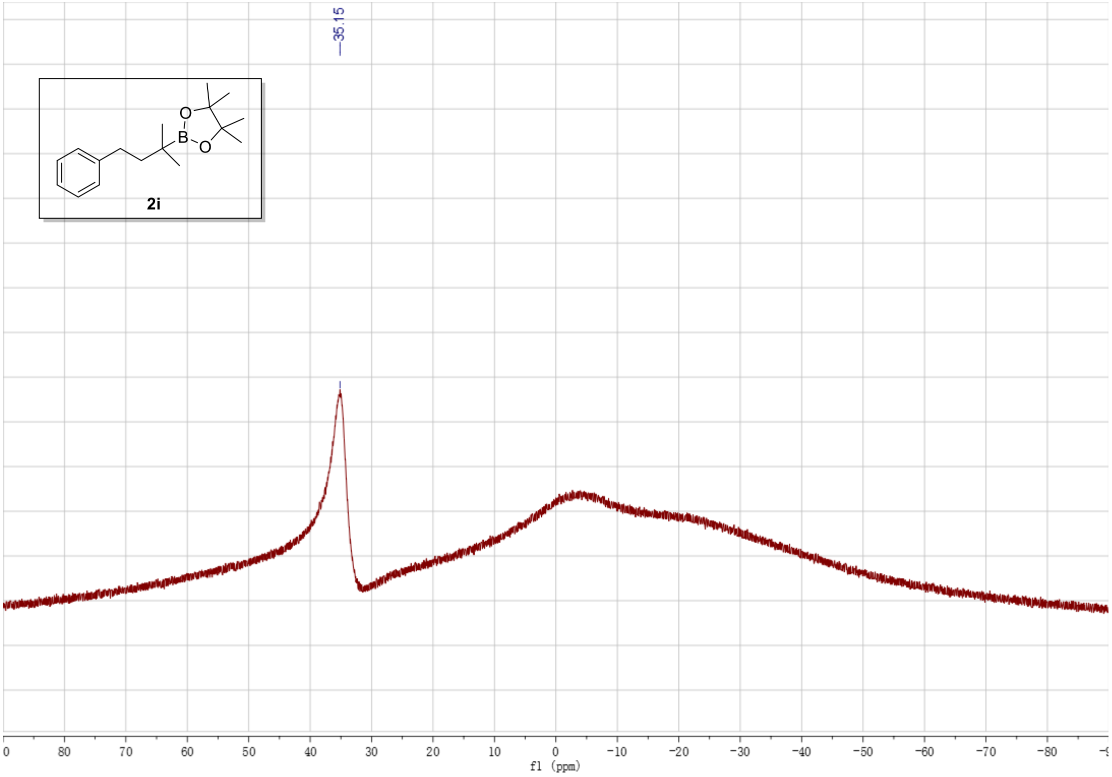

Supplementary Figure 24. ^{11}B spectrum of **2i**.

4,4,5,5-tetramethyl-2-((2R)-2,6,6-trimethylbicyclo[3.1.1]heptan-2-yl)-1,3,2-dioxaborolane (2ah)

^{1}H spectrum (500MHz, room temperature, $CDCl_3$) of (**2ah**)

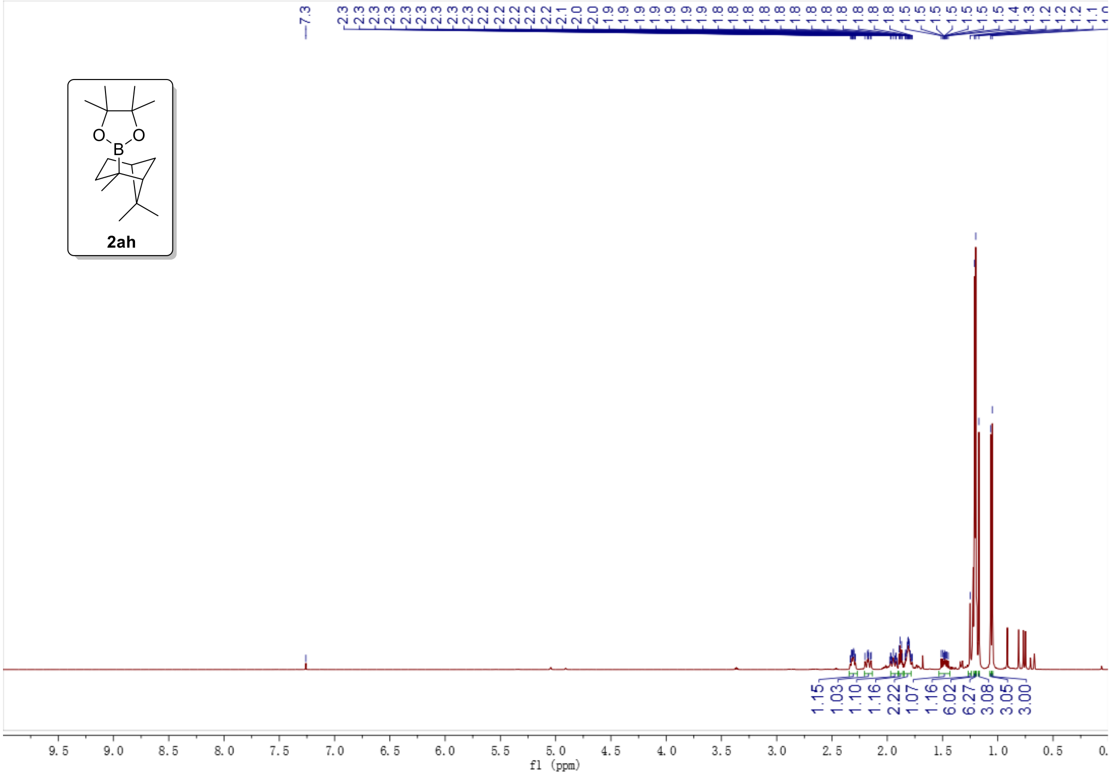

Supplementary Figure 25. ^{1}H NMR spectrum of **2ah**.

^{13}C spectrum (101 MHz, room temperature, $CDCl_3$) of (**2ah**)

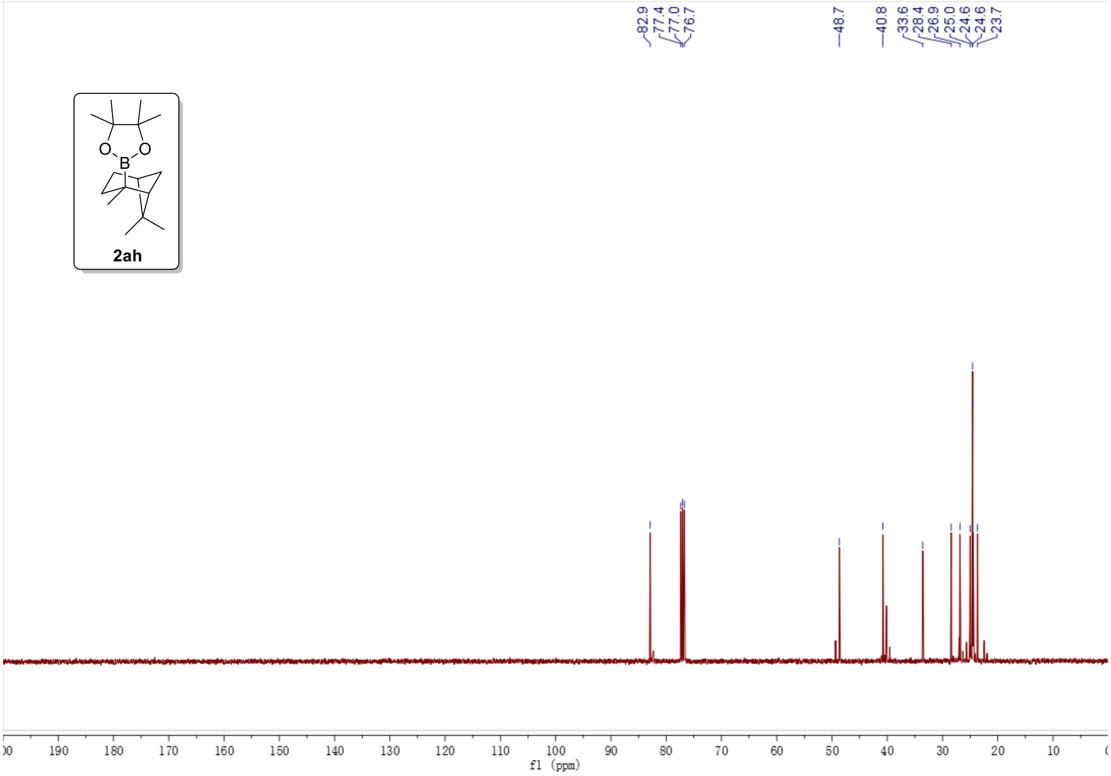

Supplementary Figure 26. ^{13}C NMR spectrum of **2ah**.

^{11}B spectrum (128 MHz, room temperature, $CDCl_3$) of (**2ah**)

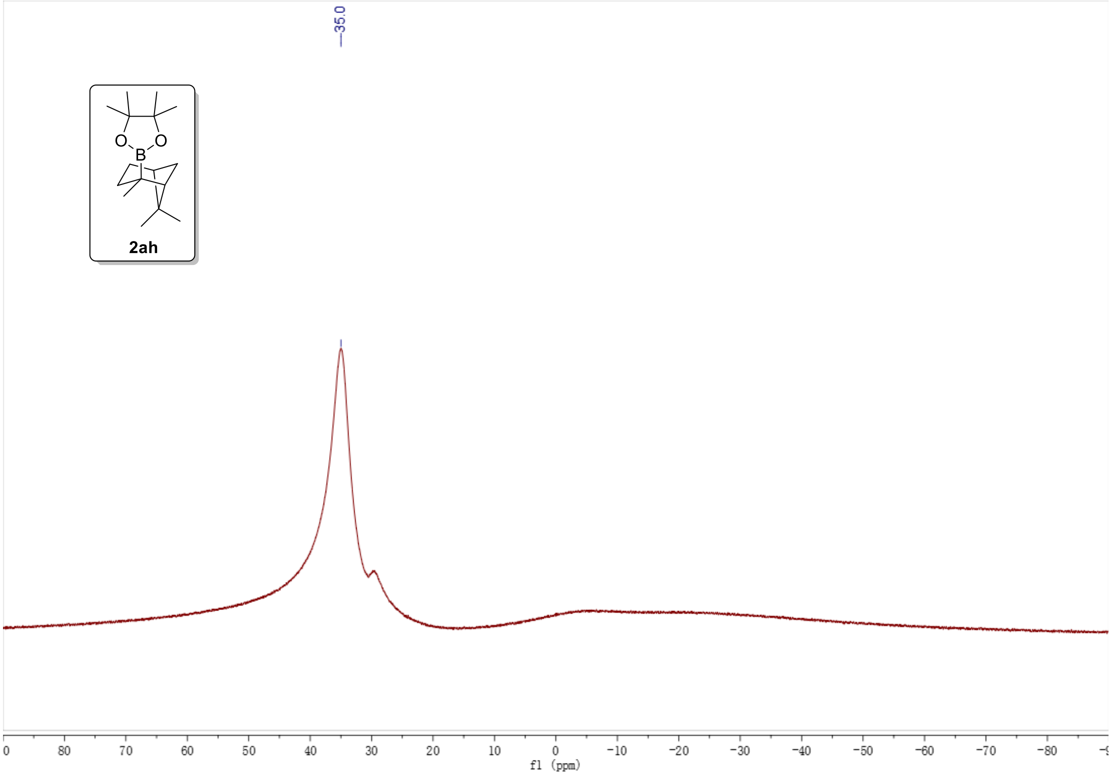

Supplementary Figure 27. ^{11}B spectrum of **2ah.**

2-(1-(benzo[d][1,3]dioxol-5-yl)-2-methylpropan-2-yl)-4,4,5,5-tetramethyl-1,3,2-dioxaborolane (2aj)

^{1}H spectrum (500 MHz, room temperature, $CDCl_3$) of (**2aj**)

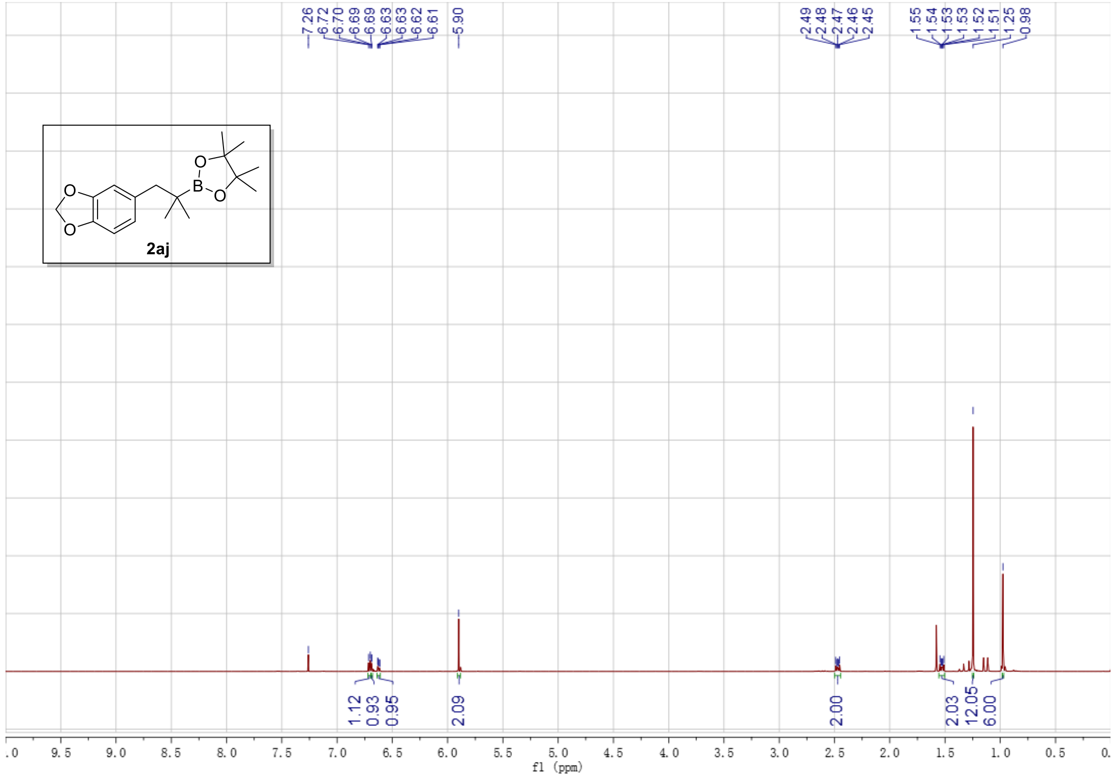

Supplementary Figure 28. ^{1}H NMR spectrum of **2aj**.

^{13}C spectrum (126 MHz, room temperature, $CDCl_3$) of (**2aj**)

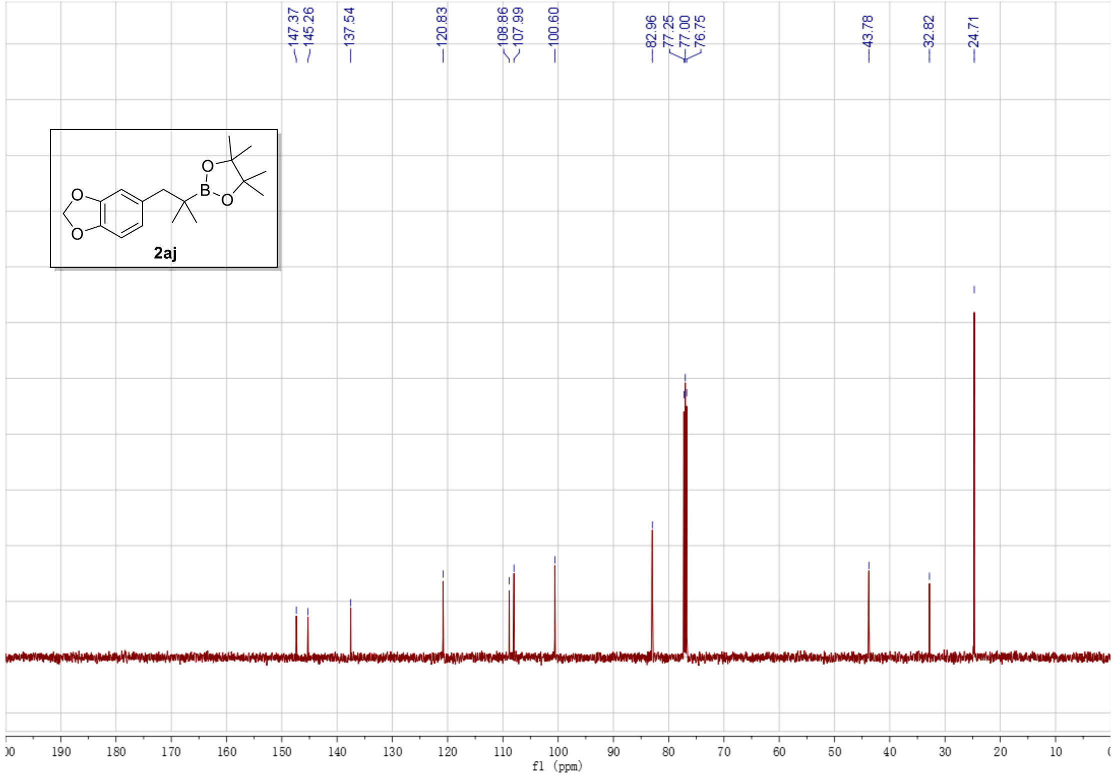

Supplementary Figure 29. ^{13}C NMR spectrum of **2aj**.

^{11}B spectrum (128 MHz, room temperature, $CDCl_3$) of (**2aj**)

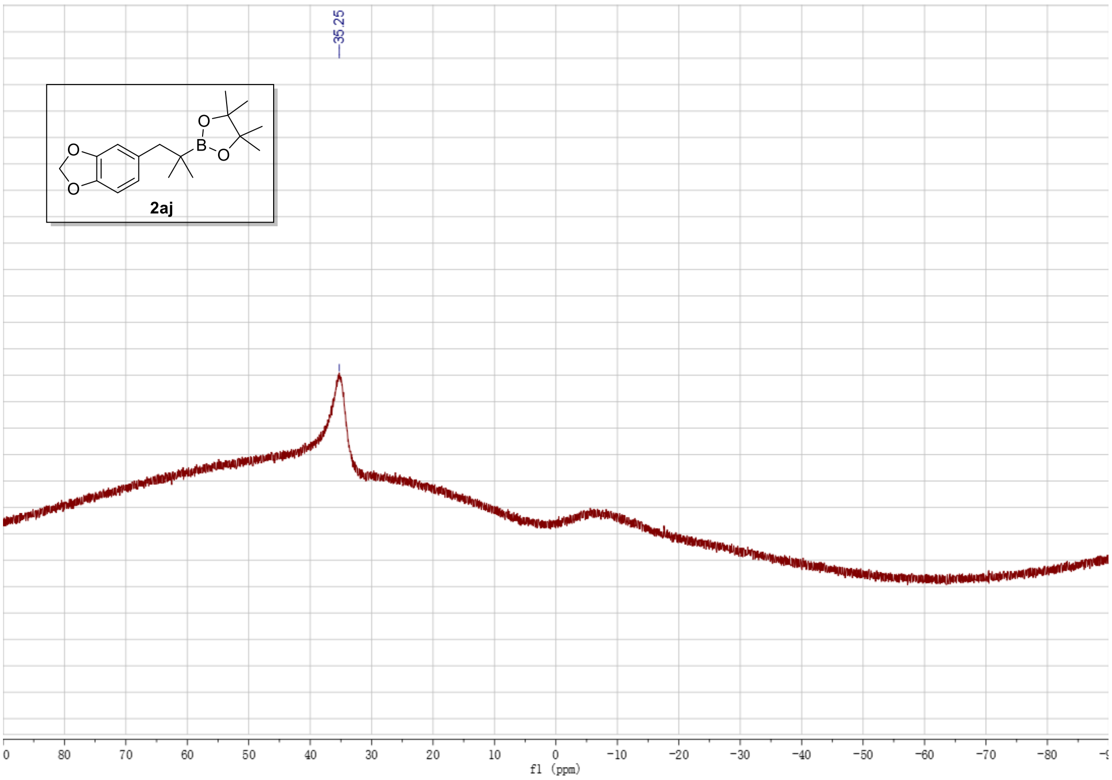

Supplementary Figure 30. ^{11}B spectrum of **2aj**.

2-(3-(4-isobutylphenyl)-2-methylbutan-2-yl)-4,4,5,5-tetramethyl-1,3,2-dioxaborolane (2ak)

^{1}H spectrum (500 MHz, room temperature, $CDCl_3$) of (**2ak**)

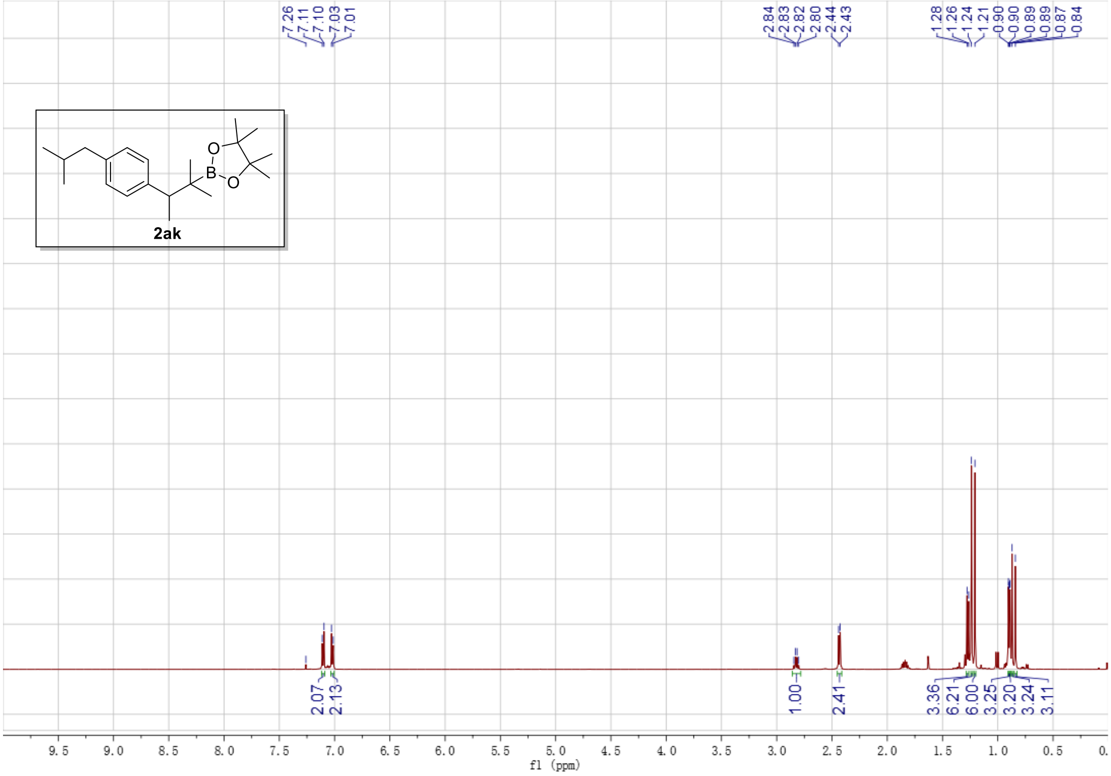

Supplementary Figure 31. ^{1}H NMR spectrum of **2ak**.

^{13}C spectrum (126 MHz, room temperature, $CDCl_3$) of (**2ak**)

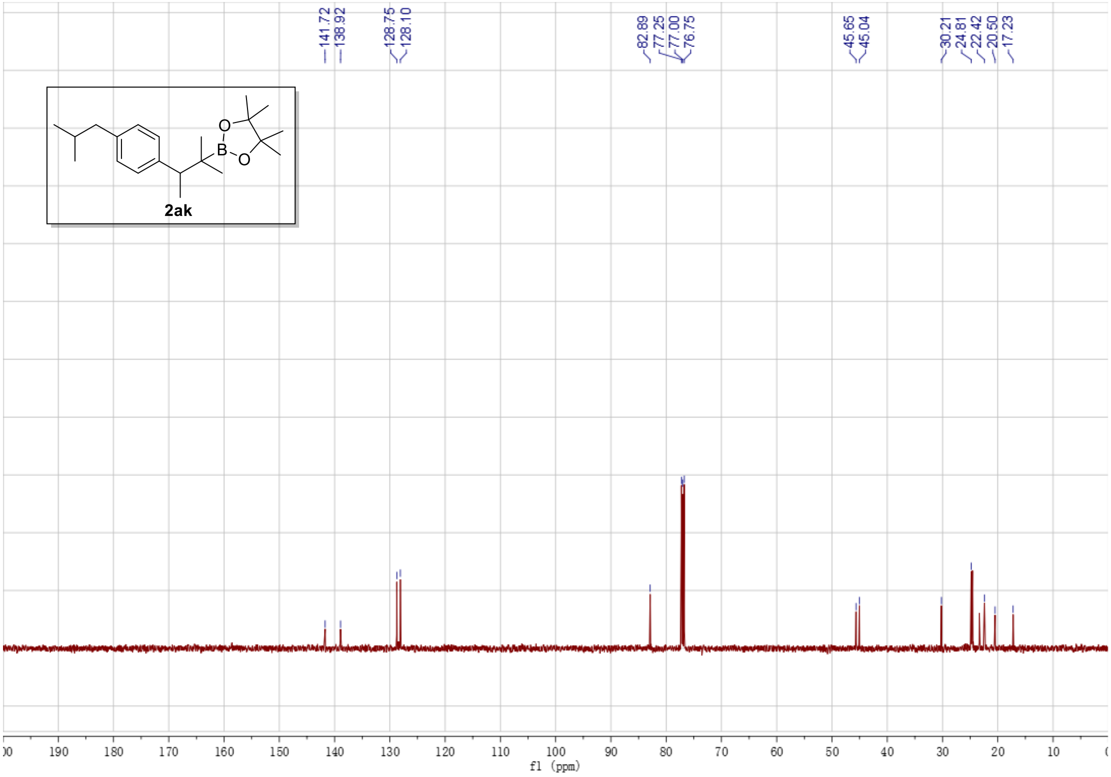

Supplementary Figure 32. ^{13}C NMR spectrum of **2ak**.

^{11}B spectrum (128 MHz, room temperature, $CDCl_3$) of (**2ak**)

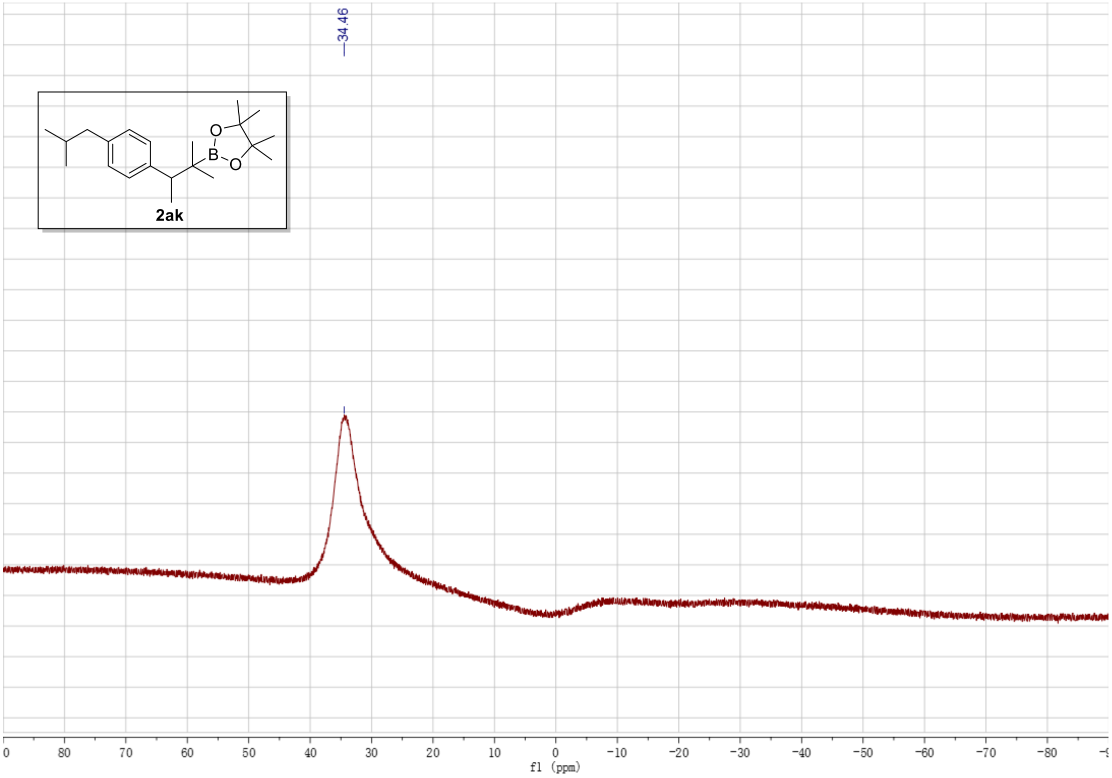

Supplementary Figure 33. ^{11}B spectrum of **2ak**.

2,2'-(2-ethylbutane-1,2-diyl)bis(4,4,5,5-tetramethyl-1,3,2-dioxaborolane) (5a)

^{1}H spectrum (500 MHz, room temperature, $CDCl_3$) of (**5a**)

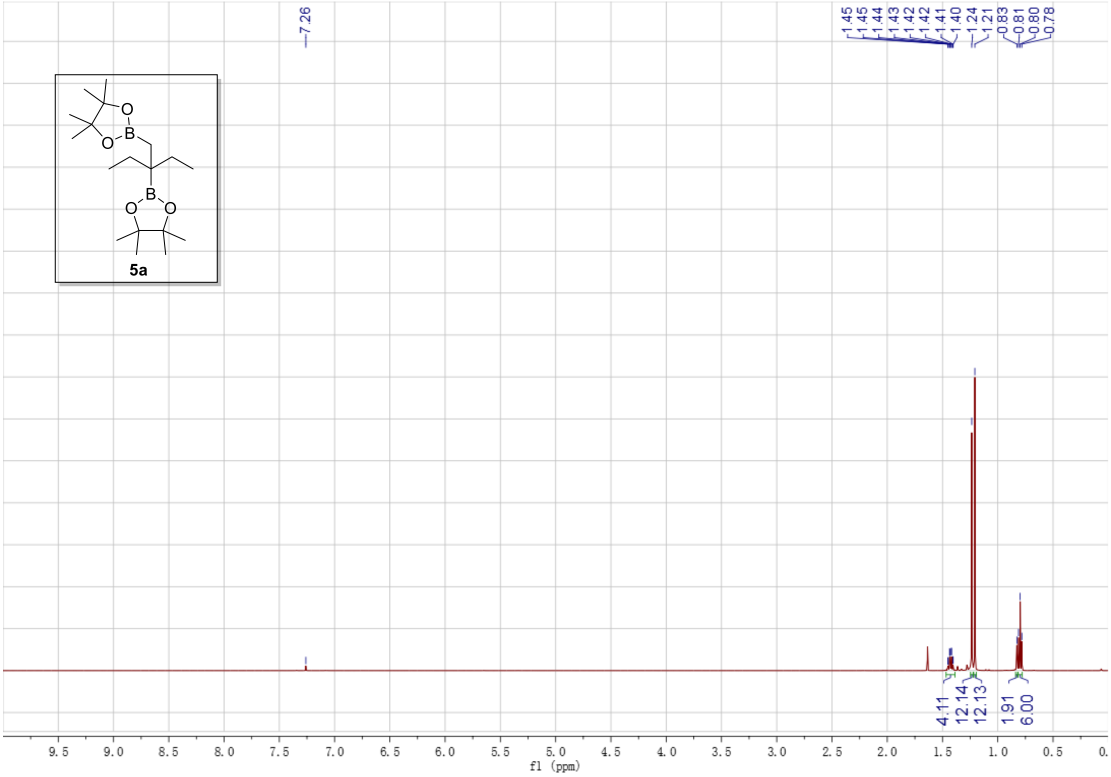

Supplementary Figure 34. ^{1}H NMR spectrum of **5a**.

^{13}C spectrum (126 MHz, room temperature, $CDCl_3$) of (**5a**)

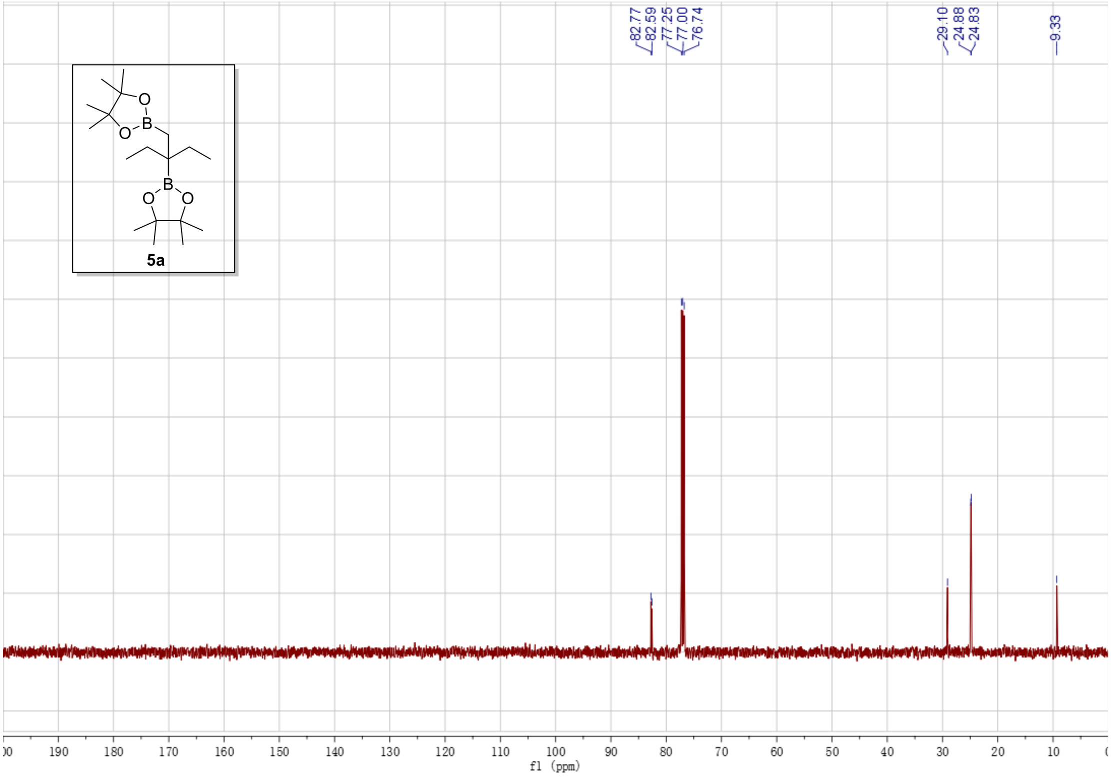

Supplementary Figure 35. ^{13}C NMR spectrum of **5a**.

^{11}B spectrum (128 MHz, room temperature, $CDCl_3$) of (**5a**)

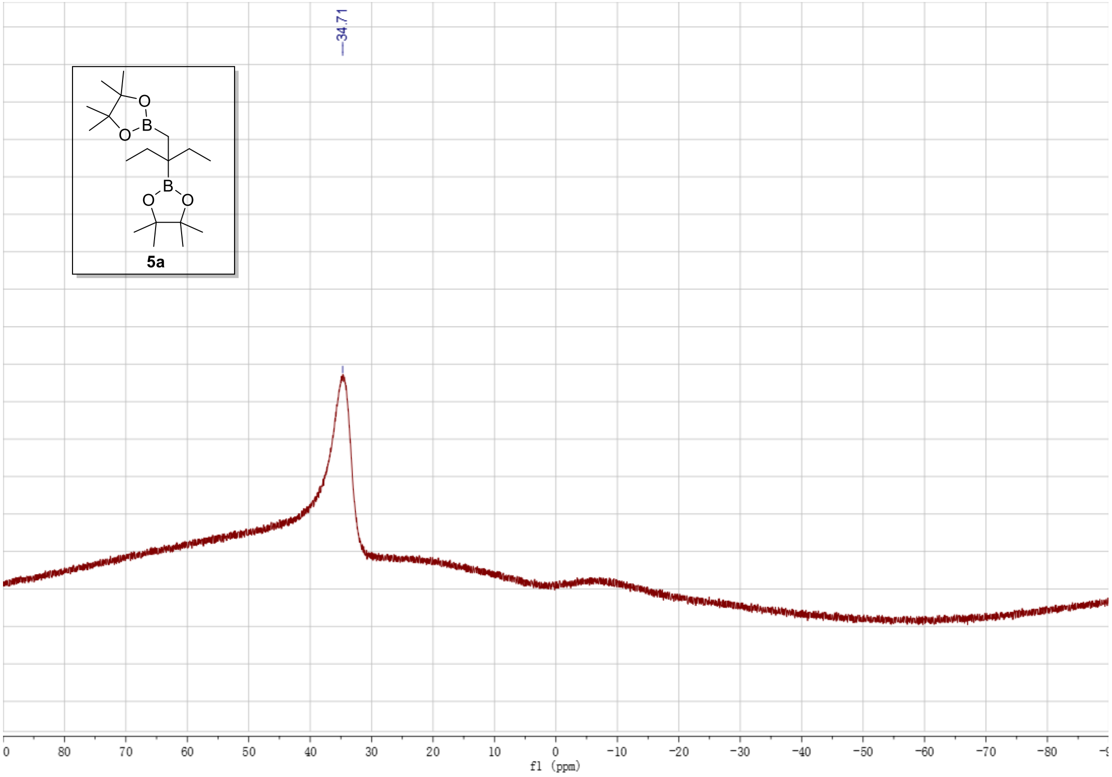

Supplementary Figure 36. ^{11}B spectrum of **5a**.

2,2'-(2-methylpentane-1,2-diyl)bis(4,4,5,5-tetramethyl-1,3,2-dioxaborolane) (5c)

^{1}H spectrum (400 MHz, room temperature, $CDCl_3$) of (**5c**)

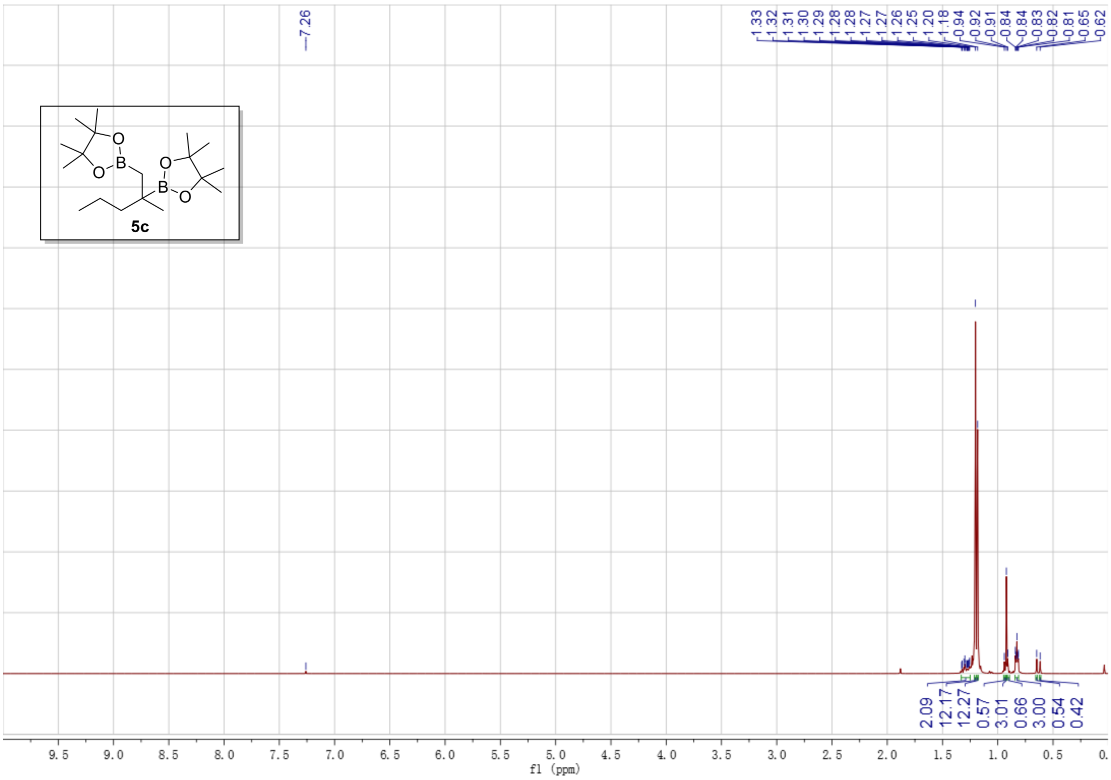

Supplementary Figure 37. ^{1}H NMR spectrum of **5c**.

^{13}C spectrum (126 MHz, room temperature, $CDCl_3$) of (**5c**)

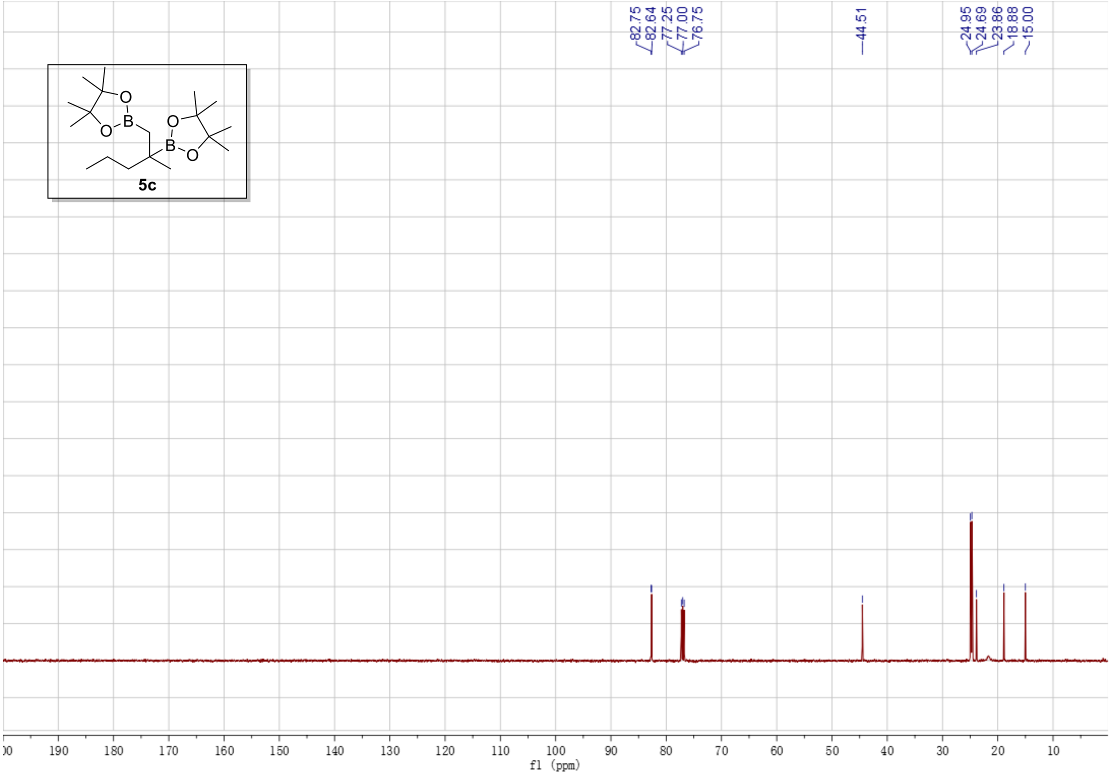

Supplementary Figure 38. ^{13}C NMR spectrum of **5c**.

^{11}B spectrum (160 MHz, room temperature, $CDCl_3$) of (**5c**)

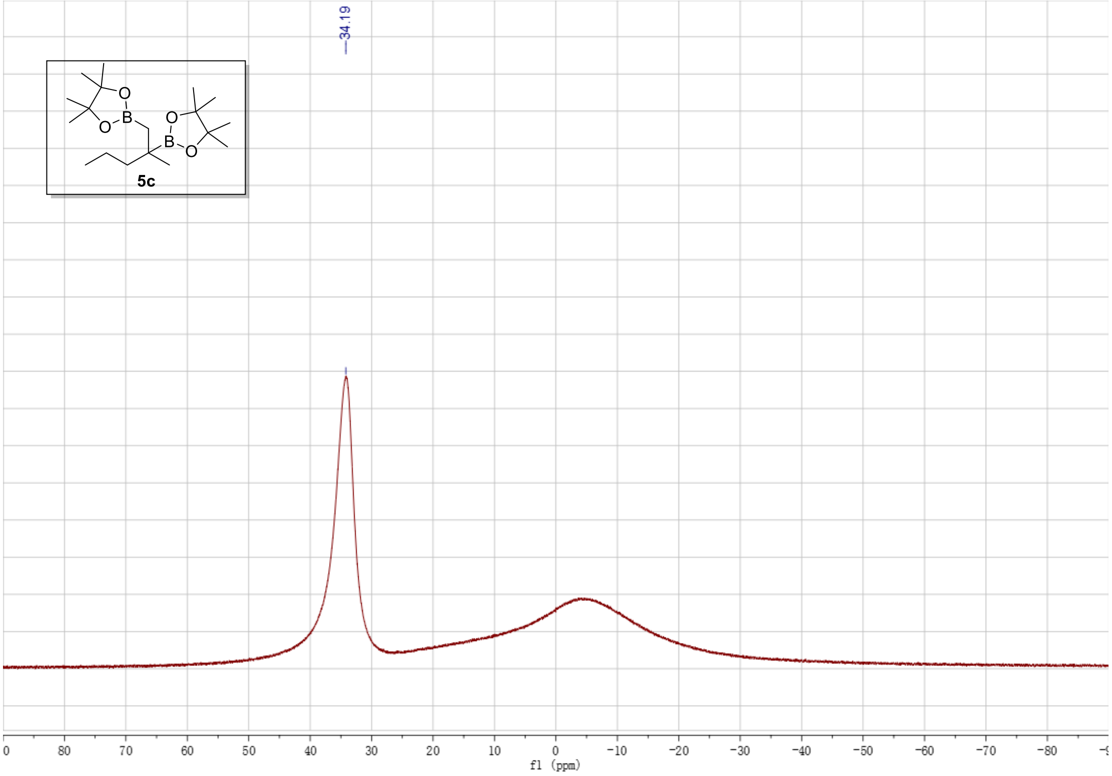

Supplementary Figure 39. ^{11}B spectrum of **5c**.

2,2'-(2-methylhexane-1,2-diyl)bis(4,4,5,5-tetramethyl-1,3,2-dioxaborolane) (5d)

^{1}H spectrum (500 MHz, room temperature, $CDCl_3$) of (**5d**)

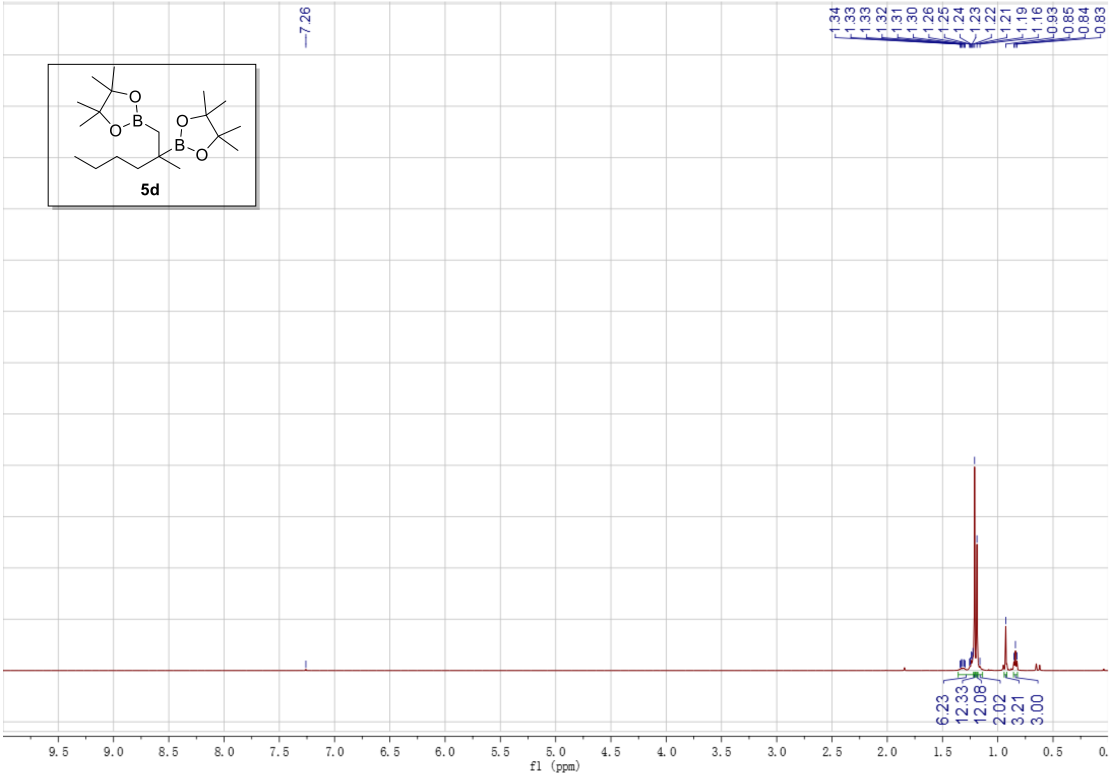

Supplementary Figure 40. ^{1}H NMR spectrum of **5d**.

^{13}C spectrum (126 MHz, room temperature, $CDCl_3$) of (**5d**)

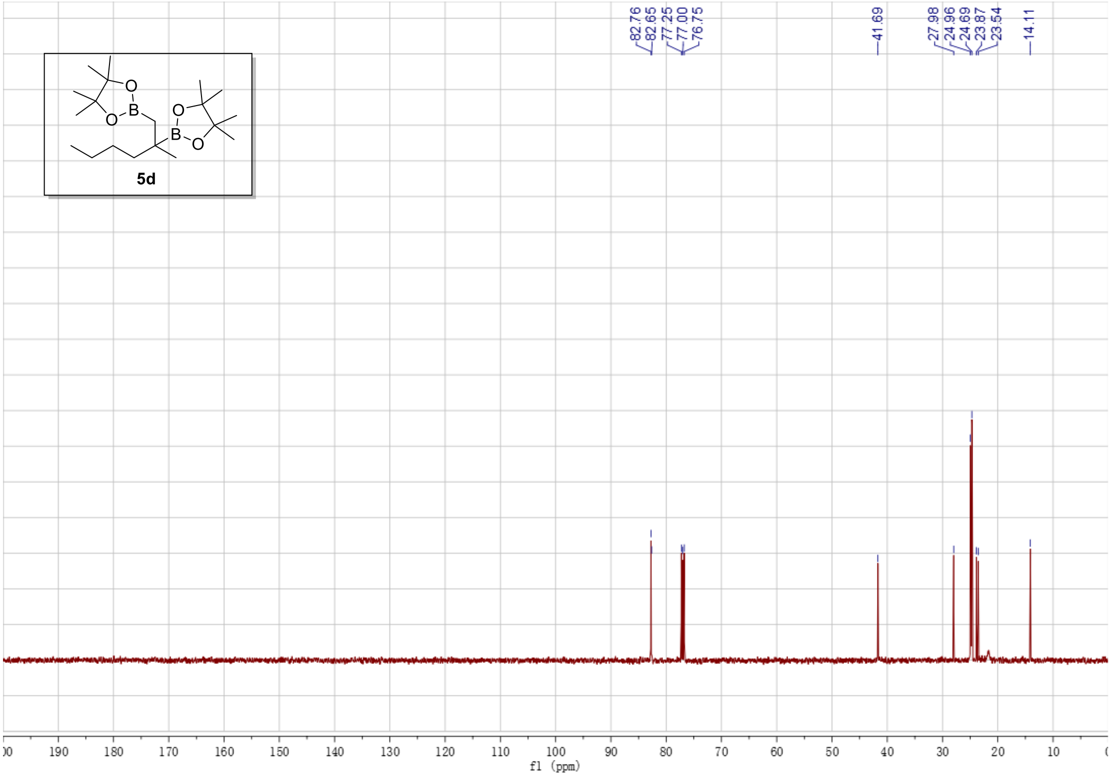

Supplementary Figure 41. ^{13}C NMR spectrum of **5d**.

^{11}B spectrum (128 MHz, room temperature, $CDCl_3$) of (**5d**)

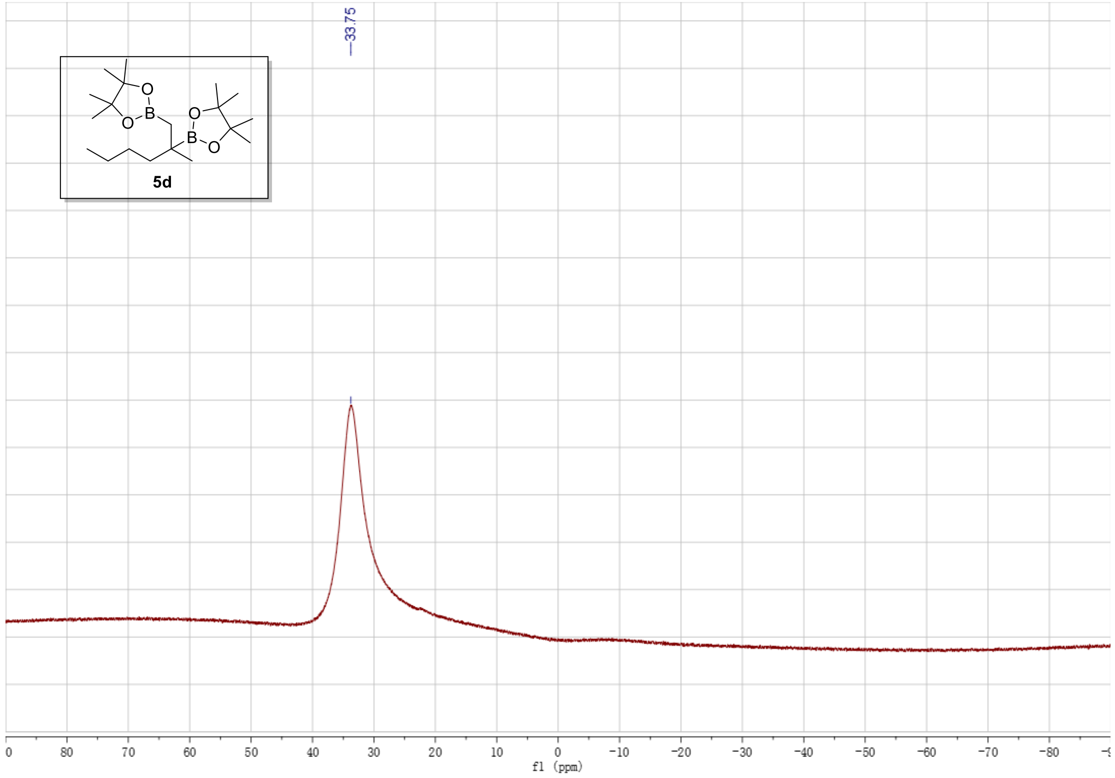

Supplementary Figure 42. ^{11}B spectrum of **5d**.

2,2'-(2-cyclohexylpropane-1,2-diyl)bis(4,4,5,5-tetramethyl-1,3,2-dioxaborolane) (5e)

^{1}H spectrum (500 MHz, room temperature, $CDCl_3$) of **(5e)**

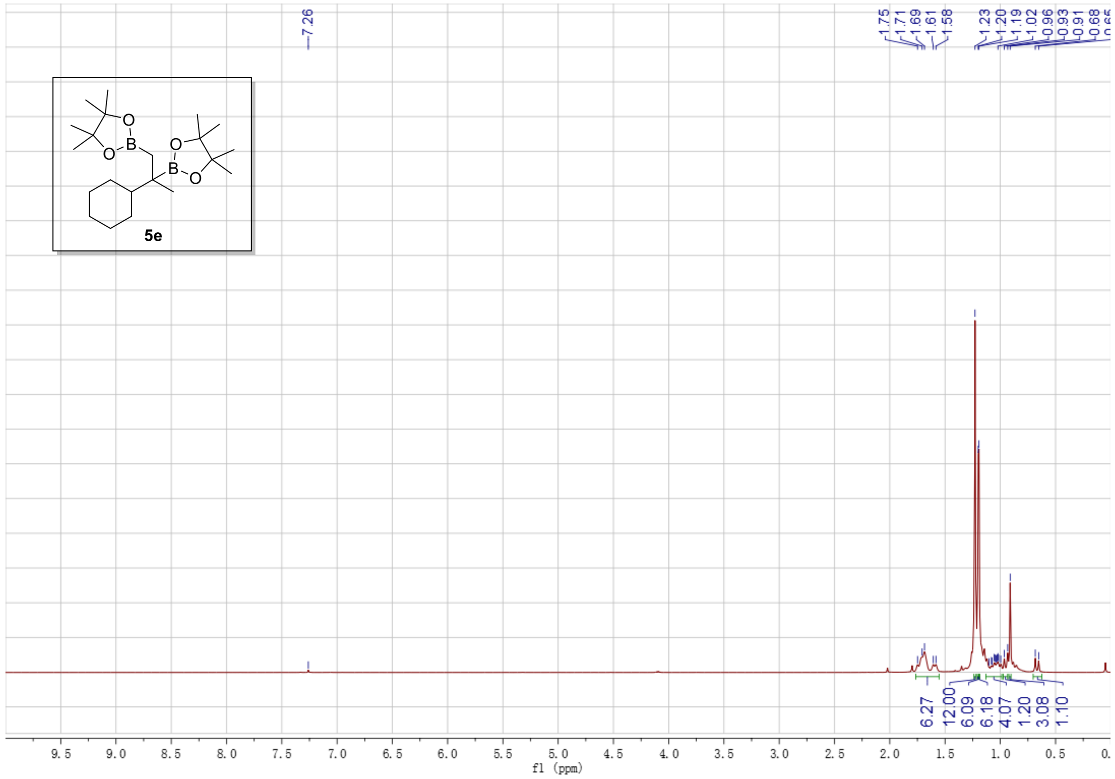

Supplementary Figure 43. ^{1}H NMR spectrum of **5e**.

^{13}C spectrum (126 MHz, room temperature, $CDCl_3$) of (**5e**)

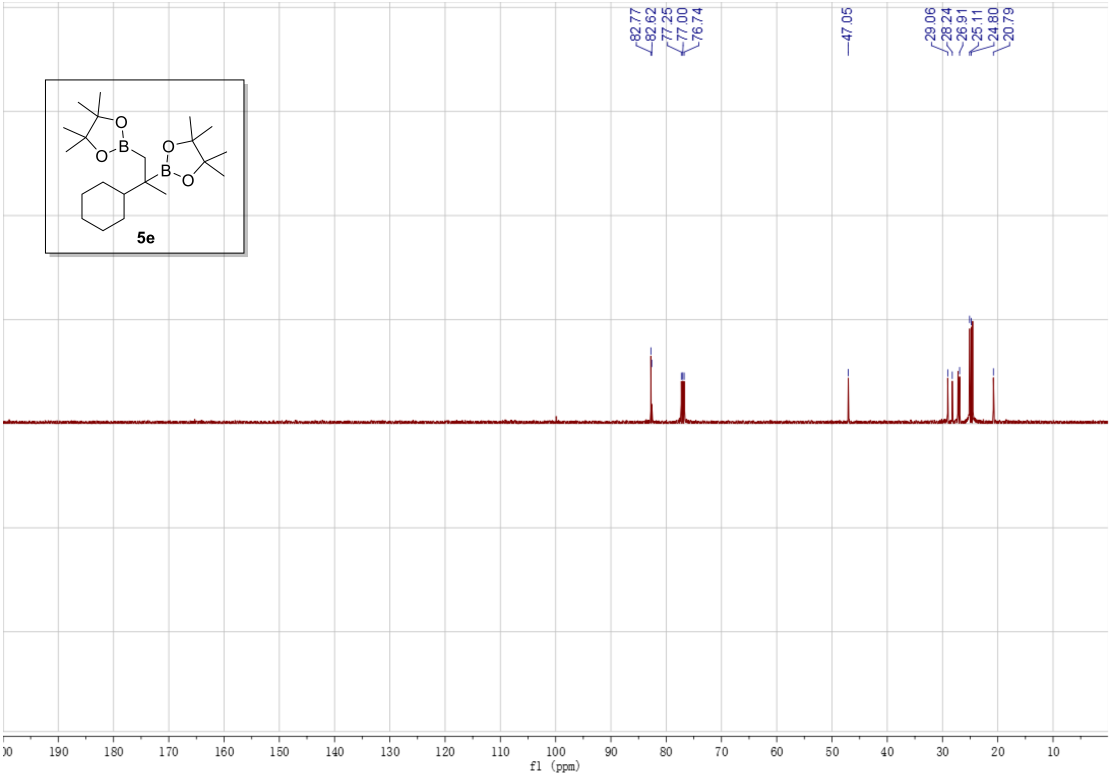

Supplementary Figure 44. ^{13}C NMR spectrum of **5e**.

^{11}B spectrum (160 MHz, room temperature, $CDCl_3$) of (**5e**)

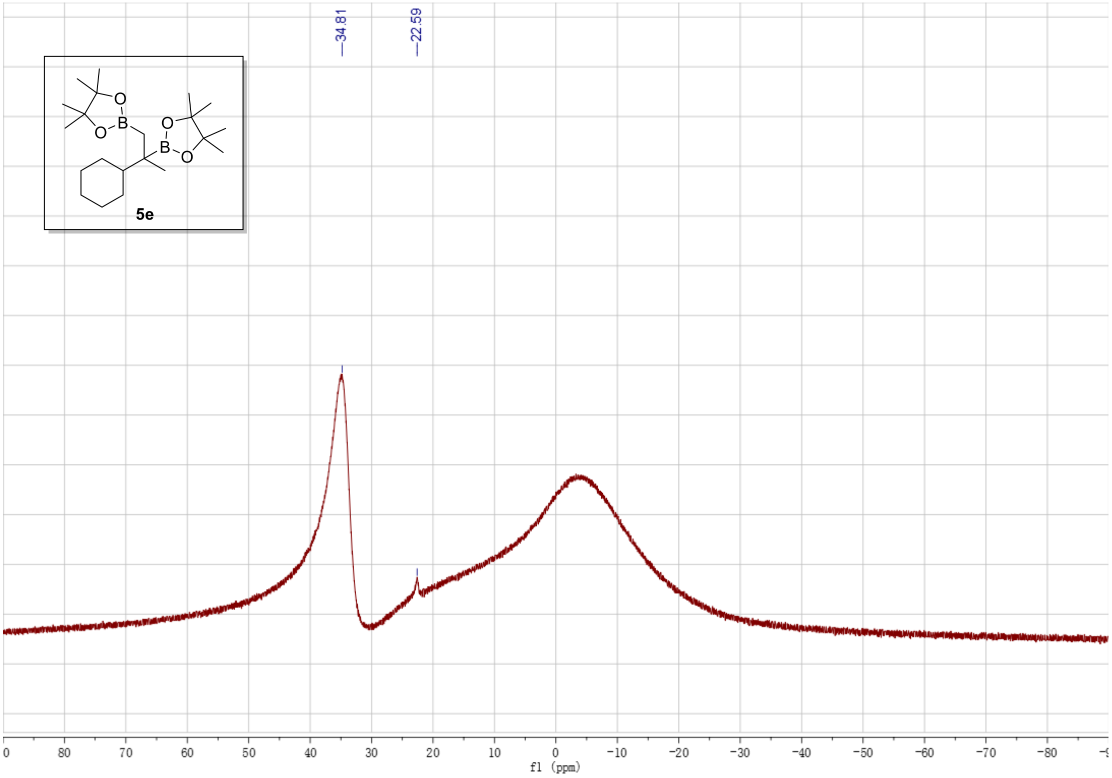

Supplementary Figure 45. ^{11}B spectrum of **5e**.

2,2'-(2-methyl-4-phenylbutane-1,2-diyl)bis(4,4,5,5-tetramethyl-1,3,2-dioxaborolane) (5f)

^{1}H spectrum (500 MHz, room temperature, $CDCl_3$) of (**5f**)

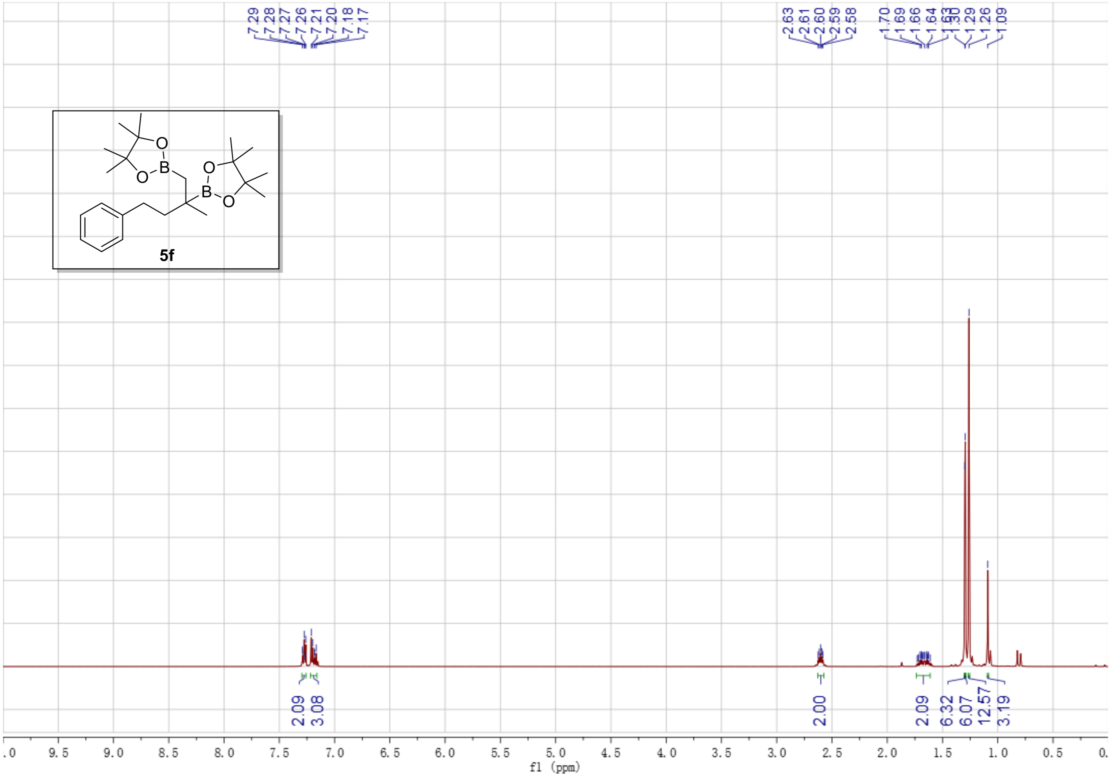

Supplementary Figure 46. ^{1}H NMR spectrum of **5f**.

^{13}C spectrum (126 MHz, room temperature, $CDCl_3$) of (**5f**)

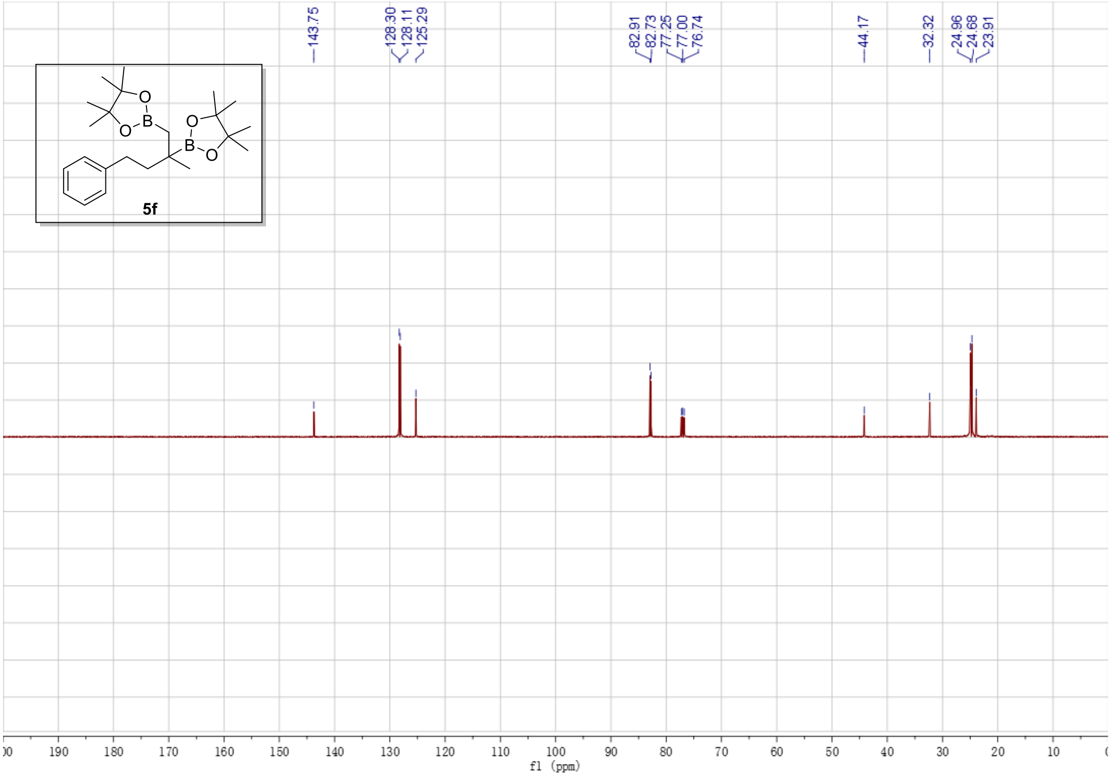

Supplementary Figure 47. ^{13}C NMR spectrum of **5f**.

^{11}B spectrum (160 MHz, room temperature, $CDCl_3$) of (**5f**)

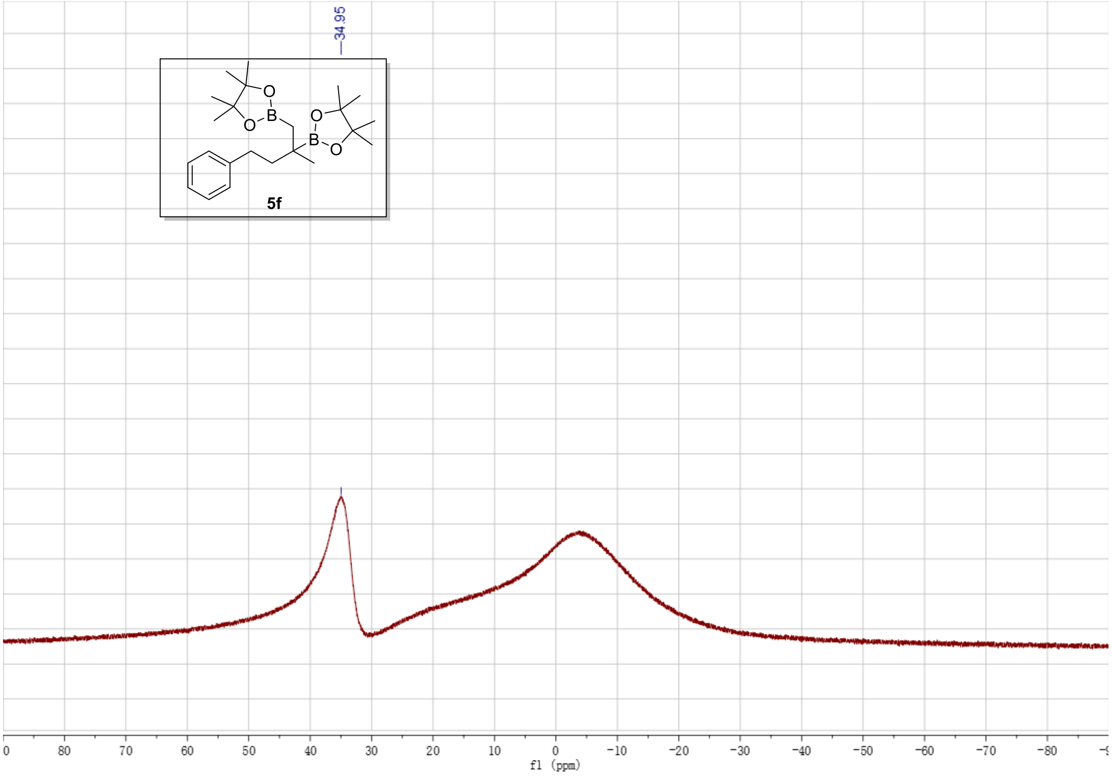

Supplementary Figure 48. ^{11}B spectrum of **5f**.

2,2'-(2,3,3-trimethylbutane-1,2-diyl)bis(4,4,5,5-tetramethyl-1,3,2-dioxaborolane) (5g)

^{1}H spectrum (500 MHz, room temperature, $CDCl_3$) of (**5g**)

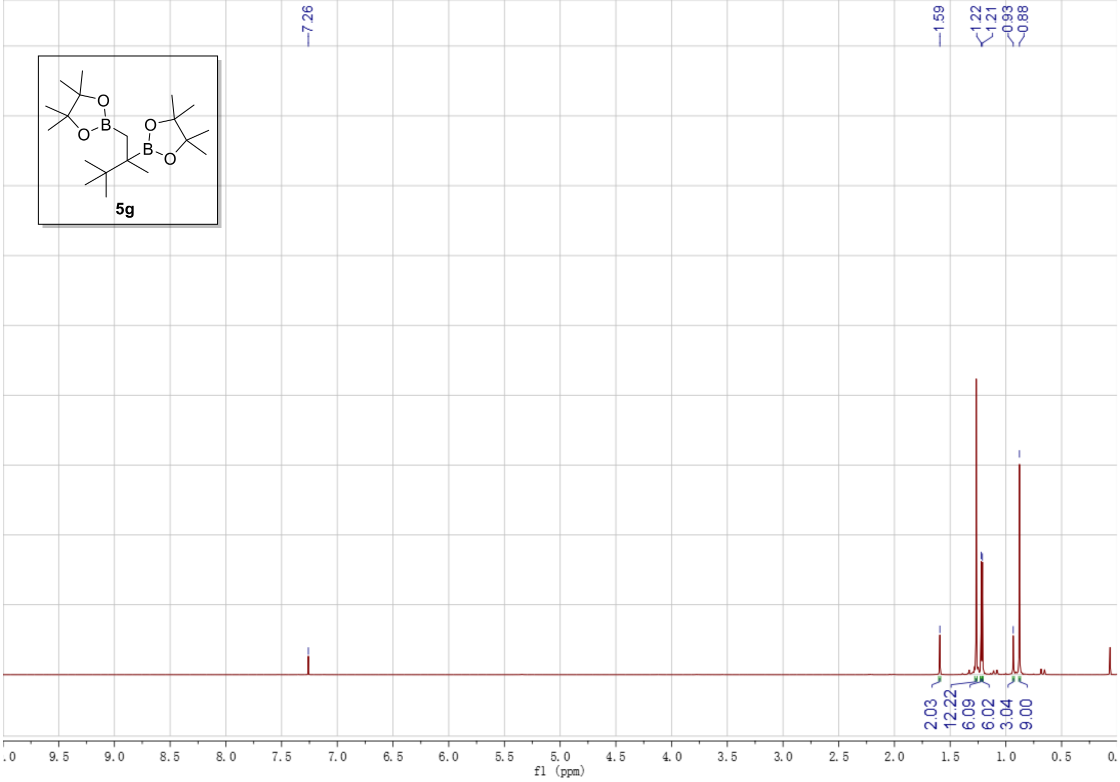

Supplementary Figure 49. ^{1}H NMR spectrum of **5g**.

^{13}C spectrum (126 MHz, room temperature, $CDCl_3$) of (**5g**)

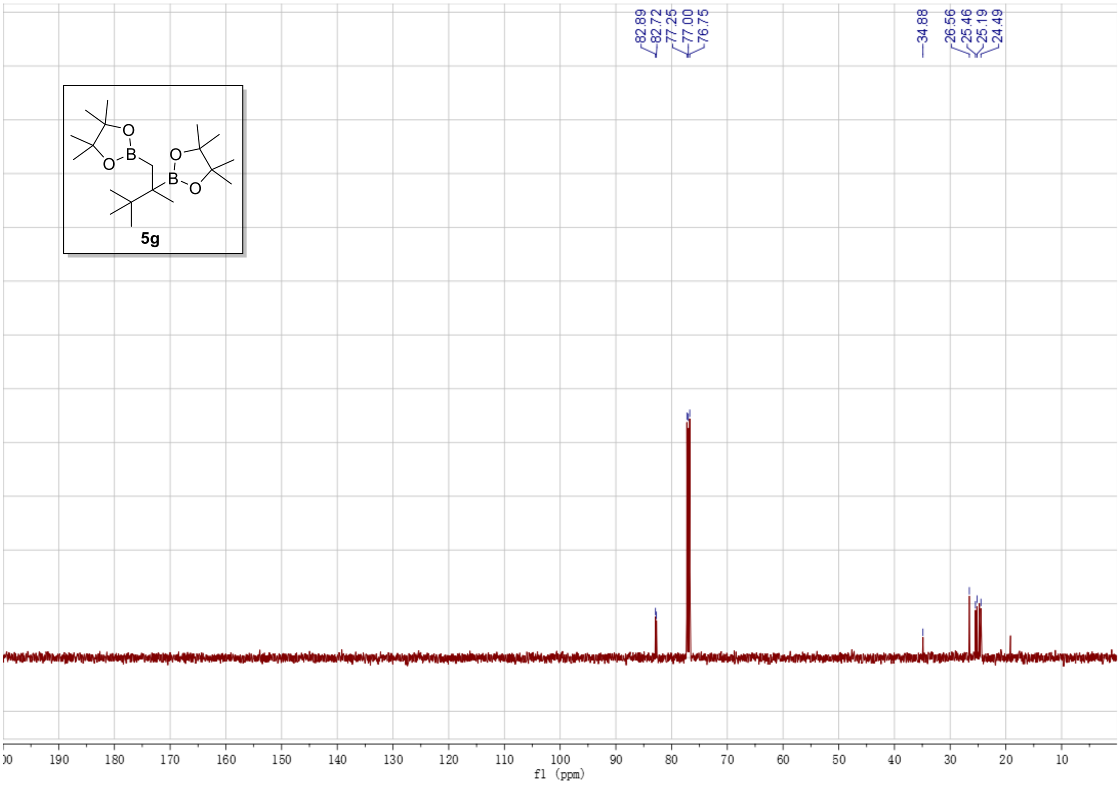

Supplementary Figure 50. ^{13}C NMR spectrum of **5g**.

^{11}B spectrum (128 MHz, room temperature, $CDCl_3$) of (**5g**)

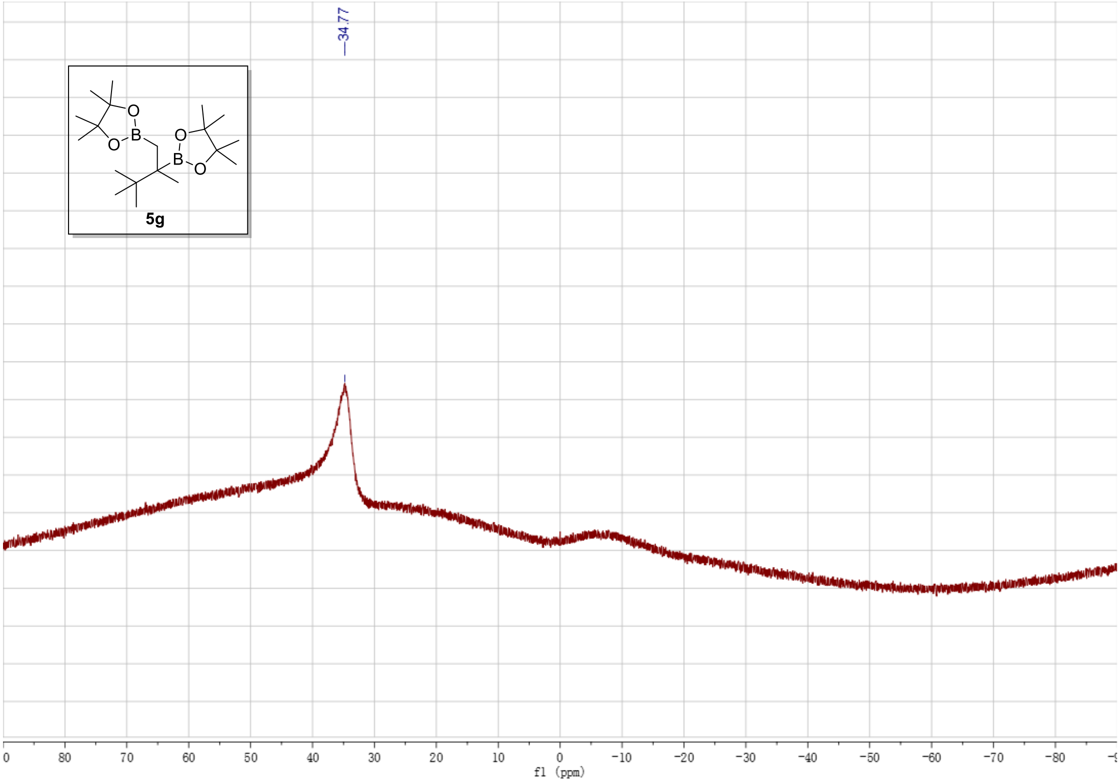

Supplementary Figure 51. ^{11}B spectrum of **5g**.

2,2'-(4-phenylbutane-2,2-diyl)bis(4,4,5,5-tetramethyl-1,3,2-dioxaborolane) (5'f)

^{1}H spectrum (400 MHz, room temperature, $CDCl_3$) of (**5'f**)

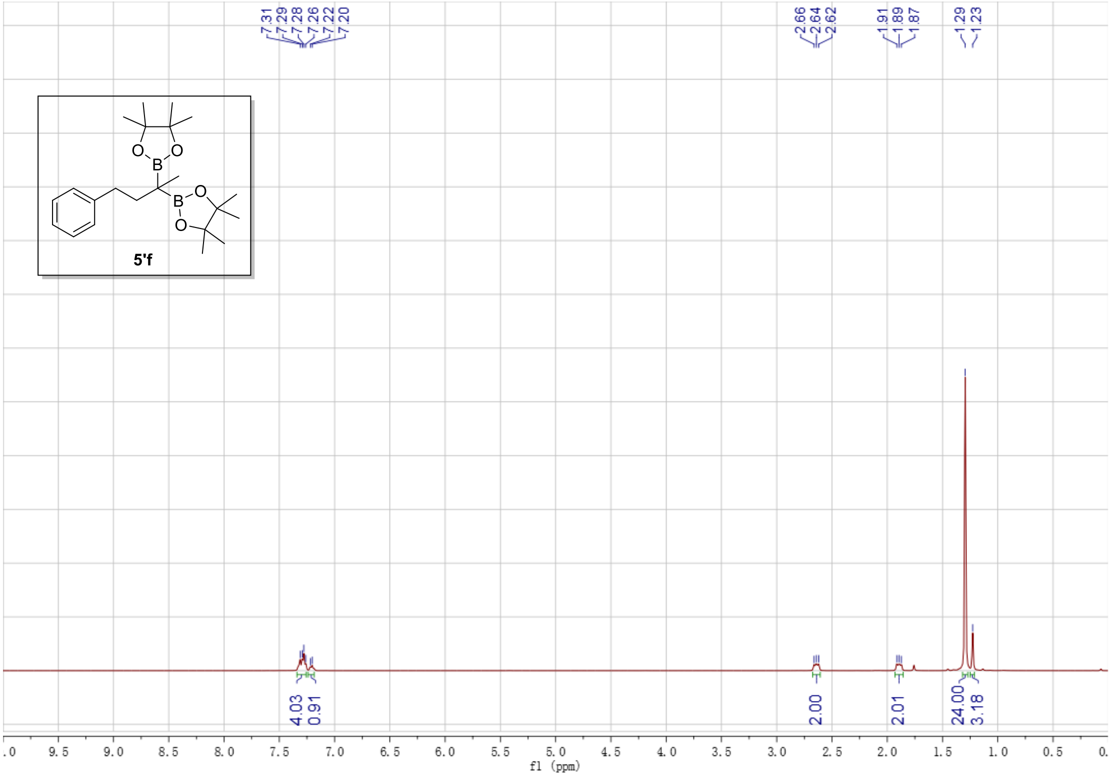

Supplementary Figure 52. ^{1}H NMR spectrum of **5'f**.

^{13}C spectrum (126 MHz, room temperature, $CDCl_3$) of (**5'f**)

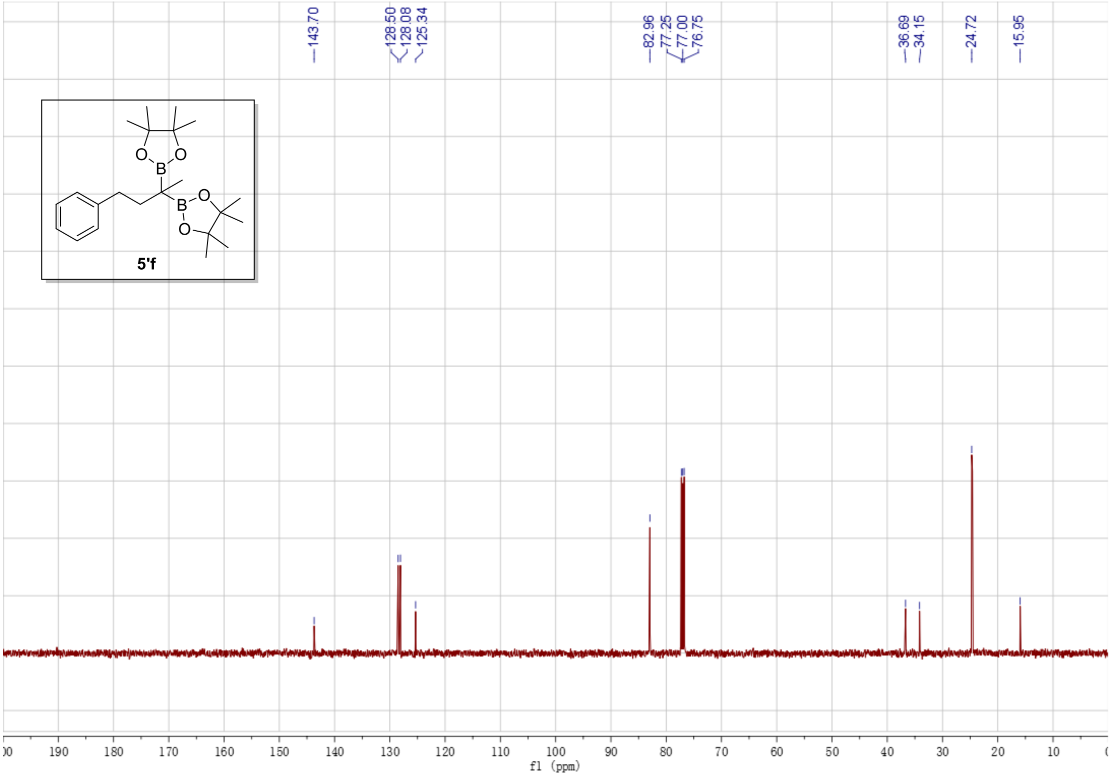

Supplementary Figure 53. ^{13}C NMR spectrum of **5'f**.

^{11}B spectrum (128 MHz, room temperature, $CDCl_3$) of (**5'f**)

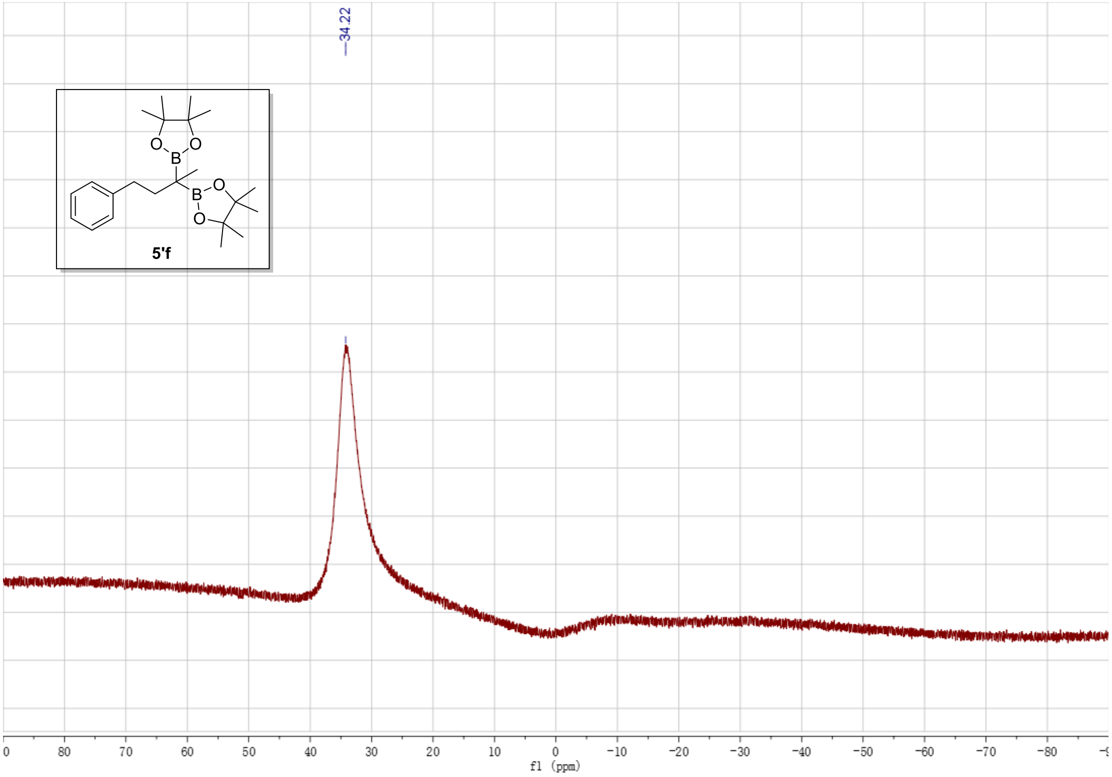

Supplementary Figure 54. ^{11}B spectrum of **5'f**.

2,2'-(4-(4-methoxyphenyl)butane-2,2-diyl)bis(4,4,5,5-tetramethyl-1,3,2-dioxaborolane) (5'g)

^{1}H spectrum (400 MHz, room temperature, $CDCl_3$) of (**5'g**)

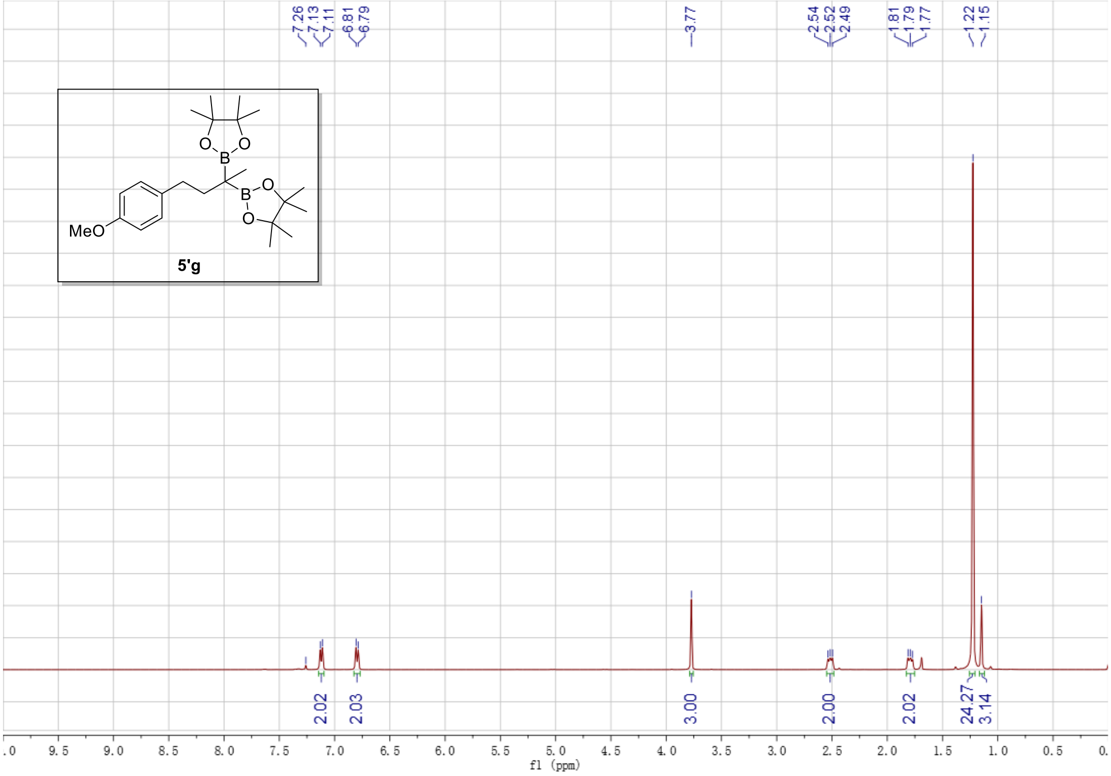

Supplementary Figure 55. ^{1}H NMR spectrum of **5'g**.

^{13}C spectrum (126 MHz, room temperature, $CDCl_3$) of (**5'g**)

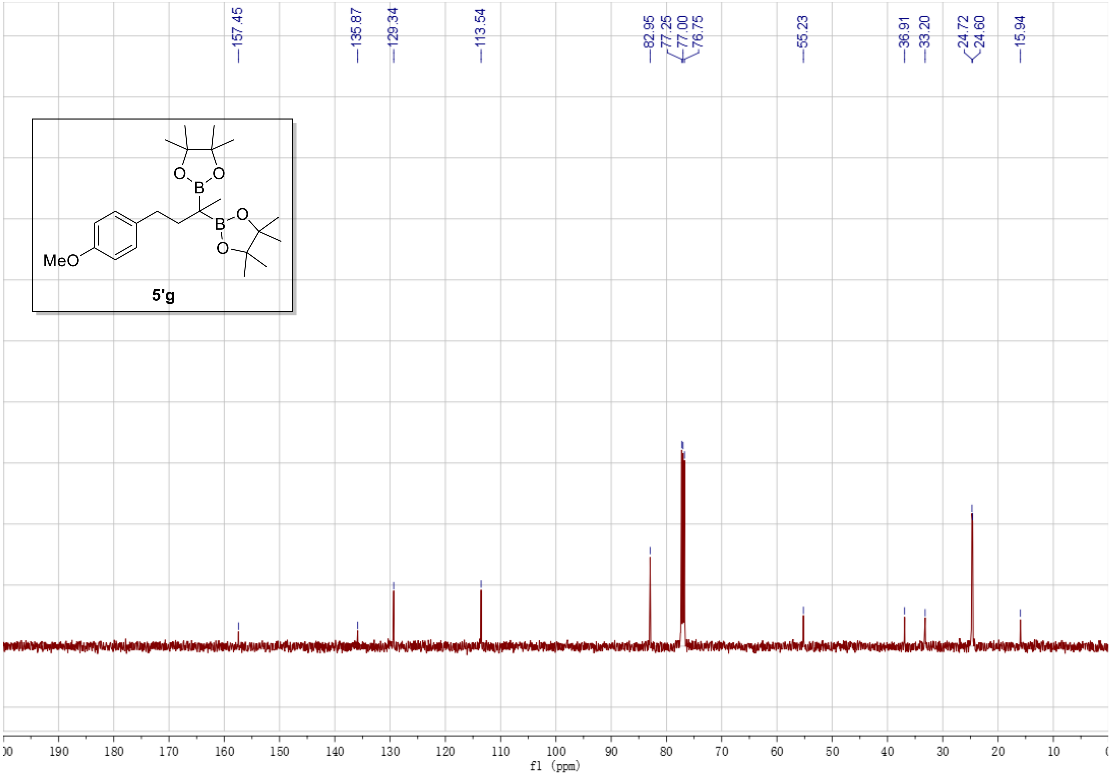

Supplementary Figure 56. ^{13}C NMR spectrum of **5'g**.

^{11}B spectrum (128 MHz, room temperature, $CDCl_3$) of (**5'g**)

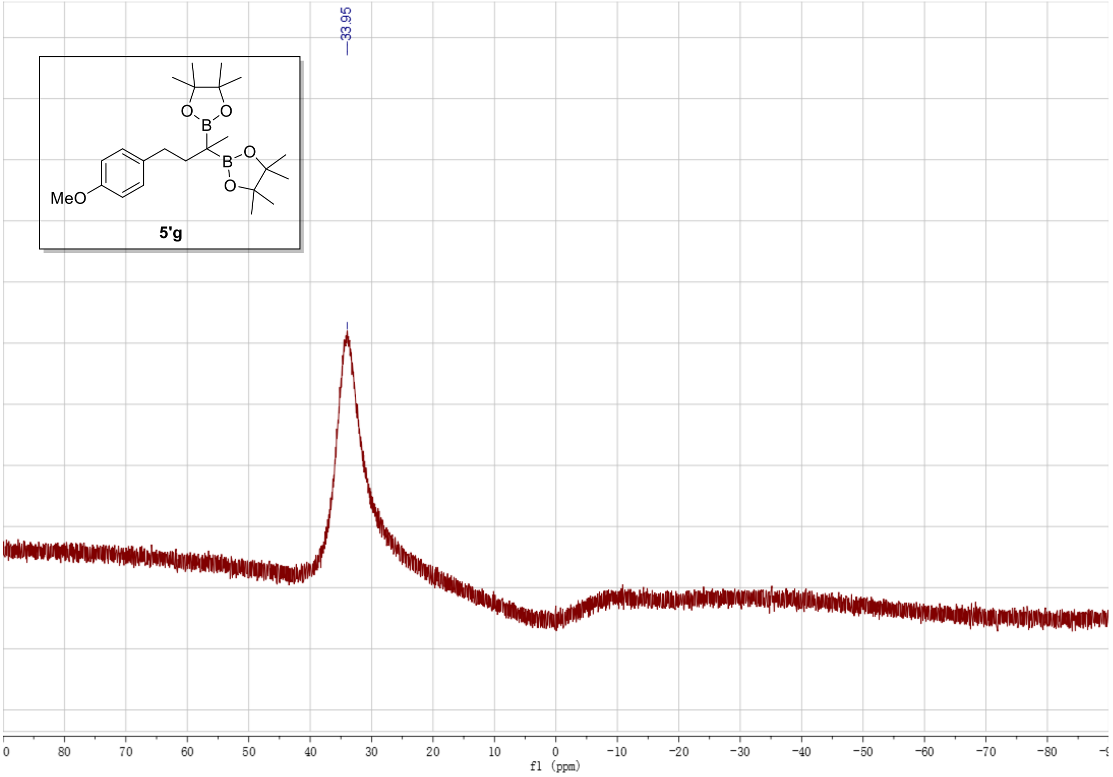

Supplementary Figure 57. ^{11}B spectrum of **5'g**.

2-(2-(3,3-dimethylbutyl)phenyl)-4,4,5,5-tetramethyl-1,3,2-dioxaborolane (4a)

^{1}H spectrum (400 MHz, room temperature, $CDCl_3$) of (**4a**)

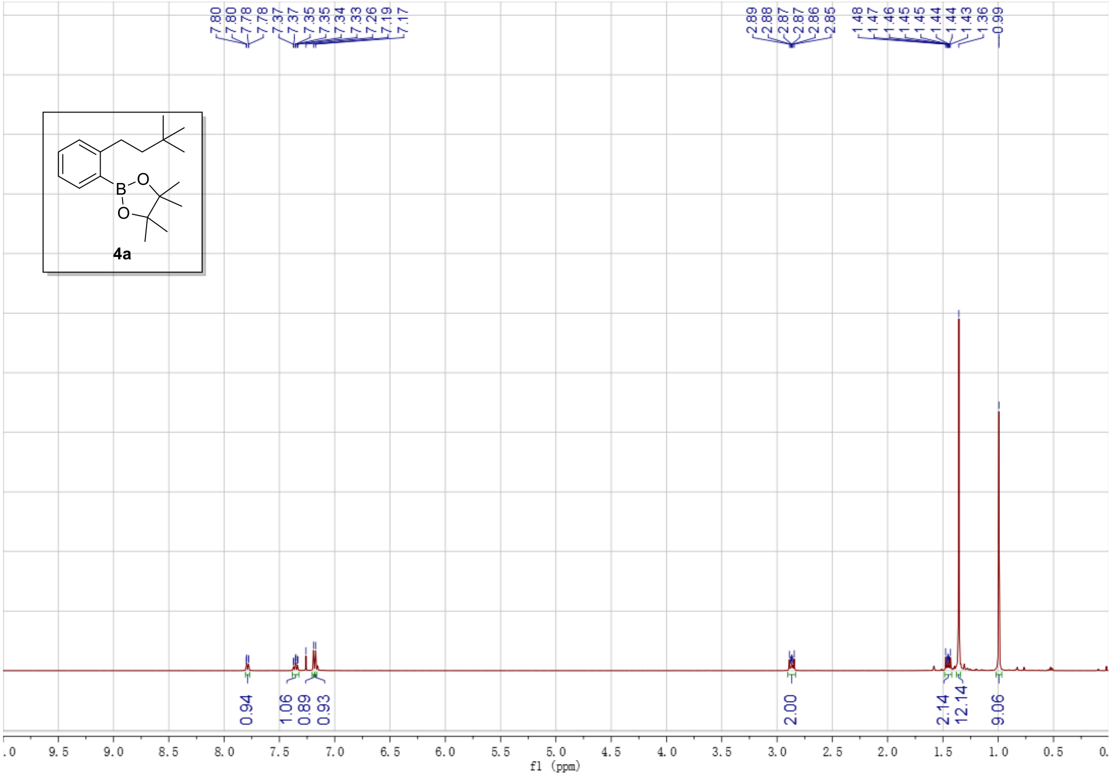

Supplementary Figure 58. ^{1}H NMR spectrum of **4a**.

^{13}C spectrum (101 MHz, room temperature, $CDCl_3$) of (**4a**)

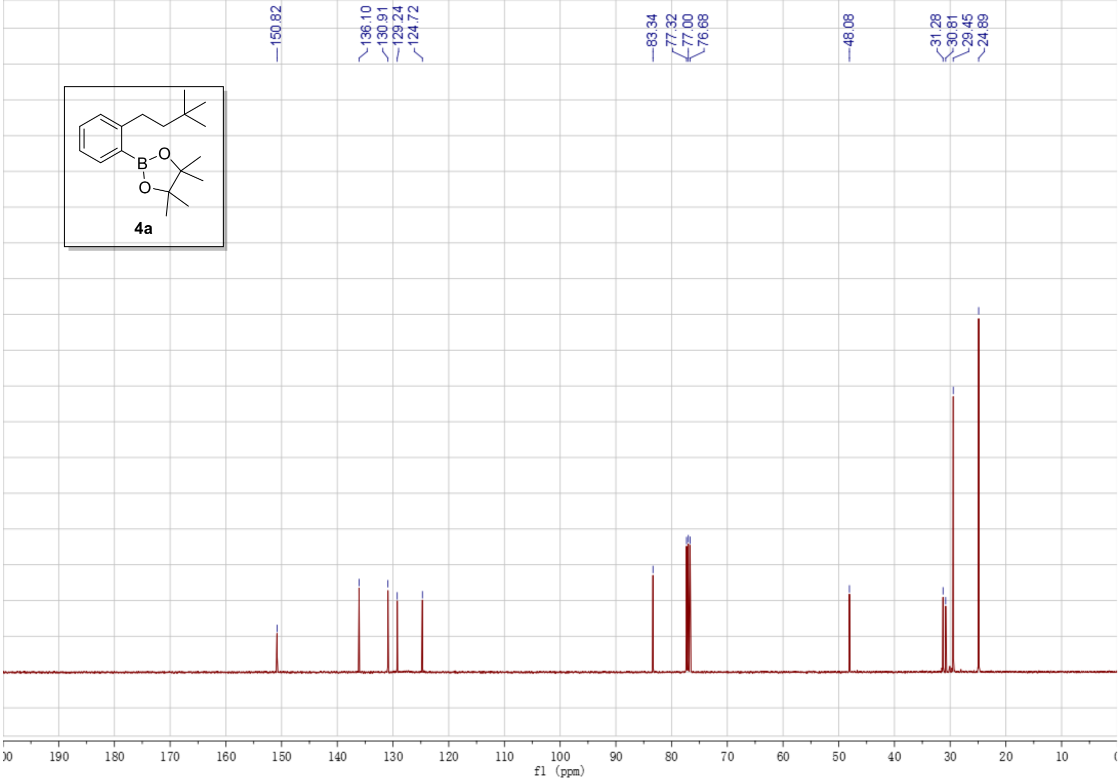

Supplementary Figure 59. ^{13}C NMR spectrum of **4a**.

^{11}B spectrum (128 MHz, room temperature, $CDCl_3$) of (**4a**)

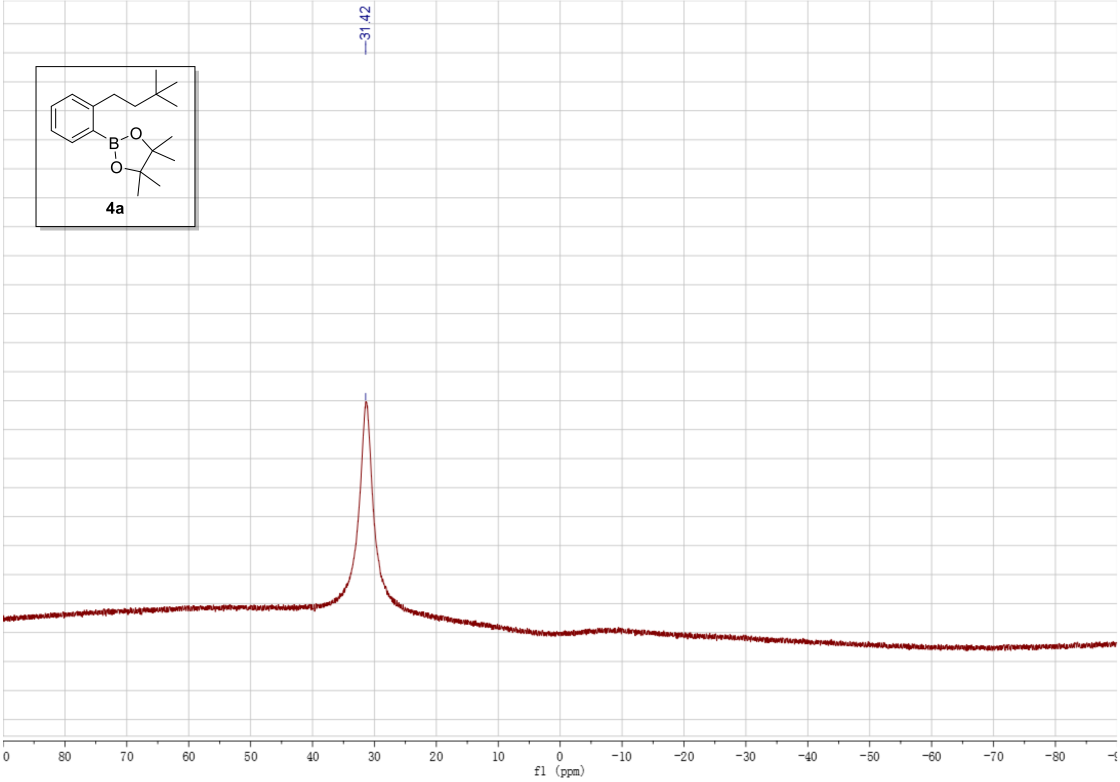

Supplementary Figure 60. ^{11}B spectrum of **4a**.

2-(2-(3-ethyl-3-methylpentyl)phenyl)-4,4,5,5-tetramethyl-1,3,2-dioxaborolane (4b)

^{1}H spectrum (500 MHz, room temperature, $CDCl_3$) of (**4b**)

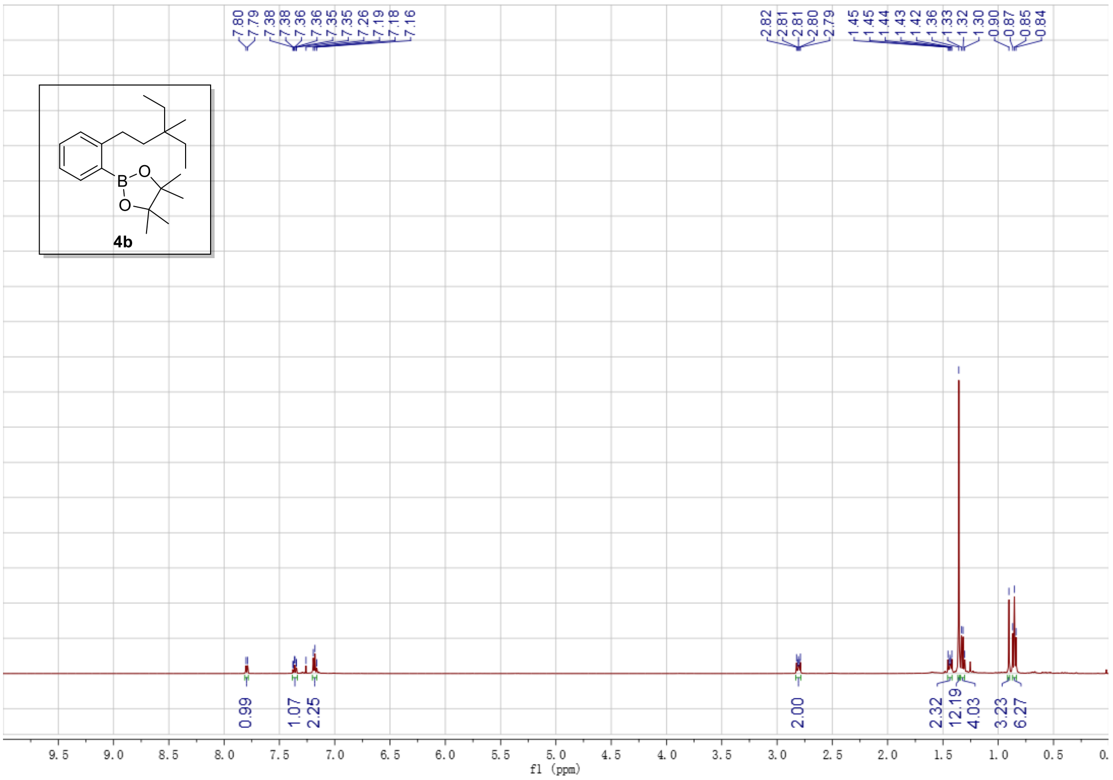

Supplementary Figure 61. ^{1}H NMR spectrum of **4b**.

^{13}C spectrum (126 MHz, room temperature, $CDCl_3$) of (**4b**)

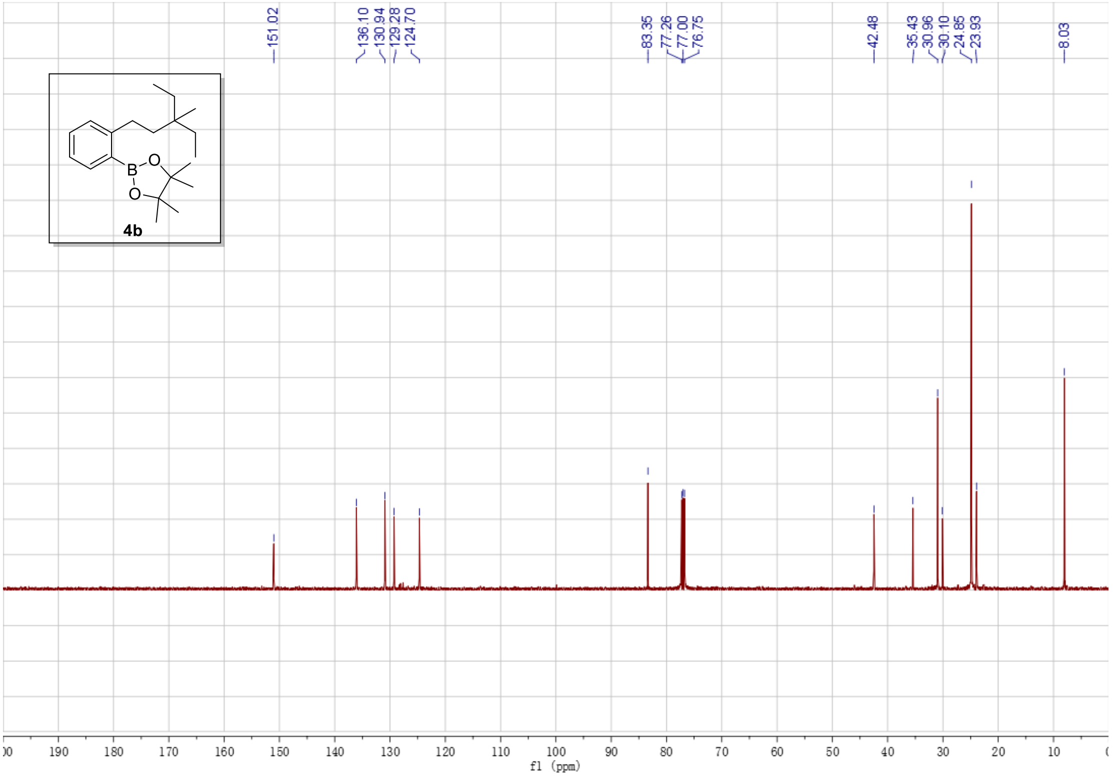

Supplementary Figure 62. ^{13}C NMR spectrum of **4b**.

^{11}B spectrum (128 MHz, room temperature, $CDCl_3$) of (**4b**)

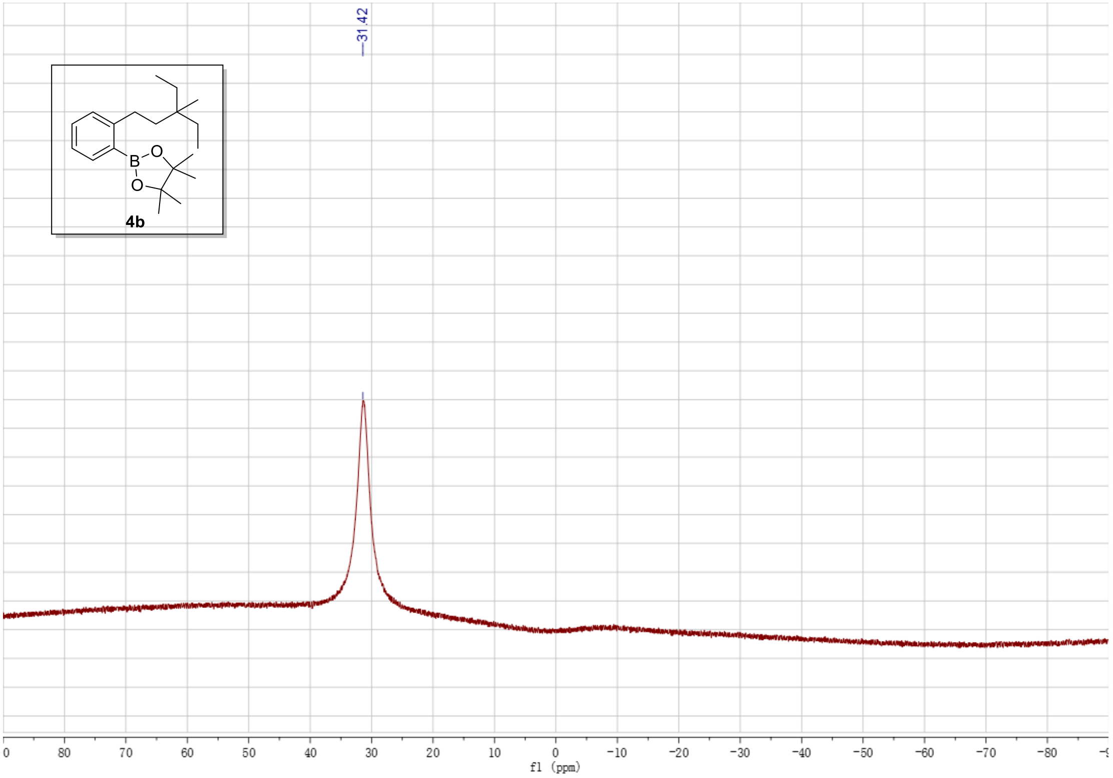

Supplementary Figure 63. ^{11}B spectrum of **4b**.

2-(2-(2-((1r,3s)-adamantan-1-yl)ethyl)phenyl)-4,4,5,5-tetramethyl-1,3,2-dioxaborolane (4c)

^{1}H spectrum (400 MHz, room temperature, $CDCl_3$) of (**4c**)

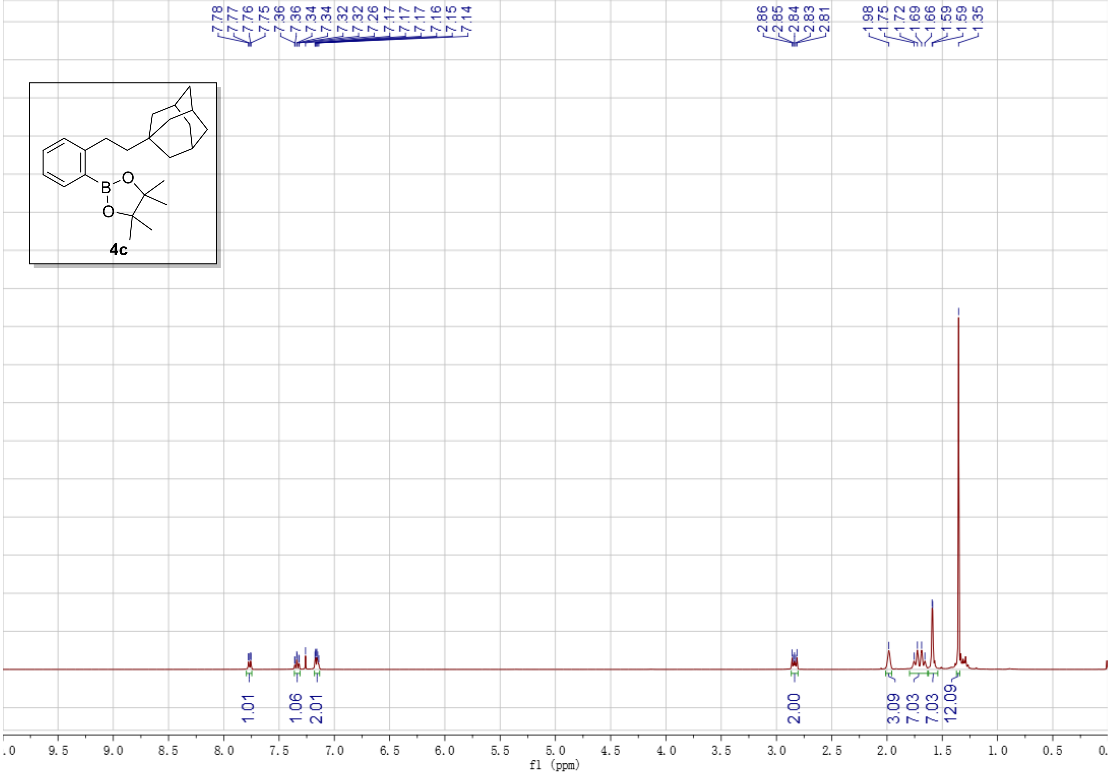

Supplementary Figure 64. ^{1}H NMR spectrum of **4c**.

^{13}C spectrum (126 MHz, room temperature, $CDCl_3$) of (**4c**)

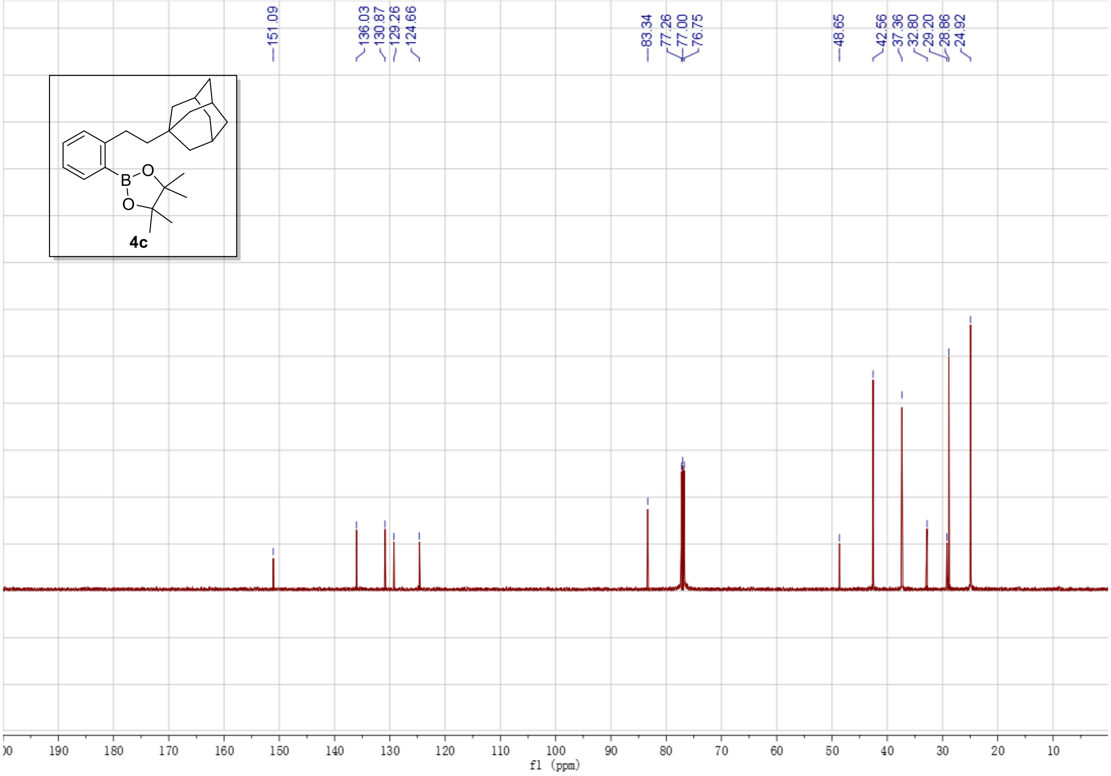

Supplementary Figure 65. ^{13}C NMR spectrum of **4c**.

^{11}B spectrum (128 MHz, room temperature, $CDCl_3$) of (**4c**)

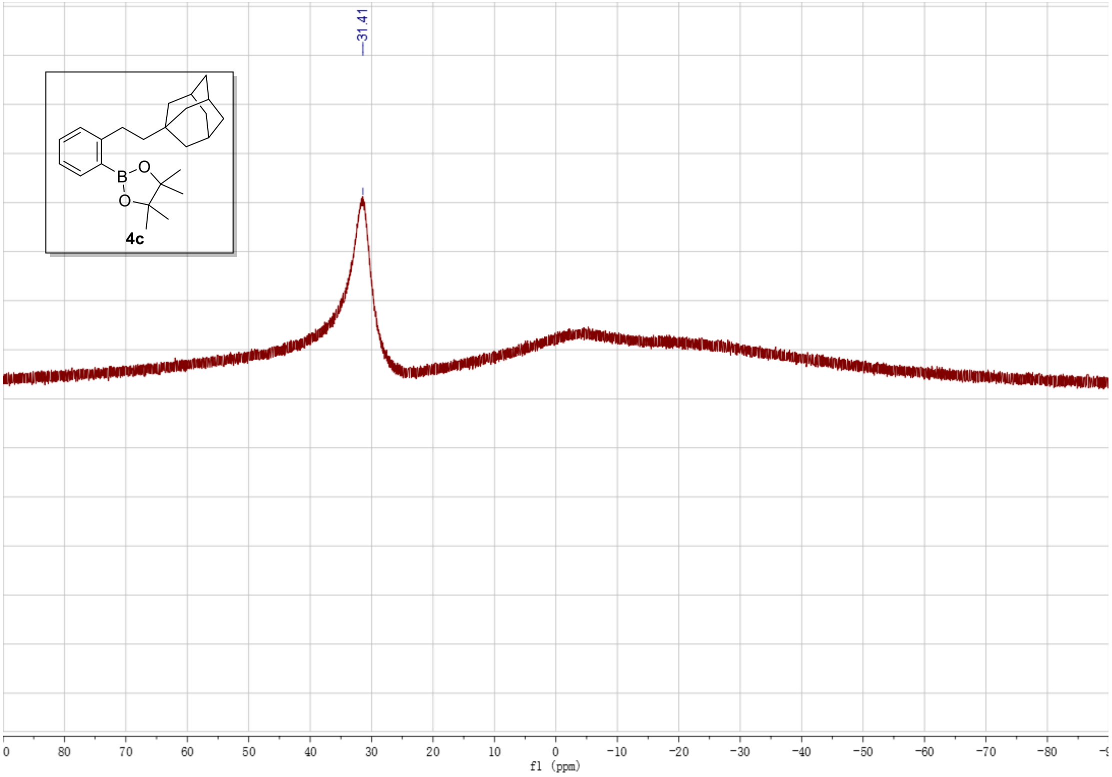

Supplementary Figure 66. ^{11}B spectrum of **4c**.

4,4,5,5-tetramethyl-2-(2-(2-(3,3,9-trimethyl-1,5-dioxaspiro[5.5]undecan-9-yl)ethyl)phenyl)-1,3,2-dioxaborolane (4d)

^{1}H spectrum (500 MHz, room temperature, $CDCl_3$) of (**4d**)

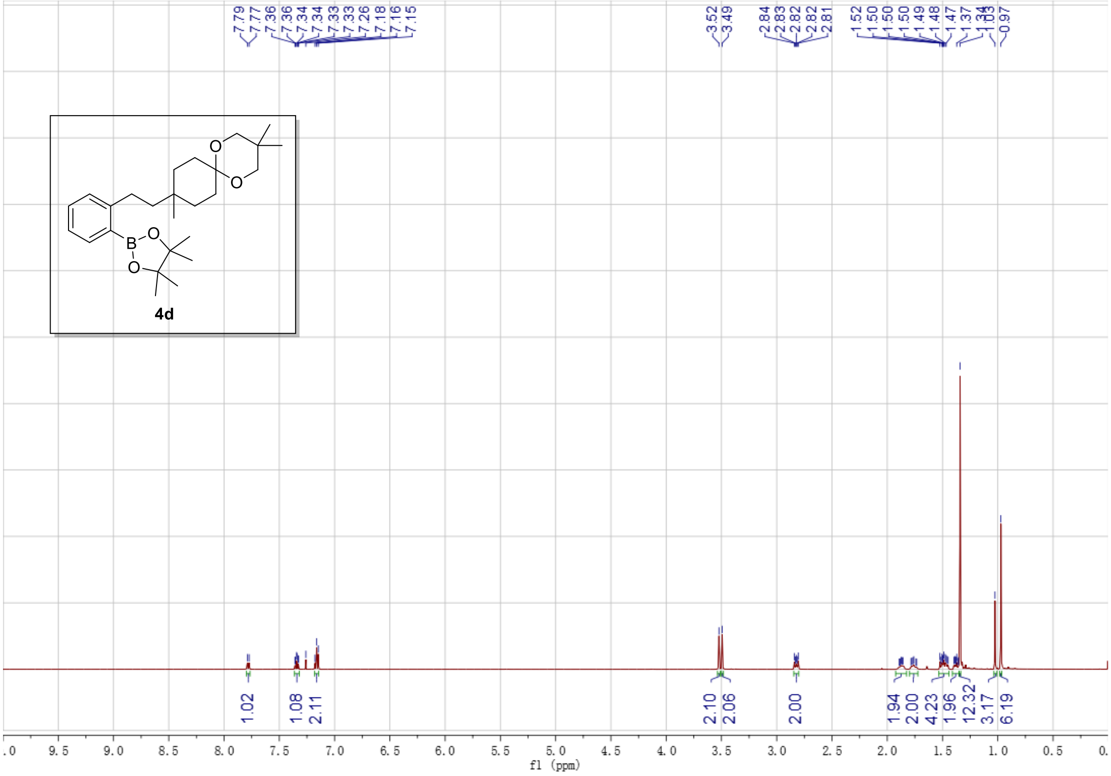

Supplementary Figure 67. ^{1}H NMR spectrum of **4d**.

^{13}C spectrum (126 MHz, room temperature, $CDCl_3$) of (**4d**)

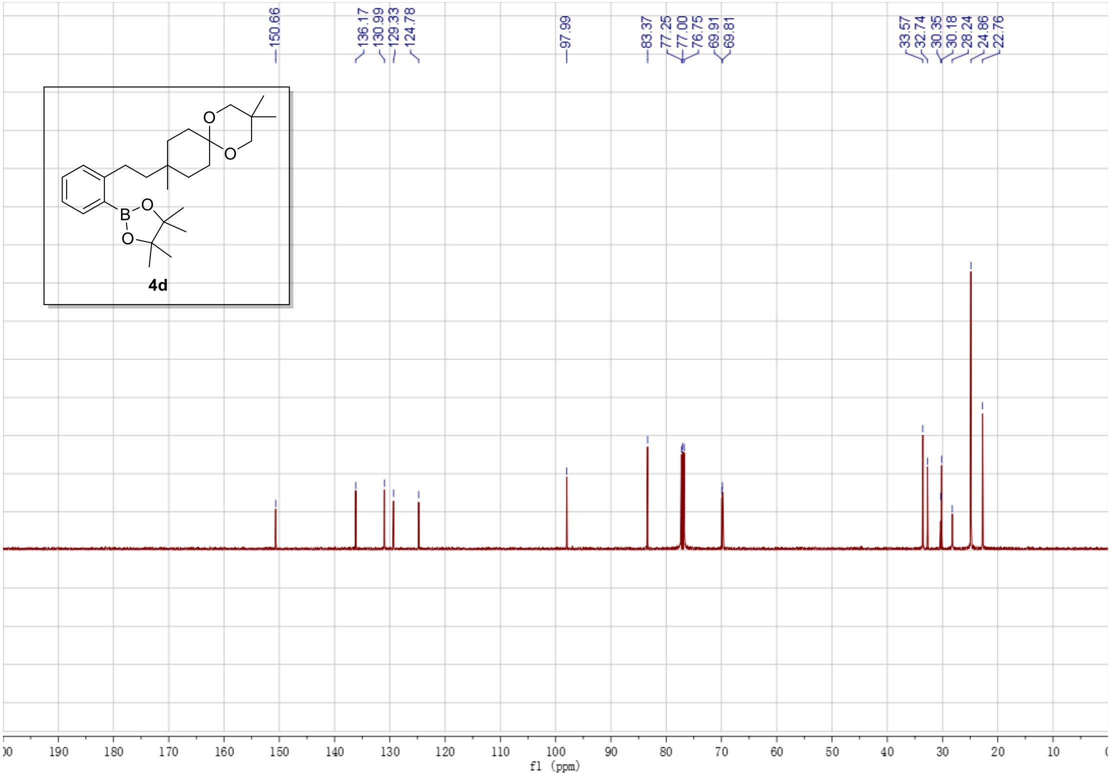

Supplementary Figure 68. ^{13}C NMR spectrum of **4d**.

^{11}B spectrum (128 MHz, room temperature, $CDCl_3$) of (**4d**)

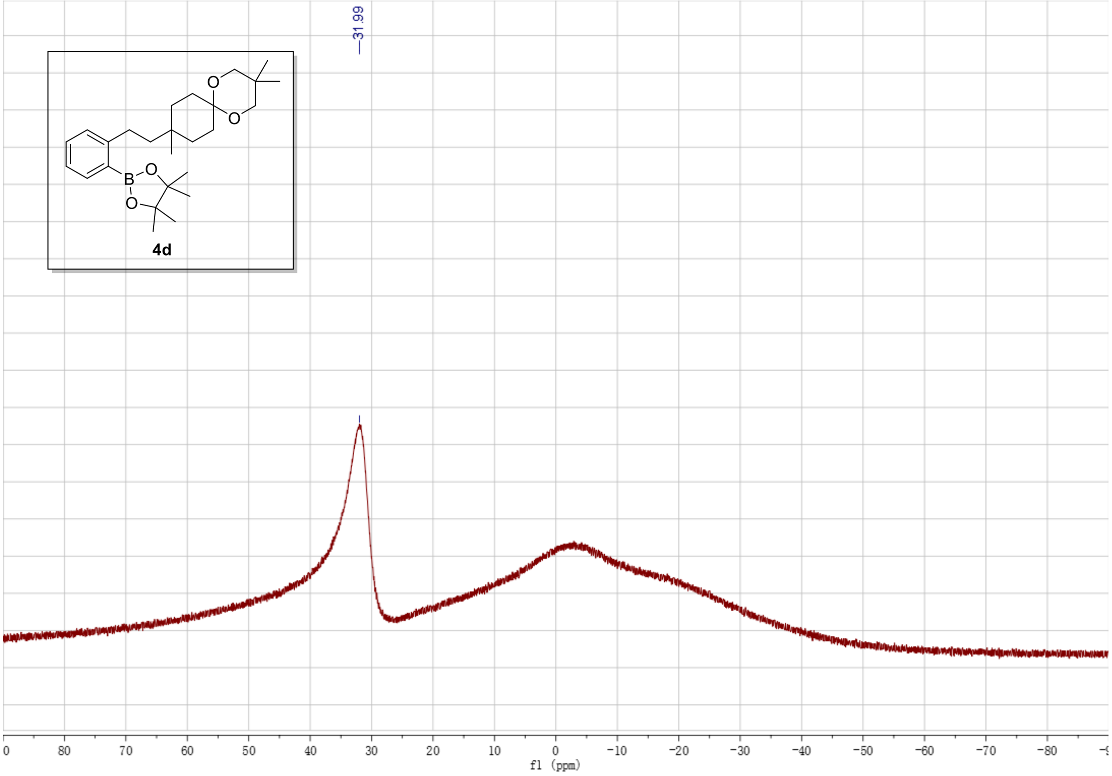

Supplementary Figure 69. ^{11}B spectrum of **4d**.

Ethyl3,3-dimethyl-5-(2-(4,4,5,5-tetramethyl-1,3,2-dioxaborolan-2-yl)phenyl)pentanoate (4e)

^{1}H spectrum (500 MHz, room temperature, $CDCl_3$) of (**4e**)

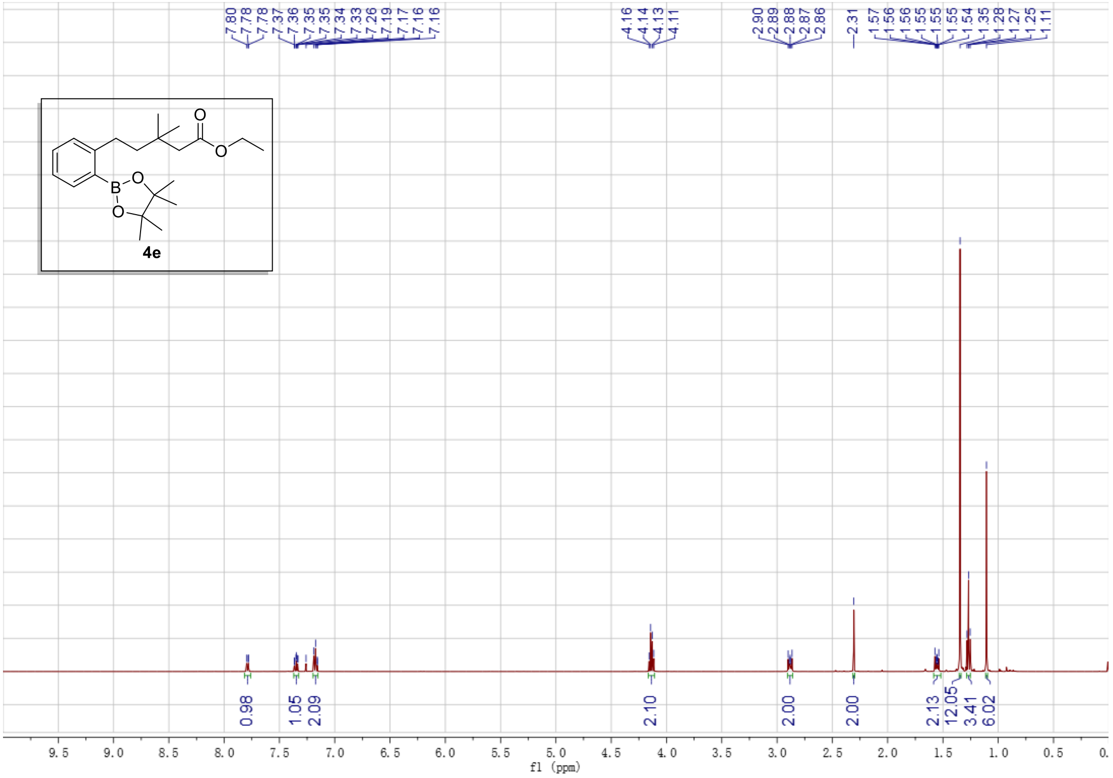

Supplementary Figure 70. ^{1}H NMR spectrum of **4e**.

^{13}C spectrum (126 MHz, room temperature, $CDCl_3$) of (**4e**)

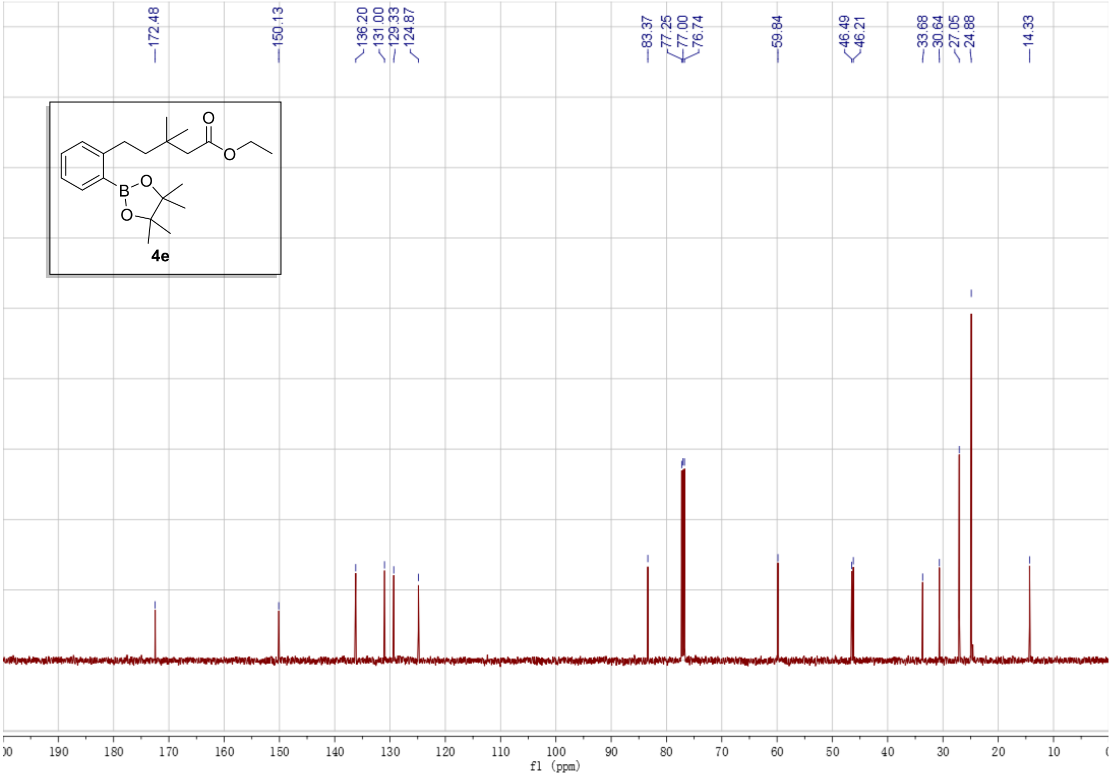

Supplementary Figure 71. ^{13}C NMR spectrum of **4e**.

^{11}B spectrum (128 MHz, room temperature, $CDCl_3$) of (**4e**)

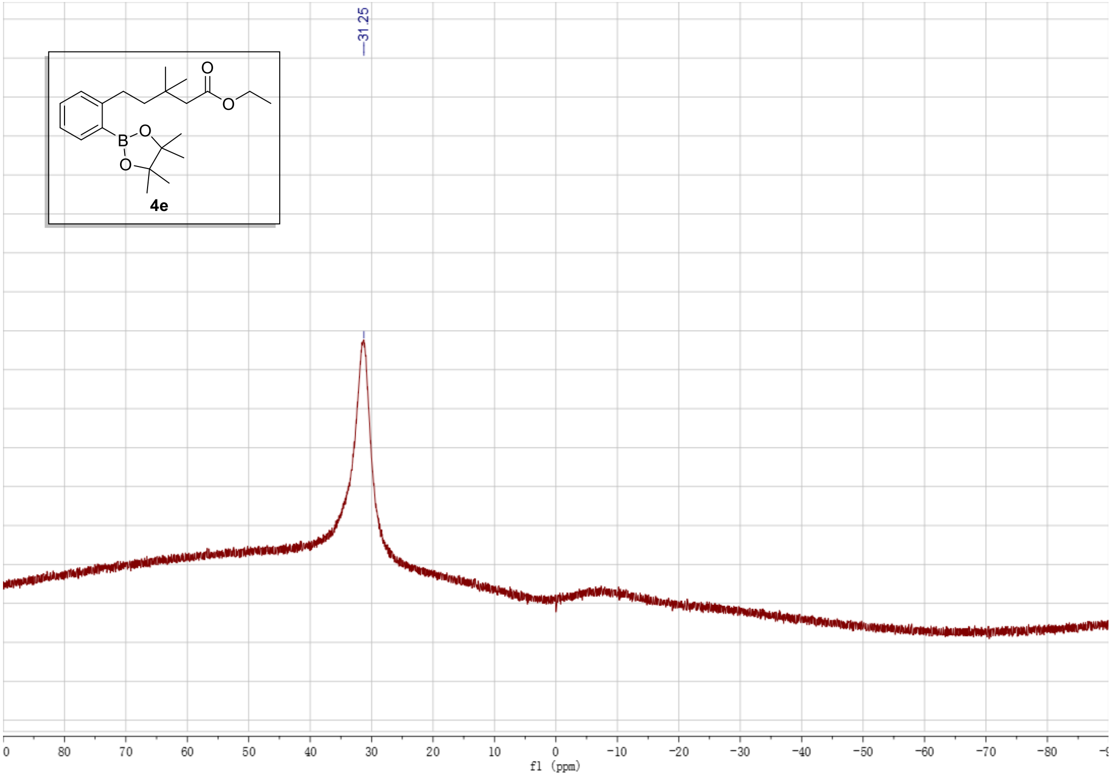

Supplementary Figure 72. ^{11}B spectrum of **4e**.

Benzyl-4-methyl-4-(2-(4,4,5,5-tetramethyl-1,3,2-dioxaborolan-2-yl)phenethyl)piperidine-1-carboxylate (4f)

^{1}H spectrum (500 MHz, room temperature, $CDCl_3$) of (**4f**)

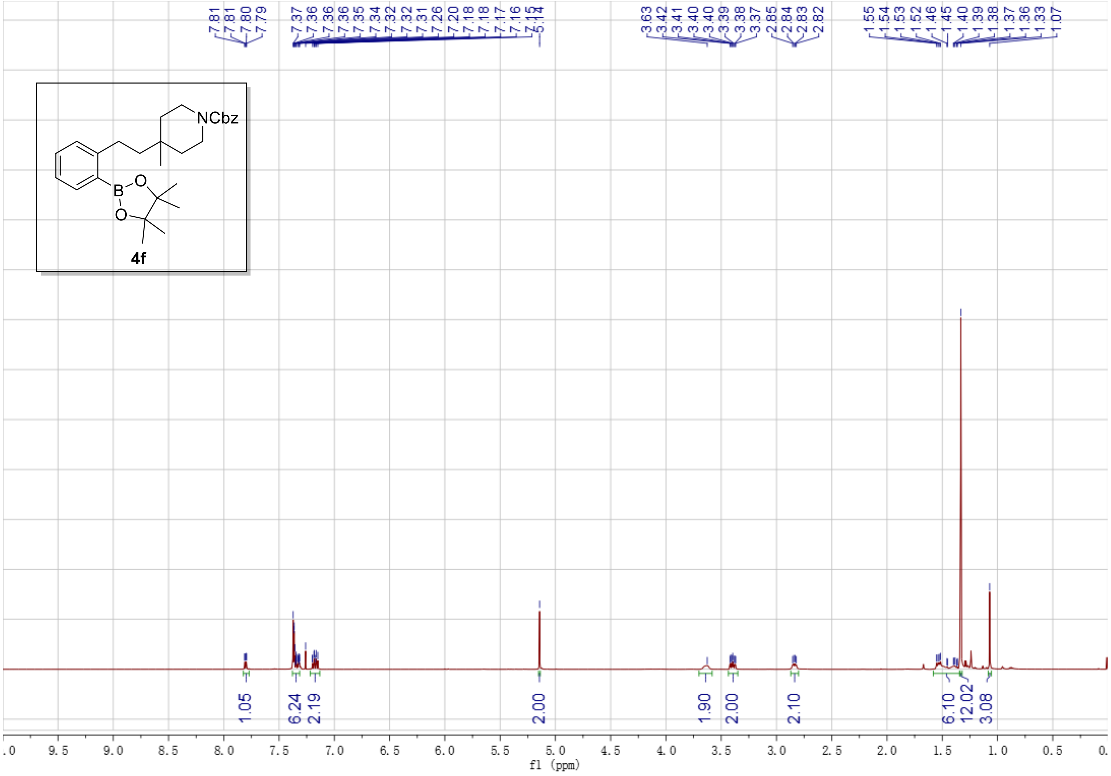

Supplementary Figure 73. ^{1}H NMR spectrum of **4f**.

^{13}C spectrum (126 MHz, room temperature, $CDCl_3$) of (**4f**)

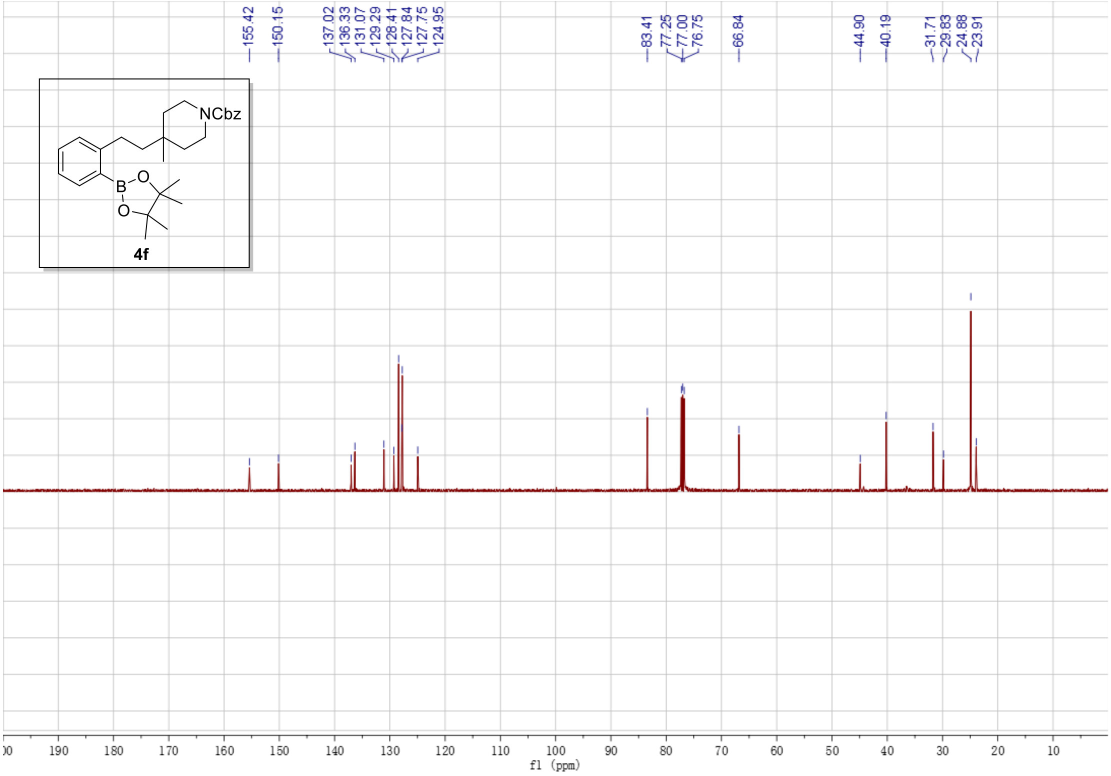

Supplementary Figure 74. ^{13}C NMR spectrum of **4f**.

^{11}B spectrum (128 MHz, room temperature, $CDCl_3$) of (**4f**)

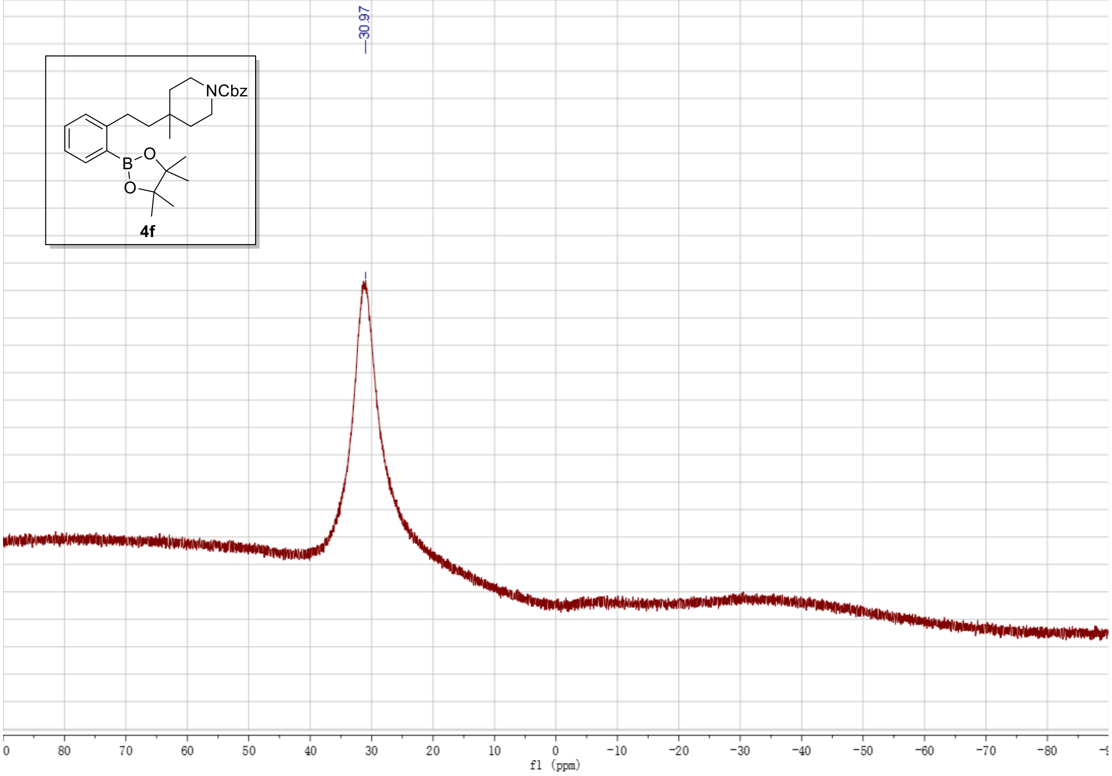

Supplementary Figure 75. ^{11}B spectrum of **4f**.

Tert-butyl((3,3-dimethyl-5-(2-(4,4,5,5-tetramethyl-1,3,2-dioxaborolan-2-yl)phenyl)pentyl)oxy)diphenylsilane (4g)

^{1}H spectrum (500 MHz, room temperature, $CDCl_3$) of (**4g**)

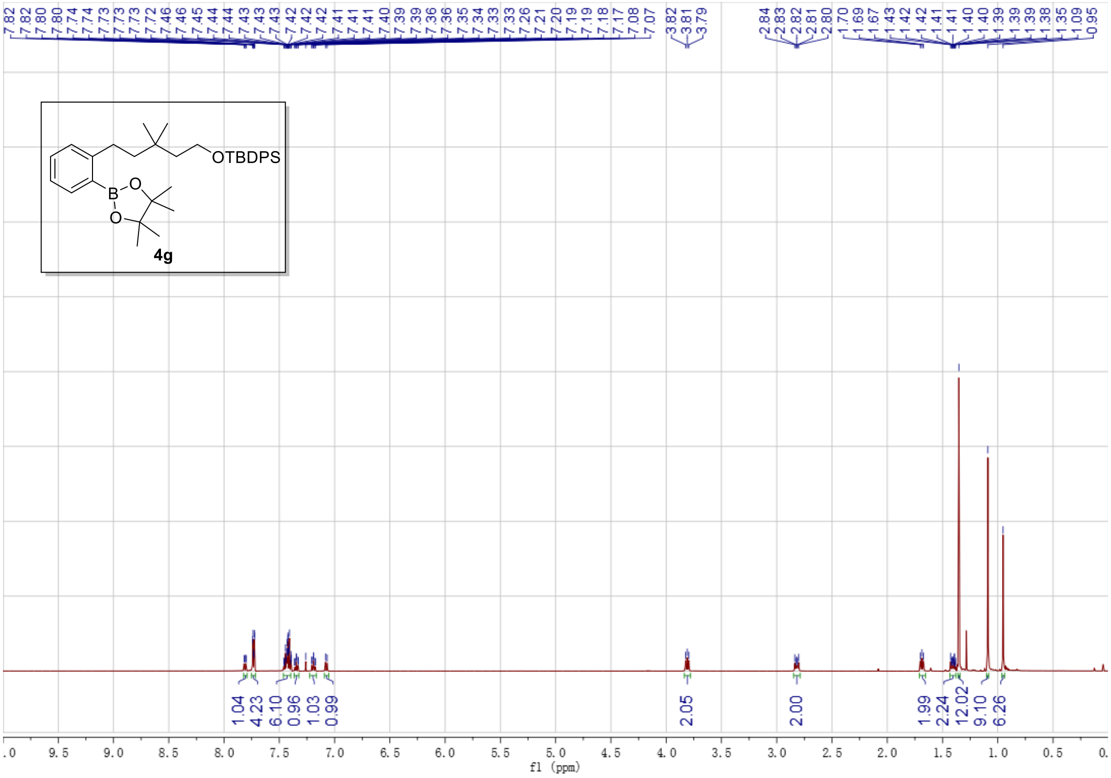

Supplementary Figure 76. ^{1}H NMR spectrum of **4g**.

^{13}C spectrum (126 MHz, room temperature, $CDCl_3$) of (**4g**)

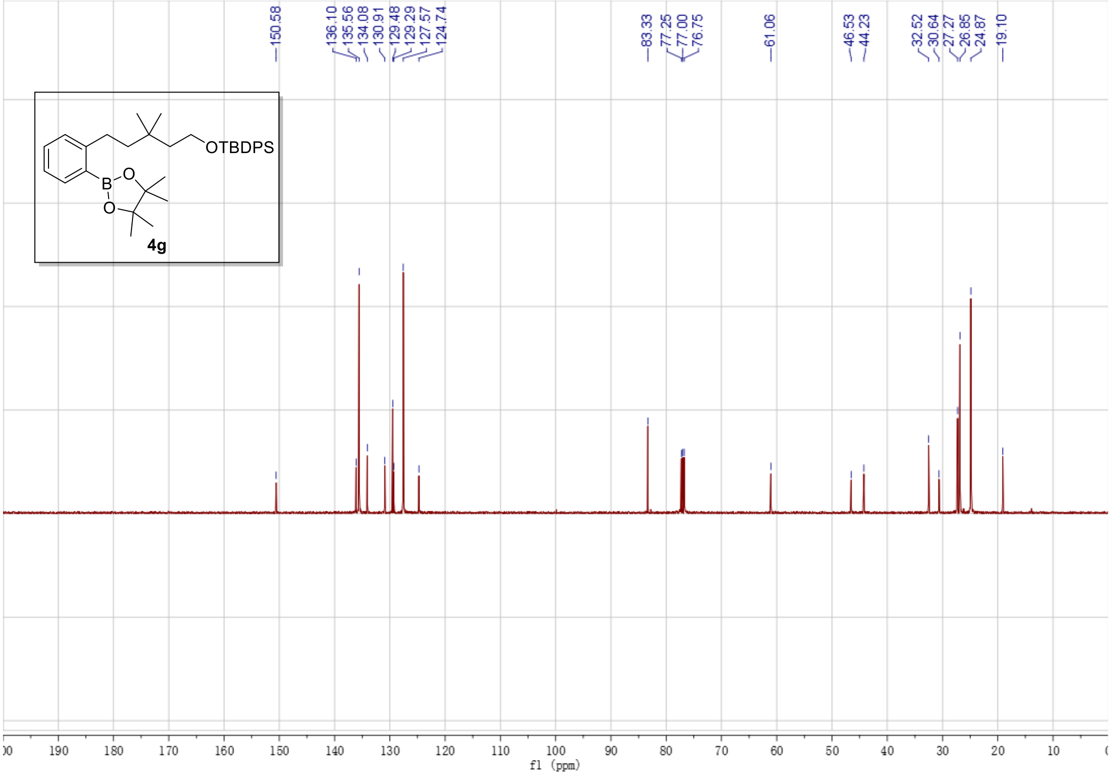

Supplementary Figure 77. ^{13}C NMR spectrum of **4g**.

^{11}B spectrum (128 MHz, room temperature, $CDCl_3$) of (**4g**)

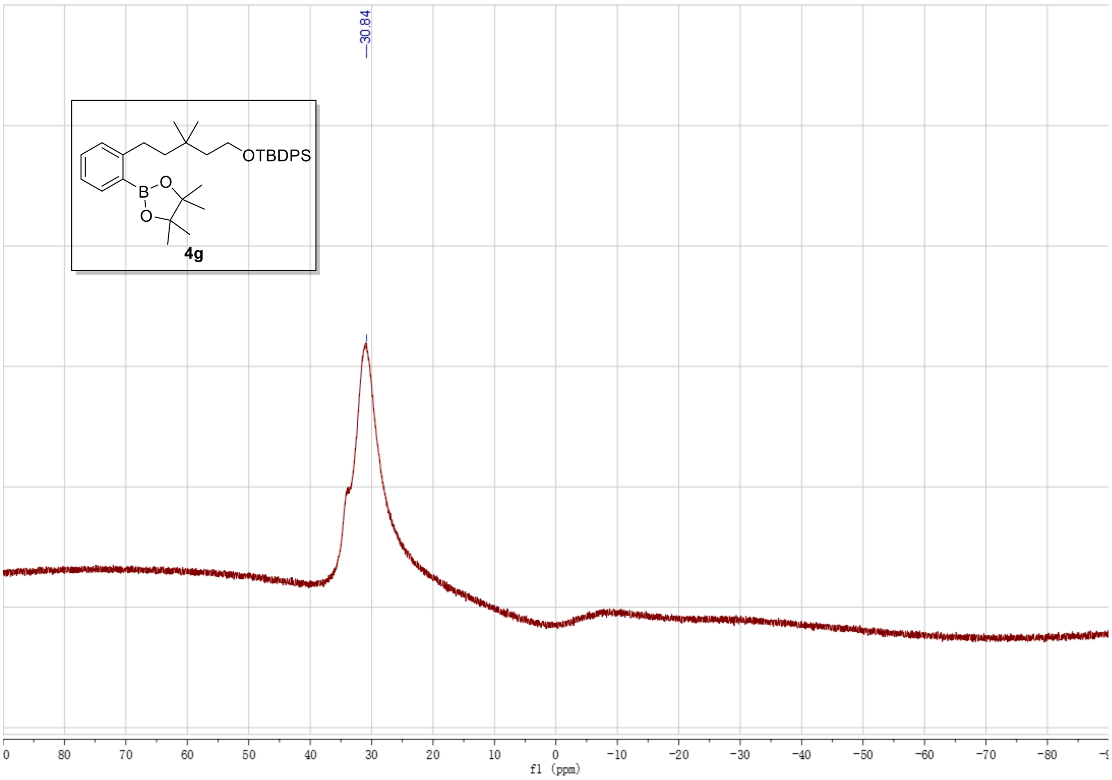

Supplementary Figure 78. ^{11}B spectrum of **4g**.

4,4,5,5-tetramethyl-2-(2-(2-(1-methyl-4-phenylcyclohexyl)ethyl)phenyl)-1,3,2-dioxaborolane (4h)

^{1}H spectrum (500 MHz, room temperature, $CDCl_3$) of (**4h**)

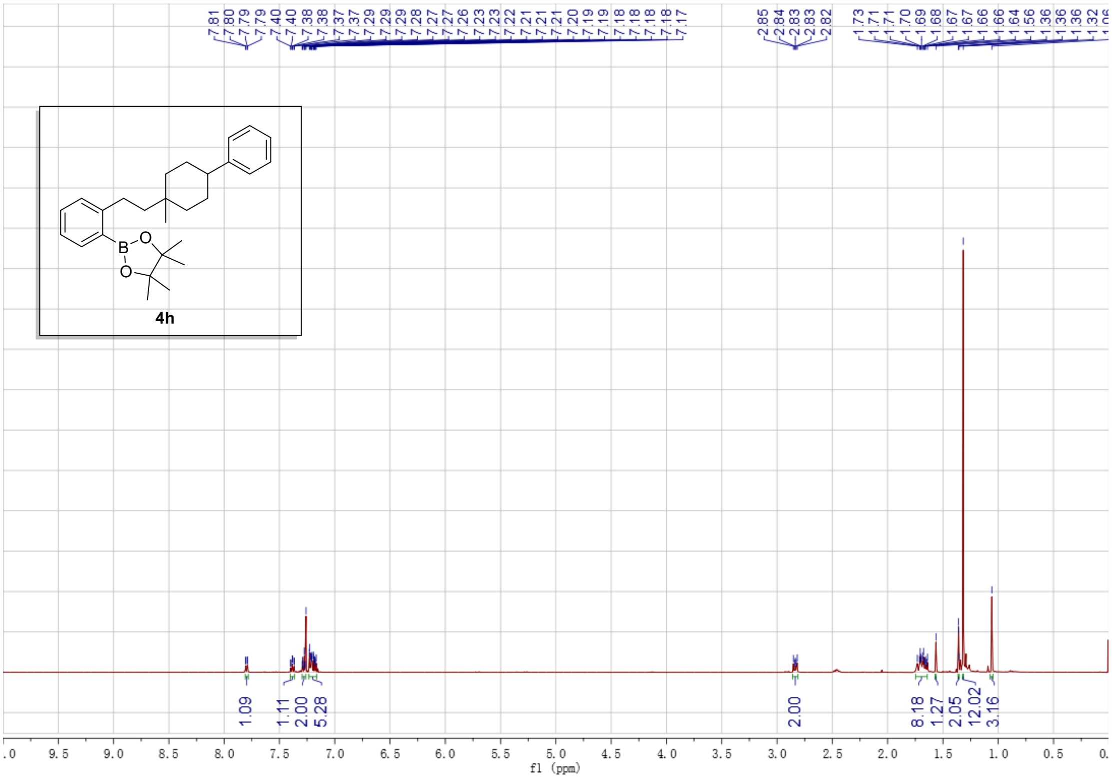

Supplementary Figure 79. ^{1}H NMR spectrum of **4h**.

^{13}C spectrum (126 MHz, room temperature, $CDCl_3$) of (**4h**)

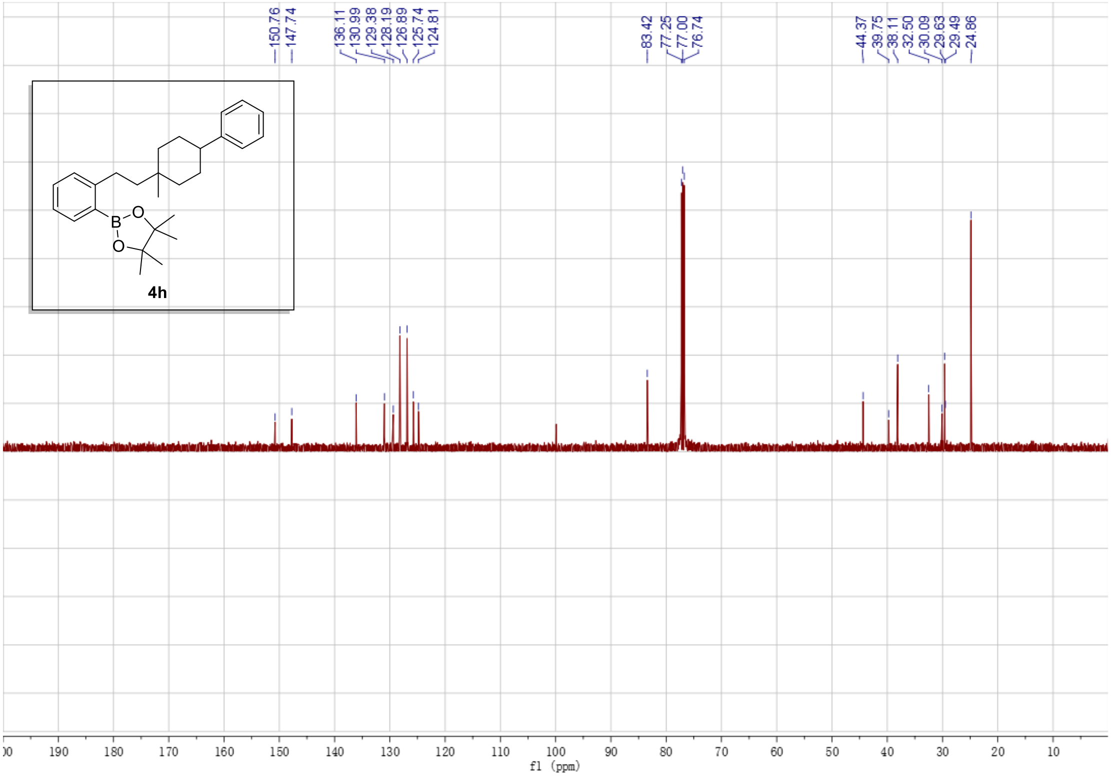

Supplementary Figure 80. ^{13}C NMR spectrum of **4h**.

^{11}B spectrum (128 MHz, room temperature, $CDCl_3$) of (**4h**)

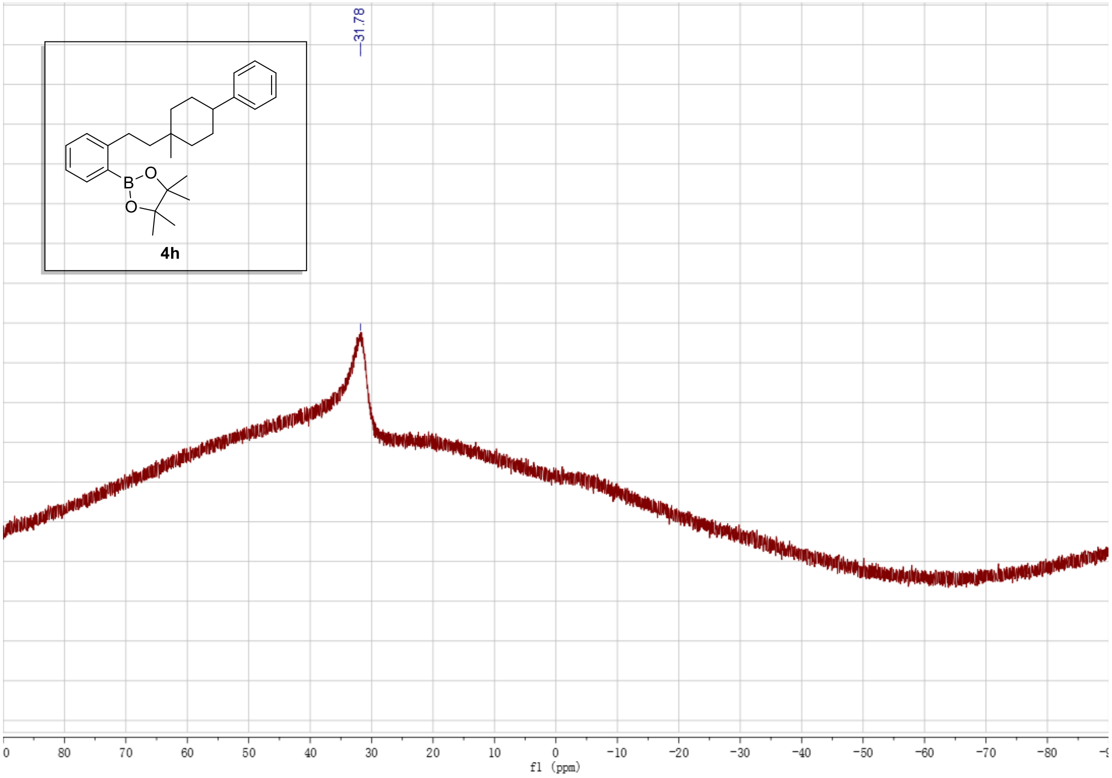

Supplementary Figure 81. ^{11}B spectrum of **4h**.

2-(2-(3,3-dimethyl-5-phenylpentyl)phenyl)-4,4,5,5-tetramethyl-1,3,2-dioxaborolane (4i)

^{1}H spectrum (500 MHz, room temperature, $CDCl_3$) of (**4i**)

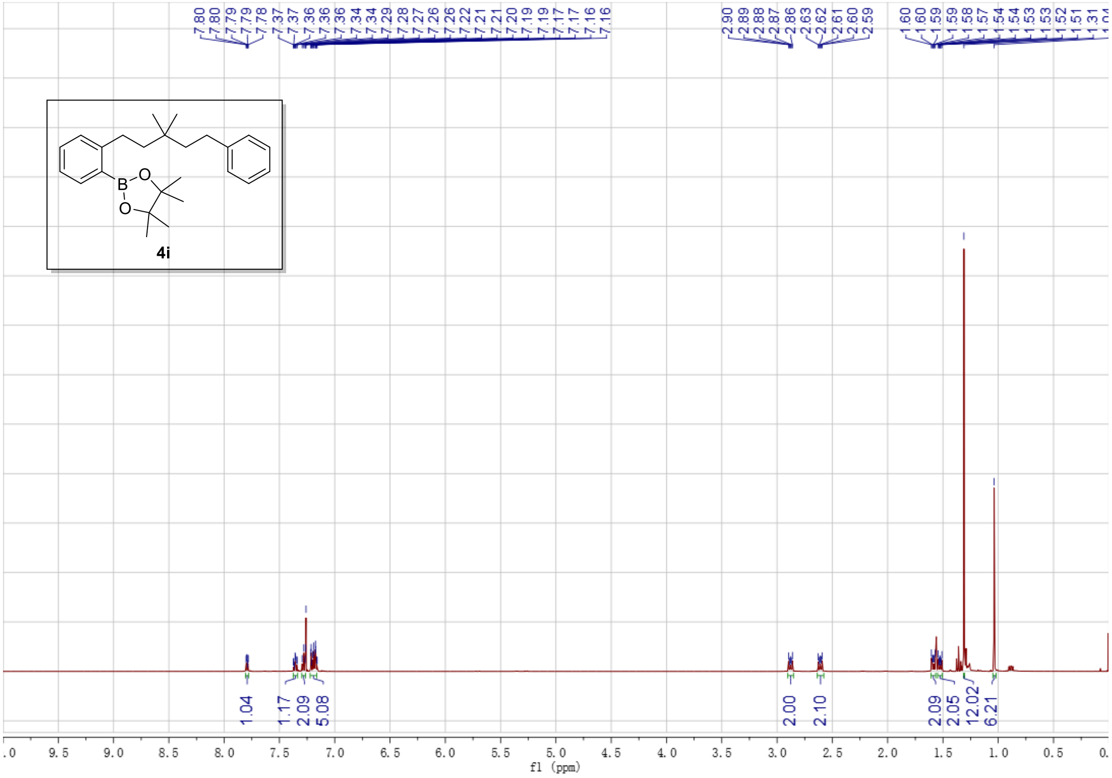

Supplementary Figure 82. ^{1}H NMR spectrum of **4i**.

^{13}C spectrum (126 MHz, room temperature, $CDCl_3$) of (**4i**)

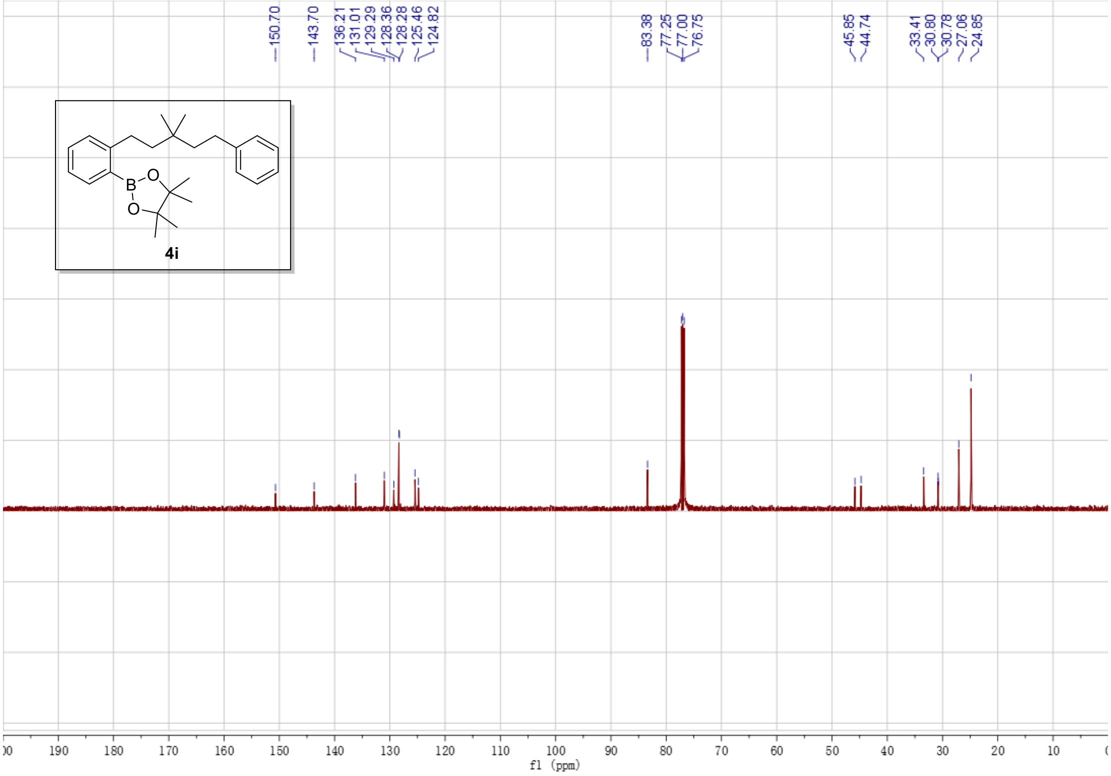

Supplementary Figure 83. ^{13}C NMR spectrum of **4i**.

^{11}B spectrum (128 MHz, room temperature, $CDCl_3$) of (**4i**)

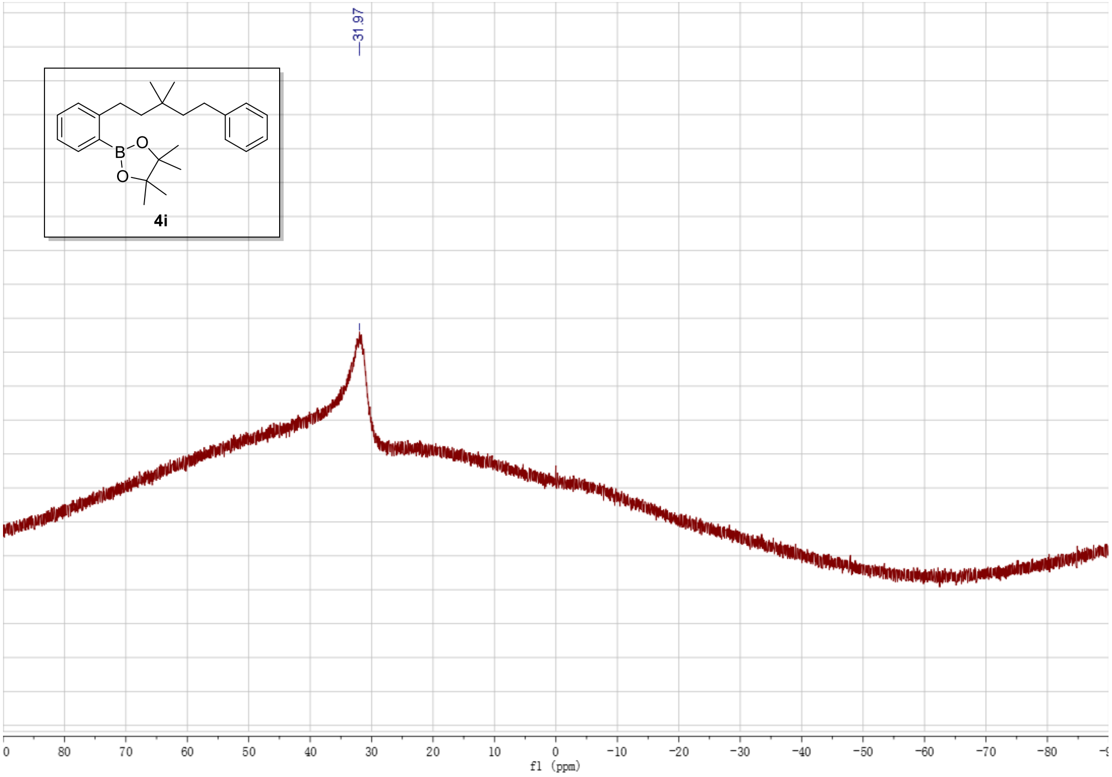

Supplementary Figure 84. ^{11}B spectrum of **4i**.

2-(2-(5-(4-methoxyphenyl)-3,3-dimethylpentyl)phenyl)-4,4,5,5-tetramethyl-1,3,2-dioxaborolane (4j)

^{1}H spectrum (500 MHz, room temperature, $CDCl_3$) of (**4j**)

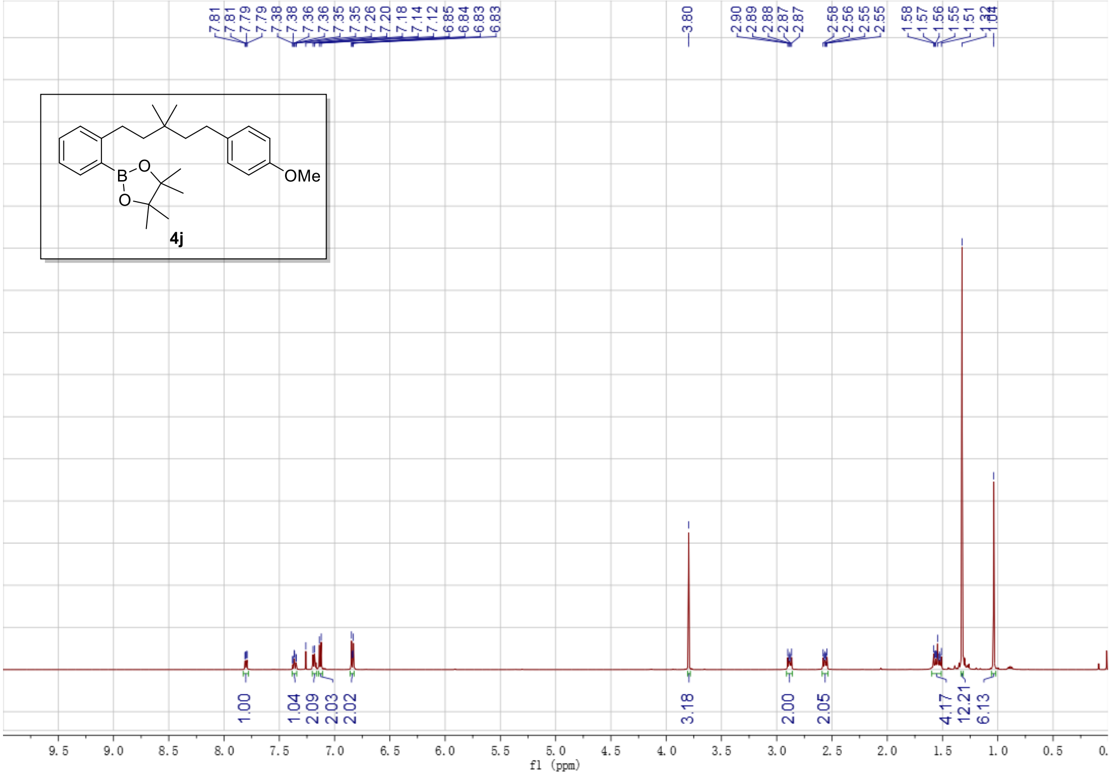

Supplementary Figure 85. ^{1}H NMR spectrum of **4j**.

^{13}C spectrum (126 MHz, room temperature, $CDCl_3$) of (**4j**)

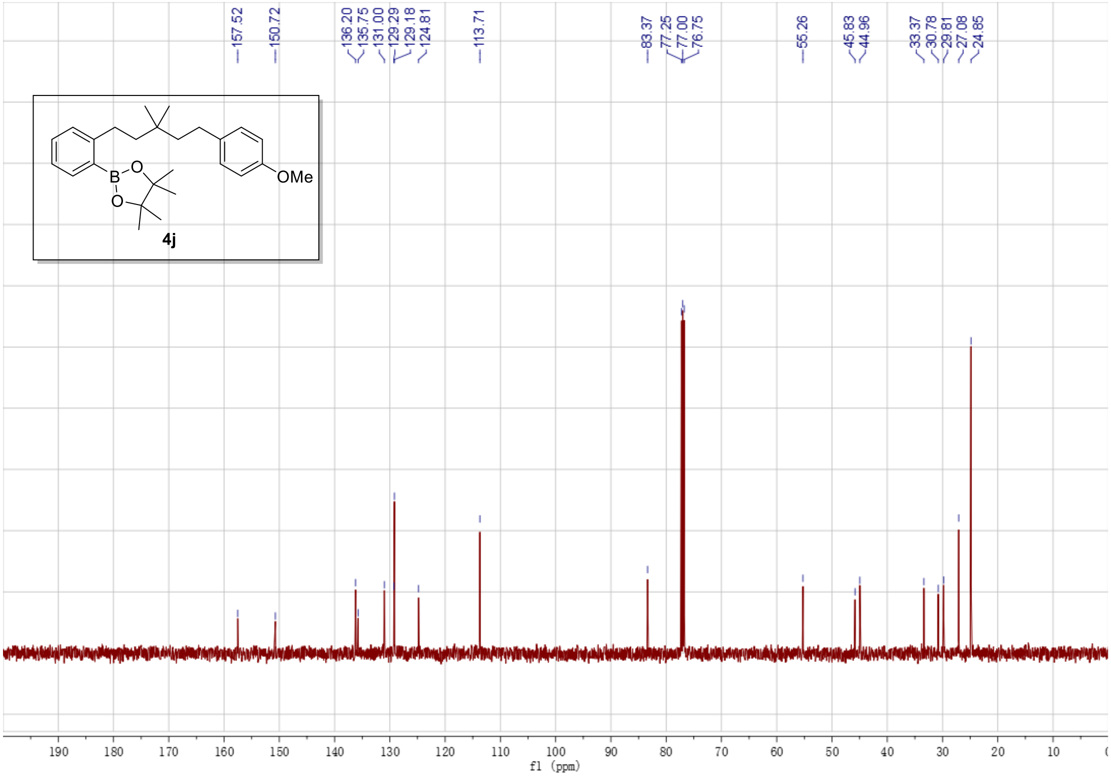

Supplementary Figure 86. ^{13}C NMR spectrum of **4j**.

^{11}B spectrum (128 MHz, room temperature, $CDCl_3$) of (**4j**)

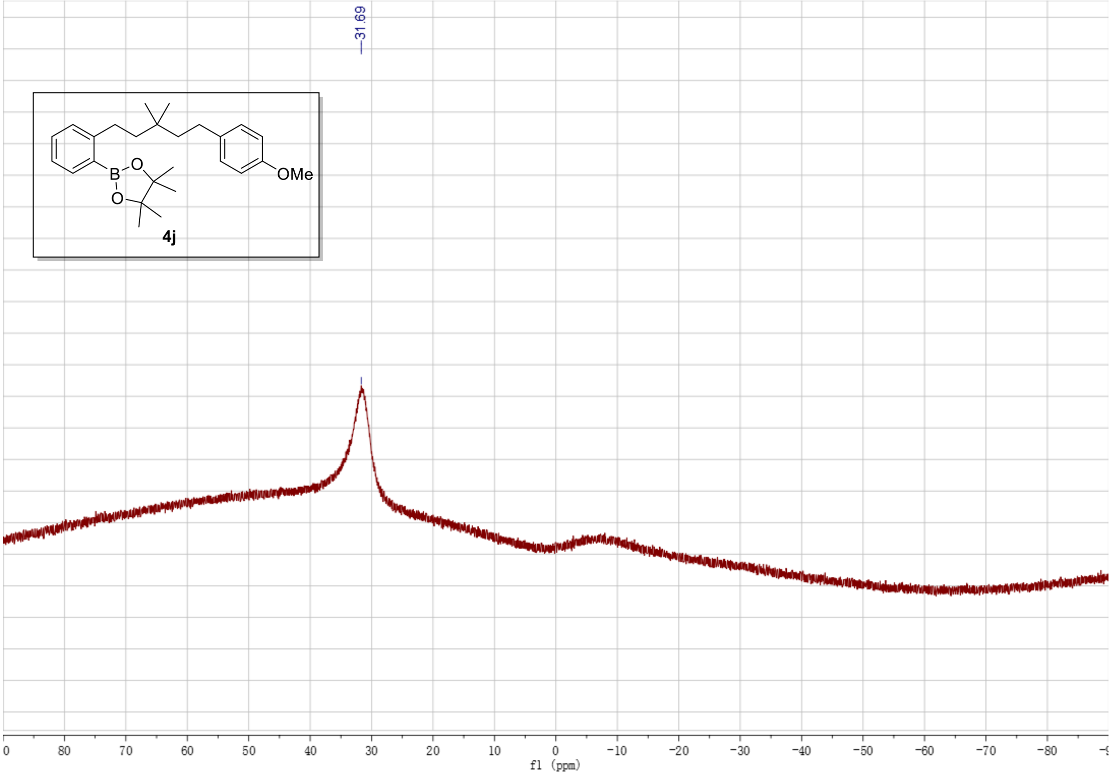

Supplementary Figure 87. ^{11}B spectrum of **4j**.

2-(2-(5-(4-methoxyphenoxy)-3,3-dimethylpentyl)phenyl)-4,4,5,5-tetramethyl-1,3,2-dioxaborolane (4k)

^{1}H spectrum (500 MHz, room temperature, $CDCl_3$) of (**4k**)

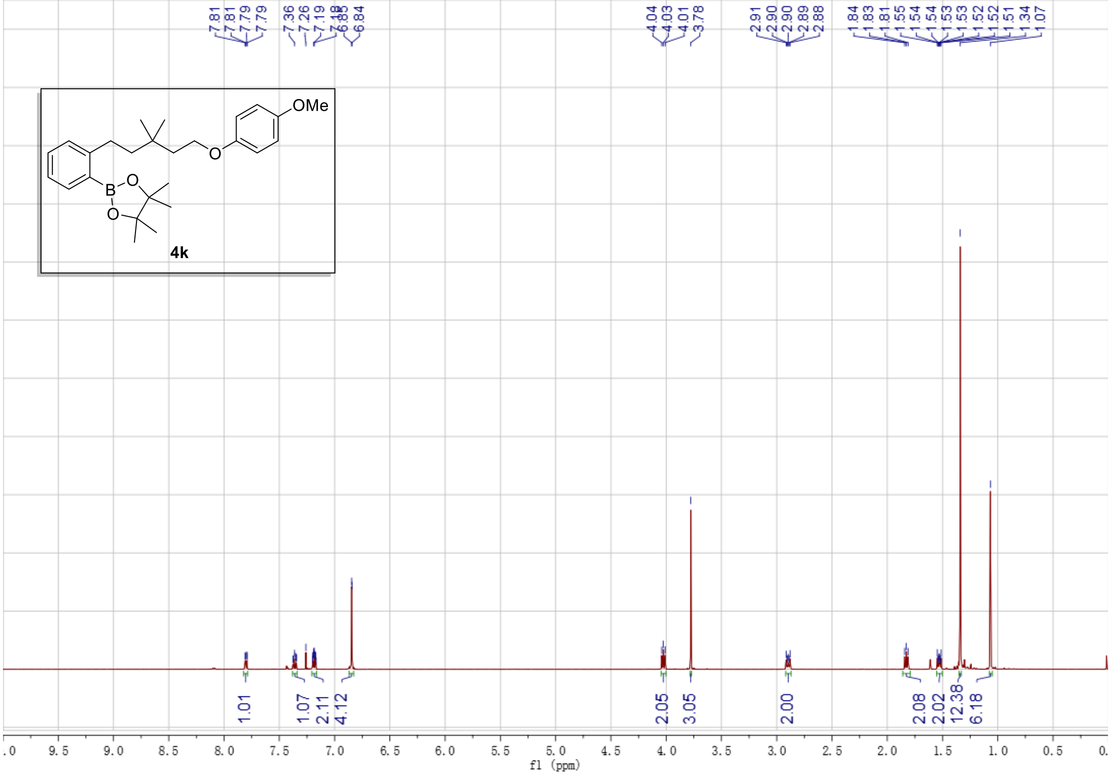

Supplementary Figure 88. ^{1}H NMR spectrum of **4k**.

^{13}C spectrum (126 MHz, room temperature, $CDCl_3$) of (**4k**)

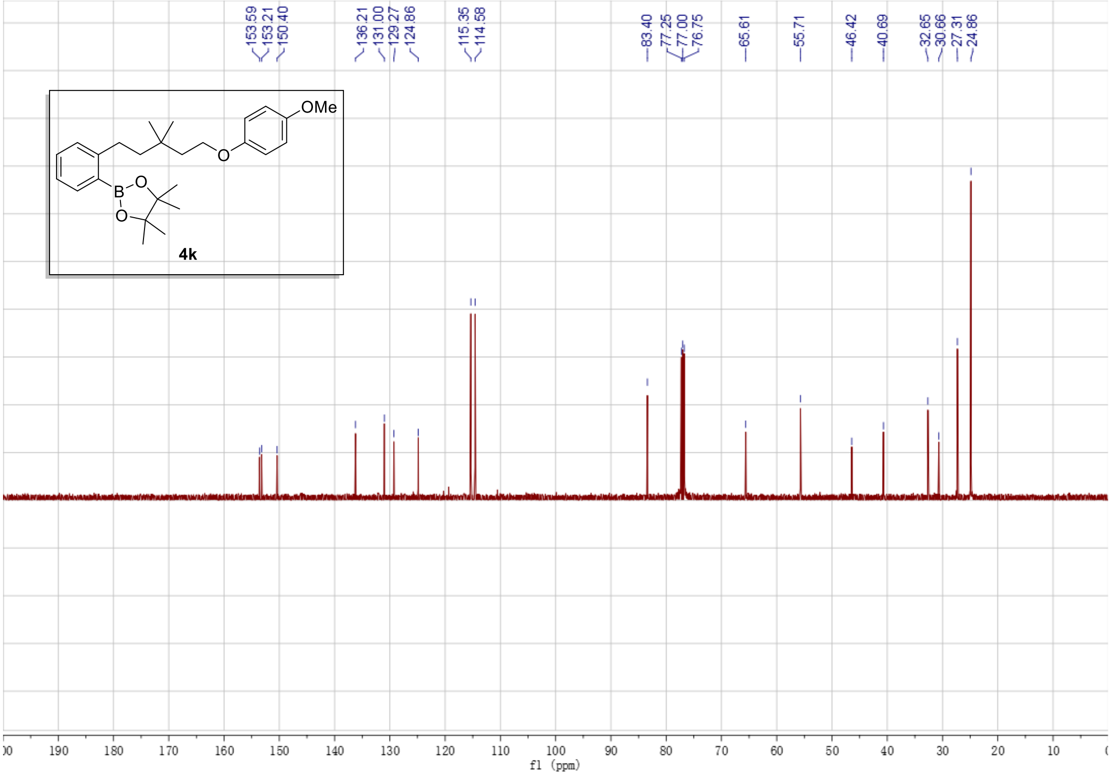

Supplementary Figure 89. ^{13}C NMR spectrum of **4k**.

^{11}B spectrum (128 MHz, room temperature, $CDCl_3$) of (**4k**)

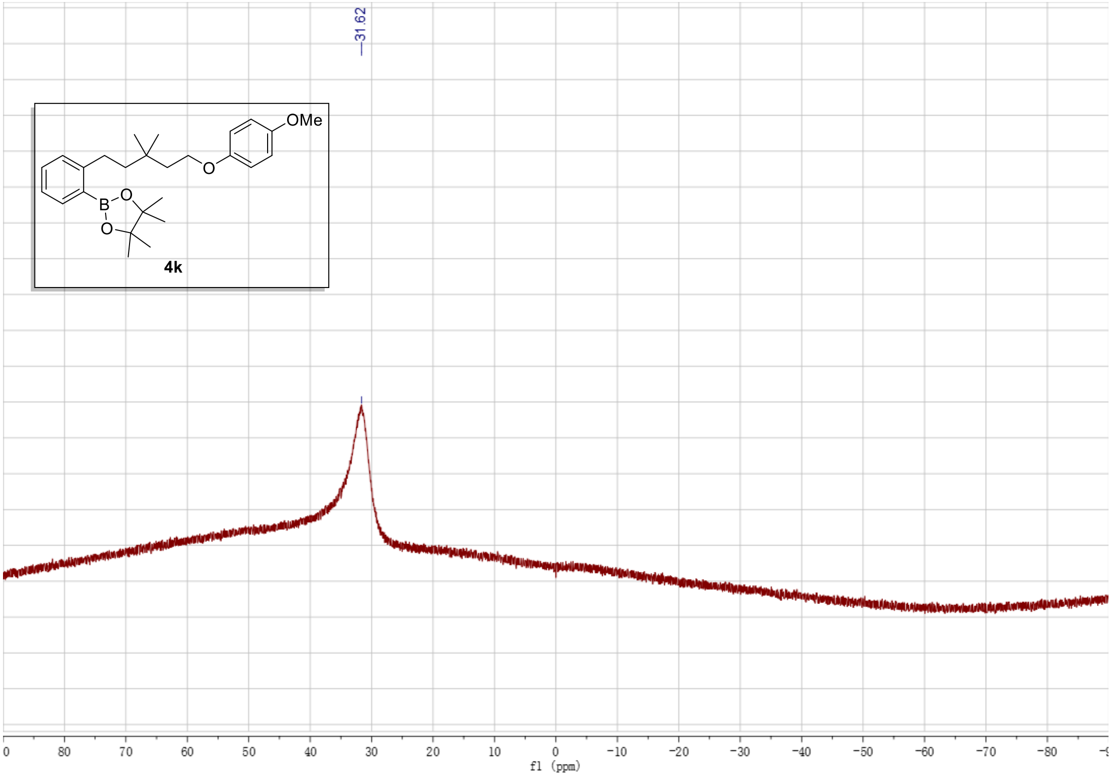

Supplementary Figure 90. ^{11}B spectrum of **4k**.

2-(2-(5-(4-fluorophenoxy)-3,3-dimethylpentyl)phenyl)-4,4,5,5-tetramethyl-1,3,2-dioxaborolane (4l)

^{1}H spectrum (500 MHz, room temperature, $CDCl_3$) of (**4l**)

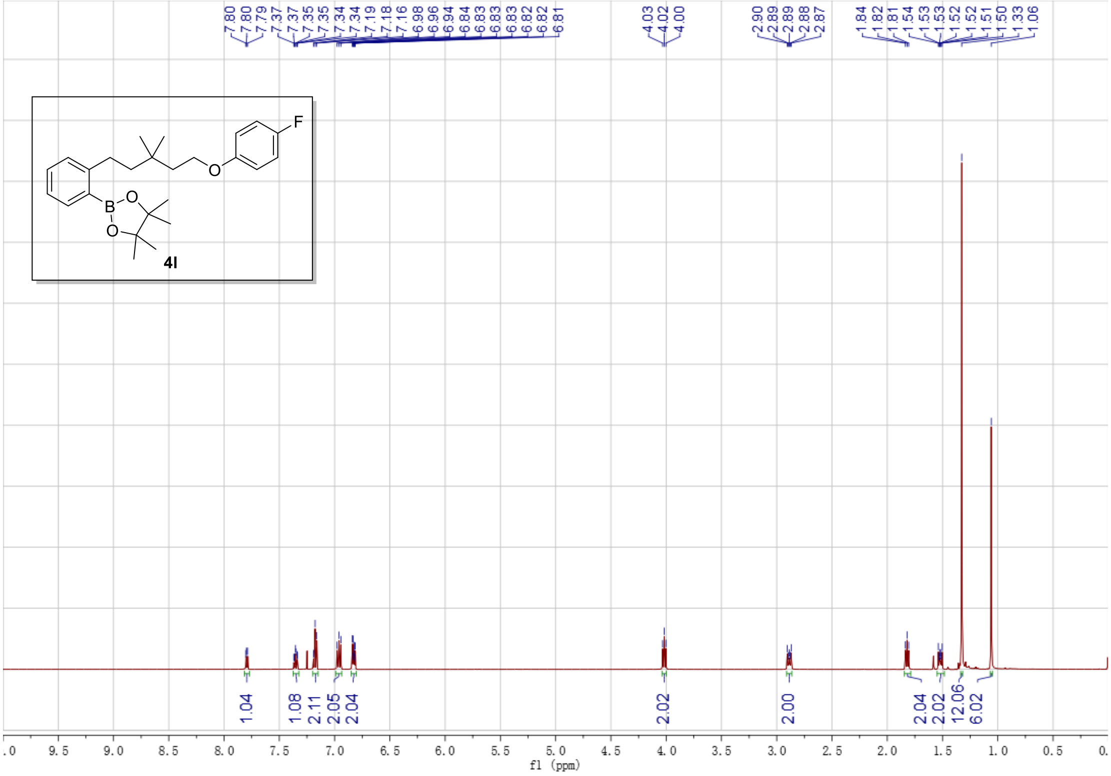

Supplementary Figure 91. ^{1}H NMR spectrum of **4l**.

^{13}C spectrum (126 MHz, room temperature, $CDCl_3$) of (**4l**)

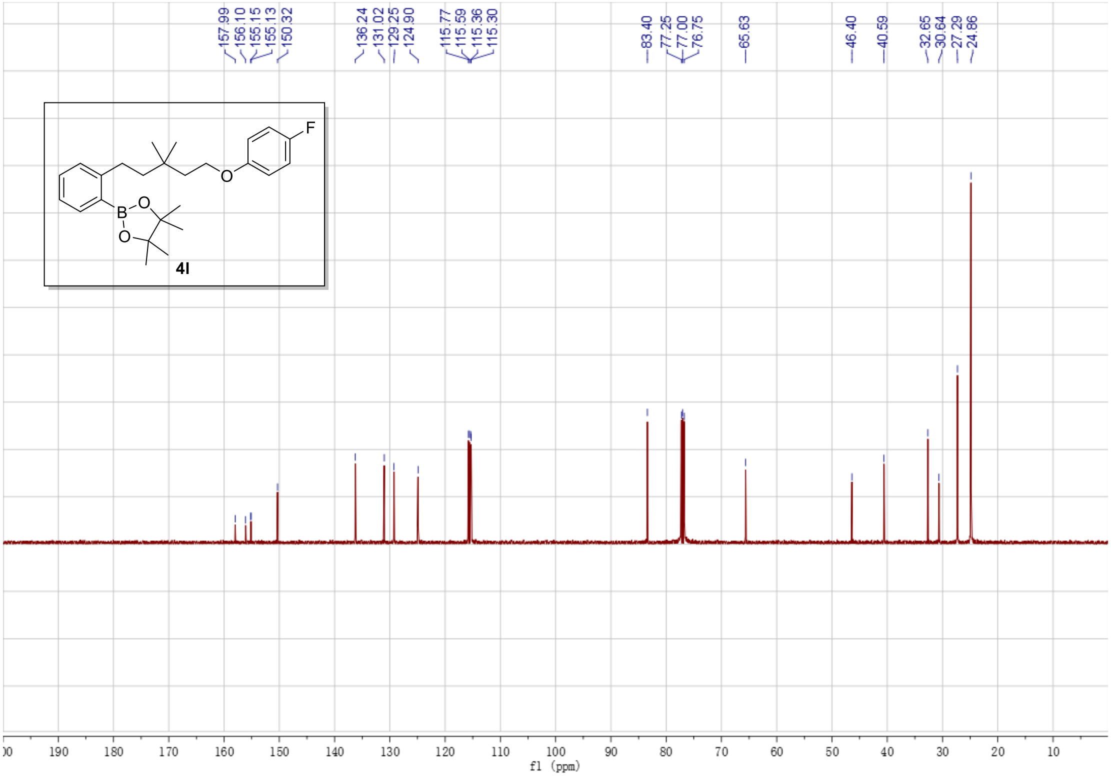

Supplementary Figure 92. ^{13}C NMR spectrum of **4l**.

^{11}B spectrum (128 MHz, room temperature, $CDCl_3$) of (**4l**)

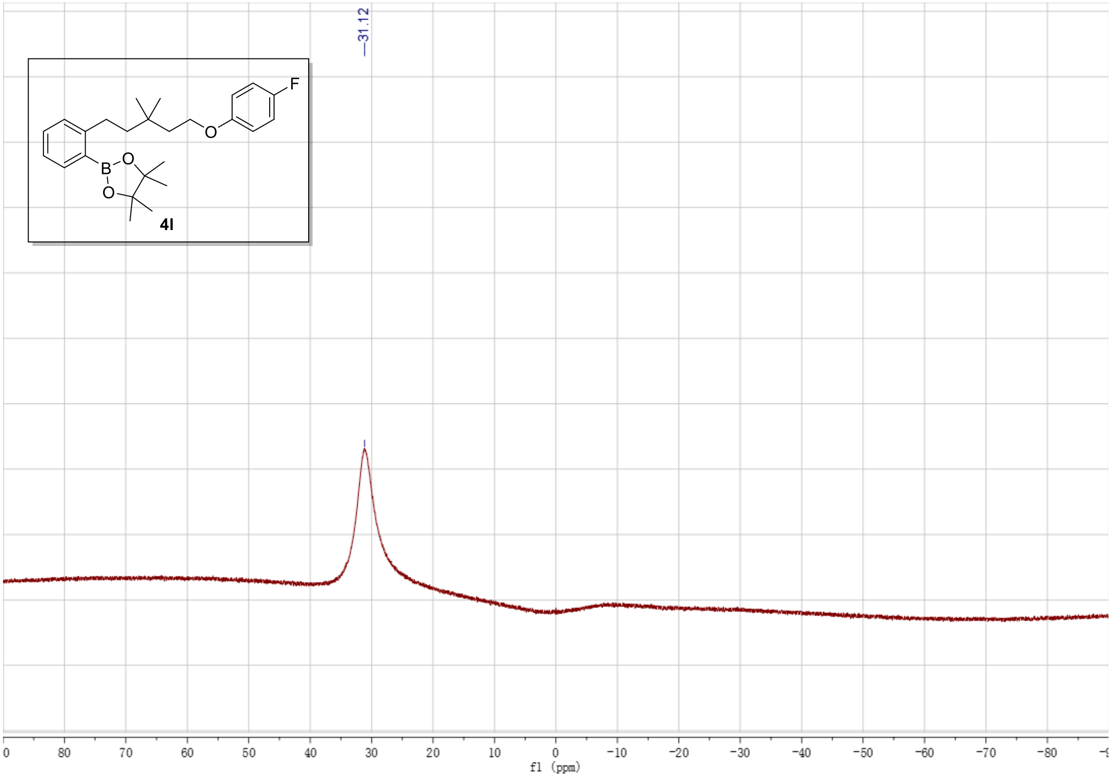

Supplementary Figure 93. ^{11}B spectrum of **4l**.

^{19}F spectrum (471 MHz, room temperature, $CDCl_3$) of (**4l**)

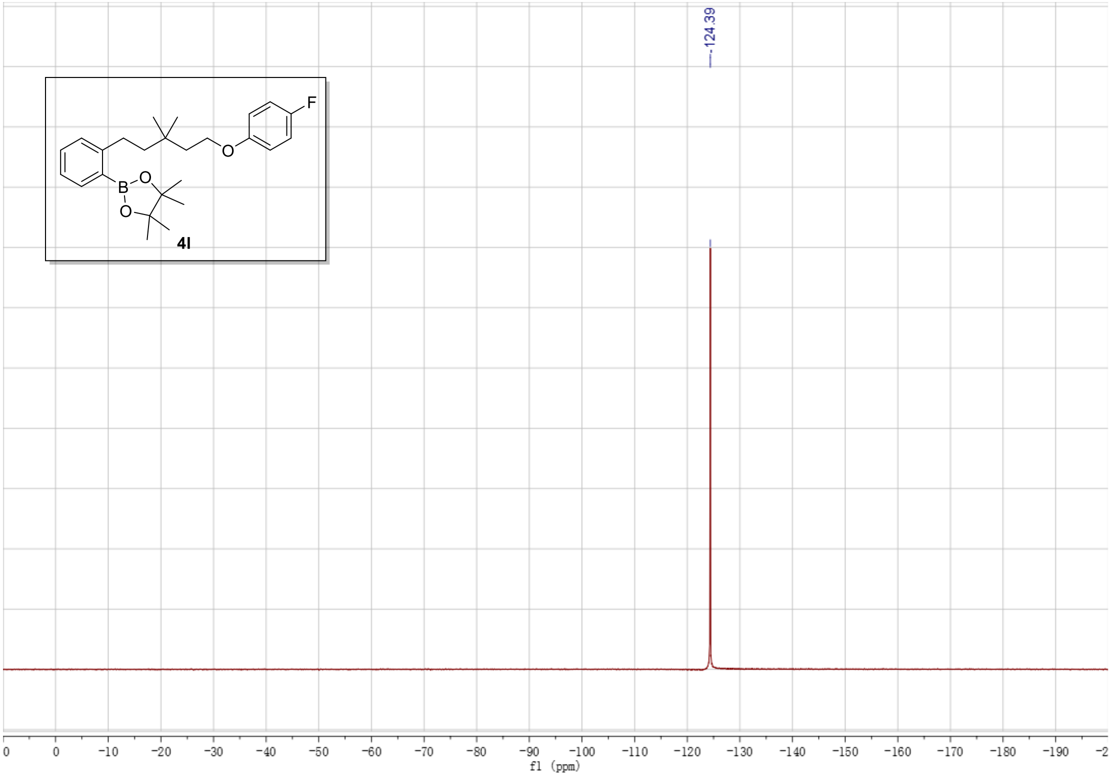

Supplementary Figure 94. ^{19}F spectrum of **4l**.

2-(2-(3,3-dimethyl-5-(4-(trifluoromethyl)phenoxy)pentyl)phenyl)-4,4,5,5-tetramethyl-1,3,2-dioxaborolane (4m)

^{1}H spectrum (500 MHz, room temperature, $CDCl_3$) of (**4m**)

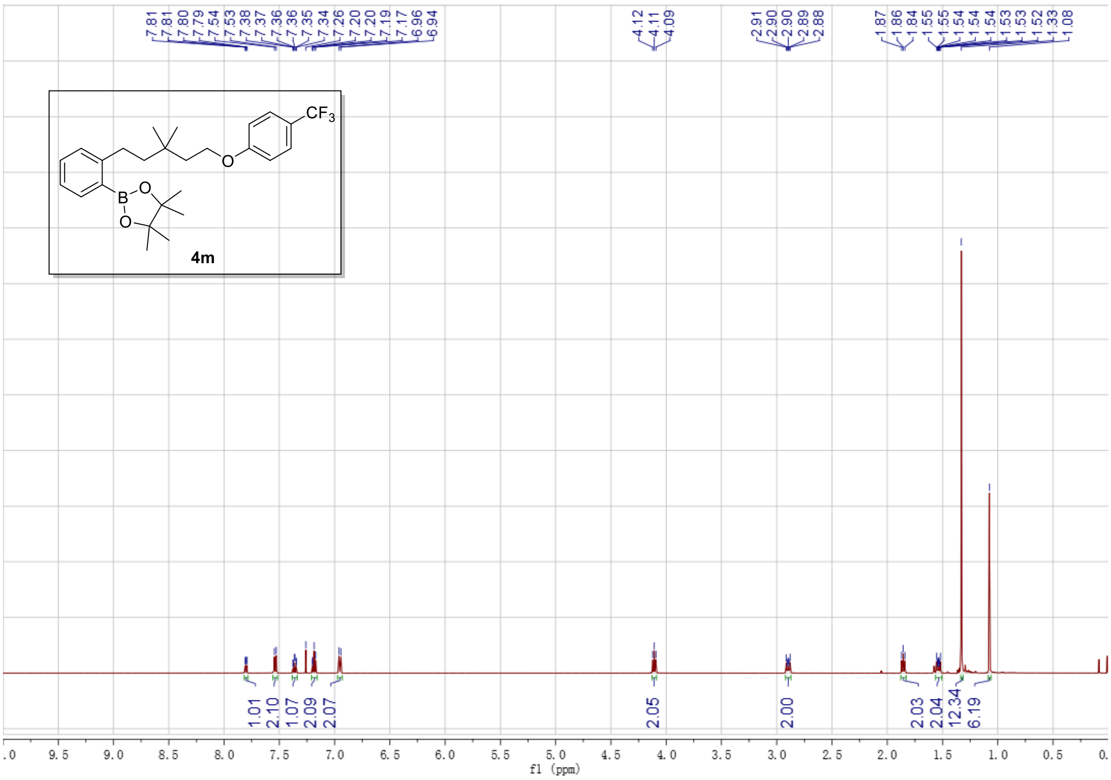

Supplementary Figure 95. ^{1}H NMR spectrum of **4m**.

^{13}C spectrum (126 MHz, room temperature, $CDCl_3$) of (**4m**)

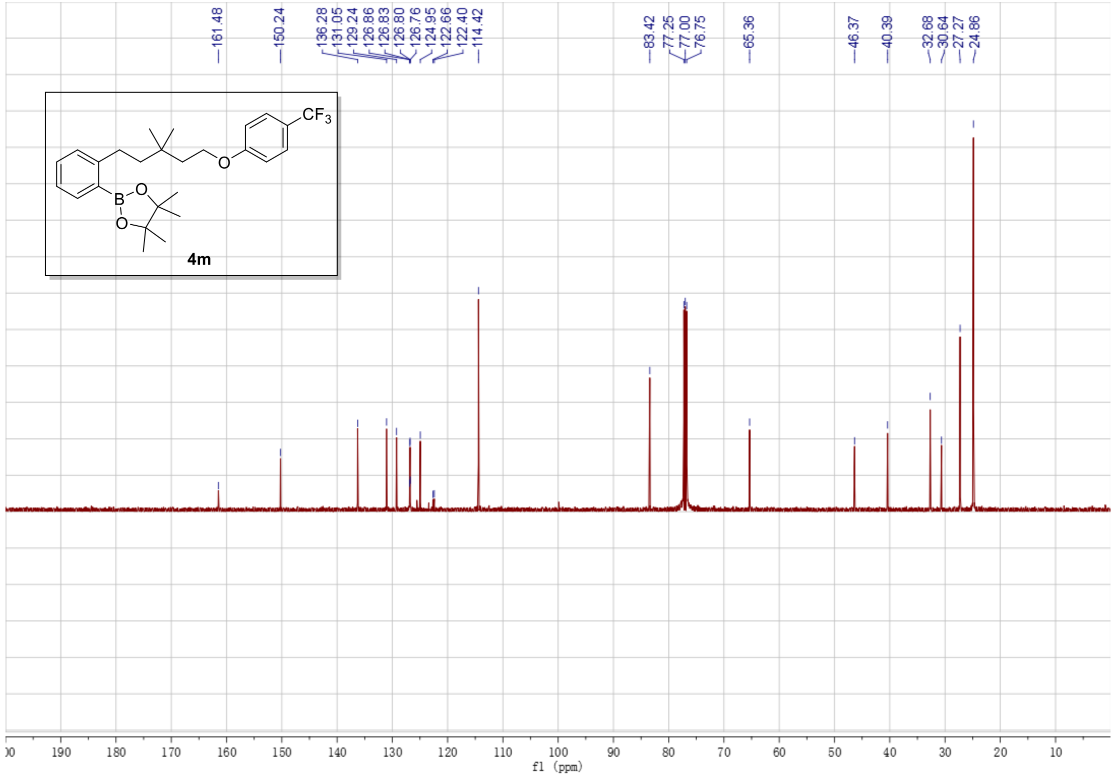

Supplementary Figure 96. ^{13}C NMR spectrum of **4m**.

^{11}B spectrum (128 MHz, room temperature, $CDCl_3$) of (**4m**)

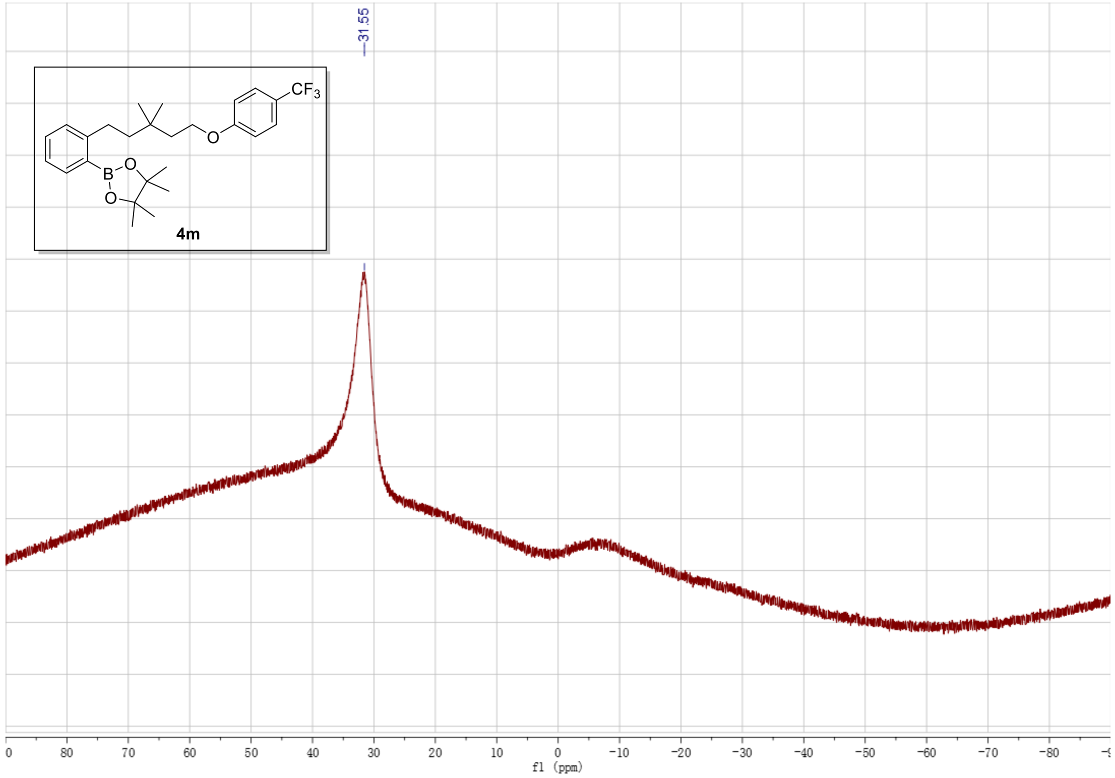

Supplementary Figure 97. ^{11}B spectrum of **4m**.

^{19}F spectrum (471 MHz, room temperature, $CDCl_3$) of (**4m**)

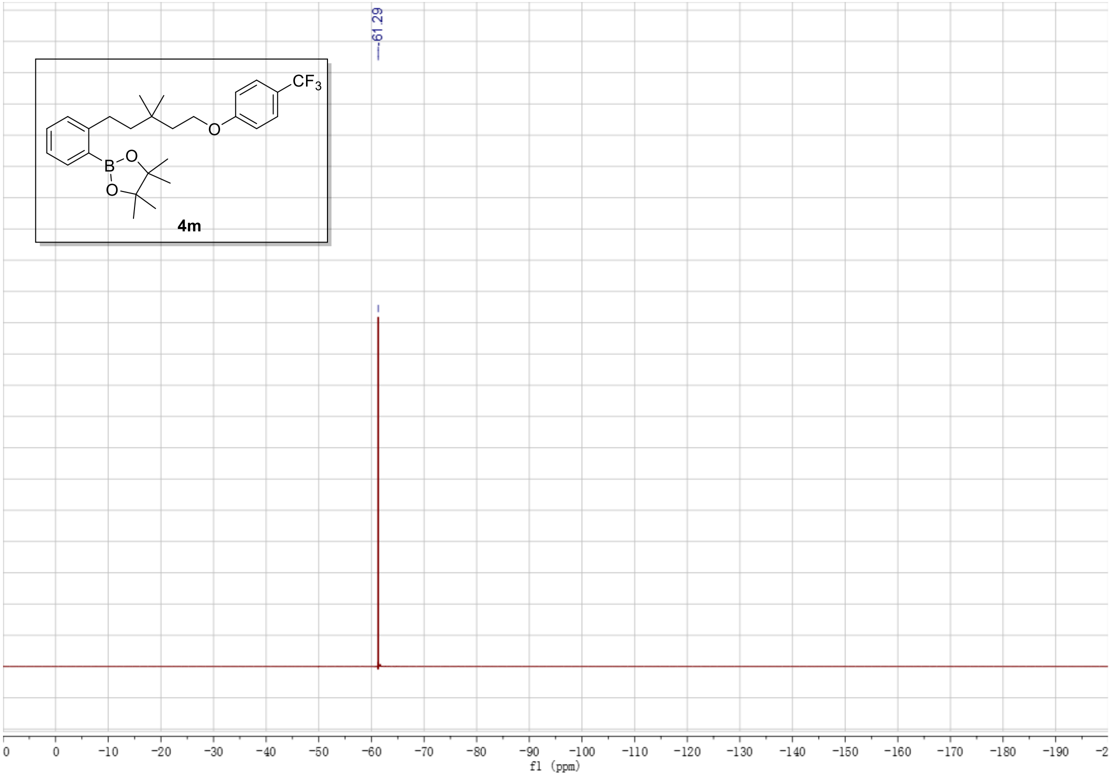

Supplementary Figure 98. ^{19}F spectrum of **4m**.

2-(2-(3,3-dimethyl-5-(4-(trifluoromethoxy)phenoxy)pentyl)phenyl)-4,4,5,5-tetramethyl-1,3,2-dioxaborolane (4n)

^{1}H spectrum (500 MHz, room temperature, $CDCl_3$) of (**4n**)

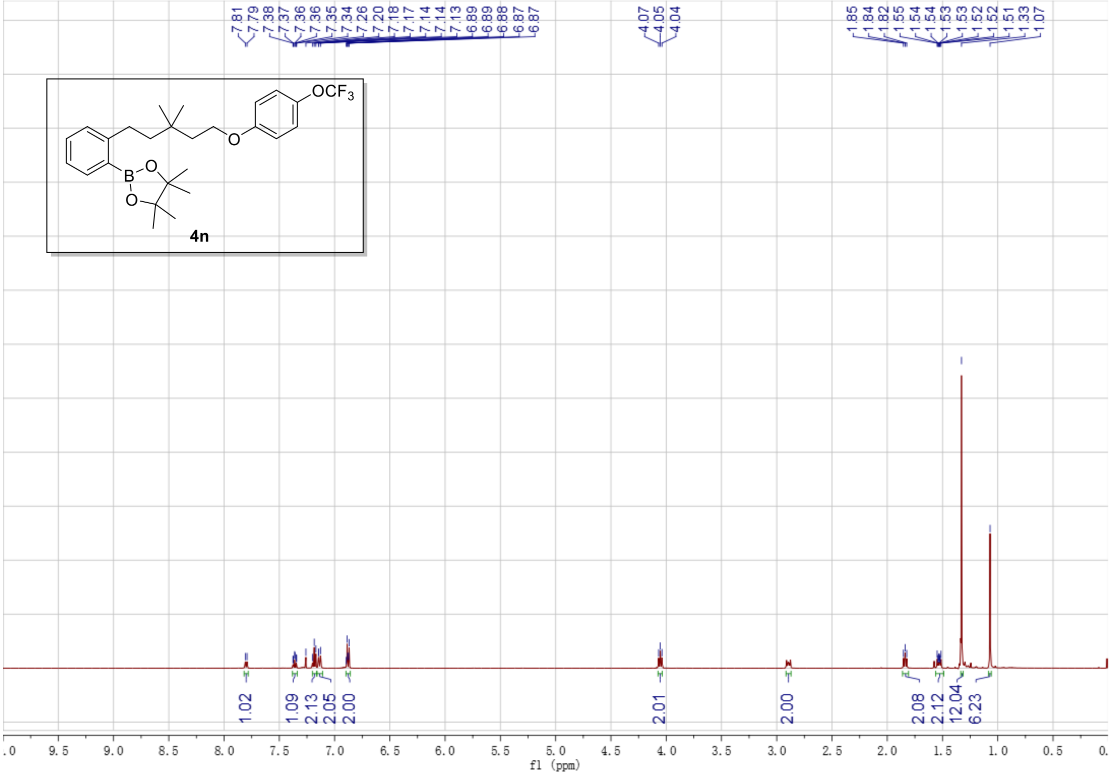

Supplementary Figure 99. ^{1}H NMR spectrum of **4n**.

^{13}C spectrum (126 MHz, room temperature, $CDCl_3$) of (**4n**)

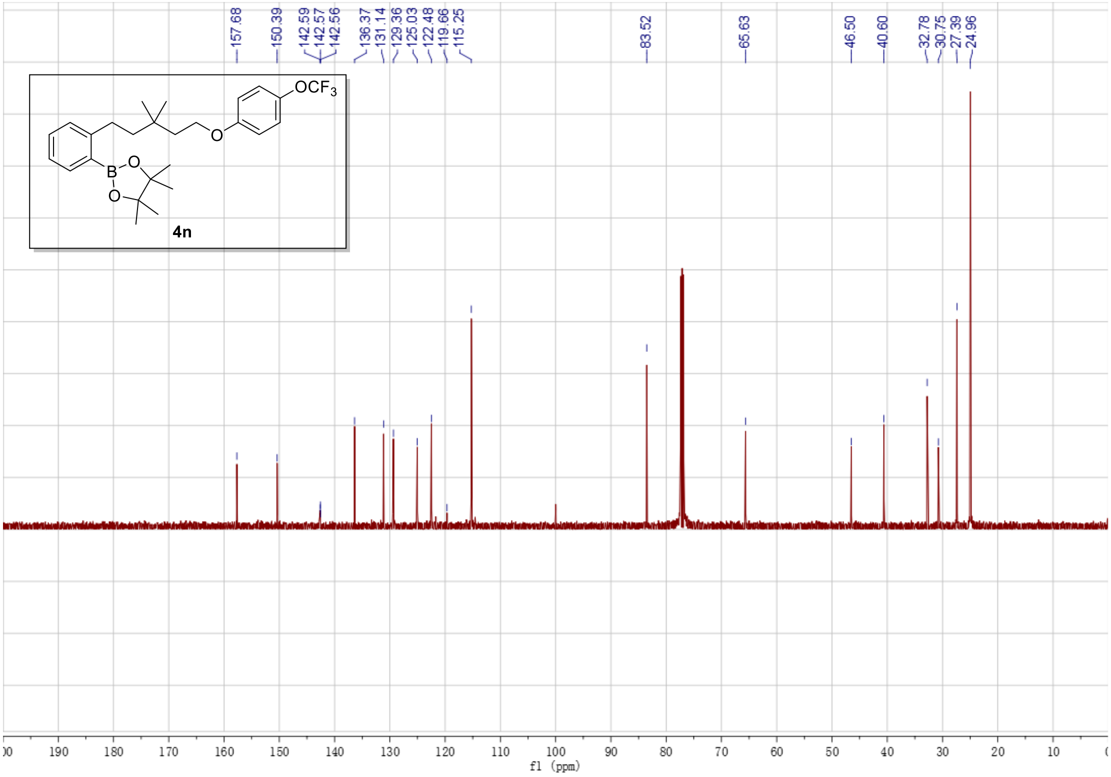

Supplementary Figure 100. ^{13}C NMR spectrum of **4n**.

^{11}B spectrum (128 MHz, room temperature, $CDCl_3$) of (**4n**)

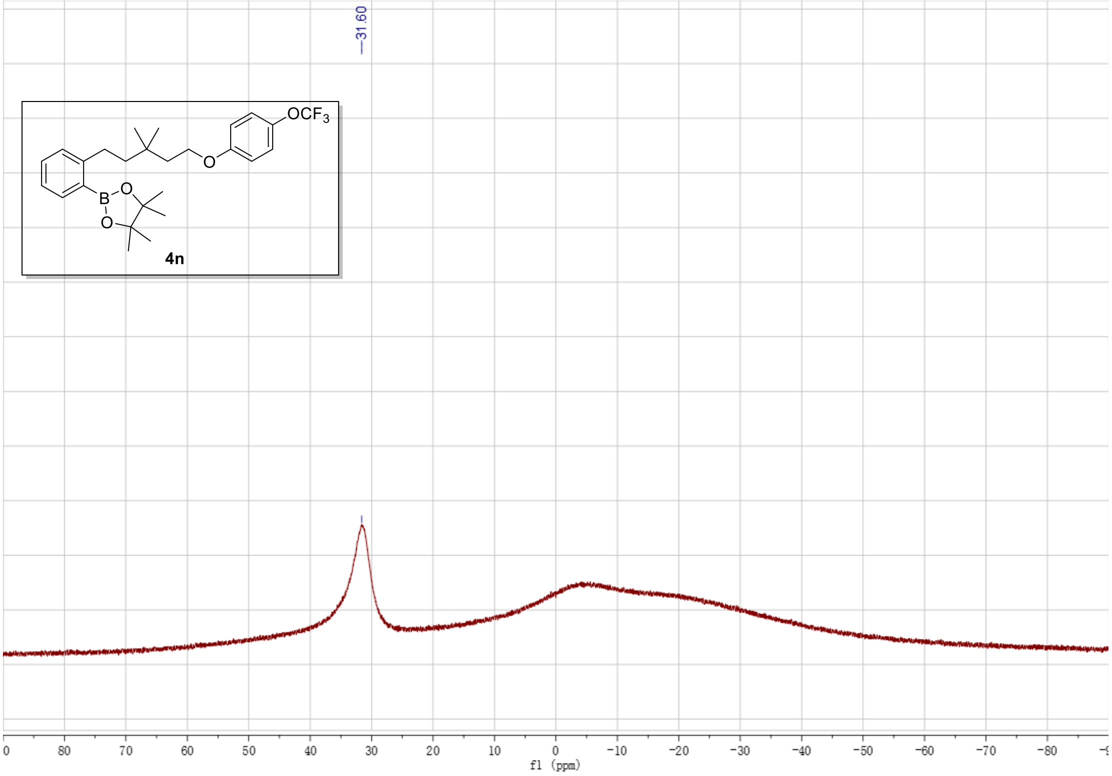

Supplementary Figure 101. ^{11}B spectrum of **4n**.

^{19}F spectrum (471 MHz, room temperature, $CDCl_3$) of (**4n**)

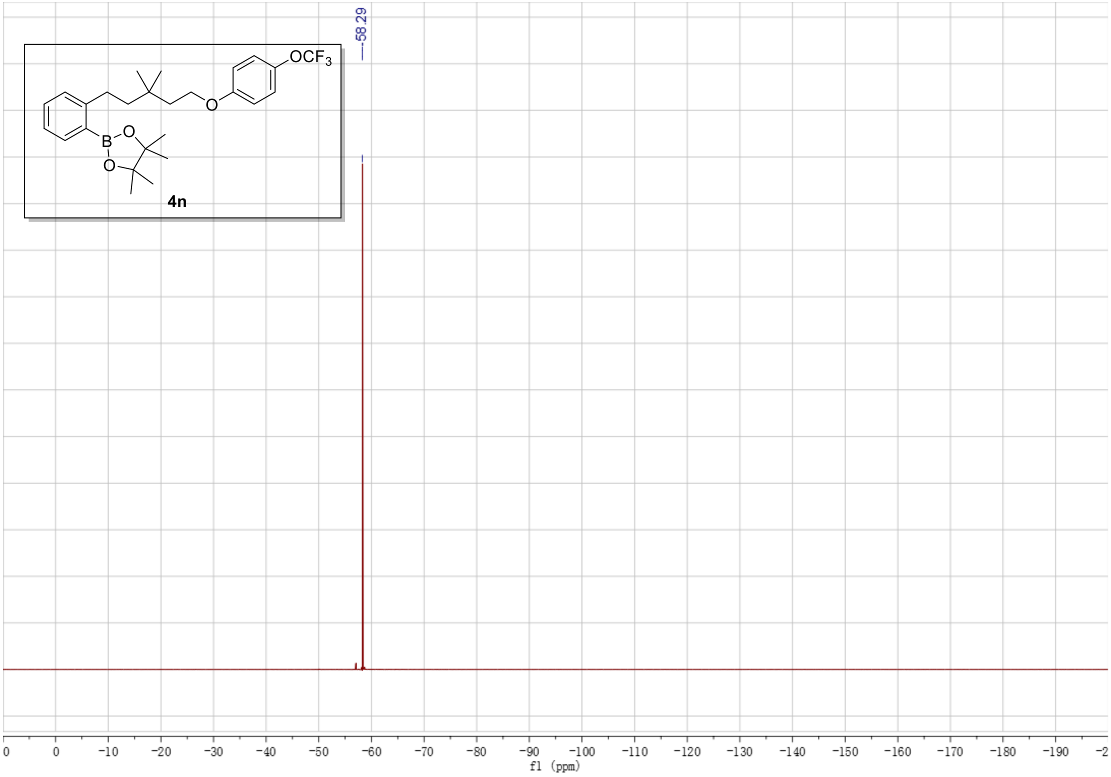

Supplementary Figure 102. ^{19}F spectrum of **4n**.

2-(2-(3,3-dimethylbutyl)-5-methylphenyl)-4,4,5,5-tetramethyl-1,3,2-dioxaborolane (4o)

^{1}H spectrum (400 MHz, room temperature, $CDCl_3$) of (**4o**)

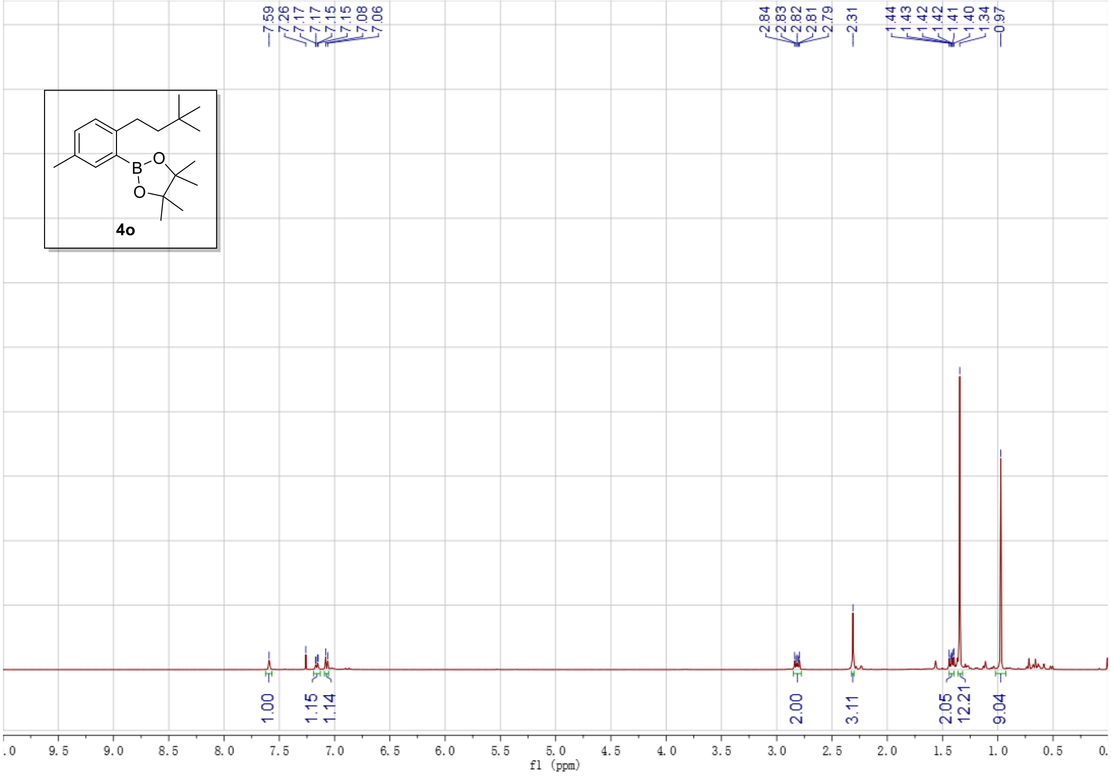

Supplementary Figure 103. ^{1}H NMR spectrum of **4o**.

^{13}C spectrum (126 MHz, room temperature, $CDCl_3$) of (**4o**)

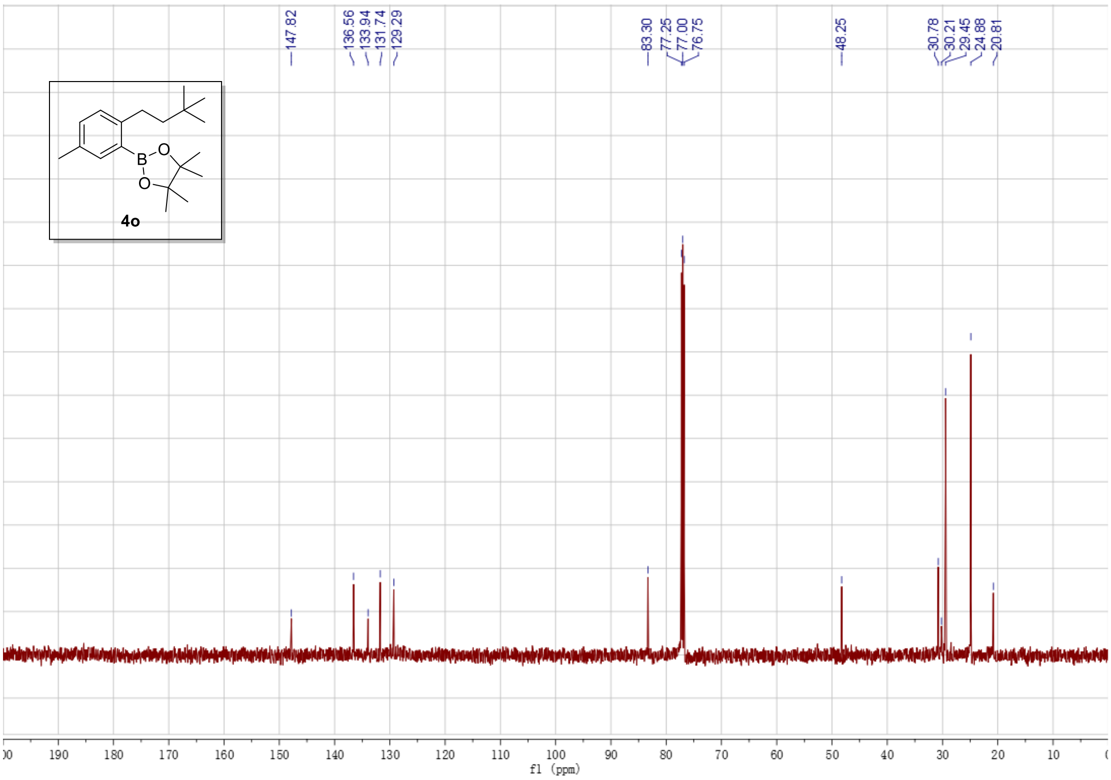

Supplementary Figure 104. ^{13}C NMR spectrum of **4o**.

^{11}B spectrum (128 MHz, room temperature, $CDCl_3$) of (**4o**)

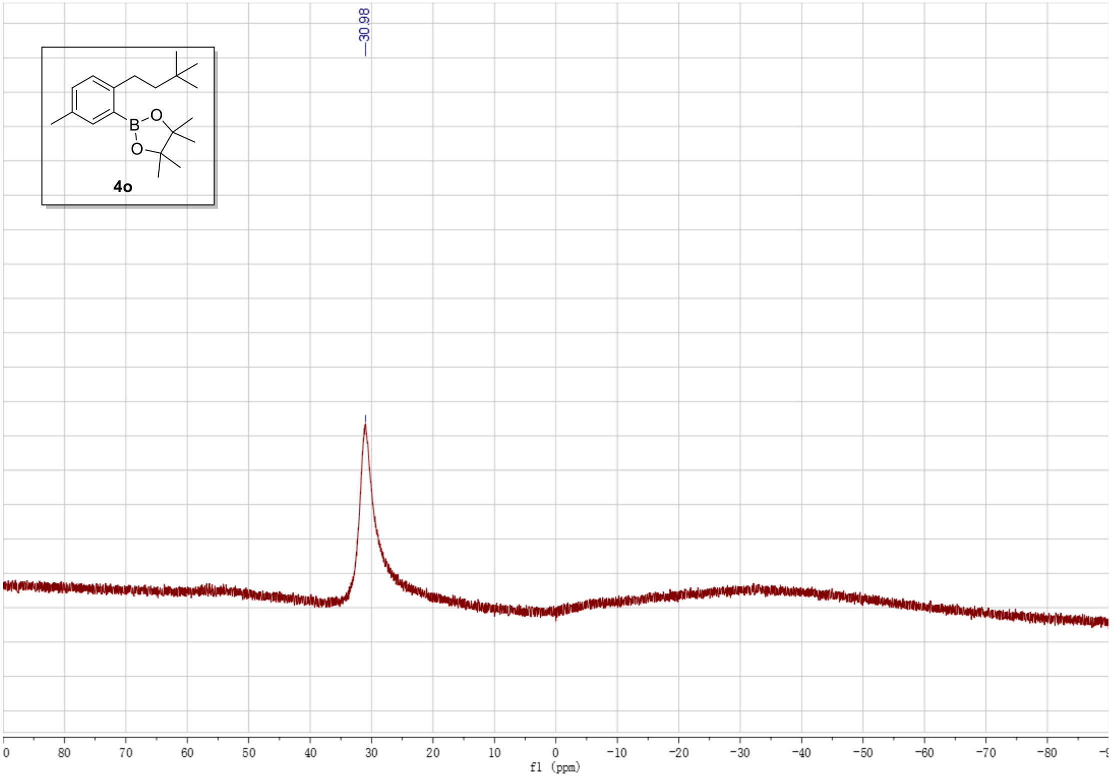

Supplementary Figure 105. ^{11}B spectrum of **4o**.

2-(2-(3,3-dimethylbutyl)-4-methoxyphenyl)-4,4,5,5-tetramethyl-1,3,2-dioxaborolane (4p)

^{1}H spectrum (500 MHz, room temperature, $CDCl_3$) of (**4p**)

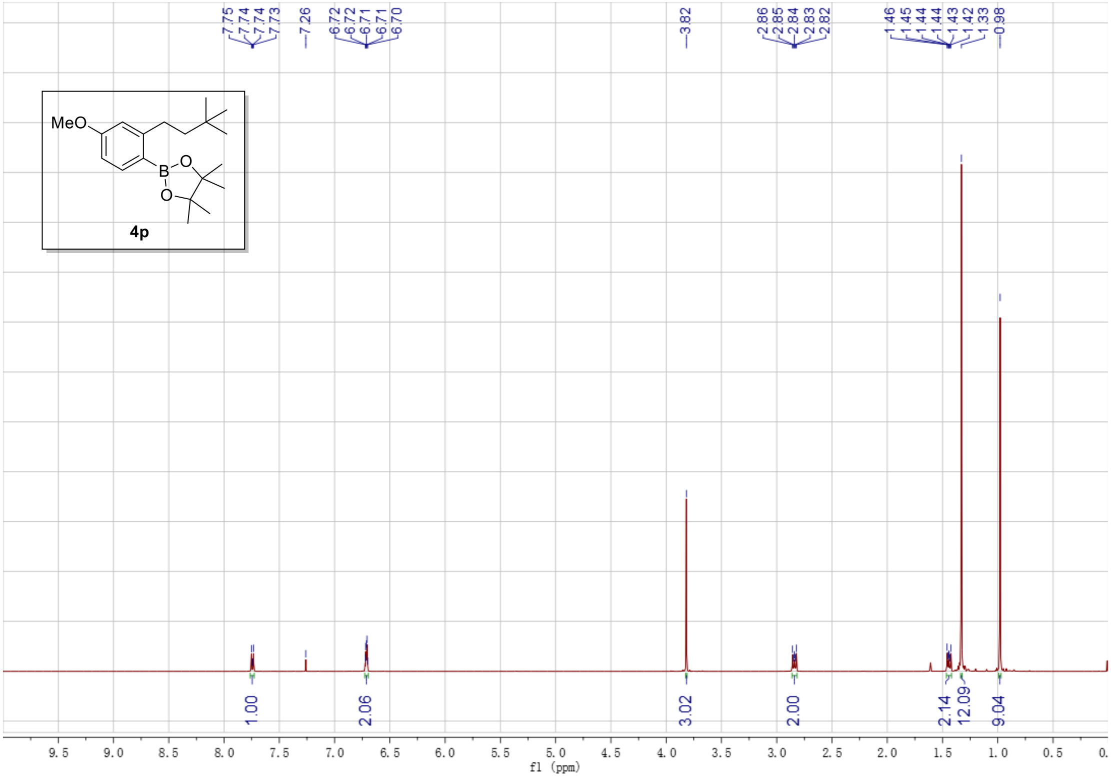

Supplementary Figure 106. ^{1}H NMR spectrum of **4p**.

^{13}C spectrum (126 MHz, room temperature, $CDCl_3$) of (**4p**)

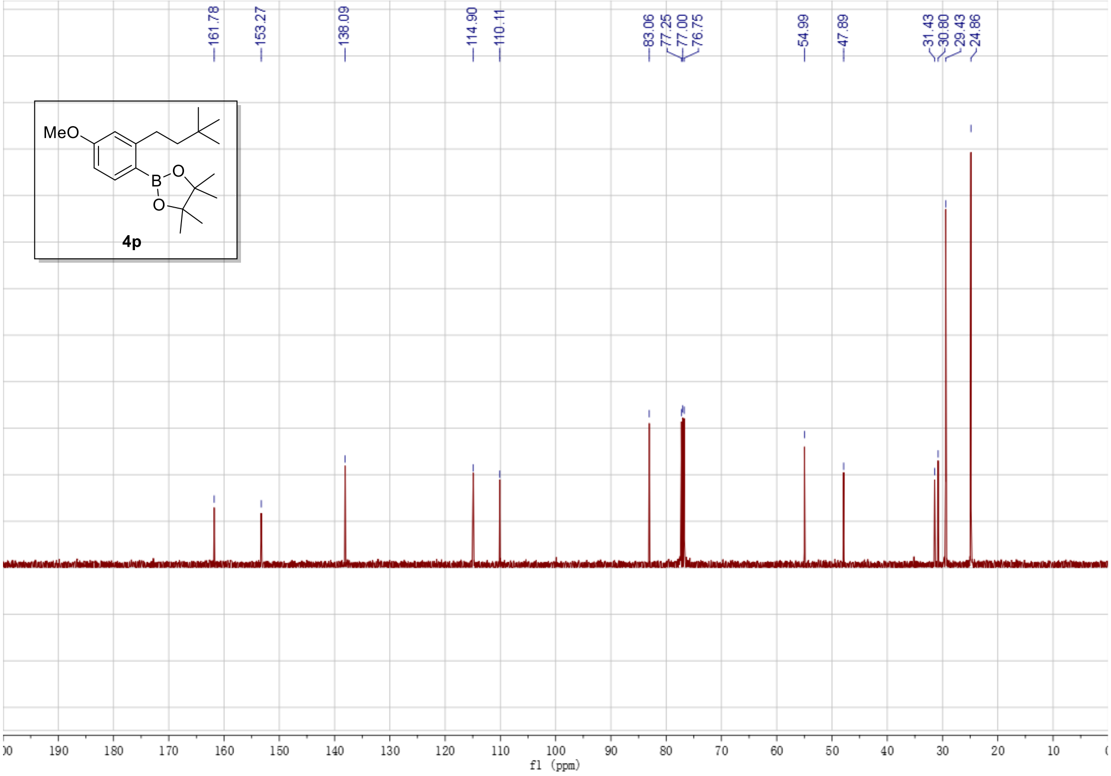

Supplementary Figure 107. ^{13}C NMR spectrum of **4p**.

^{11}B spectrum (160 MHz, room temperature, $CDCl_3$) of (**4p**)

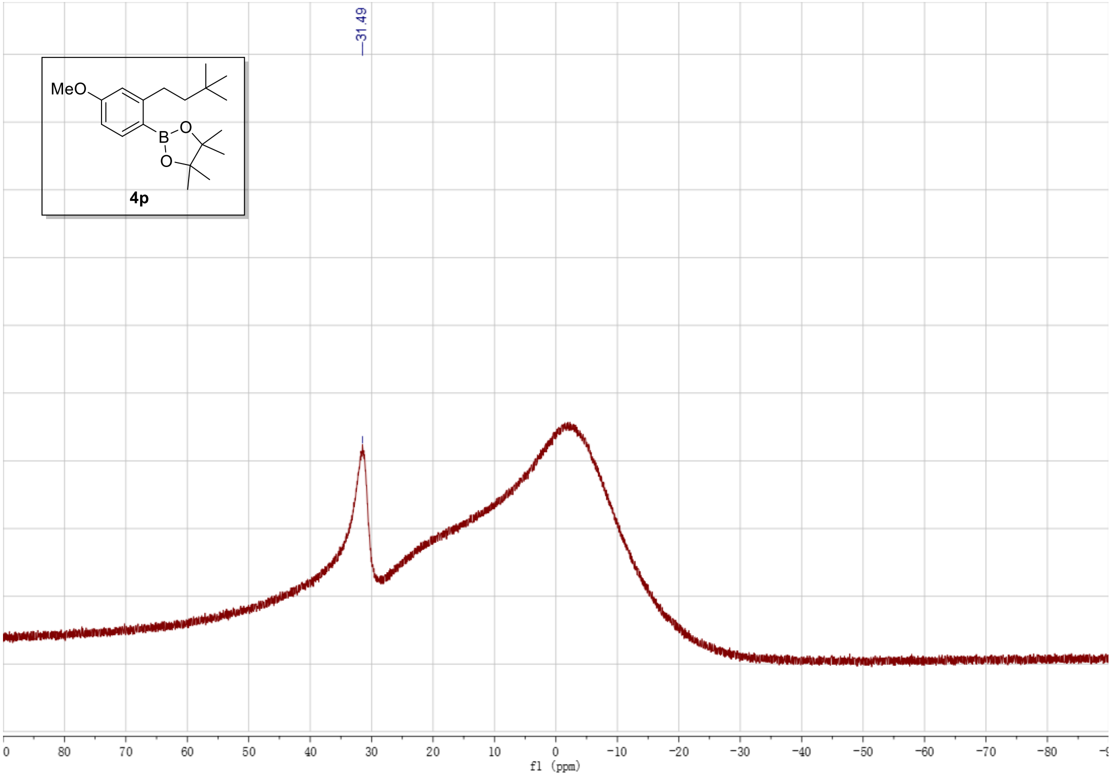

Supplementary Figure 108. ^{11}B spectrum of **4p**.

2-(2-(3,3-dimethylbutyl)-4,5-dimethoxyphenyl)-4,4,5,5-tetramethyl-1,3,2-dioxaborolane (4q)

^{1}H spectrum (400 MHz, room temperature, $CDCl_3$) of (**4q**)

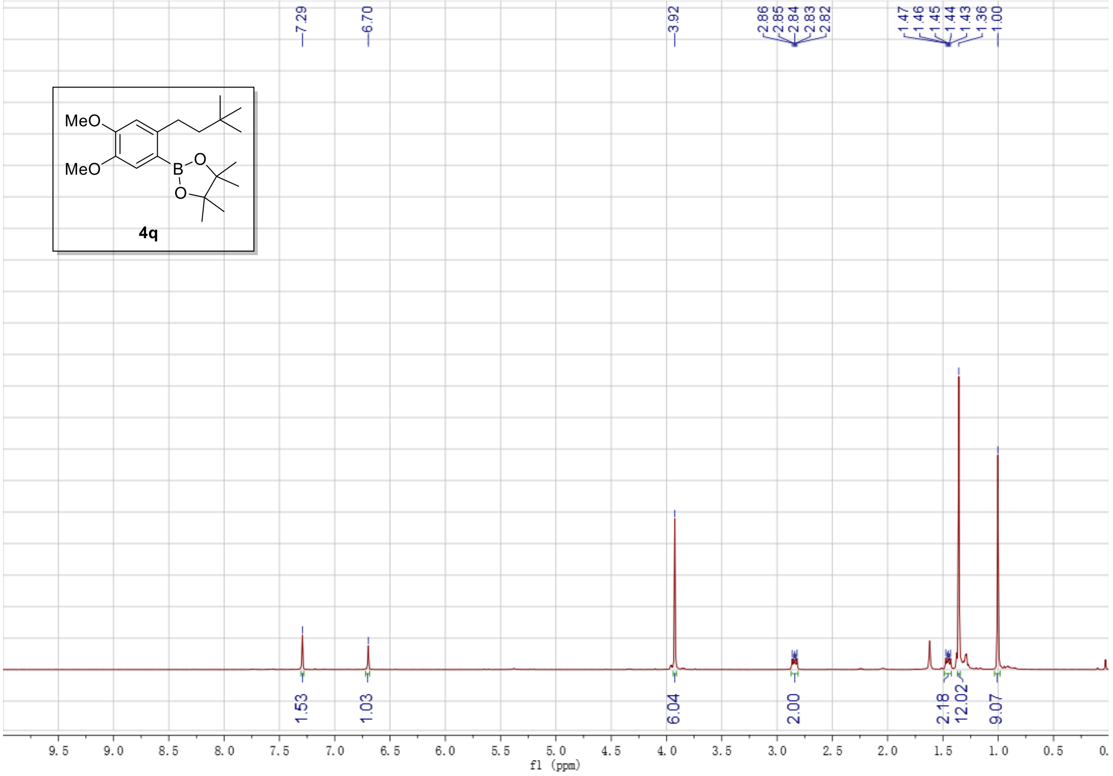

Supplementary Figure 109. ^{1}H NMR spectrum of **4q**.

^{13}C spectrum (101 MHz, room temperature, $CDCl_3$) of (**4q**)

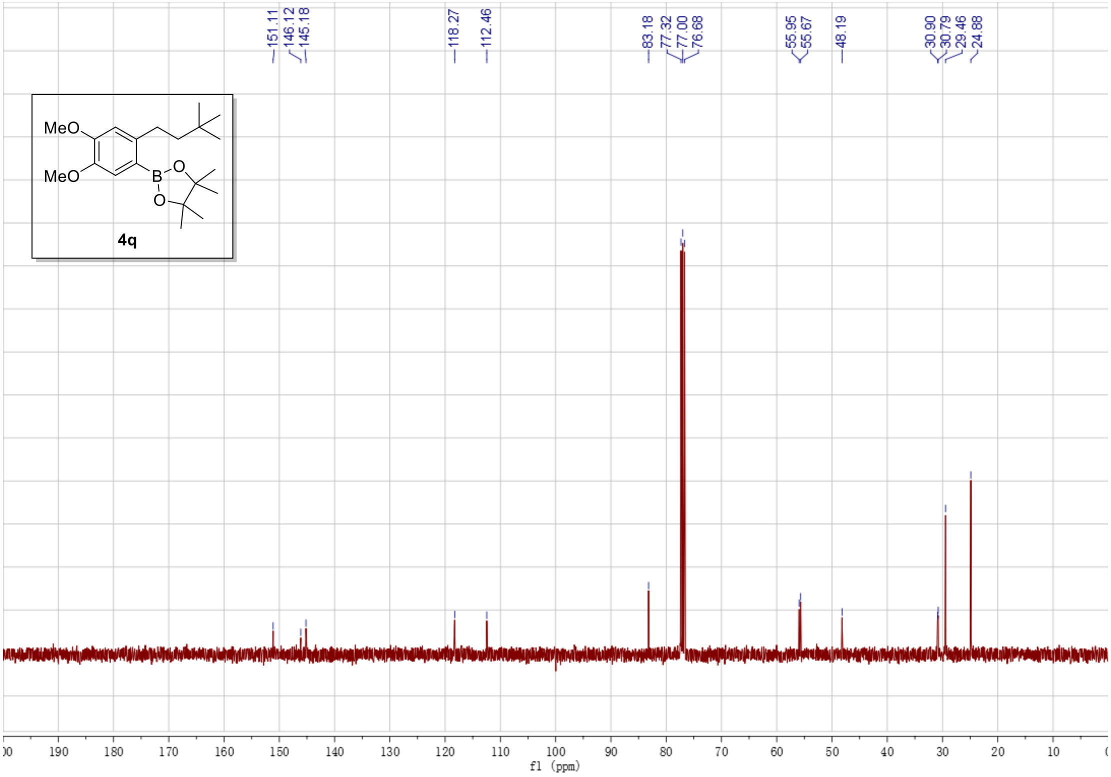

Supplementary Figure 110. ^{13}C NMR spectrum of **4q**.

^{11}B spectrum (128 MHz, room temperature, $CDCl_3$) of (**4q**)

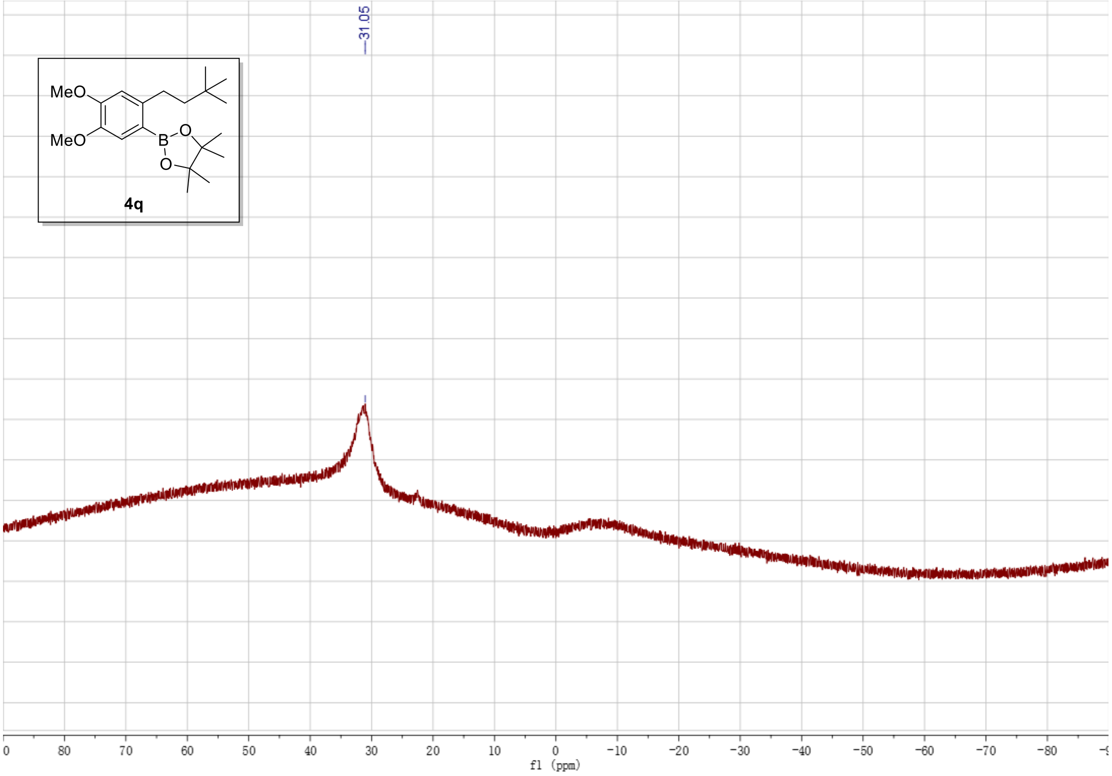

Supplementary Figure 111. ^{11}B spectrum of **4q**.

2-(6-(3,3-dimethylbutyl)benzo[d][1,3]dioxol-5-yl)-4,4,5,5-tetramethyl-1,3,2-dioxaborolane (4r)

^{1}H spectrum (500 MHz, room temperature, $CDCl_3$) of (**4r**)

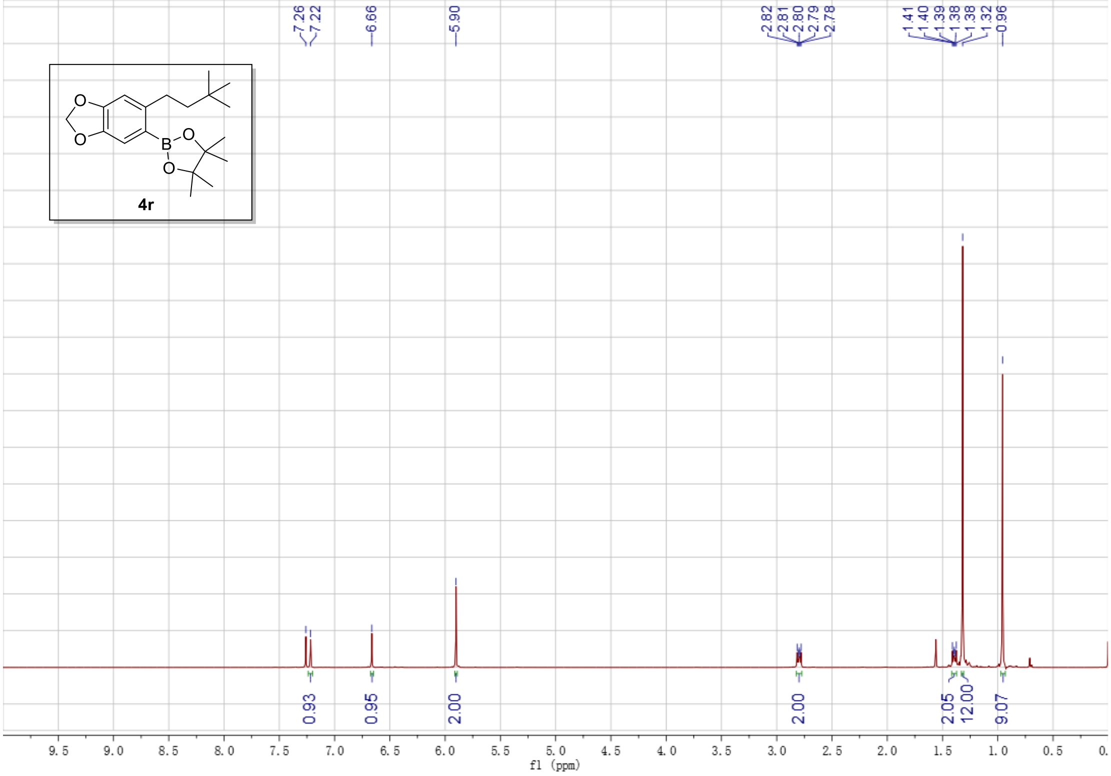

Supplementary Figure 112. ^{1}H NMR spectrum of **4r**.

^{13}C spectrum (126 MHz, room temperature, $CDCl_3$) of (**4r**)

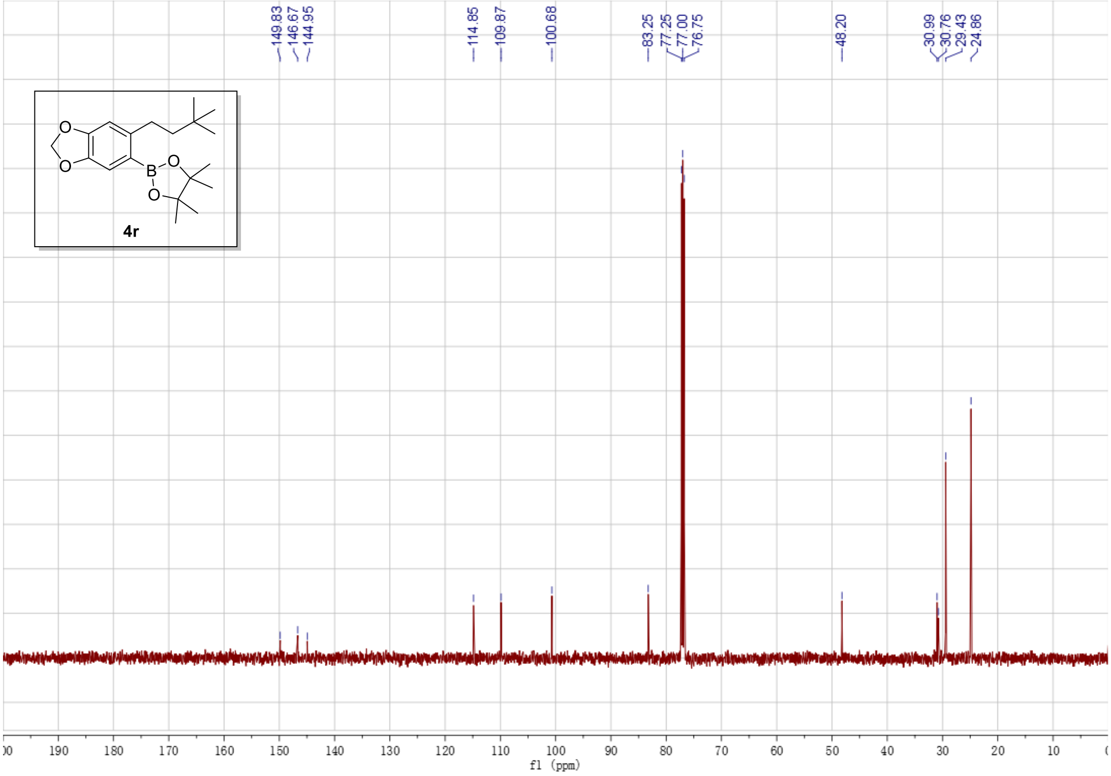

Supplementary Figure 113. ^{13}C NMR spectrum of **4r**.

^{11}B spectrum (128 MHz, room temperature, $CDCl_3$) of (**4r**)

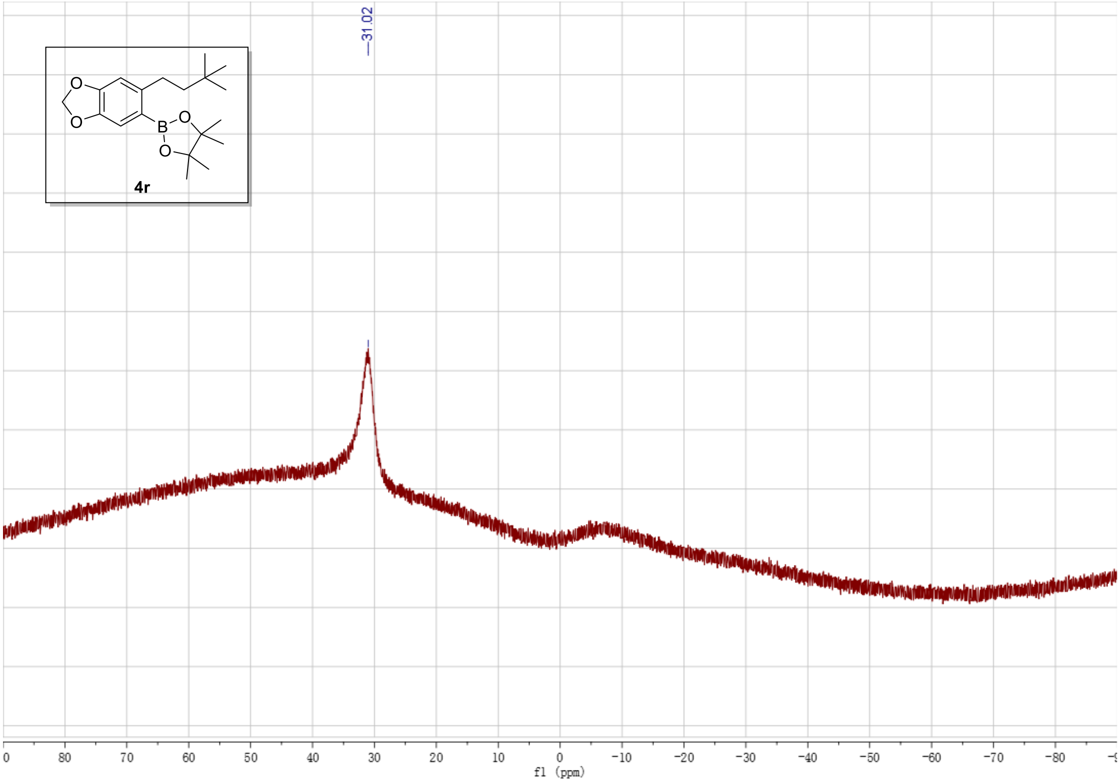

Supplementary Figure 114. ^{11}B spectrum of **4r**.

2-(2-(3,3-dimethylbutyl)-3-fluorophenyl)-4,4,5,5-tetramethyl-1,3,2-dioxaborolane (4s)

^{1}H spectrum (500 MHz, room temperature, $CDCl_3$) of (**4s**)

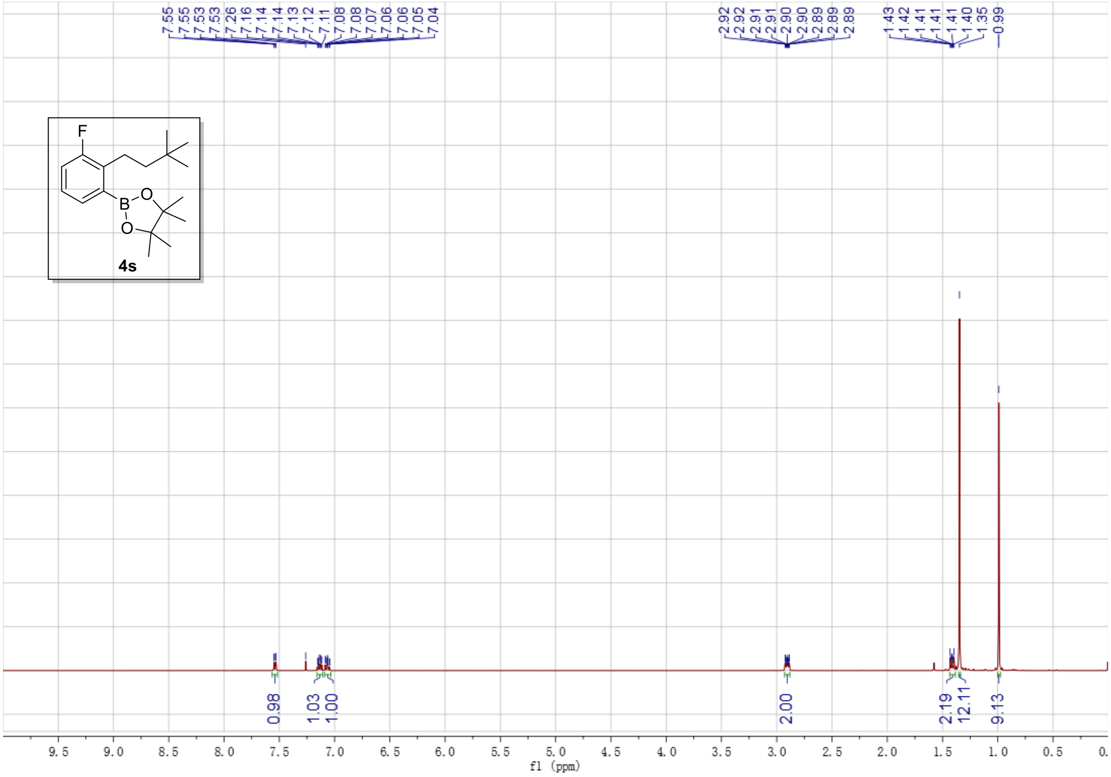

Supplementary Figure 115. ^{1}H NMR spectrum of **4s**.

^{13}C spectrum (126 MHz, room temperature, $CDCl_3$) of (**4s**)

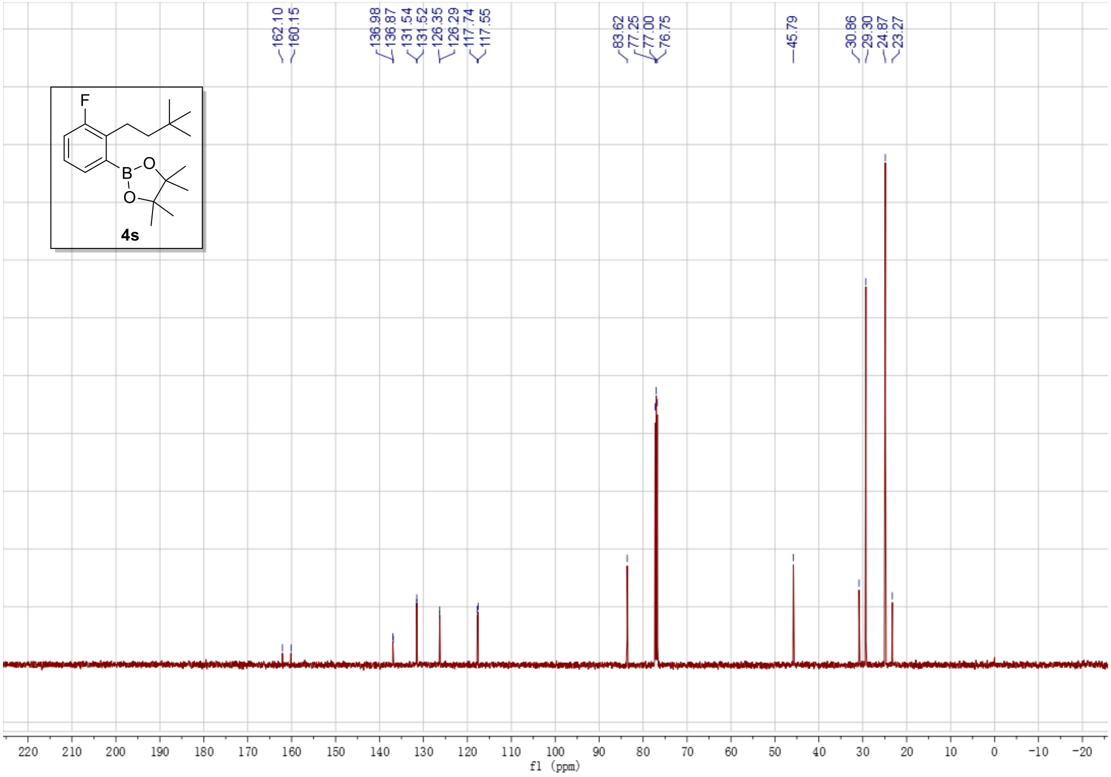

Supplementary Figure 116. ^{13}C NMR spectrum of **4s**.

^{11}B spectrum (128 MHz, room temperature, $CDCl_3$) of (**4s**)

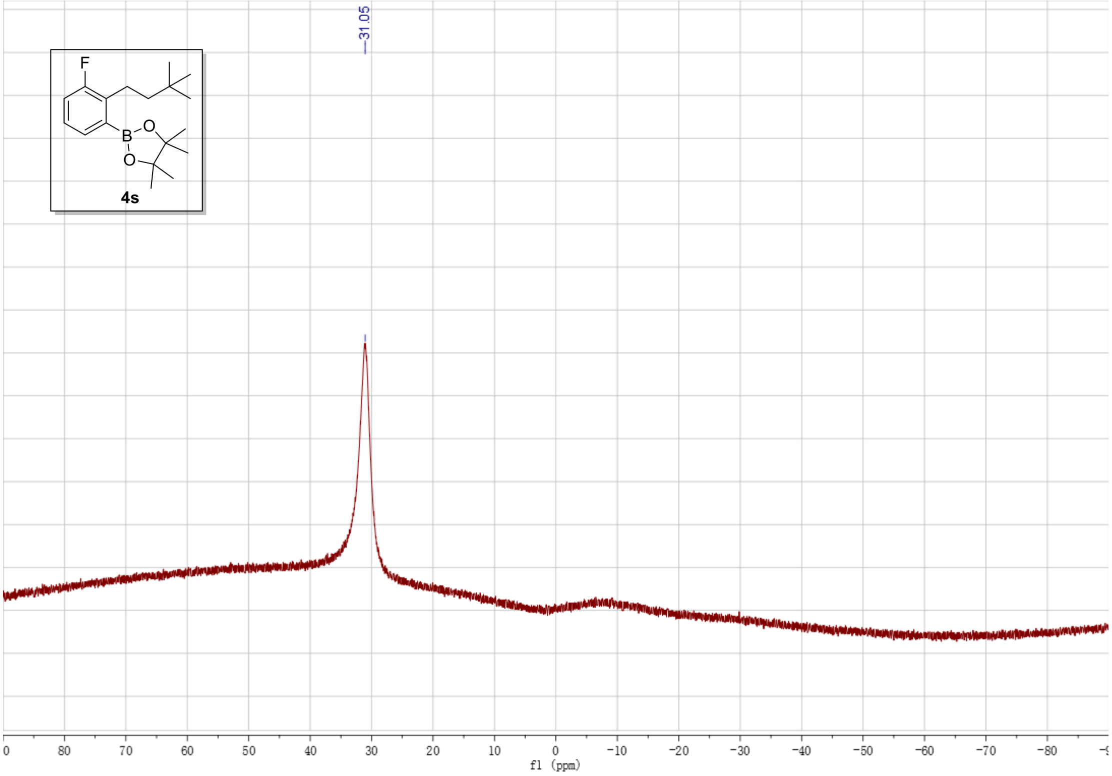

Supplementary Figure 117. ^{11}B spectrum of **4s**.

^{19}F spectrum (471 MHz, room temperature, $CDCl_3$) of (**4s**)

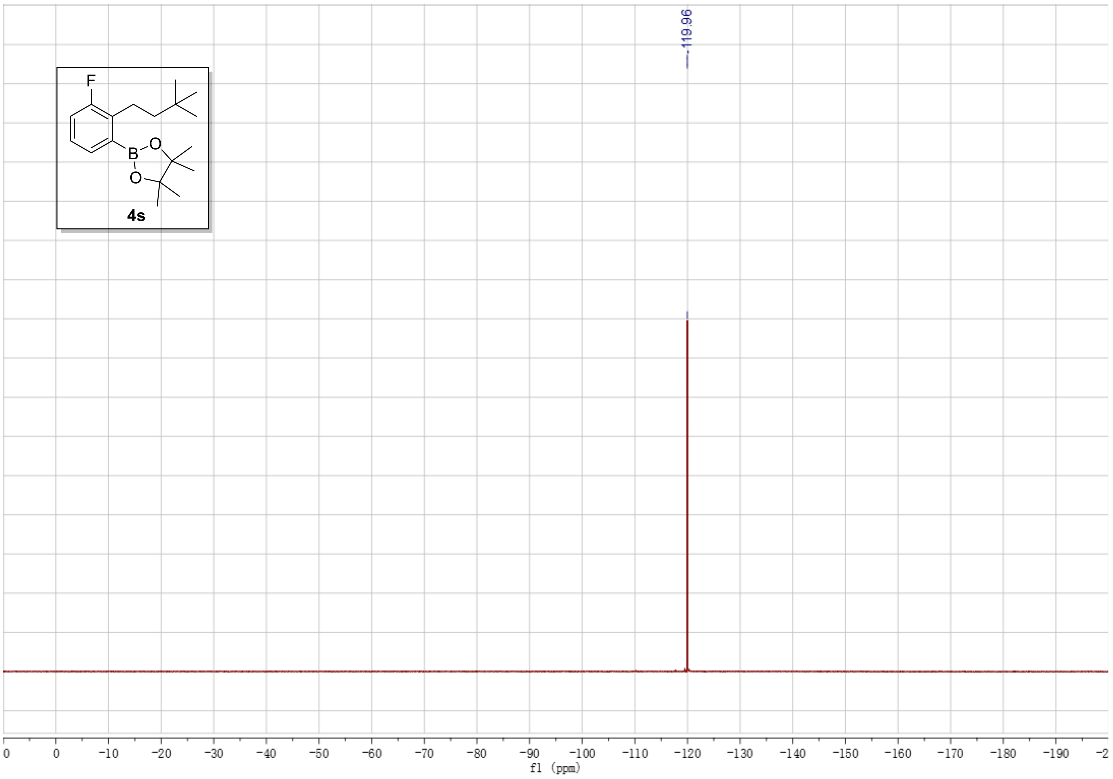

Supplementary Figure 118. ^{19}F spectrum of **4s**.

2-(2-(3,3-dimethylbutyl)-4-fluorophenyl)-4,4,5,5-tetramethyl-1,3,2-dioxaborolane (4t)

^{1}H spectrum (500 MHz, room temperature, $CDCl_3$) of (**4t**)

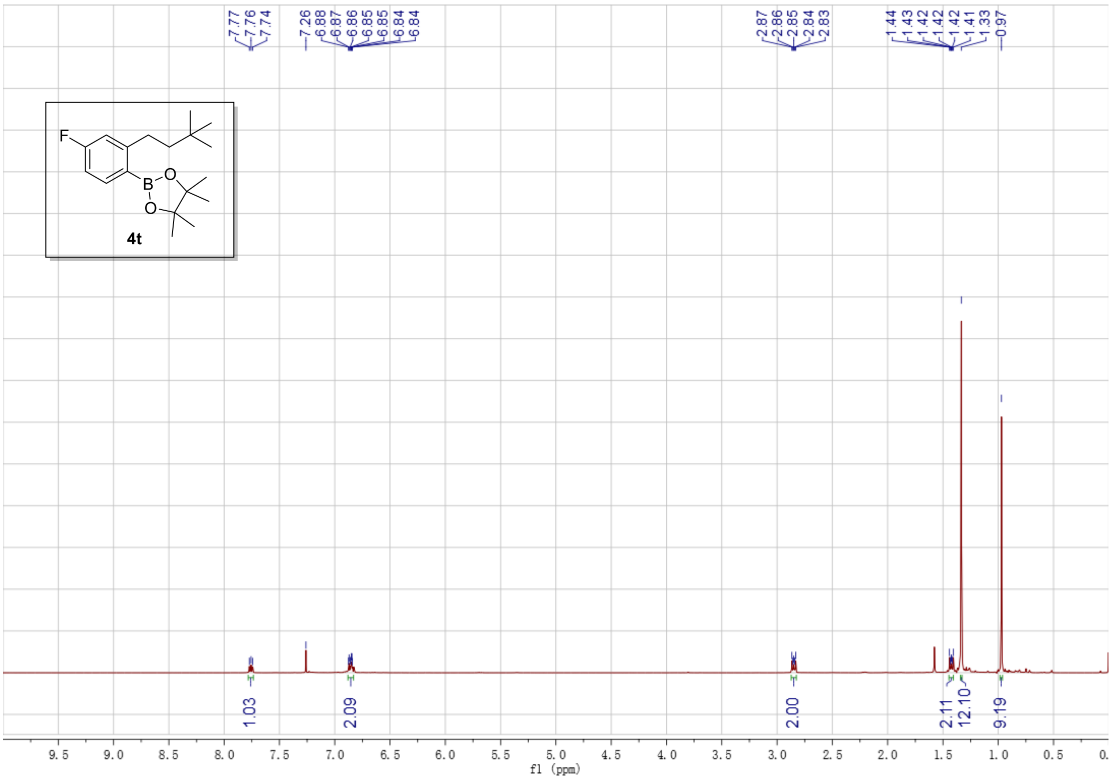

Supplementary Figure 119. ^{1}H NMR spectrum of **4t**.

^{13}C spectrum (126 MHz, room temperature, $CDCl_3$) of (**4t**)

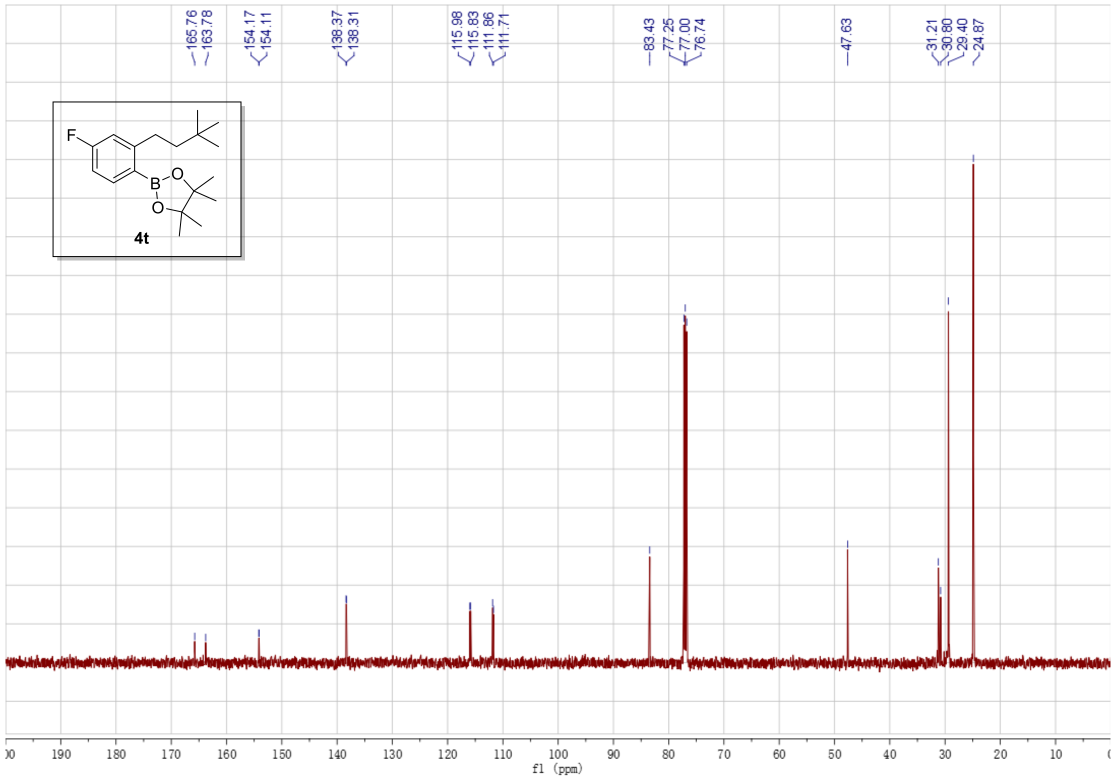

Supplementary Figure 120. ^{13}C NMR spectrum of **4t**.

^{11}B spectrum (128 MHz, room temperature, $CDCl_3$) of (**4t**)

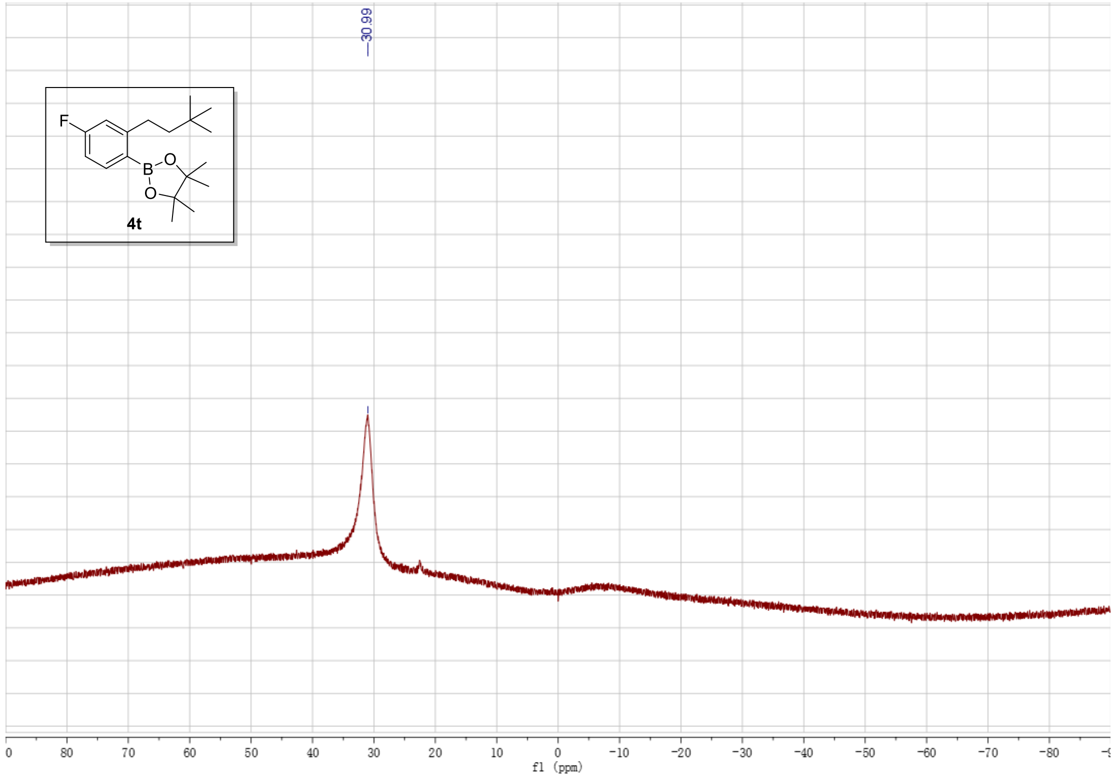

Supplementary Figure 121. ^{11}B spectrum of **4t**.

^{19}F spectrum (471 MHz, room temperature, $CDCl_3$) of (**4t**)

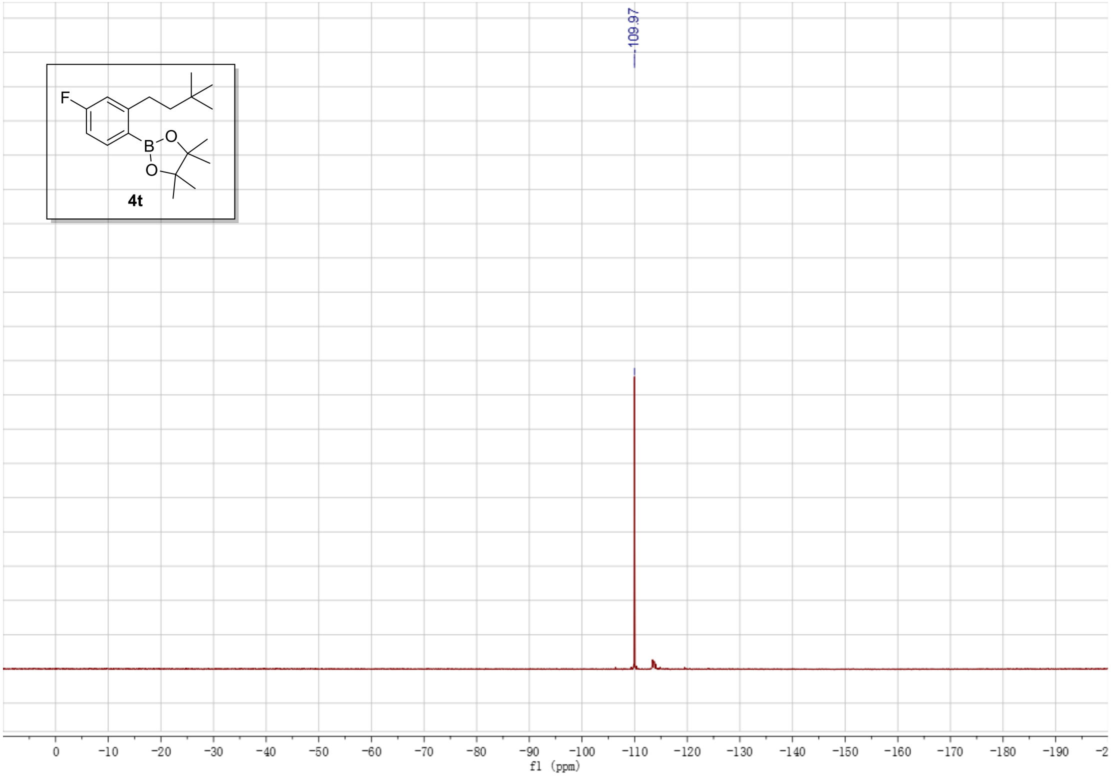

Supplementary Figure 122. ^{19}F spectrum of **4t**.

2-(2-(3,3-dimethylbutyl)-5-fluorophenyl)-4,4,5,5-tetramethyl-1,3,2-dioxaborolan (4u)

^{1}H spectrum (500 MHz, room temperature, $CDCl_3$) of (**4u**)

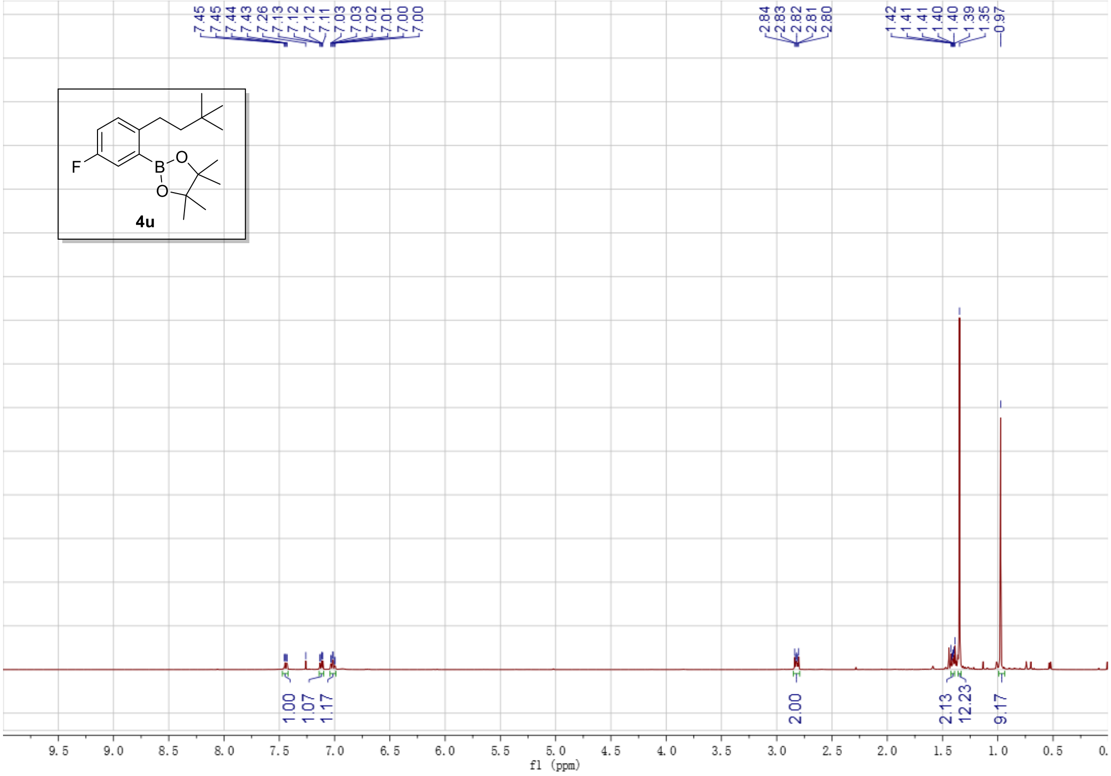

Supplementary Figure 123. ^{1}H NMR spectrum of **4u**.

^{13}C spectrum (126 MHz, room temperature, $CDCl_3$) of (**4u**)

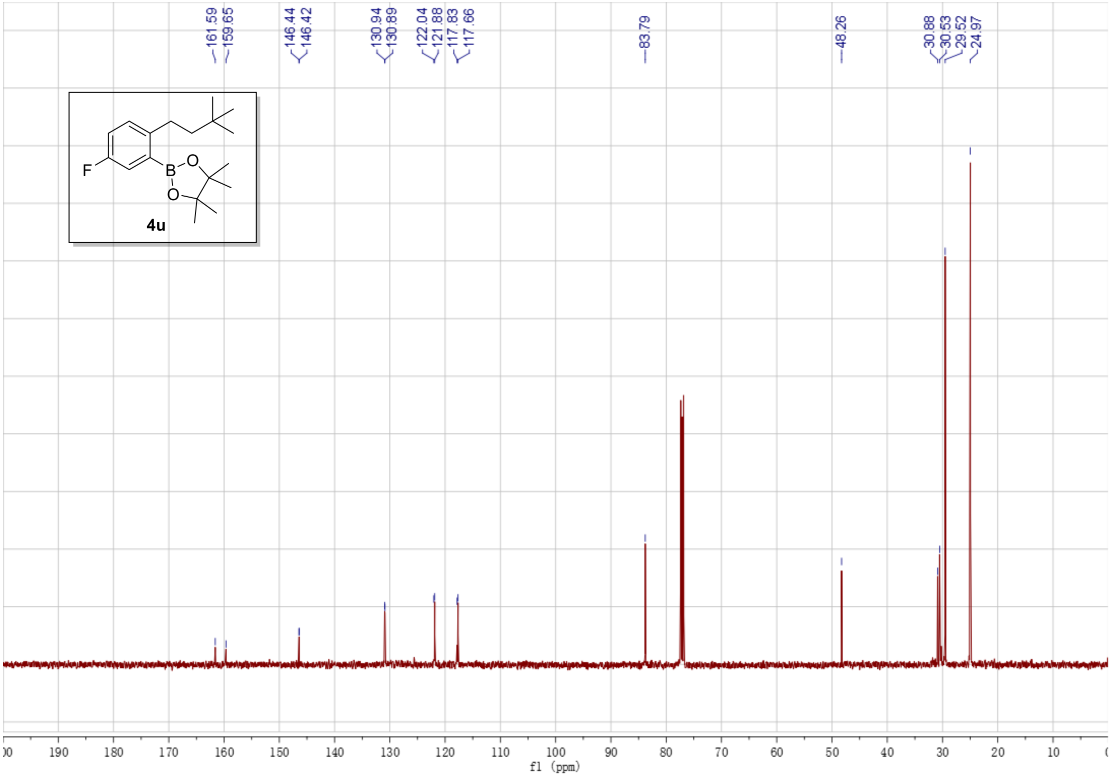

Supplementary Figure 124. ^{13}C NMR spectrum of **4u**.

^{11}B spectrum (128 MHz, room temperature, $CDCl_3$) of (**4u**)

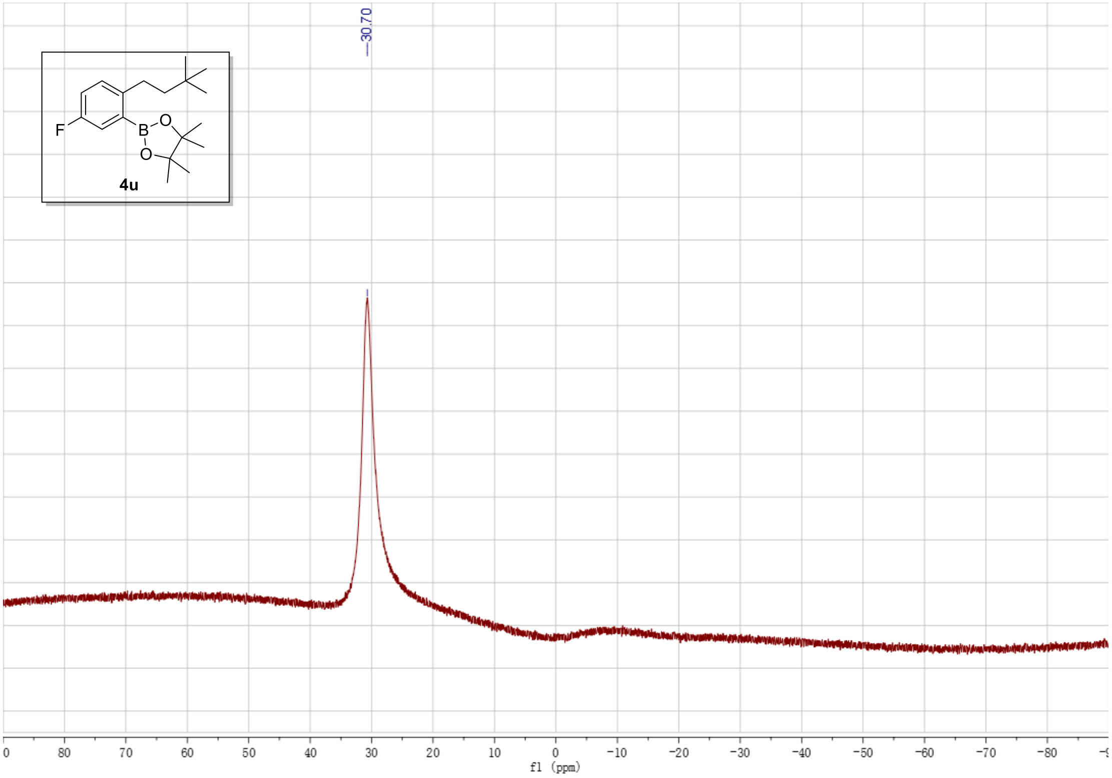

Supplementary Figure 125. ^{11}B spectrum of **4u**.

^{19}F spectrum (471 MHz, room temperature, $CDCl_3$) of (**4u**)

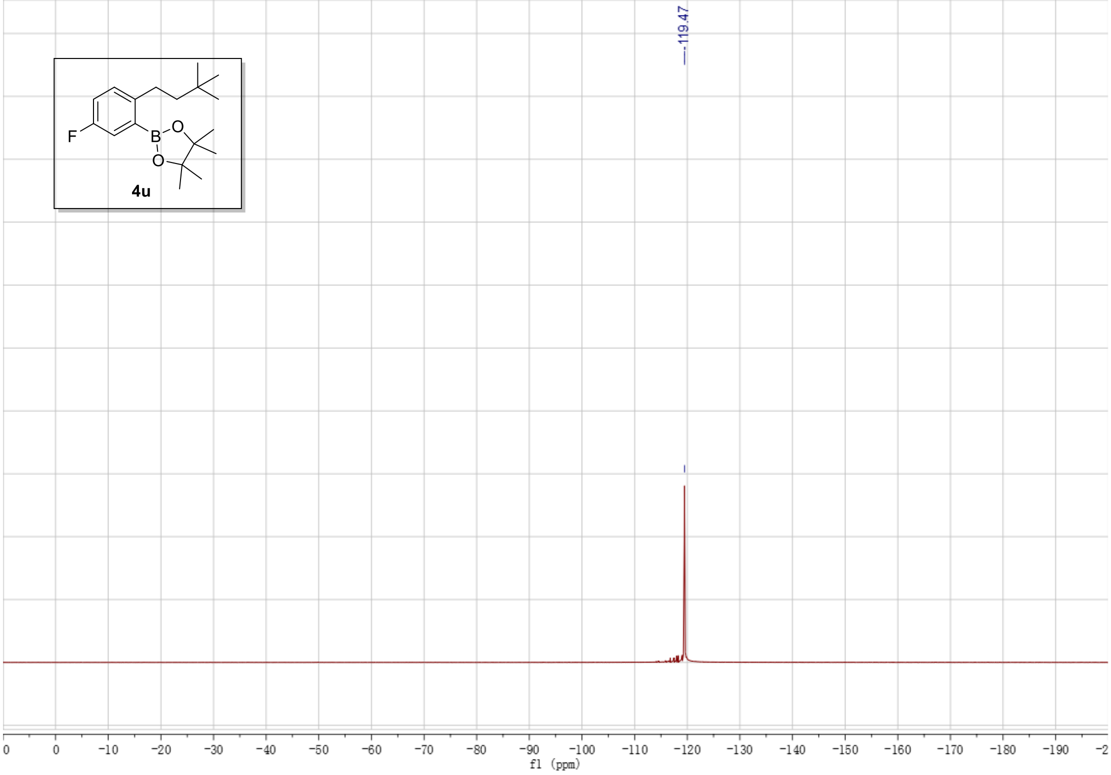

Supplementary Figure 126. ^{19}F spectrum of **4u**.

2-(2-(3,3-dimethylbutyl)naphthalen-1-yl)-4,4,5,5-tetramethyl-1,3,2-dioxaborolane (4v)

^{1}H spectrum (500 MHz, room temperature, $CDCl_3$) of (**4v**)

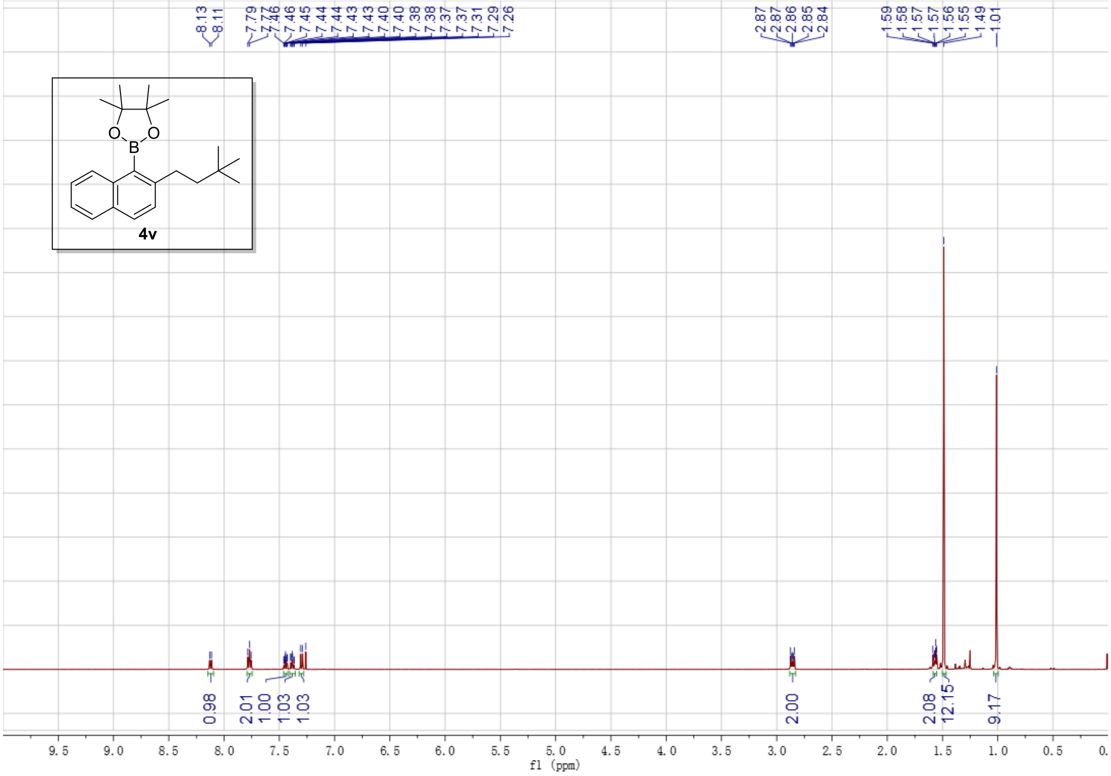

Supplementary Figure 127. ^{1}H NMR spectrum of **4v**.

^{13}C spectrum (126 MHz, room temperature, $CDCl_3$) of (**4v**)

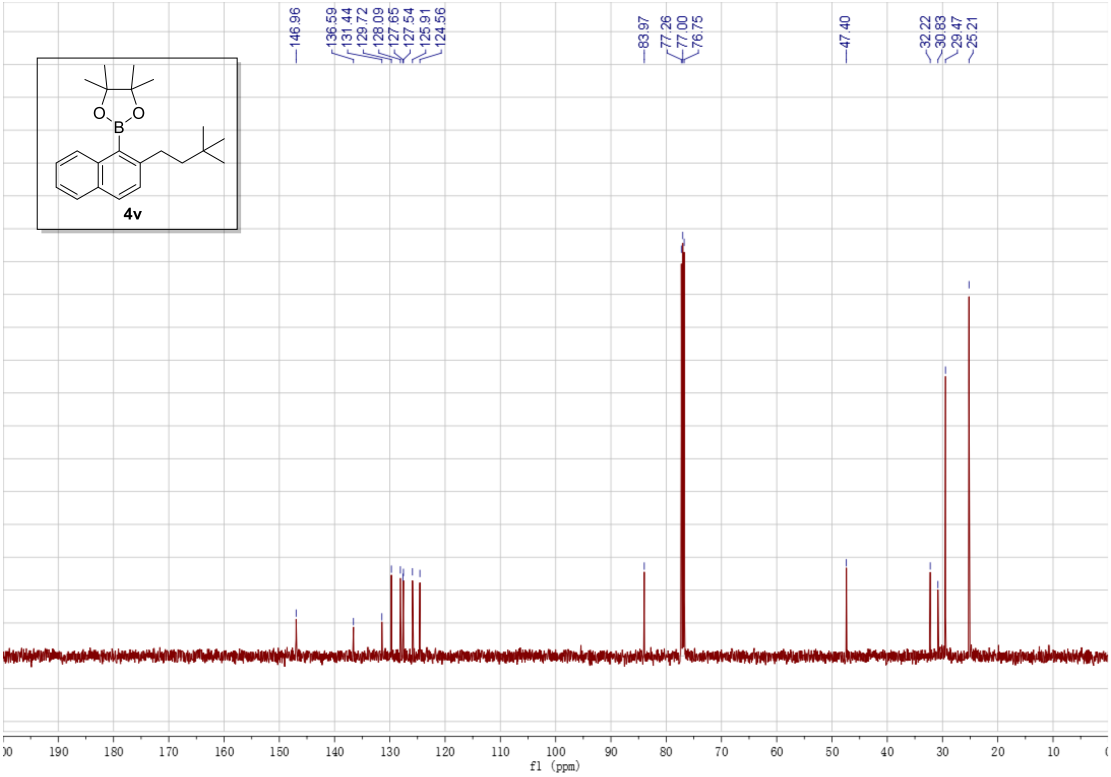

Supplementary Figure 128. ^{13}C NMR spectrum of **4v**.

^{11}B spectrum (128 MHz, room temperature, $CDCl_3$) of (**4v**)

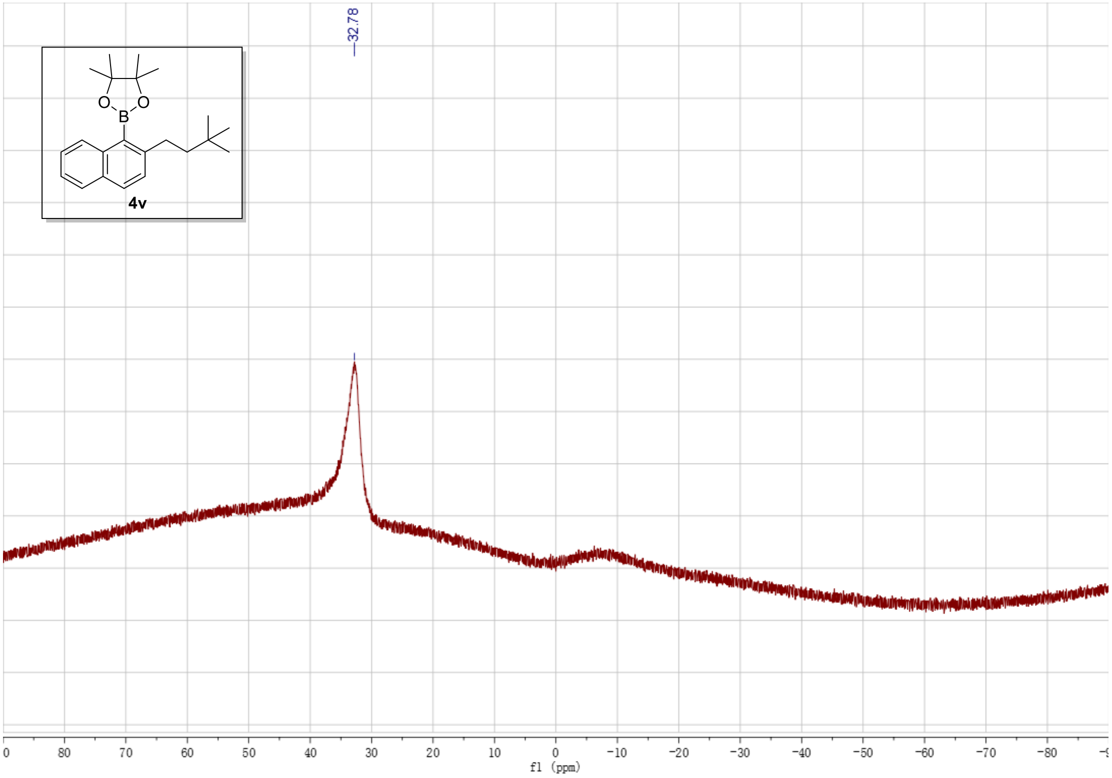

Supplementary Figure 129. ^{11}B spectrum of **4v**.

2-(3-(3,3-dimethylbutyl)phenyl)-4,4,5,5-tetramethyl-1,3,2-dioxaborolane (4w)

^{1}H spectrum (500 MHz, room temperature, $CDCl_3$) of (**4w**)

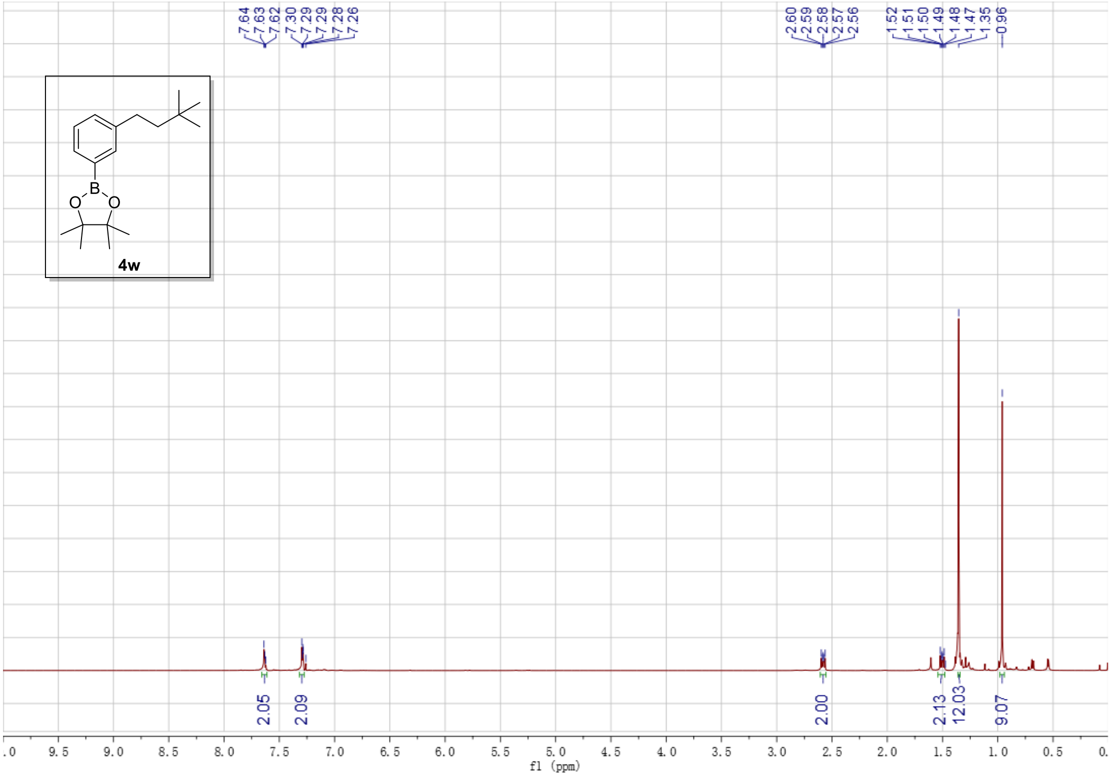

Supplementary Figure 130. ^{1}H NMR spectrum of **4w**.

^{13}C spectrum (126MHz, room temperature, $CDCl_3$) of (**4w**)

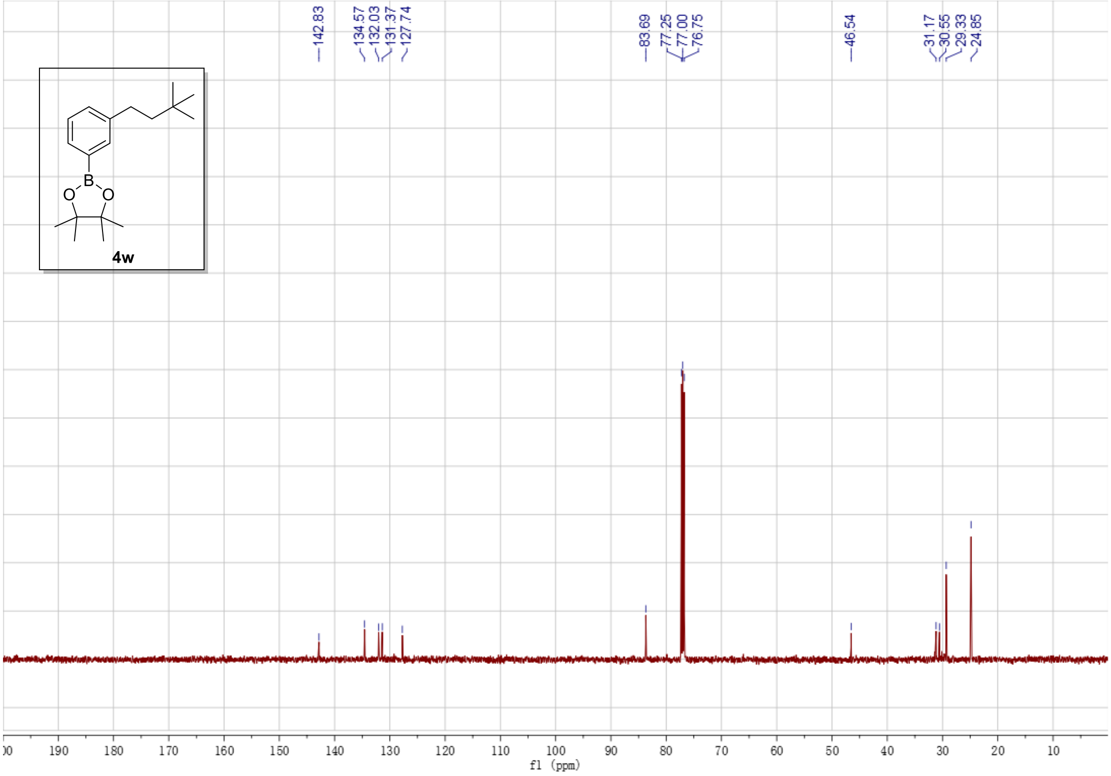

Supplementary Figure 131. ^{13}C NMR spectrum of **4w**.

^{11}B spectrum (128 MHz, room temperature, $CDCl_3$) of (**4w**)

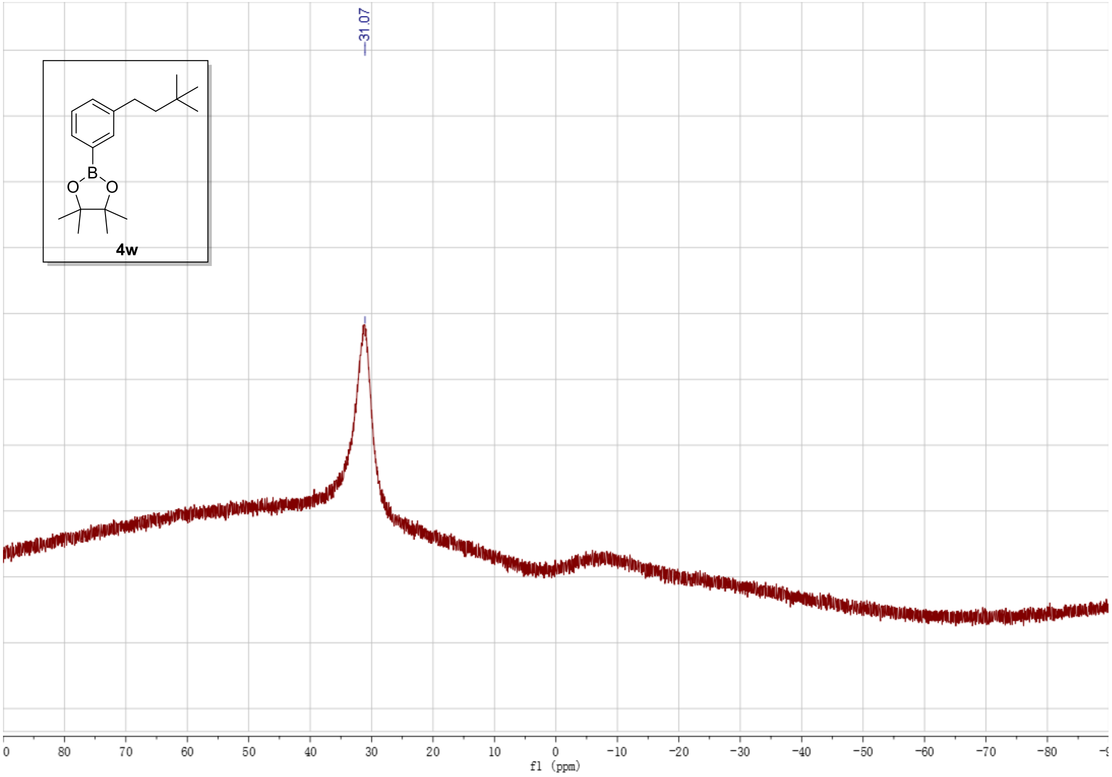

Supplementary Figure 132. ^{11}B spectrum of **4w**.

2-(4-(3,3-dimethylbutyl)phenyl)-4,4,5,5-tetramethyl-1,3,2-dioxaborolane (4x)

^{1}H spectrum (500 MHz, room temperature, $CDCl_3$) of (**4x**)

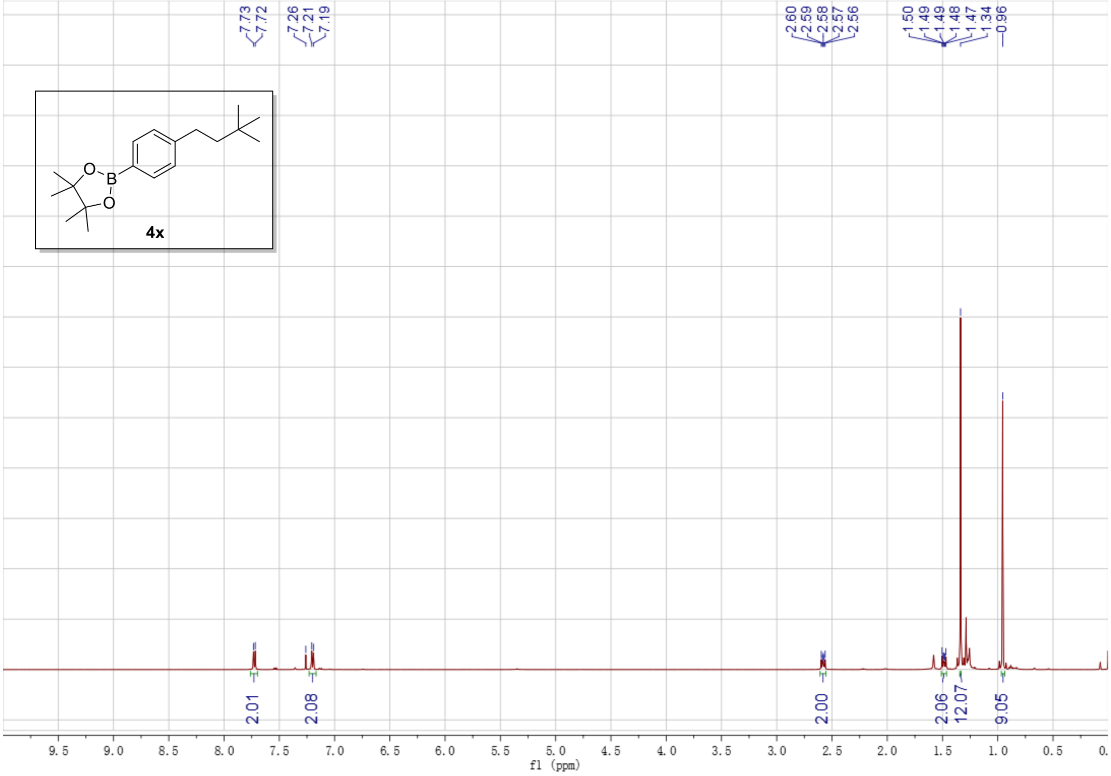

Supplementary Figure 133. ^{1}H NMR spectrum of **4x**.

^{13}C spectrum (126 MHz, room temperature, $CDCl_3$) of (**4x**)

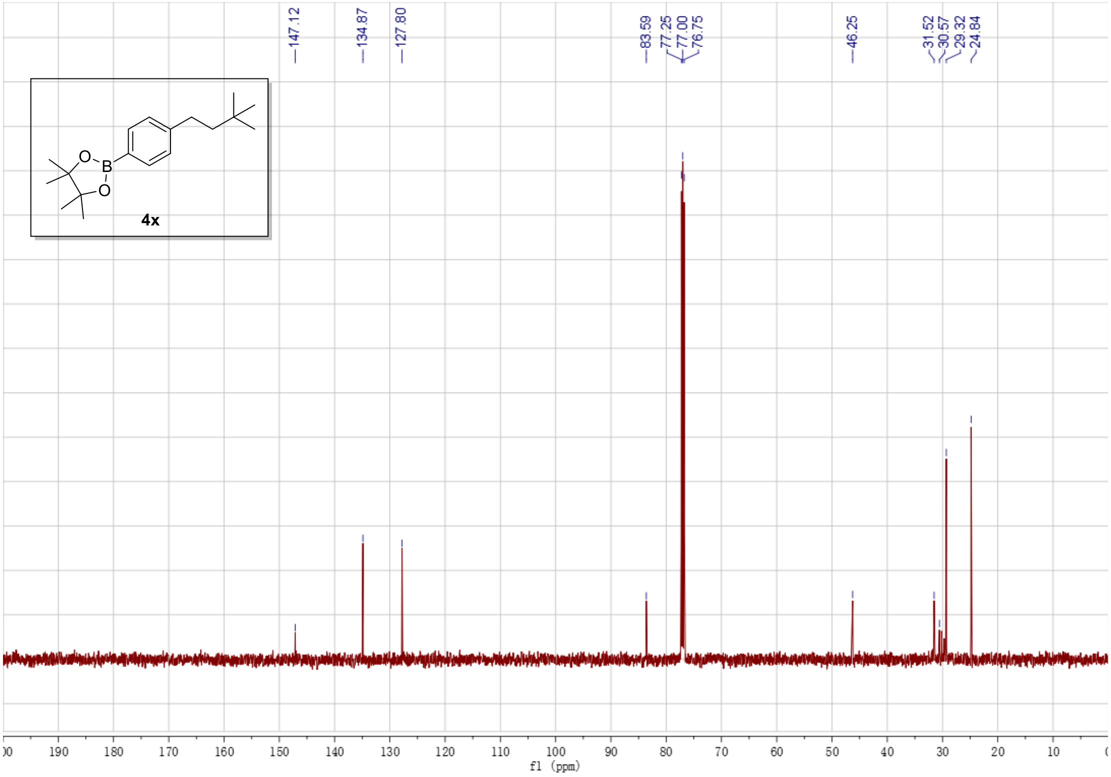

Supplementary Figure 134. ^{13}C NMR spectrum of **4x**.

^{11}B spectrum (128 MHz, room temperature, $CDCl_3$) of (**4x**)

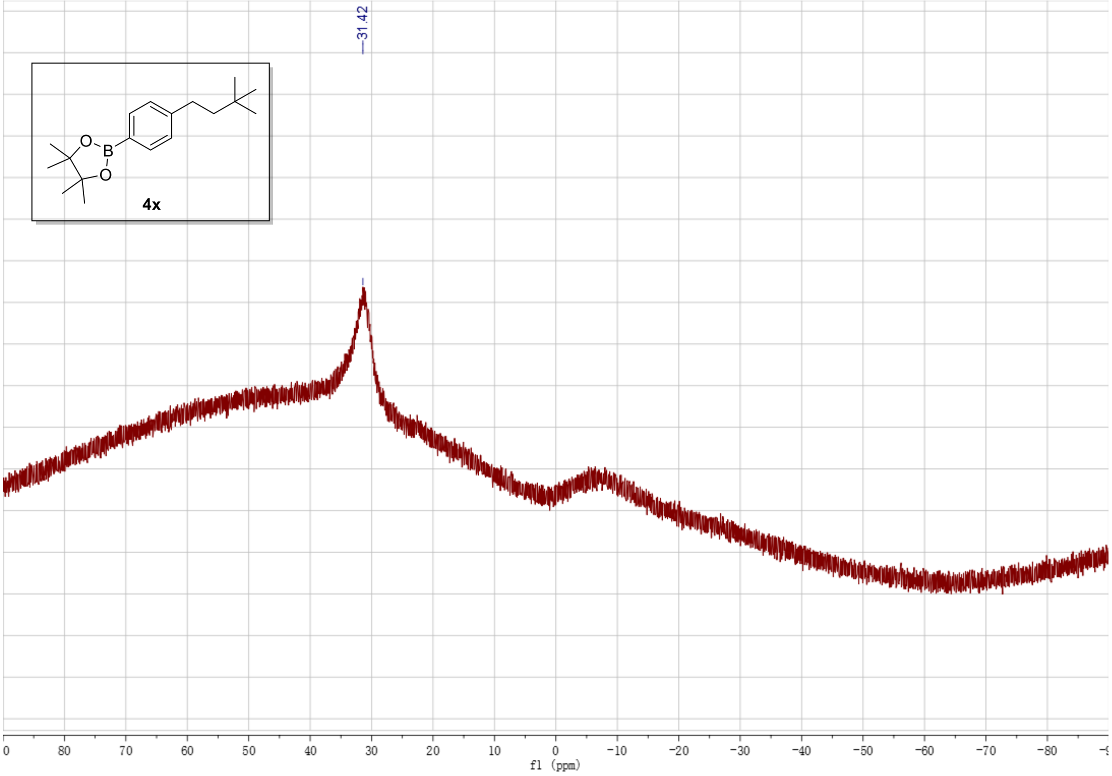

Supplementary Figure 135. ^{11}B spectrum of **4x**.

2-(4-(4,4-dimethylpentan-2-yl)phenyl)-4,4,5,5-tetramethyl-1,3,2-dioxaborolane (4y)

^{1}H spectrum (500 MHz, room temperature, $CDCl_3$) of (**4y**)

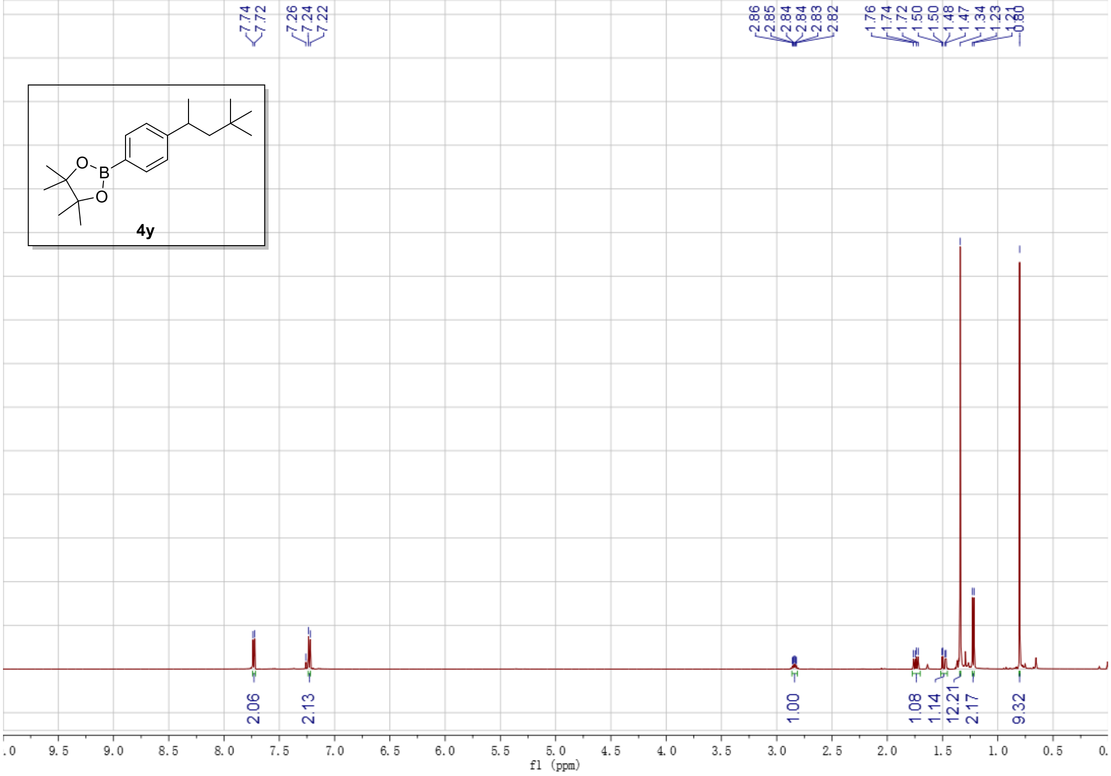

Supplementary Figure 136. ^{1}H NMR spectrum of **4y**.

^{13}C spectrum (126 MHz, room temperature, $CDCl_3$) of (**4y**)

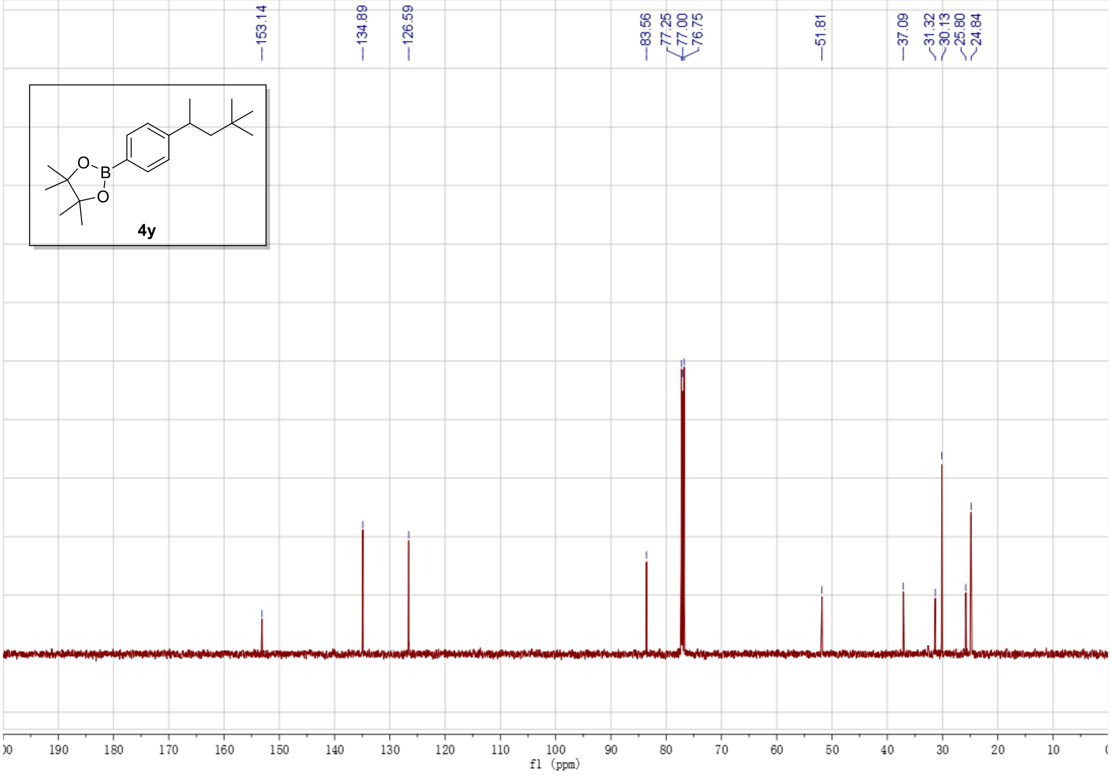

Supplementary Figure 137. ^{13}C NMR spectrum of **4y**.

^{11}B spectrum (128 MHz, room temperature, $CDCl_3$) of (**4y**)

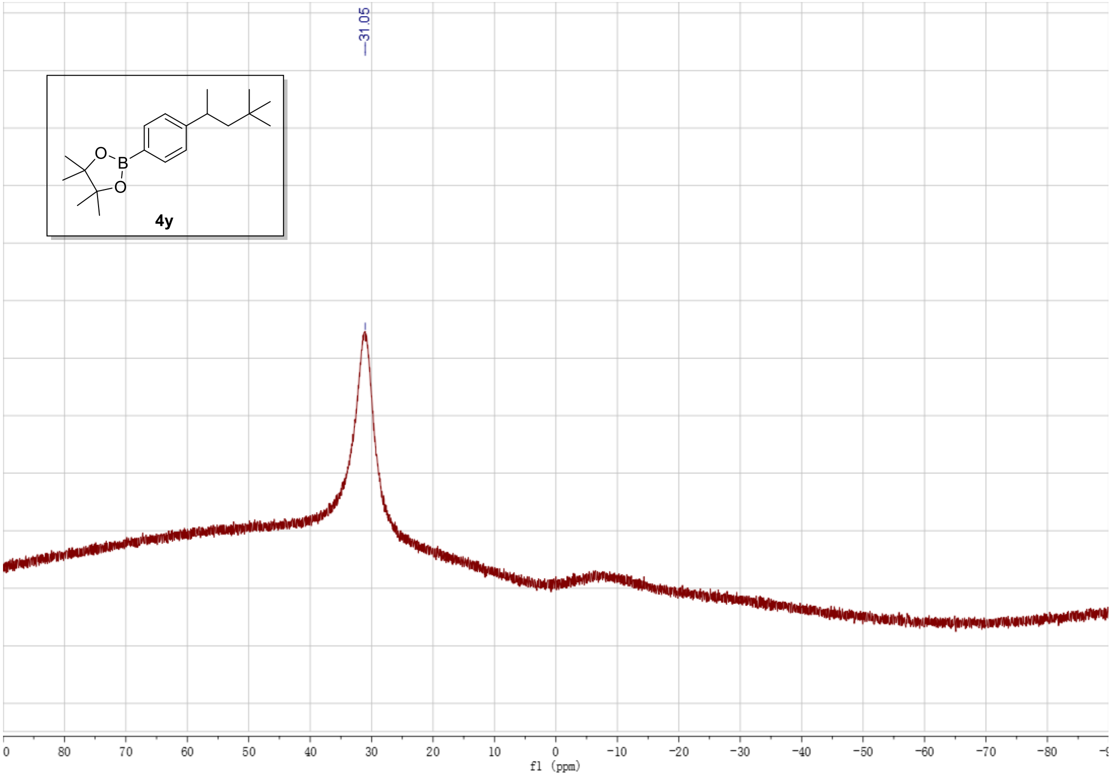

Supplementary Figure 138. ^{11}B spectrum of **4y**.

2-(4-(5,5-dimethylhexan-3-yl)phenyl)-4,4,5,5-tetramethyl-1,3,2-dioxaborolane (4z)
^{1}H spectrum (500 MHz, room temperature, $CDCl_3$) of (**4z**)

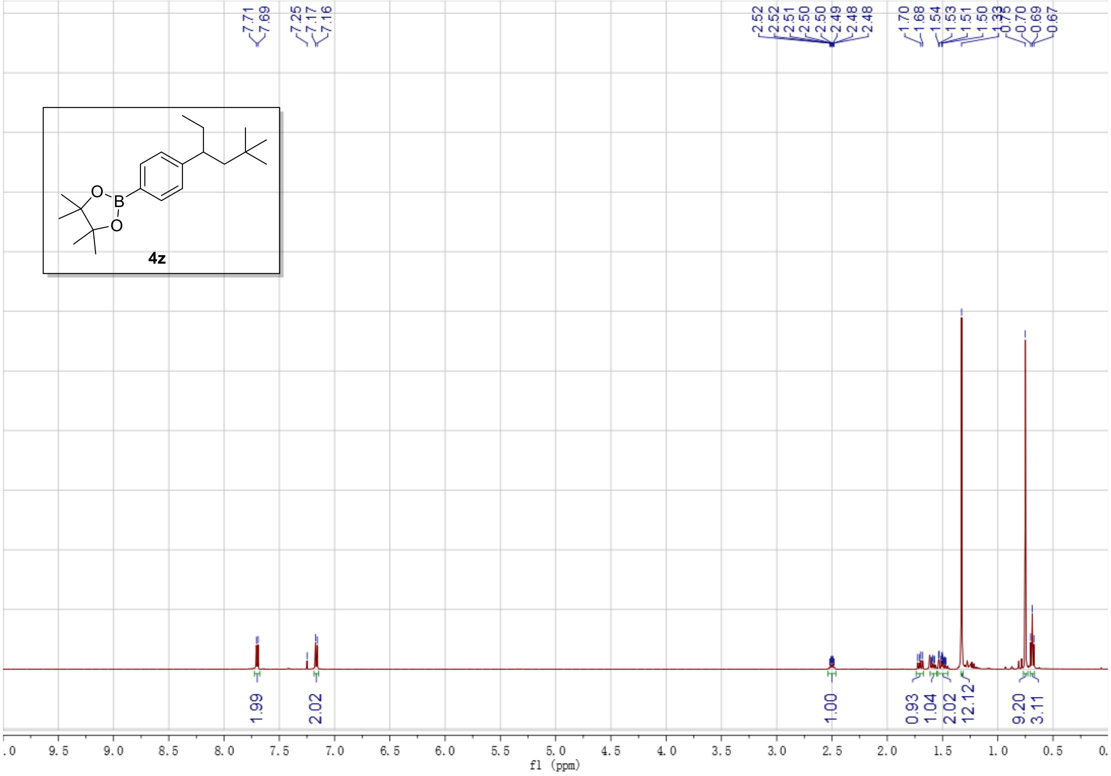

Supplementary Figure 139. ^{1}H NMR spectrum of **4z**.

^{13}C spectrum (126 MHz, room temperature, $CDCl_3$) of (**4z**)

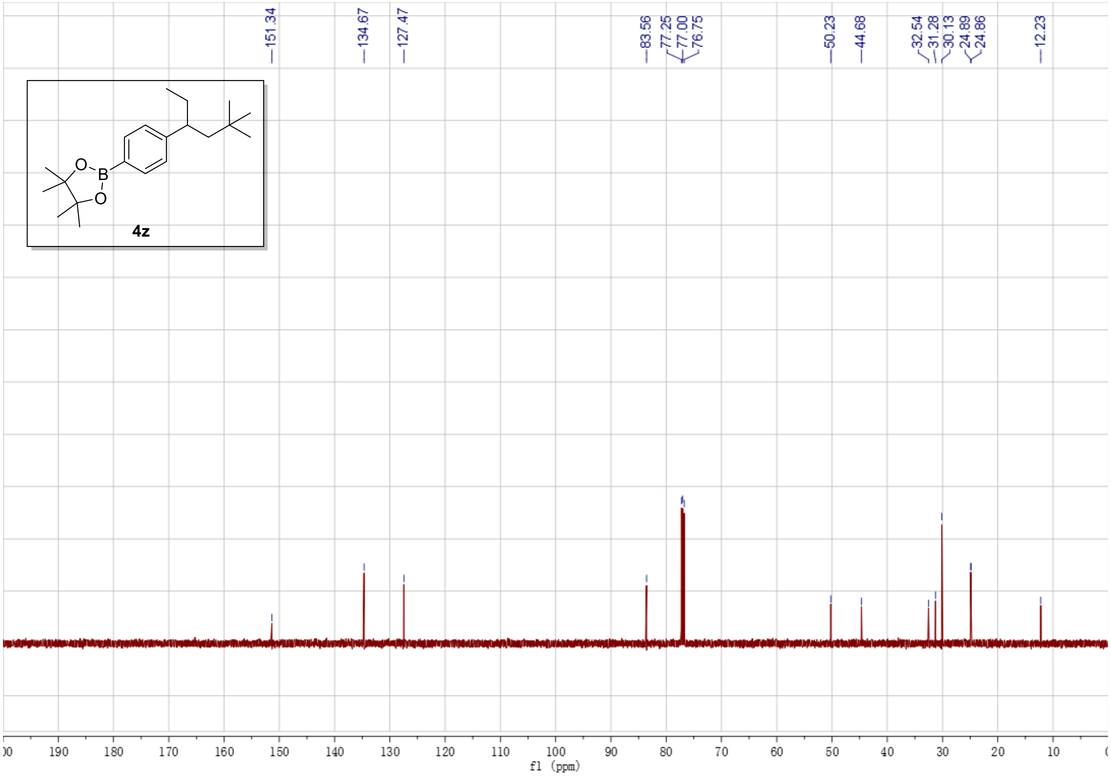

Supplementary Figure 140. ^{13}C NMR spectrum of **4z**.

^{11}B spectrum (128 MHz, room temperature, $CDCl_3$) of (**4z**)

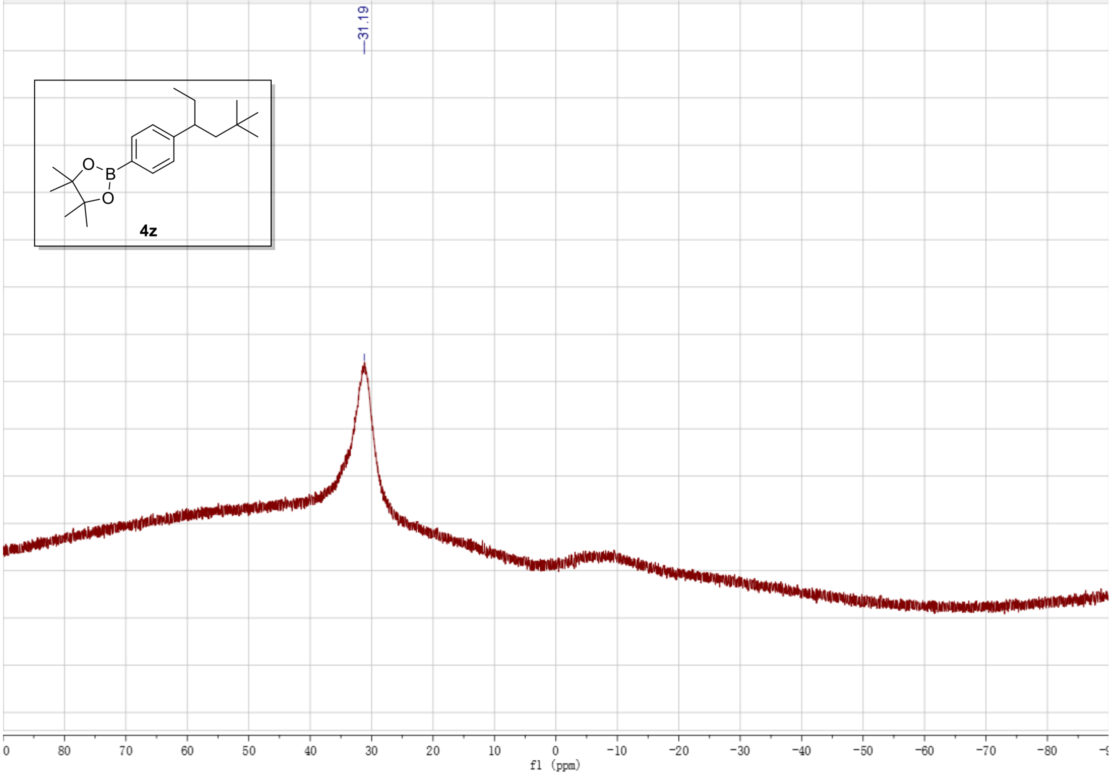

Supplementary Figure 141. ^{11}B spectrum of **4z**.

2-(4-(2,2-dimethyloctan-4-yl)phenyl)-4,4,5,5-tetramethyl-1,3,2-dioxaborolane (4aa)

^{1}H spectrum (500 MHz, room temperature, $CDCl_3$) of (**4aa**)

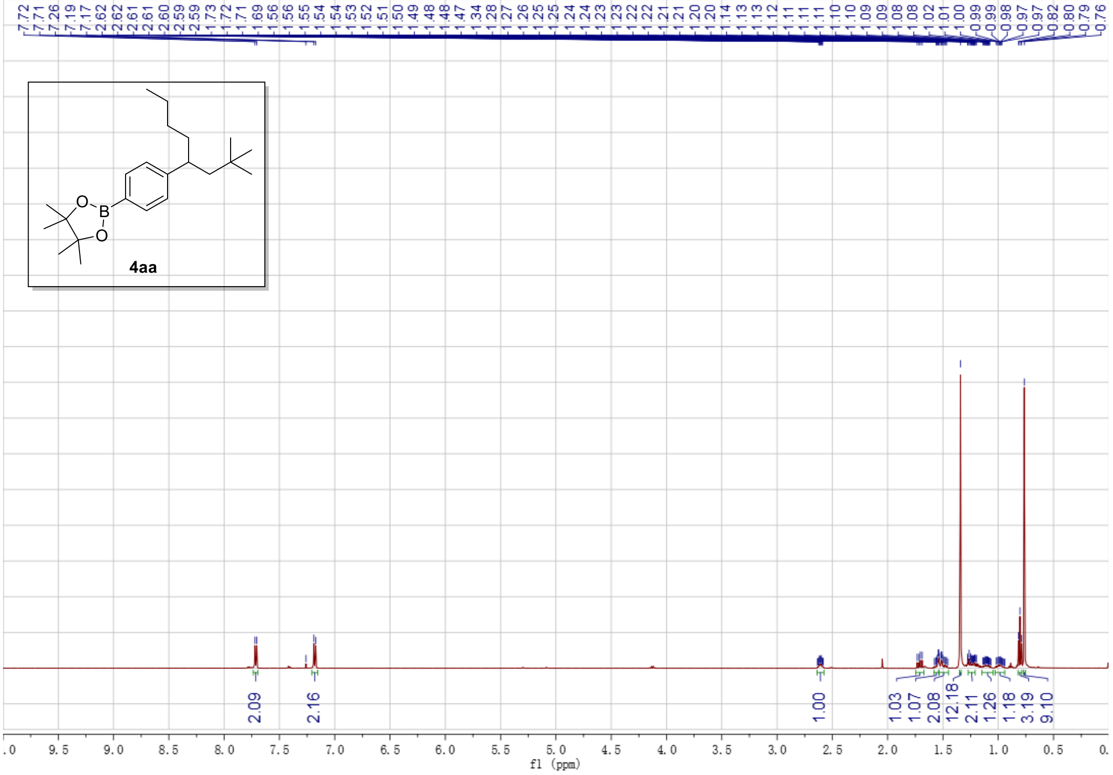

Supplementary Figure 142. ^{1}H NMR spectrum of **4aa**.

^{13}C spectrum (126 MHz, room temperature, $CDCl_3$) of (**4aa**)

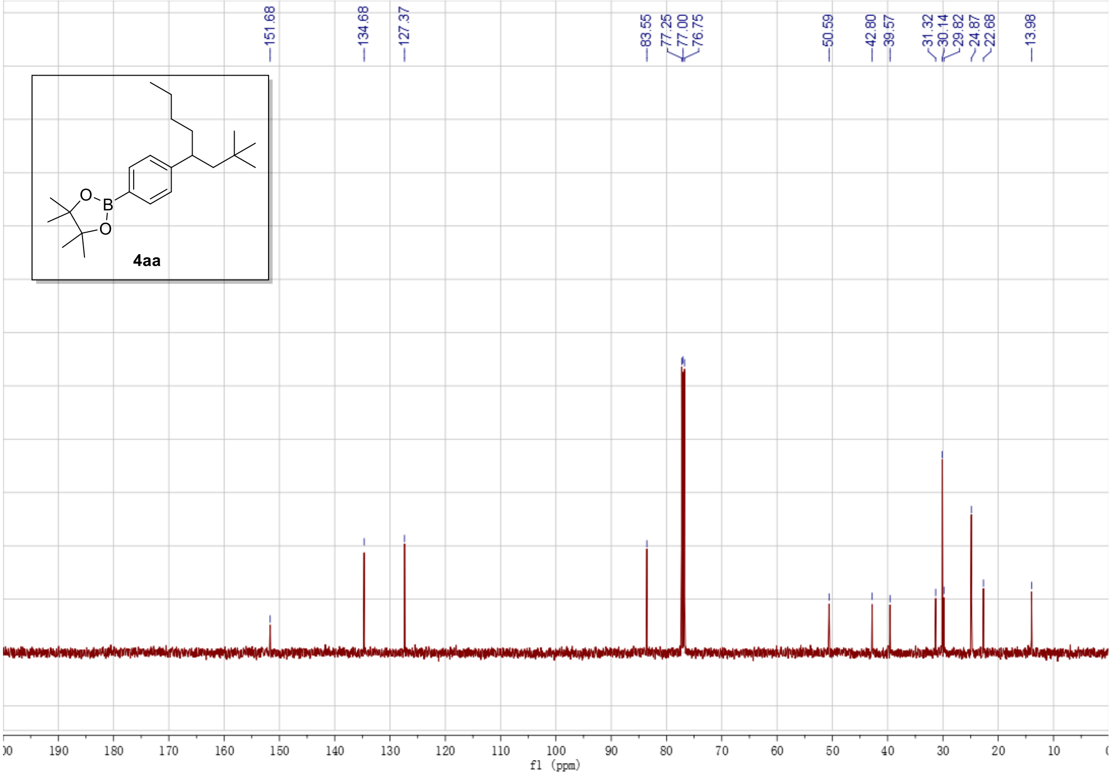

Supplementary Figure 143. ^{13}C NMR spectrum of **4aa**.

^{11}B spectrum (128 MHz, room temperature, $CDCl_3$) of (**4aa**)

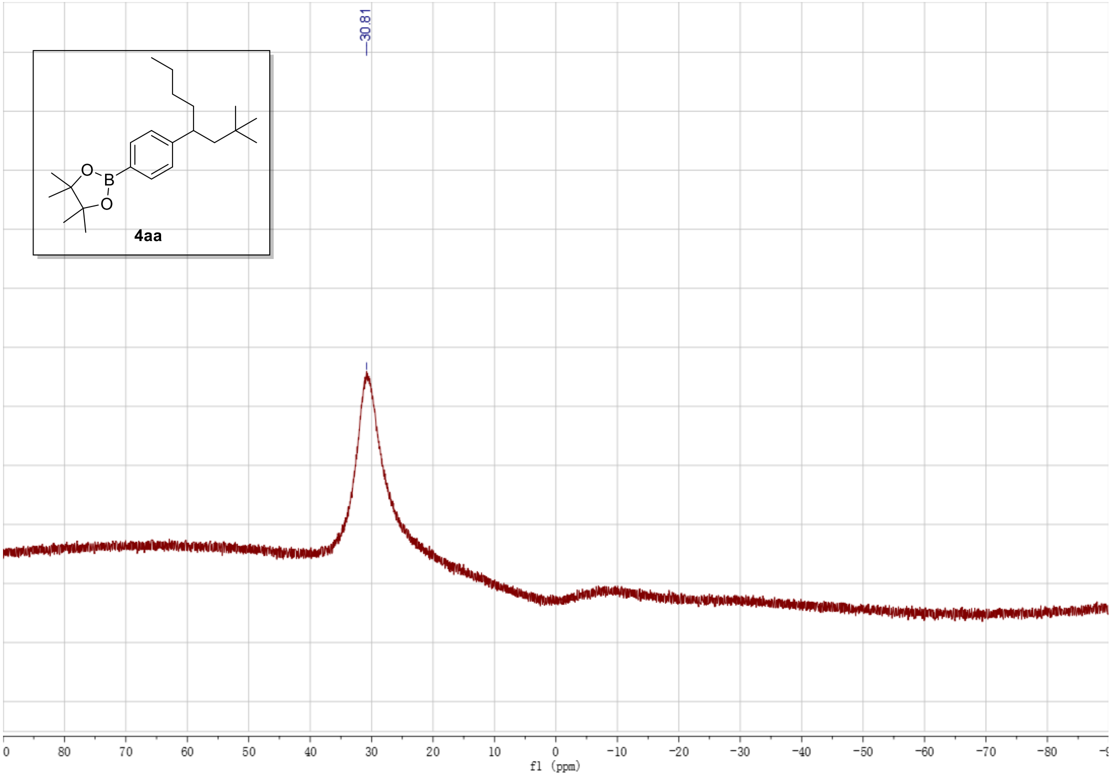

Supplementary Figure 144. ^{11}B spectrum of **4aa**.

2-(4-(4,4-dimethyl-1-phenylpentan-2-yl)phenyl)-4,4,5,5-tetramethyl-1,3,2-dioxaborolane (4ab)

^{1}H spectrum (500 MHz, room temperature, $CDCl_3$) of (**4ab**)

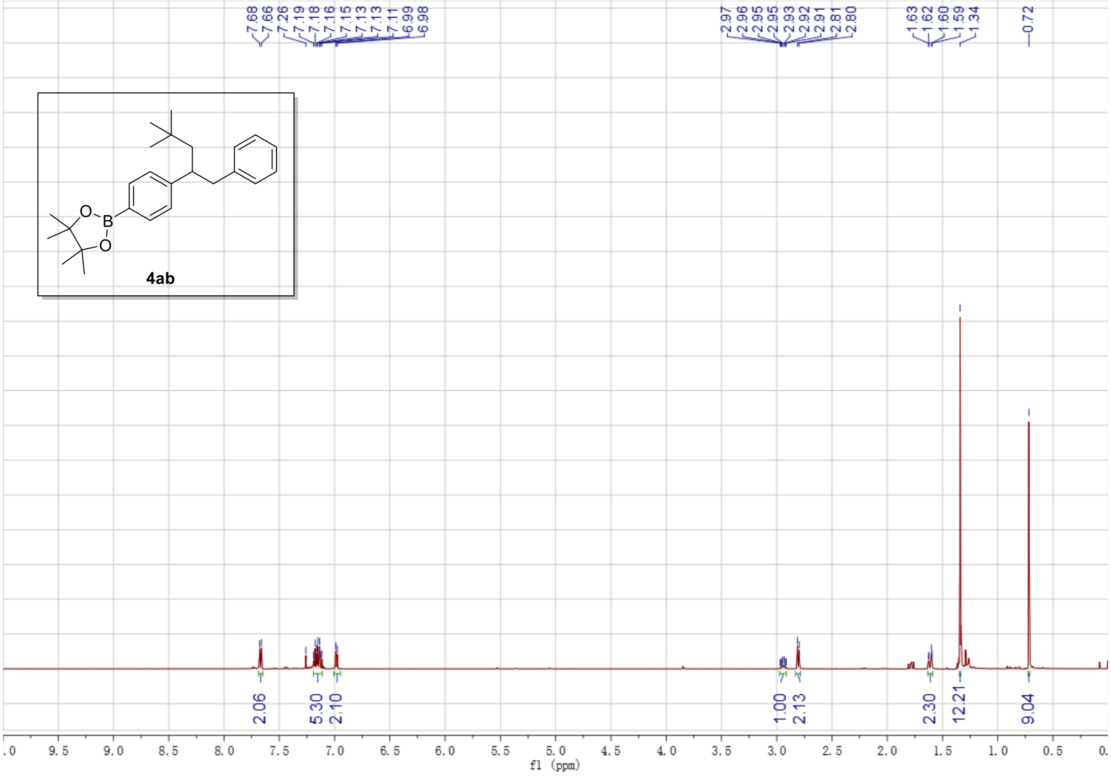

Supplementary Figure 145. ^{1}H NMR spectrum of **4ab**.

^{13}C spectrum (126 MHz, room temperature, $CDCl_3$) of (**4ab**)

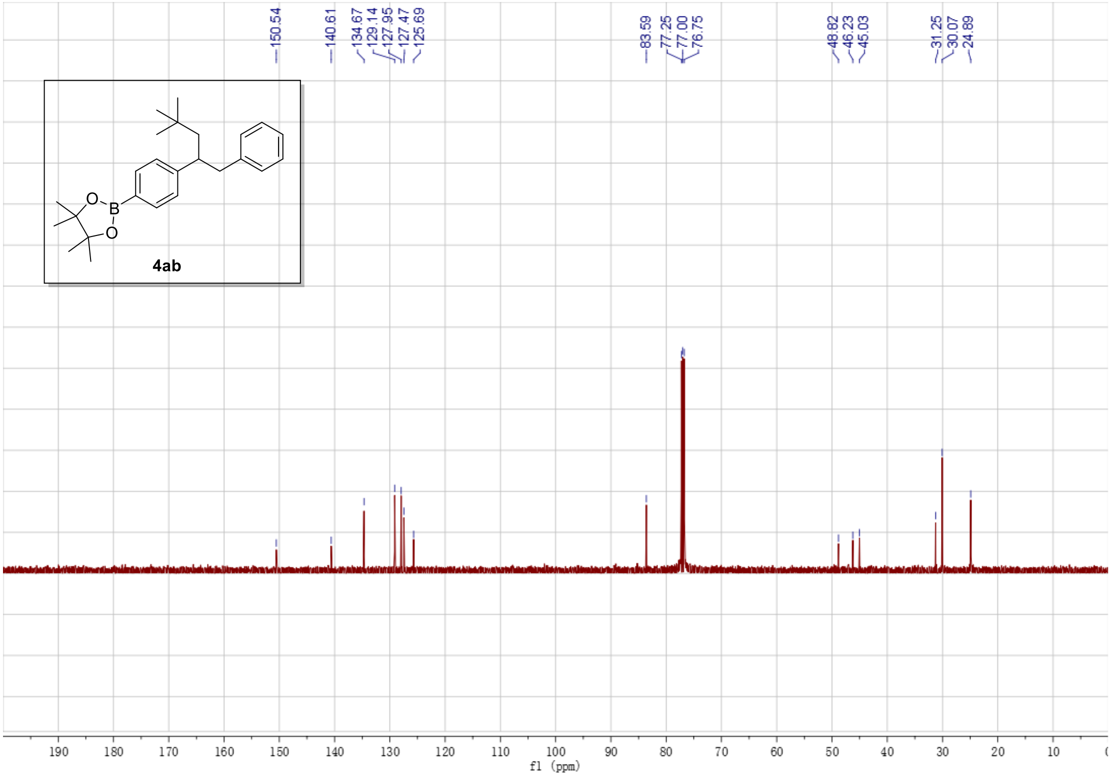

Supplementary Figure 146. ^{13}C NMR spectrum of **4ab**.

^{11}B spectrum (128 MHz, room temperature, $CDCl_3$) of (**4ab**)

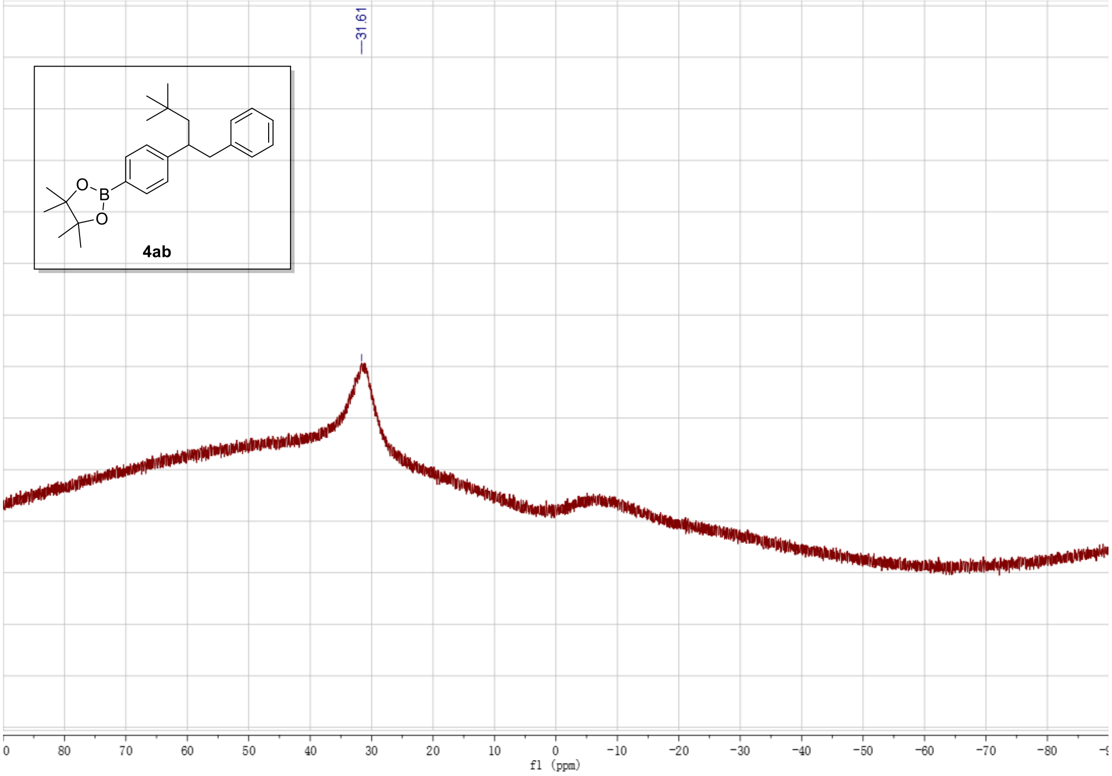

Supplementary Figure 147. ^{11}B spectrum of **4ab**.

4,4,5,5-tetramethyl-2-(1-neopentyl-2,3-dihydro-1H-inden-5-yl)-1,3,2-dioxaborolane (4ac)

^{1}H spectrum (500 MHz, room temperature, $CDCl_3$) of (**4ac**)

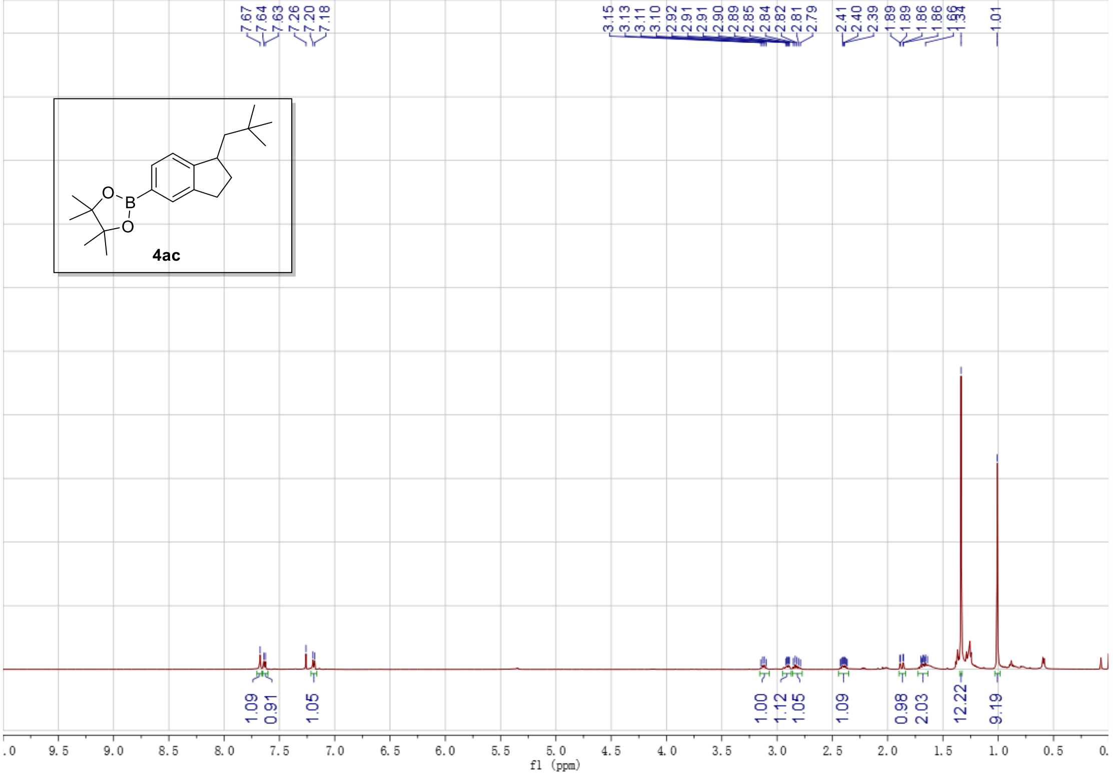

Supplementary Figure 148. ^{1}H NMR spectrum of **4ac**.

^{13}C spectrum (126 MHz, room temperature, $CDCl_3$) of (**4ac**)

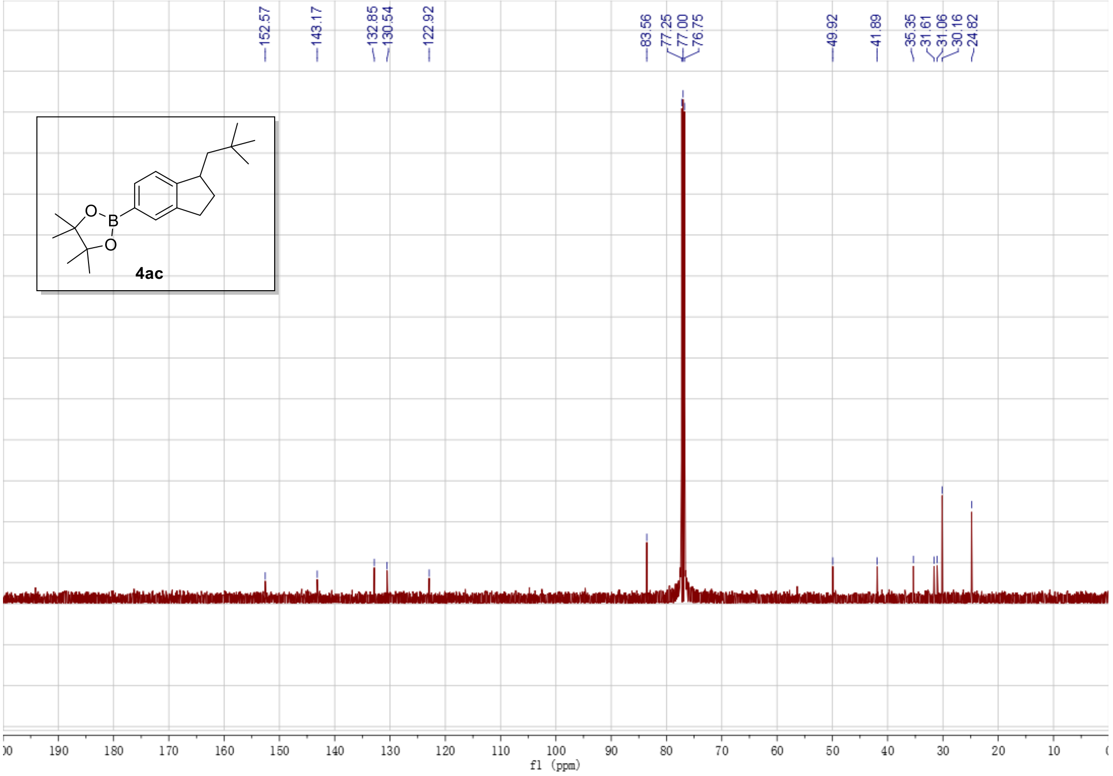

Supplementary Figure 149. ^{13}C NMR spectrum of **4ac**.

^{11}B spectrum (128 MHz, room temperature, $CDCl_3$) of (**4ac**)

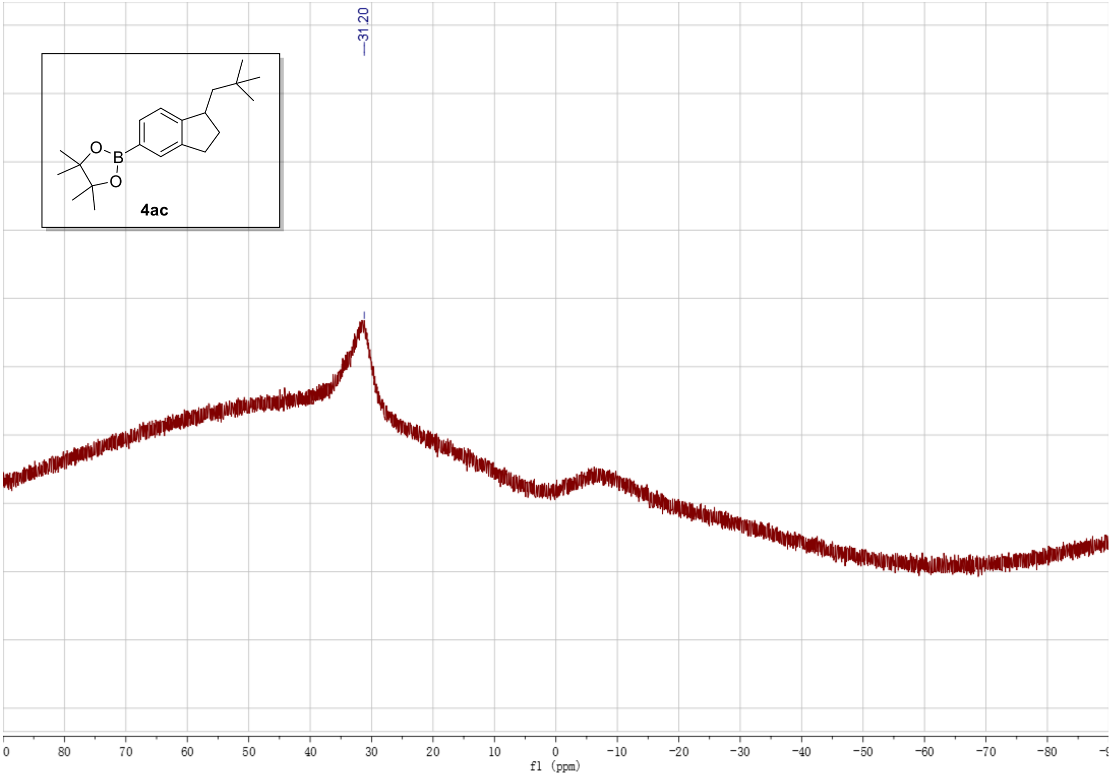

Supplementary Figure 150. ^{11}B spectrum of **4ac**.

2-(2-(2-cyclobutylethyl)-4-methoxyphenyl)-4,4,5,5-tetramethyl-1,3,2-dioxaborolane (4ad)

^{1}H spectrum (500 MHz, room temperature, $CDCl_3$) of (**4ad**)

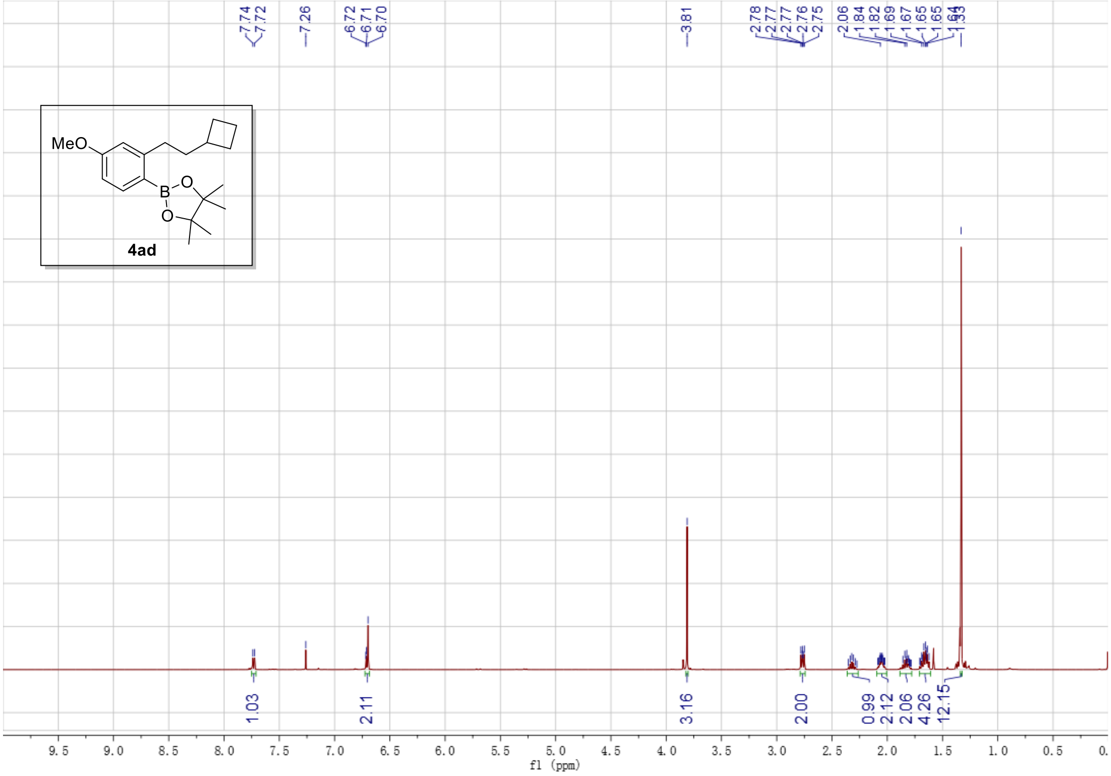

Supplementary Figure 151. ^{1}H NMR spectrum of **4ad**.

^{13}C spectrum (126 MHz, room temperature, $CDCl_3$) of (**4ad**)

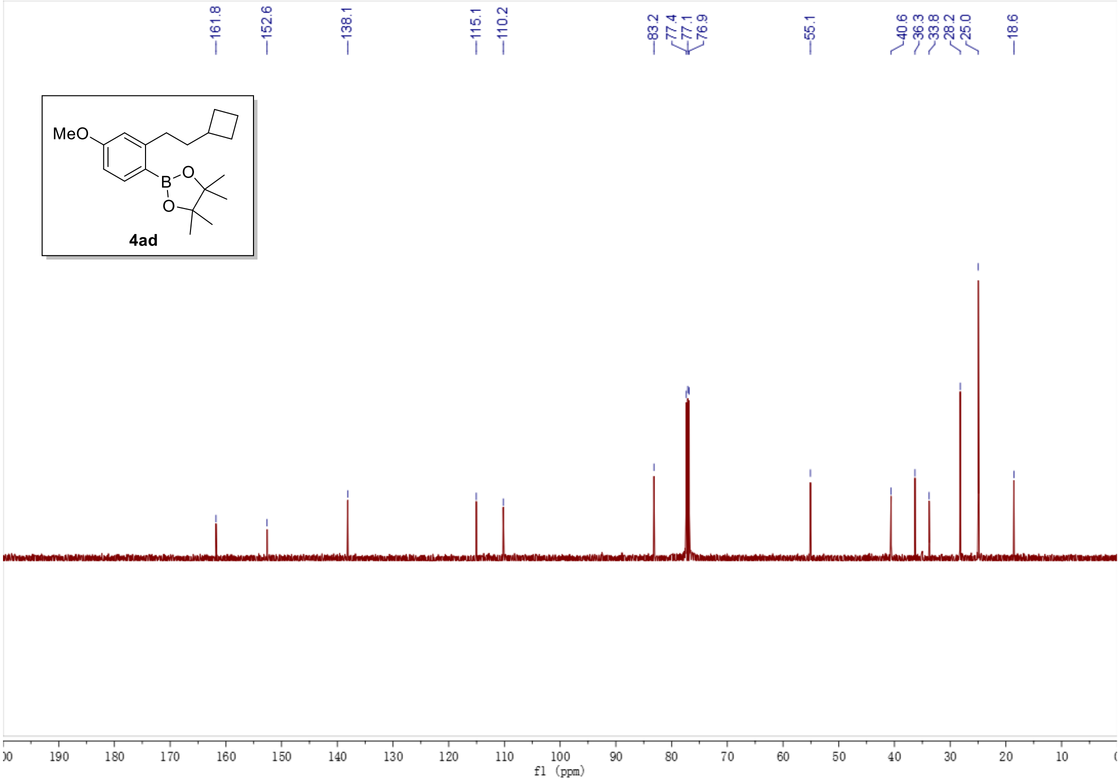

Supplementary Figure 152. ^{13}C NMR spectrum of **4ad**.

^{11}B spectrum (128 MHz, room temperature, $CDCl_3$) of (**4ad**)

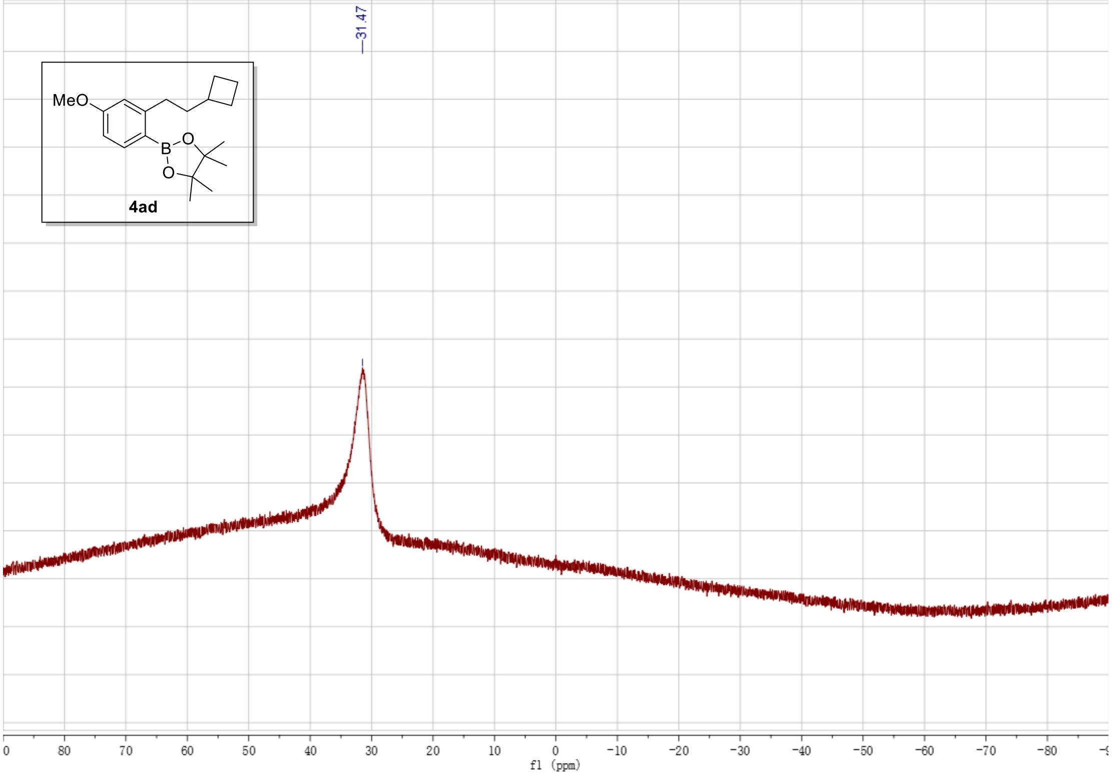

Supplementary Figure 153. ^{11}B spectrum of **4ad**.

2-(2-(2-cyclopentylethyl)-4-methoxyphenyl)-4,4,5,5-tetramethyl-1,3,2-dioxaborolane (4ae)

^{1}H spectrum (500 MHz, room temperature, $CDCl_3$) of (**4ae**)

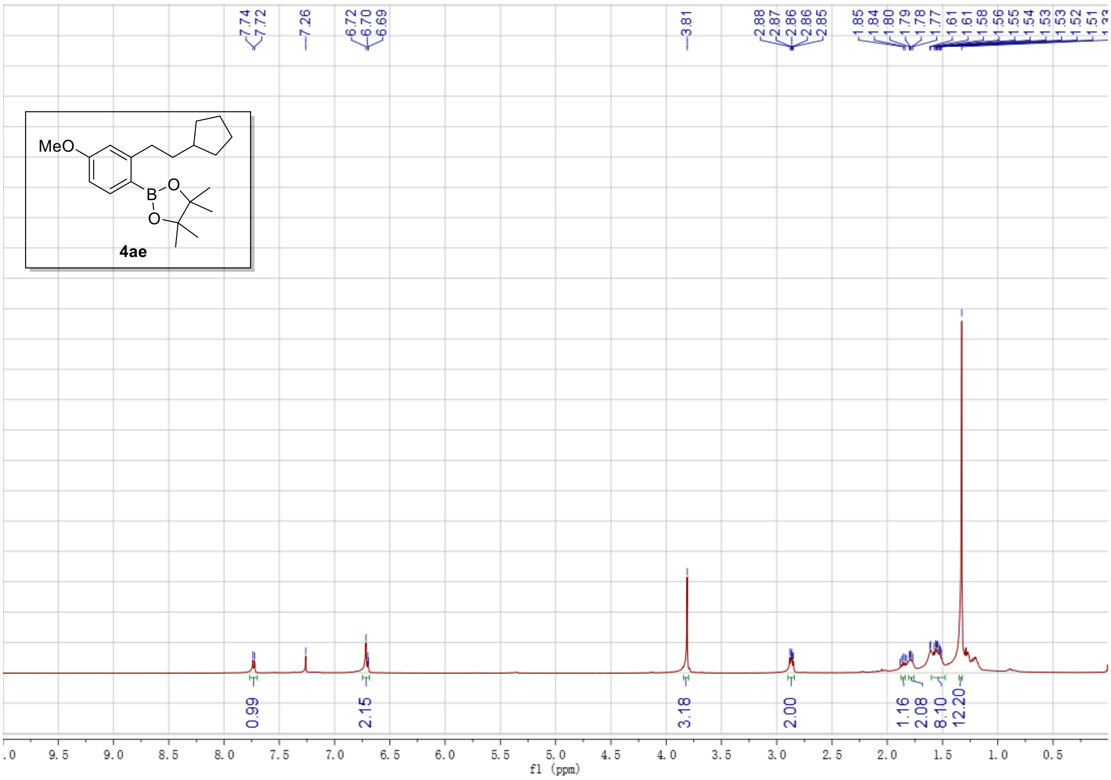

Supplementary Figure 154. ^{1}H NMR spectrum of **4ae**.

^{13}C spectrum (126 MHz, room temperature, $CDCl_3$) of (**4ae**)

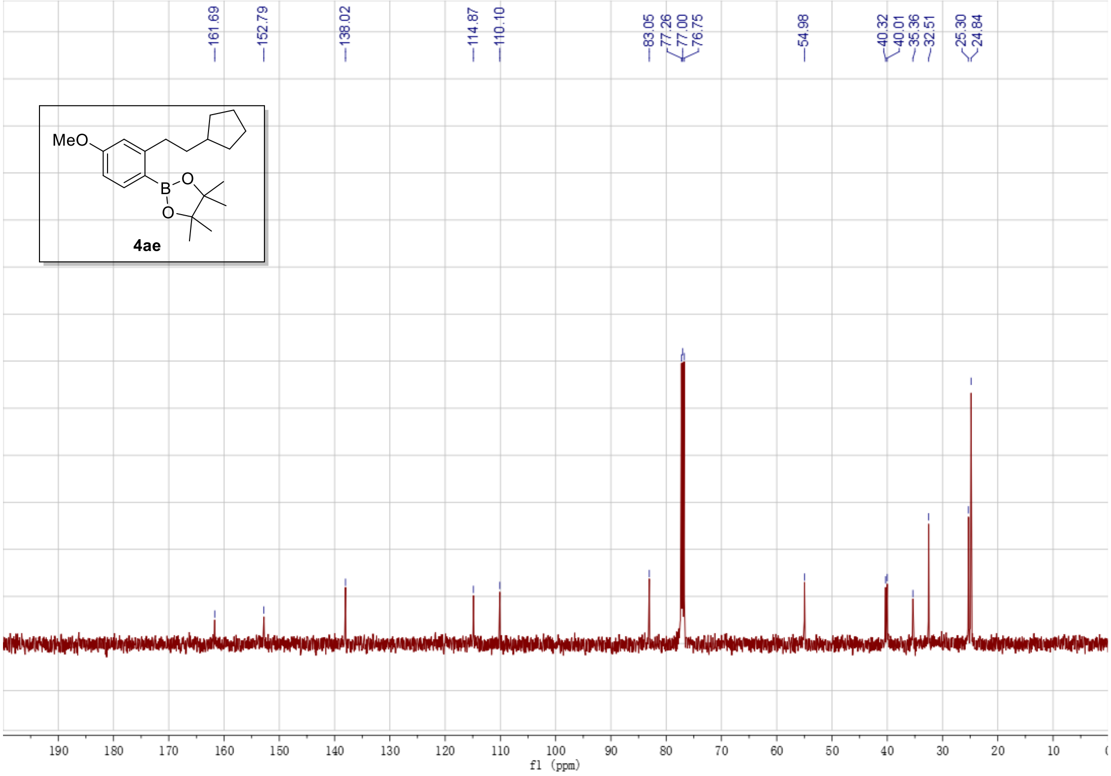

Supplementary Figure 155. ^{13}C NMR spectrum of **4ae**.

^{11}B spectrum (160 MHz, room temperature, $CDCl_3$) of (**4ae**)

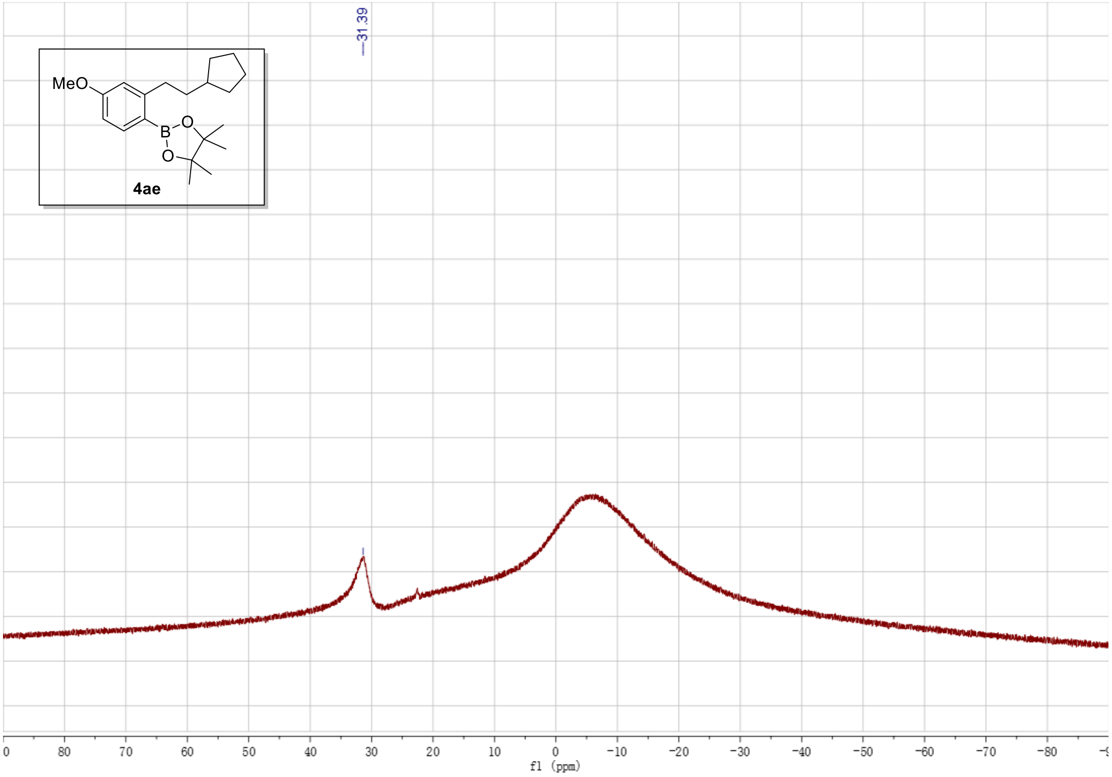

Supplementary Figure 156. ^{11}B spectrum of **4ae**.

2-(2-(2-cyclohexylethyl)-4-methoxyphenyl)-4,4,5,5-tetramethyl-1,3,2-dioxaborolane (4af)

^{1}H spectrum (500 MHz, room temperature, $CDCl_3$) of (**4af**)

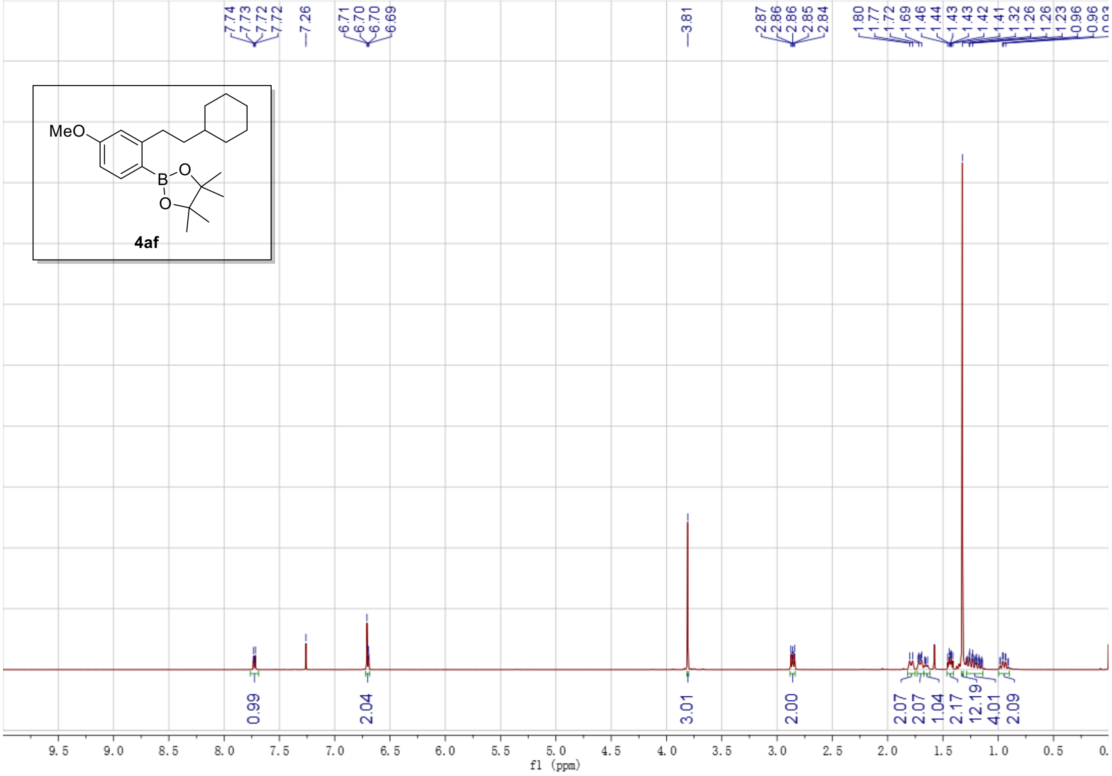

Supplementary Figure 157. ^{1}H NMR spectrum of **4af**.

^{13}C spectrum (126 MHz, room temperature, $CDCl_3$) of (**4af**)

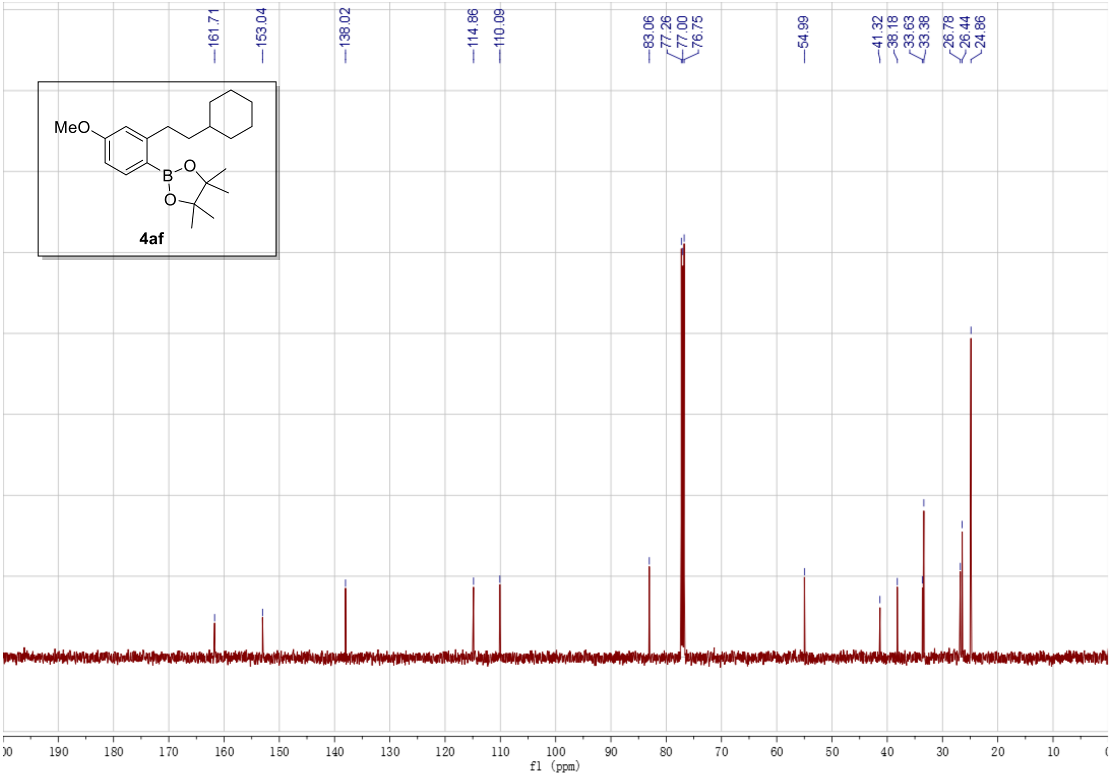

Supplementary Figure 158. ^{13}C NMR spectrum of **4af**.

^{11}B spectrum (128 MHz, room temperature, $CDCl_3$) of (**4af**)

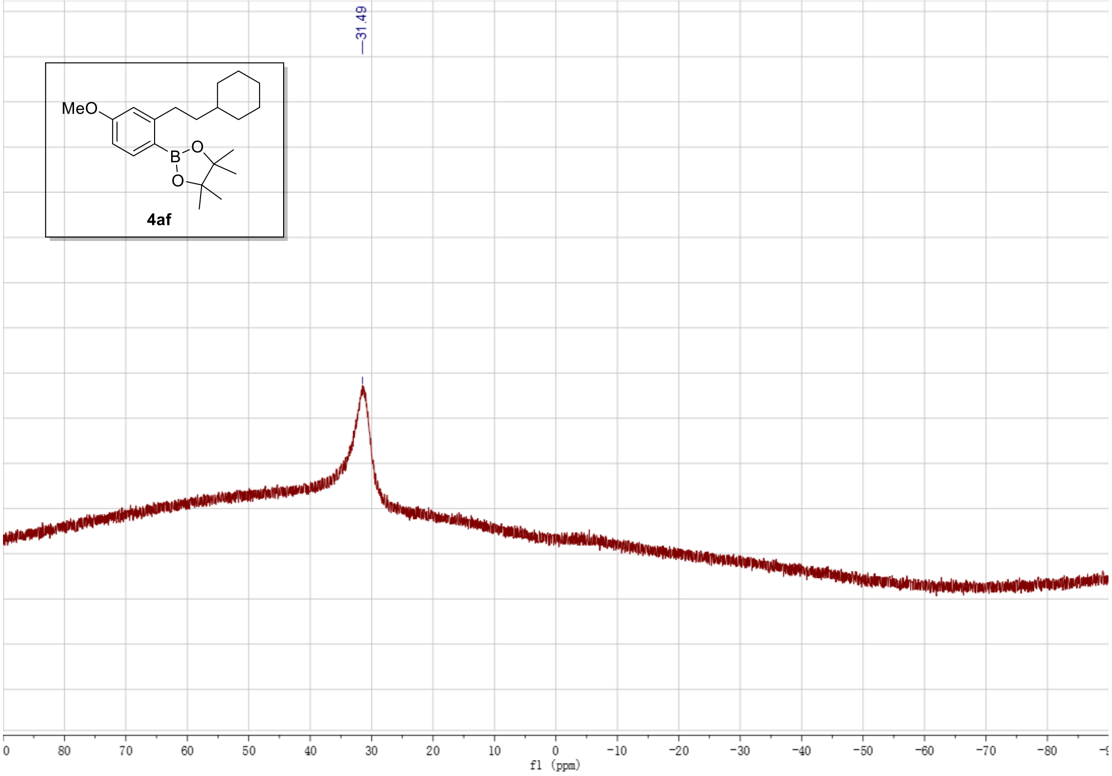

Supplementary Figure 159. ^{11}B spectrum of **4af**.

2-(2-(2-cycloheptylethyl)-4-methoxyphenyl)-4,4,5,5-tetramethyl-1,3,2-dioxaborolane (4ag)

^{1}H spectrum (400 MHz, room temperature, $CDCl_3$) of (**4ag**)

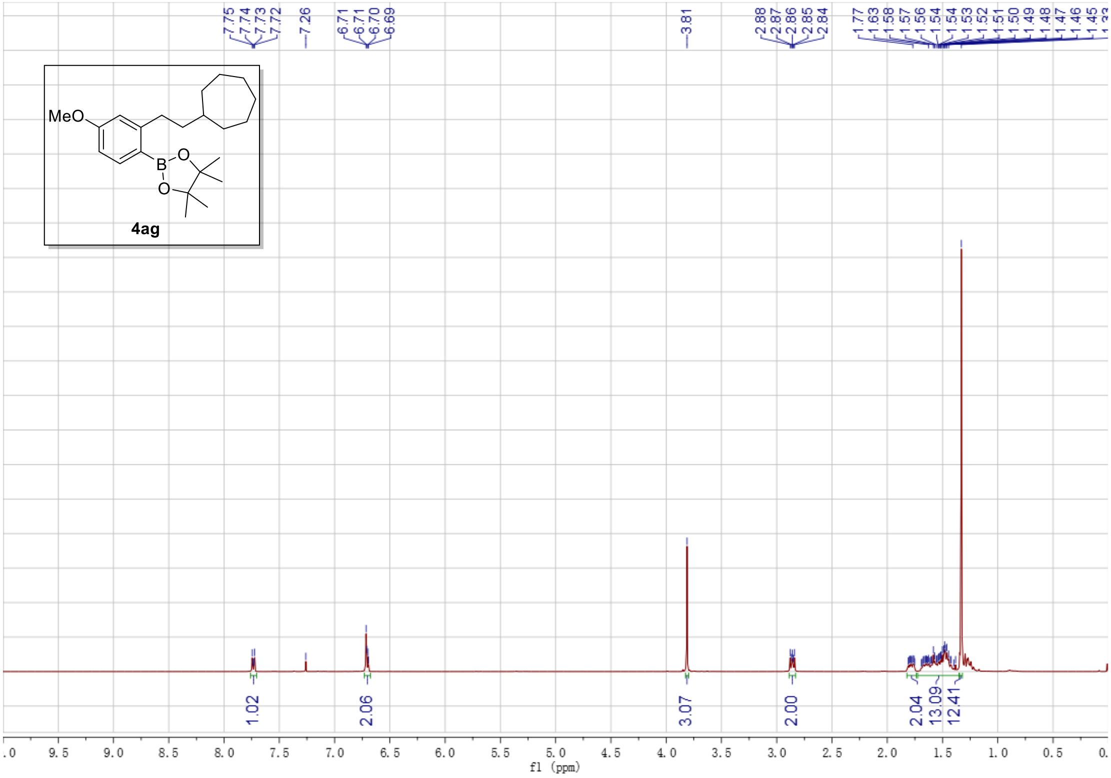

Supplementary Figure 160. ^{1}H NMR spectrum of **4ag**.

^{13}C spectrum (126 MHz, room temperature, $CDCl_3$) of (**4ag**)

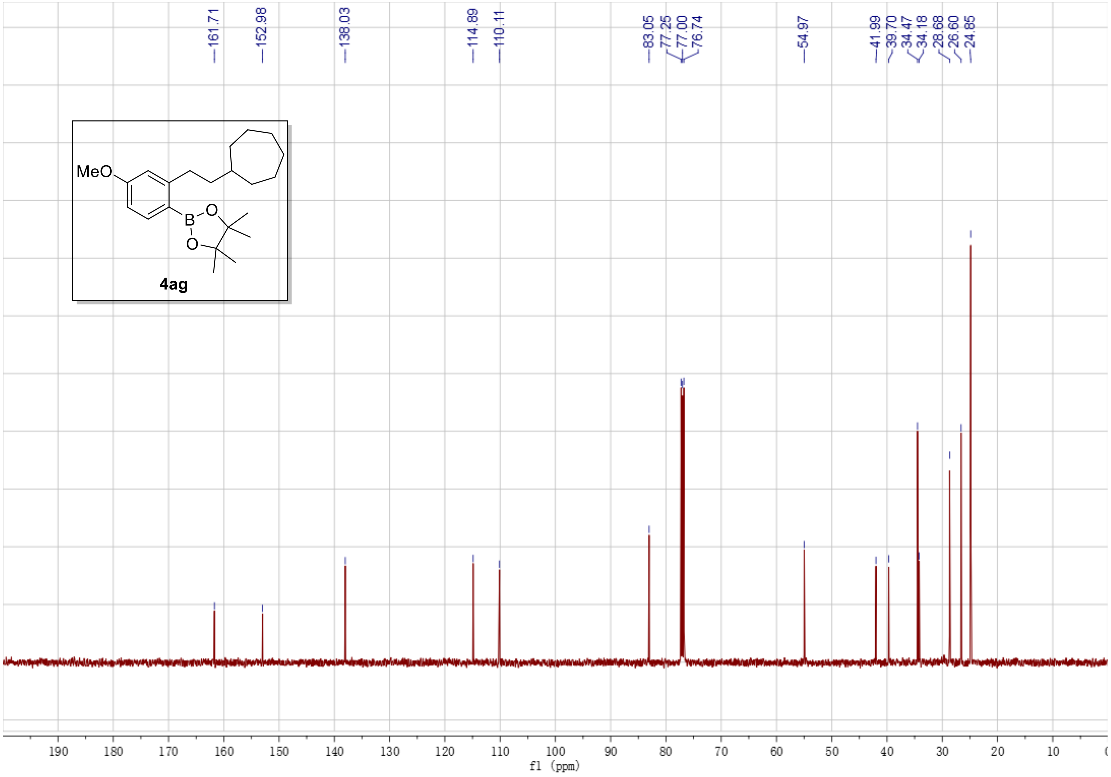

Supplementary Figure 161. ^{13}C NMR spectrum of **4ag**.

^{11}B spectrum (160 MHz, room temperature, CDCl$_3$) of (**4ag**)

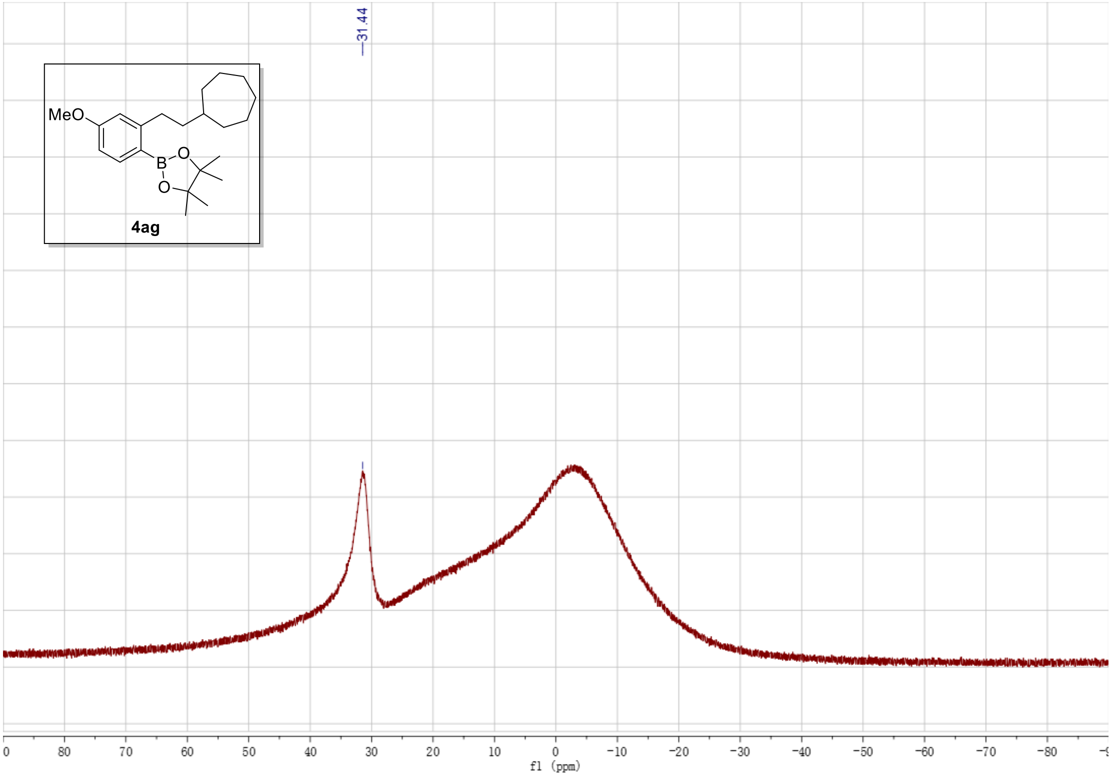

Supplementary Figure 162. ^{11}B spectrum of **4ag**.

4,4,5,5-tetramethyl-2-(2-(2-((2S,4R)-1,2,3,3,4-pentamethylcyclohexyl)ethyl)phenyl)-1,3,2-dioxaborolane (4ah)

^{1}H spectrum (400 MHz, room temperature, CDCl$_3$) of (**4ah**)

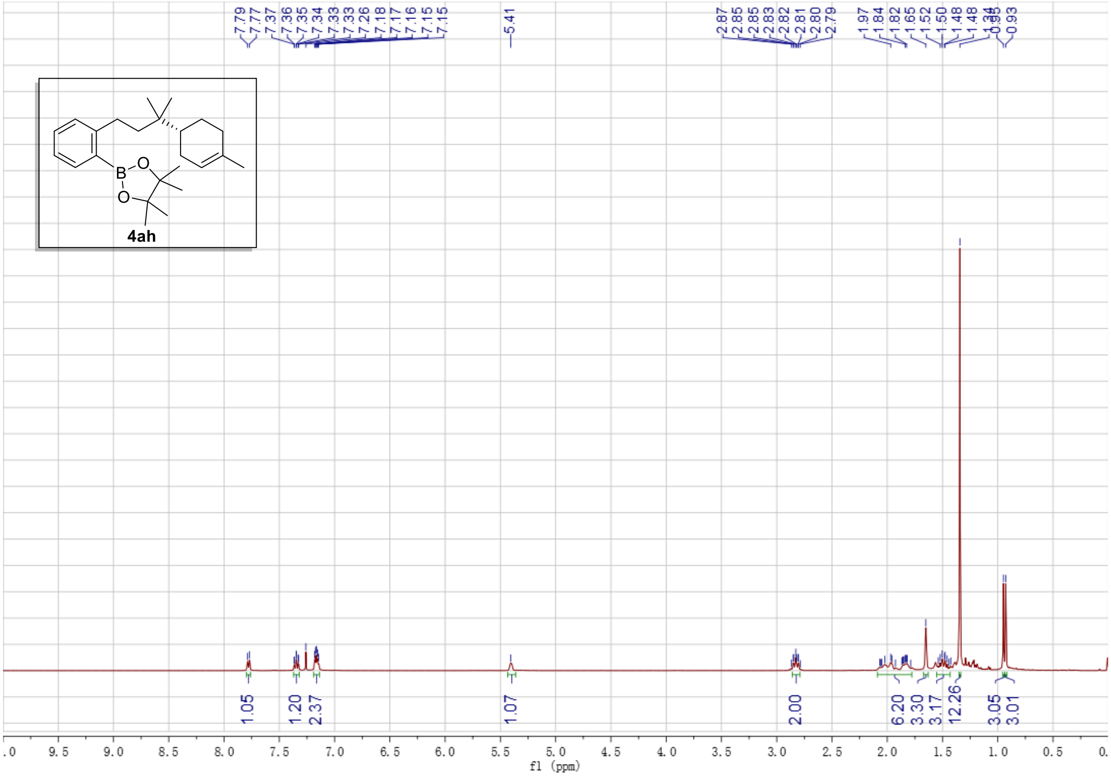

Supplementary Figure 163. ^{1}H NMR spectrum of **4ah**.

^{13}C spectrum (101 MHz, room temperature, $CDCl_3$) of (**4ah**)

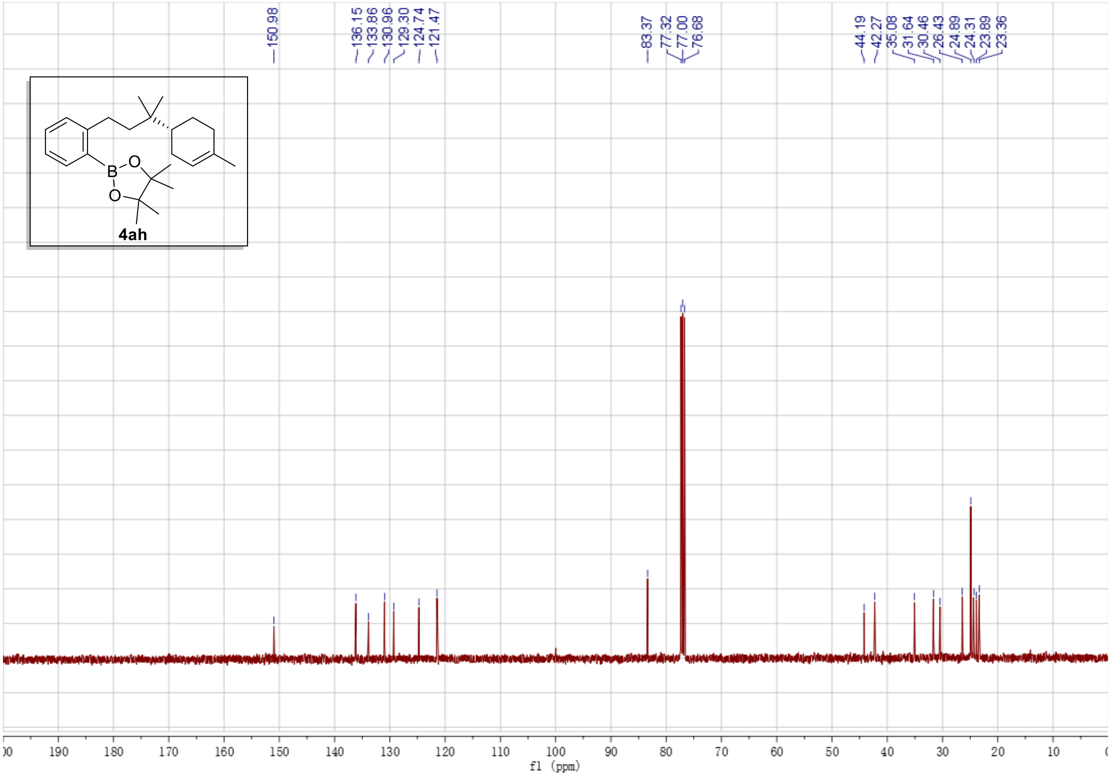

Supplementary Figure 164. ^{13}C NMR spectrum of **4ah**.

^{11}B spectrum (128 MHz, room temperature, $CDCl_3$) of (**4ah**)

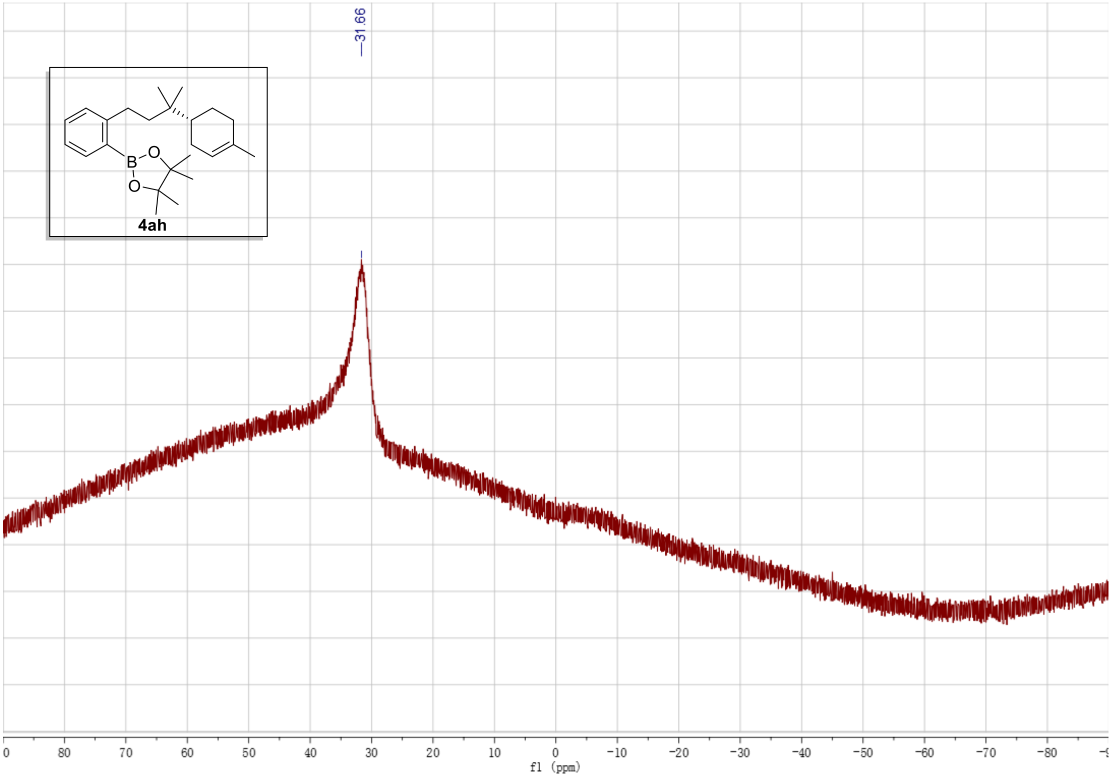

Supplementary Figure 165. ^{11}B spectrum of **4ah**.

2-(2-(6-(2,5-dimethylphenoxy)-3,3-dimethylhexyl)phenyl)-4,4,5,5-tetramethyl-1,3,2-dioxaborolane (4ai)

^{1}H spectrum (400 MHz, room temperature, $CDCl_3$) of (**4ai**)

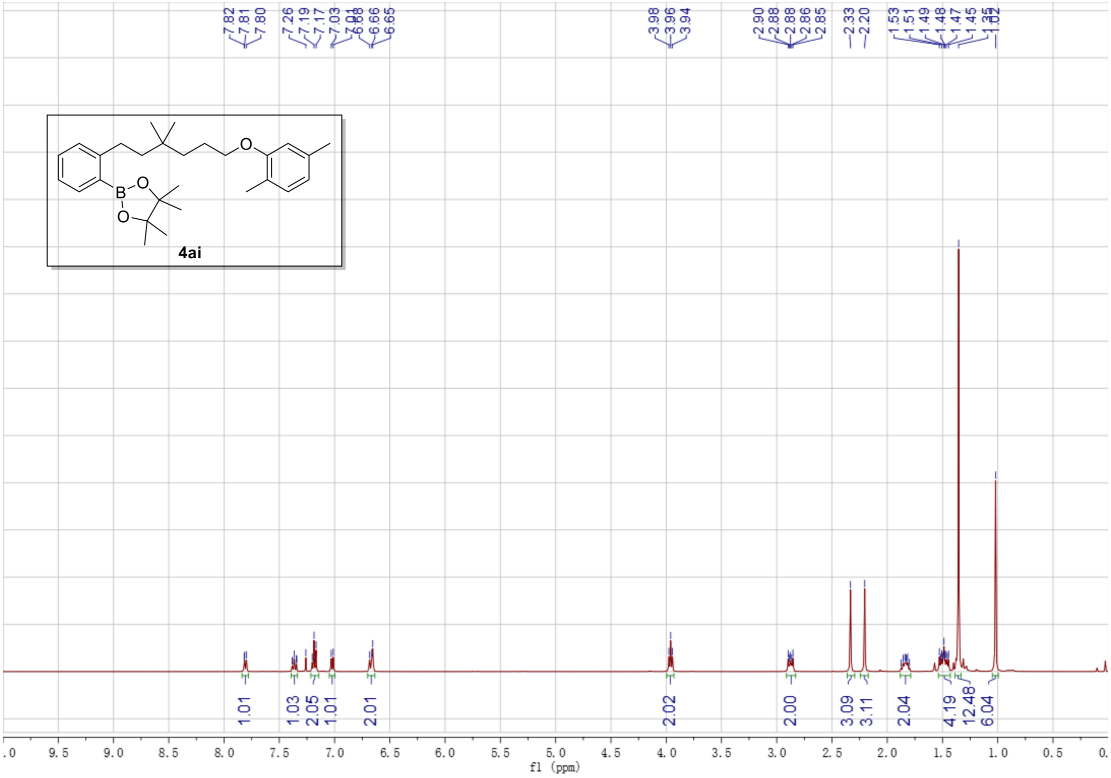

Supplementary Figure 166. ^{1}H NMR spectrum of **4ai**.

^{13}C spectrum (126 MHz, room temperature, $CDCl_3$) of (**4ai**)

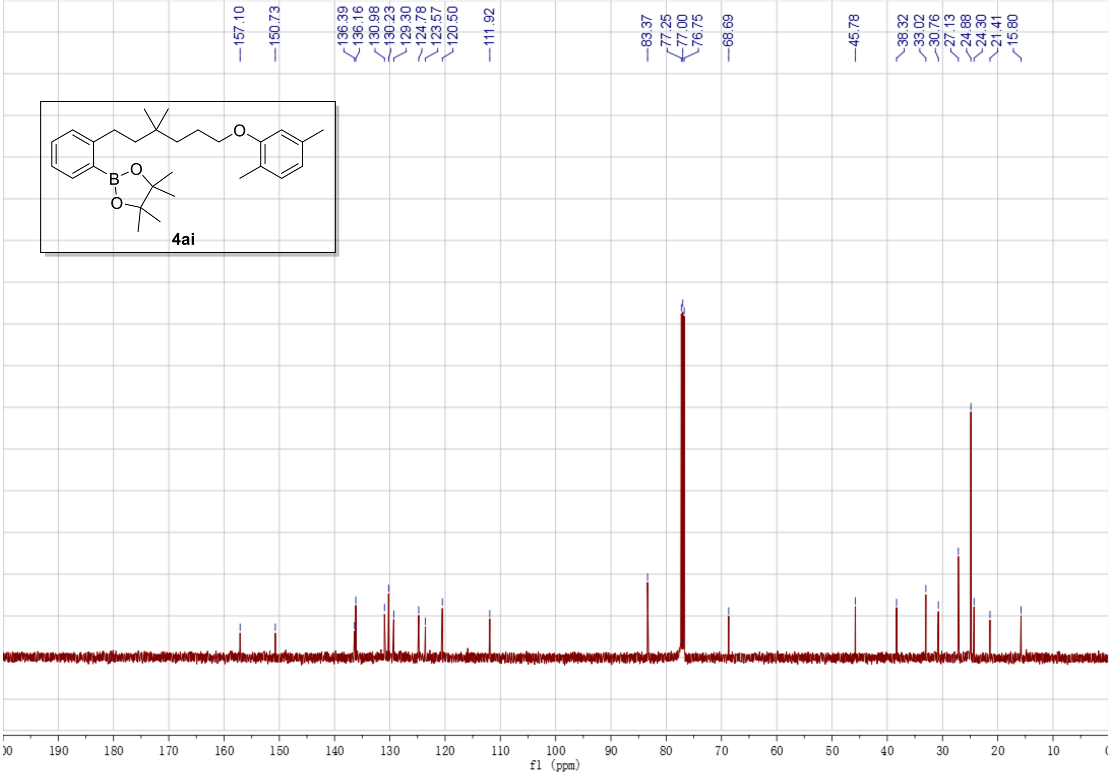

Supplementary Figure 167. ^{13}C NMR spectrum of **4ai**.

^{11}B spectrum (128 MHz, room temperature, $CDCl_3$) of (**4ai**)

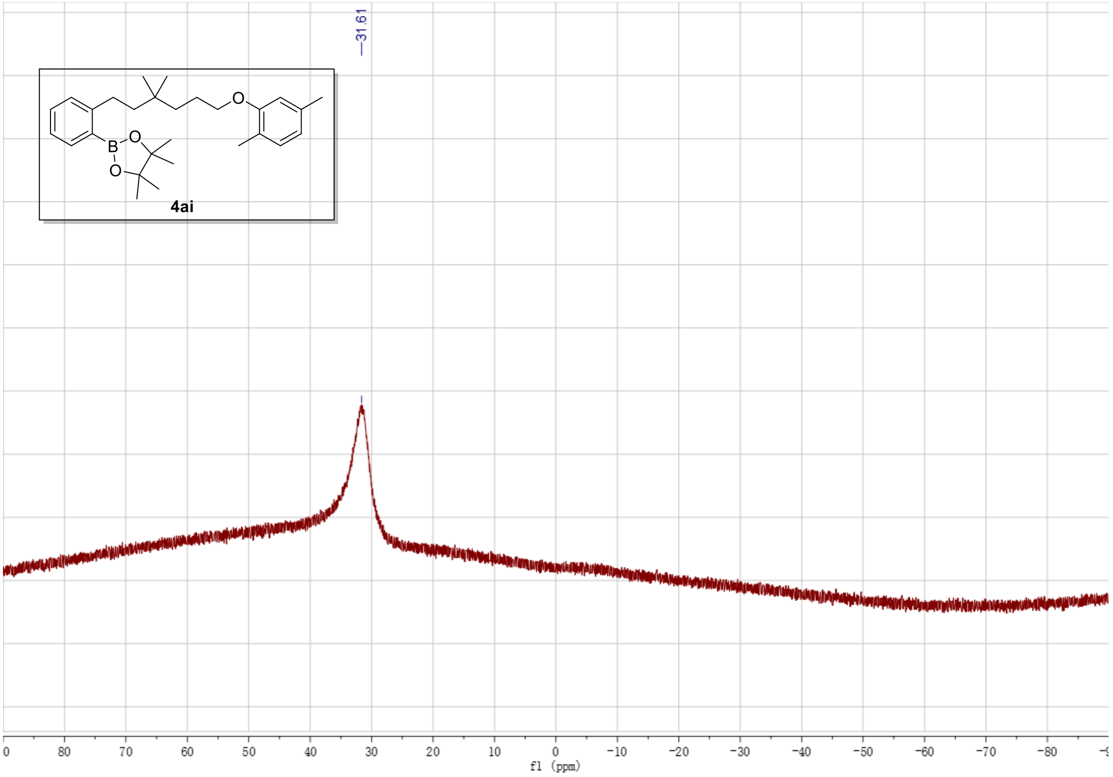

Supplementary Figure 168. ^{11}B spectrum of **4ai**.

2-(2-(5-(benzo[d][1,3]dioxol-5-yl)-3,3-dimethylpentyl)phenyl)-4,4,5,5-tetramethyl-1,3,2-dioxaborolane (4aj)

^{1}H spectrum (500 MHz, room temperature,$CDCl_3$) of (**4aj**)

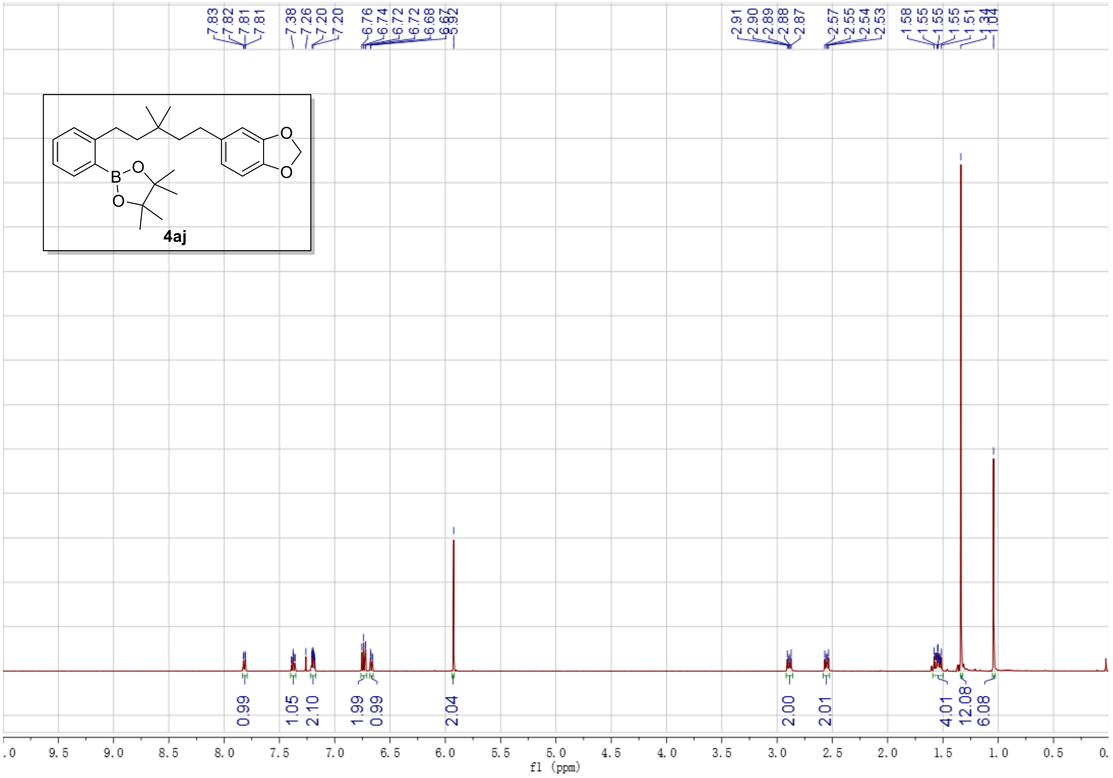

Supplementary Figure 169. ^{1}H NMR spectrum of **4aj**.

^{13}C spectrum (126 MHz, room temperature, $CDCl_3$) of (**4aj**)

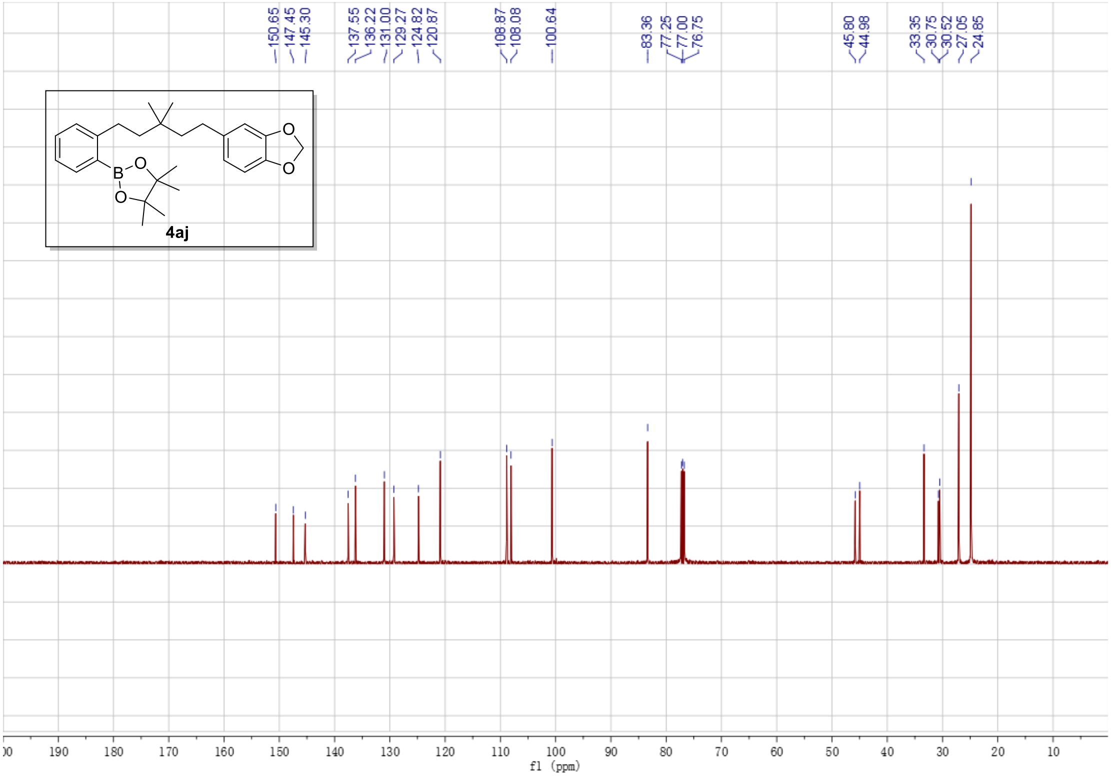

Supplementary Figure 170. ^{13}C NMR spectrum of **4aj**.

^{11}B spectrum (128 MHz, room temperature, $CDCl_3$) of (**4aj**)

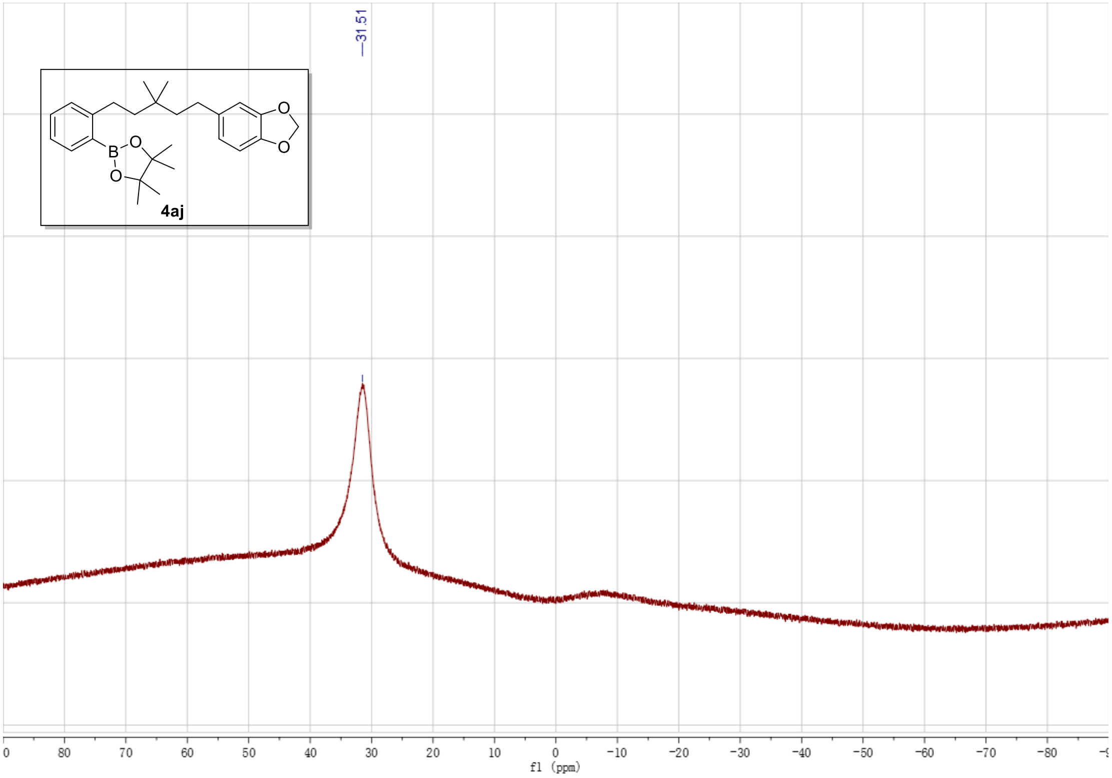

Supplementary Figure 171. ^{11}B spectrum of **4aj**.

2-(4-(4-(4-isobutylphenyl)-3,3-dimethylpentyl)phenyl)-4,4,5,5-tetramethyl-1,3,2-dioxaborolane (4ak)

^{1}H spectrum (500 MHz, room temperature, $CDCl_3$) of (**4ak**)

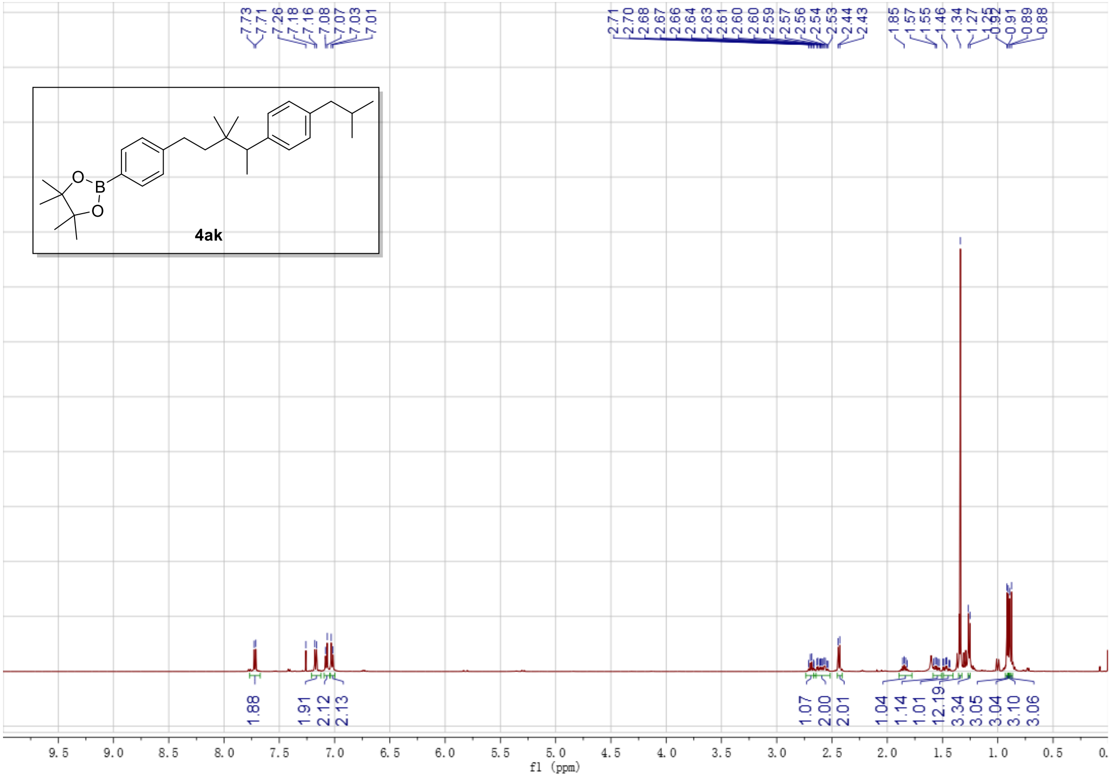

Supplementary Figure 172. ^{1}H NMR spectrum of **4ak**.

^{13}C spectrum (126 MHz, room temperature, $CDCl_3$) of (**4ak**)

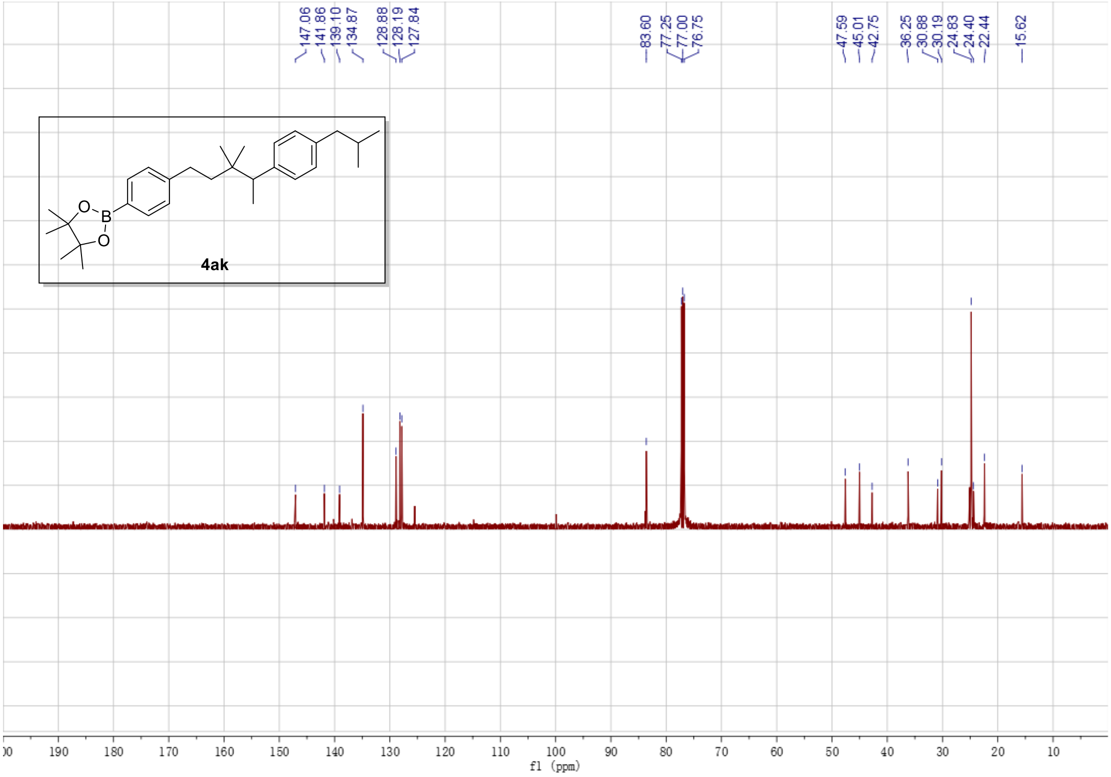

Supplementary Figure 173. ^{13}C NMR spectrum of **4ak**.

^{11}B spectrum (128 MHz, room temperature, $CDCl_3$) of (**4ak**)

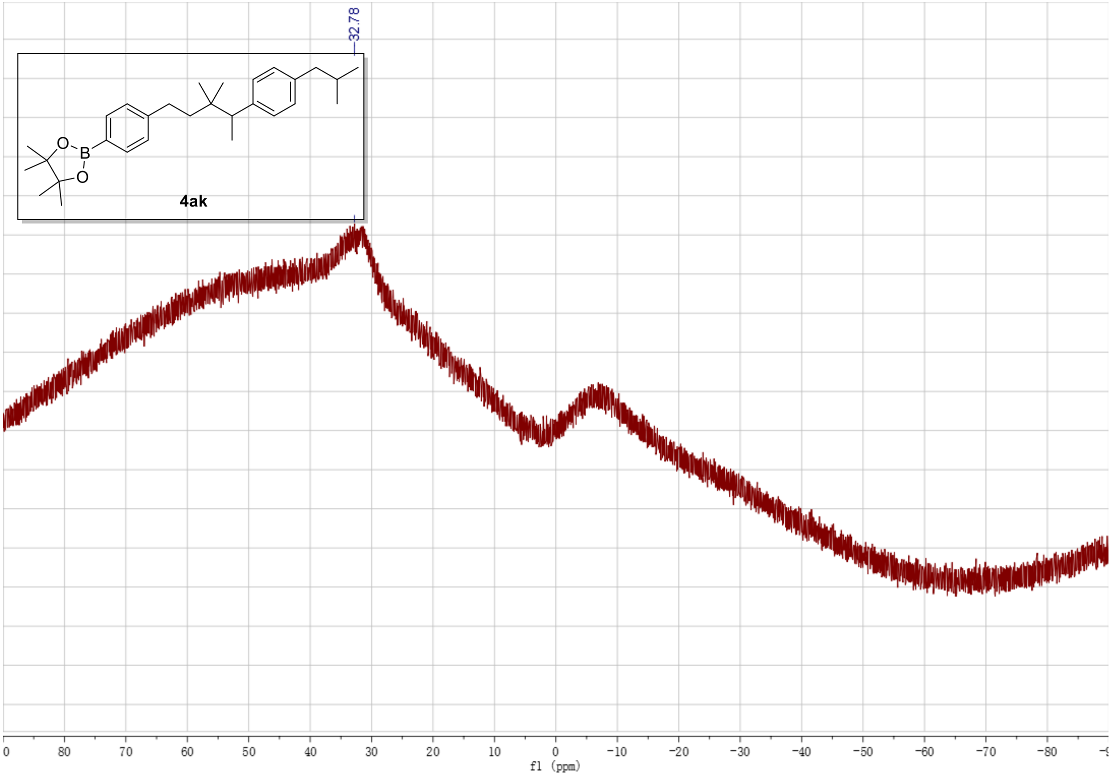

Supplementary Figure 174. ^{11}B spectrum of **4ak**.

2-(2,2-diethyl-4-(2-(4,4,5,5-tetramethyl-1,3,2-dioxaborolan-2-yl)phenyl)butyl)-4,4,5,5-tetramethyl-1,3,2-dioxaborolane (6a)

^{1}H spectrum (500 MHz, room temperature, $CDCl_3$) of (**6a**)

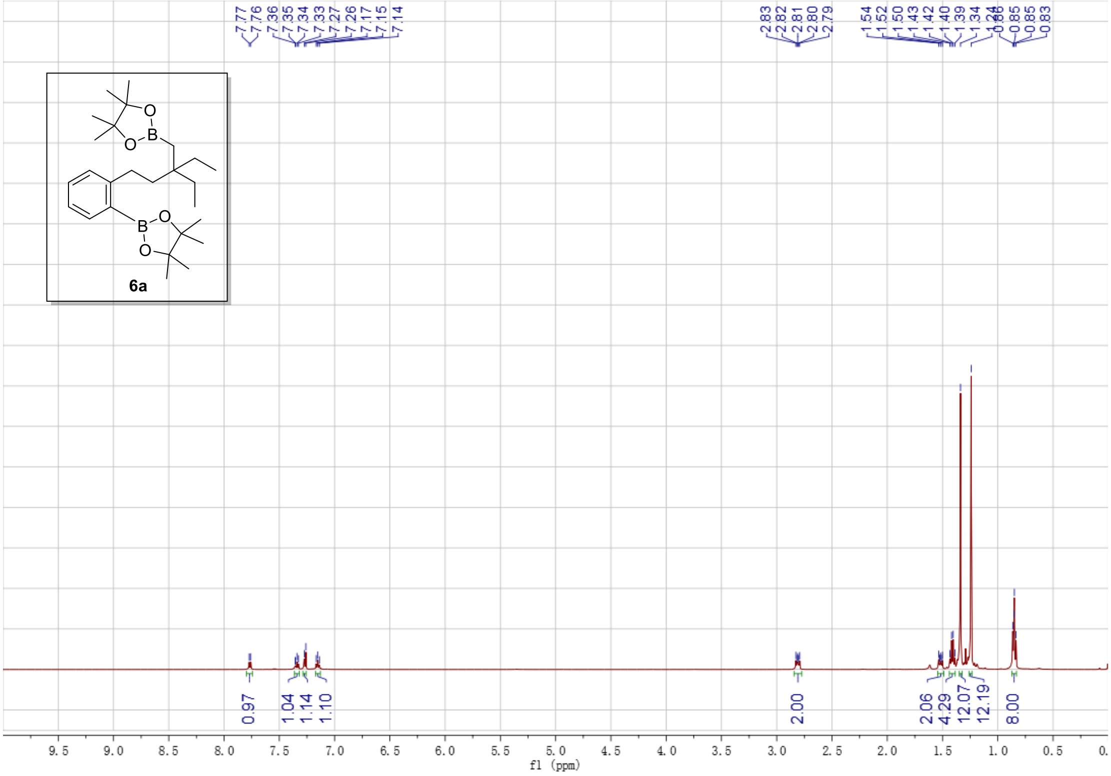

Supplementary Figure 175. ^{1}H NMR spectrum of **6a**.

^{13}C spectrum (126 MHz, room temperature, $CDCl_3$) of (**6a**)

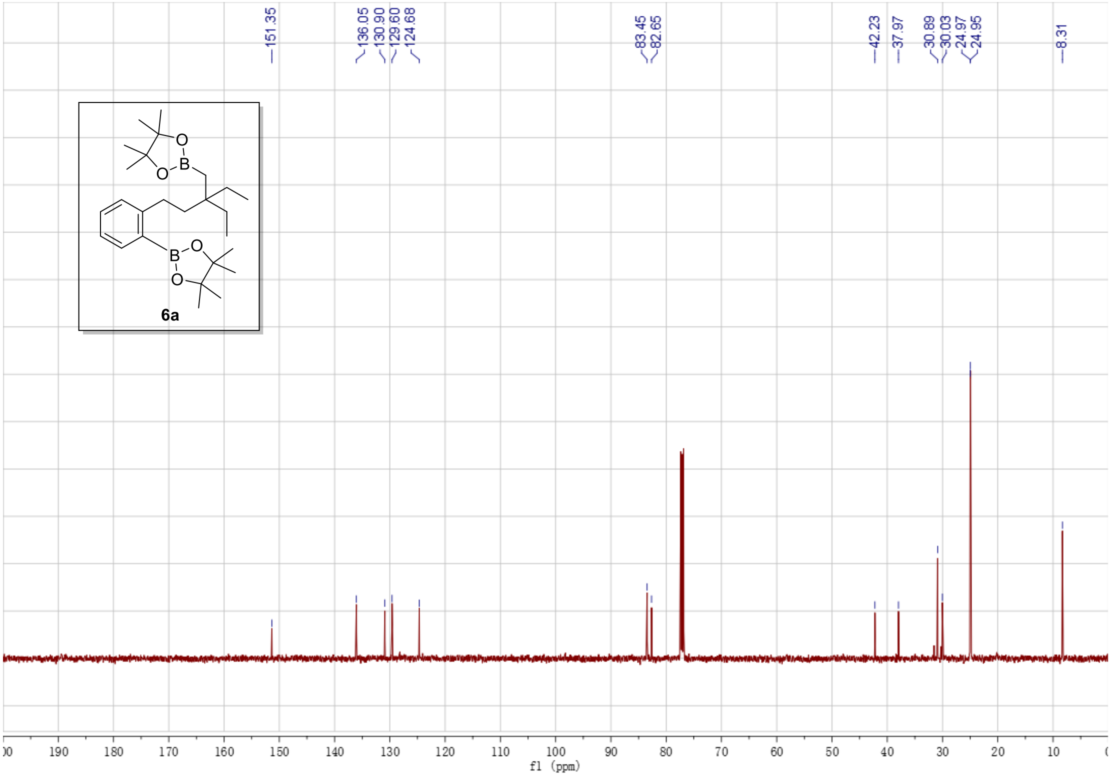

Supplementary Figure 176. ^{13}C NMR spectrum of **6a**.

^{11}B spectrum (160 MHz, room temperature, $CDCl_3$) of (**6a**)

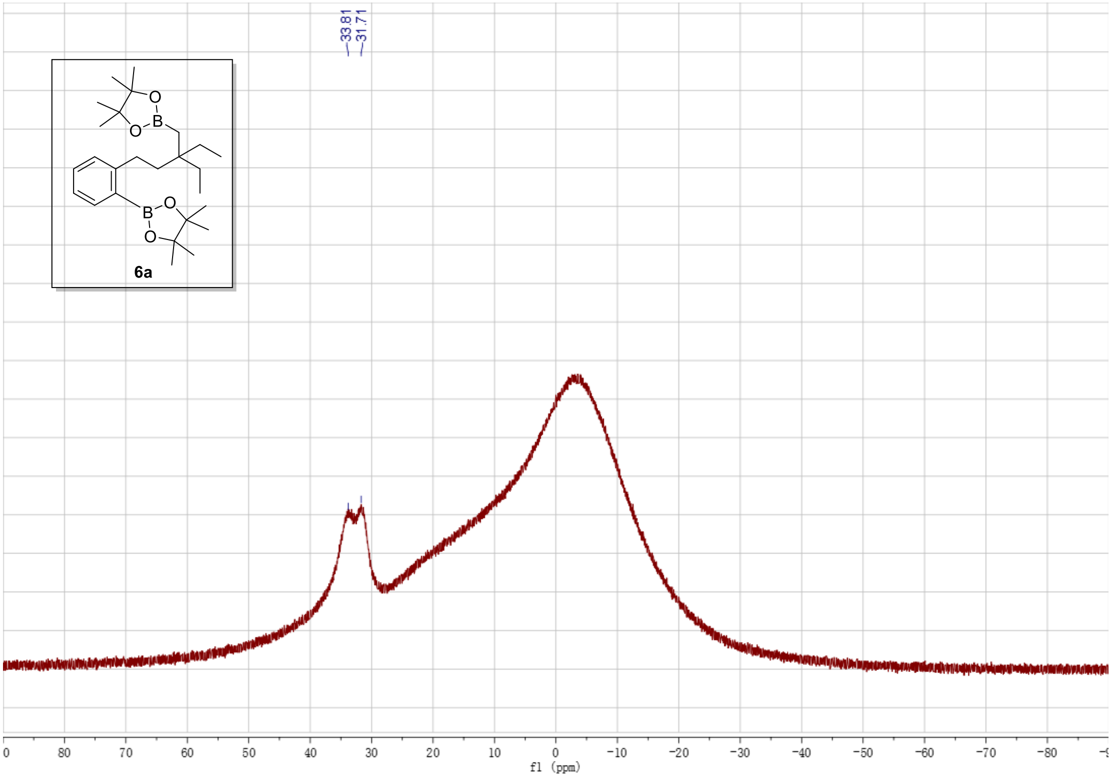

Supplementary Figure 177. ^{11}B spectrum of **6a**.

2-(2-(3,4-dimethyl-3-((4,4,5,5-tetramethyl-1,3,2-dioxaborolan-2-yl)methyl)pentyl)phenyl)-4,4,5,5-tetramethyl-1,3,2-dioxaborolane (6b)

^{1}H spectrum (500 MHz, room temperature, $CDCl_3$) of (**6b**)

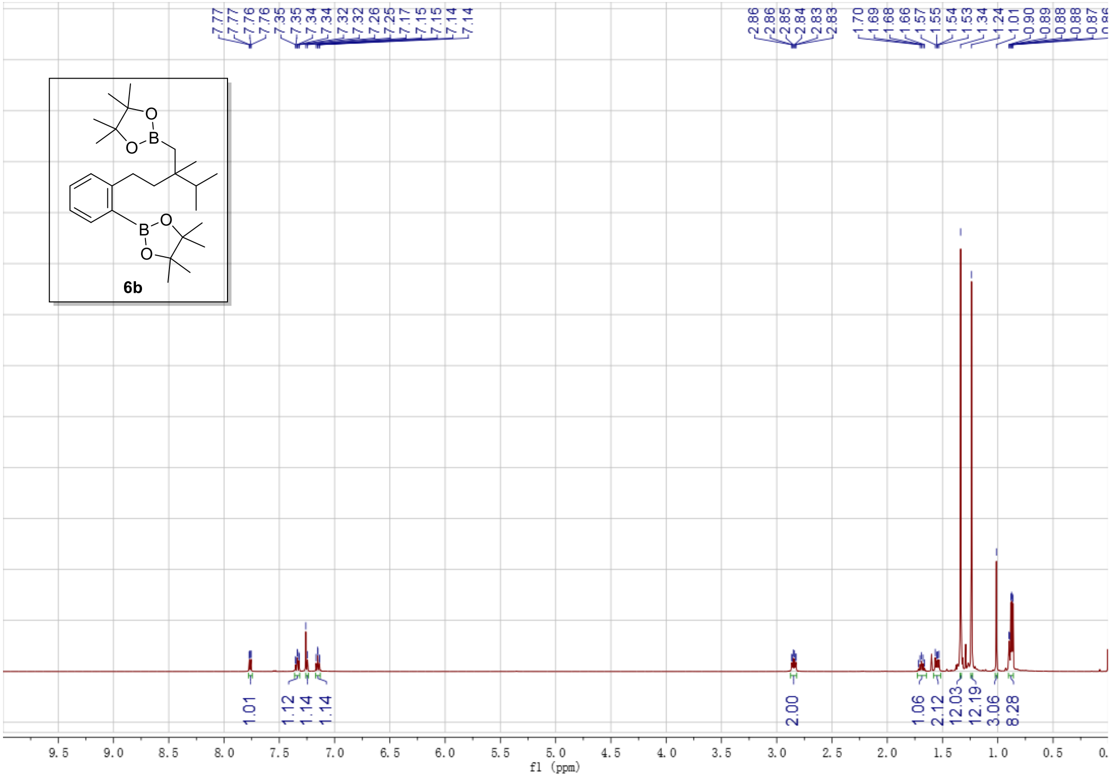

Supplementary Figure 178. ^{1}H NMR spectrum of **6b**.

^{13}C spectrum (126 MHz, room temperature, $CDCl_3$) of (**6b**)

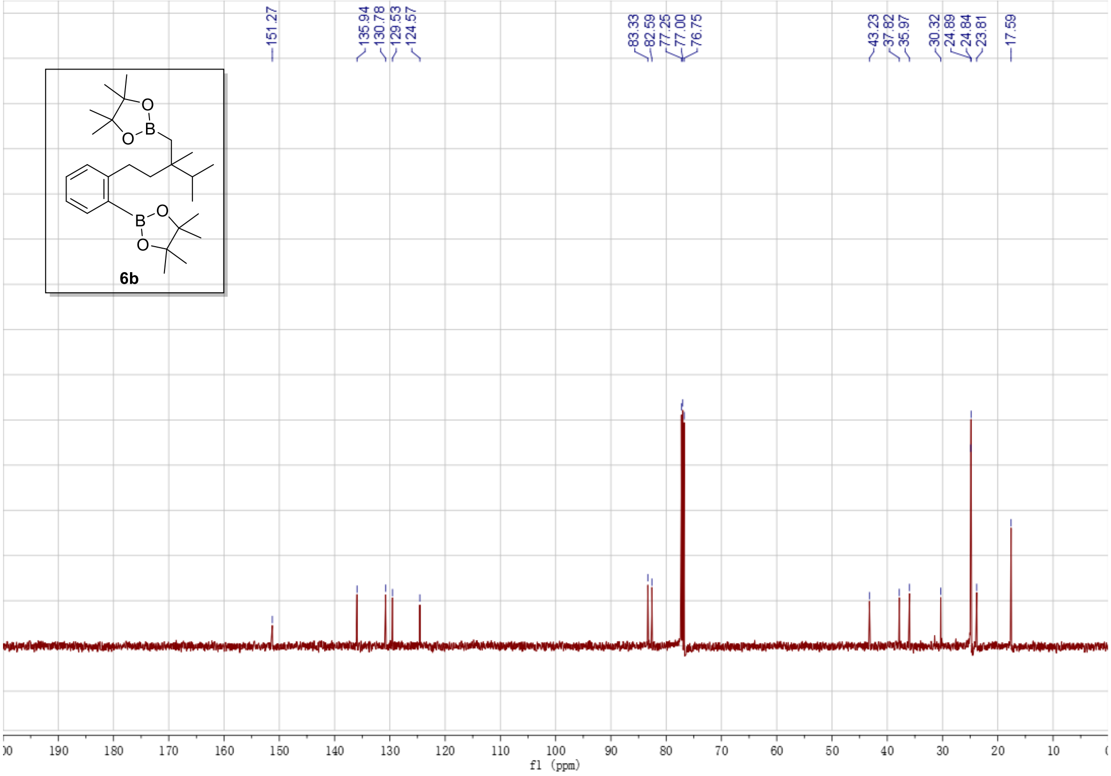

Supplementary Figure 179. ^{13}C NMR spectrum of **6b**.

^{11}B spectrum (128 MHz, room temperature, $CDCl_3$) of (**6b**)

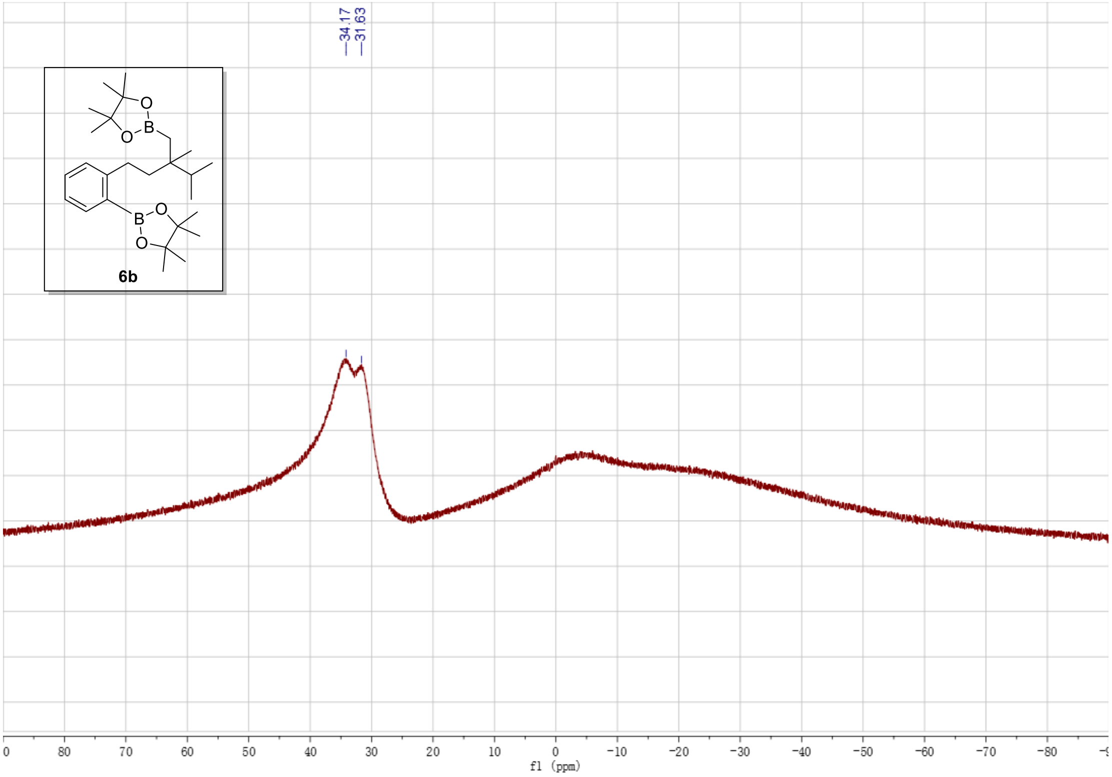

Supplementary Figure 180. ^{11}B spectrum of **6b**.

4,4,5,5-tetramethyl-2-(2-methyl-2-(2-(4,4,5,5-tetramethyl-1,3,2-dioxaborolan-2-yl)phenethyl)pentyl)-1,3,2-dioxaborolane (6c)

^{1}H spectrum (500 MHz, room temperature, $CDCl_3$) of (**6c**)

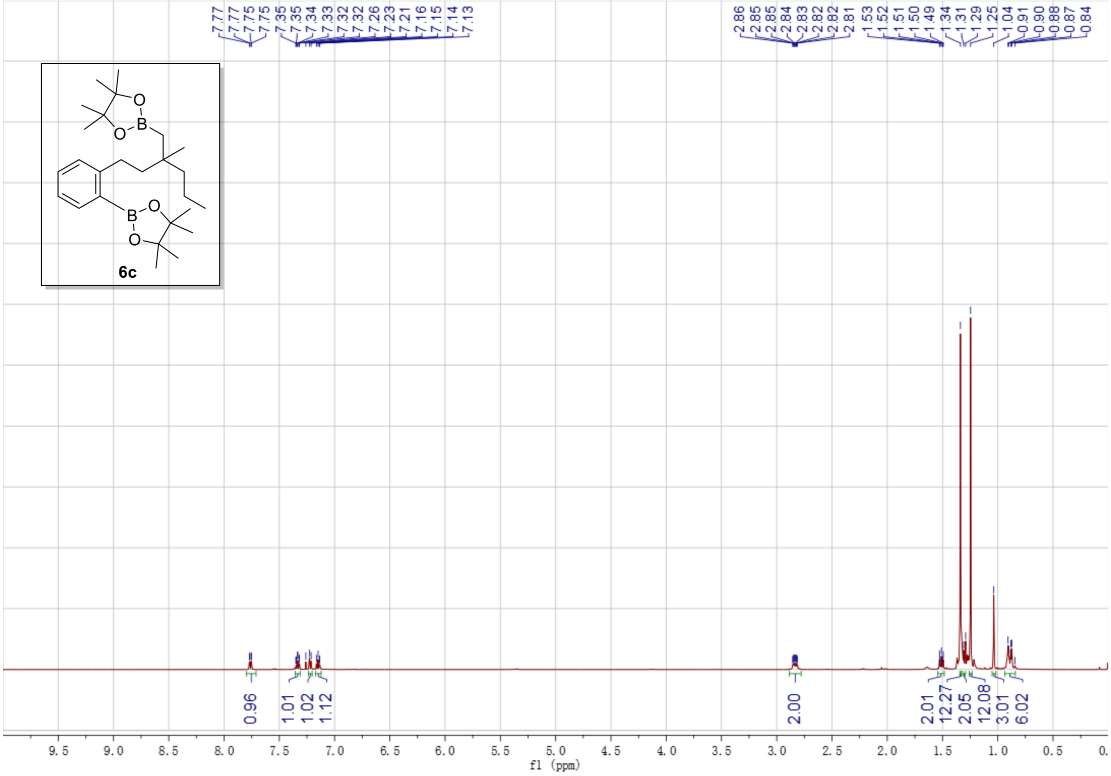

Supplementary Figure 181. ^{1}H NMR spectrum of **6c**.

^{13}C spectrum (126 MHz, room temperature, $CDCl_3$) of (**6c**)

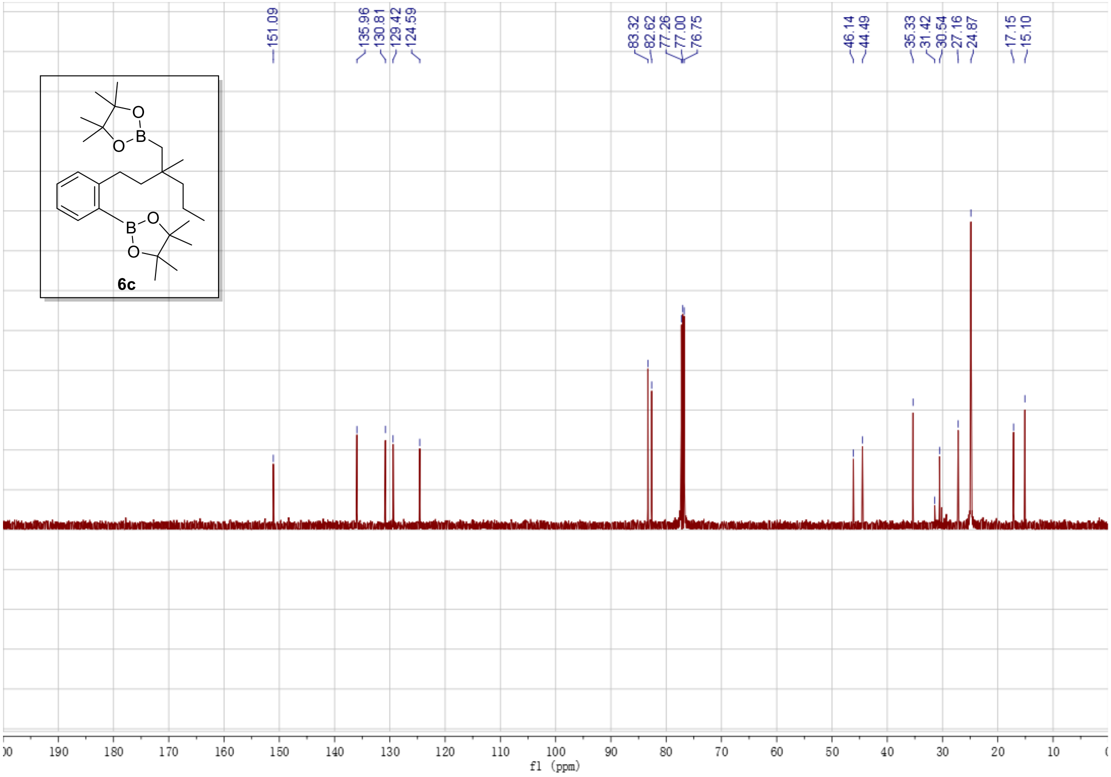

Supplementary Figure 182. ^{13}C NMR spectrum of **6c**.

^{11}B spectrum (160 MHz, room temperature, $CDCl_3$) of (**6c**)

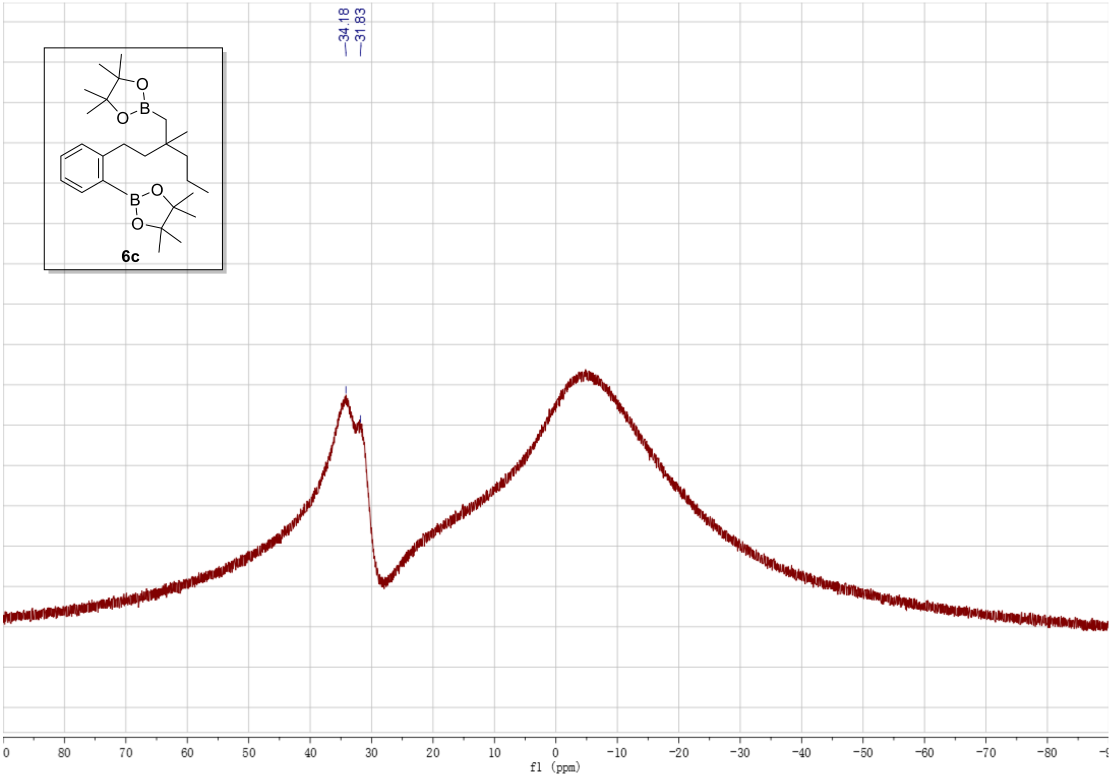

Supplementary Figure 183. ^{11}B spectrum of **6c**.

4,4,5,5-tetramethyl-2-(2-methyl-2-(2-(4,4,5,5-tetramethyl-1,3,2-dioxaborolan-2-yl)phenethyl)hexyl)-1,3,2-dioxaborolane (6d)

^{1}H spectrum (500 MHz, room temperature, $CDCl_3$) of (**6d**)

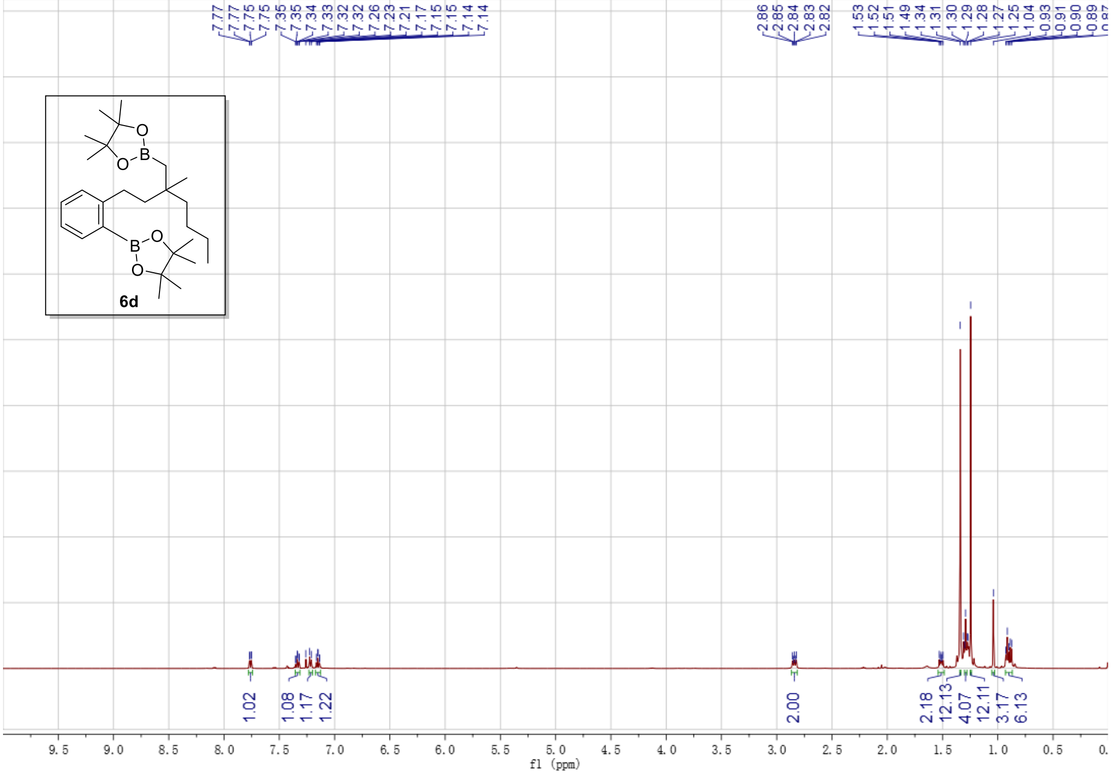

Supplementary Figure 184. ^{1}H NMR spectrum of **6d**.

^{13}C spectrum (126 MHz, room temperature, $CDCl_3$) of (**6d**)

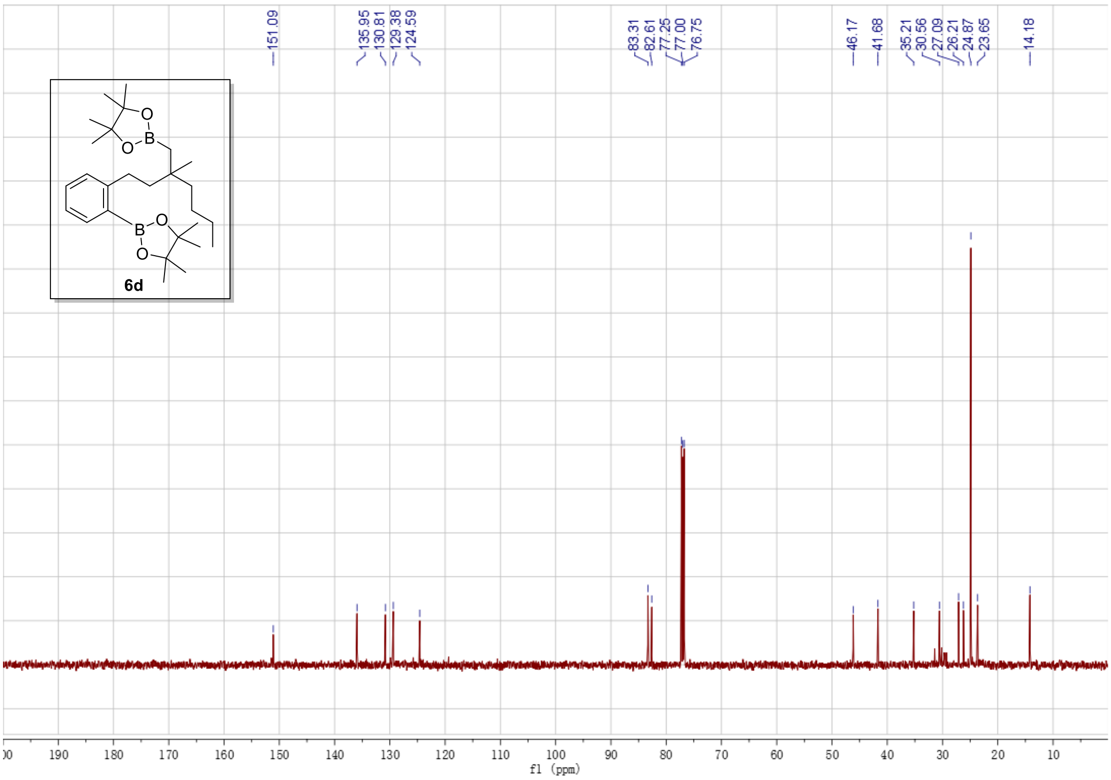

Supplementary Figure 185. ^{13}C NMR spectrum of **6d**.

^{11}B spectrum (160 MHz, room temperature, $CDCl_3$) of (**6d**)

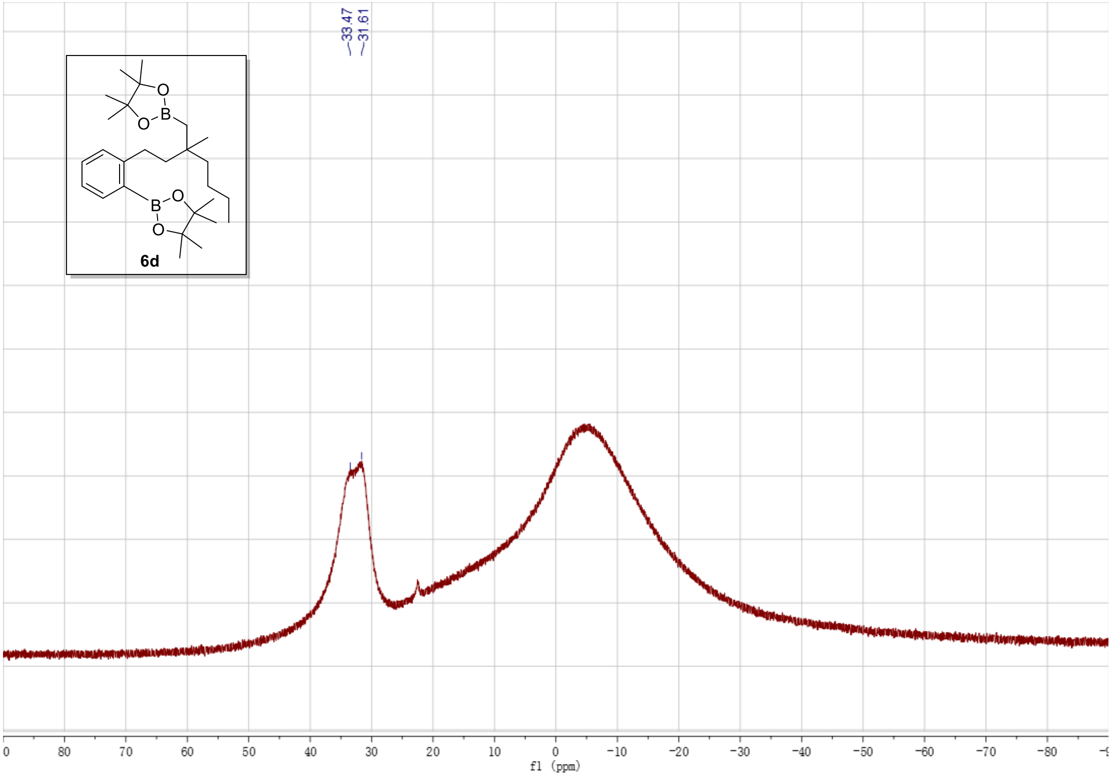

Supplementary Figure 186. ^{11}B spectrum of **6d**.

2-(2-cyclohexyl-2-methyl-4-(2-(4,4,5,5-tetramethyl-1,3,2-dioxaborolan-2-yl)phenyl)butyl)-4,4,5,5-tetramethyl-1,3,2-dioxaborolane (6e)

^{1}H spectrum (500 MHz, room temperature, $CDCl_3$) of (**6e**)

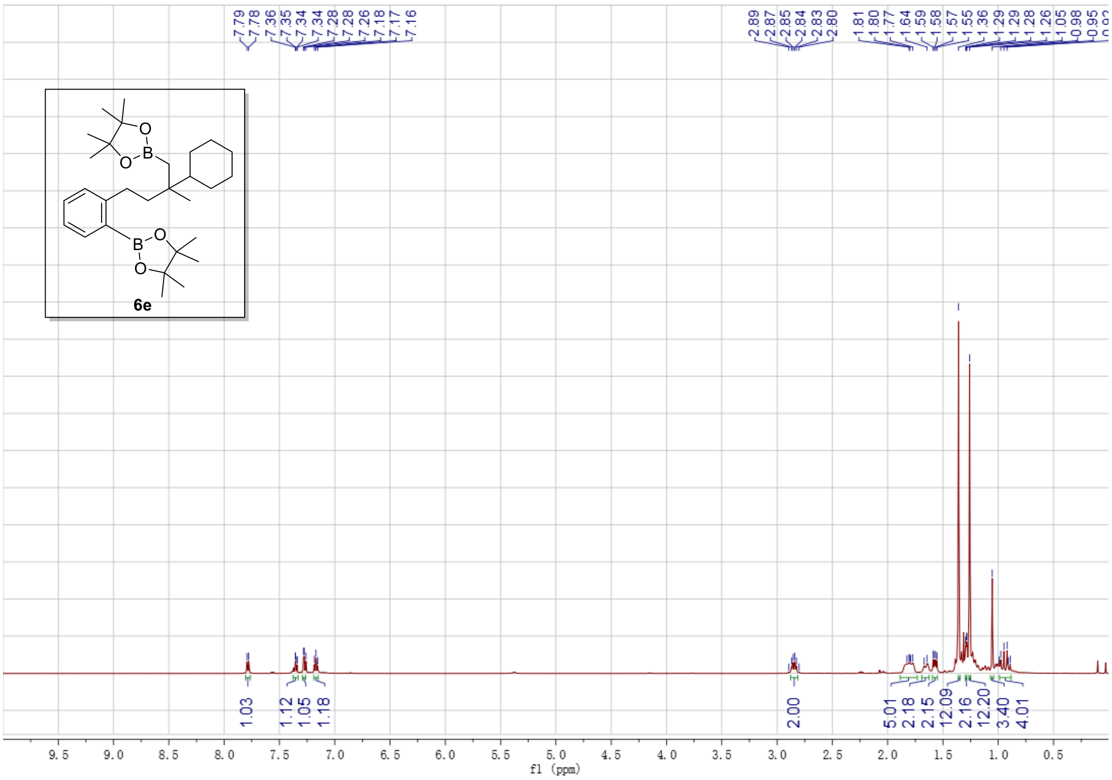

Supplementary Figure 187. ^{1}H NMR spectrum of **6e**.

^{13}C spectrum (126 MHz, room temperature, $CDCl_3$) of (**6e**)

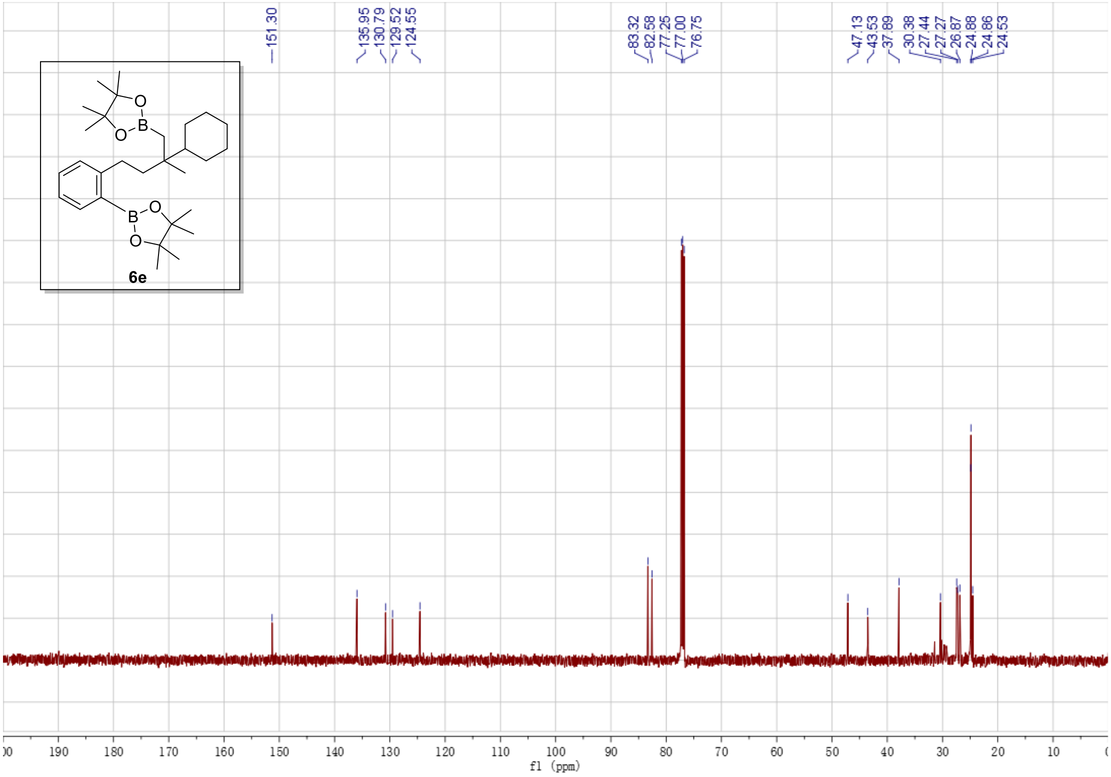

Supplementary Figure 188. ^{13}C NMR spectrum of **6e**.

^{11}B spectrum (160 MHz, room temperature, $CDCl_3$) of (**6e**)

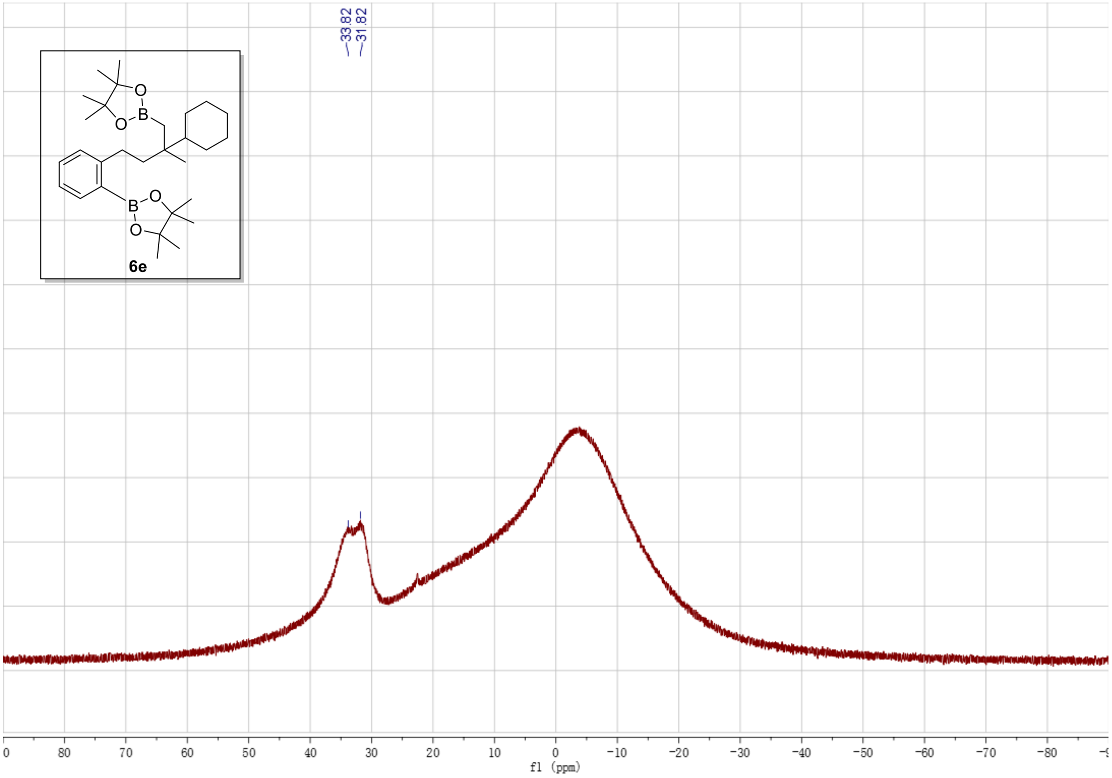

Supplementary Figure 189. ^{11}B spectrum of **6e**.

4,4,5,5-tetramethyl-2-(2-methyl-2-phenethyl-4-(2-(4,4,5,5-tetramethyl-1,3,2-dioxaborolan-2-yl)phenyl)butyl)-1,3,2-dioxaborolane (6f)

^{1}H spectrum (500 MHz, room temperature, $CDCl_3$) of (**6f**)

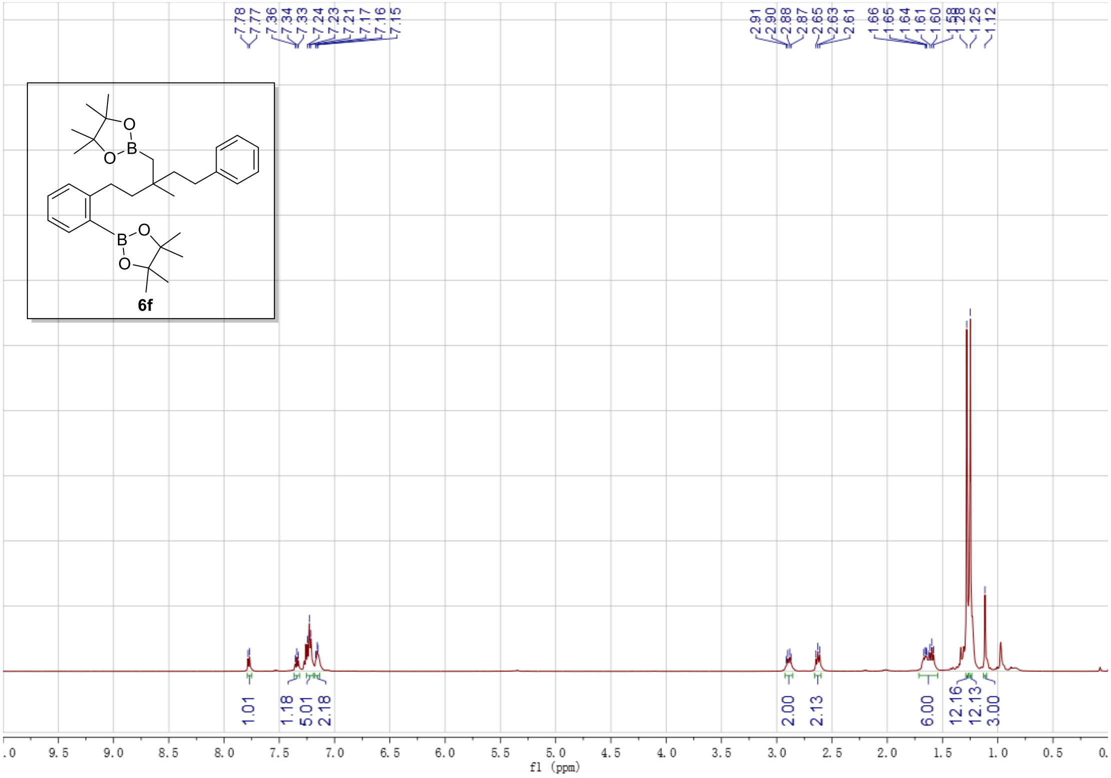

Supplementary Figure 190. ^{1}H NMR spectrum of **6f**.

^{13}C spectrum (126 MHz, room temperature, $CDCl_3$) of (**6f**)

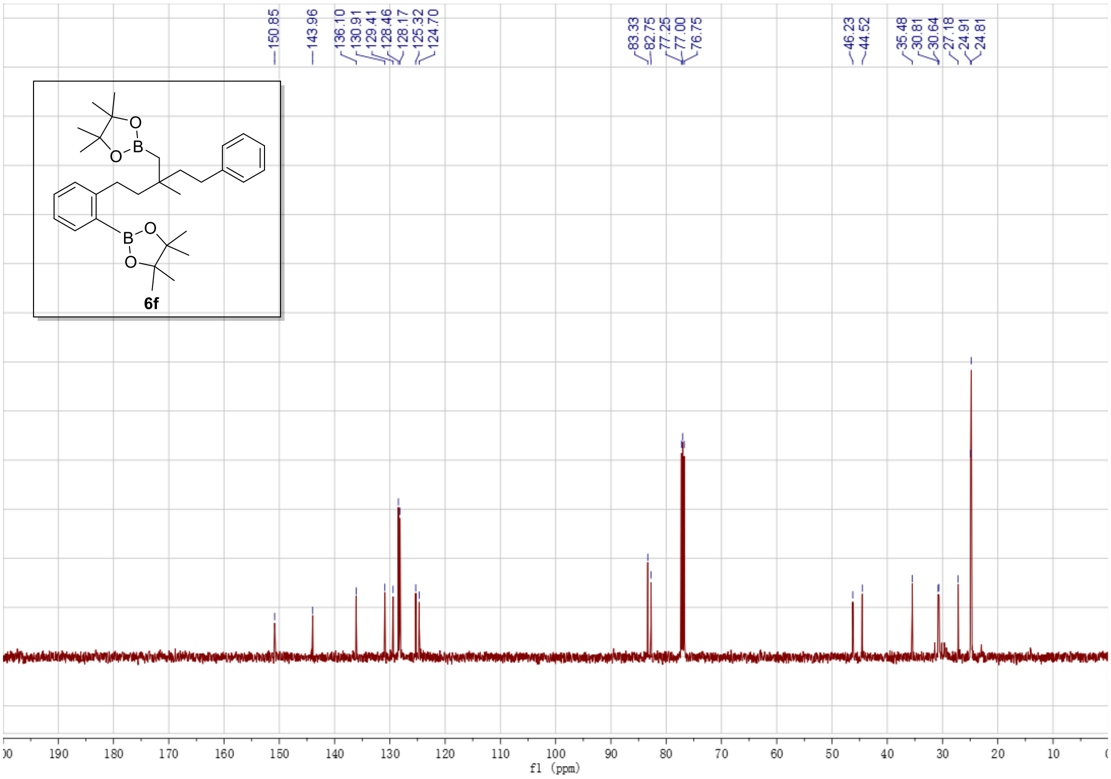

Supplementary Figure 191. ^{13}C NMR spectrum of **6f**.

^{11}B spectrum (160 MHz, room temperature, $CDCl_3$) of (**6f**)

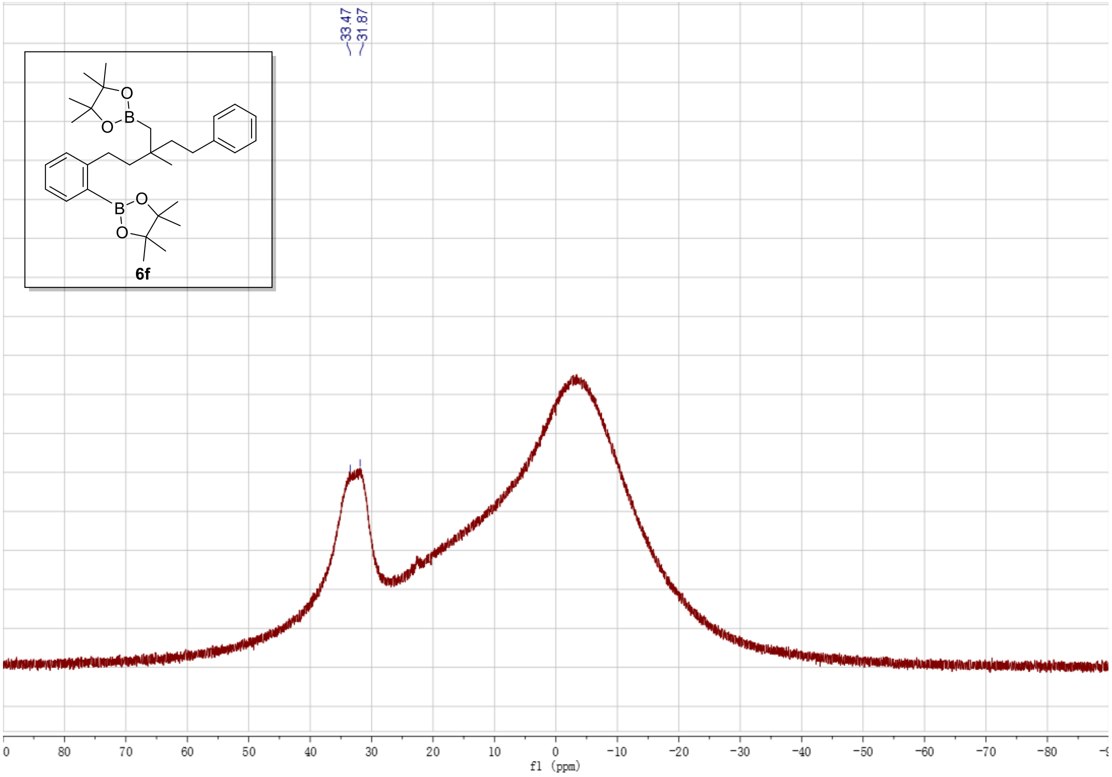

Supplementary Figure 192. ^{11}B spectrum of **6f**.

4,4,5,5-tetramethyl-2-(2,3,3-trimethyl-2-(2-(4,4,5,5-tetramethyl-1,3,2-dioxaborolan-2-yl)phenethyl)butyl)-1,3,2-dioxaborolane (6g)

^{1}H spectrum (500 MHz, room temperature, $CDCl_3$) of (**6g**)

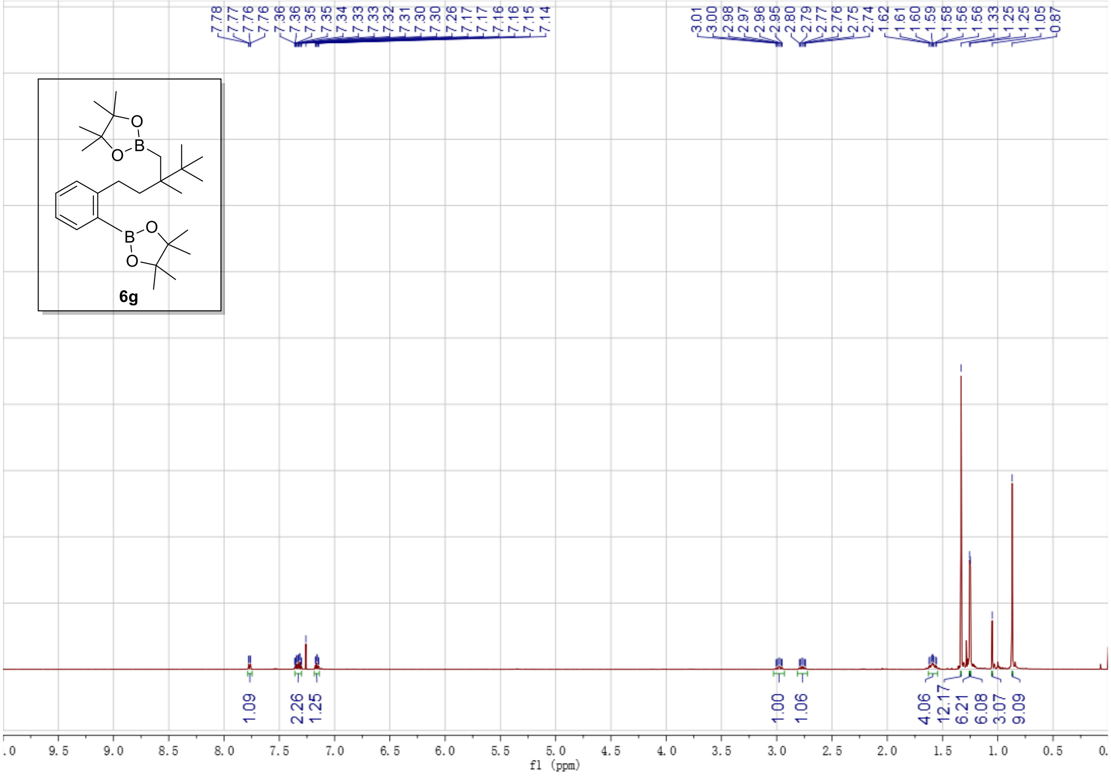

Supplementary Figure 193. ^{1}H NMR spectrum of **6g**.

^{13}C spectrum (126 MHz, room temperature, $CDCl_3$) of (**6g**)

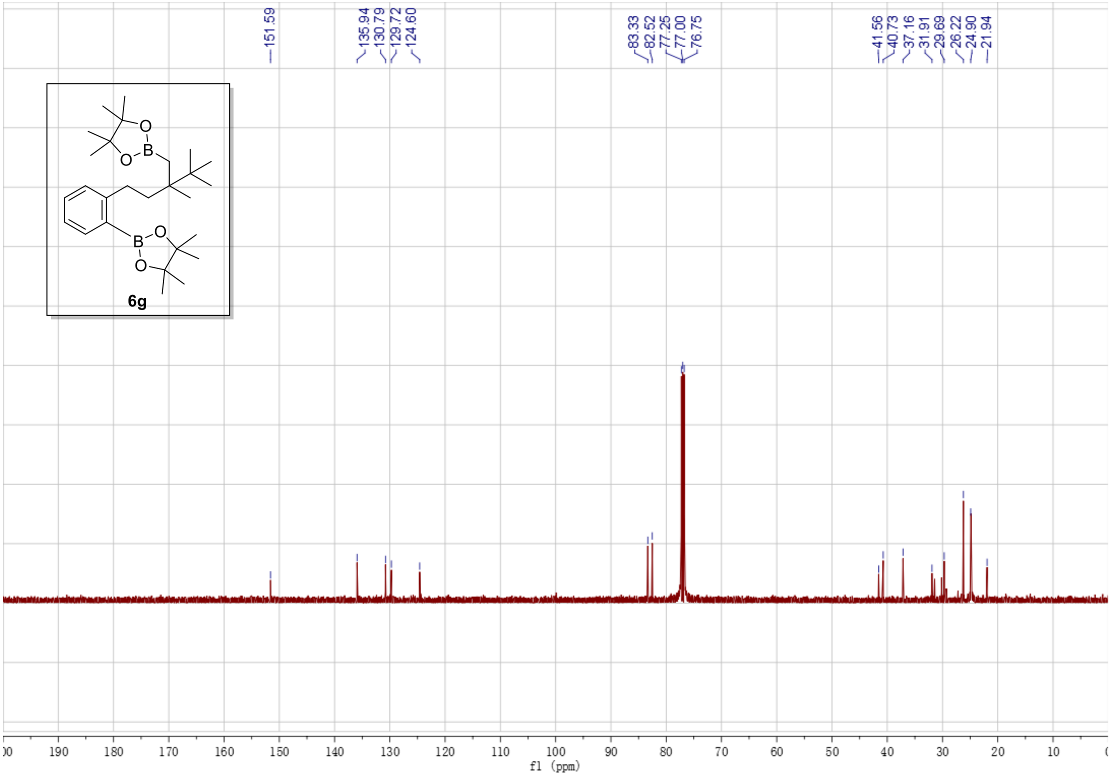

Supplementary Figure 194. ^{13}C NMR spectrum of **6g**.

^{11}B spectrum (160 MHz, room temperature, $CDCl_3$) of (**6g**)

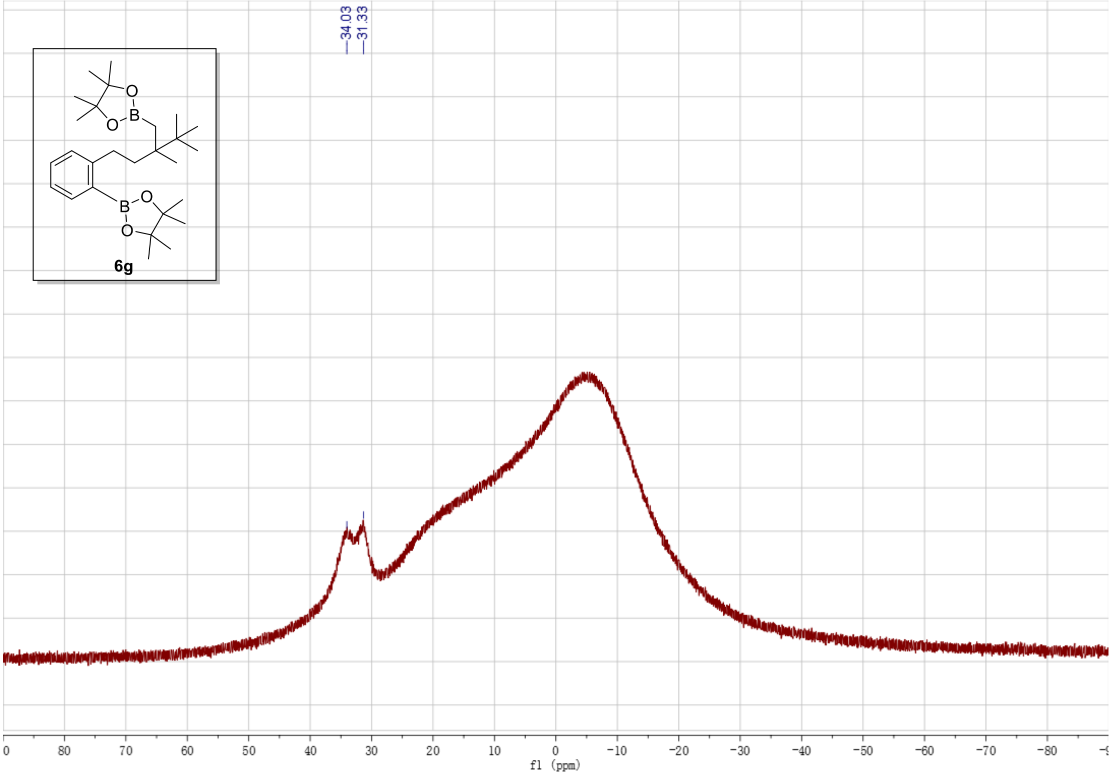

Supplementary Figure 195. ^{11}B spectrum of **6g**.

4,4,5,5-tetramethyl-2-(2-(5-phenyl-3-(4,4,5,5-tetramethyl-1,3,2-dioxaborolan-2-yl)pentyl)phenyl)-1,3,2-dioxaborolane (6'a)

^{1}H spectrum (500 MHz, room temperature, $CDCl_3$) of (**6'a**)

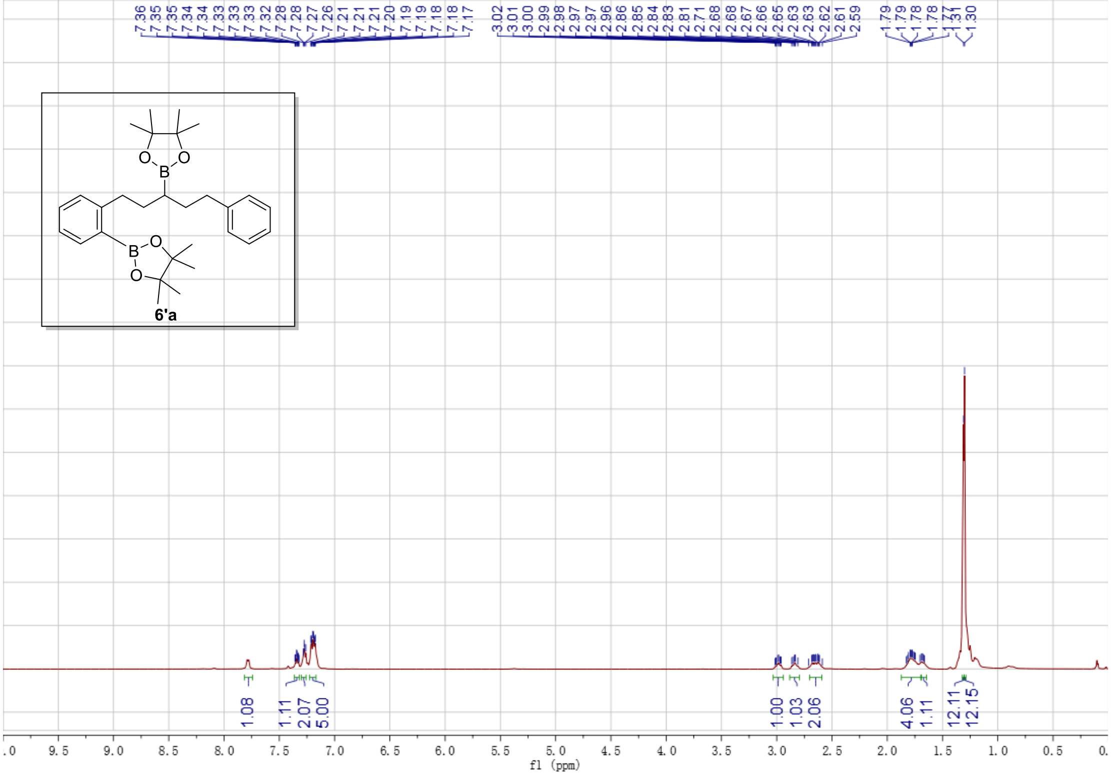

Supplementary Figure 196. ^{1}H NMR spectrum of **6'a**.

^{13}C spectrum (126 MHz, room temperature, $CDCl_3$) of (**6'a**)

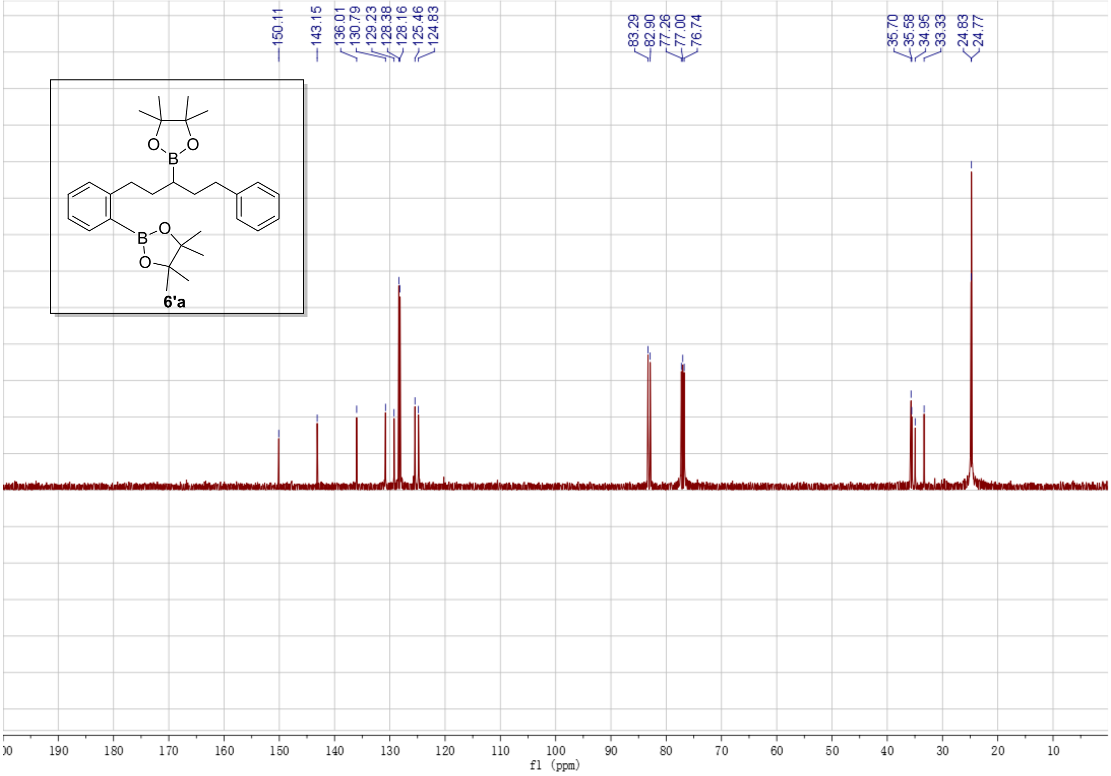

Supplementary Figure 197. ^{13}C NMR spectrum of **6'a**.

^{11}B spectrum (128 MHz, room temperature, $CDCl_3$) of (**6'a**)

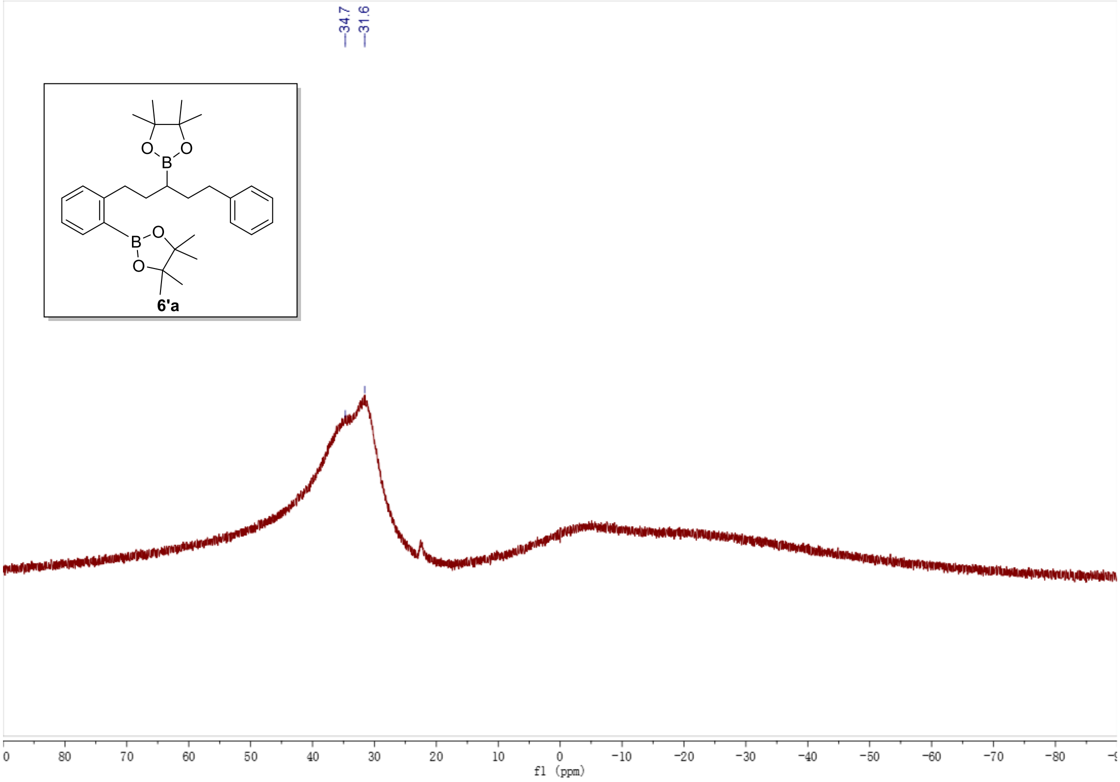

Supplementary Figure 198. ^{11}B spectrum of **6'a**.

4,4,5,5-tetramethyl-2-(2-(3-(4,4,5,5-tetramethyl-1,3,2-dioxaborolan-2-yl)-4-(m-tolyl)butyl)phenyl)-1,3,2-dioxaborolane (6'b)

^{1}H spectrum (500 MHz, room temperature, $CDCl_3$) of (**6'b**)

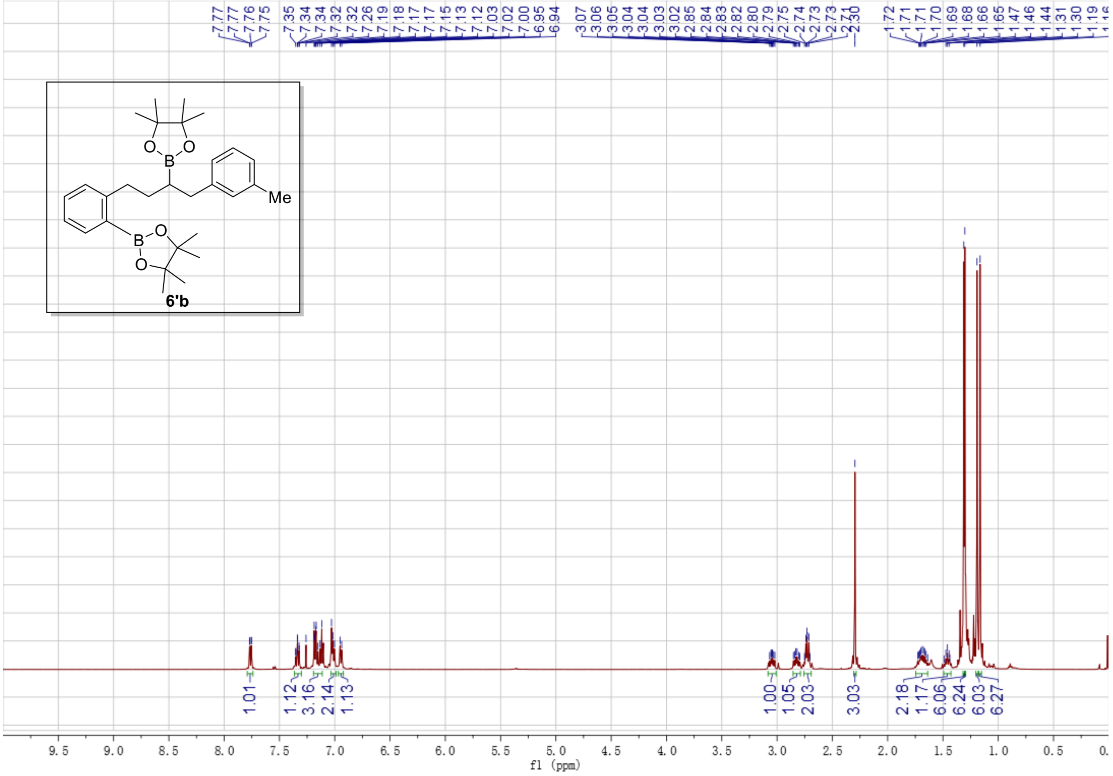

Supplementary Figure 199. ^{1}H NMR spectrum of **6'b**.

^{13}C spectrum (126 MHz, room temperature, $CDCl_3$) of (**6'b**)

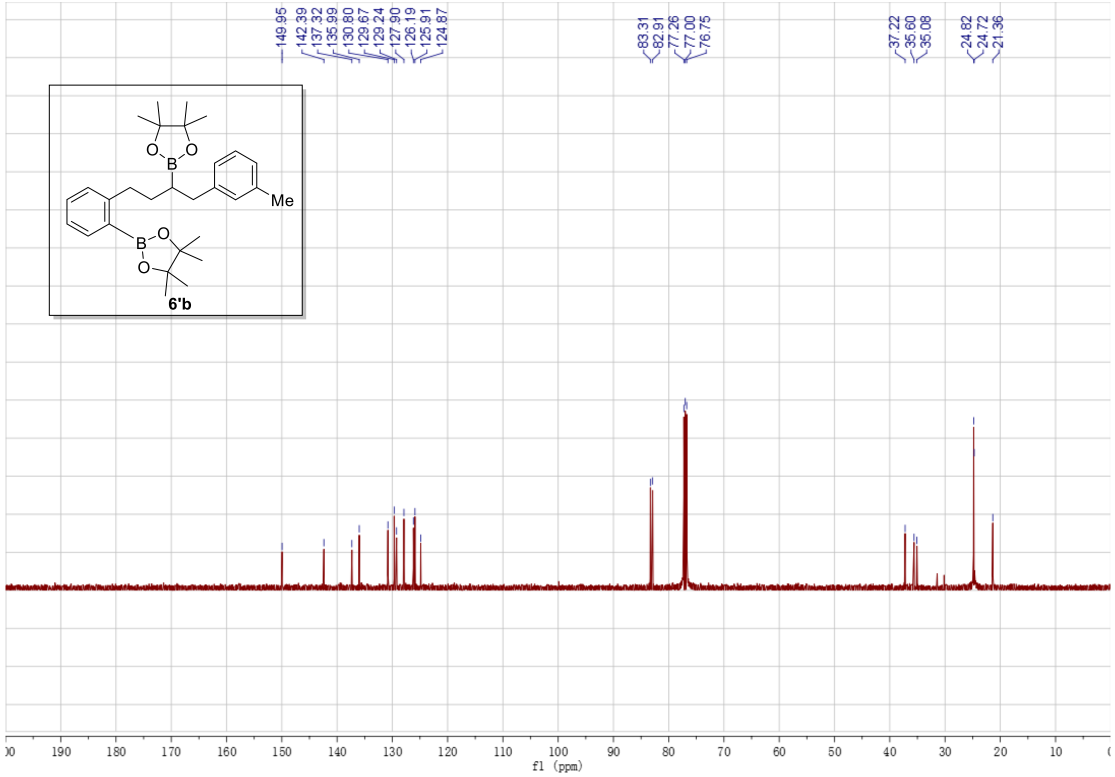

Supplementary Figure 200. ^{13}C NMR spectrum of **6'b**.

^{11}B spectrum (128 MHz, room temperature, $CDCl_3$) of (**6'b**)

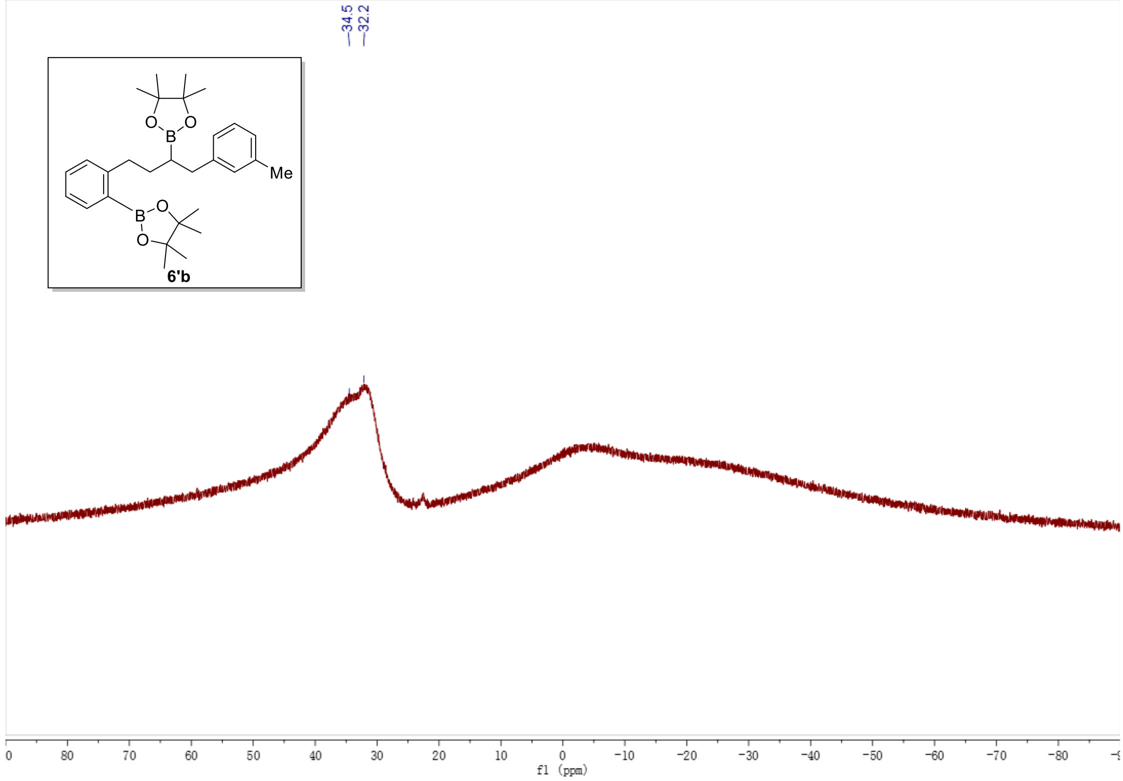

Supplementary Figure 201. ^{11}B spectrum of **6'b**.

2-(2-(4-(3-chlorophenyl)-3-(4,4,5,5-tetramethyl-1,3,2-dioxaborolan-2-yl)butyl)phenyl)-4,4,5,5-tetramethyl-1,3,2-dioxaborolane (6'c)

^{1}H spectrum (400 MHz, room temperature, $CDCl_3$) of (**6'c**)

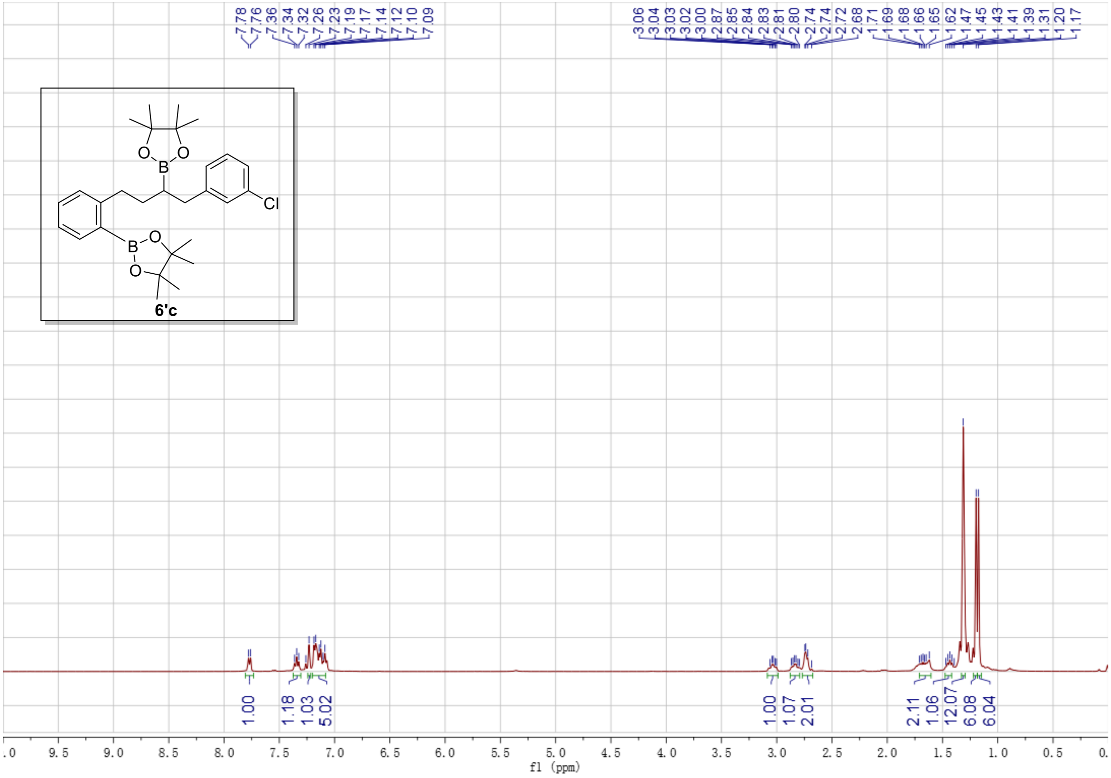

Supplementary Figure 202. ^{1}H NMR spectrum of **6'c**.

^{13}C spectrum (126 MHz, room temperature, $CDCl_3$) of (**6'c**)

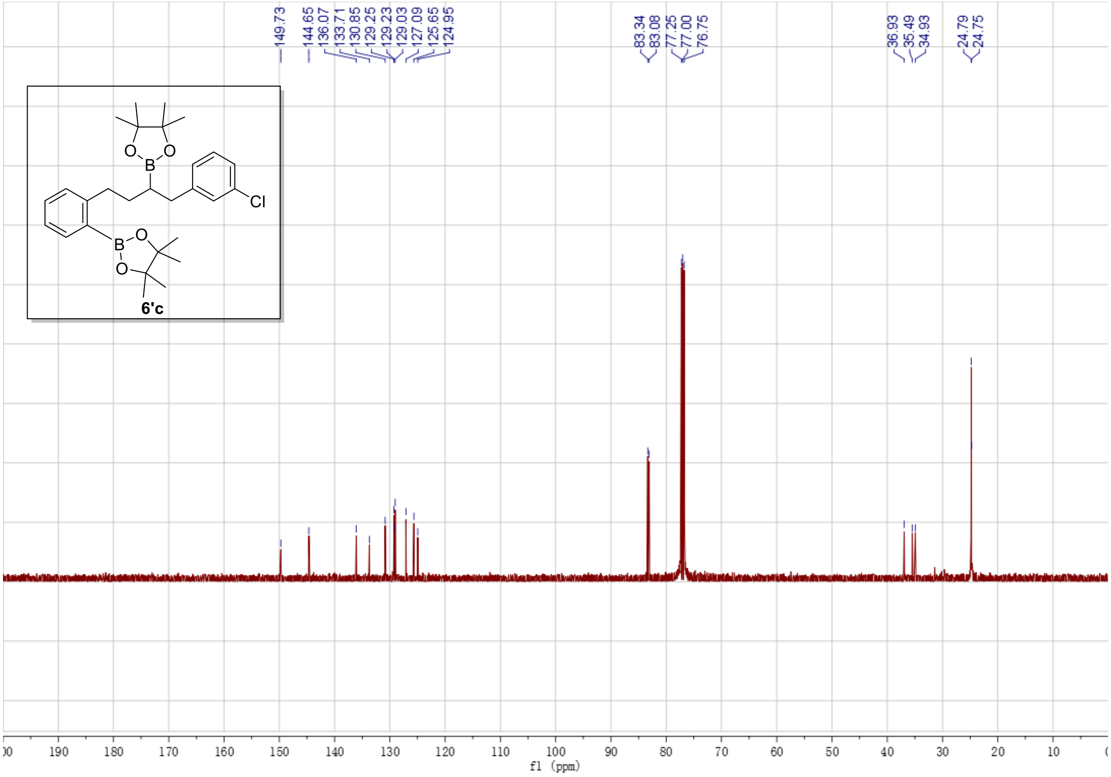

Supplementary Figure 203. ^{13}C NMR spectrum of **6'c**.

^{11}B spectrum (128 MHz, room temperature, $CDCl_3$) of (**6'c**)

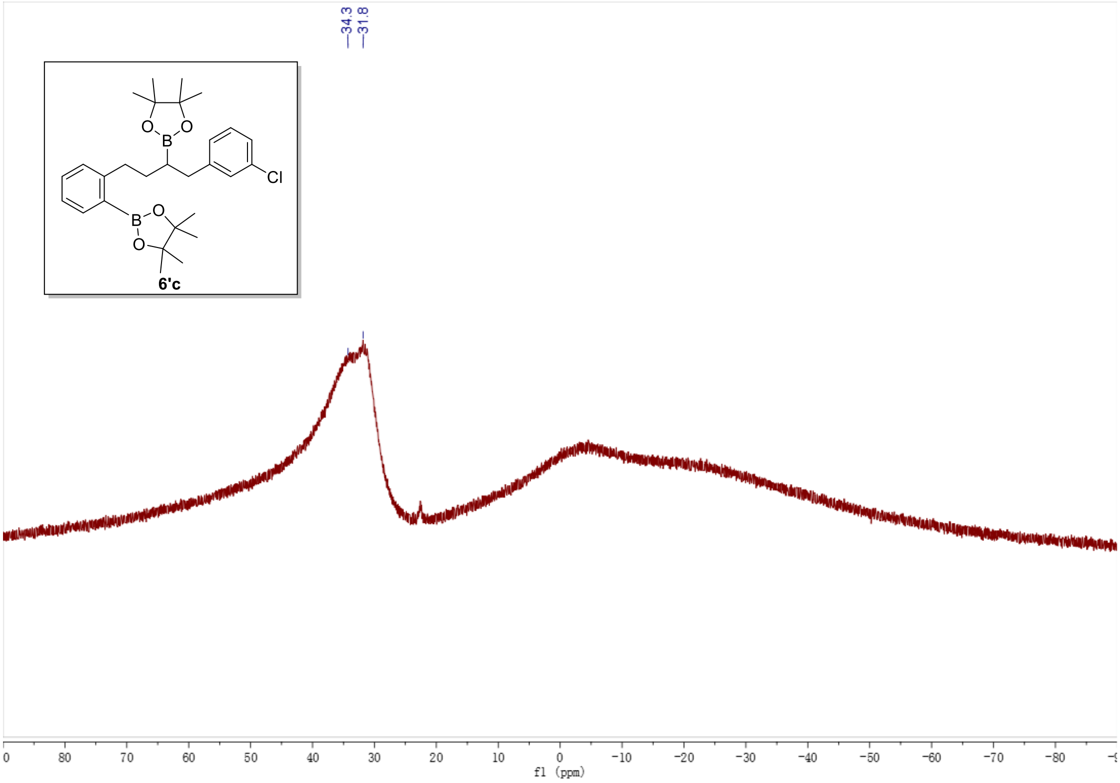

Supplementary Figure 204. ^{11}B spectrum of **6'c**.

2-(2-(4-(3-fluorophenyl)-3-(4,4,5,5-tetramethyl-1,3,2-dioxaborolan-2-yl)butyl)phenyl)-4,4,5,5-tetramethyl-1,3,2-dioxaborolane (6'd)

^{1}H spectrum (500 MHz, room temperature, $CDCl_3$) of (**6'd**)

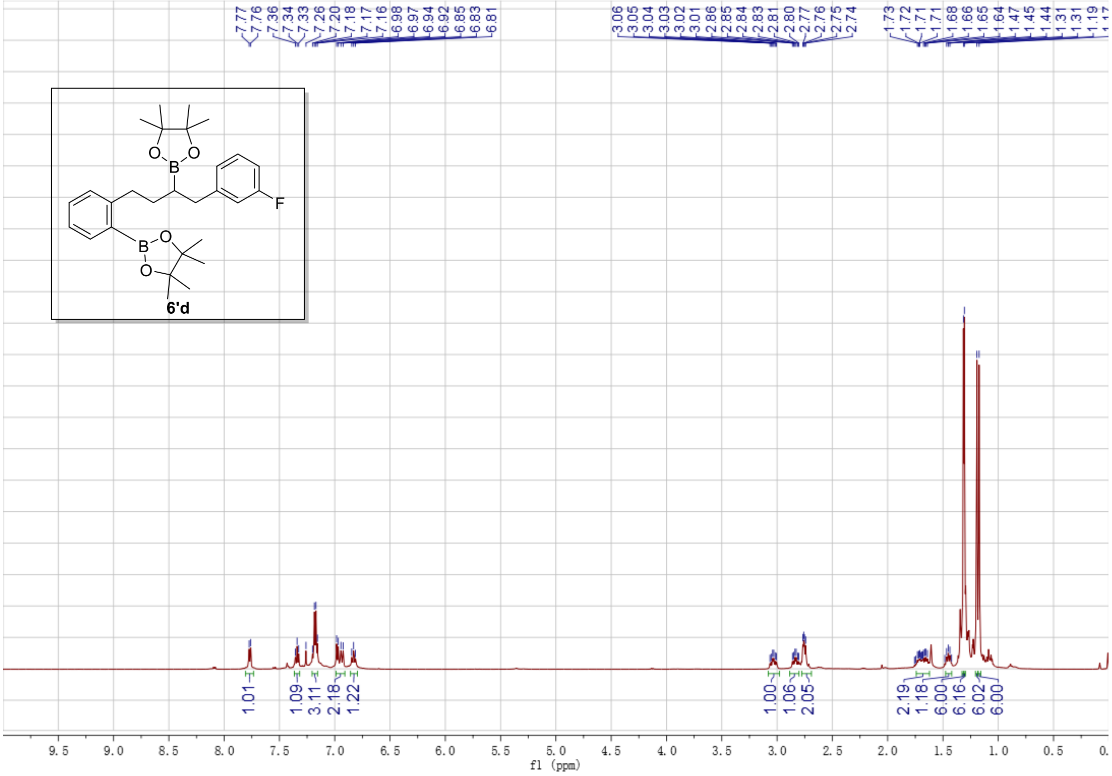

Supplementary Figure 205. ^{1}H NMR spectrum of **6'd**.

^{13}C spectrum (126 MHz, room temperature, $CDCl_3$) of (**6'd**)

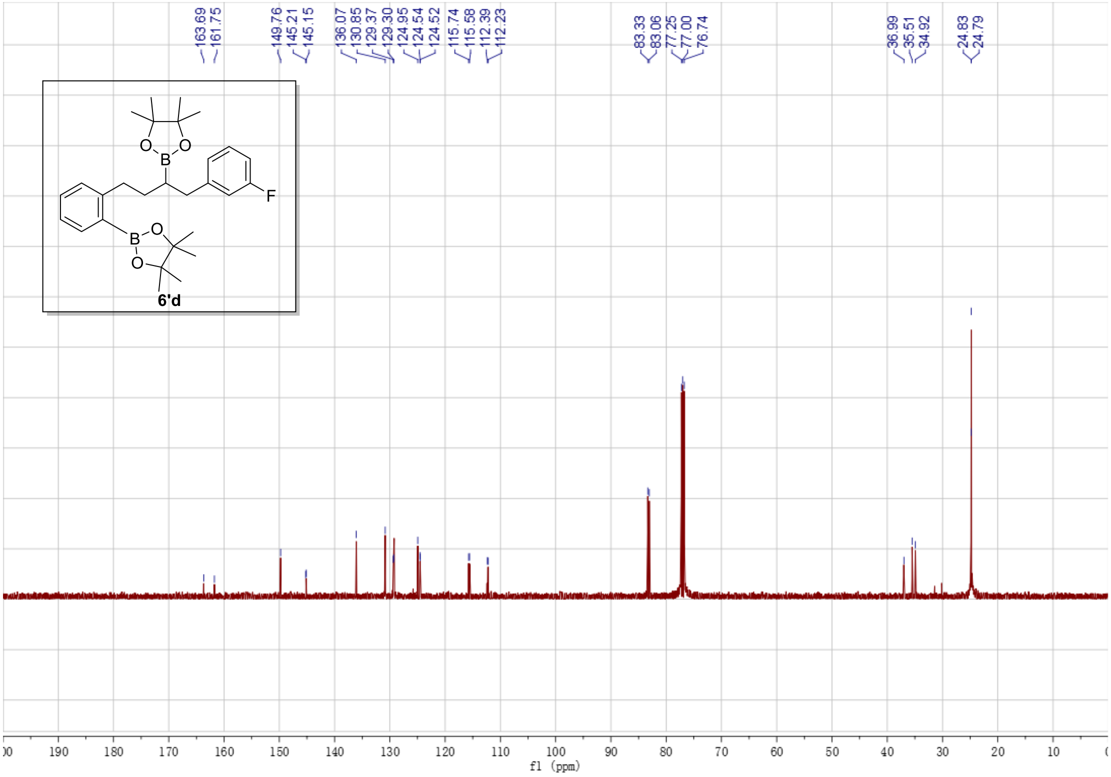

Supplementary Figure 206. ^{13}C NMR spectrum of **6'd**.

^{11}B spectrum (128 MHz, room temperature, $CDCl_3$) of (**6'd**)

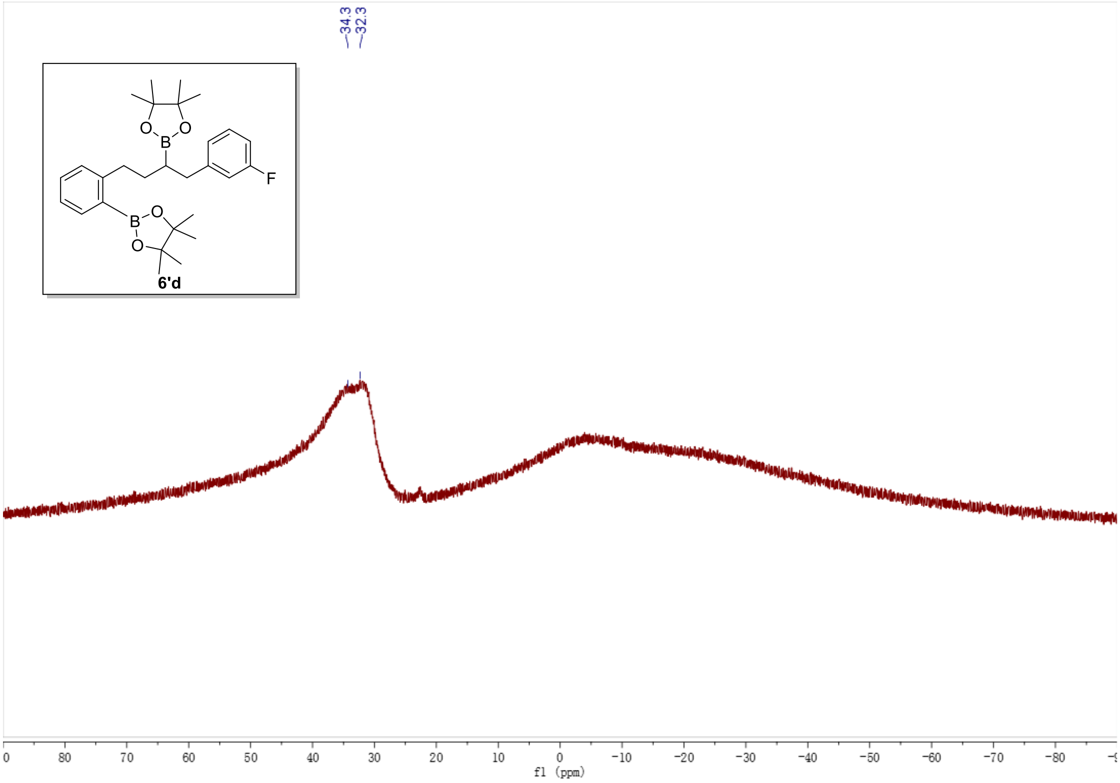

Supplementary Figure 207. ^{11}B spectrum of **6'd**.

^{19}F spectrum (471 MHz, room temperature, $CDCl_3$) of (**6'd**)

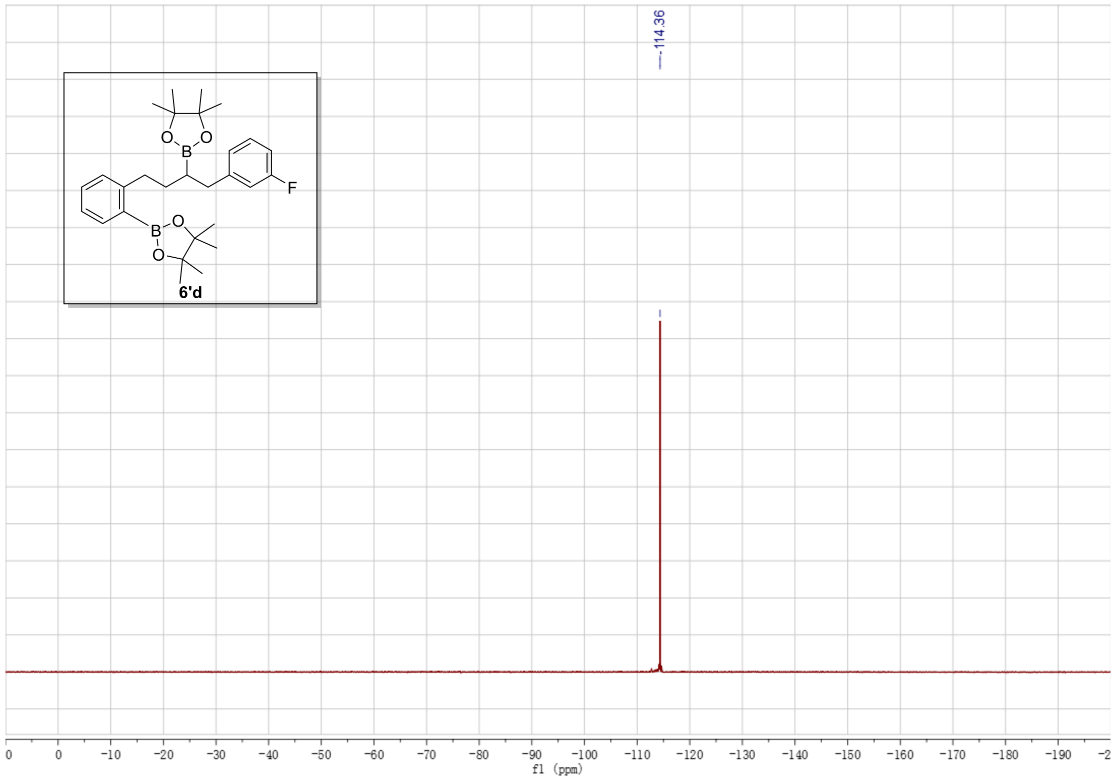

Supplementary Figure 208. ^{19}F spectrum of **6'd**.

2-(2-(4-(4-(tert-butyl)phenyl)-3-(4,4,5,5-tetramethyl-1,3,2-dioxaborolan-2-yl)butyl)phenyl)-4,4,5,5-tetramethyl-1,3,2-dioxaborolane (6'e)

^{1}H spectrum (400 MHz, room temperature, $CDCl_3$) of (**6'e**)

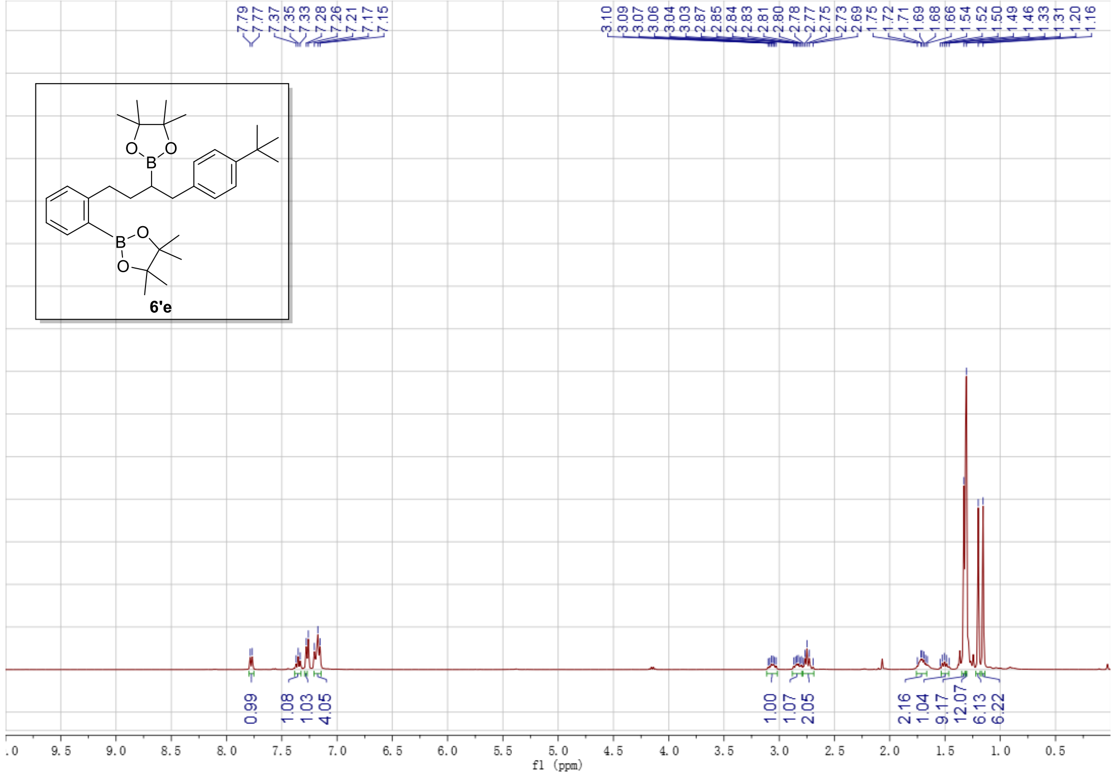

Supplementary Figure 209. ^{1}H NMR spectrum of **6'e**.

^{13}C spectrum (126 MHz, room temperature, CDCl$_3$) of (**6'e**)

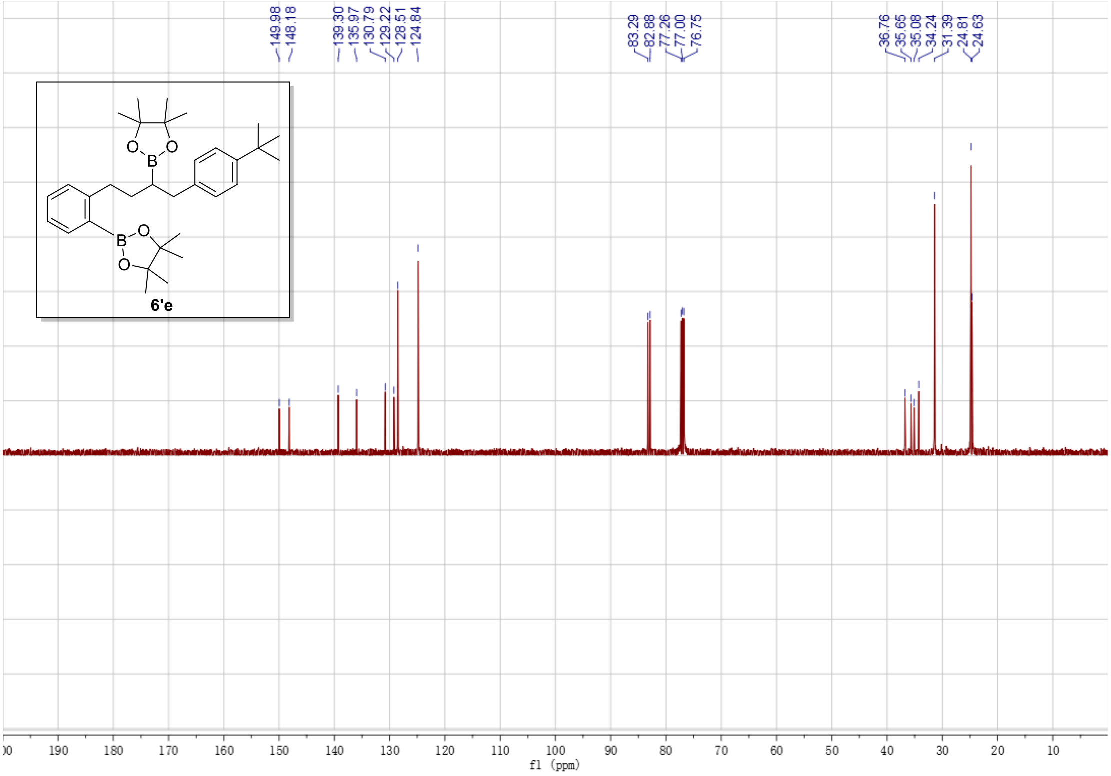

Supplementary Figure 210. ^{13}C NMR spectrum of **6'e**.

^{11}B spectrum (128 MHz, room temperature, CDCl$_3$) of (**6'e**)

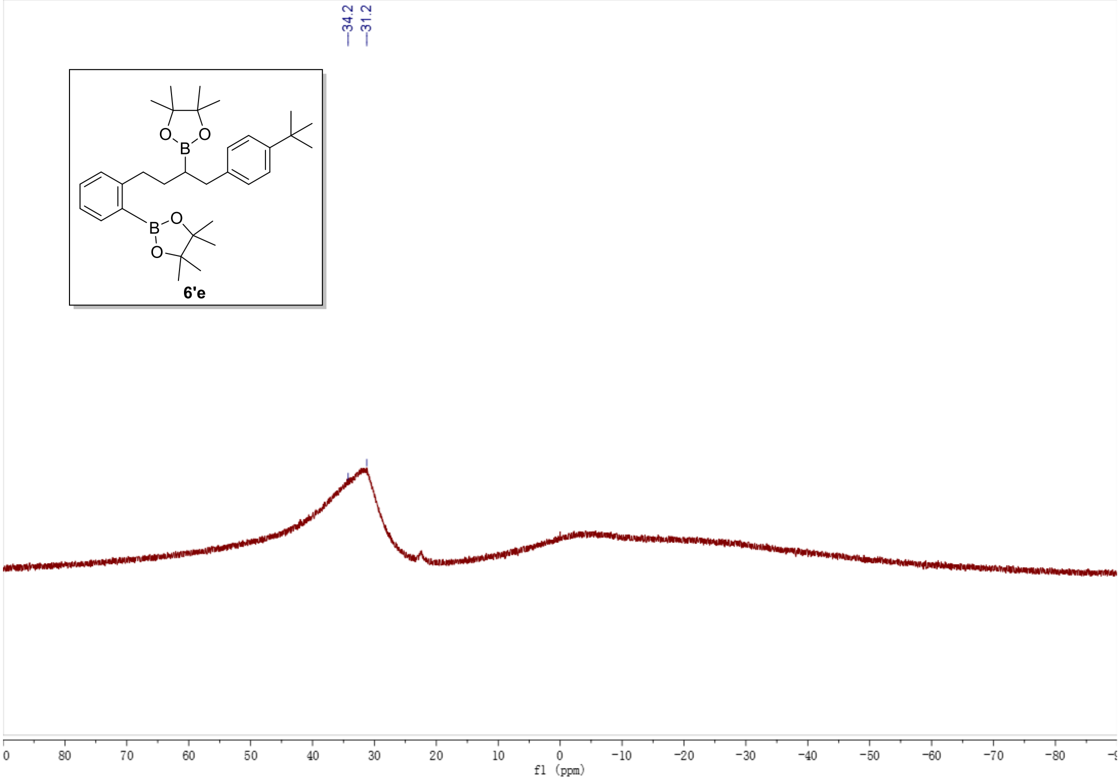

Supplementary Figure 211. ^{11}B spectrum of **6'e**.

4,4,5,5-tetramethyl-2-(3-methyl-1-phenyl-5-(2-(4,4,5,5-tetramethyl-1,3,2-dioxaborolan-2-yl)phenyl)pentan-3-yl)-1,3,2-dioxaborolane (6'f)

^{1}H spectrum (500 MHz, room temperature, $CDCl_3$) of (**6'f**)

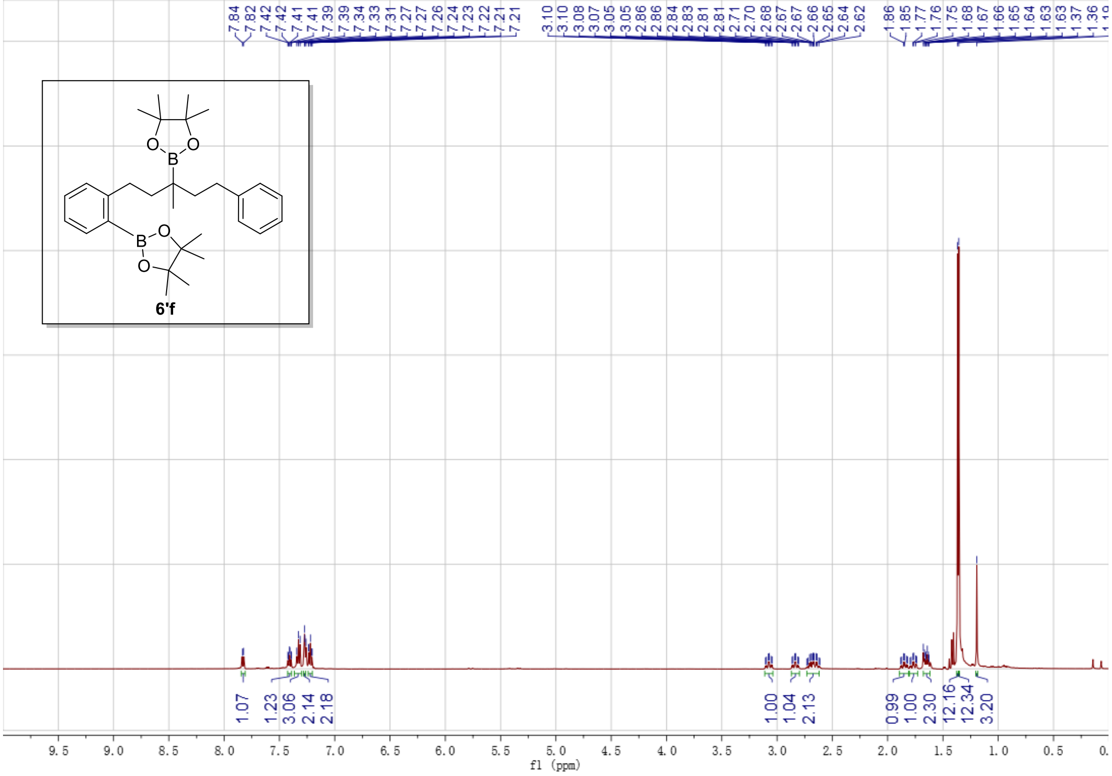

Supplementary Figure 212. ^{1}H NMR spectrum of **6'f**.

^{13}C spectrum (126 MHz, room temperature, $CDCl_3$) of (**6'f**)

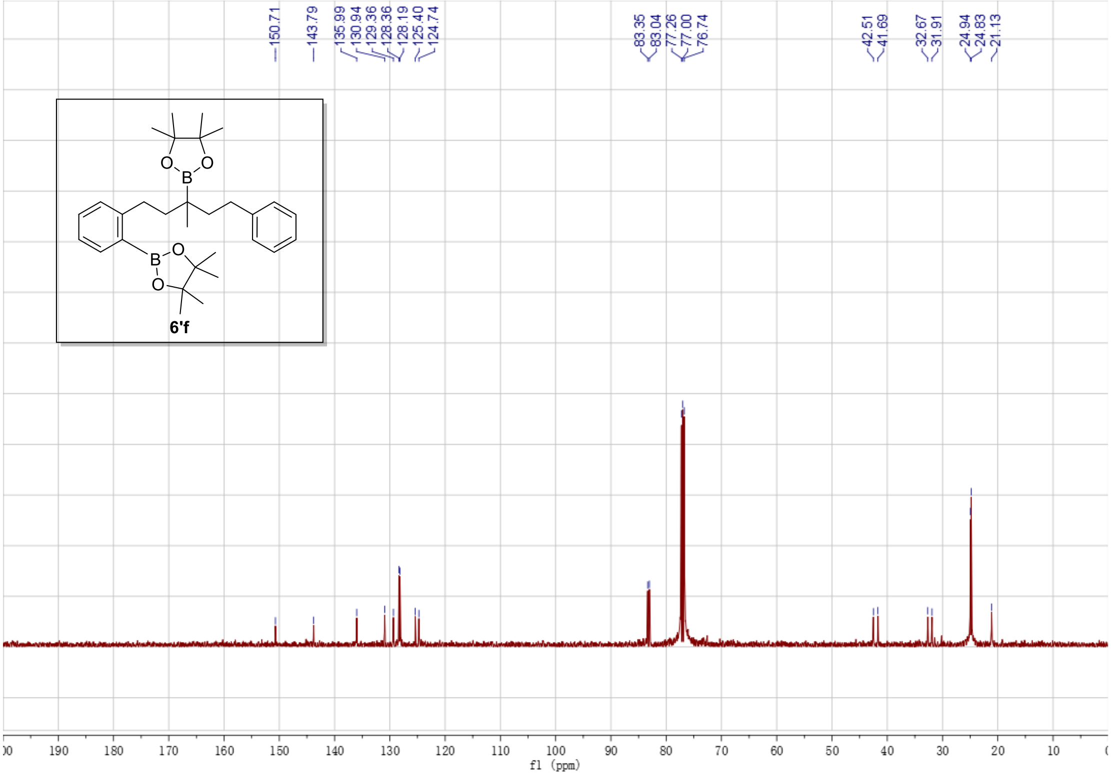

Supplementary Figure 213. ^{13}C NMR spectrum of **6'f**.

^{11}B spectrum (128 MHz, room temperature, $CDCl_3$) of (**6'f**)

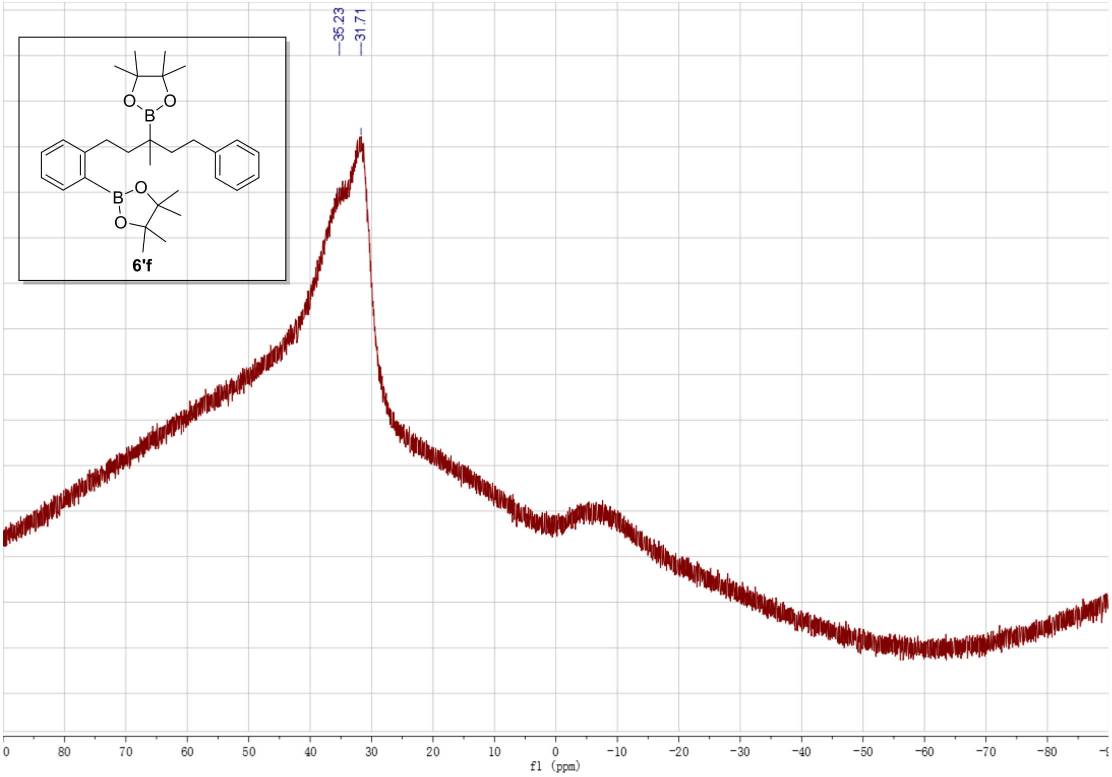

Supplementary Figure 214. ^{11}B spectrum of **6'f**.

2-(2-(5-(4-methoxyphenyl)-3-methyl-3-(4,4,5,5-tetramethyl-1,3,2-dioxaborolan-2-yl)pentyl)phenyl)-4,4,5,5-tetramethyl-1,3,2-dioxaborolane (6'g)

^{1}H spectrum (500 MHz, room temperature, $CDCl_3$) of (**6'g**)

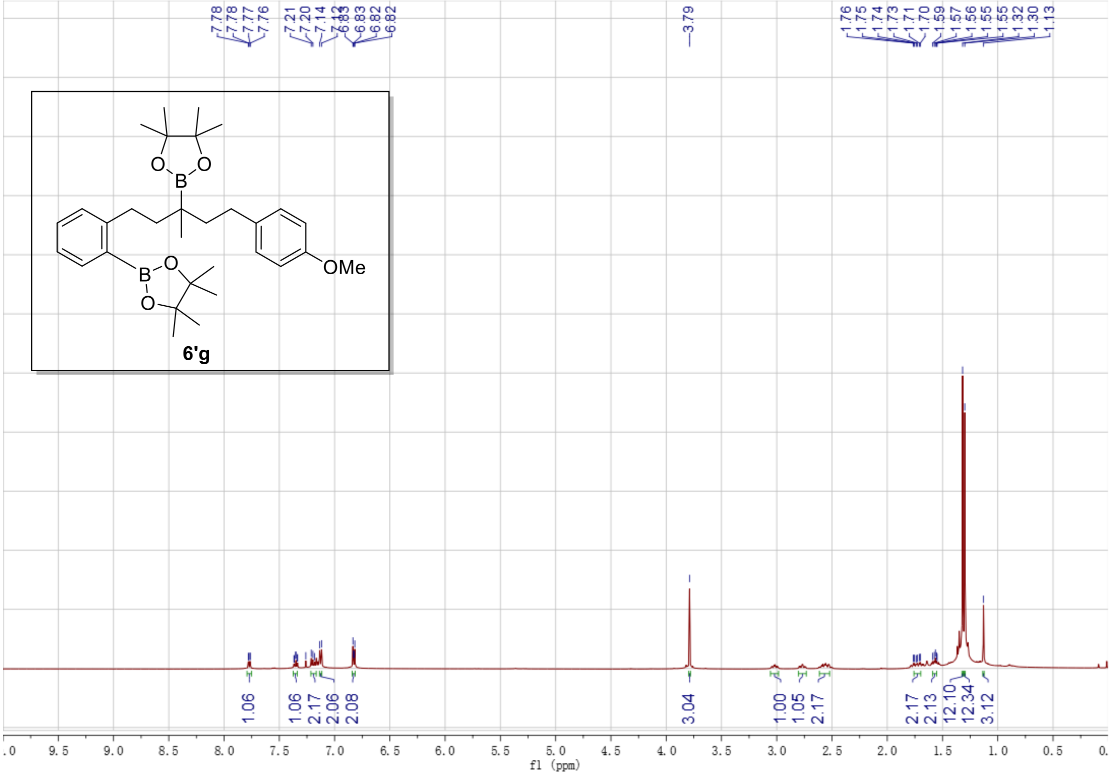

Supplementary Figure 215. ^{1}H NMR spectrum of **6'g**.

^{13}C spectrum (126 MHz, room temperature, $CDCl_3$) of (**6'g**)

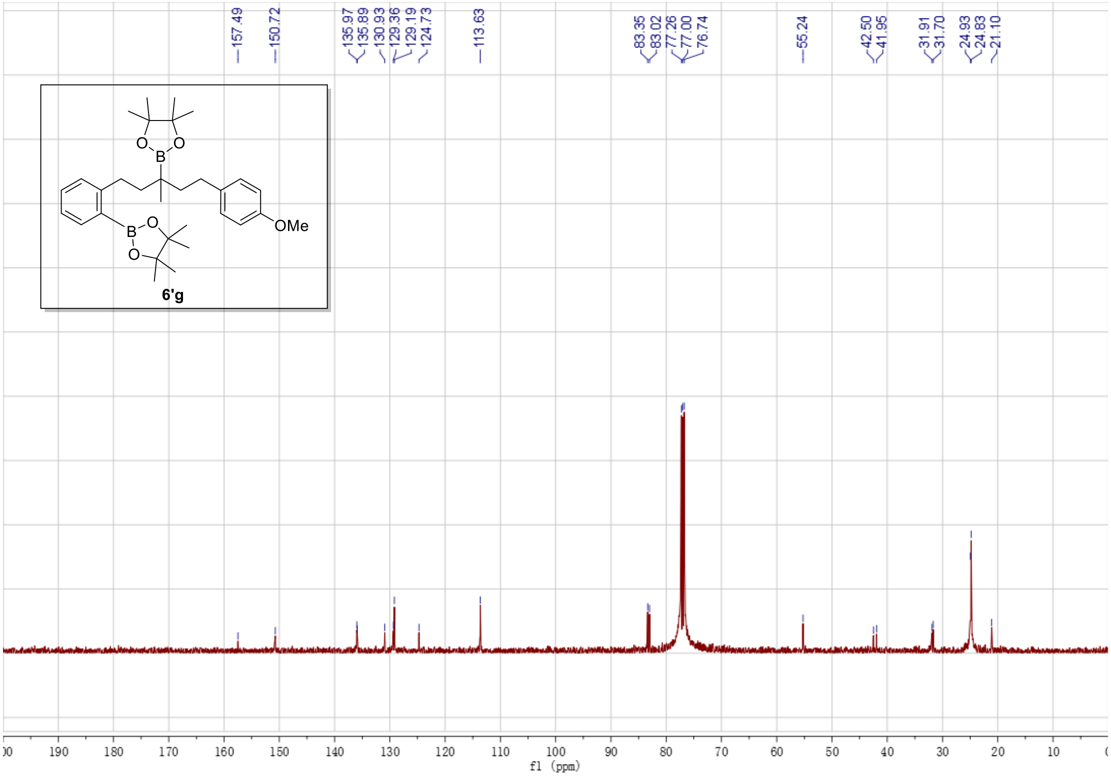

Supplementary Figure 216. ^{13}C NMR spectrum of **6'g**.

^{11}B spectrum (128 MHz, room temperature, $CDCl_3$) of (**6'g**)

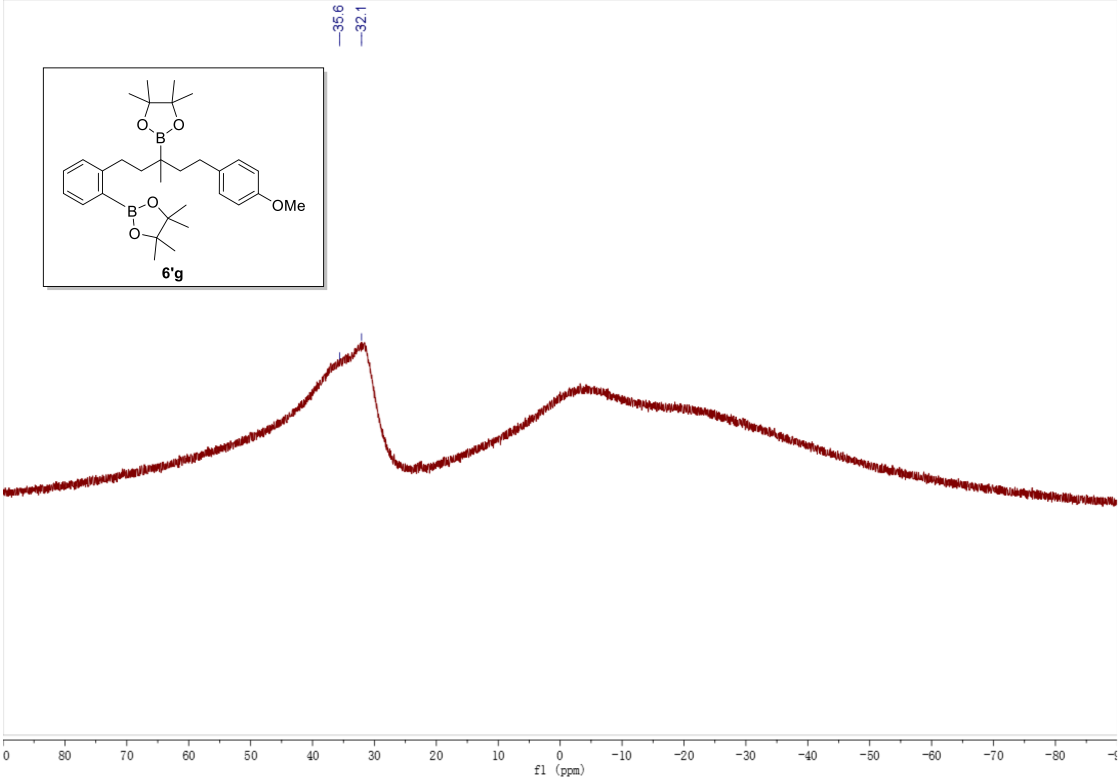

Supplementary Figure 217. ^{11}B spectrum of **6'g**.

4,4,5,5-tetramethyl-2-(2-(2-(1-(4,4,5,5-tetramethyl-1,3,2-dioxaborolan-2-yl)cyclopentyl)ethyl)phenyl)-1,3,2-dioxaborolane (6'h)

^{1}H spectrum (500 MHz, room temperature, $CDCl_3$) of (**6'h**)

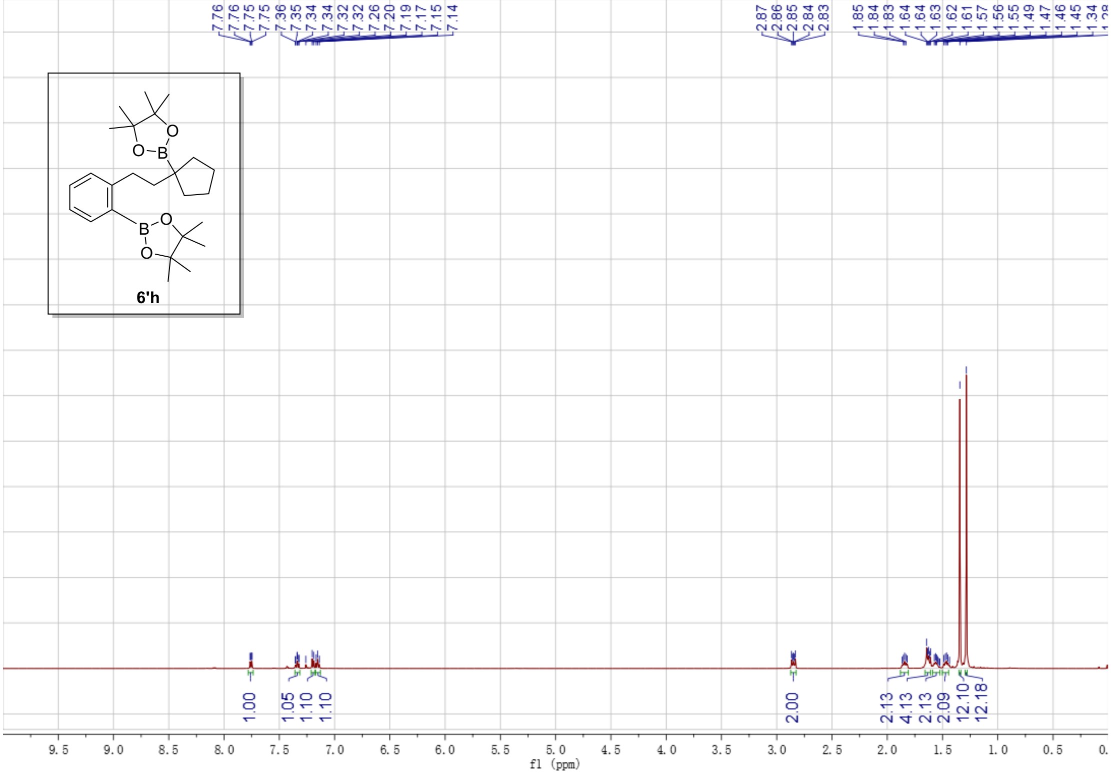

Supplementary Figure 218. ^{1}H NMR spectrum of **6'h**.

^{13}C spectrum (126 MHz, room temperature, $CDCl_3$) of (**6'h**)

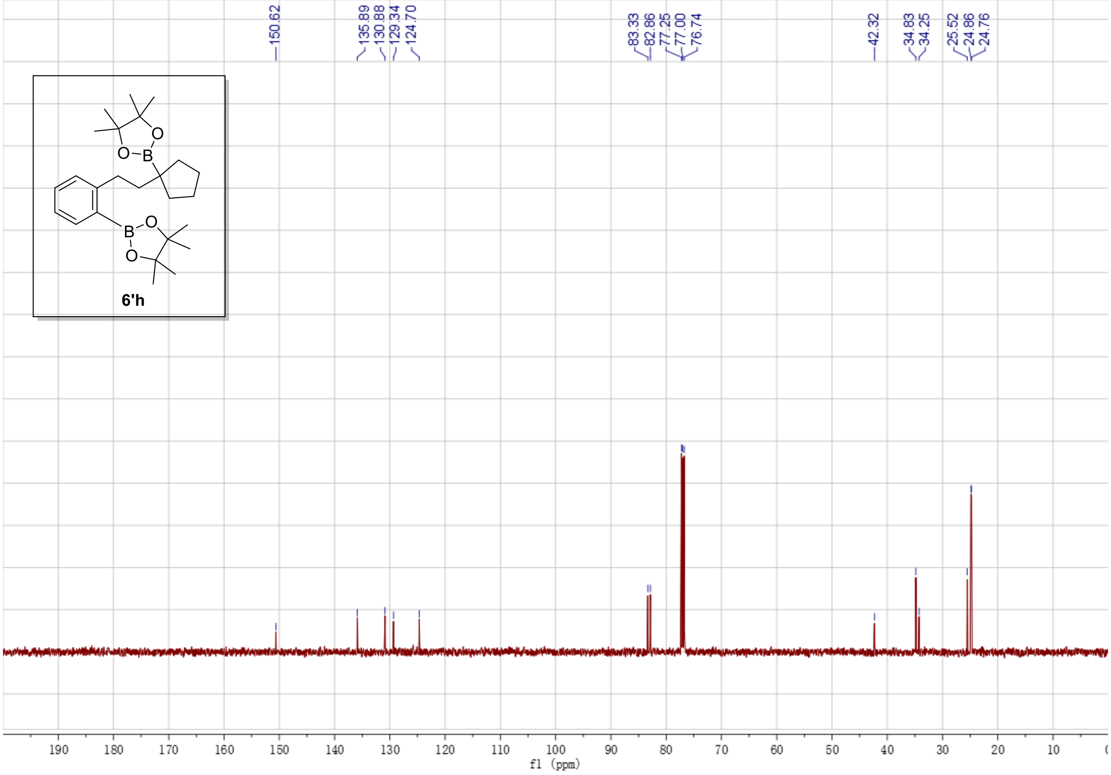

Supplementary Figure 219. ^{13}C NMR spectrum of **6'h**.

^{11}B spectrum (128 MHz, room temperature, $CDCl_3$) of (**6'h**)

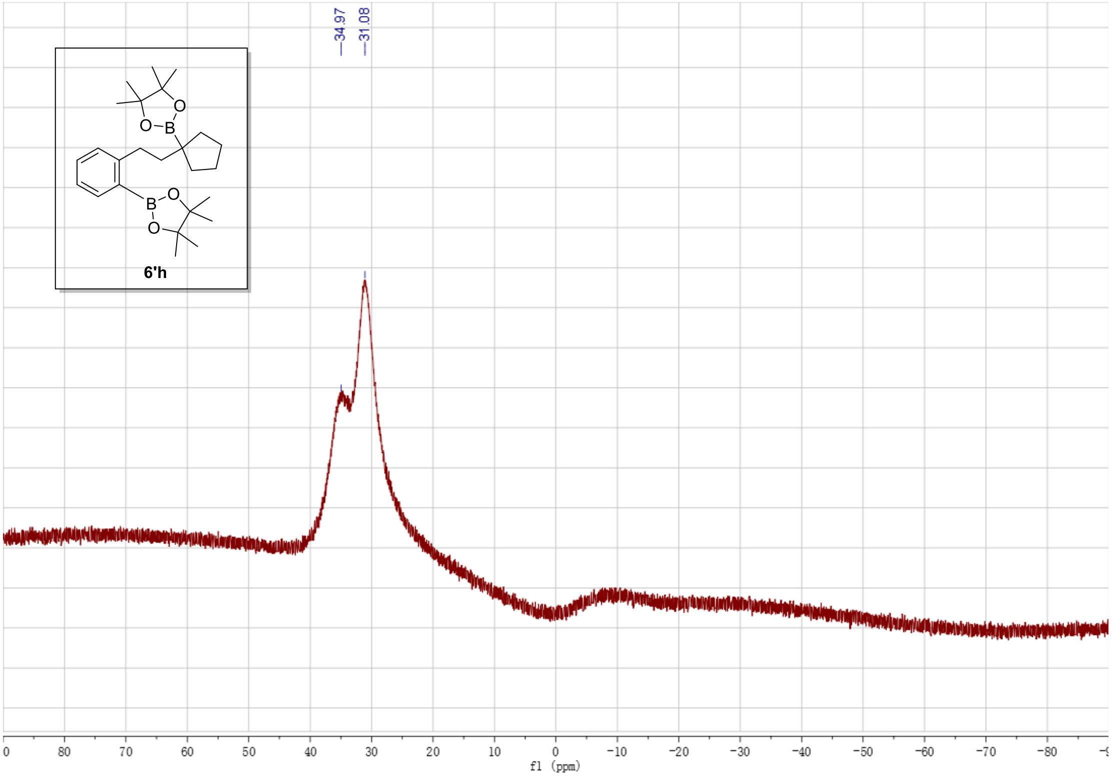

Supplementary Figure 220. ^{11}B spectrum of **6'h**.

4,4,5,5-tetramethyl-2-(2-(2-(1-(4,4,5,5-tetramethyl-1,3,2-dioxaborolan-2-yl)cyclohexyl)ethyl)phenyl)-1,3,2-dioxaborolane (6'i)

^{1}H spectrum (500 MHz, room temperature, $CDCl_3$) of (**6'i**)

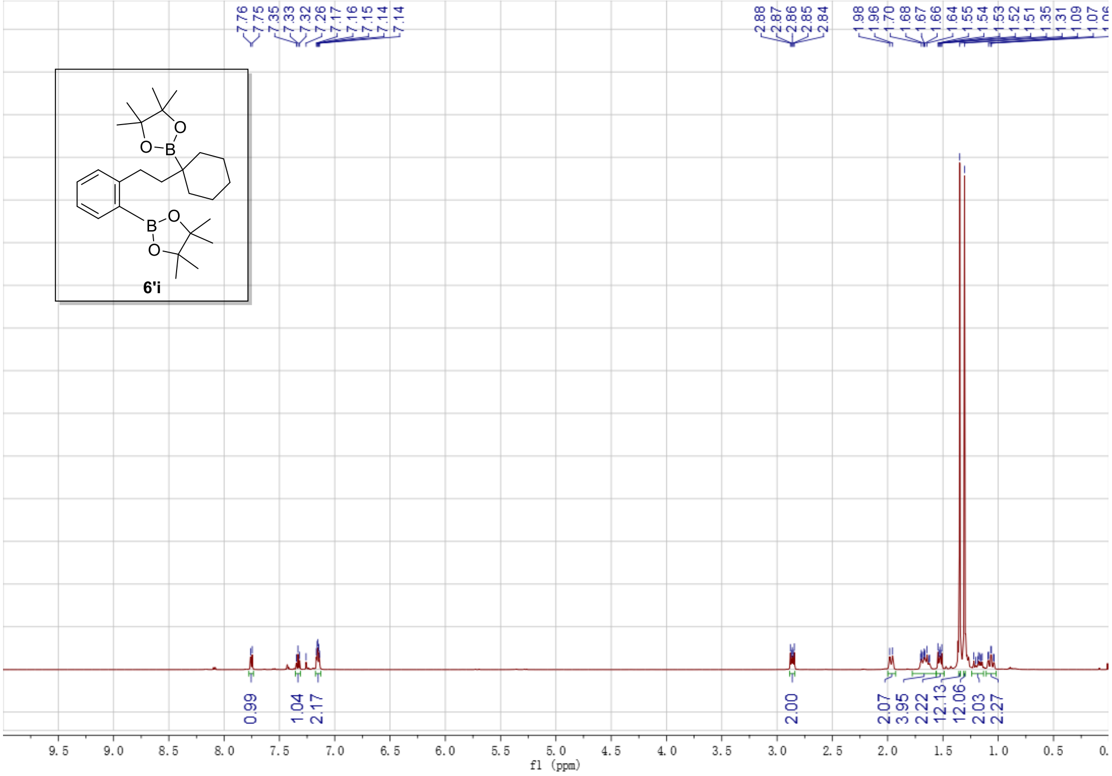

Supplementary Figure 221. ^{1}H NMR spectrum of **6'i**.

^{13}C spectrum (126 MHz, room temperature, $CDCl_3$) of (**6'i**)

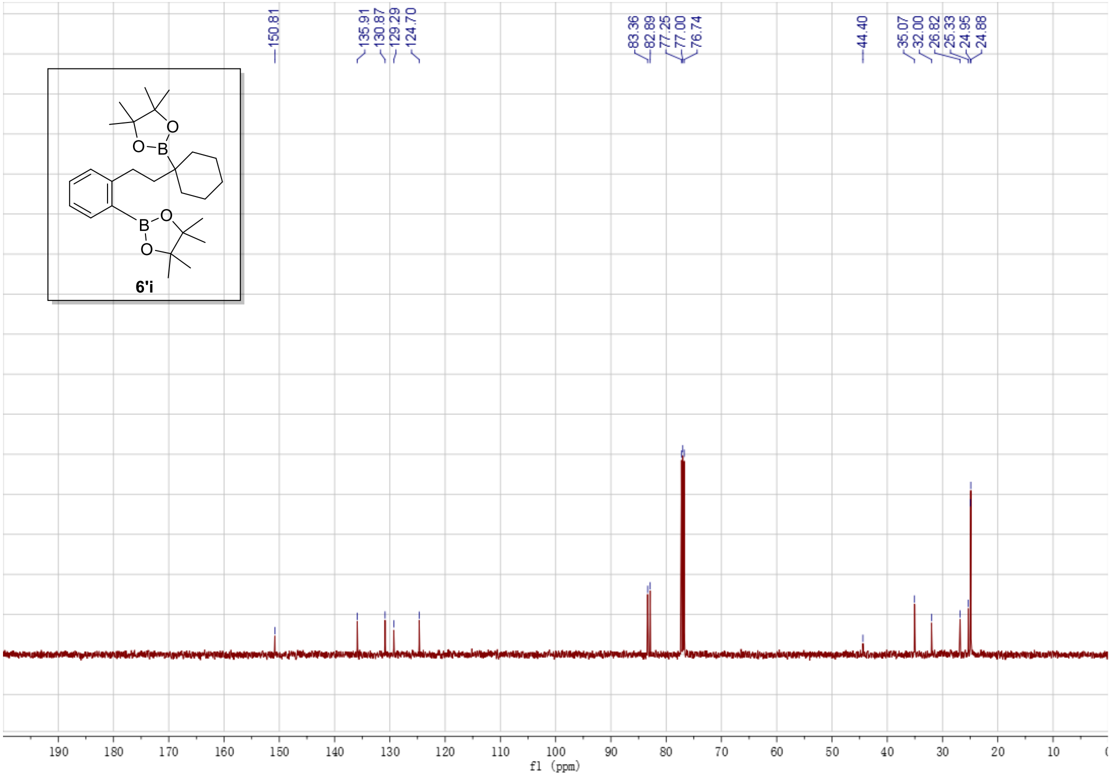

Supplementary Figure 222. ^{13}C NMR spectrum of **6'i**.

^{11}B spectrum (128 MHz, room temperature, $CDCl_3$) of (**6'i**)

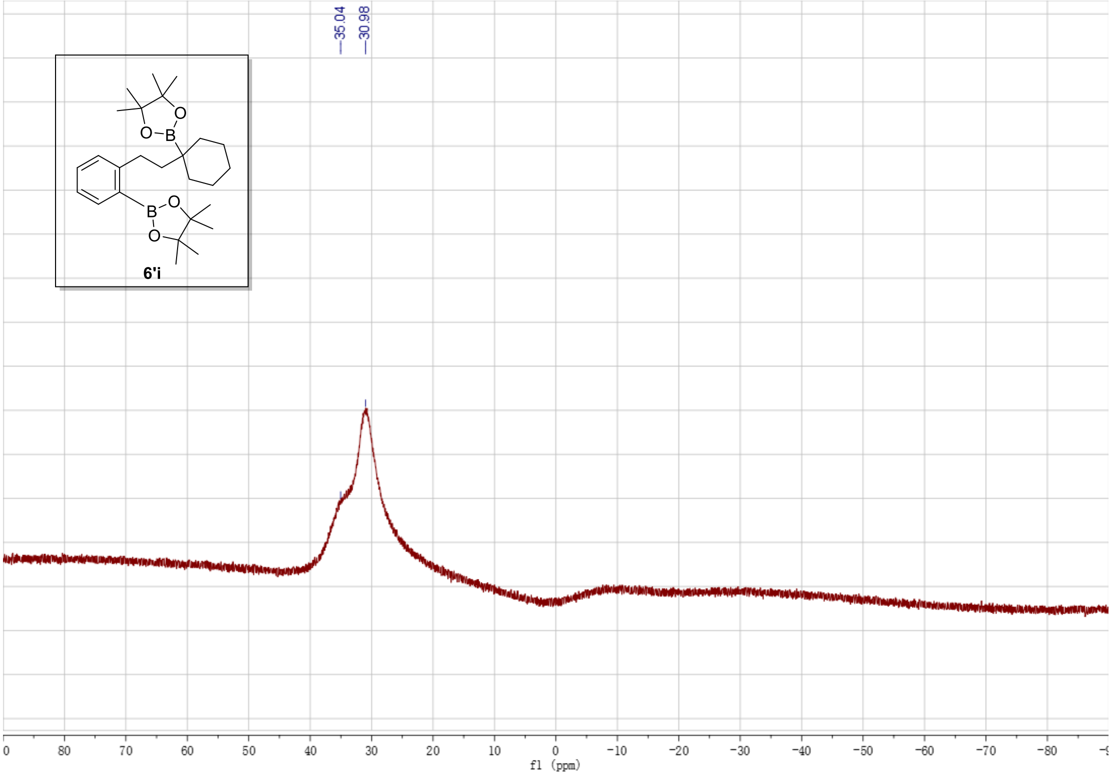

Supplementary Figure 223. ^{11}B spectrum of **6'i**.

2-(2-(4,4-dimethyl-1-phenylpentan-2-yl)phenyl)-4,4,5,5-tetramethyl-1,3,2-dioxaborolane (8a)

^{1}H spectrum (500 MHz, room temperature, $CDCl_3$) of (**8a**)

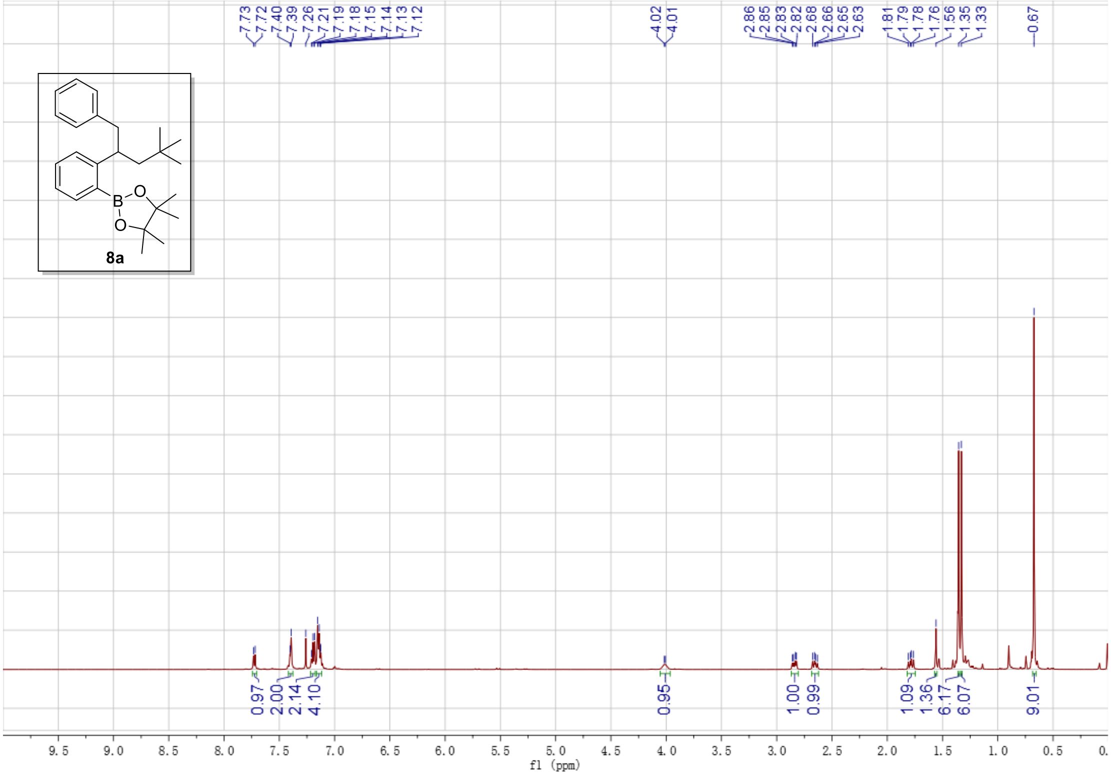

Supplementary Figure 224. ^{1}H NMR spectrum of **8a**.

^{13}C spectrum (126 MHz, room temperature, $CDCl_3$) of (**8a**)

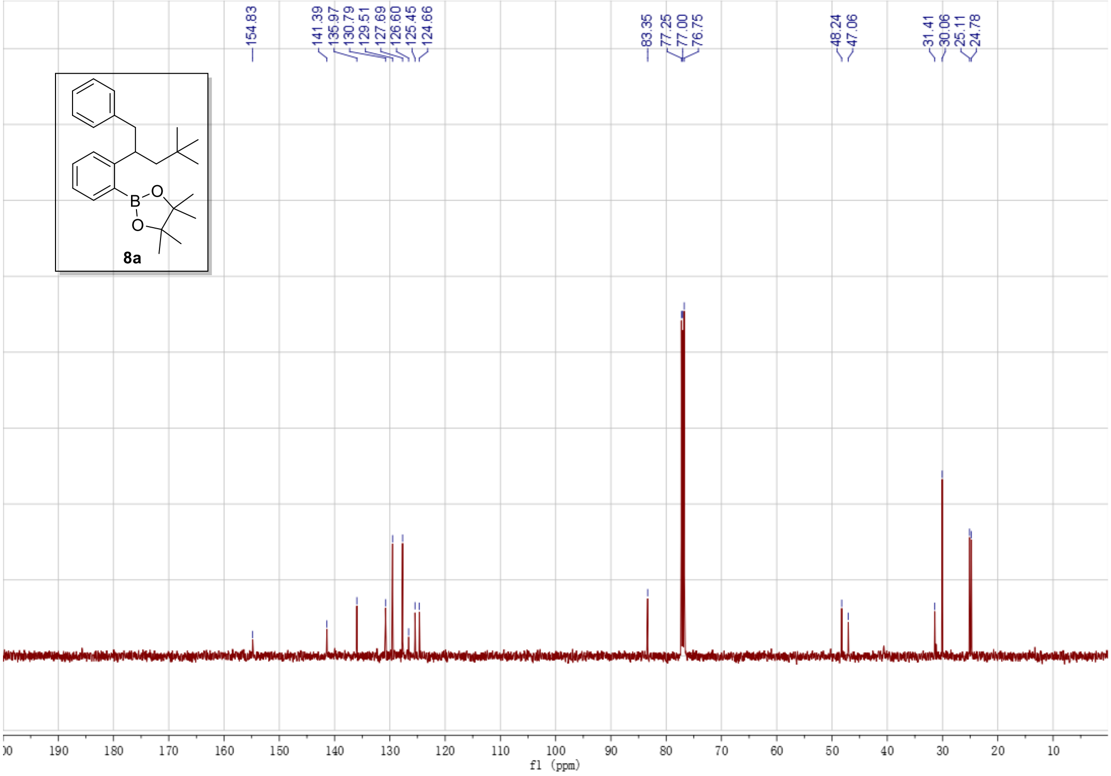

Supplementary Figure 225. ^{13}C NMR spectrum of **8a**.

^{11}B spectrum (128 MHz, room temperature, $CDCl_3$) of (**8a**)

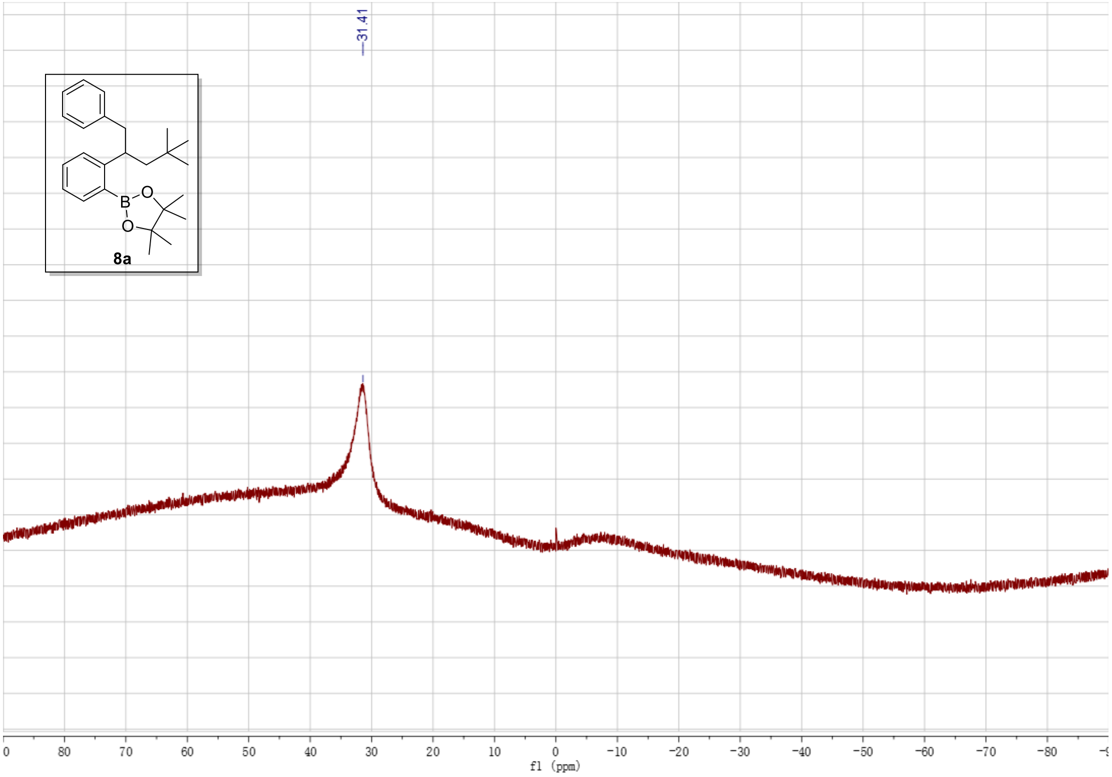

Supplementary Figure 226. ^{11}B spectrum of **8a**.

2-(2-(1-(4-fluorophenyl)-4,4-dimethylpentan-2-yl)phenyl)-4,4,5,5-tetramethyl-1,3,2-dioxaborolane (8b)

^{1}H spectrum (500MHz, room temperature, $CDCl_3$) of (**8b**)

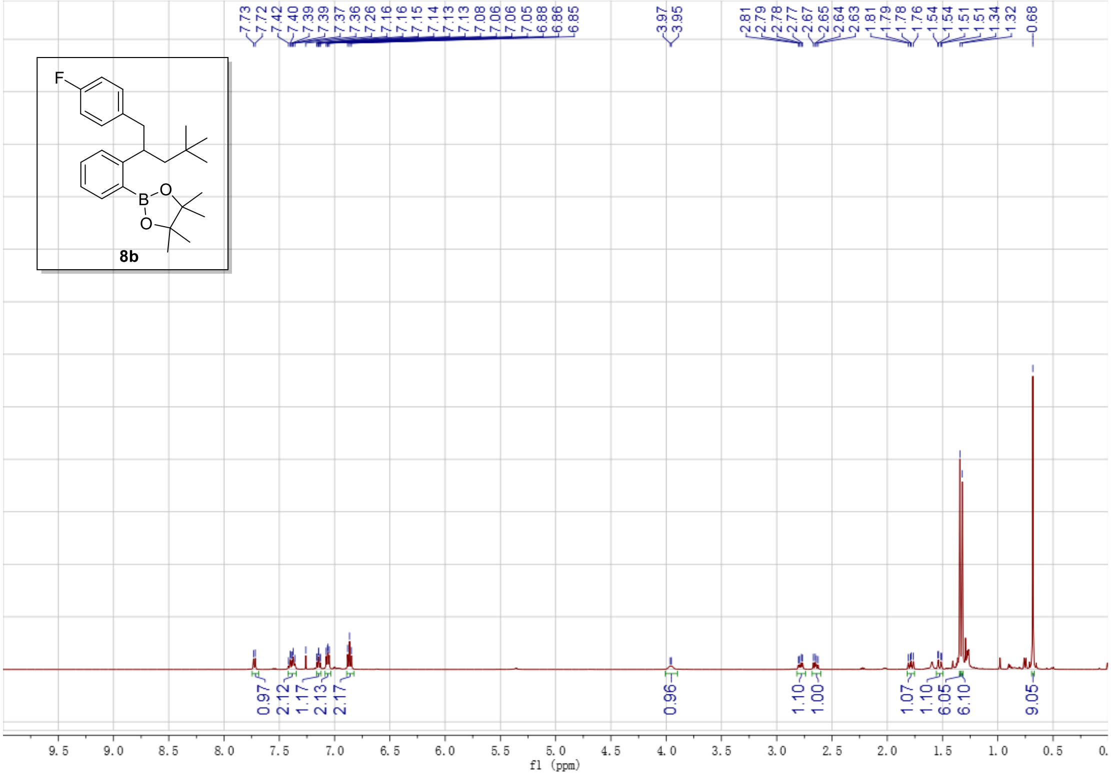

Supplementary Figure 227. ^{1}H NMR spectrum of **8b**.

^{13}C spectrum (126 MHz, room temperature, $CDCl_3$) of (**8b**)

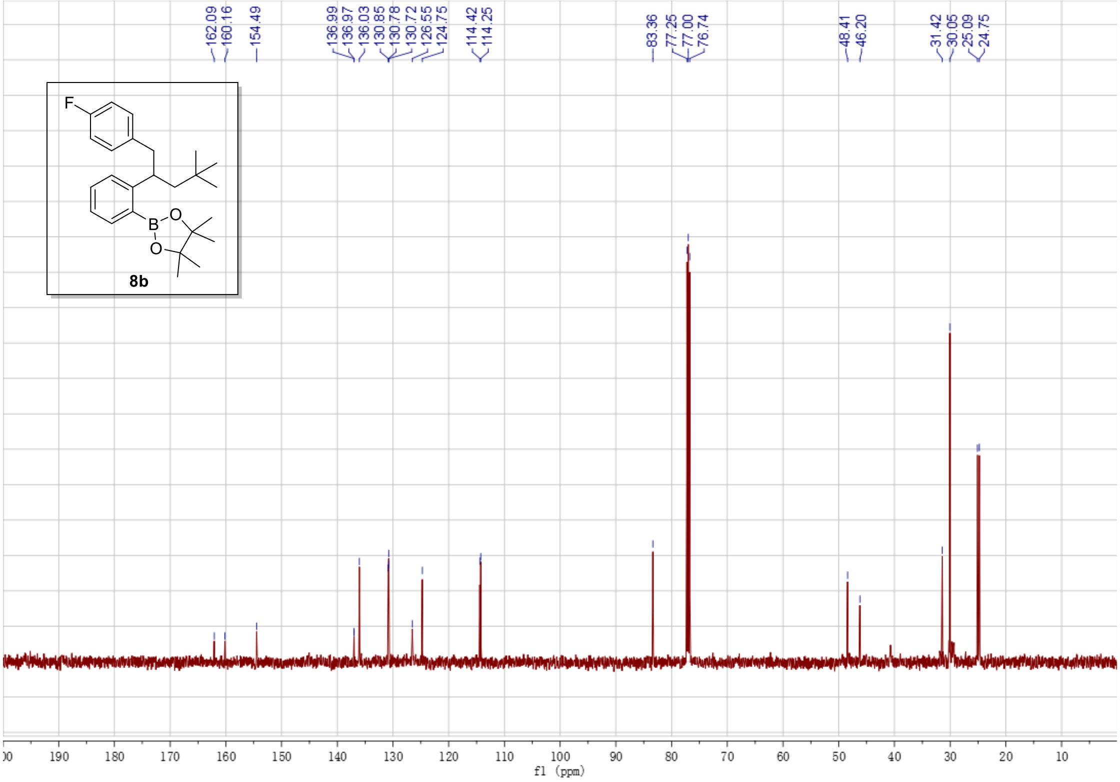

Supplementary Figure 228. ^{13}C NMR spectrum of **8b**.

^{11}B spectrum (160 MHz, room temperature, $CDCl_3$) of (**8b**)

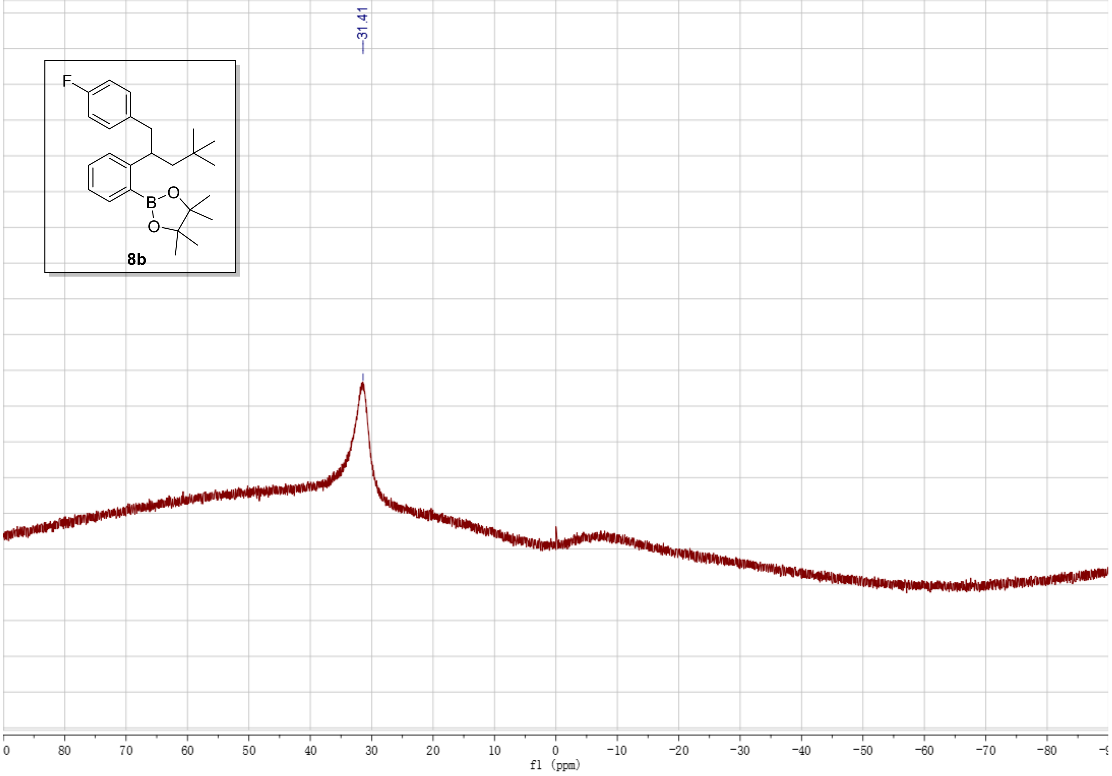

Supplementary Figure 229. ^{11}B spectrum of **8b**.

^{19}F spectrum (471 MHz, room temperature, $CDCl_3$) of (**8b**)

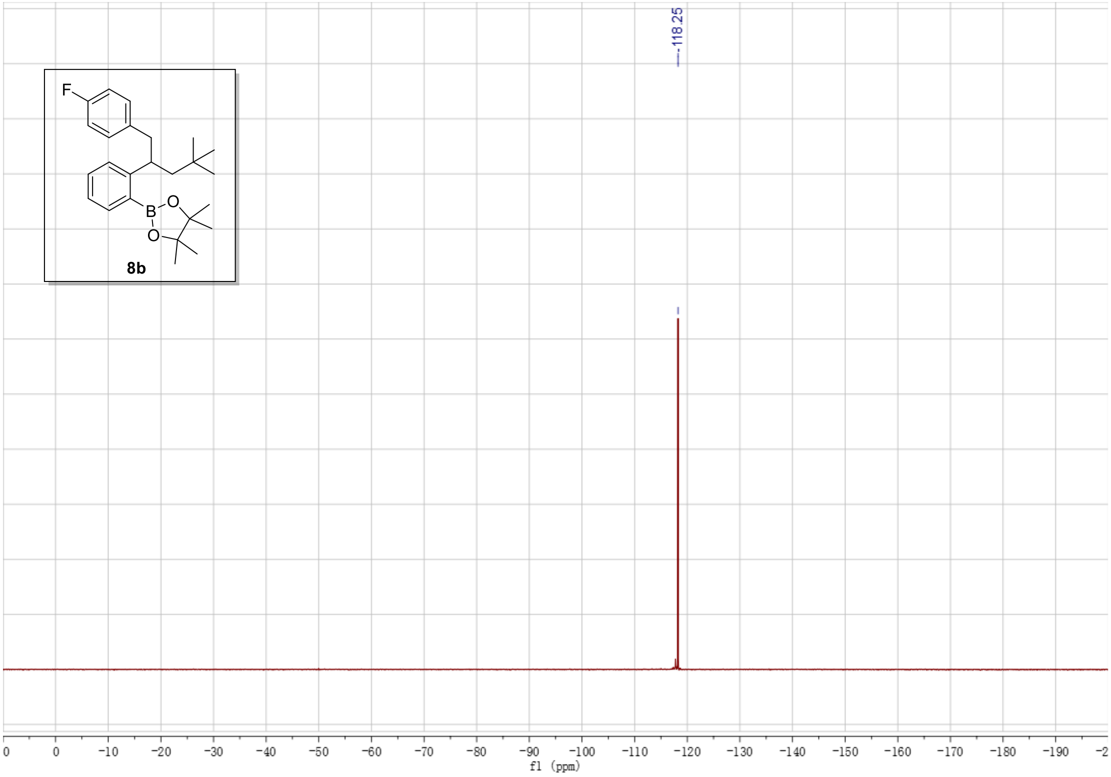

Supplementary Figure 230. ^{19}F spectrum of **8b**.

2-(2-(4,4-dimethyl-1-(p-tolyl)pentan-2-yl)phenyl)-4,4,5,5-tetramethyl-1,3,2-dioxaborolane (8c)

^{1}H spectrum (500 MHz, room temperature,$CDCl_3$) of (**8c**)

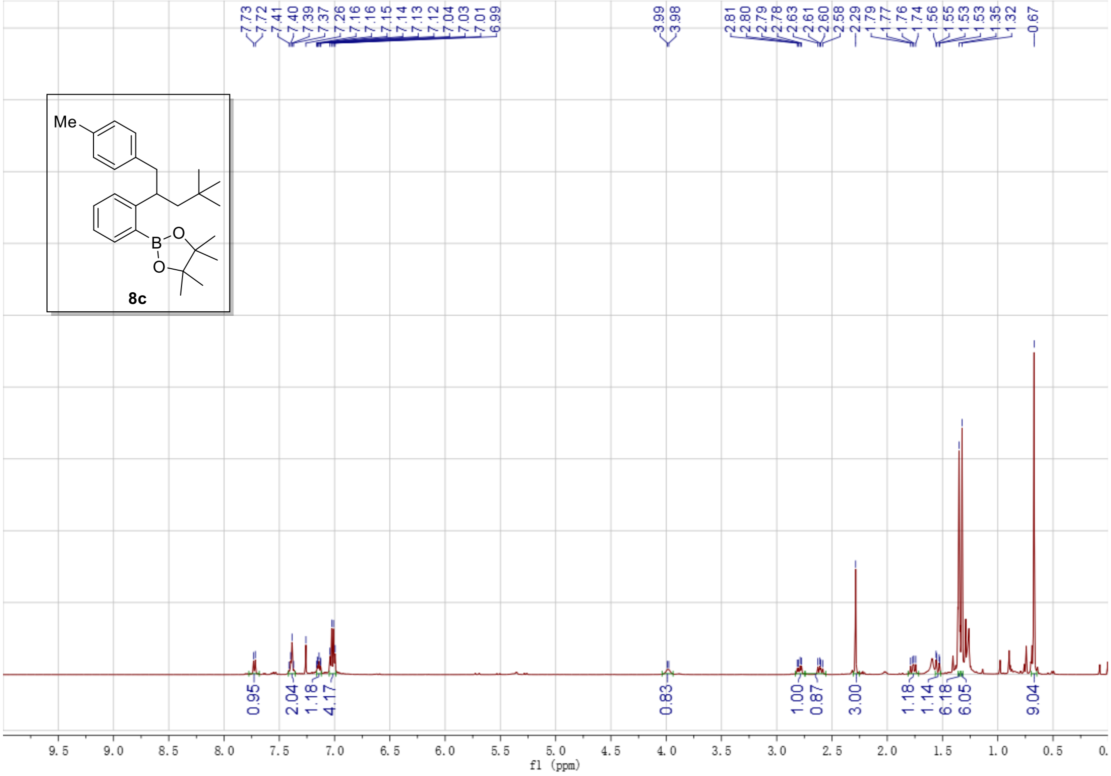

Supplementary Figure 231. ^{1}H NMR spectrum of **8c**.

^{13}C spectrum (126 MHz, room temperature, $CDCl_3$) of (**8c**)

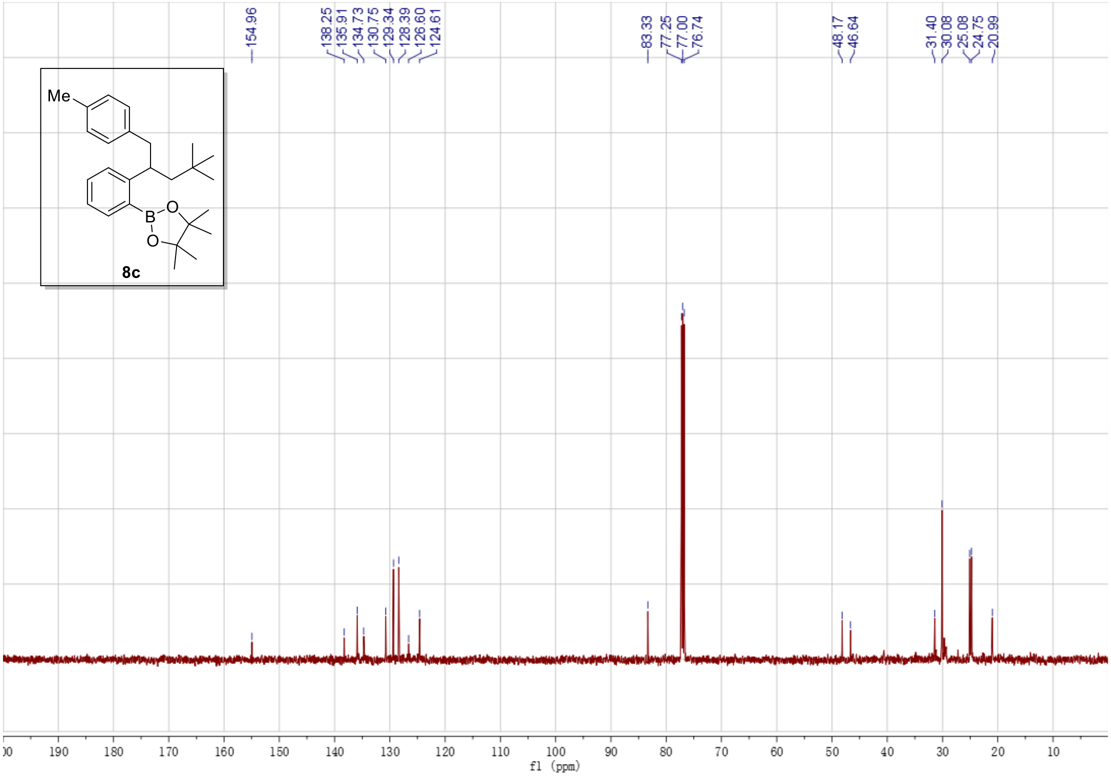

Supplementary Figure 232. ^{13}C NMR spectrum of **8c**.

^{11}B spectrum (160 MHz, room temperature, $CDCl_3$) of (**8c**)

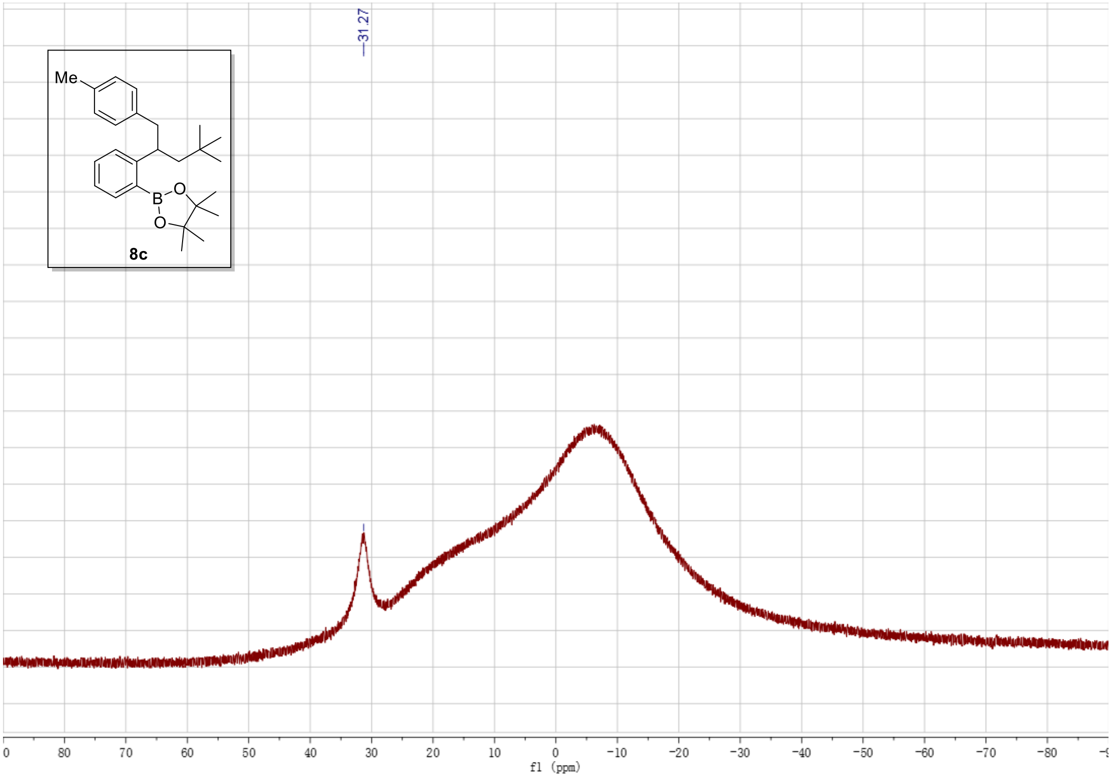

Supplementary Figure 233. ^{11}B spectrum of **8c**.

2-(2-(1-(3-bromophenyl)-4,4-dimethylpentan-2-yl)phenyl)-4,4,5,5-tetramethyl-1,3,2-dioxaborolane (8d)

^{1}H spectrum (400 MHz, room temperature, $CDCl_3$) of (**8d**)

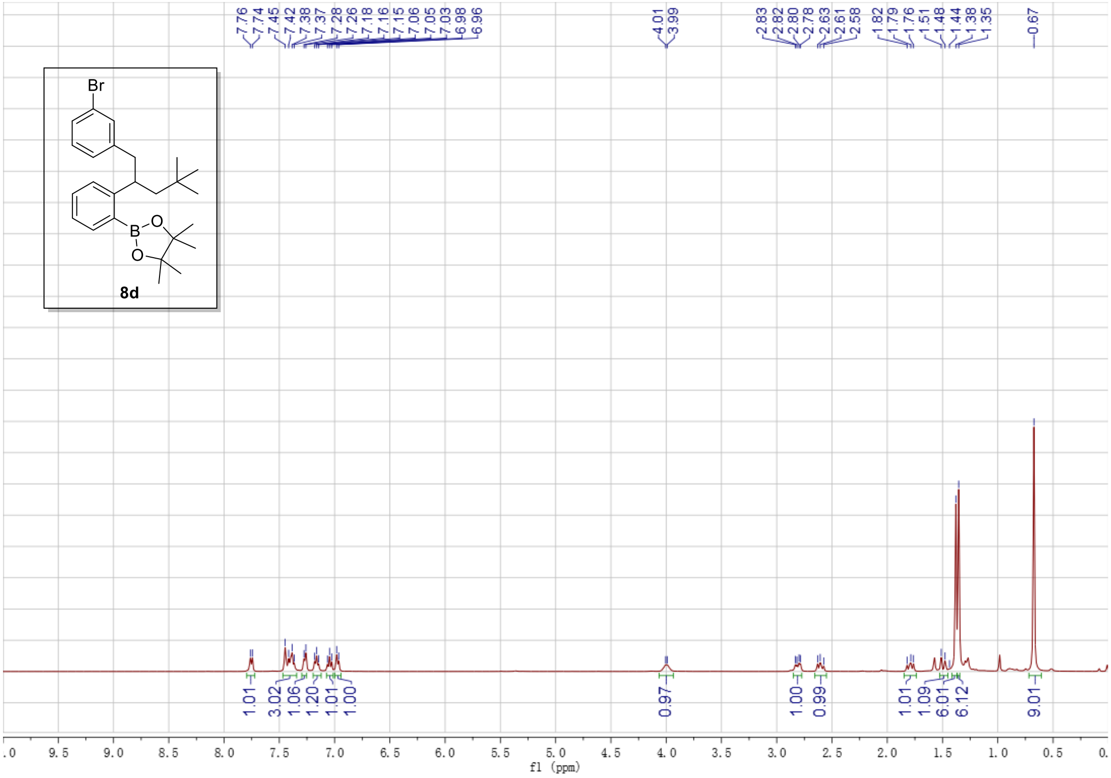

Supplementary Figure 234. ^{1}H NMR spectrum of **8d**.

^{13}C spectrum (126 MHz, room temperature, $CDCl_3$) of (**8d**)

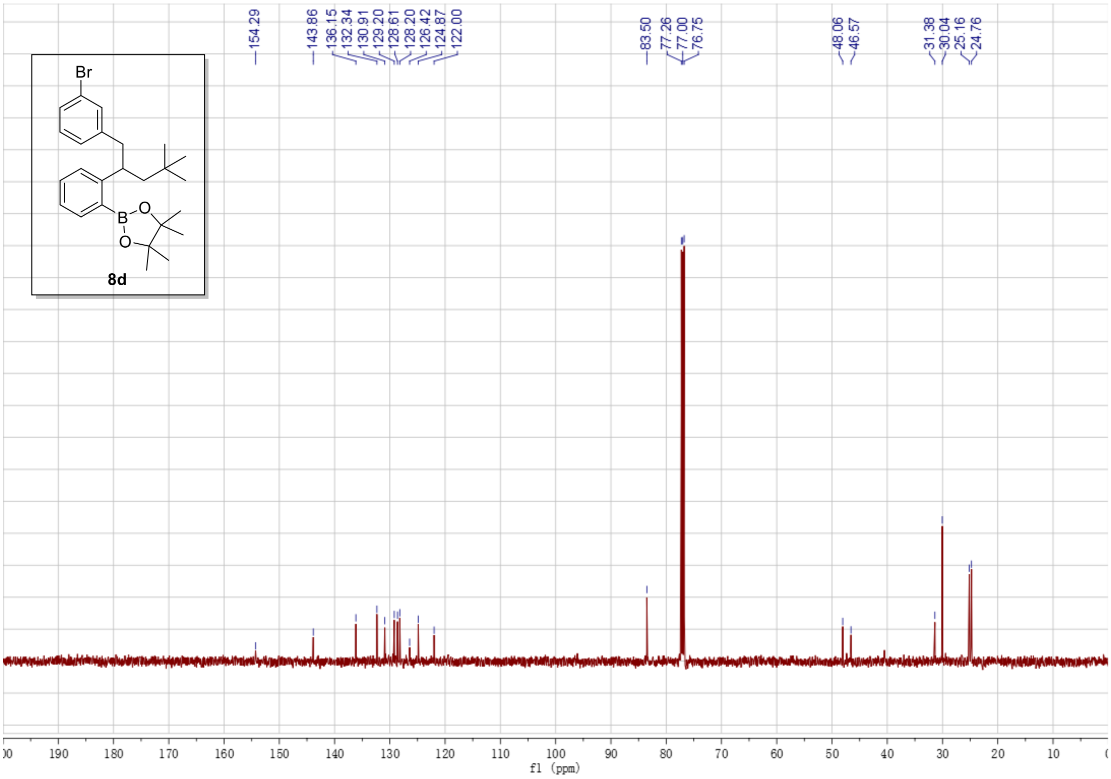

Supplementary Figure 235. ^{13}C NMR spectrum of **8d**.

^{11}B spectrum (160 MHz, room temperature, $CDCl_3$) of (**8d**)

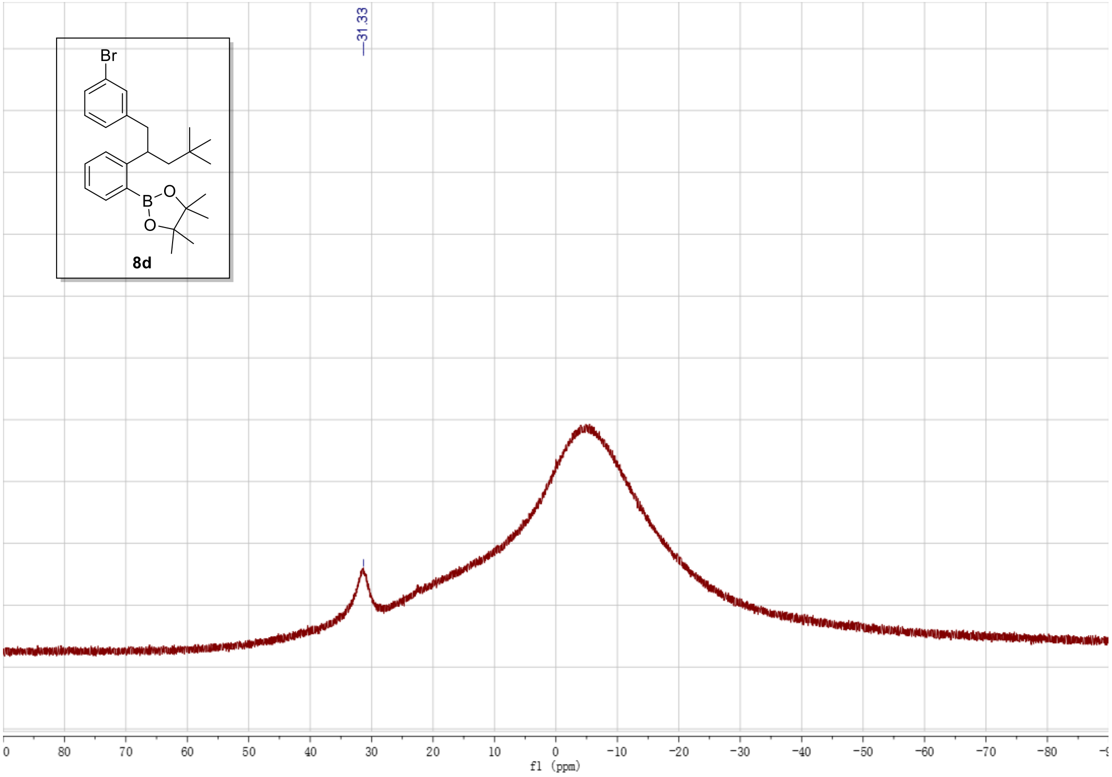

Supplementary Figure 236. ^{11}B spectrum of **8d**.

2-(2-(1-(3-methoxyphenyl)-4,4-dimethylpentan-2-yl)phenyl)-4,4,5,5-tetramethyl-1,3,2-dioxaborolane (8e)

^{1}H spectrum (400 MHz, room temperature, $CDCl_3$) of (**8e**)

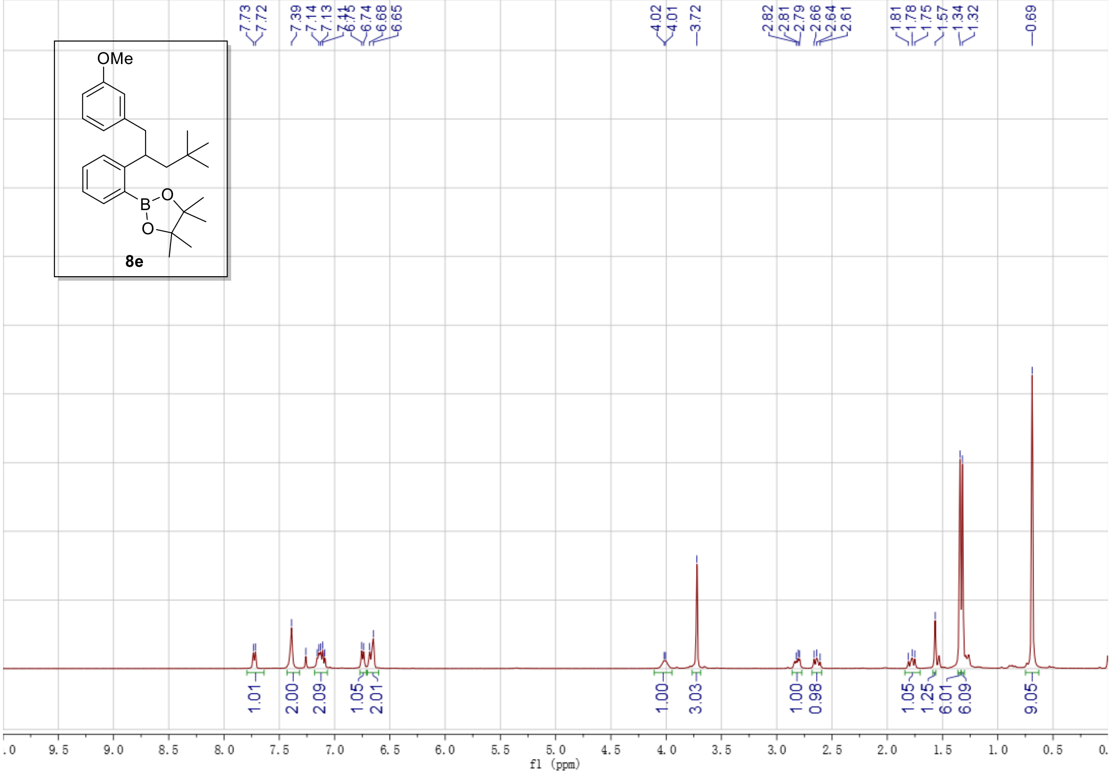

Supplementary Figure 237. ^{1}H NMR spectrum of **8e**.

^{13}C spectrum (126 MHz, room temperature, $CDCl_3$) of (**8e**)

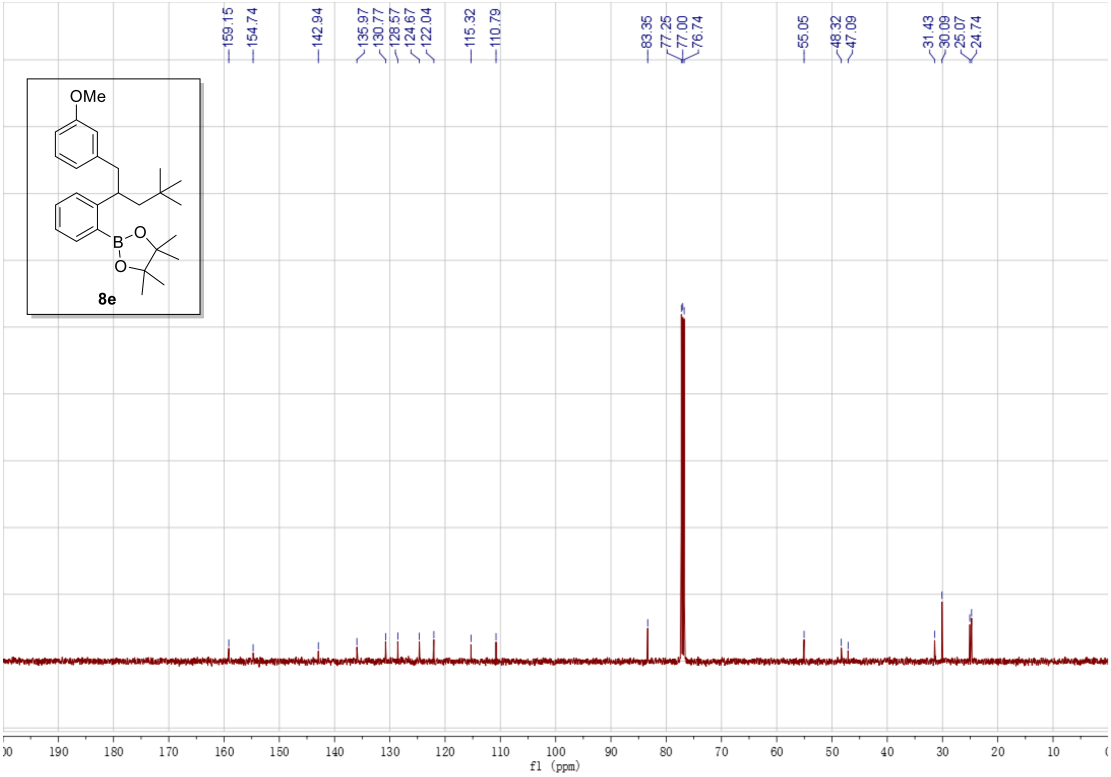

Supplementary Figure 238. ^{13}C NMR spectrum of **8e**.

^{11}B spectrum (128 MHz, room temperature, $CDCl_3$) of (**8e**)

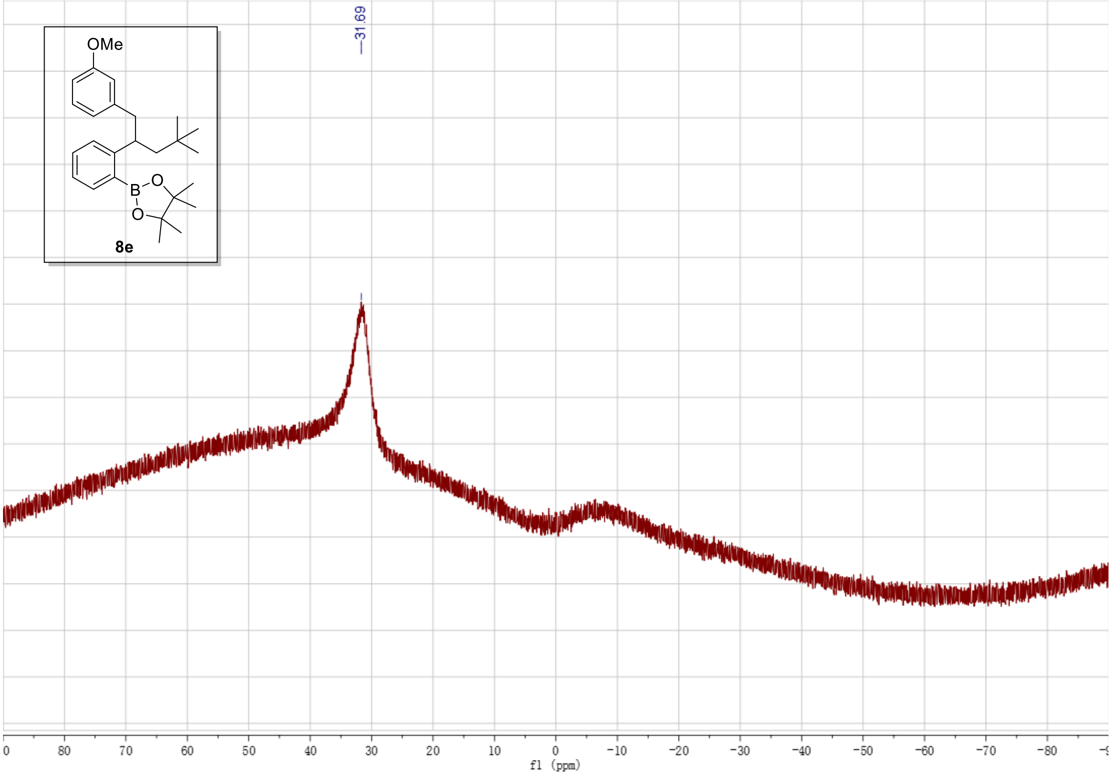

Supplementary Figure 239. ^{11}B spectrum of **8e**.

2-(2-(6,6-dimethylhept-1-en-4-yl)phenyl)-4,4,5,5-tetramethyl-1,3,2-dioxaborolane (8f)

^{1}H spectrum (500 MHz, room temperature, $CDCl_3$) of (**8f**)

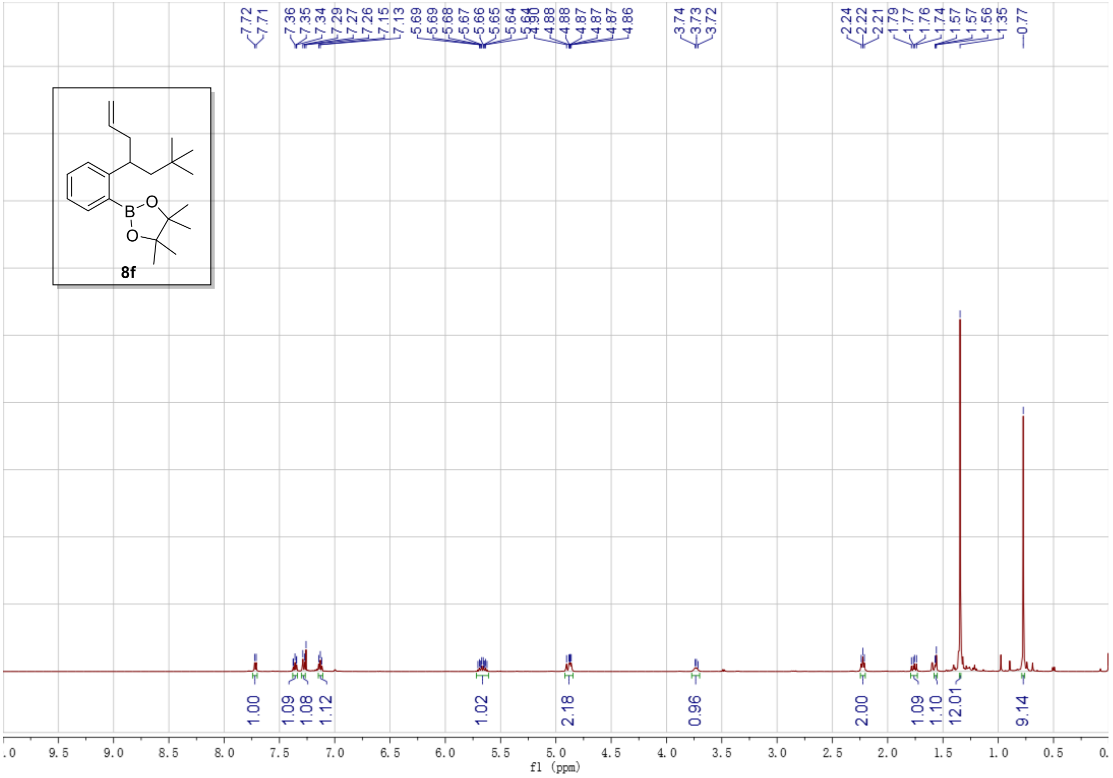

Supplementary Figure 240. ^{1}H NMR spectrum of **8f**.

^{13}C spectrum (126 MHz, room temperature, $CDCl_3$) of (**8f**)

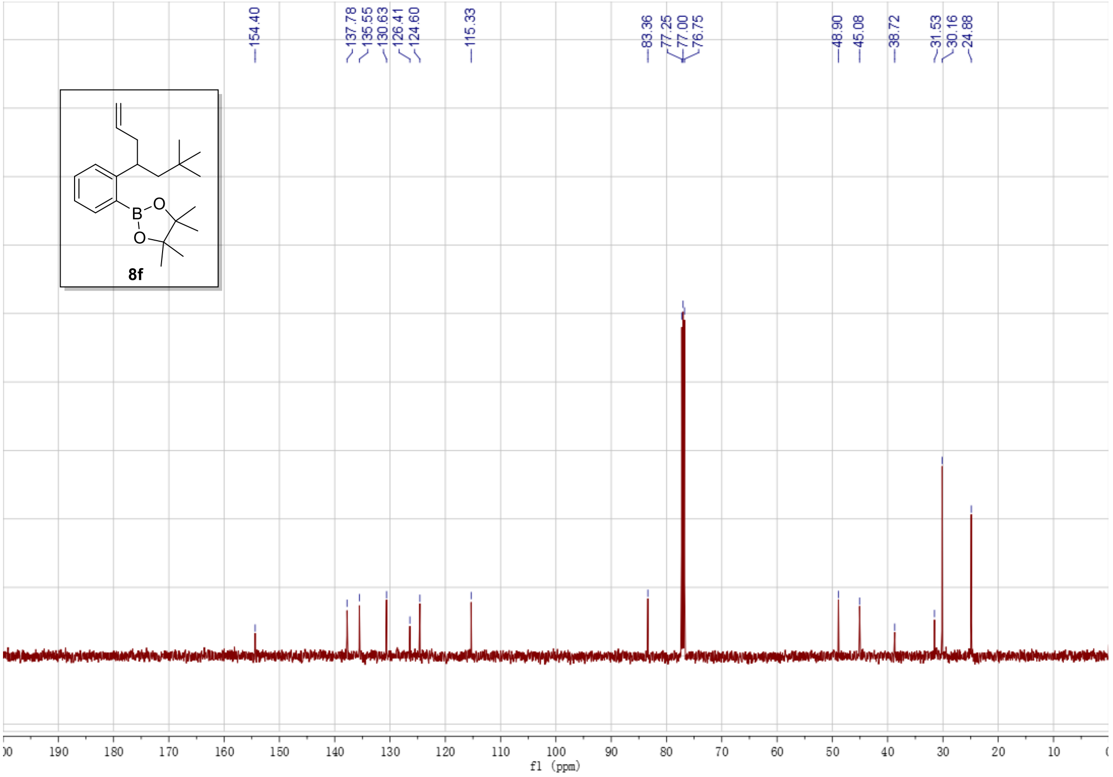

Supplementary Figure 241. ^{13}C NMR spectrum of **8f**.

^{11}B spectrum (128 MHz, room temperature, $CDCl_3$) of (**8f**)

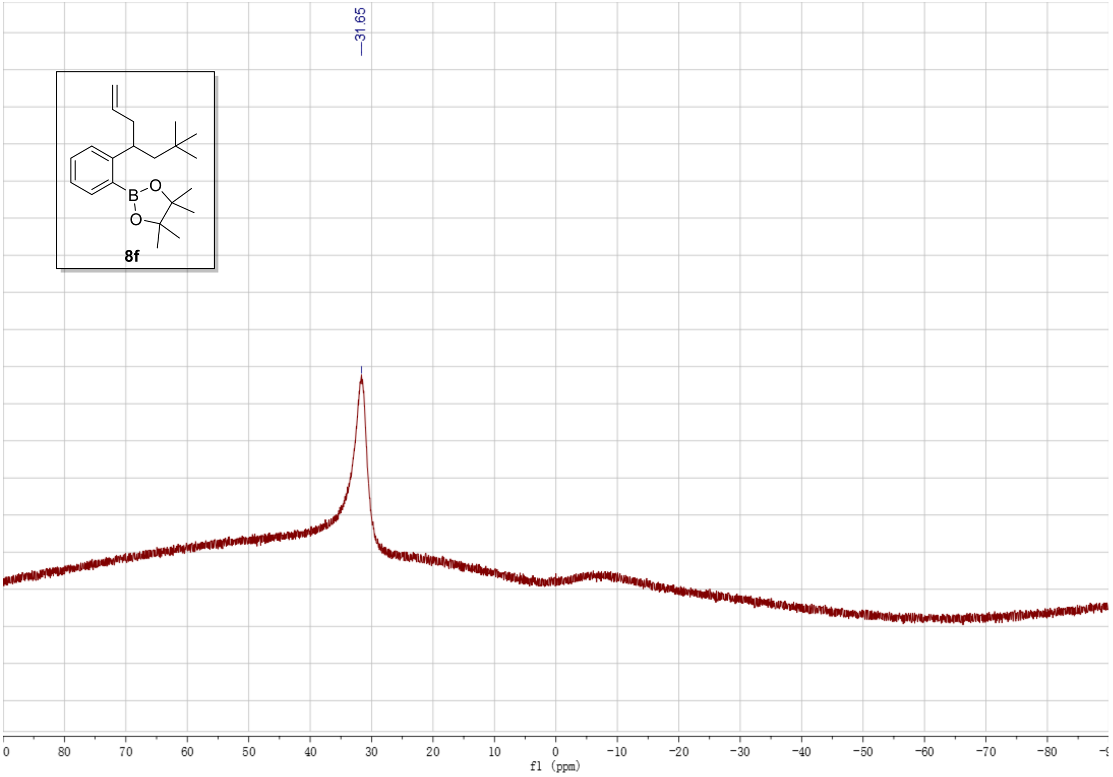

Supplementary Figure 242. ^{11}B spectrum of **8f**.

4,4,5,5-tetramethyl-2-(2-(2,6,6-trimethylhept-1-en-4-yl)phenyl)-1,3,2-dioxaborolane (8g)

^{1}H spectrum (500 MHz, room temperature, $CDCl_3$) of (**8g**)

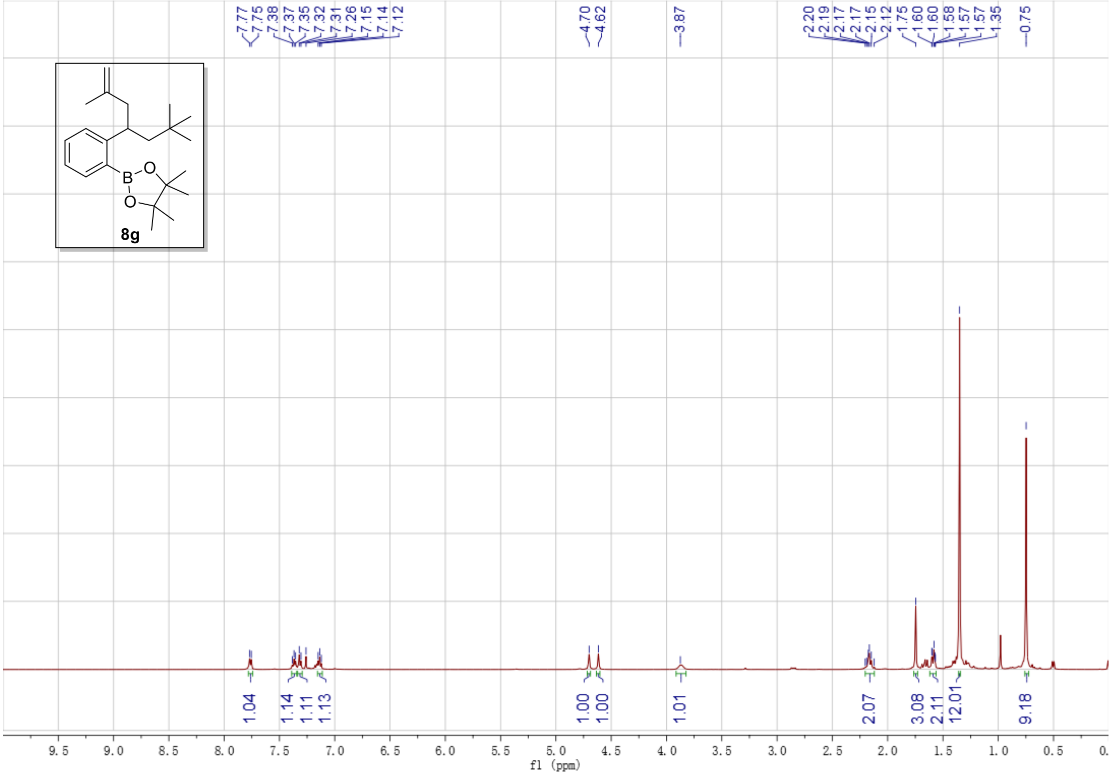

Supplementary Figure 243. ^{1}H NMR spectrum of **8g**.

^{13}C spectrum (126 MHz, room temperature, $CDCl_3$) of (**8g**)

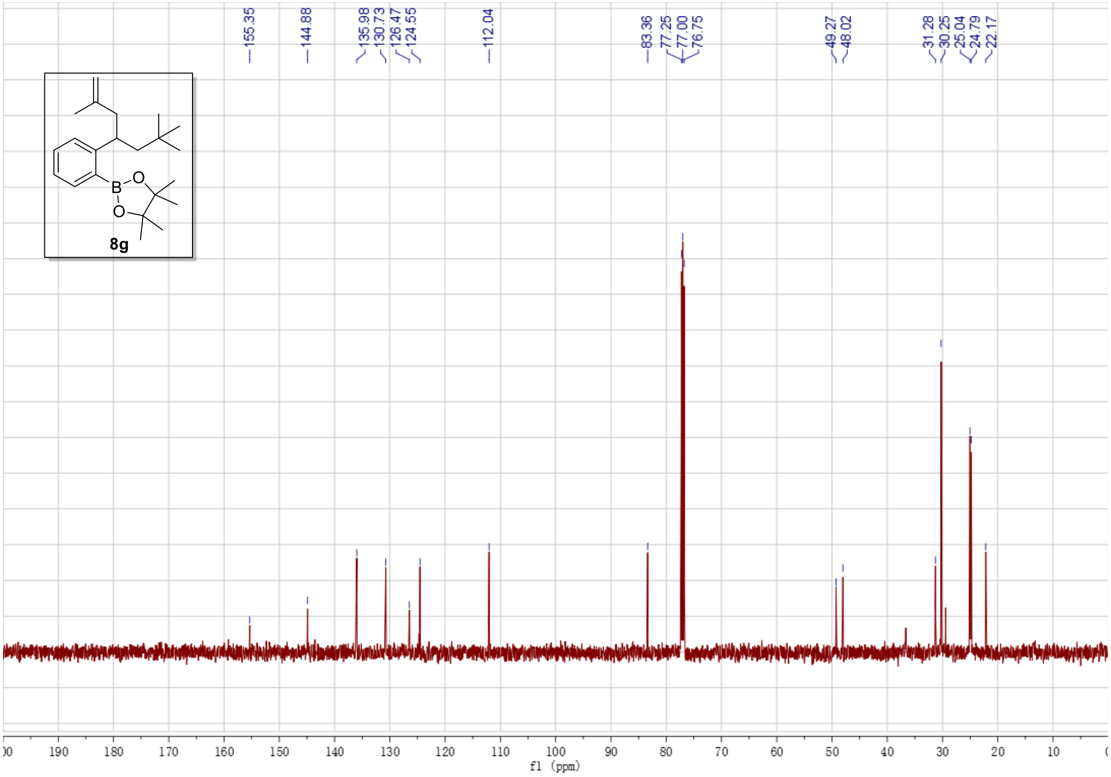

Supplementary Figure 244. ^{13}C NMR spectrum of **8g**.

^{11}B spectrum (128 MHz, room temperature, $CDCl_3$) of

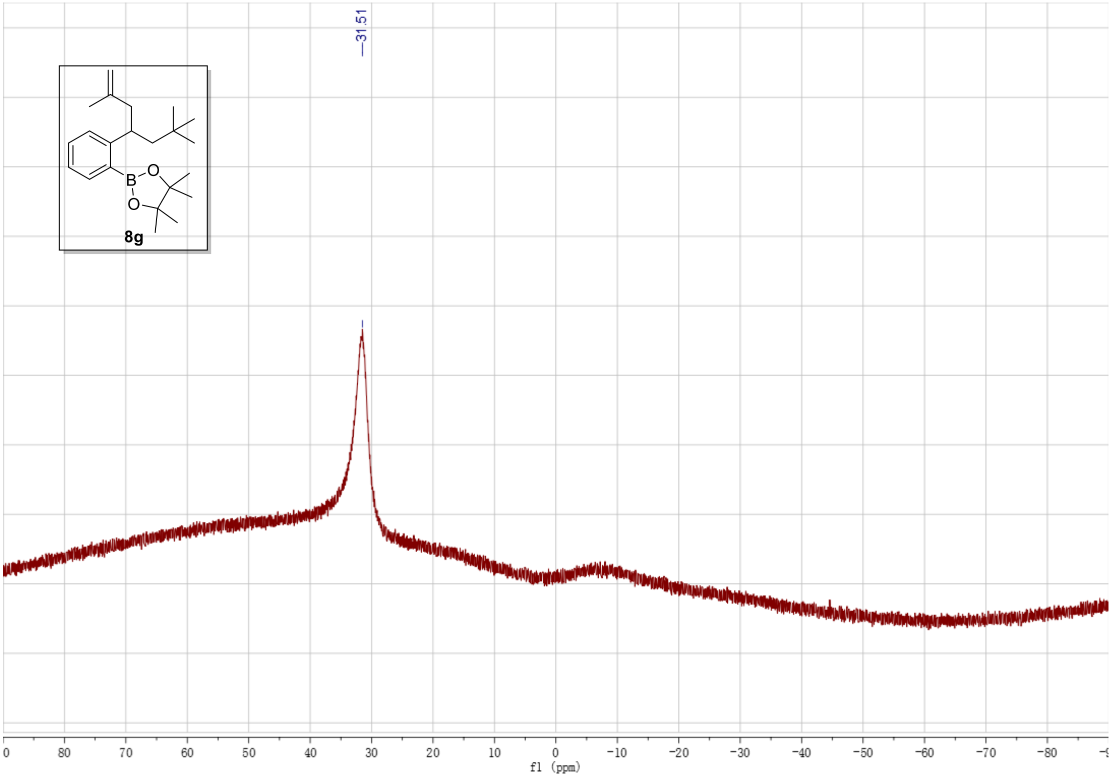

Supplementary Figure 245. ^{11}B spectrum of **8g**.

2-(2-(1-(cyclohex-2-en-1-yl)-3,3-dimethylbutyl)phenyl)-4,4,5,5-tetramethyl-1,3,2-dioxaborolane (8h)

^{1}H spectrum (500 MHz, room temperature, $CDCl_3$) of (**8h**)

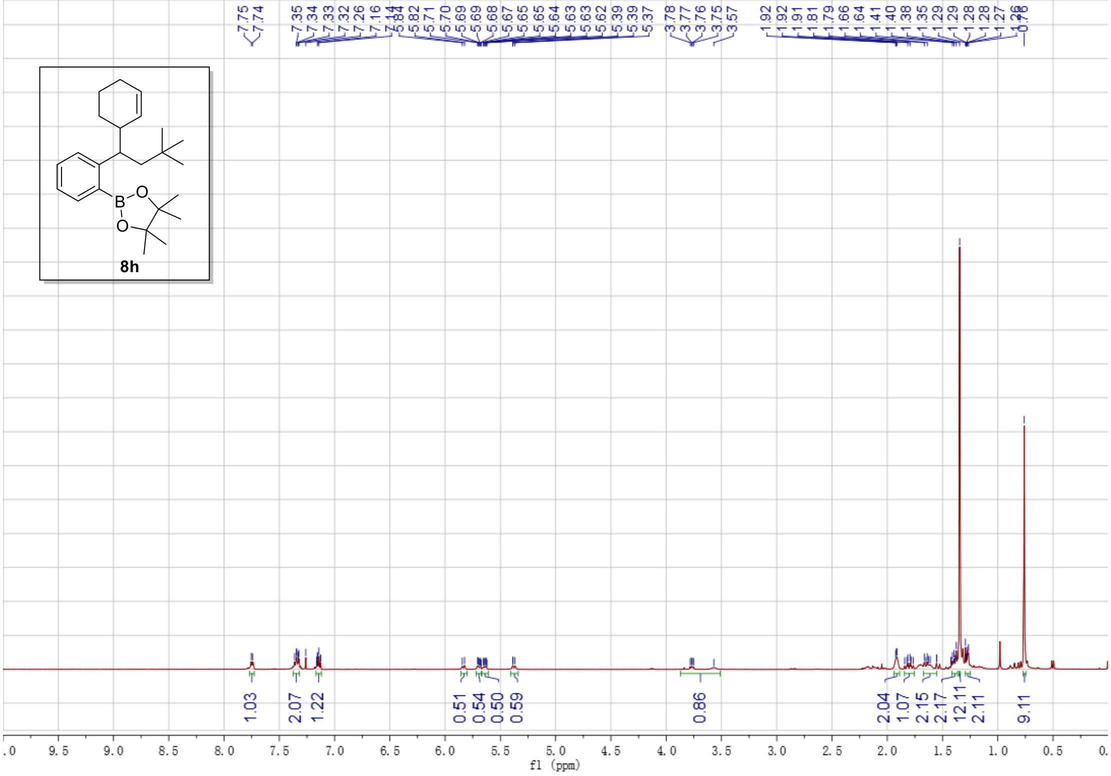

Supplementary Figure 246. ^{1}H NMR spectrum of **8h**.

^{13}C spectrum (126 MHz, room temperature, $CDCl_3$) of (**8h**)

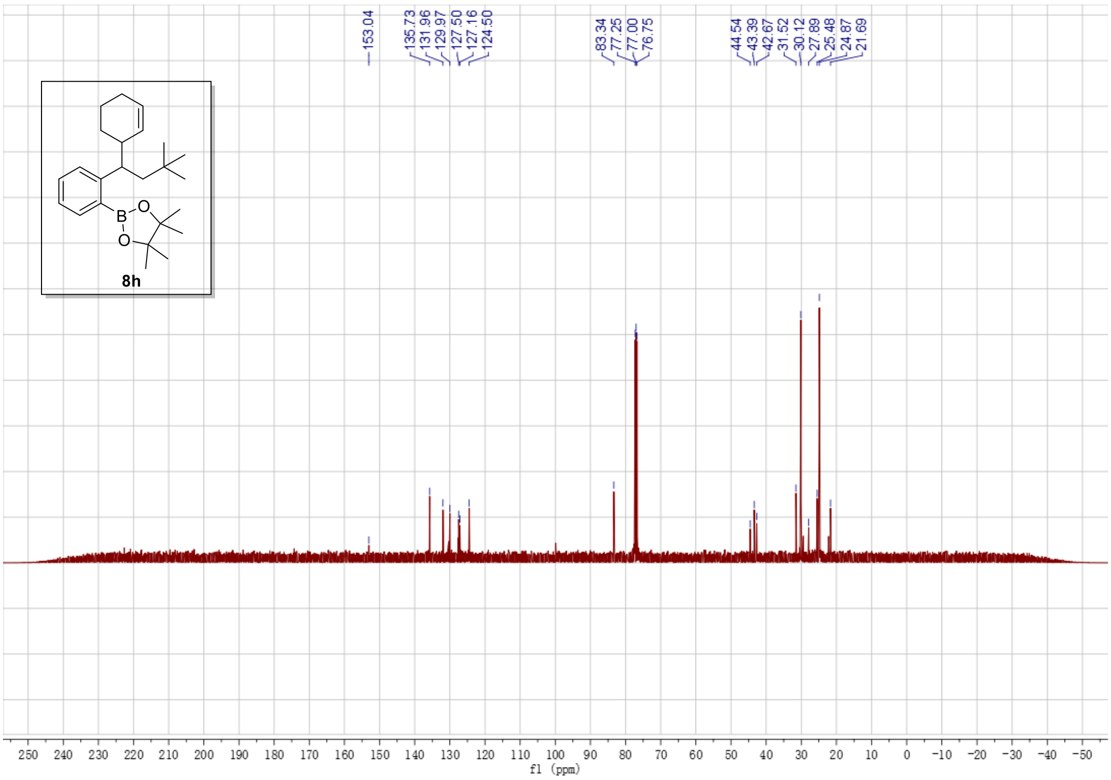

Supplementary Figure 247. ^{13}C NMR spectrum of **8h**.

^{11}B spectrum (160 MHz, room temperature, $CDCl_3$) of (**8h**)

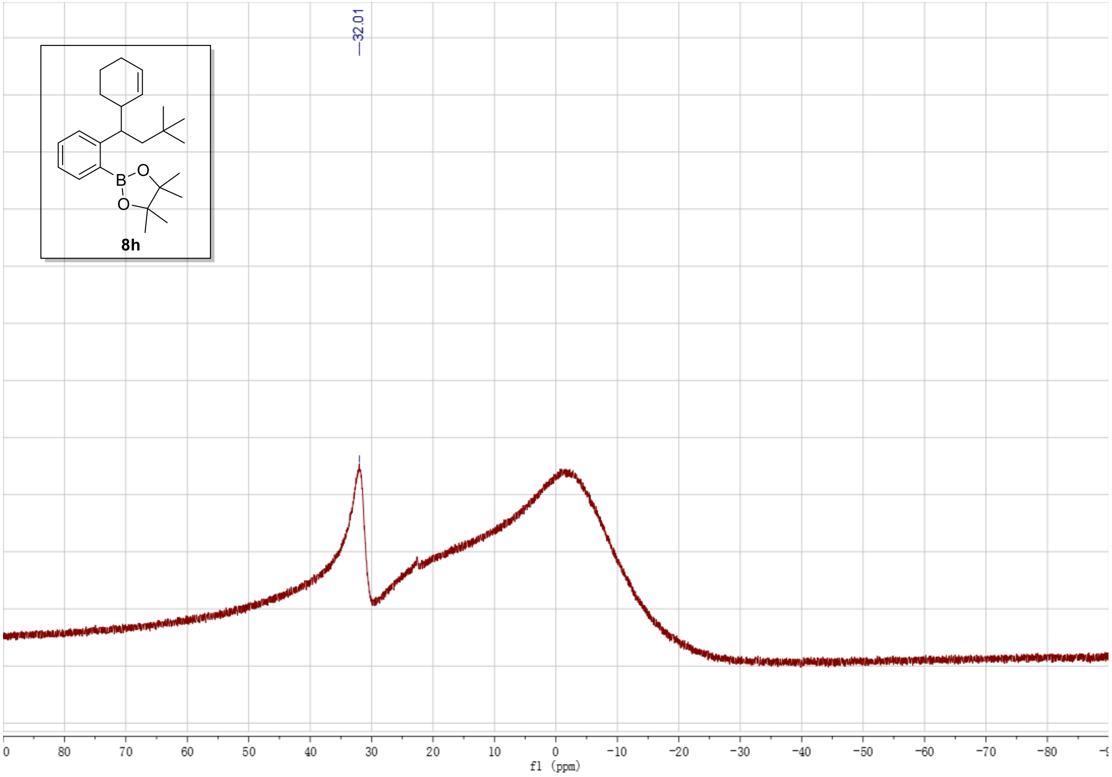

Supplementary Figure 248. ^{11}B spectrum of **8h**.

1-benzyl-2-(3,3-dimethylbutyl)-4-methoxybenzene (9)

^{1}H spectrum (500 MHz, room temperature, $CDCl_3$) of (**9**)

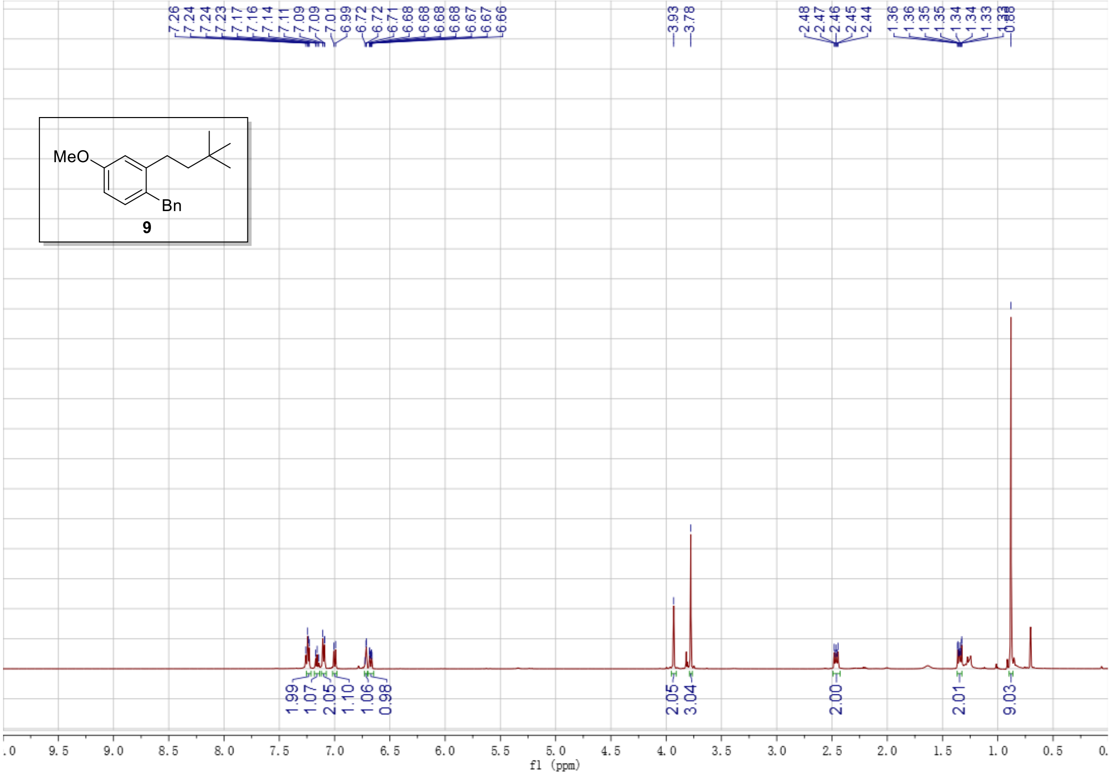

Supplementary Figure 249. ^{1}H NMR spectrum of **9**.

^{13}C spectrum (126 MHz, room temperature, $CDCl_3$) of (**9**)

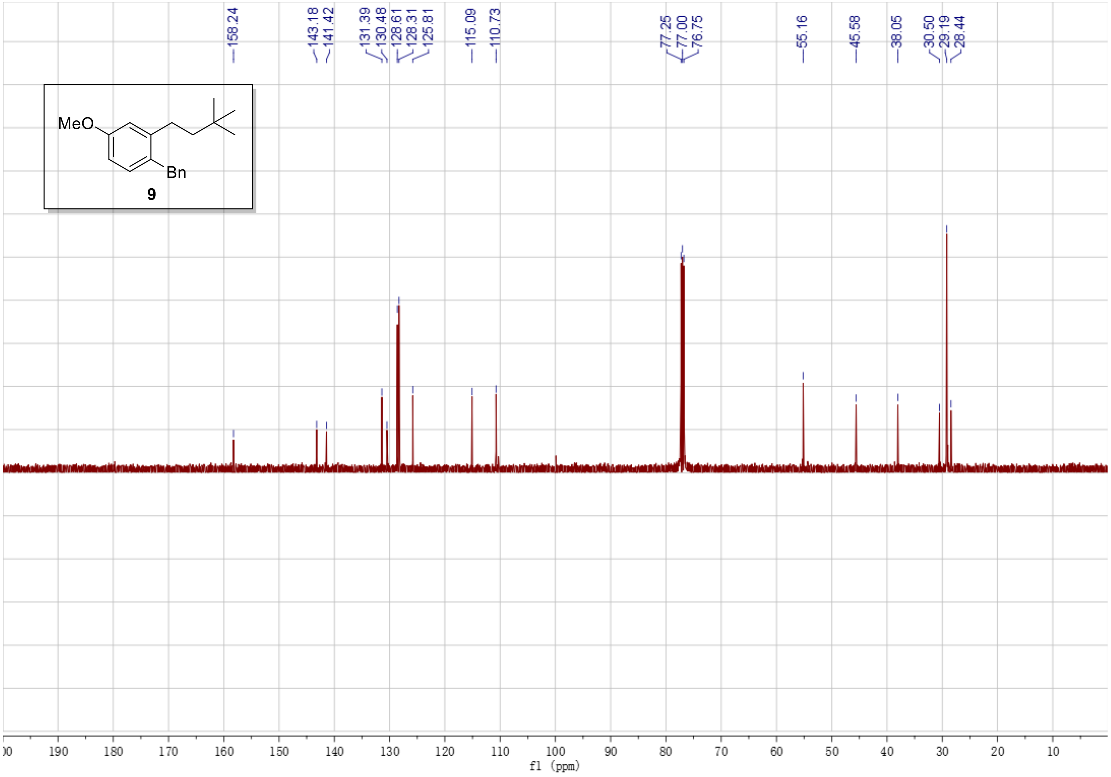

Supplementary Figure 250. ^{13}C NMR spectrum of **9**.

2-(3,3-dimethylbutyl)-4-methoxy-1-methylbenzene (10)

^{1}H spectrum (500 MHz, room temperature, $CDCl_3$) of (**10**)

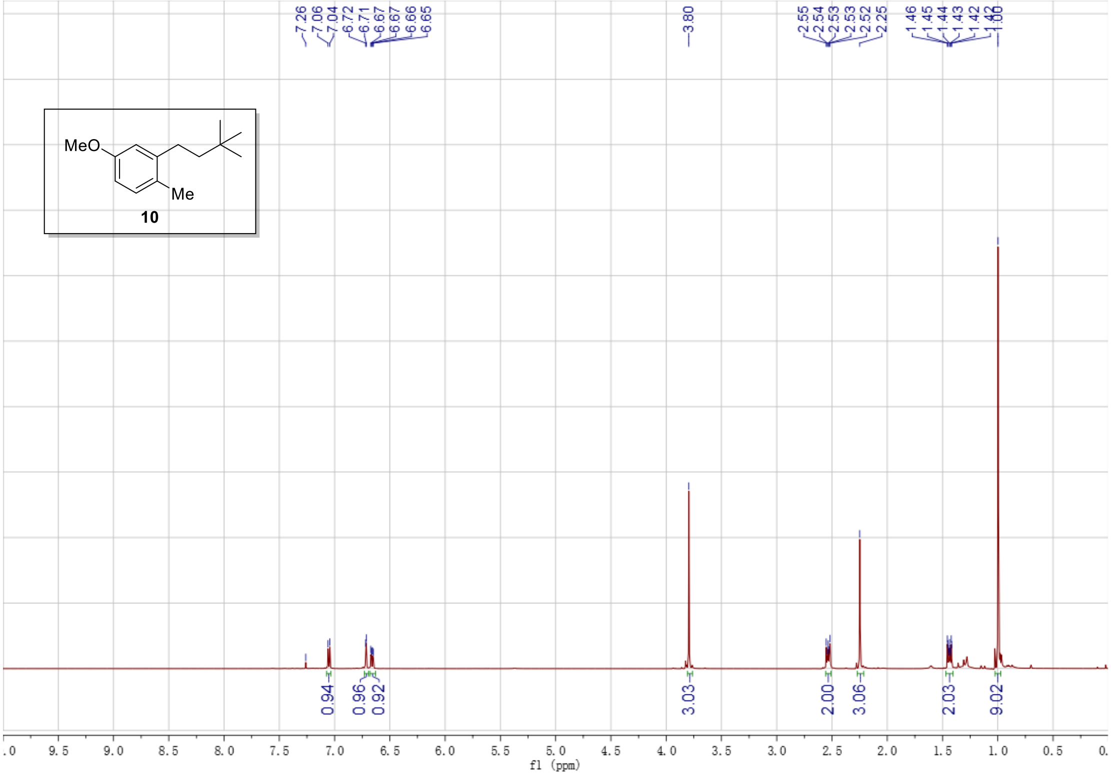

Supplementary Figure 251. ^{1}H NMR spectrum of **10**.

^{13}C spectrum (126 MHz, room temperature, $CDCl_3$) of (**10**)

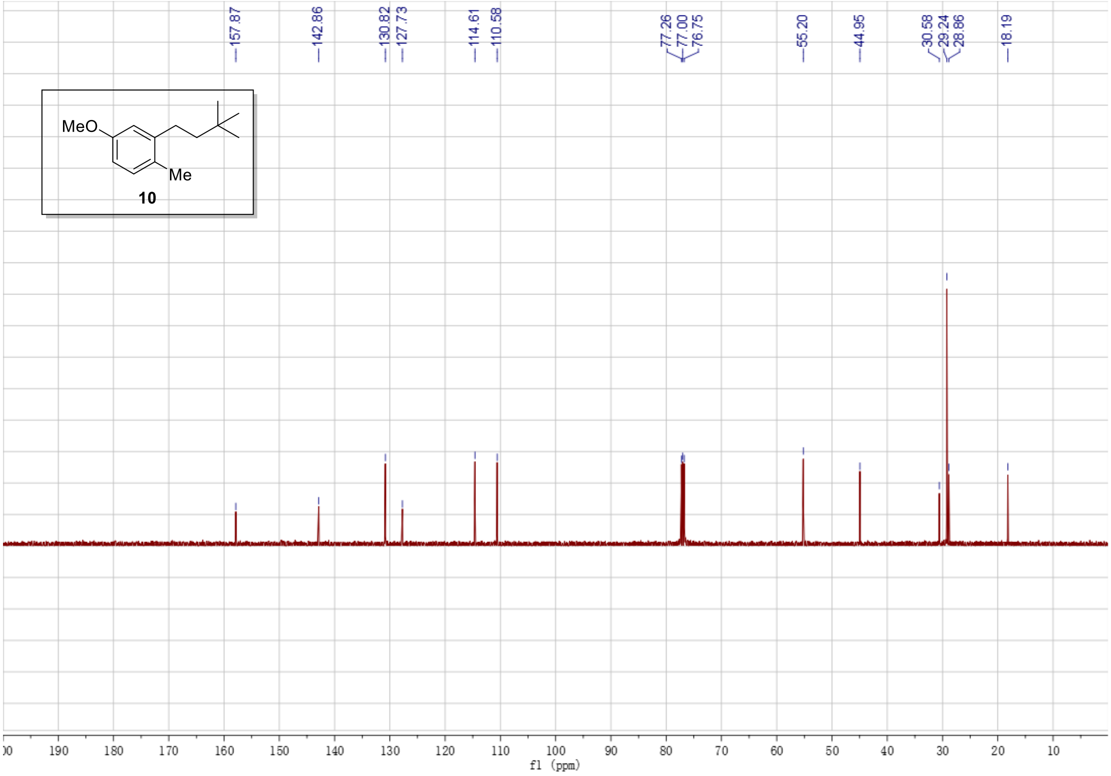

Supplementary Figure 252. ^{13}C NMR spectrum of **10**.

2-(2-(3,3-dimethylbutyl)-4-methoxyphenyl)thiophene (11)

^{1}H spectrum (500 MHz, room temperature, $CDCl_3$) of (**11**)

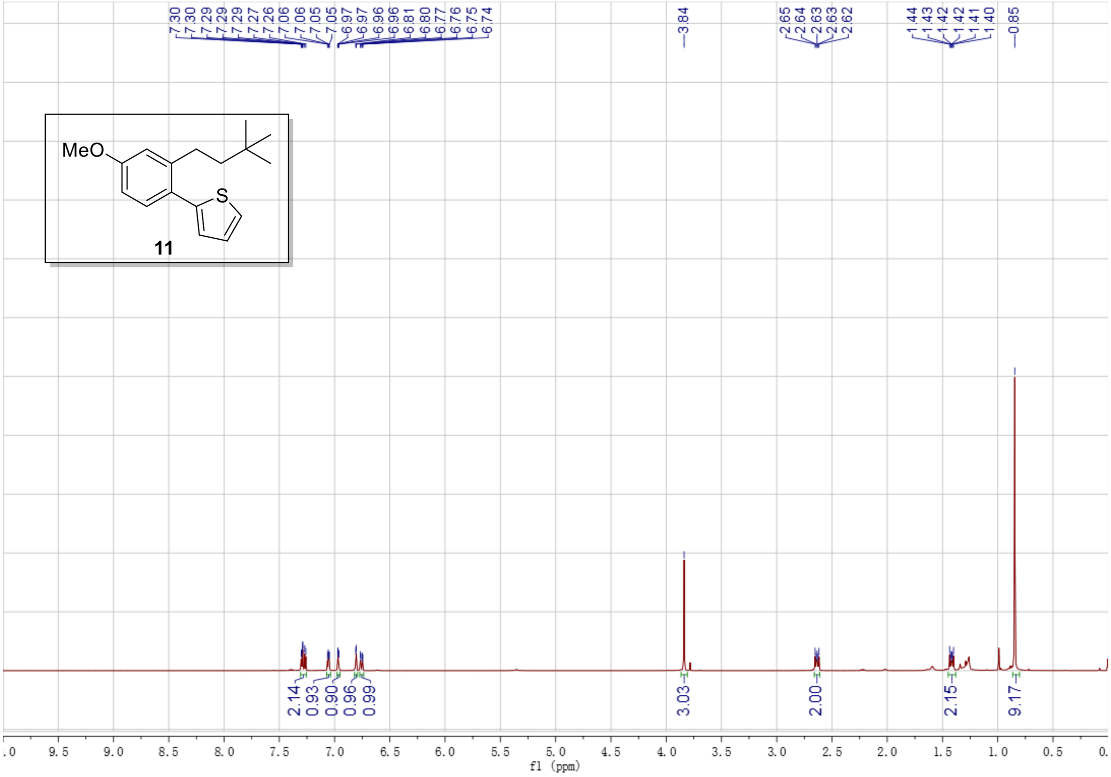

Supplementary Figure 253. ^{1}H NMR spectrum of **11**.

^{13}C spectrum (126 MHz, room temperature, $CDCl_3$) of (**11**)

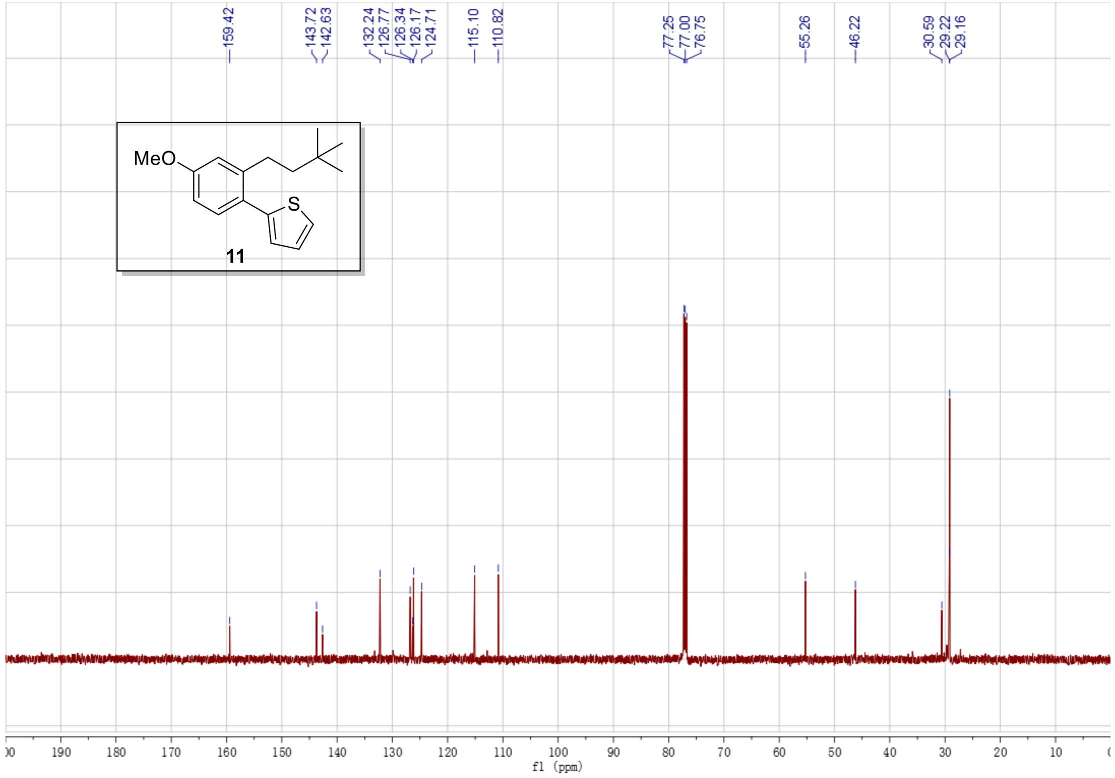

Supplementary Figure 254. ^{13}C NMR spectrum of **11**.

2-(3-ethyl-3-(hydroxymethyl)pentyl)phenol (12)

^{1}H spectrum (400 MHz, room temperature, $CDCl_3$) of (**12**)

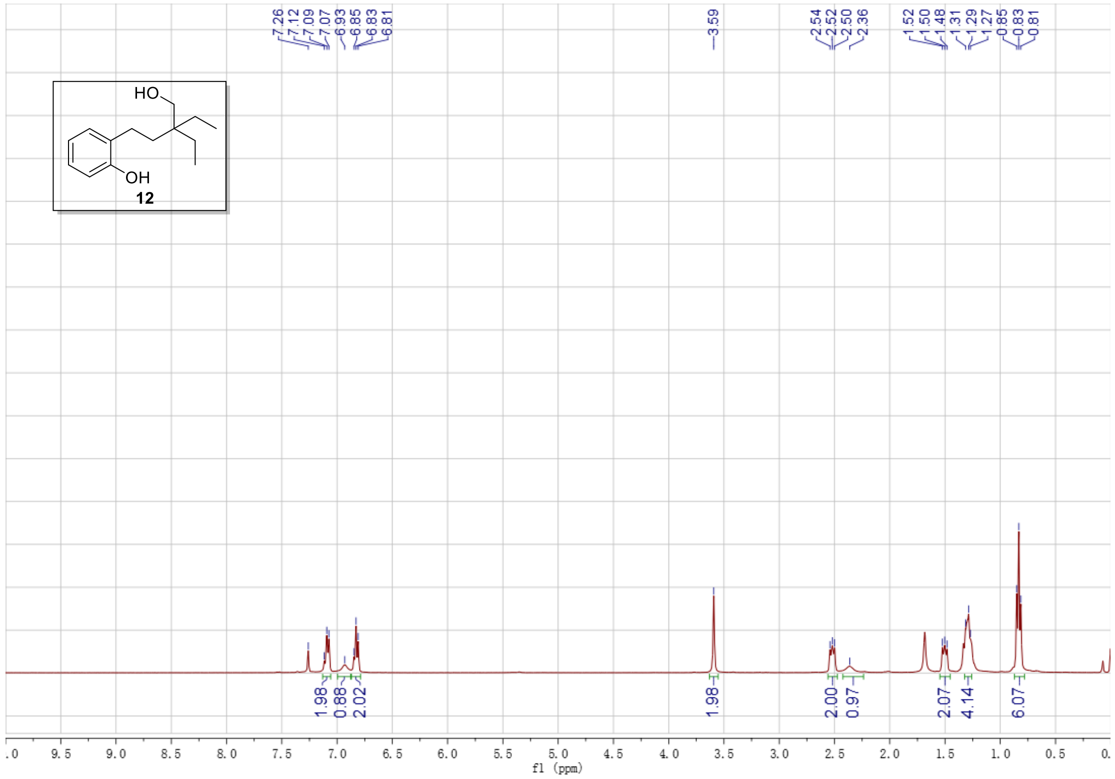

Supplementary Figure 255. ^{1}H NMR spectrum of **12**.

^{13}C spectrum (126 MHz, room temperature, $CDCl_3$) of (**12**)

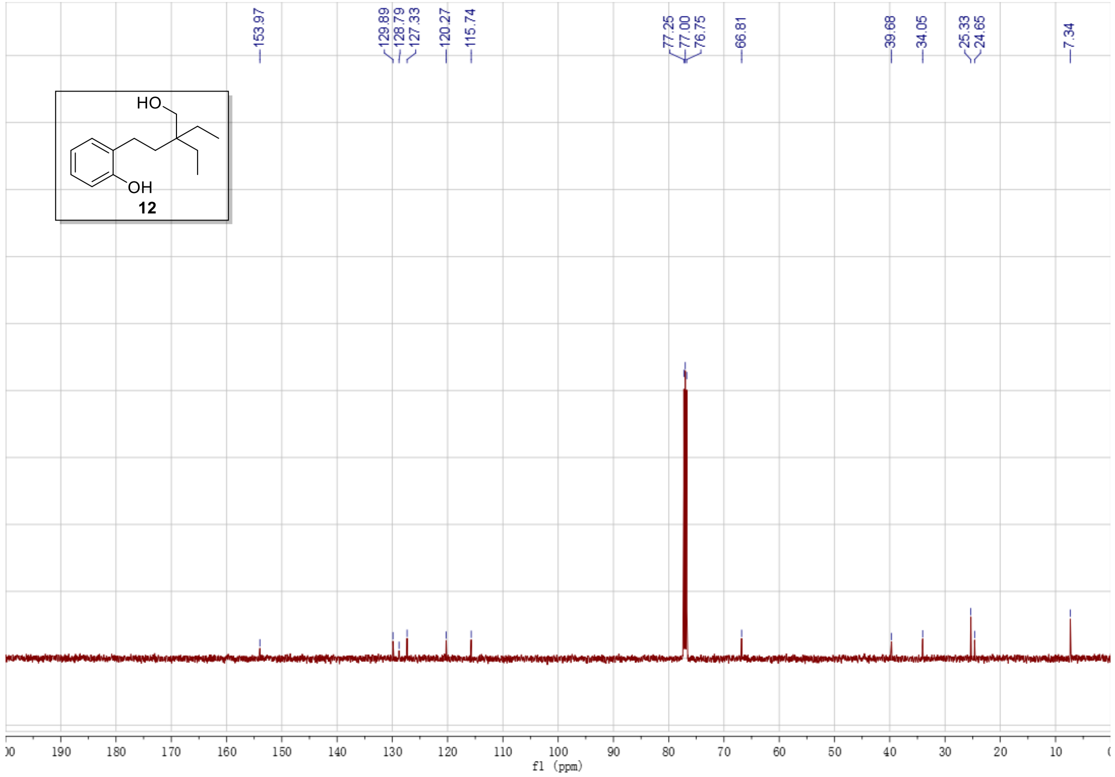

Supplementary Figure 256. ^{13}C NMR spectrum of **12**.

2-(4-([1,1'-biphenyl]-2-yl)-2,2-diethylbutyl)-4,4,5,5-tetramethyl-1,3,2-dioxaborolane (13)

^{1}H spectrum (500 MHz, room temperature, $CDCl_3$) of **(13)**

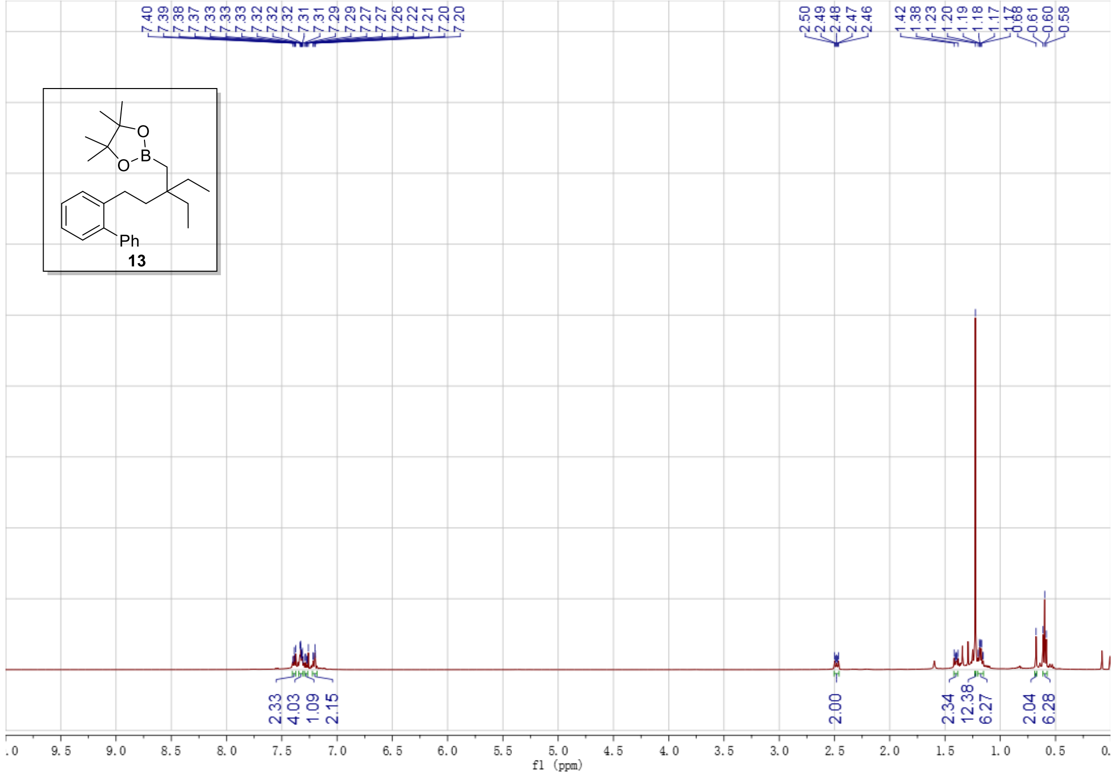

Supplementary Figure 257. ^{1}H NMR spectrum of **13**.

^{13}C spectrum (126 MHz, room temperature, $CDCl_3$) of (**13**)

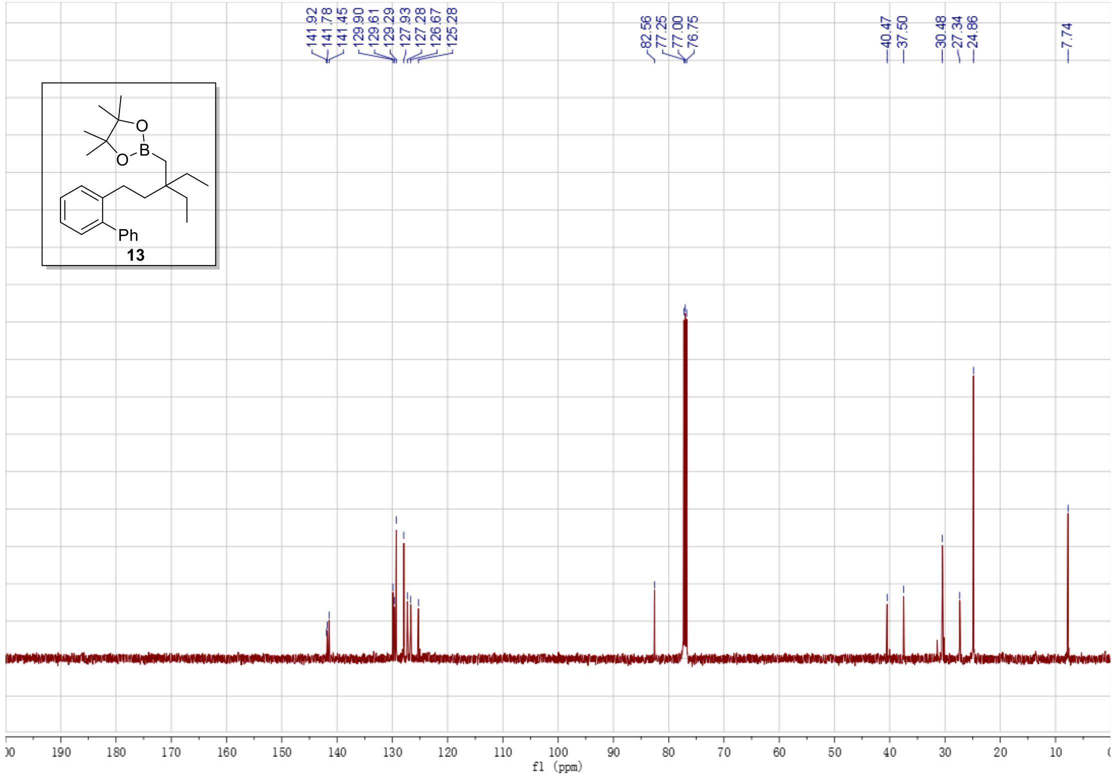

Supplementary Figure 258. ^{13}C NMR spectrum of **13**.

^{11}B spectrum (160 MHz, room temperature, $CDCl_3$) of (**13**)

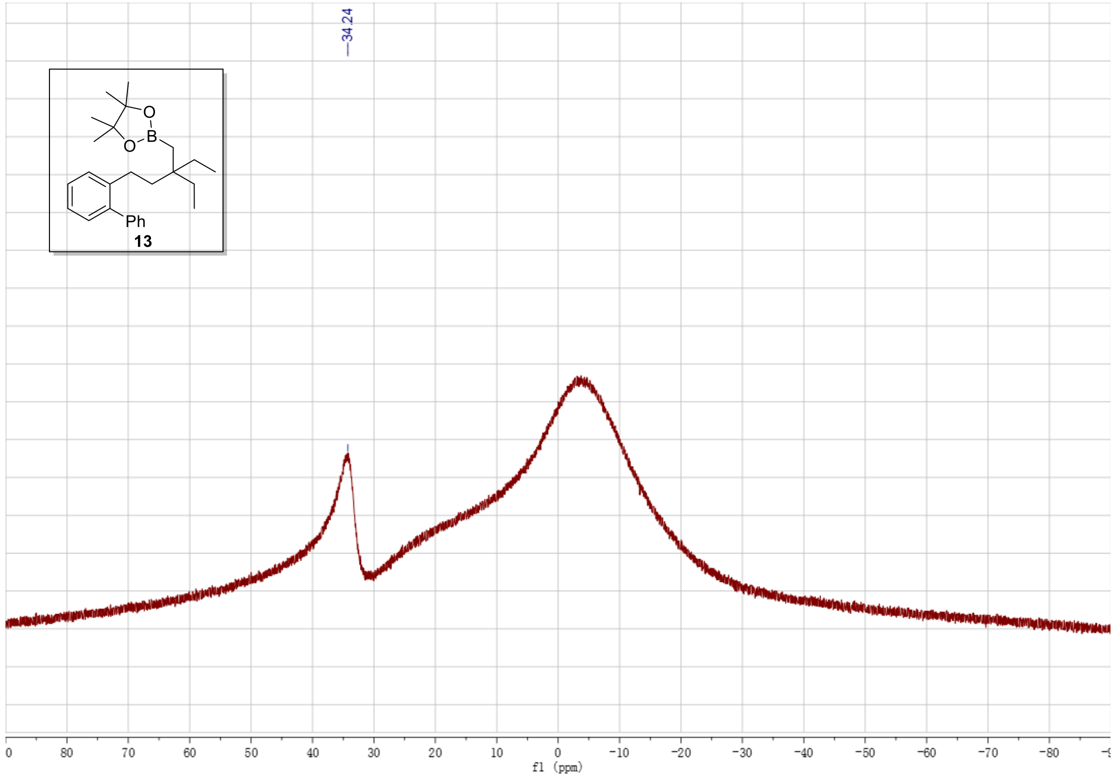

Supplementary Figure 259. ^{11}B spectrum of **13**.

2-(5-([1,1'-biphenyl]-2-yl)-3,3-diethylpentyl)-4,4,5,5-tetramethyl-1,3,2-dioxaborolane (14)

^{1}H spectrum (500 MHz, room temperature, $CDCl_3$) of **(14)**

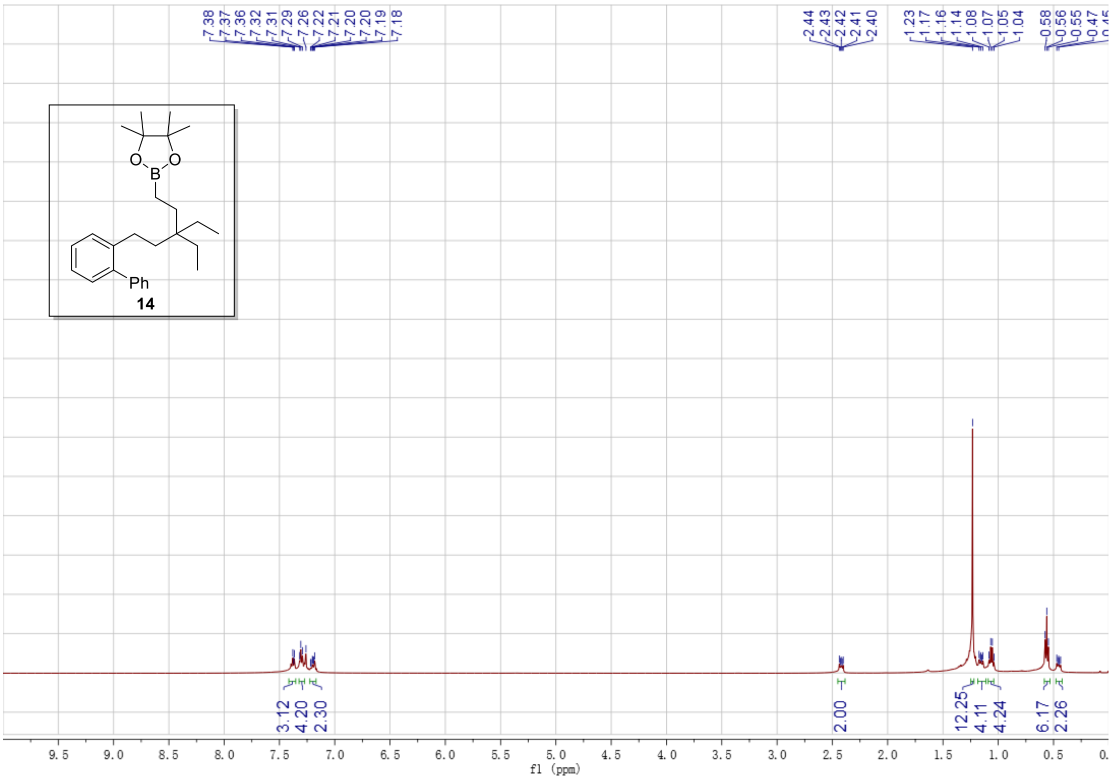

Supplementary Figure 260. ^{1}H NMR spectrum of **14**.

^{13}C spectrum (126 MHz, room temperature, $CDCl_3$) of (**14**)

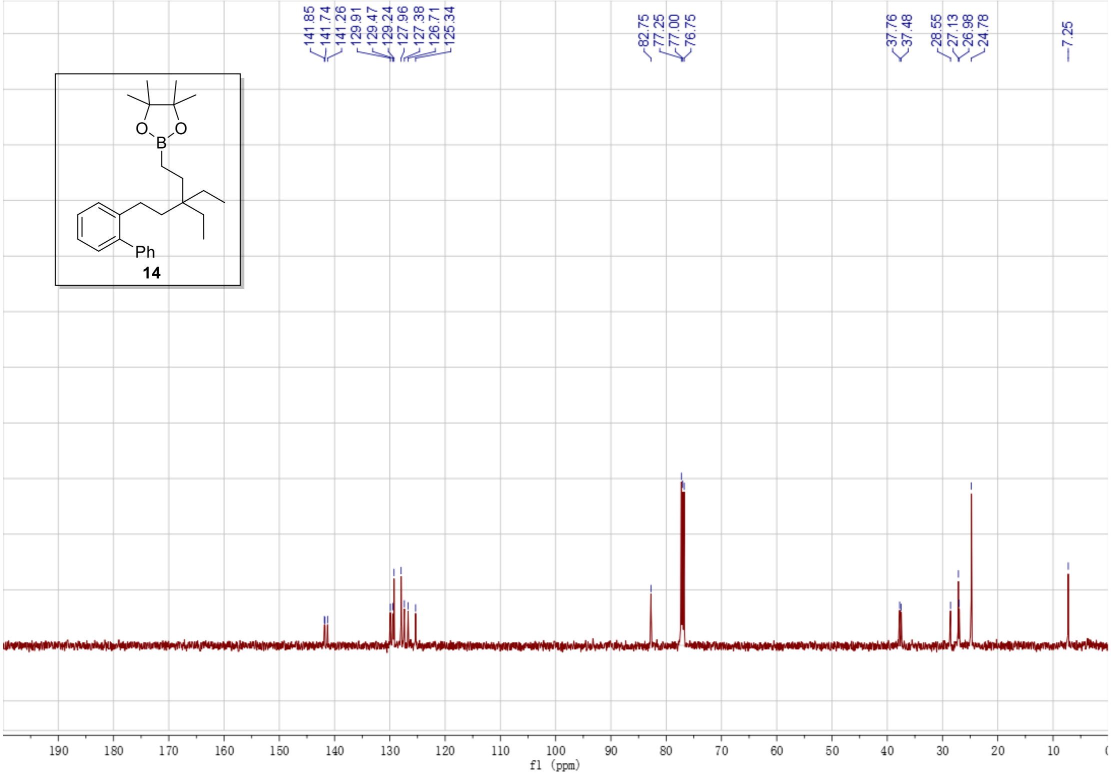

Supplementary Figure 261. ^{13}C NMR spectrum of **14**.

^{11}B spectrum (160 MHz, room temperature, $CDCl_3$) of (**14**)

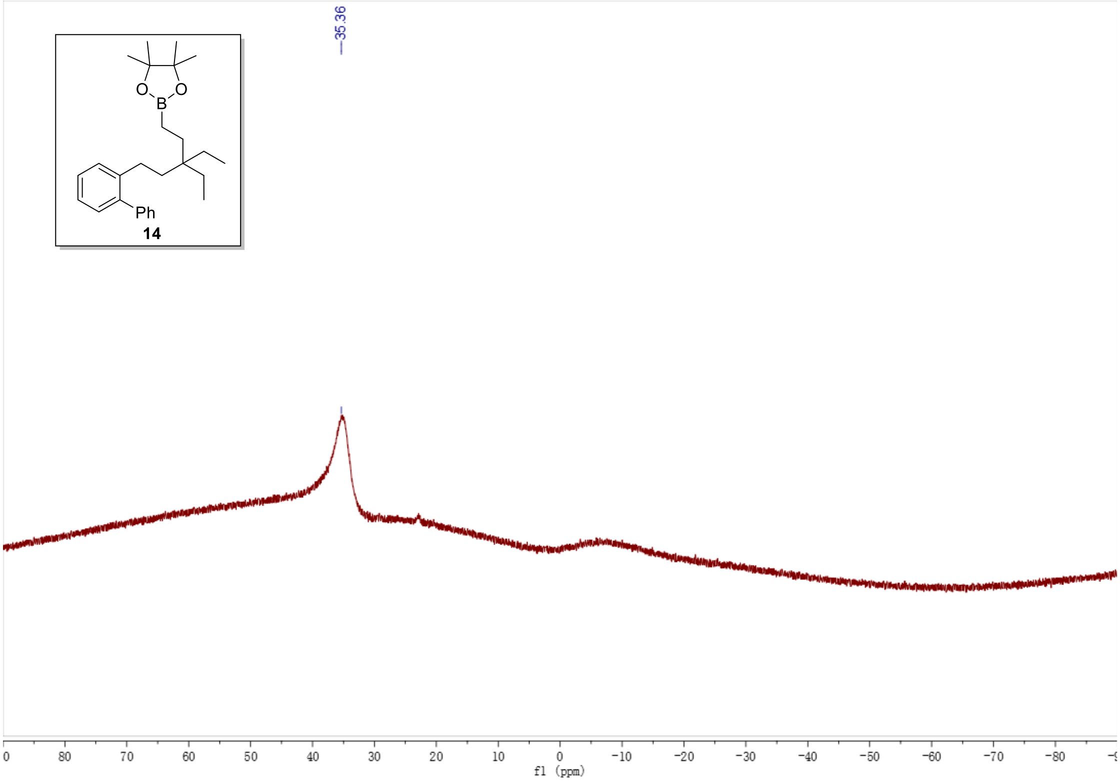

Supplementary Figure 262. ^{11}B spectrum of **14**.

2-(3,3-diethylhex-5-en-1-yl)-1,1'-biphenyl (15)

^{1}H spectrum (500 MHz, room temperature, $CDCl_3$) of **(15)**

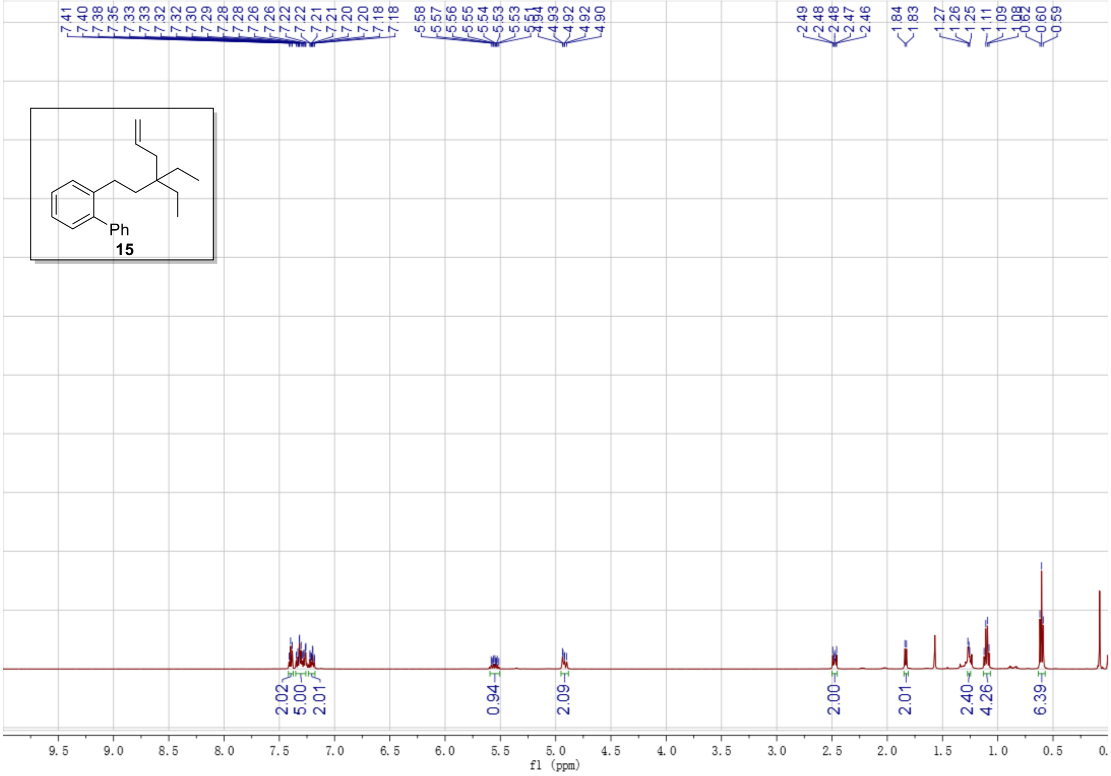

Supplementary Figure 263. ^{1}H NMR spectrum of **15**.

^{13}C spectrum (126 MHz, room temperature, $CDCl_3$) of (**15**)

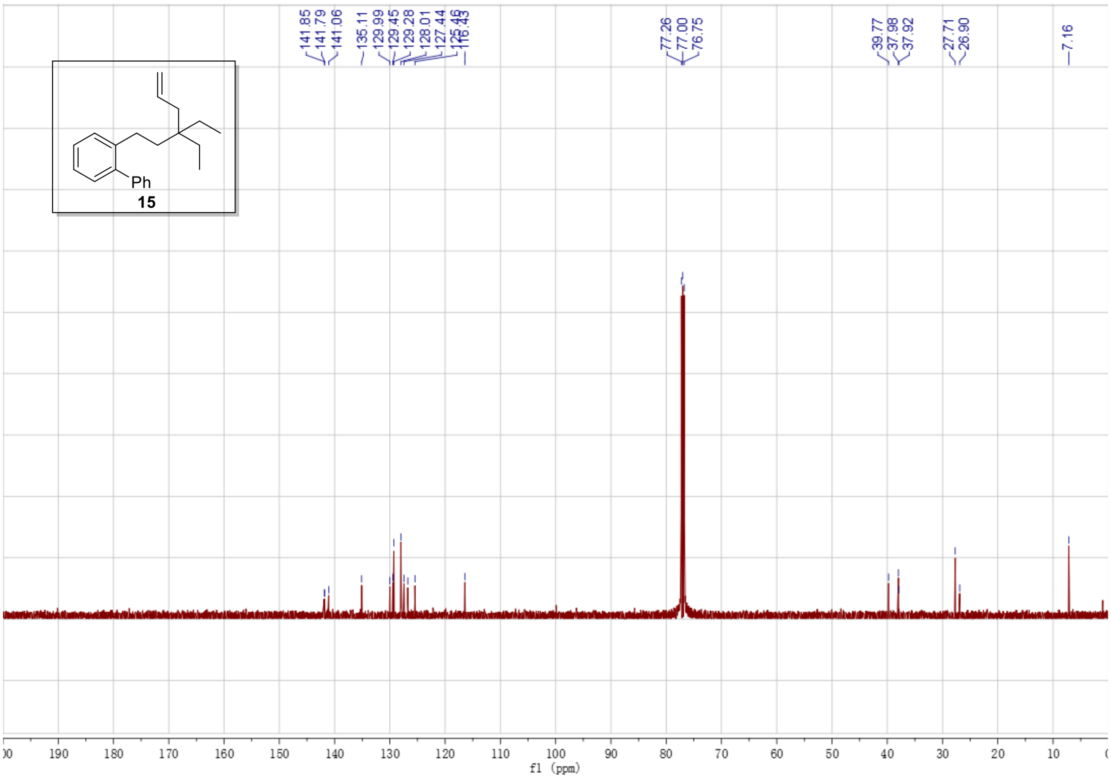

Supplementary Figure 264. ^{13}C NMR spectrum of **15**.

2-(2-(3,3-dimethylbutyl-1-d)phenyl)-4,4,5,5-tetramethyl-1,3,2-dioxaborolane (4a-D)

^{1}H spectrum (500 MHz, room temperature, $CDCl_3$) of (**4a-D**)

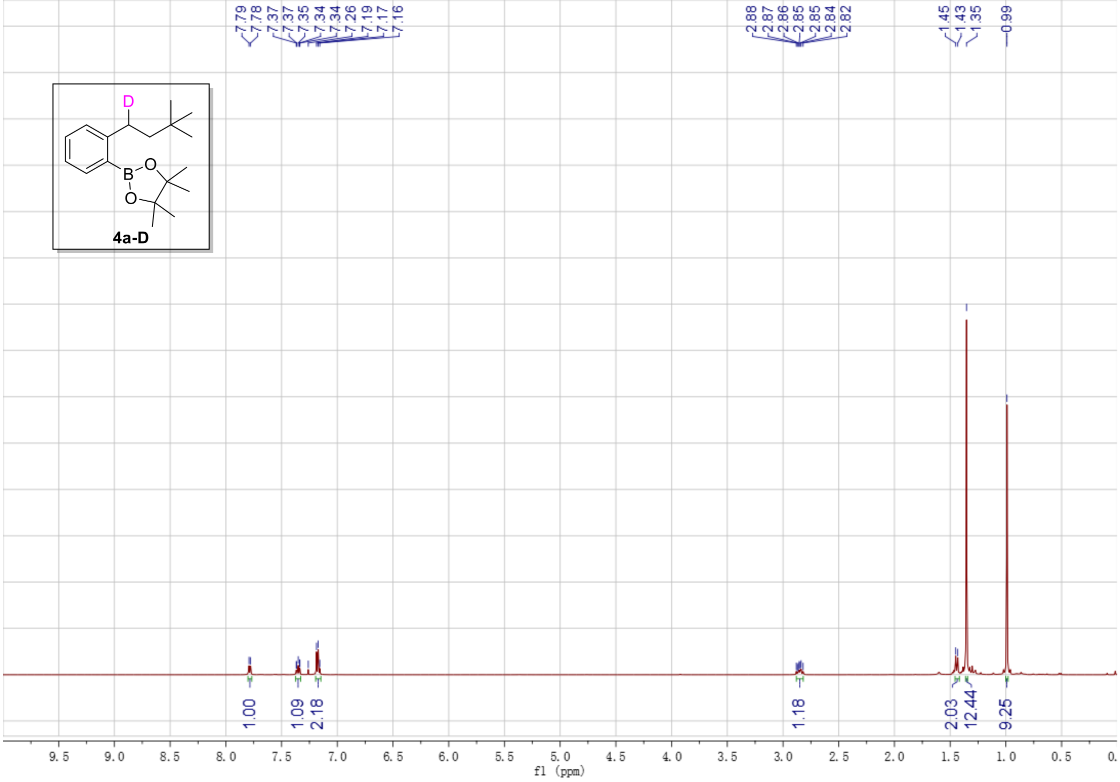

Supplementary Figure 265. ^{1}H NMR spectrum of **4a-D**.

^{13}C spectrum (126 MHz, room temperature, $CDCl_3$) of (**4a-D**)

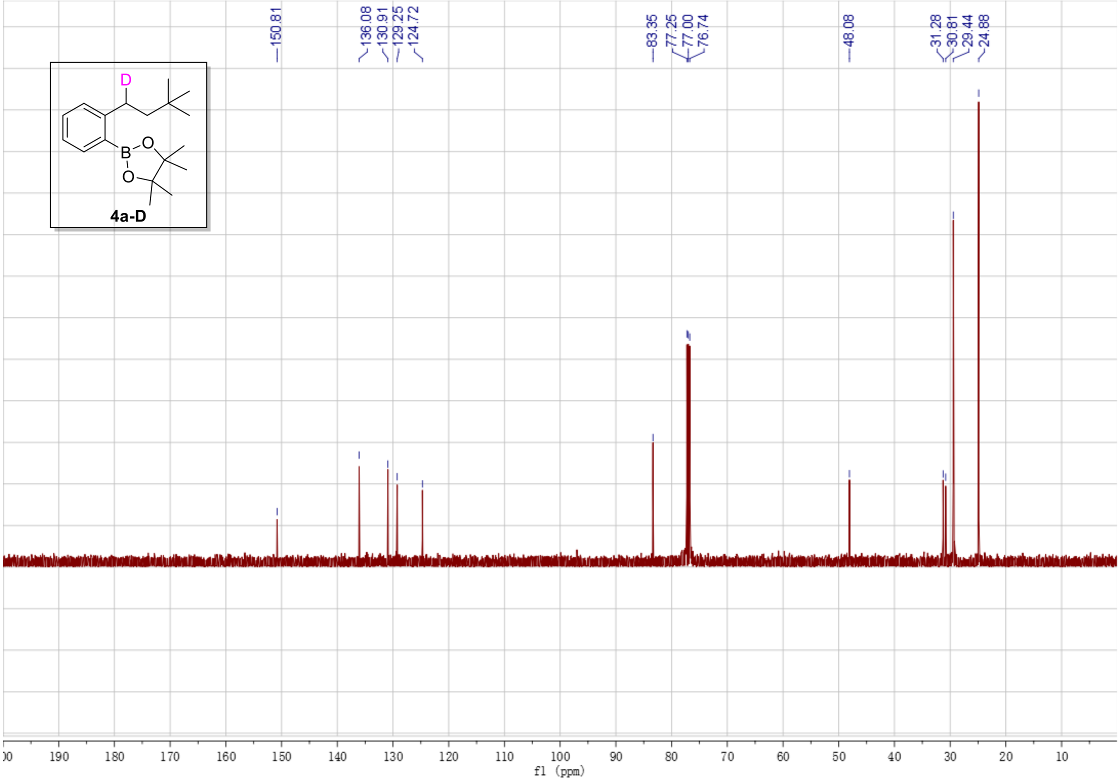

Supplementary Figure 266. ^{13}C NMR spectrum of **4a-D**.

^{11}B spectrum (160 MHz, room temperature, $CDCl_3$) of (**4a-D**)

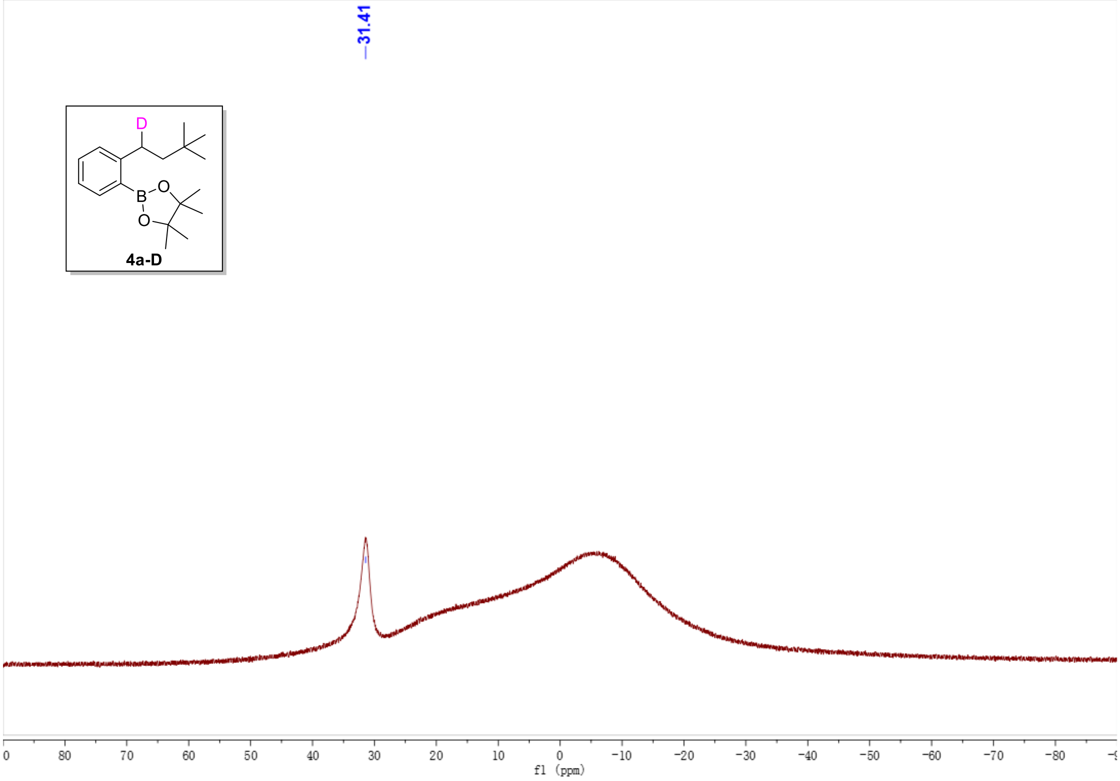

Supplementary Figure 267. ^{11}B spectrum of **4a-D**.

2.10 Supplementary References

1. Atack, T. C. & Cook, S. P. Manganese-Catalyzed Borylation of Unactivated Alkyl Chlorides. *J. Am. Chem. Soc.* **138**, 6139-6142, (2016).

2. Campbell, M. W., Compton, J. S., Kelly, C. B. & Molander, G. A. Three-Component Olefin Dicarbofunctionalization Enabled by Nickel/Photoredox Dual Catalysis. *J. Am. Chem. Soc.* **141**, 20069-20078, (2019).

3. Guo, L. et al. General Method for Enantioselective Three-Component Carboarylation of Alkenes Enabled by Visible-Light Dual Photoredox/Nickel Catalysis. *J. Am. Chem. Soc.* **142**, 20390–20399, (2020).

4. Su, W. *et al.* Ligand-Free Iron-Catalyzed Regioselectivity-Controlled Hydroboration of Aliphatic Terminal Alkenes. *ACS Catalysis* **10**, 11963-11970, (2020).

5. Farre, A. *et al.* Amine Catalysis for the Organocatalytic Diboration of Challenging Alkenes. *Chemistry - A European Journal.* **22**, 17552-17556, (2016).

6. Kaiser, D., Noble, A., Fasano, V. & Aggarwal, V. K. 1,2-Boron Shifts of beta-Boryl Radicals Generated from Bis-boronic Esters Using Photoredox Catalysis. *J. Am. Chem. Soc.* **141**, 14104-14109, (2019).

7. Li, H. et al. Formal carbon insertion of N-tosylhydrazone into B-B and B-Si bonds: *gem*-diborylation and *gem*-silylborylation of sp^3 carbon. *Org. Lett.* **16**, 448-451, (2014).

8. (a) Wang, S. *et al.* Palladium-Catalyzed anti-Selective Fluoroalkylboration of Internal and Terminal Alkynes. *Org. Lett.* **20**, 5631-5635, (2018). (b) Kuang, Z. et al. Cu-Catalyzed Regio- and Stereodivergent Chemoselective *sp/sp* 1,3- and 1,4-Diborylations of CF_3-Containing 1,3-Enynes. *Chem* **6**, 2347-2363, (2020).

9. Bonet, A., Odachowski, M., Leonori, D., Essafi, S. & Aggarwal, V. K. Enantiospecific $sp(^2)$-$sp(^3)$ coupling of secondary and tertiary boronic esters. *Nat. Chem.* **6**, 584-589, 1971 (2014).

10. Shi, D., Xia, C. & Liu, C. Photoinduced Transition-Metal-Free Alkynylation of Alkyl Pinacol Boronates. *CCS Chemistry* **3**, 1718-1728, (2021).

11. Sonawane, R. P. et al. Enantioselective construction of quaternary stereogenic centers from tertiary boronic esters: methodology and applications. *Angew. Chem. Int Ed. Engl.* **50**, 3760-3763, (2011).
